# Structural insights and evaluation of the potential impact of missense variants on the interactions of SLIT2 with ROBO1/4 in cancer progression

**DOI:** 10.1038/s41598-020-78882-2

**Published:** 2020-12-14

**Authors:** Debmalya Sengupta, Gairika Bhattacharya, Sayak Ganguli, Mainak Sengupta

**Affiliations:** 1 Department of Genetics, University of Calcutta, University College of Science (UCSTA), 35, Ballygunge Circular Road, Kolkata, 700 019 India; 2 Department of Biotechnology, St. Xavier’s College (Autonomous), 30, Mother Teresa Sarani, Kolkata, 700 016 India; 3 Cactus Communications, Mumbai, India

**Keywords:** Data acquisition, Data integration, Data mining, Data processing, Literature mining, Protein analysis, Protein function predictions, Protein structure predictions, Sequence annotation

## Abstract

The cognate interaction of ROBO1/4 with its ligand SLIT2 is known to be involved in lung cancer progression. However, the precise role of genetic variants, disrupting the molecular interactions is less understood. All cancer-associated missense variants of ROBO1/4 and SLIT2 from COSMIC were screened for their pathogenicity. Homology modelling was done in Modeller 9.17, followed by molecular simulation in GROMACS. Rigid docking was performed for the cognate partners in PatchDock with refinement in HADDOCK server. Post-docking alterations in conformational, stoichiometric, as well as structural parameters, were assessed. The disruptive variants were ranked using a weighted scoring scheme. In silico prioritisation of 825 variants revealed 379 to be potentially pathogenic out of which, about 12% of the variants, i.e. ROBO1 (14), ROBO4 (8), and SLIT2 (23) altered the cognate docking. Six variants of ROBO1 and 5 variants of ROBO4 were identified as "high disruptors" of interactions with SLIT2 wild type. Likewise, 17 and 13 variants of SLIT2 were found to be "high disruptors" of its interaction with ROBO1 and ROBO4, respectively. Our study is the first report on the impact of cancer-associated missense variants on ROBO1/4 and SLIT2 interactions that might be the drivers of lung cancer progression.

## Introduction

Cancer morbidity is mainly attributed to metastasis^1,2^, where the invasion and migration of tumour cells are critical phenomena. However, the pathways that regulate metastasis are still unclear, which acts as a deterrent for an introduction of therapeutic modalities. Recent researches indicated that the SLIT-ROBO pathway also plays a critical role in the modulation of chemokine activation and cellular migration of different lineages^3–6^. The SLIT family of proteins docks to Roundabout (ROBO) receptors and modulate morphogenesis^7^, leukocyte chemotaxis^4^, and most importantly, metastasis and angiogenesis^8,9^. SLIT2 is the most studied member of the SLIT family that has been found to exert a tumour suppressive activity, mainly by binding to the ROBO1 receptor^10^. Strong evidence suggests that SLIT/ROBO signalling requires heparan sulfate proteoglycans (HSPGs) as a co-receptor^11–13^. HSPGs consist of a large proteoglycan core protein decorated with heparan sulfate chains, which stabilises the interaction between ROBO1 and SLIT2^11–13^. The recognition of *SLIT2* by *ROBO1* is mediated through proper interaction of the respective domains, such as *SLIT2.D2*^14^ and *ROBO1.IG1* that, again, is facilitated by heparan sulfate units^11–13^. Less is known regarding ROBO4 and SLIT2 interaction.

The SLIT/ROBO signalling pathway has anti-migratory and pro-apoptotic properties in many cancers^15^, such as Small Cell Lung Cancer (SCLC). These tumour-suppressive effects could be weakened by mutations^16^ within the cognate partners and its mediators. Robo1 knockout mice exhibited pulmonary dysmorphogenesis that resulted in embryonic death in most cases, while the surviving ones showed bronchial hyperplasia^17^. The SLIT2-ROBO1 pathway was found to counteract HGF-mediated tumour cell migration by inhibiting CDC-42 and stimulating RAC-1^7^. Recent studies have indicated the possible anticancer attributes of SLIT2-ROBO1 signalling^18^ pathway in tumour cell migration and angiogenesis in lung cancer^19^, breast cancer^20^, gastric cancer^21^, and colorectal cancer^22^. For instance, the *SLIT2* gene is inactivated in multiple cancers^23–25^, including lung cancer^26^, often as a result of promoter hypermethylation, mutations (frequency of occurrence ~ 10%) and LOH^26,27^. Recent investigations have shown the role of Myo9b-RhoA in mediating the inhibitory effect of SLIT2 on lung cancer cell migration^28^.

Similarly, the downregulation of USP33 (a ubiquitin ligase enzyme) reduces the stability of the ROBO1 receptor in lung cancer cells^29^ and breast cancer cell migration^30^. Interestingly, ROBO1 was found to be a specific serum biomarker in SCLC^31^. Thus, it is well-established that the *SLIT2-ROBO1* signalling pathway is tumour suppressive in many cancers, including lung cancer^26,28,29^. The anti-angiogenic effect of ROBO4 is also well documented^8,32^. Increased ROBO4 expression has been correlated with increased overall survival in early-stage non-small cell lung cancer^33^. However, the role of the genetic variants on the interaction of SLIT2/ROBO1/4, potentially altering the anti-angiogenic pathway and overall tumour suppressive function, is yet to be elucidated.

This work aims at the in silico prioritisation, and pathogenicity assessment of the missense variants of SLIT2/ROBO1/4 reported in lung cancer cases, based on sequence- and structure-based parameters followed by docking analyses to determine the interaction dynamics. We hypothesise that the variants would be responsible for the disruption of effective receptor-ligand docking and could result in the alteration of its tumour suppressive function within the pulmonary cells leading to lung cancer metastasis. These variants either individually or in combination^34^ could act as docking disruptors by inducing conformational changes, like fluctuations in the secondary structural elements, alterations in solvent accessible surface area or solvation free energy, number of hydrogen bonds, number of salt bridges, partial unfolding or displacement of helices, any altered local/global flexibility and docking pose as compared to the wild-type proteins. It is worth mentioning that in this work, the missense variants reported in other cancers were also taken into consideration because some of them could also be found in lung cancer with the incorporation of more sample data using advanced sequencing technologies. For instance, p.R119Q (Arg119Glu) is reported in both colorectal cancer and lung cancer. Likewise, more variants reported in other cancers could be found in lung cancer with further studies.

## Methods

### Data mining

Somatic variants of *ROBO1*, *ROBO4*, and *SLIT2* were curated from the "Catalogue Of Somatic Mutations In Cancer" (COSMIC, release v73; http://cancer.sanger.ac.uk/cosmic) database^35^. The canonical sequences for ROBO1 (UniProt sequence ID: Q9Y6N7-1), ROBO4 (UniProt sequence ID: Q8WZ75-1) and SLIT2 (UniProt sequence ID: O94813-1) were curated from UniProt (https://www.uniprot.org/)^36,37^ and the secondary structures were predicted using PSIPRED (Position Specific Iterated BLAST based secondary structure PREDiction) (http://bioinf.cs.ucl.ac.uk/psipred/) server^38^.

### Identification of the degree of disorder in the proteins

The IUPred2A (Intrinsically Unstructured protein Prediction 2A) (https://iupred2a.elte.hu/)^39^ analysis was done to understand the presence or absence of disordered or intrinsically unstructured regions in the context of the amino acid sequences. The presence of Intrinsically Unstructured Regions (IUR) tends to add a degree of randomness to the native conformation of the protein structures^40^.

### Variant prioritisation by sequence-specific parameters

**Figure 1:**
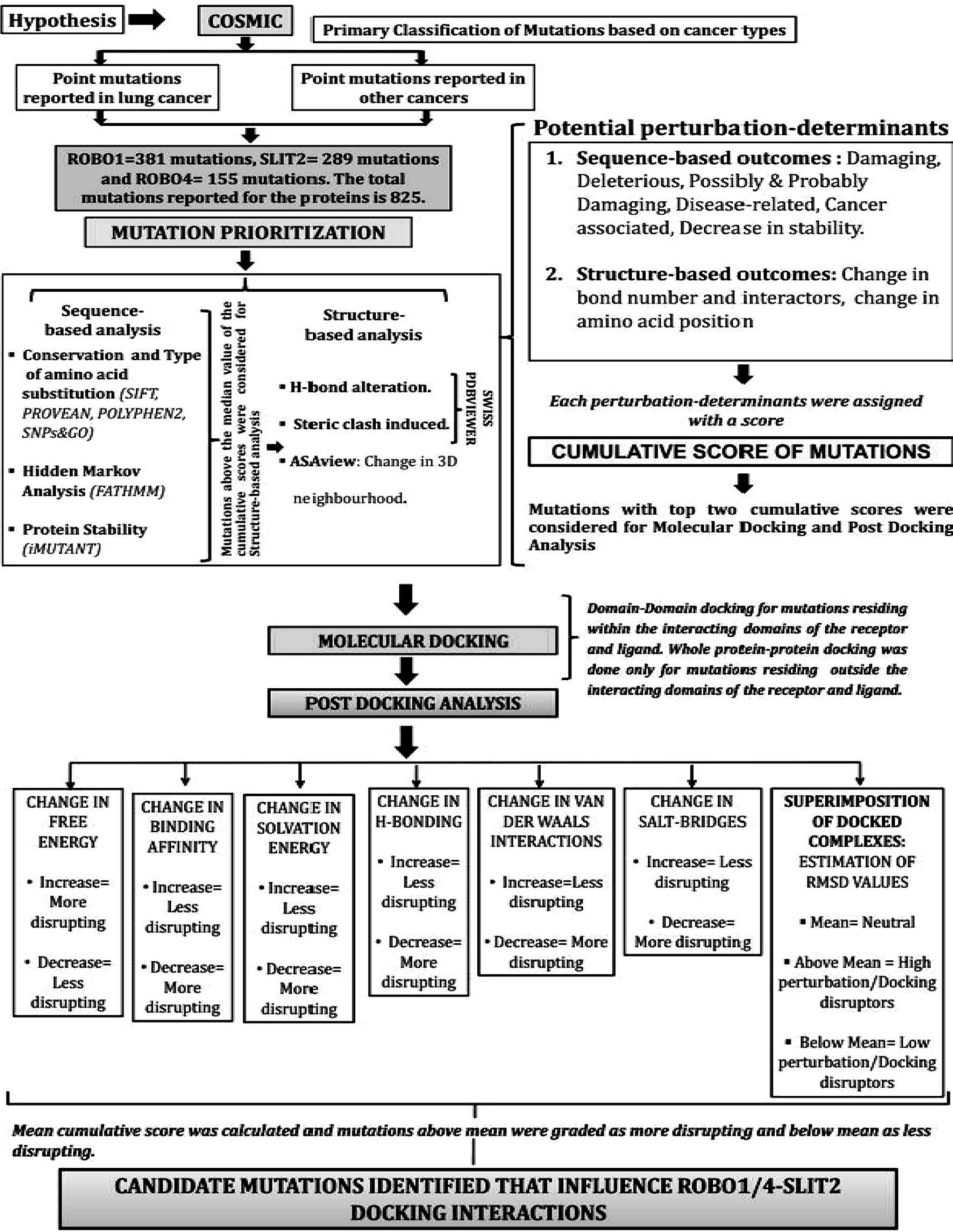
Schema of analysis. The work includes data mining followed by variant prioritisation and molecular docking for identification of candidate molecular targets as potential disruptors of ROBO/SLIT signalling mediated by ROBO1/SLIT2 and ROBO4/SLIT2.

The variants obtained from data mining were subjected to an in silico prioritisation pipeline for their pathogenicity determination by sequence-based and structure-based analysis (Fig. 1). In Silico analysis of all the variants (categorised as "*lung cancer-associated missense variants"* and *"missense variants associated with other cancers")* were done by PROVEAN (Protein Variation Effect Analyzer) (http://provean.jcvi.org/index.php)^41^, which predicted whether amino acid substitutions or indels are "deleterious" or "Neutral". The PROVEAN web server also includes the algorithm of SIFT (Sorting Intolerant From Tolerant)^42^ that predicted whether a variant is "damaging" (SIFT Score ≤ 0.05) or "tolerated" (SIFT Score > 0.05) based on sequence homology and the physical properties of amino acids. The same set of variants were analysed in *PolyPhen2* (Polymorphism Phenotyping v2)^43^ (http://genetics.bwh.harvard.edu/pph2/) that predicted the functional significance of an amino acid substitution as "Probably damaging", "Possibly damaging" or "Benign". The entire set of variants were further analysed in SNPs&GO (Single Nucleotide Polymorphisms & Gene Ontology) (https://snps-and-go.biocomp.unibo.it/snps-and-go/index.html)^44^, which predicted whether the variant is associated with "Disease" or not. Further, iMUTANT 2.0 (http://folding.biofold.org/i-mutant/i-mutant2.0.html)^45 ^predicted whether the variants "increases" or "decreases" the stability of the protein structures calculated from the input protein sequence. Finally, the variants were analysed in FATHMM (Functional Analysis Through Hidden Markov Models) (http://fathmm.biocompute.org.uk/)^46^, which predicted whether a protein variant is "cancer-associated" or a "passenger" variant by combining sequence conservation within hidden Markov models (HMMs). The scoring scheme followed for sequence-based variant prioritisation is depicted in (Table 1). A weighted score was applied to the outcomes of each prediction from the softwares. More weightage was given to the outcome, depicting the disruptive/damaging nature of the mutations and was assigned with higher scores while the benign/neutral or tolerated mutations were given less weightage with lower scores. For example, p.R119Q (ROBO1.IG1) is a “*deleterious*” mutation according to PROVEAN and was assigned with score 1 while p.D930N (SLIT2) is a “*neutral*” mutation and was assigned with score 0. The cumulative score for each variant was calculated for the sequence-based analysis, and the variants with the cumulative score above the median value of the distribution of cumulative scores of all the variants were selected for further structure-based variant prioritisation.

**Table 1:** The scoring scheme for the prioritisation of the variants of ROBO1, ROBO4, and SLIT2. Each of the predictive outcomes is assessed by comparing with the wild type protein sequence or structure.

Webservers	Predictive outcome for the mutants	Score
(**A**) **Sequence-based analysis**		
Provean	Neutral	0
	Deleterious	1
SIFT	Tolerated	0
	Damaging	1
Polyphen2	Benign	0
	Possibly Damaging	1
	Probably Damaging	2
SNPs&GO	Neutral	0
	Disease-related	1
iMUTANT 2.0	Increase Stability	0
	Decrease Stability	1
FATHMM	Passenger Variants	0
	Cancer-associated Variants	1
(**B**) **Structure-based analysis**		
H-bond	No Change compared to Wild Type (in any of the features like Bond Number, Bond Length, Interacting residues)	0
	Change compared to Wild Type (in any of the features like Bond Number, Bond Length, Interacting residues)	1
Steric clash	No Change compared to Wild Type	0
	Change compared to Wild Type	1
Asaview	The mutant residue has no positional alteration compared to the wild type	0
	The mutant residue shifts its position compared to the wild type	1

### Generation of Homology models for the wild type proteins

Complete crystal structure of the proteins was not available in the Protein Data Bank. Therefore, homology models were generated to study the alterations in the structural dynamics of ROBO1/SLIT2 and ROBO4/SLIT2 interactions based on variants. Once the secondary structures were obtained from PSIPRED^38^, the sequences were subjected to BLAST analyses (https://blast.ncbi.nlm.nih.gov/Blast.cgi )^47,48^ using the Protein Data Bank (https://www.rcsb.org/)^49^as the target database, to identify suitable homologous templates. The results showed four suitable templates each for ROBO1 (2V9Q, 5NOI, 2EO9, and 3WIH), ROBO4 (2V9Q, 2VRA, 3PUC, and 2VR9) and SLIT2 (2V70, 2V9T, 2V9S, and 2WFH). Each of the proteins and their corresponding interaction domains was modelled separately by comparative homology modelling using Modeller 9.17^50^, and the modelled structures were then subjected to molecular simulation for 40 ns using a GROMOS96 43a2 (GROningen MOlecular Simulation) force field in GROMACS (GROningen MAChine for Chemical Simulation)^51,52^. Following the simulation, the structures were analysed for torsional/ conformational stability from the Ramachandran plots of the models using the MolProbity server^53^. The Q–MEAN score verified the integrity and validity of the 3D structures of the generated homology models.

### Generation of Mutant Structures from wild type homology models

The variants prioritised by the sequence-based analysis were inserted within the wild type homology models of ROBO1, ROBO4 and SLIT2 by using the MUTATE tool of DeepView v4.1.0^54^. The mutant proteins were subjected to the simulation as mentioned earlier conditions and validation protocol to generate the final mutated homology models of the proteins.

### Structural analyses of the mutant models

The mutant models were assessed for alterations in the pattern of hydrogen bond interactions (in terms of the interacting residues), bond length and also in steric clashes between residues in comparison to their corresponding wild type structures (Fig. 1). Deviation of the loco-regional status of the mutable amino acid residues of mutant ROBO1, ROBO4, and SLIT2 compared to their wild types were analysed in terms of the Accessible Surface Area (ASA) or Solvent Accessibility (SA) in a webserver called ASAview (http://ccbb.jnu.ac.in/shandar/servers/asaview/ )^55^. The scoring scheme followed for assigning a score to each outcome of the structure-based variant prioritisation is depicted in (Table 1).

A cumulative score for each variant (i.e. variants selected after sequence-based analysis) was calculated, and the variants with top two cumulative scores were selected for the subsequent docking analysis. The selection criteria were kept similar for variants residing within the docking domains and outside the docking domains, belonging to both lung cancer and non-lung cancer datasets.

After the prioritisation of the variants, alterations in potential ligand-binding hotspots, due to variants in ROBO1, ROBO4 and SLIT2 proteins were analysed in DoGSiteScorer (https://proteins.plus/) webserver^56^. This analysis was not used for prioritisation of the variants, but to assess the change in the respective structural attributes based on the variants finally selected for docking. The following parameters were assessed;

- *Drug score* The average drug score of all the pockets for both wild types and mutants was calculated. The mean drug score of wild type structures was annotated as '*Neutral*'. The mean drug score of the mutants equal to the corresponding wild type was also annotated as '*Neutral*'. Annotation '*Favourable*' was assigned if the mean drug score of the mutant is more than its wild type. If the mean drug score of the mutants is less than the corresponding wild type, annotation '*Unfavourable*' was assigned. This parameter depicts ligand (i.e., drugs, small molecules) binding capability of the structure, which could be considered as therapeutic targets.
- *The number of ligand-binding pockets* The number of ligand-binding pockets in the wild type structure was considered as the reference and was annotated as '*Neutral*'. Gain in pockets upon variant is considered as '*Increase*' while the loss of pockets upon variant was termed as '*Decrease*'.

### Molecular docking

Rigid docking of the whole proteins and also the interacting domains of the same was done using PatchDock (https://bioinfo3d.cs.tau.ac.il/PatchDock/) server^57^. The free energy of interaction of the wild type (native) and mutant complexes in the form of atomic contact energy based on shape complementarity principles was obtained from PatchDock and validated by using HADDOCK (High Ambiguity Driven protein–protein DOCKing) (https://haddock.science.uu.nl/) server^58,59^. The molecular docking was elaborately performed as a wild type to wild type and mutant to wild type proteins/domains. The best docking pose was selected based on the global energy of the docked complex and the biological relevance concerning the dynamics of the interaction between the membrane-bound receptor to its extracellular ligand.

### Analyses of docked complexes

The mutant-to-wild type complexes were then analysed for their residue-specific interactions using PDBsum (http://www.ebi.ac.uk/thornton-srv/databases/pdbsum/Generate.html) server^60^ and were compared with wild type-to-wild type docked complexes for changes in the bond number.

Change in the binding affinity (ΔΔG_Bind_ kcal/mol) due to variants were also checked for mutant-to-wild type complexes about wild type-to-wild type complexes in a web server named BeAtMuSiC v1.0 (prediction of Binding Affinity Changes upon MutationS) (http://babylone.ulb.ac.be/beatmusic/index.php)^61^.

Similarly, the change in free energy (ΔG kcal/mol) due to variants was also checked for mutant-to-wild type complexes about wild type-to-wild type complexes during docking. Further, gain in solvation energy upon complex formation, for mutant-to-wild type docked complexes about wild type-to-wild type docked complexes, were assessed in PDBePISA (Protein Data Bank in Europe Proteins, Interfaces, Structures and Assemblies) webserver (http://www.ebi.ac.uk /pdbe/pisa/)^62^.

A weighted scoring scheme was followed for assigning a score to each outcome of the post-docking analysis (Table 2).

**Table 2:** Weighted scoring scheme for the docked complexes for the variants of ROBO1, ROBO4, and SLIT2. Each of the predictive outcomes is assessed by comparing with the wild type to wild type docked complex.

Parameters	Predictive outcome for the mutants	Weighted score
(**A**) **Post docking analysis**		
Changes in free energy (ΔG)	No change compared to wild type complex	0
	More negative compared to wild type complex	1
	Less negative compared to wild type complex	2
Changes in binding affinity (ΔΔG_Bind_)	No change compared to wild type complex	0
	More negative compared to wild type complex	1
	Less negative compared to wild type complex	2
Gain in solvation energy (Δ^i^G)	No change compared to Wild Type complex	0
	More negative compared to wild type complex	1
	Less negative compared to wild type complex	2
Change in h-bond number	No change compared to wild type complex	0
	Increased compared to wild type complex	1
	Decreased compared to wild type complex	2
Change in van der waals interactions	No change compared to wild type complex	0
	Increased compared to wild type complex	1
	Decreased compared to wild type complex	2
Change in the number of salt-bridges	No Change Compared To Wild Type complex	0
	Increased compared to wild type complex	1
	Decreased compared to wild type complex	2
Super-imposition (mean RMSD)	RMSD of the mutant docked complex is equal to the mean RMSD	0
	RMSD of the mutant docked complex is lesser than the mean RMSD	1
	RMSD of the mutant docked complex is higher than the mean RMSD	2

### Analyses of structural distortion

The mutant-to-wild type complexes were superimposed upon wild type-to-wild type complexes using *Matchmaker* tool of UCSF-Chimera 1.14rc^63^ where the two docked complexes were subjected to pairwise alignment per residue to identify non-alignment of the superimposed structures at the residue per se or for the overall conformation of the interacting partners. The degree of conformational distortion of the mutant complex compared to the wild type was assessed by the magnitude of the RMSD (Root Mean Square Deviation) values. The mean RMSD value for all the complexes was estimated, and the scoring of the variants was done (Table 2).

#### Cumulative score and grading of variants from docked complex analysis

Following the weighted scoring scheme as depicted in (Table 2), a cumulative score was estimated for each docked complex by merely summing the individual weighted score of each of the parameters analysed post docking. It was followed by the calculation of the mean of the cumulative score distribution of all mutant complexes for the respective docking categories, i.e. ROBO1.IG1:SLIT2.D2, ROBO4.IG1-2:SLIT2.D2, ROBO1:SLIT2, and ROBO4:SLIT2. The cumulative score of the wild type:wild type docked complexes was denoted as '0′. A variant was graded as *"More disrupting*" when its cumulative score was more than the mean of the cumulative scores of all the mutant complexes of a specific docking category and "*Less disrupting*" when its cumulative score was less than the mean of the cumulative scores of all the mutant complex for a specific docking category as mentioned above.

## Results

### Curation and categorisation of somatic variants

Systematic mining of the COSMIC^35^ database (release v73) for somatic missense variants revealed 381 variants in ROBO1, 289 variants in SLIT2 and 155 variants in ROBO4. Out of 825 variants; 34 in ROBO1, 57 in SLIT2, and 34 in ROBO4 were reported to be associated with lung cancer. Thus, these were categorised as *lung cancer dataset*. In comparison, the other 255 variants in ROBO1, 324 variants in SLIT2 and 121 variants in ROBO4 were considered as *non-lung cancer dataset*.

### Prioritisation of variants on sequence-based and structure-based parameters

Prioritisation of the variants was done according to our predefined pipeline, as shown in (Fig. 1). The variants were analysed in PROVEAN^41^ that predicted nearly 50% of ROBO1, 29% of ROBO4, and 63% of SLIT2 variants of lung cancer dataset to be "deleterious". Similarly, 40% of ROBO1, 18% of ROBO4, and 56% of SLIT2 variants of non-lung cancer dataset were predicted to be "deleterious" by PROVEAN.

Similarly, analysis in SIFT^42^ revealed about 65% of ROBO1, 47% of ROBO4, and 60% of SLIT2 variants of lung cancer dataset to be "damaging". About 61% of ROBO1, 45% of ROBO4, and 61% of SLIT2 variants of non-lung cancer dataset were predicted to be "damaging" by SIFT.

*PolyPhen2*^43^ predicted about 15% of ROBO1, 15% of ROBO4, and 23% of SLIT2 variants of lung cancer dataset to be "possibly damaging". About 13% of ROBO1, 13% of ROBO4, and 19% of SLIT2 variants of non-lung cancer dataset were predicted to be "possibly damaging" by *PolyPhen2*. Similarly, about 53% of ROBO1, 50% of ROBO4, and 46% of SLIT2 variants of lung cancer dataset to be "possibly damaging", while 45% of ROBO1, 50% of ROBO4, and 43% of SLIT2 variants of non-lung cancer dataset were predicted to be "probably damaging" by *PolyPhen2*. *SNPs&GO*^44^ predicted about 35% of ROBO1, 35% of ROBO4, and 32% of SLIT2 variants of lung cancer dataset to be "Disease-related". About 25% of ROBO1, 25% of ROBO4, and 35% of SLIT2 variants of non-lung cancer dataset were predicted to be "Disease-related" by *SNPs&GO*.

Interestingly, iMUTANT 2.0 revealed 91% of ROBO1, 79% of ROBO4, and 84% of SLIT2 variants of lung cancer dataset to cause a "decrease" in protein stability. About 89% of ROBO1, 76% of ROBO4, and 83% of SLIT2 variants of non-lung cancer dataset were predicted to cause a "decrease" in protein stability.

Amino acid sequence-based predictions from FATHMM^46^ using hidden Markov Models revealed 0% of ROBO1, 6% of ROBO4, and 0% of SLIT2 variants of lung cancer dataset to be associated with some form of "cancer". About, 0.4% of ROBO1, 0.8% of ROBO4, and 2% of SLIT2 variants of non-lung cancer dataset were predicted to be associated with "cancer" by FATHMM.

The cumulative score of all sequence-based parameters per variant was calculated (Table 1). The variants with a cumulative score > 3 (median value) of the distribution of the cumulative scores, were selected for further structure-based analysis. Thus, from the lung cancer dataset, 18 variants of ROBO1, 29 variants of SLIT2 and 13 variants of ROBO4 were selected for structure-based analysis. From the non-lung cancer dataset, 113 variants of ROBO1, 134 variants of SLIT2 and 41 variants of ROBO4 were selected for structure-based analysis. The detailed results of the final set of prioritised variants are summarised in the following table (Table 3). The list of all variants of the three proteins with sequence-based scores is summarised in (Supplementary Information, Table S1).

**Table 3:** The final set of in silico prioritised variants by sequence-based and structure-based parameters.

Genes	Variants	PROVEAN	SIFT	Polyphen2	SNPs&GO	iMUTANT 2.0	FATHMM	H-bond change	Steric clash change	ASA	Total score
(**A**) **Lung cancer dataset**											
(I) Variants within docking domain											
ROBO1	p.R119Q	1	1	2	1	1	0	1	0	1	8
ROBO1	p.K103N	1	1	2	1	1	0	1	0	1	8
SLIT2	p.D435V	1	1	2	1	1	0	1	1	1	9
SLIT2	p.R306L	1	1	2	1	1	0	1	1	1	9
SLIT2	p.Y323H	1	0	2	1	1	0	1	1	1	8
ROBO4	p.G102C	1	1	2	0	1	0	1	1	1	8
ROBO4	p.C207S	1	1	2	1	1	1	1	0	0	8
(II) Variants outside docking domain											
ROBO1	p.R635S	1	1	2	1	1	0	1	1	1	9
ROBO1	p.D312V	1	1	2	1	1	0	1	1	1	9
ROBO1	p.R306I	1	1	2	1	1	0	1	1	1	9
SLIT2	p.P54R	1	1	2	1	1	0	1	1	1	9
SLIT2	p.P665S	1	1	2	1	1	0	1	1	0	8
ROBO4	p.G829W	1	1	2	1	1	0	1	1	1	9
ROBO4	p.W367C	1	1	2	1	1	1	1	0	1	9
(**B**) **Non-lung cancer dataset**											
(I) Variants within docking domain											
ROBO1	p.I78S	1	1	2	1	1	0	1	0	1	8
ROBO1	p.G82V	1	1	2	1	1	0	1	0	1	8
ROBO1	p.P123L	1	1	2	1	1	0	1	1	0	8
ROBO1	p.R131C	1	1	2	1	1	0	1	0	1	8
ROBO1	p.G143R	1	1	2	1	1	0	1	0	1	8
ROBO1	p.G154E	1	1	2	1	1	0	1	0	1	8
SLIT2	p.C436F	1	1	2	1	1	0	1	1	1	9
(**B**) **Non-lung cancer dataset**											
(I) Variants within docking domain											
SLIT2	p.R388W	1	1	2	1	1	0	1	1	1	9
SLIT2	p.R456H	1	1	2	1	1	0	1	1	1	9
SLIT2	p.N430K	1	1	2	1	1	0	1	1	1	9
SLIT2	p.R420W	1	1	2	1	1	0	0	1	1	8
SLIT2	p.L350V	1	1	2	1	1	0	0	1	1	8
SLIT2	p.P431L	1	1	2	1	1	0	1	1	1	9
SLIT2	p.L331P	1	1	2	1	1	0	1	1	0	8
SLIT2	p.L398F	1	1	2	1	1	0	1	1	0	8
SLIT2	p.L371P	1	1	2	1	0	0	1	1	1	8
SLIT2	p.S352L	1	1	2	1	1	0	1	1	1	9
SLIT2	p.R462C	1	1	2	1	1	0	1	1	1	9
SLIT2	p.A340T	1	1	2	0	1	0	1	1	1	8
SLIT2	p.P322L	1	1	2	0	1	0	1	1	1	8
SLIT2	p.R461C	1	1	2	1	1	0	1	0	1	8
ROBO4	p.G86E	1	1	2	1	0	0	1	1	1	8
ROBO4	p.C207F	1	1	2	1	1	1	0	1	1	9
(II) Variants outside docking domain											
ROBO1	p.P176L	1	1	2	1	1	0	1	1	1	9
ROBO1	p.G337V	1	1	2	1	1	0	1	1	0	8
ROBO1	p.C476Y	1	1	2	1	1	0	1	1	0	8
SLIT2	p.C215Y	1	1	2	1	0	1	1	1	0	8
SLIT2	p.G576R	1	1	2	1	1	0	1	1	0	8
SLIT2	p.S636C	1	1	2	1	1	0	1	0	1	8
ROBO4	p.G425V	1	1	2	1	1	0	1	1	0	8
ROBO4	p.G876V	1	1	2	1	1	0	1	1	0	8

The generation of mutant homology models as described in the Materials and Methods section and structural analysis of the mutants versus their respective wild types revealed variant-induced alteration in local hydrogen bond number and bond length among the interacting amino acid residues for the variants; 10 in ROBO1, 10 in ROBO4 and 14 in SLIT2 from lung cancer dataset. Similarly, for non-lung cancer dataset, the variants conferring change in local hydrogen bond number and bond length are 50 in ROBO1, 31 in ROBO4, and 89 in SLIT2. Steric clash-induced destabilisations, primarily found in variants involving bulky amino acids were 7 in ROBO1, 5 in ROBO4 and 9 in SLIT2 from the lung cancer dataset. In comparison to, 9 variants in ROBO1, variants in ROBO4, and 34 variants from the non-lung cancer dataset were found. The representative images of variant-induced changes for both the changes in H-bond and steric clashes compared to wild types are depicted in (Supplementary Information, Fig. S1). The detailed results of variant-specific alterations in local H-bond (number, length, interacting residues) and steric clash in the protein structures for the final set of prioritised variants are summarised in the following tables (Table 3, Supplementary Information, Table S2).

Analysis of the mutant structures of all the interacting domains of the 3 proteins in ASAview revealed 1 variant of SLIT2.D2 exhibit an inter-orbit shift in the position of the mutant amino acid residue compared to wild type structure in the lung cancer dataset. Similarly, in the non-lung cancer dataset, 5 variants of ROBO1.IG1, 7 variants of SLIT2.D2 and 2 variants of ROBO4.IG1-2 domains show an inter-orbit shift of mutant amino acid compared to wild types. However, 2 variants of ROBO1.IG1, 2 variants of SLIT2.D2 and 1 variant of ROBO4.IG1-2 domains in lung cancer dataset and 6 variants in SLIT2.D2 in the non-lung cancer dataset exhibits an intra-orbit amino acid position shift compared to wild types.

Similarly, for outside the docking domains, 3 variants of ROBO1 and 2 variants of ROBO4 show inter-orbital shift of mutant amino acid residue compared to wild type residue in the lung cancer dataset. In the non-lung cancer dataset, 1 variant of ROBO1 show inter-orbital shift of mutant amino acid residue compared to wild type residue. Only 1 variant of SLIT2 of lung cancer dataset show intra-orbital shift of mutant amino acid residue compared to wild type residue. The remaining variants do not have any impact on the change in the amino acid position compared to the wild type within the 3D protein structure. The representative images of the spiral plots are depicted in (Supplementary Information, Fig. S2), and the results of the final set of prioritised variants with their respective scores are summarised in (Table 3).

Finally, for the interacting domains, the prioritisation analysis revealed 2 variants of ROBO1, 2 variants of ROBO4 and 3 variants of SLIT2 in lung cancer dataset and 6 variants of ROBO1, 2 variants of ROBO4 and 15 variants of SLIT2 from the non-lung cancer dataset as depicted in (Table 3). These variants were further analysed for their effect on the domain-domain interactions. Similarly, for full protein–protein interaction involving variants within the non-interacting regions of the proteins; 3 variants of ROBO1, 2 variants of SLIT2 and 2 variants of ROBO4 from lung cancer dataset and 3 variants of ROBO1, 3 variants of SLIT2, and 2 variants of ROBO4 were prioritised for further docking and post-docking analyses. The results of the final set of prioritised variants are depicted in (Table 3).

### Structure validation and analysis

The sequence-based analysis of the wild type models for residue-specific IURs from IuPred2A indicates that 35.5% (38 disordered/69 ordered) residues of ROBO1.IG1, 59.6% (112 disordered/76 ordered) residues of ROBO4.IG1-2, and 0% (0 disordered/210 ordered) residues of SLIT2.D2 to be intrinsically unstructured (Supplementary Information, Fig. S2, Table S2). Similarly, 51.3% (847 disordered/804 ordered) residues of ROBO1, 45.4% (457 disordered/550 ordered) residues of ROBO4, and 0.005% (07 disordered/1522 ordered) residues of SLIT2 whole protein models, to be intrinsically unstructured (Supplementary Information, Fig. S3, Table S2). These analyses have led to the rationalisation of the Ramachandran plots and Q-mean validation outcomes, which indicate a high level of structural dynamism in protein conformation related to their function. The unstructured residues identified in the analyses may contribute towards the flexibility of the protein backbone and adjoining side chains to allow interaction with their cognate partners. It may also explain the role of HSPGs as a stabiliser of the interaction between ROBOs and SLITs.

The wild types and prioritised mutants of the generated homology models were assessed for their conformational stability in 3D by their respective Ramachandran plots and Q-mean (https:// swissmodel.expasy.org/qmean/) scores. The individual Q-mean scores of all the wild types and the mutants of both partial interacting domains and full protein models were analysed and are graphically represented in (Supplementary Information, Fig. S4, Table S3). Further, analysis of conformational stability by Ramachandran plots of the representative top-scoring variants of the interacting domains and whole protein models along with their respective wild types, are depicted in (Supplementary Information, Fig. S5). The percentage of amino acid residues in the allowed regions and the percentage of amino acids favoured in the Ramachandran plot assessed the stability of the protein structures.

Further, analysis of the effect of variants on the ligand/small molecule/drug binding capability of the proteins in DoGSiteScorer and the changes in the number of ligand binding pockets upon mutations is depicted in (Supplementary Information, Table S4). Representative images of the impact of variants on drug/ligand binding pockets in the proteins are depicted in (Supplementary Information, Fig. S6).

### Molecular docking and post-docking analysis

Mutant-to-wild type molecular docking of the receptors (ROBO1 and ROBO4) and ligand (SLIT2) was performed to assess the effect of variants on the protein–protein interaction. Several parameters that influence the docking efficiency, such as '*Free energy change*'^64^, '*Change in Binding affinity*', '*Gain in solvation energy*', '*Change in H-bond number*', '*Change in Van der Waals interactions*', '*Change in the number of salt bridges*' were considered. The analysis revealed that all the mutant ROBO1.IG1-SLIT2.D2 docked complexes have increased free energy change compared to the respective wild type. One variant in ROBO4.IG1-2 (G102C) docked to SLIT2.D2 (WT), and 1 variant in SLIT2.D2 docked to ROBO4.IG1-2 (WT) caused a decrease in free energy upon docking as compared to the wild type-wild type docked complexes. The results for both lung cancer and non-lung cancer datasets are summarised in the following table (Table 4).

**Table 4:** Weighted score matrix for the analyses of docked complexes with the final gradation of the variants. An assessment of the protein–protein interaction influenced by somatic variants.

Protein–Protein Interactions	Changes in free energy (ΔG)	Changes in Binding affinity (ΔΔG_Bind_)	Gain in Solvation energy (Δ^i^G)	H-bonds (N)	Van der Waals interactions (N)	Salt-bridges (N)	Super-imposition (RMSD)	Total Score (TS)	Predictive outcome
**(A) Domain-domain docking interactions**									
(I) ROBO1.IG1 + SLIT2.D2									
p.Y323H + p.ROBO1.IG1(WT)	Increase:2	Decrease:2	Decrease:2	Decrease:2	Increase:1	Decrease:2	2	13	More disrupting (TS > Mean TS)
p.G82V + p.SLIT2.D2(WT)	Increase:2	Decrease:2	Decrease:2	Decrease:2	Increase:1	Decrease:2	1	12	More disrupting (TS > Mean TS)
p.C436F + p.ROBO1.IG1(WT)	Increase:2	Decrease:2	Decrease:2	Increase:1	Increase:1	Decrease:2	2	12	More disrupting (TS > Mean TS)
p.R388W + p.ROBO1.IG1(WT)	Increase:2	Decrease:2	Decrease:2	Increase:1	Increase:1	Decrease:2	2	12	More disrupting (TS > Mean TS)
p.R461C + p.ROBO1.IG1(WT)	Increase:2	Decrease:2	Decrease:2	Increase:1	Increase:1	Decrease:2	2	12	More disrupting (TS > Mean TS)
p.L350V + p.ROBO1.IG1(WT)	Increase:2	Decrease:2	Decrease:2	Increase:1	Increase:1	Decrease:2	2	12	More disrupting (TS > Mean TS)
p.L331P + p.ROBO1.IG1(WT)	Increase:2	Decrease:2	Decrease:2	Decrease:2	Increase:1	Decrease:2	1	12	More disrupting (TS > Mean TS)
p.L398F + p.ROBO1.IG1(WT)	Increase:2	Decrease:2	Decrease:2	Decrease:2	Increase:1	Increase:1	2	12	More disrupting (TS > Mean TS)
p.G154E + p.SLIT2.D2(WT)	Increase:2	Decrease:2	Decrease:2	Decrease:2	Increase:1	Increase:1	1	11	More disrupting (TS > Mean TS)
p.R306L + p.ROBO1.IG1(WT)	Increase:2	Decrease:2	Decrease:2	Increase:1	Increase:1	Decrease:2	1	11	More disrupting (TS > Mean TS)
p.R456H + p.ROBO1.IG1(WT)	Increase:2	Decrease:2	Decrease:2	Increase:1	Increase:1	Increase:1	2	11	More disrupting (TS > Mean TS)
p.N430K + p.ROBO1.IG1(WT)	Increase:2	Decrease:2	Decrease:2	Increase:1	Increase:1	Decrease:2	1	11	More disrupting (TS > Mean TS)
p.R420W + p.ROBO1.IG1(WT)	Increase:2	Decrease:2	Decrease:2	Increase:1	Increase:1	Decrease:2	1	11	More disrupting (TS > Mean TS)
p.P431L + p.ROBO1.IG1(WT)	Increase:2	Decrease:2	Decrease:2	Increase:1	Increase:1	Increase:1	2	11	More disrupting (TS > Mean TS)
p.L371P + p.ROBO1.IG1(WT)	Increase:2	Decrease:2	Decrease:2	Decrease:2	Increase:1	Neutral:0	2	11	More disrupting (TS > Mean TS)
p.A340T + p.ROBO1.IG1(WT)	Increase:2	Decrease:2	Decrease:2	Increase:1	Increase:1	Decrease:2	1	11	More disrupting (TS > Mean TS)
** Mean = 10.88**									
p.R119Q + p.SLIT2.D2(WT)	Increase:2	Decrease:2	Decrease:2	Neutral:0	Increase:1	Increase:1	2	10	Less disrupting (TS < Mean TS)
p.I78S + p.SLIT2.D2(WT)	Increase:2	Decrease:2	Decrease:2	Increase:1	Increase:1	Neutral:0	2	10	Less disrupting (TS < Mean TS)
p.P123L + p.SLIT2.D2(WT)	Increase:2	Decrease:2	Decrease:2	Increase:1	Increase:1	Increase:1	1	10	Less disrupting (TS < Mean TS)
p.R131C + p.SLIT2.D2(WT)	Increase:2	Decrease:2	Decrease:2	Neutral:0	Increase:1	Decrease:2	1	10	Less disrupting (TS < Mean TS)
p.D435V + p.ROBO1.IG1(WT)	Increase:2	Decrease:2	Decrease:2	Neutral:0	Increase:1	Decrease:2	1	10	Less disrupting (TS < Mean TS)
p.S352L + p.ROBO1.IG1(WT)	Increase:2	Increase:1	Decrease:2	Increase:1	Increase:1	Increase:1	2	10	Less disrupting (TS < Mean TS)
p.R462C + p.ROBO1.IG1(WT)	Increase:2	Decrease:2	Decrease:2	Increase:1	Increase:1	Neutral:0	2	10	Less disrupting (TS < Mean TS)
p.P322L + p.ROBO1.IG1(WT)	Increase:2	Decrease:2	Decrease:2	Neutral:0	Increase:1	Decrease:2	1	10	Less disrupting (TS < Mean TS)
p.K103N + p.SLIT2.D2(WT)	Increase:2	Decrease:2	Decrease:2	Neutral:0	Increase:1	Increase:1	1	9	Less disrupting (TS < Mean TS)
p.G143R + p.SLIT2.D2(WT)	Increase:2	Decrease:2	Decrease:2	Neutral:0	Increase:1	Increase:1	1	9	Less disrupting (TS < Mean TS)
(II) ROBO4.IG1-2 + SLIT2.D2									
p.S352L + p.ROBO4.IG1-2(WT)	Increase:2	Decrease:2	Decrease:2	Decrease:2	Decrease:2	Decrease:2	2	14	More disrupting (TS > Mean TS)
p.R462C + p.ROBO4.IG1-2(WT)	Increase:2	Decrease:2	Decrease:2	Decrease:2	Decrease:2	Decrease:2	2	14	More disrupting (TS > Mean TS)
p.D435V + p.ROBO4.IG1-2(WT)	Increase:2	Decrease:2	Increase:1	Decrease:2	Decrease:2	Decrease:2	2	13	More disrupting (TS > Mean TS)
p.Y323H + p.ROBO4.IG1-2(WT)	Increase:2	Decrease:2	Increase:1	Decrease:2	Decrease:2	Decrease:2	2	13	More disrupting (TS > Mean TS)
p.R461C + p.ROBO4.IG1-2(WT)	Increase:2	Decrease:2	Increase:1	Decrease:2	Decrease:2	Decrease:2	2	13	More disrupting (TS > Mean TS)
p.R420W + p.ROBO4.IG1-2(WT)	Increase:2	Decrease:2	Increase:1	Decrease:2	Decrease:2	Decrease:2	2	13	More disrupting (TS > Mean TS)
p.C436F + p.ROBO4.IG1-2(WT)	Increase:2	Decrease:2	Decrease:2	Increase:1	Increase:1	Decrease:2	2	12	More disrupting (TS > Mean TS)
p.R388W + p.ROBO4.IG1-2(WT)	Increase:2	Decrease:2	Decrease:2	Increase:1	Increase:1	Decrease:2	2	12	More disrupting (TS > Mean TS)
p.L371P + p.ROBO4.IG1-2(WT)	Increase:2	Decrease:2	Decrease:2	Increase:1	Increase:1	Decrease:2	2	12	More disrupting (TS > Mean TS)
p.C207S + p.SLIT2.D2(WT)	Increase:2	Decrease:2	Decrease:2	Decrease:2	Increase:1	Increase:1	2	12	More disrupting (TS > Mean TS)
p.P431L + p.ROBO4.IG1-2(WT)	Increase:2	Decrease:2	Decrease:2	Neutral:0	Increase:1	Decrease:2	2	11	More disrupting (TS > Mean TS)
p.A340T + p.ROBO4.IG1-2(WT)	Increase:2	Decrease:2	Decrease:2	Decrease:2	Decrease:2	Neutral:0	1	11	More disrupting (TS > Mean TS)
** Mean = 10.90**									
p.R306L + p.ROBO4.IG1-2(WT)	Increase:2	Decrease:2	Increase:1	Neutral:0	Increase:1	Decrease:2	2	10	Less disrupting (TS < Mean TS)
p.L350V + p.ROBO4.IG1-2(WT)	Increase:2	Decrease:2	Decrease:2	Increase:1	Decrease:2	Neutral:0	1	10	Less disrupting (TS < Mean TS)
p.L331P + p.ROBO4.IG1-2(WT)	Increase:2	Decrease:2	Decrease:2	Increase:1	Decrease:2	Neutral:0	1	10	Less disrupting (TS < Mean TS)
p.C207F + p.SLIT2.D2(WT)	Increase:2	Decrease:2	Increase:1	Neutral:0	Increase:1	Decrease:2	2	10	Less disrupting (TS < Mean TS)
p.G86E + p.SLIT2.D2(WT)	Increase:2	Decrease:2	Neutral:0	Neutral:0	Increase:1	Decrease:2	2	9	Less disrupting (TS < Mean TS)
p.N430K + p.ROBO4.IG1-2(WT)	Increase:2	Decrease:2	Decrease:2	Neutral:0	Decrease:2	Neutral:0	1	9	Less disrupting (TS < Mean TS)
p.P322L + p.ROBO4.IG1-2(WT)	Increase:2	Decrease:2	Decrease:2	Neutral:0	Decrease:2	Neutral:0	1	9	Less disrupting (TS < Mean TS)
p.R456H + p.ROBO4.IG1-2(WT)	Increase:2	Decrease:2	Increase:1	Neutral:0	Decrease:2	Neutral:0	1	8	Less disrupting (TS < Mean TS)
p.L398F + p.ROBO4.IG1-2(WT)	Decrease:1	Decrease:2	Decrease:2	Neutral:0	Increase:1	Increase:1	1	8	Less disrupting (TS < Mean TS)
p.G102C + p.SLIT2.D2(WT)	Decrease:1	Increase:1	Increase:1	Decrease:2	Increase:1	Neutral:0	1	7	Less disrupting (TS < Mean TS)
(**B**) **Protein–protein docking interactions**									
(**III**) **ROBO1 + SLIT2**									
p.P54R + p.ROBO1(WT)	Increase:2	Decrease:2	Increase:1	Decrease:2	Decrease:2	Increase:1	2	12	More disrupting (TS > Mean TS)
p.P665S + p.ROBO1(WT)	Increase:2	Decrease:2	Decrease:2	Decrease:2	Increase:1	Decrease:2	1	12	More disrupting (TS > Mean TS)
p.G576R + p.ROBO1 (WT)	Increase:2	Decrease:2	Increase:1	Decrease:2	Decrease:2	Decrease:2	1	12	More disrupting (TS > Mean TS)
p.R635S + p.SLIT2 (WT)	Increase:2	Decrease:2	Increase:1	Decrease:2	Increase:1	Decrease:2	1	11	More disrupting (TS > Mean TS)
p.D312V + p.SLIT2 (WT)	Increase:2	Increase:1	Decrease:2	Neutral : 0	Decrease:2	Decrease:2	2	11	More disrupting (TS > Mean TS)
p.P176L + p.SLIT2 (WT)	Increase:2	Decrease:2	Increase:1	Decrease:2	Increase:1	Decrease:2	1	11	More disrupting (TS > Mean TS)
p.C476Y + p.SLIT2 (WT)	Increase:2	Decrease:2	Increase:1	Increase:1	Decrease:2	Decrease:2	1	11	More disrupting (TS > Mean TS)
** Mean = 10.64**									
p.R306I + p.SLIT2 (WT)	Increase:2	Increase:1	Increase:1	Increase:1	Increase:1	Decrease:2	2	10	Less disrupting (TS < Mean TS)
p.S636C + p.ROBO1 (WT)	Increase:2	Increase:1	Increase:1	Decrease:2	Decrease:2	Neutral : 0	2	10	Less disrupting (TS < Mean TS)
p.G337V + p.SLIT2 (WT)	Increase:2	Increase:1	Increase:1	Neutral : 0	Increase:1	Decrease:2	2	9	Less disrupting (TS < Mean TS)
p.C215Y + p.ROBO1 (WT)	Increase:2	Increase:1	Increase:1	Increase:1	Increase:1	Increase:1	1	8	Less disrupting (TS < Mean TS)
(**IV**) **ROBO4 + SLIT2**									
p.W397C + p.SLIT2 (WT)	Increase:2	Decrease:2	Decrease:2	Increase:1	Increase:1	Increase:1	2	11	More disrupting (TS > Mean TS)
p.G425V + p.SLIT2(WT)	Increase: 2	Decrease: 2	Increase: 1	Decrease: 2	Decrease: 2	Neutral : 0	2	11	More disrupting (TS > Mean TS)
p.G876V + p.SLIT2(WT)	Increase: 2	Decrease: 2	Increase: 1	Decrease: 2	Decrease: 2	Neutral : 0	2	11	More disrupting (TS > Mean TS)
p.P665S + p.ROBO4(WT)	Increase:2	Decrease:2	Decrease:2	Increase:1	Increase:1	Increase:1	2	11	More disrupting (TS > Mean TS)
p.G829W + p.SLIT2 (WT)	Increase:2	Increase:1	Increase:1	Decrease:2	Decrease:2	Neutral : 0	2	10	More disrupting (TS > Mean TS)
p.G576R + p.ROBO4 (WT)	Increase:2	Decrease:2	Decrease:2	Neutral : 0	Increase:1	Increase:1	2	10	More disrupting (TS > Mean TS)
** Mean = 9.222**									
p.S636C + p.ROBO4 (WT)	Increase:2	Increase:1	Increase:1	Increase:1	Increase:1	Increase:1	2	9	Less disrupting (TS < Mean TS)
p.P54R + p.ROBO4(WT)	Increase:2	Decrease:2	Neutral : 0	Neutral : 0	Decrease:2	Neutral : 0	0	6	Less disrupting (TS < Mean TS)
p.C215Y + p.ROBO4 (WT)	Increase:2	Increase:1	Increase:1	Neutral : 0	Neutral : 0	Neutral : 0	0	4	Less disrupting (TS < Mean TS)

One variant of SLIT2.D2 (S352L) docked with ROBO1.IG1 (WT) and 1 variant of ROBO4.IG1-2 (G102C) docked with SLIT2.D2 (WT) show an increase in binding affinity of the interacting proteins compared to the respective wild type docked complexes, which depicts the docking stabilising effect of the variants. The remaining 8 ROBO1.IG1 mutants docked with SLIT2.D2 (WT), 17 SLIT2.D2 mutants docked with ROBO1.IG1 (WT), 3 ROBO4.IG1-2 mutants docked with SLIT2.D2 (WT), and 18 SLIT2.D2 mutants docked with ROBO4.IG1-2 (WT) show a decrease in binding affinity compared to the respective wild type-wild type docked complexes, signifying the docking disruption effect of the variants. The results for both lung cancer and non-lung cancer datasets are summarised in the following table (Table 4).

Analysis over ePISA webserver revealed 8 ROBO1.IG1 mutants docked with SLIT2.D2 (WT), 18 SLIT2.D2 mutants docked with ROBO1.IG1 (WT), 1 ROBO4.IG1-2 mutants docked with SLIT2.D2 (WT), and 12 SLIT2.D2 mutants docked with ROBO4.IG1-2 (WT) to show a decrease in solvation energy while 2 ROBO4.IG1-2 mutants docked with SLIT2.D2 (WT), and 6 SLIT2.D2 mutants docked with ROBO4.IG1-2 (WT) show an increase in solvation energy as compared to their respective wild type docked complexes of ROBO1.IG1/SLIT2.D2 and ROBO4.IG1-2/SLIT2.D2. Only 1 ROBO4.IG1-2 mutant docked with SLIT2.D2 (WT) shows no change in solvation energy as compared to their respective wild type docked complexes. The results for both lung cancer and non-lung cancer datasets are summarised in the following table (Table 4). Analysis of the alteration in bond numbers revealed 2 ROBO1.IG1 mutants docked with SLIT2.D2 (WT), 4 SLIT2.D2 mutants docked with ROBO1.IG1 (WT), 1 ROBO4.IG1-2 mutants docked with SLIT2.D2 (WT), and 7 SLIT2.D2 mutants docked with ROBO4.IG1-2 (WT) to show a decrease in H-bond number while 2 ROBO1.IG1 mutants docked with SLIT2.D2 (WT), 12 SLIT2.D2 mutants docked with ROBO1.IG1 (WT), and 5 SLIT2.D2 mutants docked with ROBO4.IG1-2 (WT) to show an increase in H-bond number as compared to their respective wild type docked complexes. Similarly, the change in the number of van der Waals interactions and salt bridges were assessed. The alterations in the number of bonded and non-bonded interactions in mutant to wild type docked complexes, collectively contribute to a more significant perturbation/disruption in the protein–protein interaction compared to their respective wild type docked complexes. The results of the analysis for both lung cancer and non-lung cancer datasets are depicted in (Table 4).

**Figure 2:**
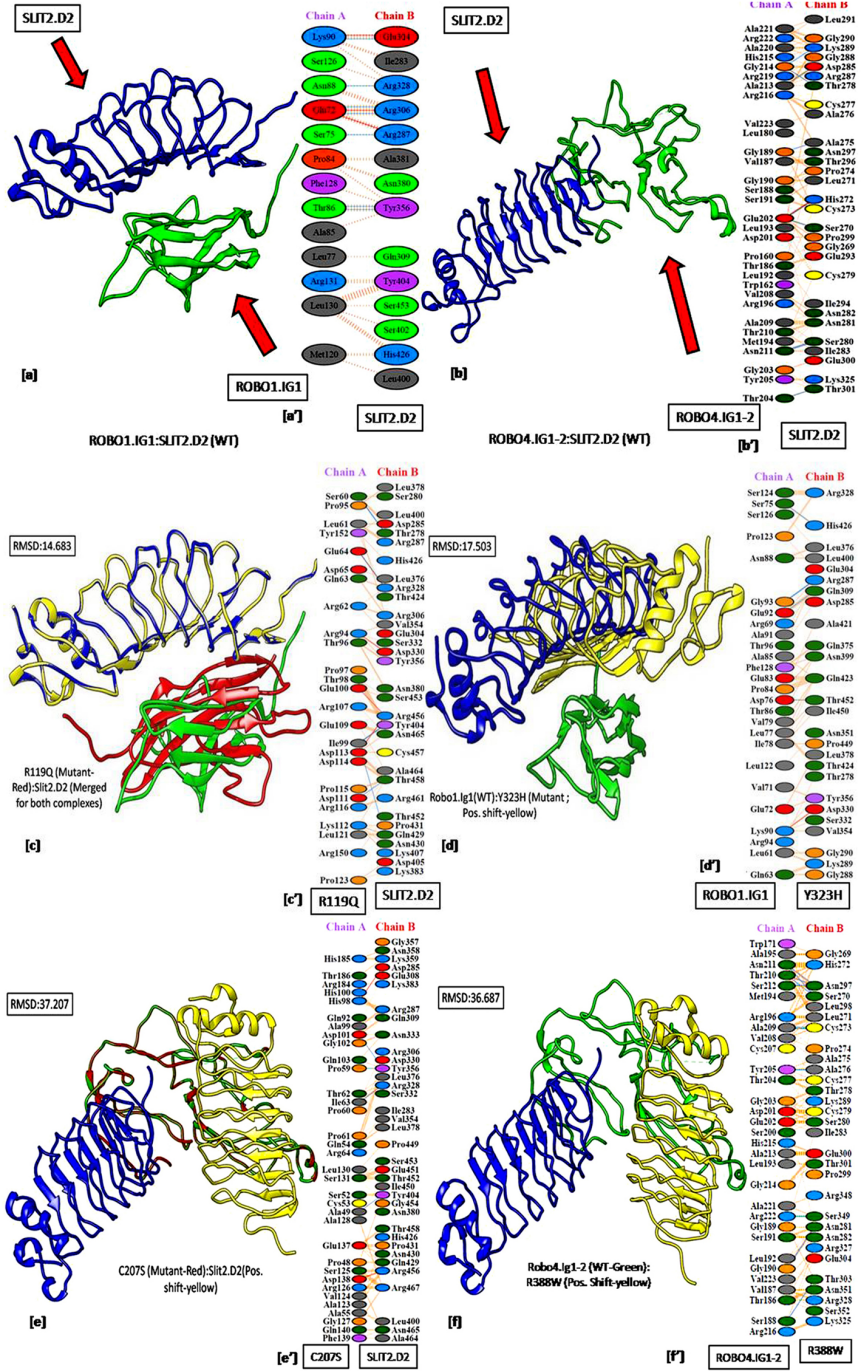
Domain-Domain interaction of wild type-to-wild type and mutant-to-wild type structures. (**a**) ROBO1.IG1(wt):SLIT2.D2(wt) docked complex, (**a′**) ROBO1.IG1(wt): SLIT2.D2(wt) interaction plot; (**b**) ROBO4.IG1-2(wt):SLIT2.D2(wt) docked complex, (**b′**) ROBO4.IG1-2(wt):SLIT2.D2(wt) interaction plot; (**c**) R119Q:SLIT2.D2(wt) docked complex, (**c′**) R119Q: SLIT2.D2(wt) interaction plot; (**d**) ROBO1.IG1(wt):Y323H docked complex, (**d′**) ROBO1.IG1(wt): Y323H interaction plot; (**e**) C207S:SLIT2.D2(wt) docked complex, (**c′**) C207S: SLIT2.D2(wt) interaction plot; (**f**) ROBO4.IG1-2(wt):R388W docked complex, (**b′**) ROBO4.IG1-2(wt):R388W interaction plot; Red depicts ROBO.IG1 and ROBO4.IG1-2 of mutant complex, Green depicts wild type ROBO1.IG1 and ROBO4.IG1-2, Blue depicts wild type SLIT2.D2, and Yellow depicts SLIT2.D2 of the mutant complex.

Finally, pairwise structural alignment of the mutant-to-wild type on wild type-to-wild type docked complexes followed by calculation of RMSD values of the alignment in UCSF-Chimera revealed true nature of the variants on structural distortions and alteration in the global conformation of the mutant complex and PPIs. Higher the RMSD value more significant is the deviation from the wild type conformation. Thus, to grade the variant, a mean score of the RMSD values of the docked complexes for ROBO1.IG1:SLIT2.D2 and ROBO4.IG1-2:SLIT2.D2 complexes were calculated separately. The RMSD value of each alignment was categorised into three groups, i.e. 'Neutral'—RMSD same as the mean value, 'High disruptor'—RMSD higher than the mean value and 'Low disruptors-RMSD lower than the mean value. Each category is depicted pictorially in the following figure (Fig. 2). The residue-specific interactions for each of the domain-domain docked complexes mentioned in Fig. 2 are summarised in Supplementary Information, Table S5.

Similarly, mutant to wild type whole protein–protein interaction analyses were done following the same protocol for the prioritised variants residing outside the interaction domains of the receptors and ligands. Out of 15 variants and 20 docked complexes, all were found to have increased free energy change compared to the respective wild types. A decrease in binding affinity was found for 12 docked complexes of 9 distinct variants, while the remaining showed an increase in binding affinity. Analysis over ePISA webserver revealed 5 docked complexes for 3 distinct variants to have decreased gain in solvation energy upon docking, 1 variant had a neutral effect on solvation energy. In comparison, 14 docked complexes with 11 distinct variants show an increase in solvation energy as compared to their respective wild type docked complexes of ROBO1.IG1:SLIT2.D2 and ROBO4.IG1-2:SLIT2.D2. About 8 variants each in the categories of interactions mentioned above cause a decrease in the number of hydrogen bonds, van der Waals interactions, and salt bridges, respectively that confers more significant perturbation/disruption in protein–protein interaction of the receptor and ligand. Following the same protocol, superimposition analysis was performed in UCSF-Chimera by a pairwise structural alignment that revealed 4 variants of ROBO1 and 3 variants of SLIT2 to have a more significant disruptive effect on ROBO1:SLIT2 docking interaction while 2 variants of ROBO1 and 2 variants of SLIT2 have a lesser disruptive effect on ROBO1:SLIT2 docking interaction.

**Figure 3:**
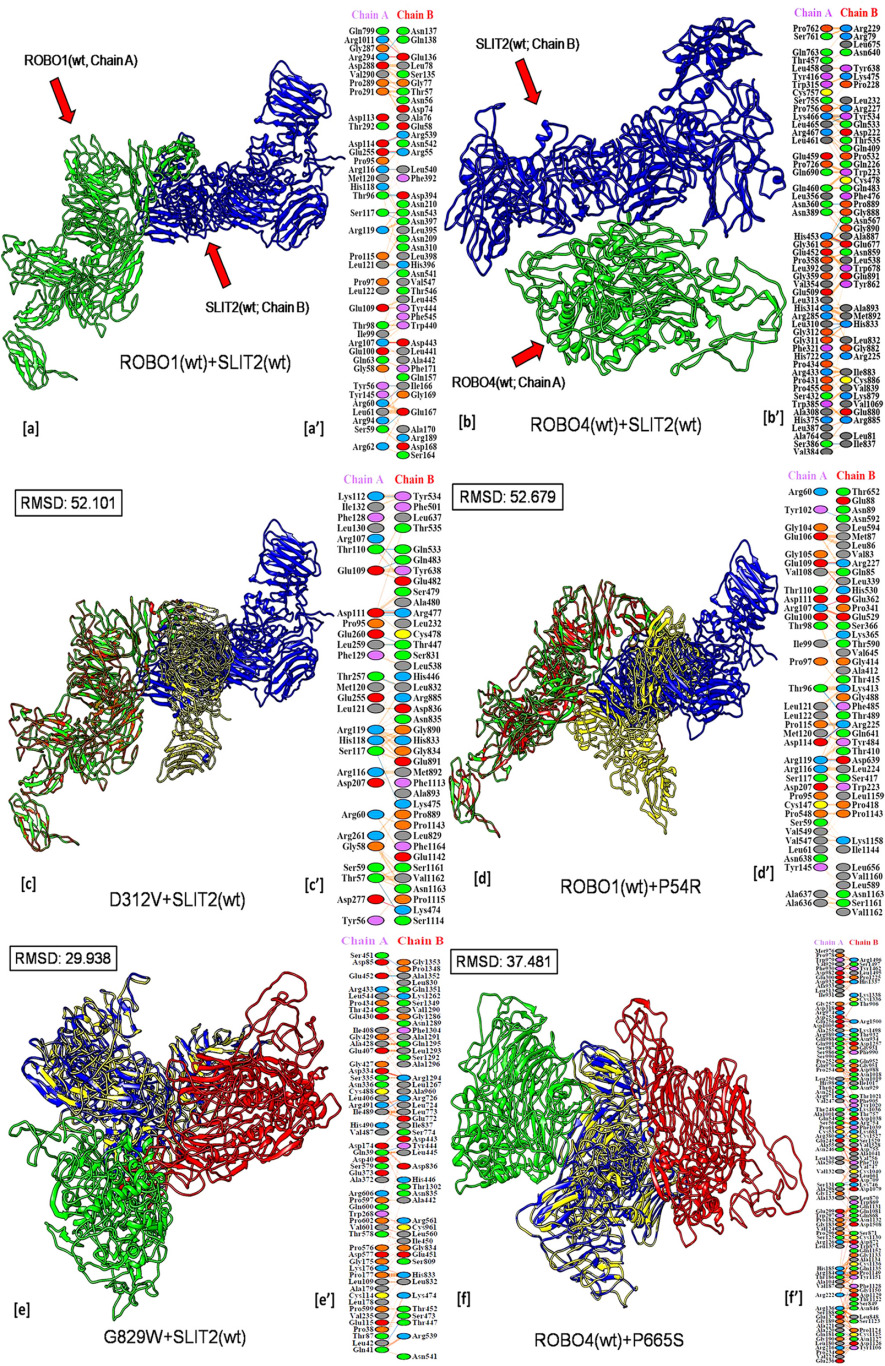
Protein–Protein interaction of wild type-to-wild type and mutant-to-wild type structures. (**a**) ROBO1(wt):SLIT2(wt) docked complex, (**a′**) ROBO1(wt):SLIT2(wt) interaction plot; (**b**) ROBO4(wt):SLIT2(wt) docked complex, (**b′**) ROBO4(wt):SLIT2(wt) interaction plot; (**c**) D312V:SLIT2(wt) docked complex, (**c′**) D312V: SLIT2(wt) interaction plot; (**d**) ROBO1(wt):P54R docked complex, (**d′**) ROBO1(wt): P54R interaction plot; (**e**) G829W:SLIT2(wt) docked complex, (**e′**) G829W: SLIT2(wt) interaction plot; (**f**) ROBO4(wt):P665S docked complex, (**b′**) ROBO4(wt):P665S interaction plot; Red depicts ROBO1 and ROBO4 of mutant complex, Green depicts wild type ROBO1 and ROBO4, Blue depicts wild type SLIT2 and Yellow depicts SLIT2 of mutant complex.

Similarly, 4 variants of ROBO4 and 2 variants of SLIT2 have a more significant disruptive effect on ROBO4:SLIT2 docking interaction whereas 3 variants of SLIT2 have a lesser disruptive effect on ROBO4:SLIT2 docking interaction (Fig. 3). The residue-specific interactions for each of the protein–protein docked complexes mentioned in Fig. 3 are summarised in Supplementary Information, Table S6. The results for both lung cancer and non-lung cancer datasets are summarised (Table 4).

**Figure 4:**
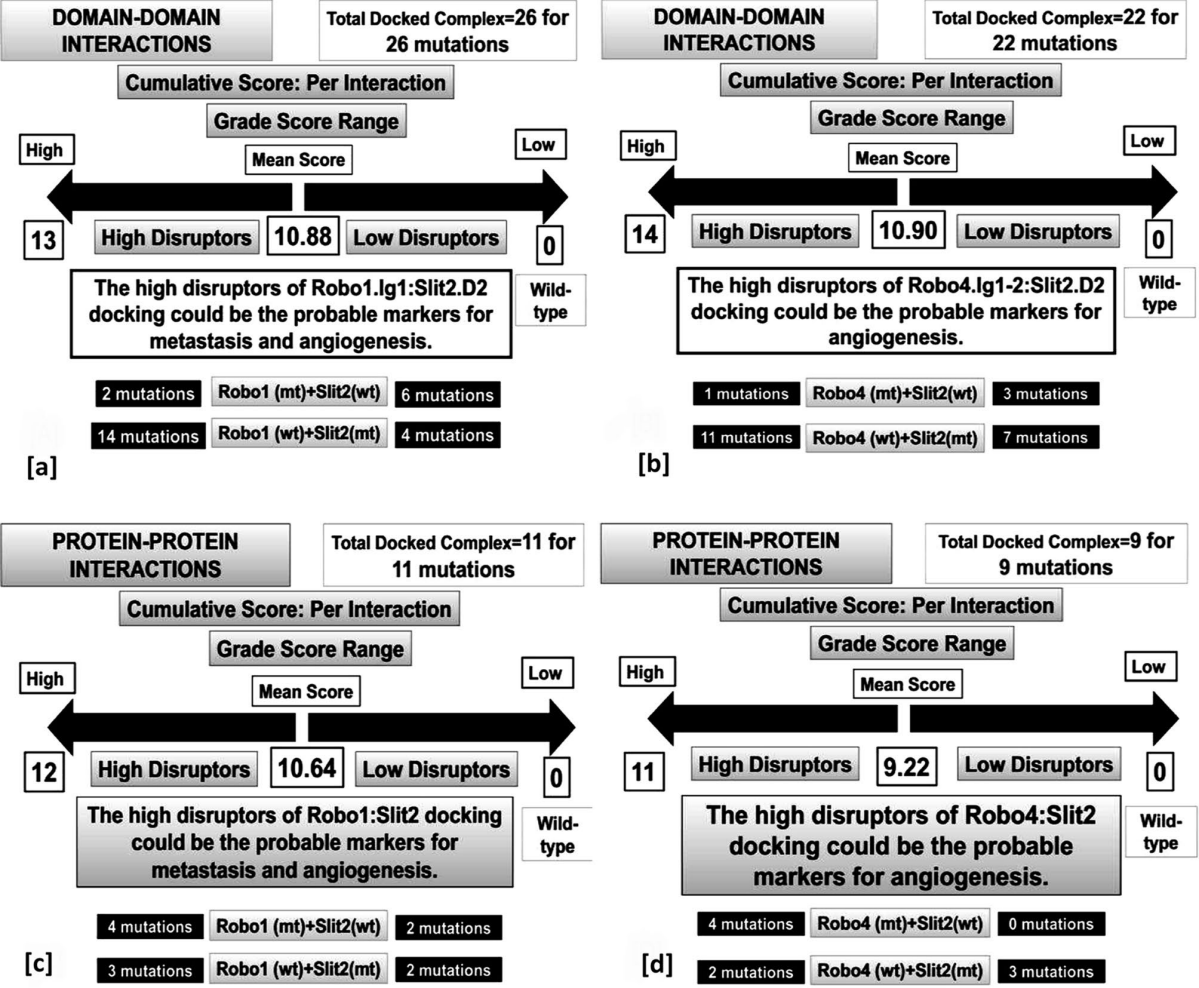
Grading of the variants for their potential impact on the interaction of the receptors to its cognate ligand. The assessment of the deviations between the interaction of wild type-to-wild type and mutant-to-wild type docked complexes. (**a**) ROBO1.IG1:SLIT2.D2 interaction, (**b**) ROBO4.IG1-2:SLIT2.D2 interaction, (**c**) ROBO1:SLIT2 interaction, and (**d**) ROBO4:SLIT2 interaction. The categorisation and distribution of the variants compared to wild types based on their cumulative scores were depicted.

A cumulative weighted score of each post-docking analysis outcomes for each mutant was calculated, followed by the estimation of the mean score of a particular docking category (Table 1). The variant was graded as "more disruptive" when its cumulative score was above the mean and as "less disruptive" when its cumulative score was below mean tending towards '0′ (wild type) (Table 4 and Fig. 4).

## Discussion

Metastasis is the process of tumour cells migrating to secondary sites through blood or lymph vessels. In lung cancer, SLIT-ROBO pathway inhibits tumour cell metastasis through SLIT/ROBO/ Myo9b/RhoA signalling axis^28^. Myosin 9b (Myo9b) is a RhoGAP protein, which modulates protrusion of lamellipodia and retraction of the tail by blocking RhoA activation in migrating immune cells^28^. Myo9b is overexpressed in lung cancer tissue, and it interacts with ROBO1-ICD through its RhoGAP domain, which suppresses the Myo9b RhoGAP activity. Thus, the regular interaction of SLIT2 with ROBO1 suppresses Myo9b leading to inhibition of metastasis^28^.

Tumour progression and survival depends on its ability to induce angiogenesis to get access to oxygen and nutrients for metabolism. ROBO1 and ROBO4 were found to express in the vascular endothelial cells and may have some role in tumour angiogenesis, but very little is known to date. Differential expression of SLIT/ROBO has been found in various phenotypes of endothelial cells where ROBO1 induces long and thin actin fibres, whereas ROBO4 was found to induce short and thick actin fibres along with the membrane ruffles^65^. SLIT2 interacts, with its cognate receptor ROBO1 in the human umbilical vein endothelial cells (HUVECs) and tumour-associated endothelial cells^9^. Studies have shown that the ROBO4 receptor imparts inhibitory signal to endothelial migration, tube formation, and vascular permeability through its interaction with SLIT2, suggesting its negative regulatory role in angiogenesis in HUVEC cells^66–68^ and an animal model for ocular angiogenesis^69^.

Our study is the first report that involves the characterisation of somatic variants of ROBO1.IG1, ROBO4.IG1-2 and SLIT2.D2 domains reported in various cancers including lung cancer, and their role in domain-domain interactions of the receptors (ROBO1.IG1 and ROBO4.IG1-2) and the ligand (SLIT2.D2).

The entire set of missense somatic variants was analysed using the tools mentioned in the materials and method segments, and the results were further categorised using a weighted scoring scheme. The method of variant prioritisation followed in this work conglomerates and assimilated predictions of multiple tools, thus reducing chances of biased misinterpretations. Our results showed that most of the variants were predicted to be "Passenger" in nature by the FATHMM algorithm for cancer driver identification. However, the same variants were predicted to be "Pathogenic" by the FATHMM algorithm for assessing pathogenicity of the coding variants. Thus, it indicates their potential role in the molecular interaction between the ROBO1/4 receptors with its cognate ligand SLIT2.

The Gromos ffG43a2 forcefield has been used previously to study the dynamics of Amyloidβ-Peptides^70^ in which the unstructured regions of the peptides have been found to exhibit high mobility in conformational sampling space which allows them better chances of interactions with cognate partners. In our study, we have also found that the unstructured residues though very minimal in number exhibit higher mobility and thus contribute towards the interaction dynamics of the proteins, which is following the observations of a previously published report^71^. Further, structure-based analysis of the selected variants was done to assess their impact on conformational and structural changes within the protein models. The structural attributes analysed, includes a change in hydrogen bond and steric clash pattern and number between wild type and mutants^72–76^. Thus, it substantiates the local structural distortion upon variants that might be detrimental to the integrity and functionality of the proteins. Change in amino acid localisation within the respective domains correlating to its accessible surface area for solvent action depicts the severity of the variants on structure–function alterations. However, our analysis for variant-induced alteration in ligand binding potential of the domains revealed the impact of such variants on the global conformational distortion and modifications of the sub-regions/pockets within the protein structure for possible therapeutic interventions targeting the ROBO/SLIT pathway in cancer pathogenesis. The variants could create or ablate a ligand/drug-binding pocket, which further signifies the role of the variants as candidate molecular targets in lung cancer biology. Our weighted scoring and subsequent grading of the variants provided insight into their pathogenicity and probable disrupting effect on molecular docking. Post-docking comparative analysis revealed the disruptive effect of the variants on docking dynamics by comparing the change in free energy, binding affinity, gain in solvation energy, bond numbers between mutant and wild type docked complexes. Some variants were found to increase the stability of the docking that indicates their potential role in altering the potential of downstream signal transduction. The pairwise structural alignment of the mutant docked complex over wild type docked complex provided more profound insight into the variant-induced structural distortions that are related to the changes in free energy, binding affinity, solvation energy, and bond numbers. Recurrent variants (p.R119Q and p.R131C of ROBO1 and p.R462C, p.A340T, p.P322L, p.R461C, p.R388W, p.R420W, and p.G576R of SLIT2) found that implies the pathogenic significance of the variants across tumour types. Thus, it signifies the potential role of the variants in critical cancer regulatory pathways, such as metastasis and angiogenesis. Our observation mostly points out towards the inter-group amino acid substitutions with critical localisation within the loops and hinge regions of the protein structures to be responsible for its potential for docking disruption. Another aspect includes the differential surface electrostatic potential induced by the variants, like p.D312V, p.G154E, and p.D435V to be an essential determiner for their impact on molecular interaction between the cognate receptor and ligand. Often the amino acids belonging to the same group, viz. p.G82V, p.I78S, also imparts significant influence on the molecular interaction between the proteins, which could be due to their localisation with the protein structure and not because of the nature of the substitution. These variants could be the potential players for disruption in ROBO/SLIT interaction, leading to increased aggressiveness and metastasis in lung cancer.

Molecular dynamics simulation of the docked complexes for 40 ns using an explicit solvent system — TIP3P water model^77^ in Gromos96 program^78^ followed by coarse-grained simulations was done to evaluate their stability. Once the simulated complexes were generated, the same was analysed for their structural similarities with the original docked complexes, and then the interaction data were also analysed. Majority of structural comparisons yielded averaged RMSD values of < 3.0 Å, which is an acceptable range for the comparisons of protein–protein interaction complexes which are generated through docking and then subjected to simulation^79^. Apart from that, the residue-specific Root Mean Square Fluctuation Values (RMSF) (Supplementary Information, Table S7) were also generated for each of the complexes. All the results indicate that the original docked complexes, as well as the simulated complexes, were conformationally stable and thus, the interaction data and the residues and sites of interactions can be considered to be a good fit for understanding Robo-Slit complexes.

Differences in the mutational landscape exist across various cancer types among between the primary and metastatic cancers. Although there is an extensive commonality in the mutations between the primary and metastatic tumours, some are specific for metastasis. Mutation sites in TP53 vary between primary and metastatic sites in Colorectal cancer, Glioma, NSCLC, and Prostate Adenocarcinoma^80^. The approach of our work paves the way to predict if mutations in ROBO1/4 and SLIT2 have the potential to disrupt their cognate physical interaction that in turn, could regulate the crucial hallmarks of cancer viz. tumour spread and metastasis. Such mutations could be the potential driver or regulator mutations for metastasis and cancer progression. These mutations could serve as molecular targets to design effective novel anticancer therapeutic approaches by precision and personalised medicine. Further, investigations are needed to confirm the computational data in vitro and in vivo, which is beyond the scope of the work presented.

This study deals with the first-ever report on the structural insight of ROBO4.IG1-2 and SLIT2.D2 interaction in a computational model. The structure of ROBO4 is distinctively different from the other ROBO receptors with only two *Immunoglobulin-like* (Ig-like) domains and two fibronectin domains. The details of the molecular interaction of ROBO4.IG1-2 with SLIT2.D2 in our study could provide shreds of evidence for the ROBO4 mediated angiogenesis in lung cancer pathogenesis.

The results of the present study validate a well designed computational pipeline to detect the pathogenic variants conferring disruption to molecular docking of candidate proteins and reduce the exorbitant expenses in the experimental determination of causal variants. With the advent of high-throughput sequencing technologies, new somatic variants have been identified in lung cancer tissue that was previously grouped under other cancers. Therefore, we considered all the variants for in silico assessment of their pathogenicity on docking disruption, which can be further explored for any real-time evidence in metastasis and angiogenesis associated with lung cancer pathophysiology.

## Supplementary Information

Supplementary Information

〹

## Data Availability

All data generated or analysed during this study are included in this published article and its Supplementary files.
