# Supplementary material for: Structural insights and evaluation of the potential impact of missense variants on the interactions of SLIT2 with ROBO1/4 in cancer progression
Source: Sci Rep. 2020 Dec 14;10:21909. doi: 10.1038/s41598-020-78882-2 (PMC7736846; doi:10.1038/s41598-020-78882-2)
Supplement: Supplementary file 1 — Supplementary Information [file 41598_2020_78882_MOESM1_ESM.pdf]

Supplementary Information

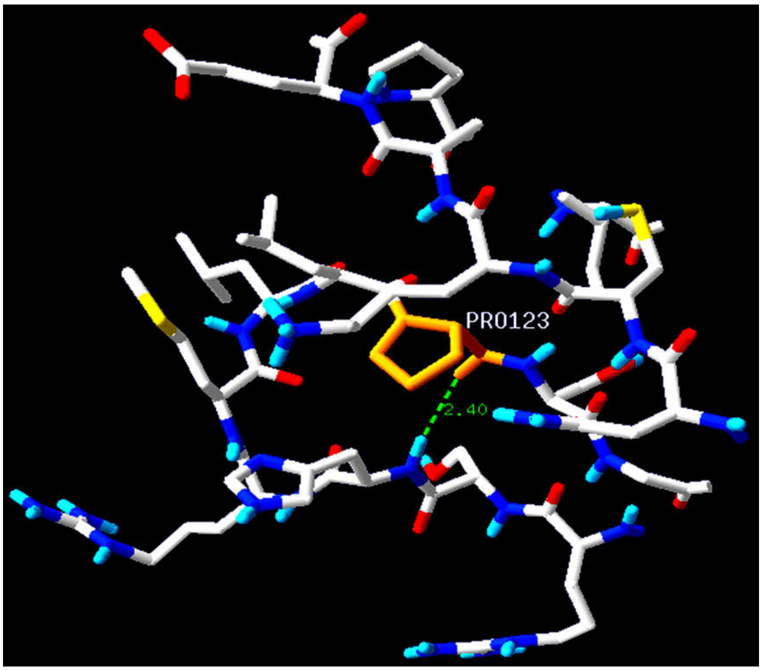
PRO123
2.40

[a]

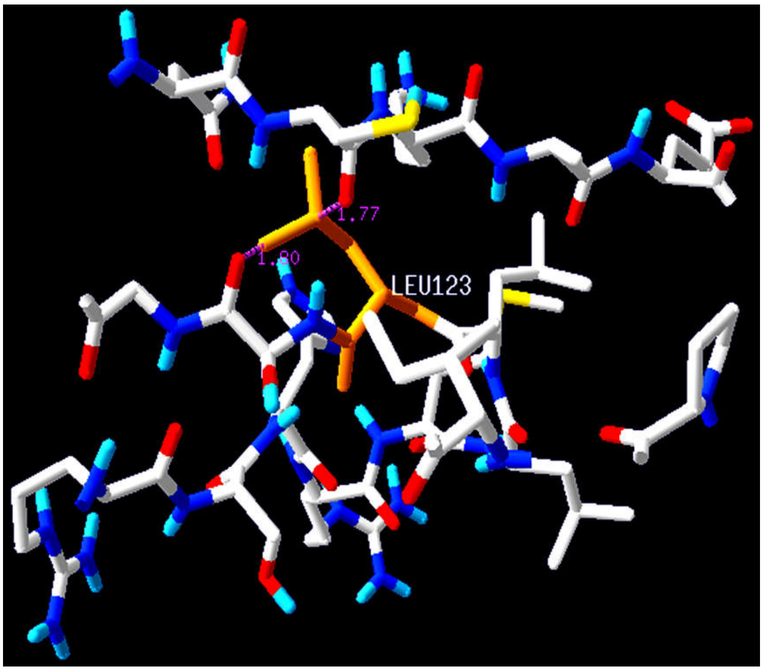
1.77
1.80
LEU123

[a']

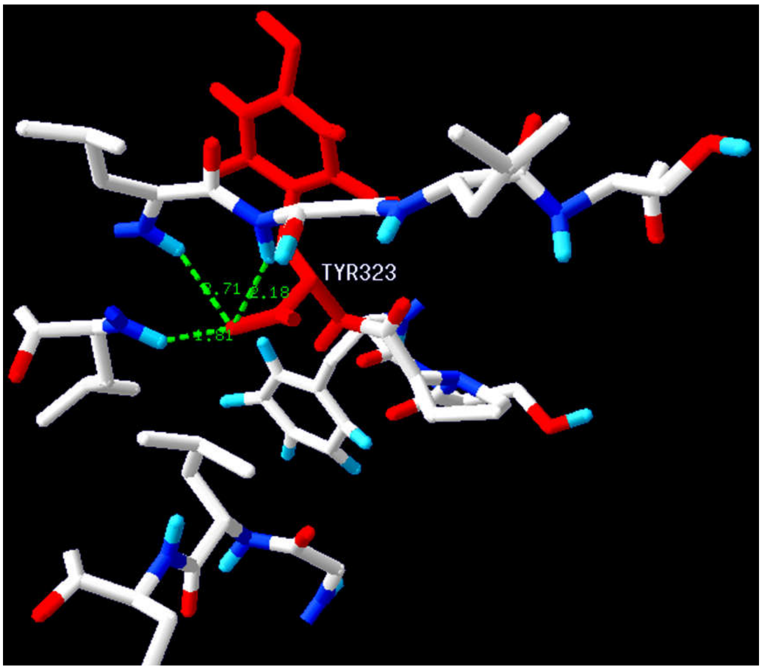
TYR323
2.18

[b]

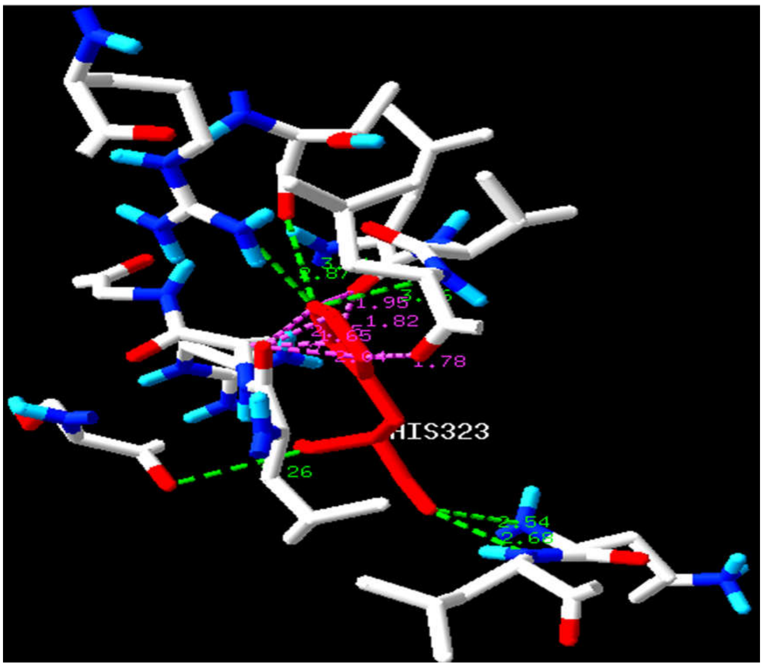
1.95
1.82
1.78
HIS323
2.54

[b']

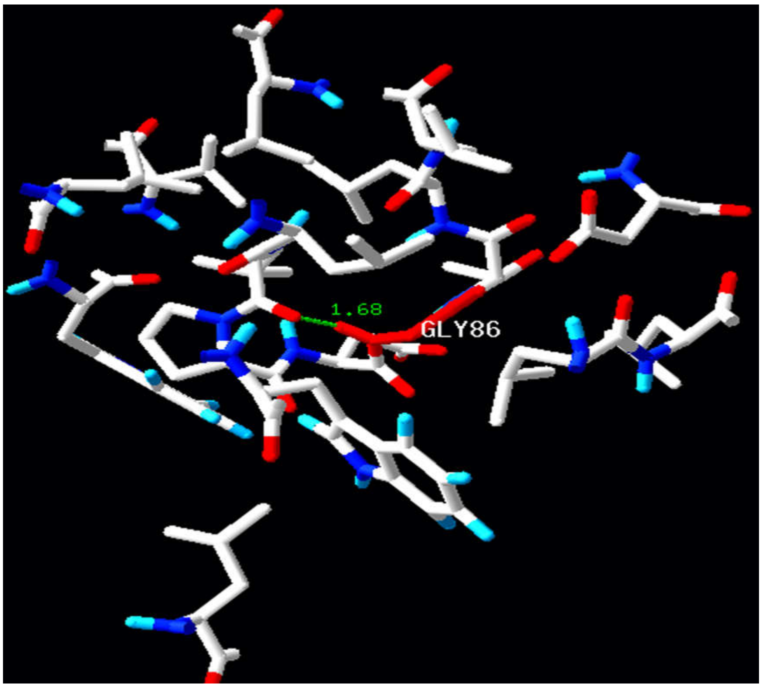
1.68
GLY86

[c]

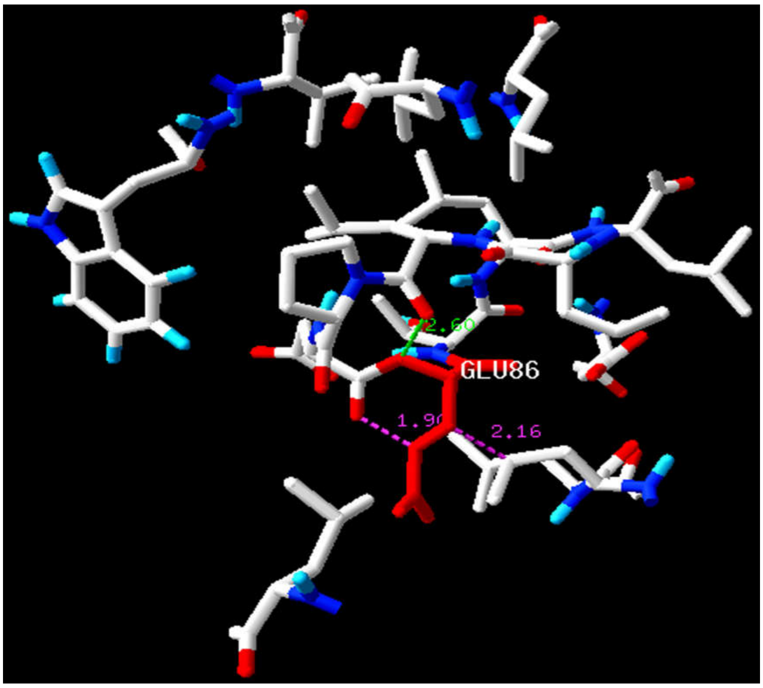
2.60
GLU86
2.16

[c']

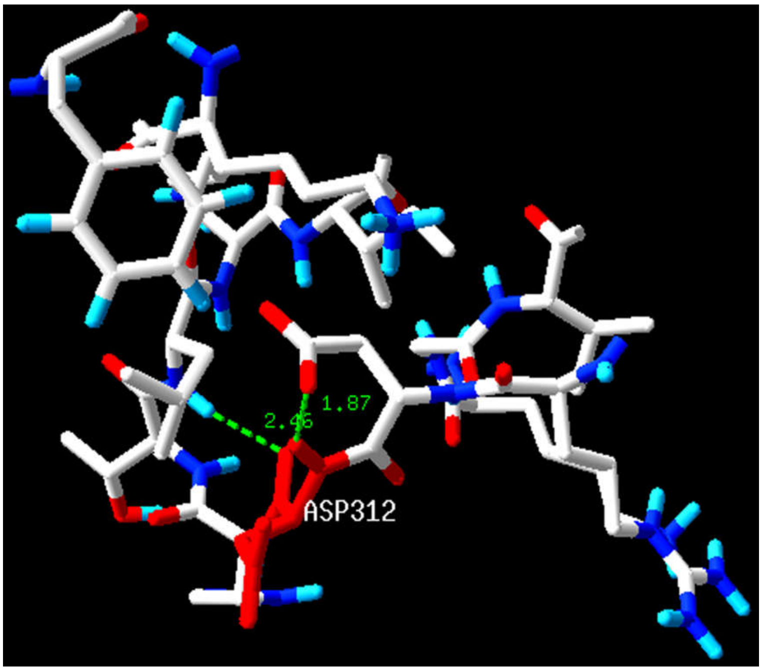

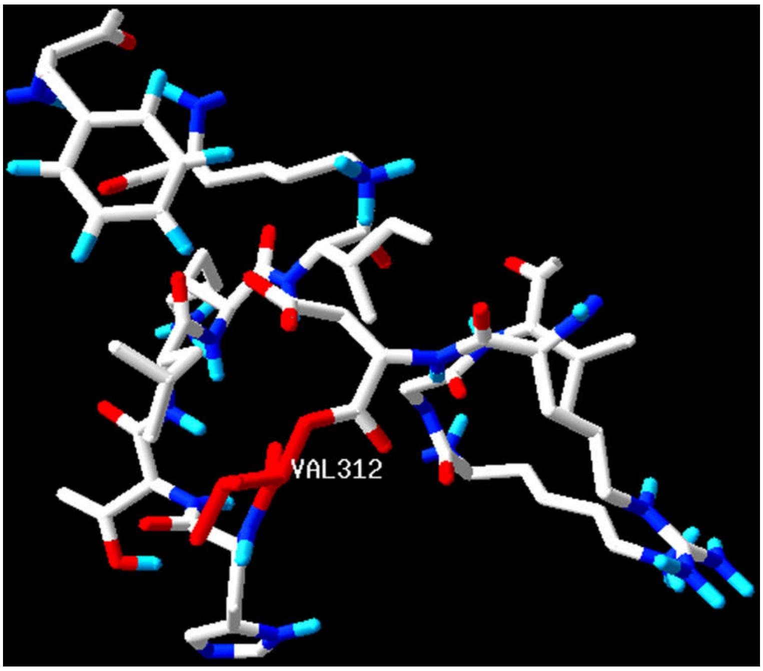

[d] [d’]

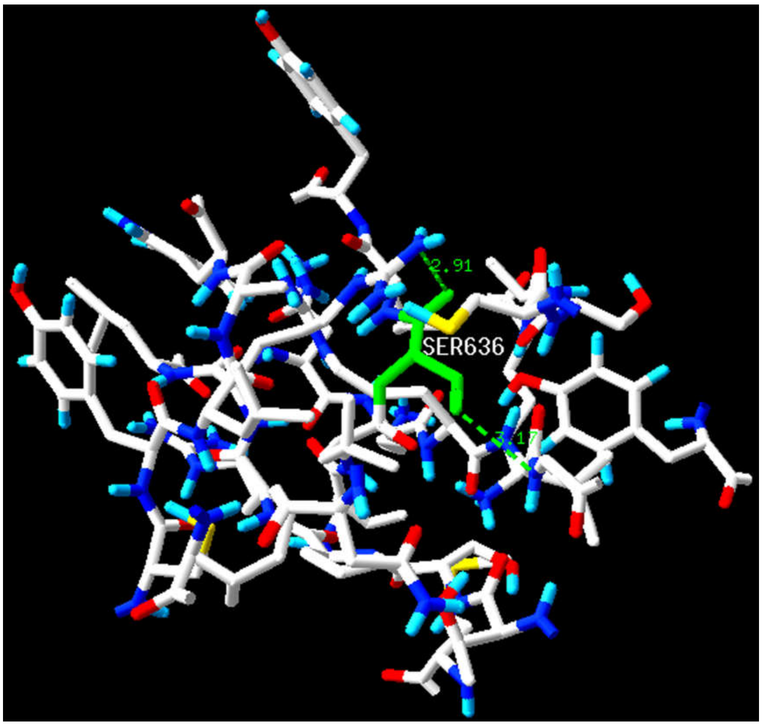

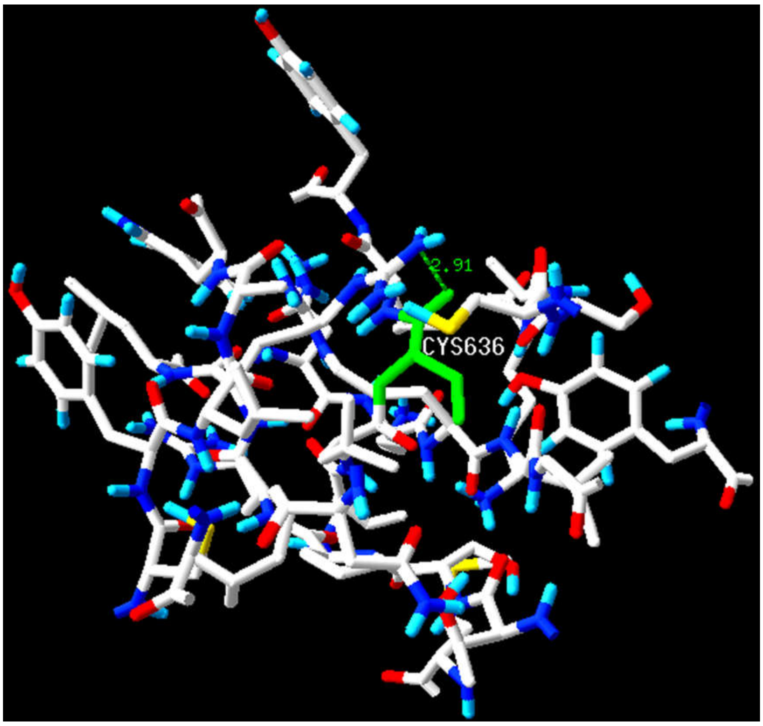

[e] [e’]

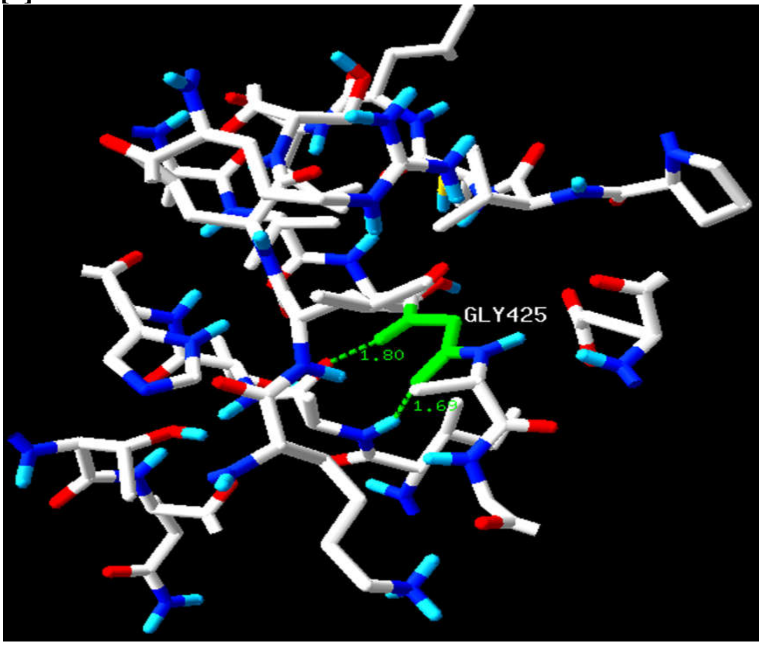

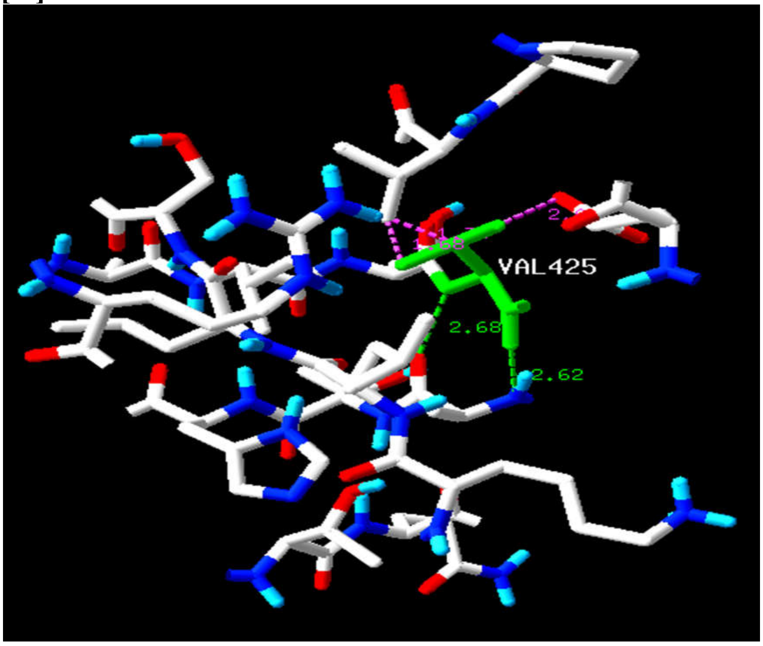

[f] [f’]

Fig. S1

Fig. S1. Effect of variants on the local structural attributes. [a] **ROBO1.IG1** (wild type: **P123,** yellow), **[a']** **ROBO1.IG1** (mutant: **L123,** yellow); **[b]** **SLIT2.D2** (wild type: **Y323,** red), **[b']** **SLIT2.D2** (mutant: **H323,** red); **[c]** **ROBO4.IG1-2** (wild type: **G86,** red), **[c']** **ROBO4.IG1-2** (mutant: **E86,** red), **[d]** **ROBO1-full protein** (wild type: **D312,** red), **[d']** **ROBO1-full protein** (mutant: **V312,** red), **[e]** **SLIT2-full protein** (wild type: **S636,** green), **[e']** **SLIT2-full protein** (mutant: **C636,** green), and **[f]** **ROBO4-full protein** (wild type: **G425,** green), **[f']** **ROBO4-full protein** (mutant: **V425,** green). *Depiction of the nature and number of H-bonds (green dotted lines) and steric clashes (pink dotted lines) changing upon variant. All the images were obtained from DeepView 4.1 (http://www.expasy.org/spdbv/).*

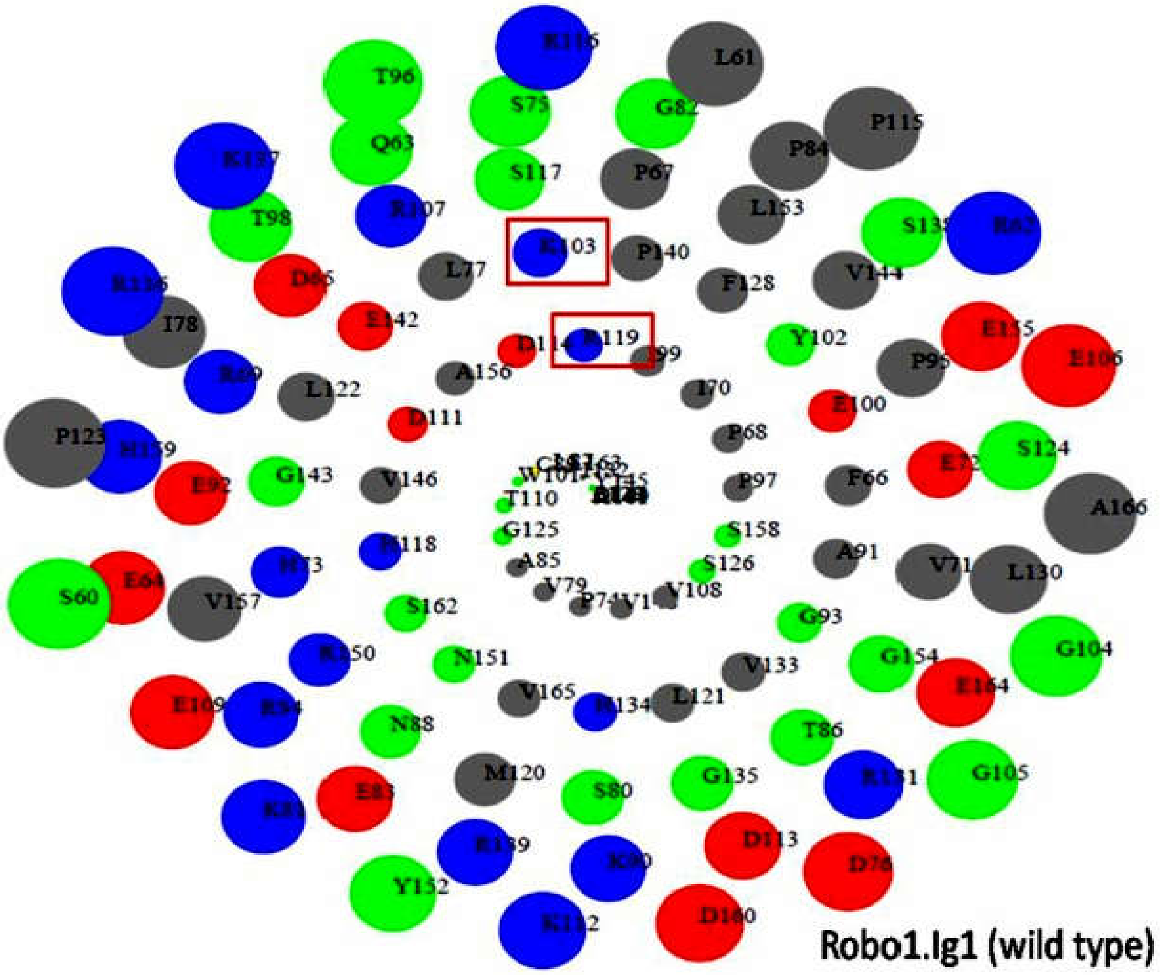

[a].

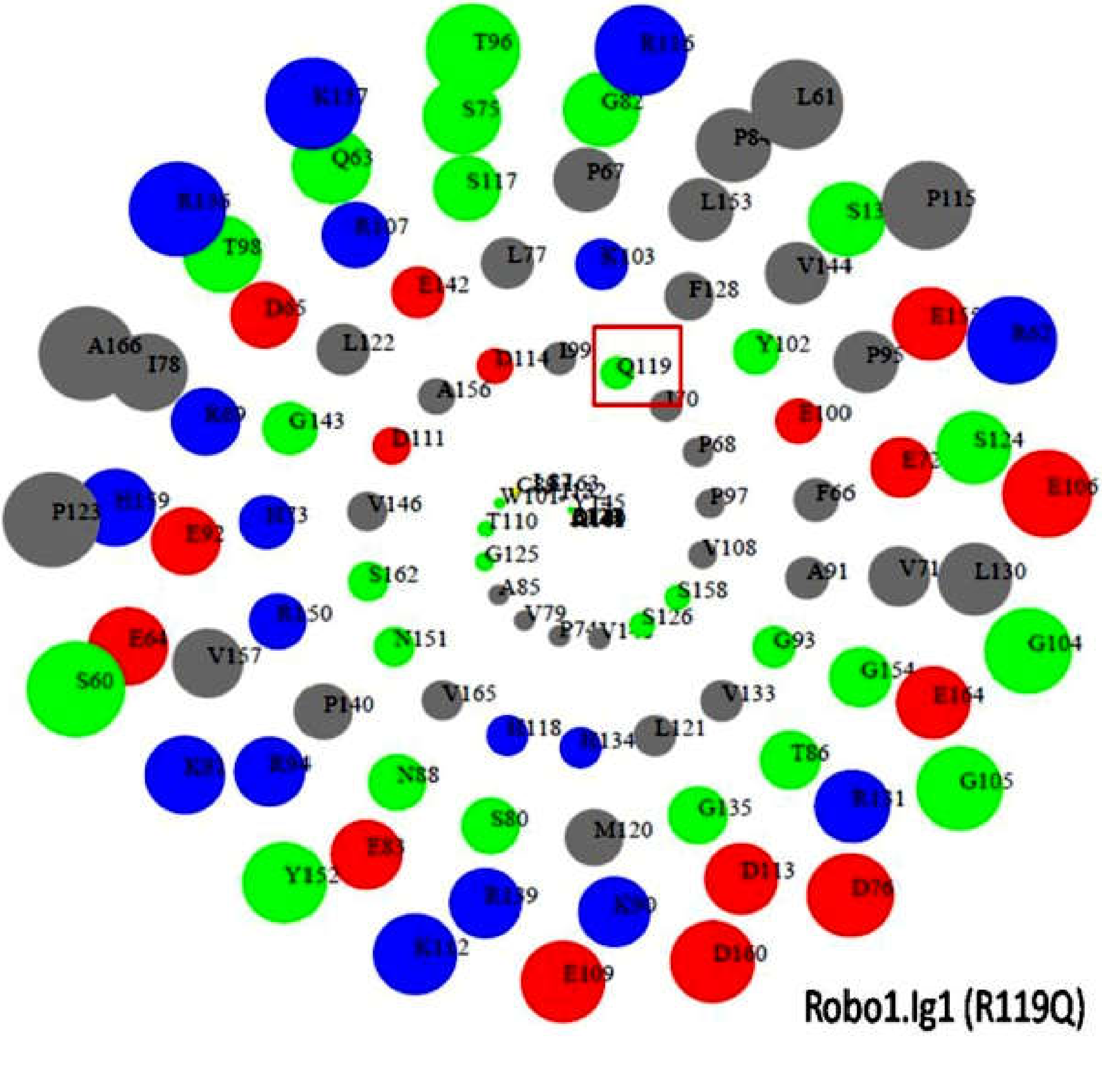

[b].

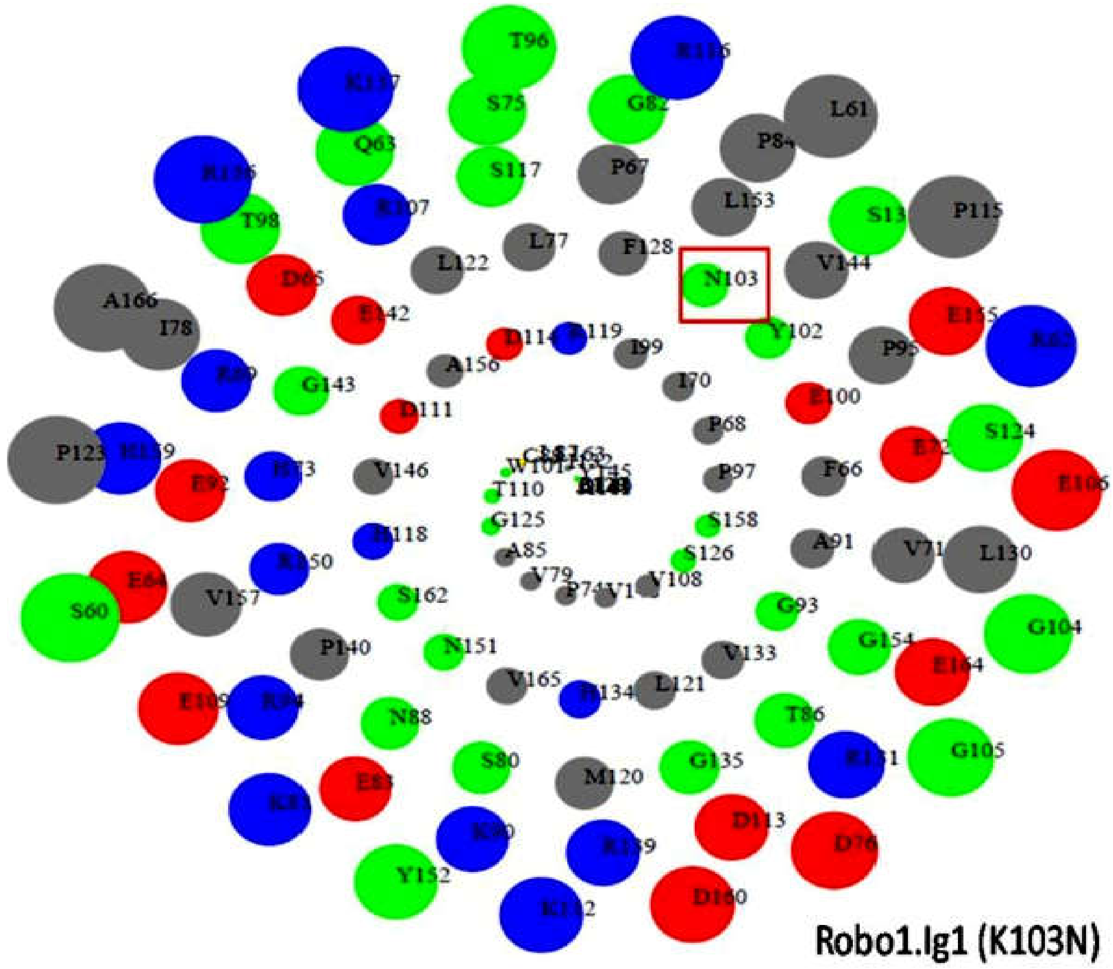
T96
K116
K137
S75
G82
L61
Q63
P84
R136
S117
P67
E107
L153
S13
P115
T98
L77
F128
L122
N103
V144
D65
A166
E142
D114
K119
I99
E155
I78
Y102
P95
A156
G143
I70
E100
D111
P68
S124
P123
E92
E72
V146
T110
P97
F66
E106
G125
S158
H118
A85
S126
A91
V71
L130
E64
V79
V108
S60
V157
S162
G93
G104
G154
P140
N151
V133
E164
V165
L121
E109
N88
T86
S80
G135
G105
M120
D113
D76
Y152
D160
Robo1.Ig1 (K103N)

[c].

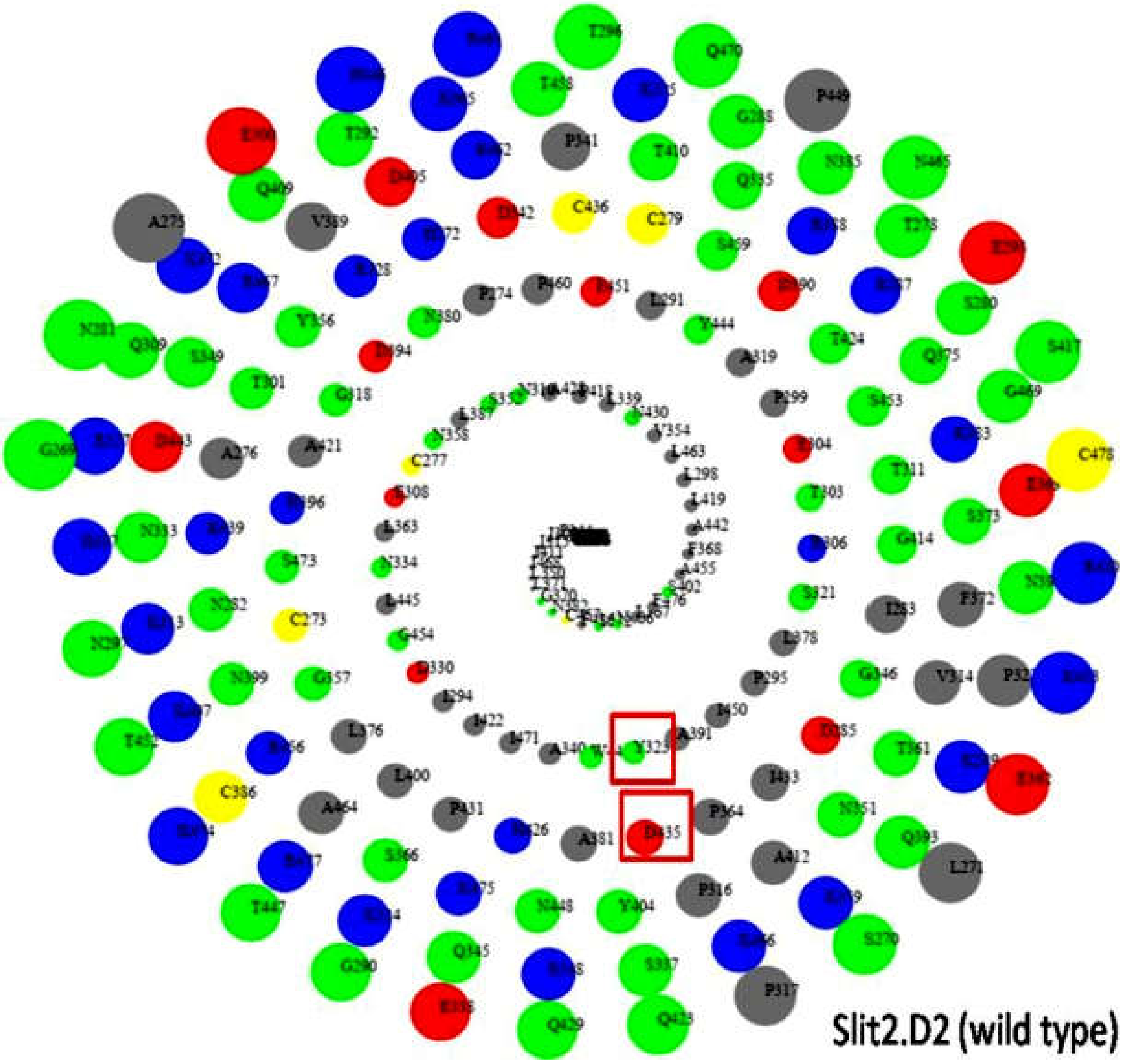
Slit2.D2 (wild type)

[d].

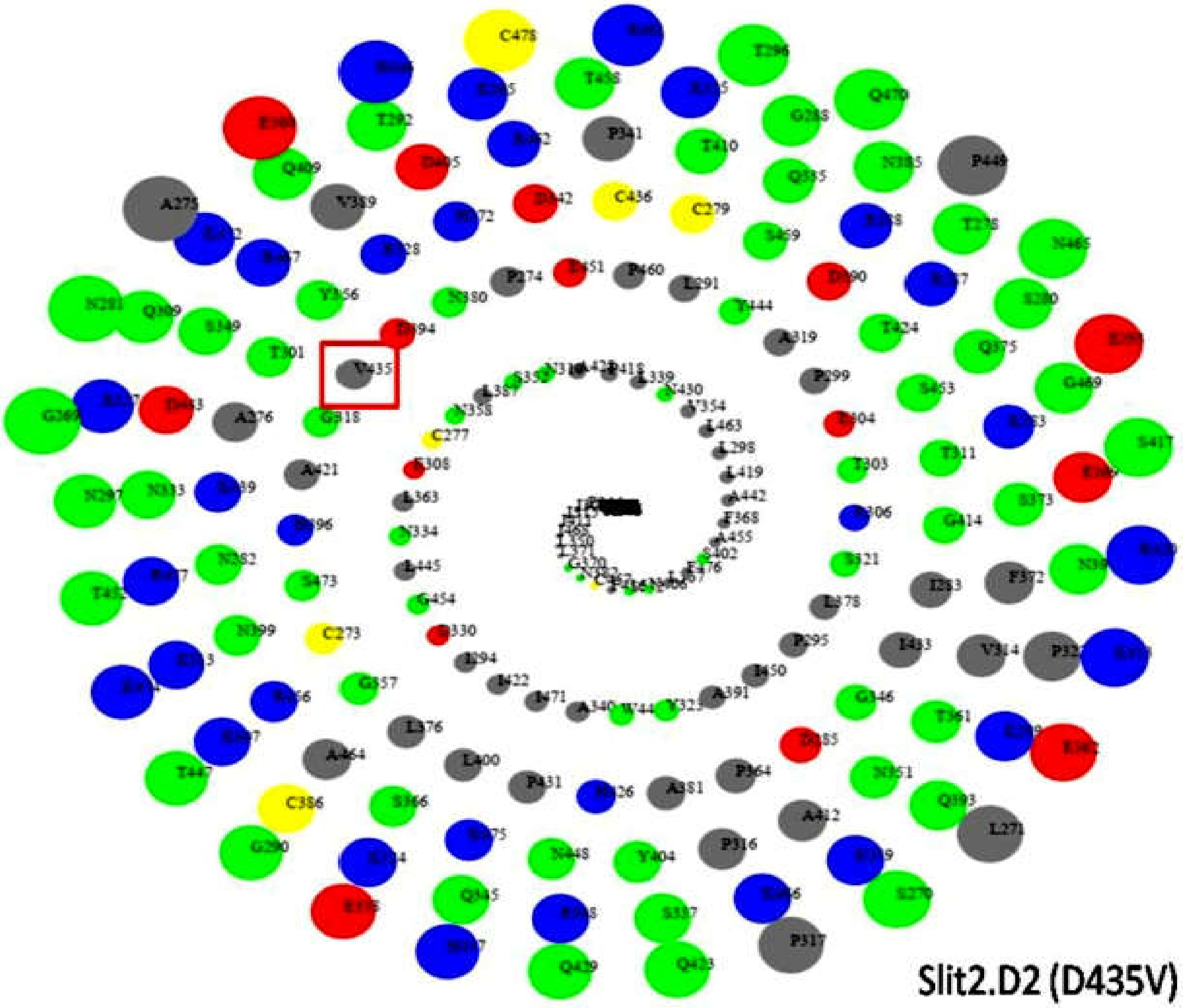

[e].

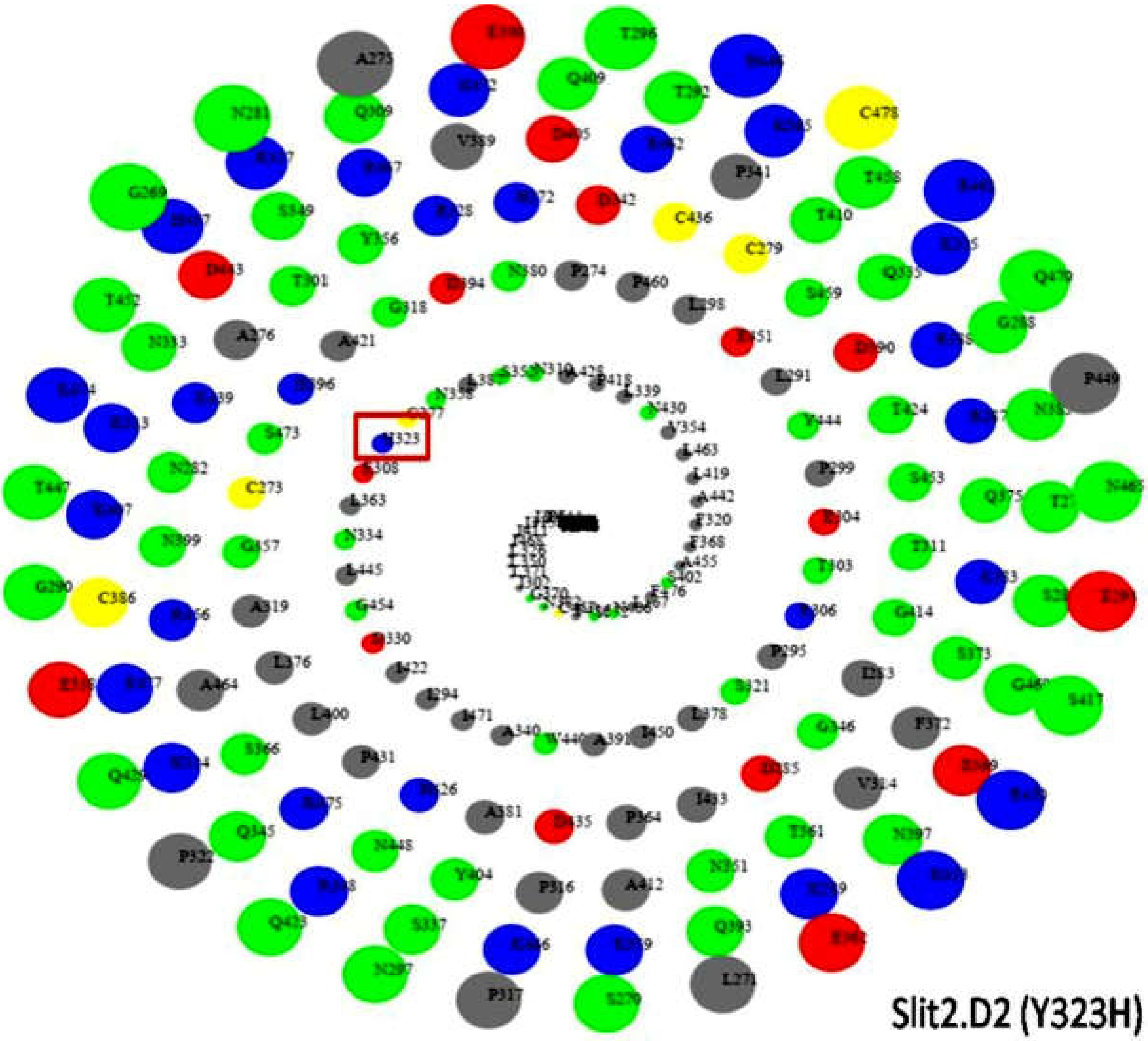

[f].

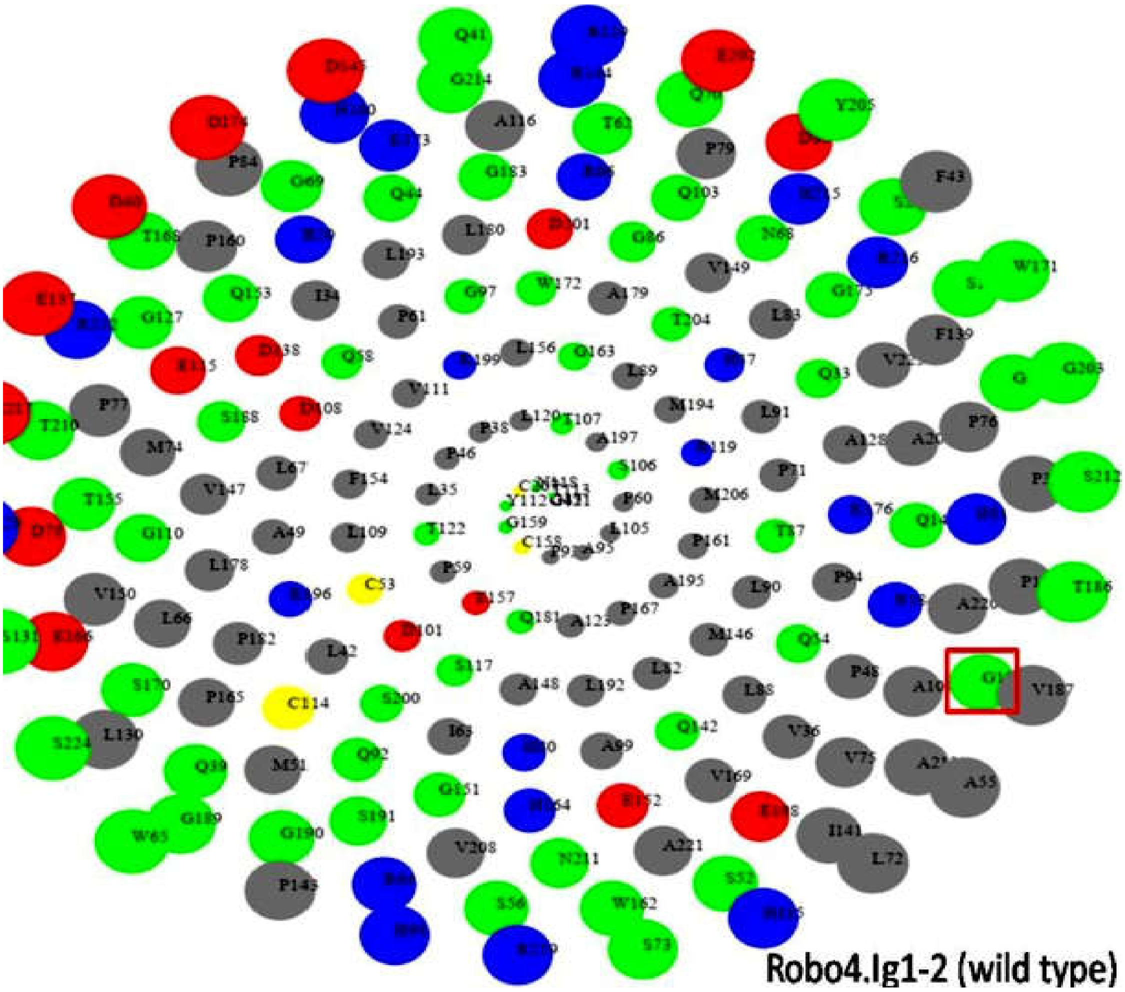

[g].

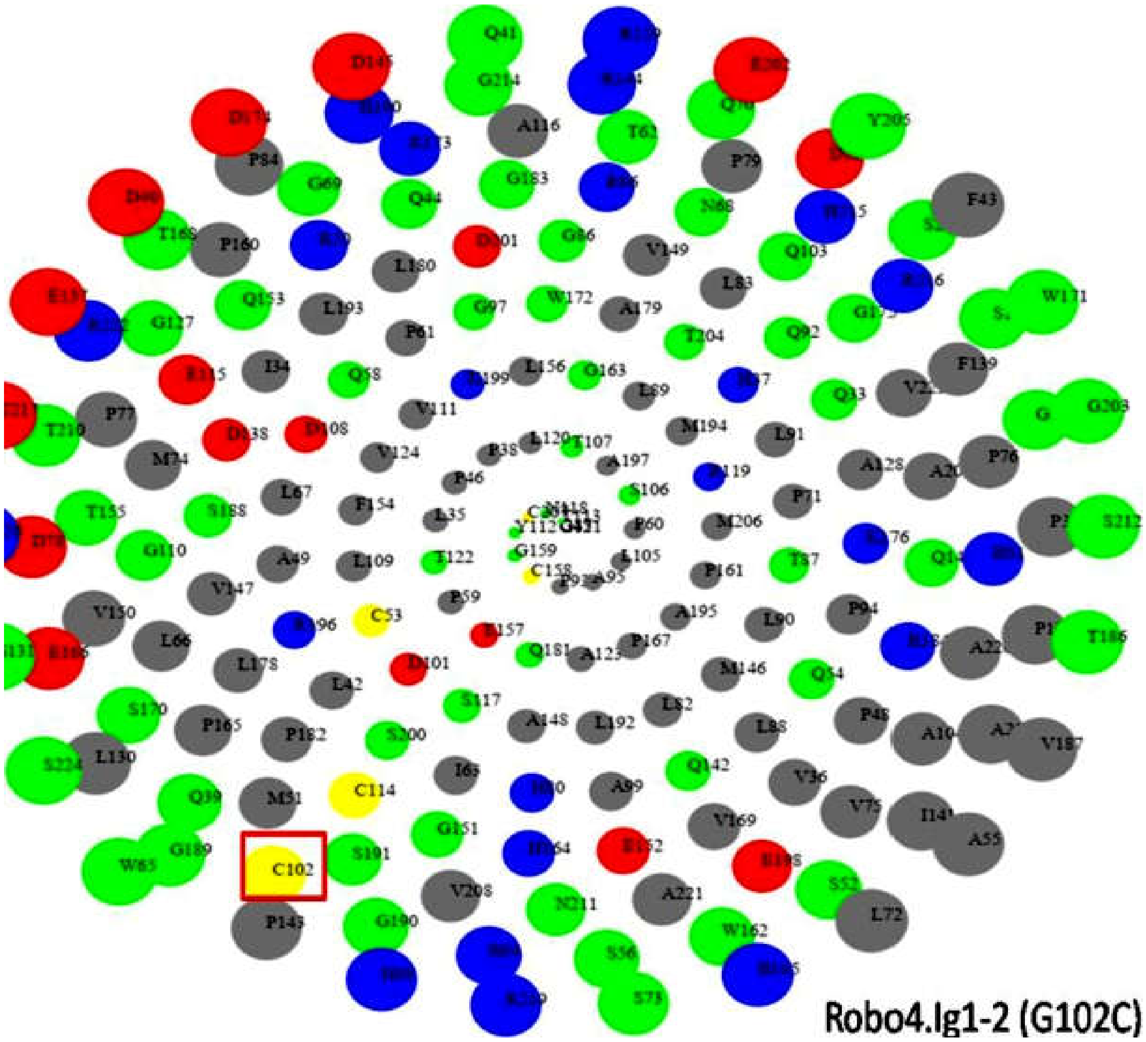

[h].

Fig. S2

Fig. S2. Spiral plot representation of the amino acid position in wild type and mutant structures of interacting domains of the 3 proteins. [a] ROBO1.IG1 (WT), [b] ROBO1.IG1 (p.R119Q), [c] ROBO1.IG1 (K103N), [d] SLIT2.D2 (WT), [e] SLIT2.D2 (D435V), [f] SLIT2.D2 (Y323H), [g] ROBO4.IG1-2 (WT), [h] ROBO4.IG1-2 (G102C). *Change in position is correlated with change in ASA. Blue- positively charged, Red- Negatively charged, Green- Hydrophobic. The red box depicts the mutable residues. WT indicates the wild types of structures. All the images were obtained from ASAView (http://dna00.bio.kyutech.ac.jp/asaview/).*

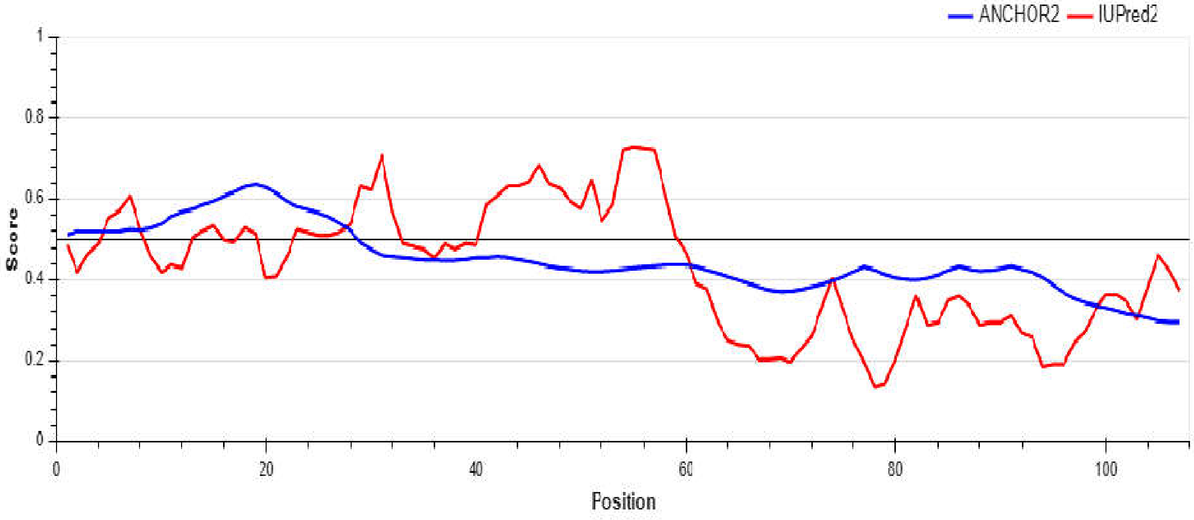
ANCHOR2
IUPred2
Score
Position
0
0.2
0.4
0.6
0.8
1
20
40
60
80
100

[a]

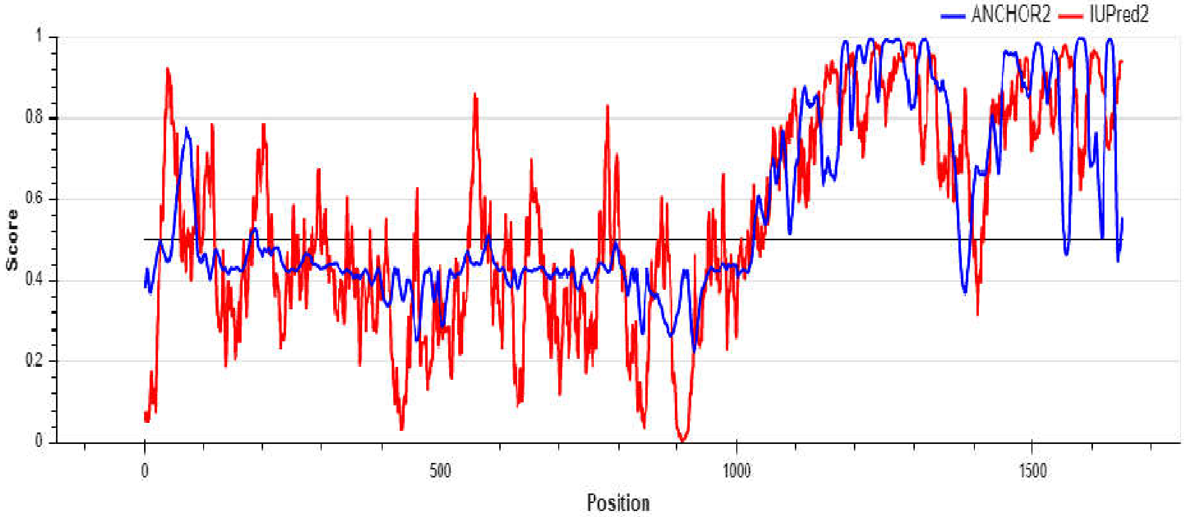
ANCHOR2
IUPred2
Score
Position
0
0.2
0.4
0.6
0.8
1
500
1000
1500

[a’].

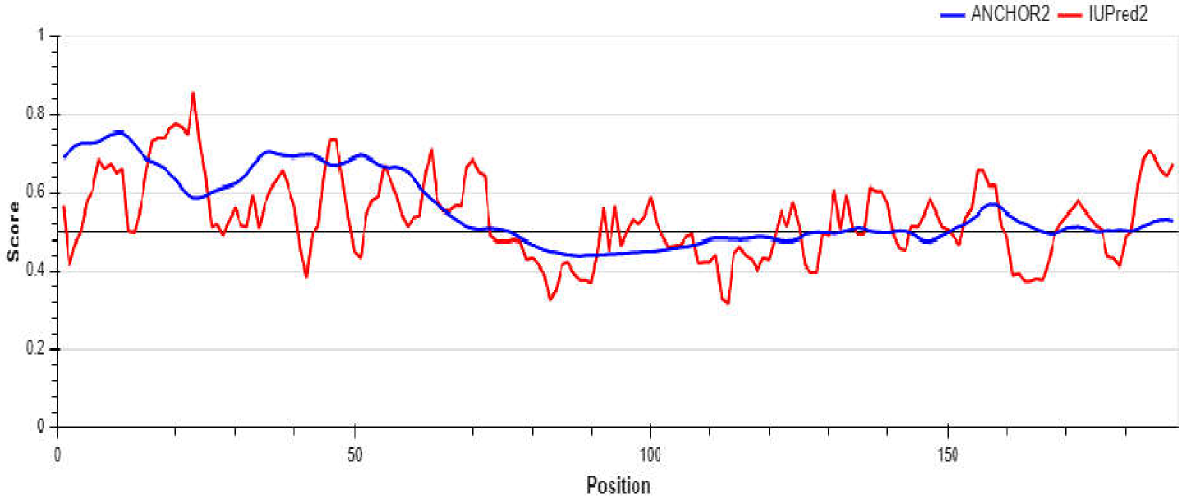
ANCHOR2
IUPred2
Score
Position
0
0.2
0.4
0.6
0.8
1
50
100
150

[b]

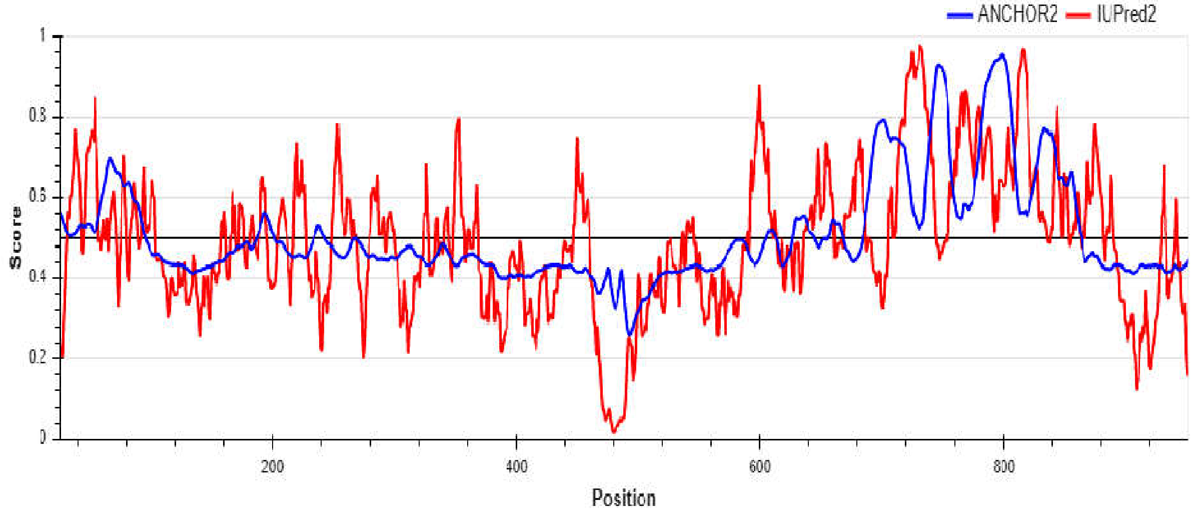
ANCHOR2
IUPred2
Score
Position
0
0.2
0.4
1
200
400
600
800

[b’]

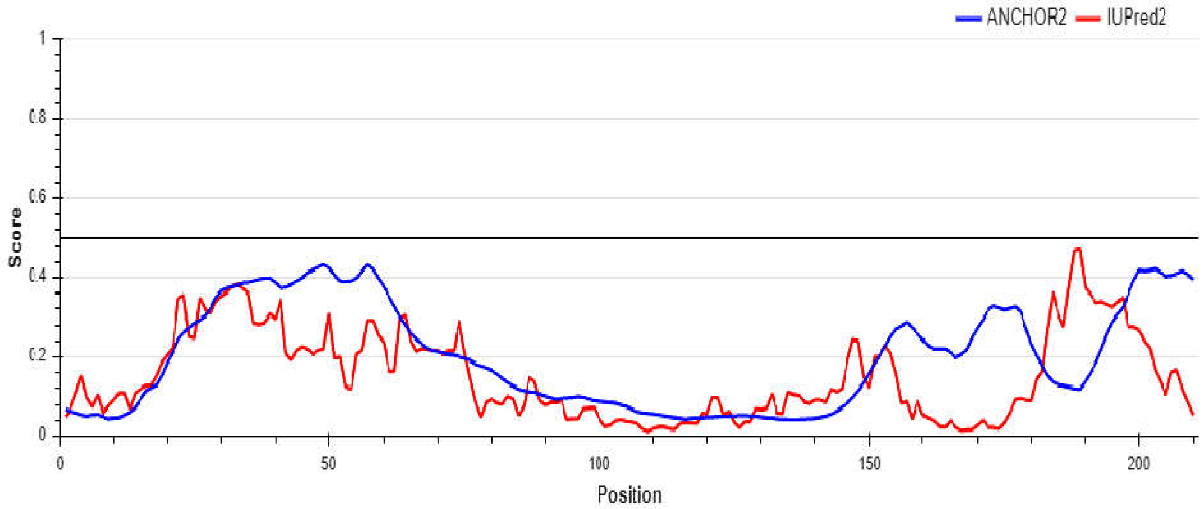

ANCHOR2
IUPred2
Score
1
0.8
0.6
0.4
0.2
0
0
50
100
150
200
Position

[c]

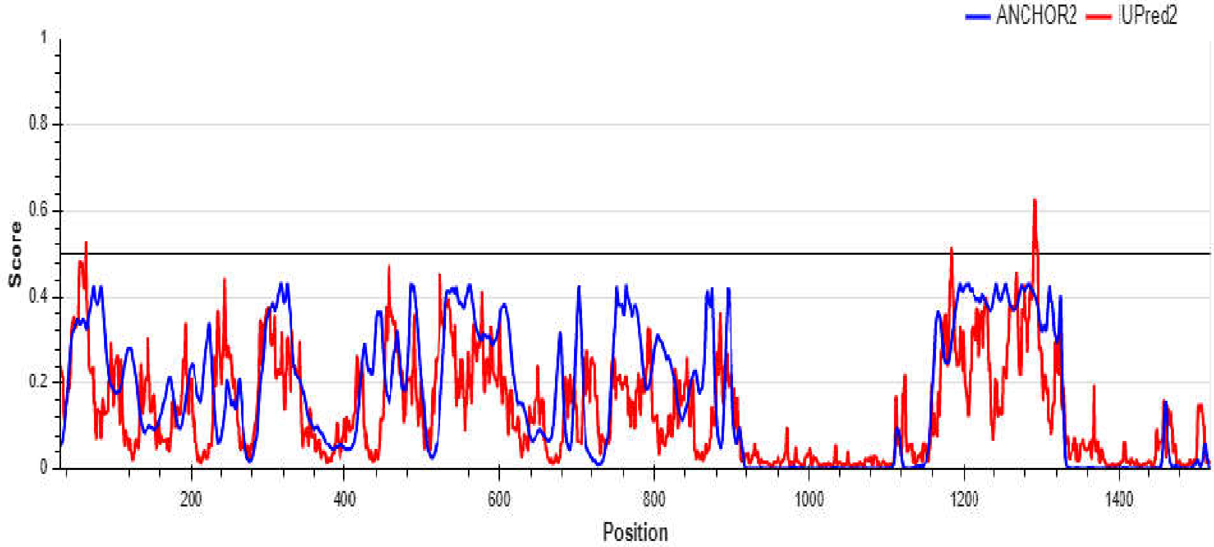

ANCHOR2
IUPred2
Score
1
0.8
0.6
0.4
0.2
0
200
400
600
800
1000
1200
1400
Position

[c’]

Fig. S3

Fig. S3. The sequence-based residue-specific intrinsically unstructured regions of the wild type structures for both partial interacting domains and whole protein 3D model. [a] ROBO1.IG1 (domain), **[a']** ROBO1 (full), **[b]** ROBO4.IG1-2 (domain), **[b']** ROBO4 (full), **[c]** SLIT2.D2 (domain), and **[c']** SLIT2 (full). *The analysis was conducted in the IuPred2A web server, and the images were obtained from the IuPred2A web server (https://iupred2a.elte.hu/). It has been found that many disordered proteins function after binding to a structured partner and undergo a disorder-to-order transition. Thus, to predict disordered binding regions, the ANCHOR web server identifies segments that reside in disordered regions and fail to form favourable intrachain interactions for proper folding and gain stabilising energy by interacting with its cognate protein partner.*

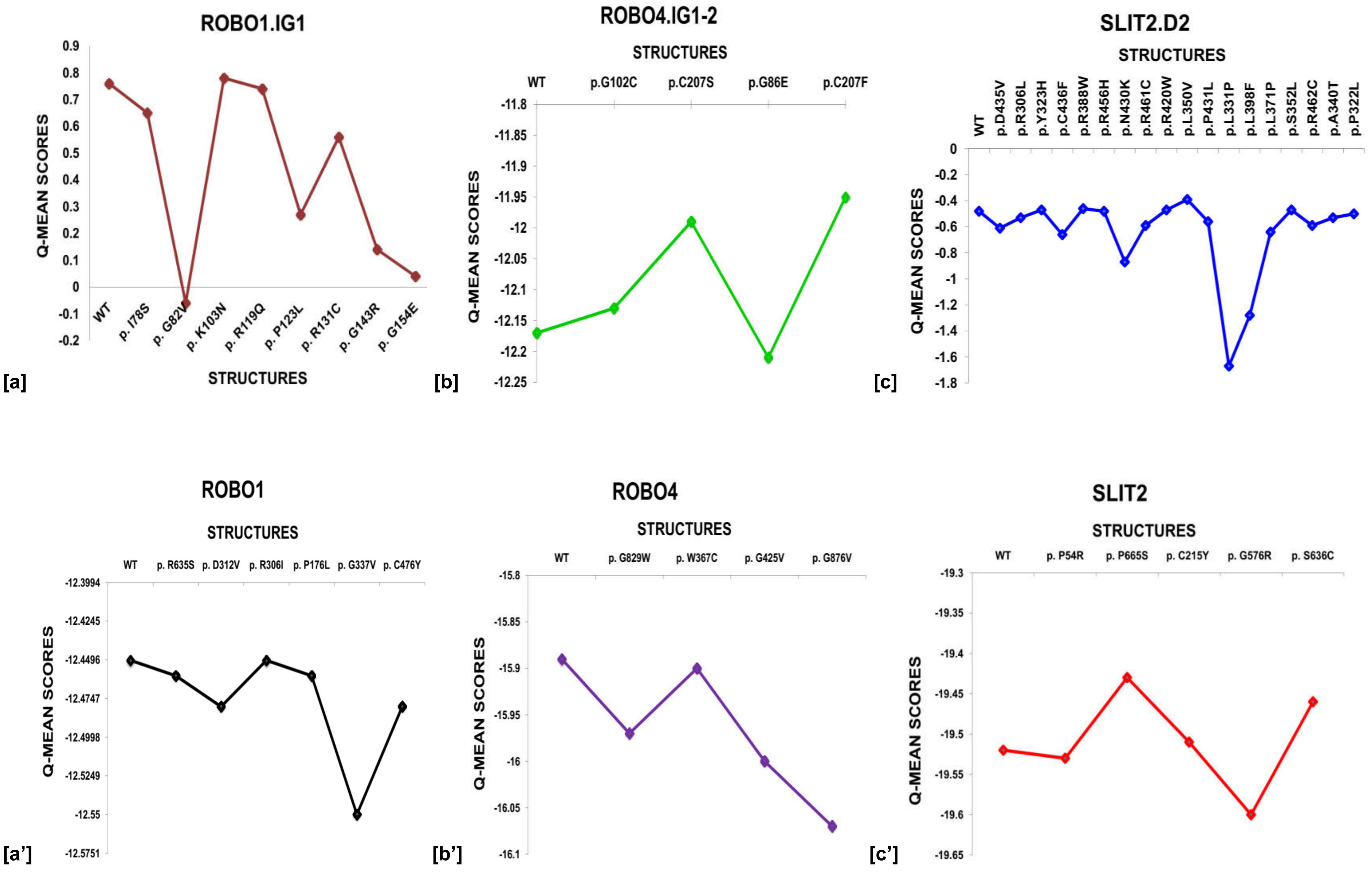
ROBO1.IG1
Q-MEAN SCORES
STRUCTURES
0.9
0.8
0.7
0.6
0.5
0.4
0.3
0.2
0.1
0
-0.1
-0.2
WT
p. I78S
p. G82V
p. K103N
p. R119Q
p. P123L
p. R131C
p. G143R
p. G154E
[a]
ROBO4.IG1-2
STRUCTURES
WT
p.G102C
p.C207S
p.G86E
p.C207F
Q-MEAN SCORES
-11.8
-11.85
-11.9
-11.95
-12
-12.05
-12.1
-12.15
-12.2
-12.25
[b]
SLIT2.D2
STRUCTURES
WT
p.D435V
p.R306L
p.Y323H
p.C436F
p.R388W
p.R456H
p.N430K
p.R461C
p.R420W
p.L350V
p.P431L
p.L331P
p.L398F
p.L371P
p.S352L
p.R462C
p.A340T
p.P322L
Q-MEAN SCORES
0
-0.2
-0.4
-0.6
-0.8
-1
-1.2
-1.4
-1.6
-1.8
[c]
ROBO1
STRUCTURES
WT
p. R635S
p. D312V
p. R306I
p. P176L
p. G337V
p. C476Y
Q-MEAN SCORES
-12.3994
-12.4245
-12.4496
-12.4747
-12.4998
-12.5249
-12.55
-12.5751
[a']
ROBO4
STRUCTURES
WT
p. G829W
p. W367C
p. G425V
p. G876V
Q-MEAN SCORES
-15.8
-15.85
-15.9
-15.95
-16
-16.05
-16.1
[b']
SLIT2
STRUCTURES
WT
p. P54R
p. P665S
p. C215Y
p. G576R
p. S636C
Q-MEAN SCORES
-19.3
-19.35
-19.4
-19.45
-19.5
-19.55
-19.6
-19.65
[c']

Fig. S4

Fig. S4. Graphical representation of Q-Mean scores of the mutants and their respective wild types of both partial interacting domains and whole protein models. [a] ROBO1.IG1, **[b]** ROBO4.IG1-2, **[c]** SLIT2.D2, **[a']** ROBO1, **[b']** ROBO4, and **[c']** SLIT2. *The Q-Mean is a composite scoring function that can derive both global (i.e. for the entire structure) and local (i.e. per residue) absolute quality estimates based on one single model. The graphical image was constructed from the Q-Mean score values of the structures as obtained from https://swissmodel.expasy.org/qmean/.*

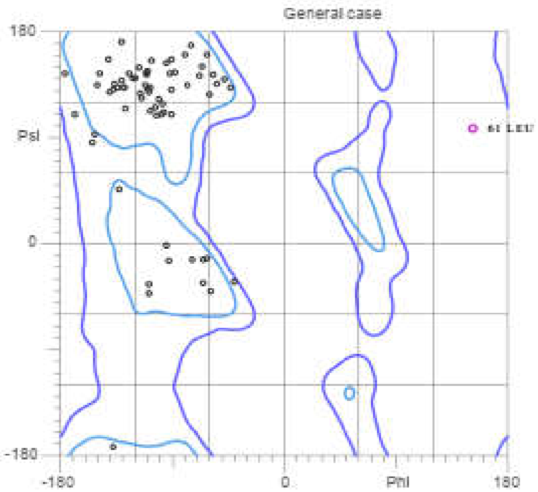

[a] ROBO1.IG1 (WT);
93.3% favoured, 99% allowed

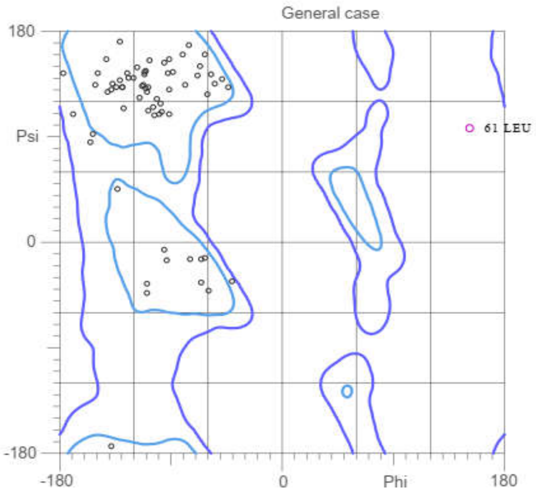

[a1] ROBO1.IG1 (R119Q);
93.3% favoured, 99% allowed

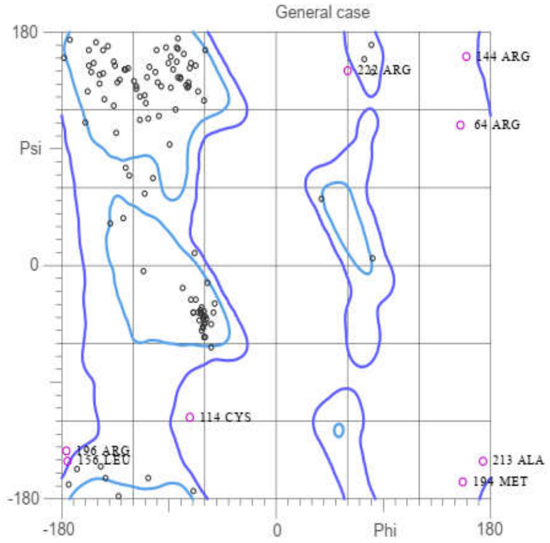

[b] ROBO4.IG1-2 (WT);
75% favoured, 91.3% allowed

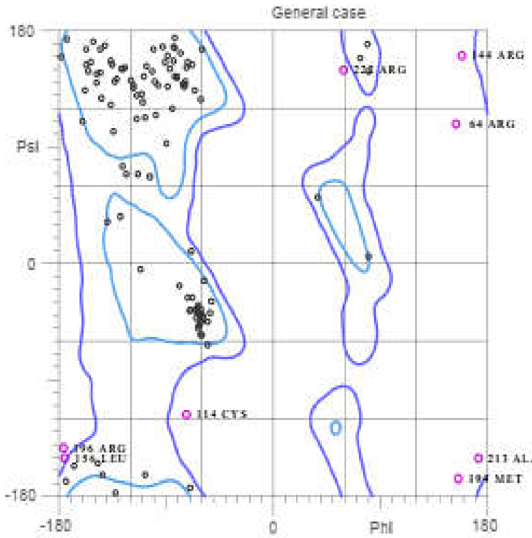

[b1] ROBO4.IG1-2 (C207S);
75% favoured, 91.3% allowed

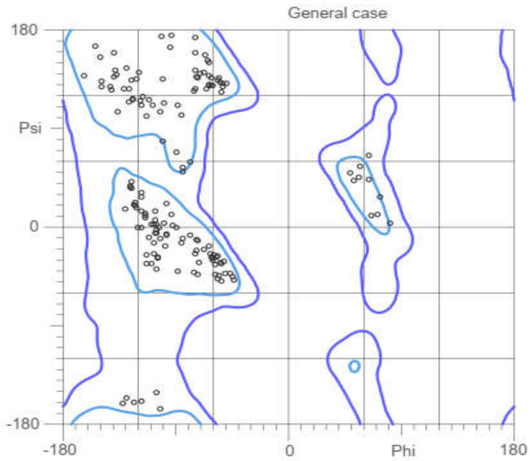

[c] SLIT2.D2 (WT);
95.2% favoured, 100% allowed

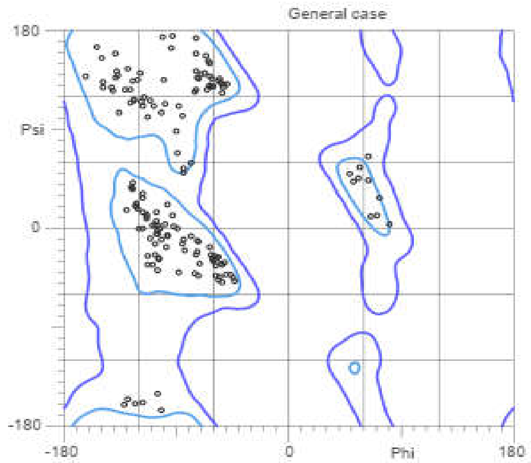

[c1] SLIT2.D2 (Y323H)
95.7% favoured, 100% allowed

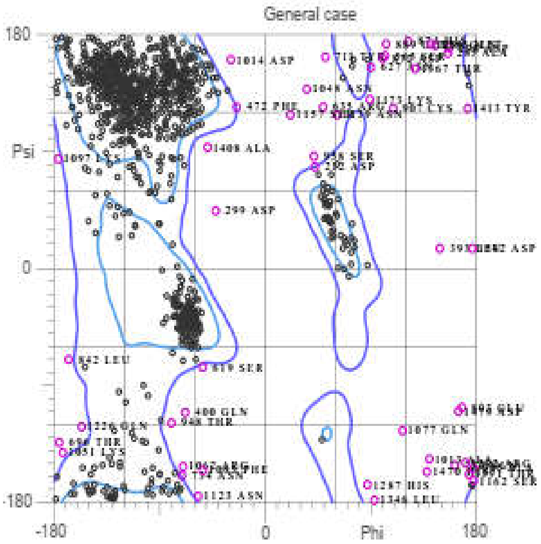

[d] ROBO1 (WT);
82.6% favoured, 93.8% allowed

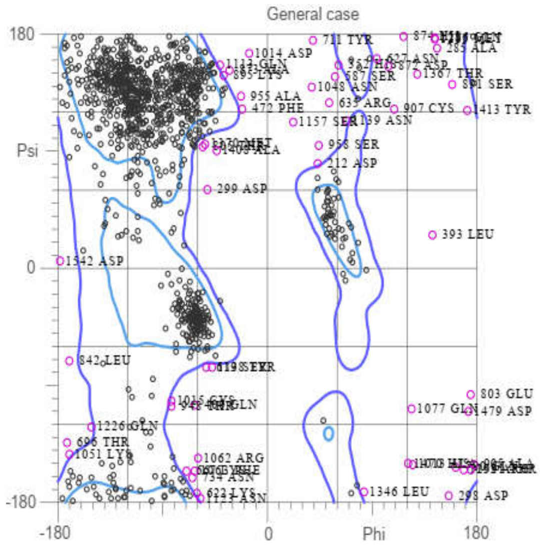

[d1] ROBO1 (D312V)
80% favoured, 93.3% allowed

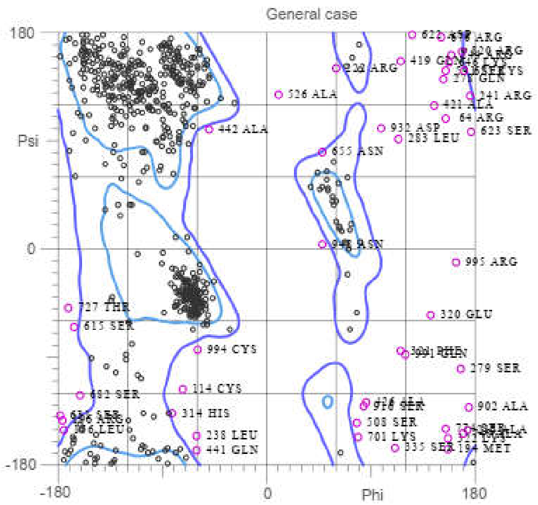

[e] ROBO4 (WT)
77.9% favoured, 91.8% allowed

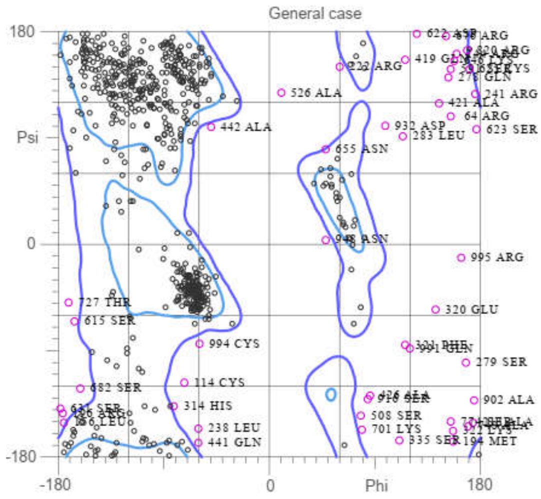

[e1] ROBO4 (W367C)
77.9% favoured, 91.8% allowed

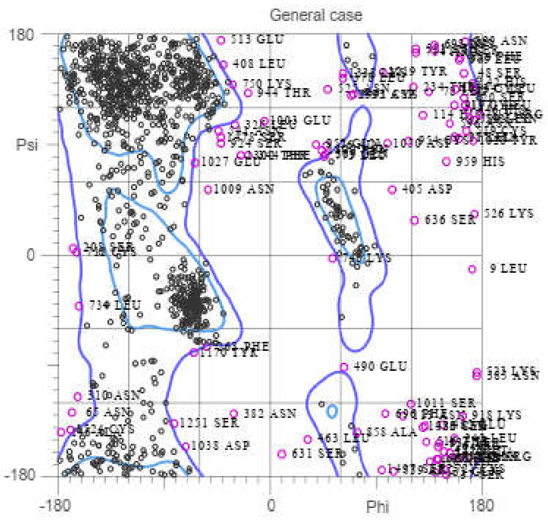

[f] SLIT2 (WT)
73.4% favoured, 90.3% allowed

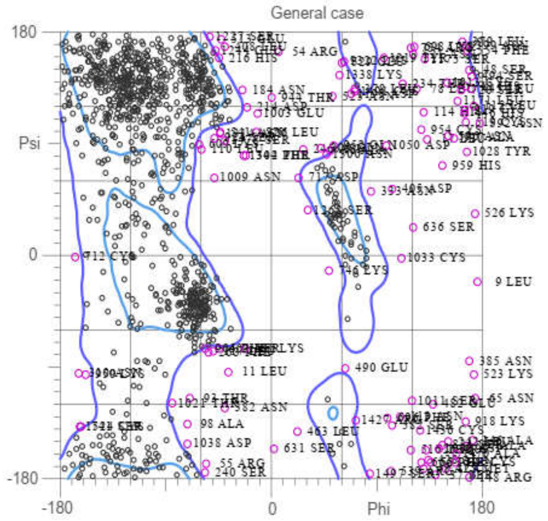

[f1] SLIT2 (P54R)
69.2% favoured, 88.2% allowed

Fig. S5

Fig. S5. Representative Ramachandran Plots of the top-scoring mutant structures with their respective wild types for both partial interacting domains and whole protein homology models. *The percentages of amino acids favoured and in the allowed regions are depicted. The images were obtained from the MolProbity Server (http://molprobity.manchester.ac.uk/).*

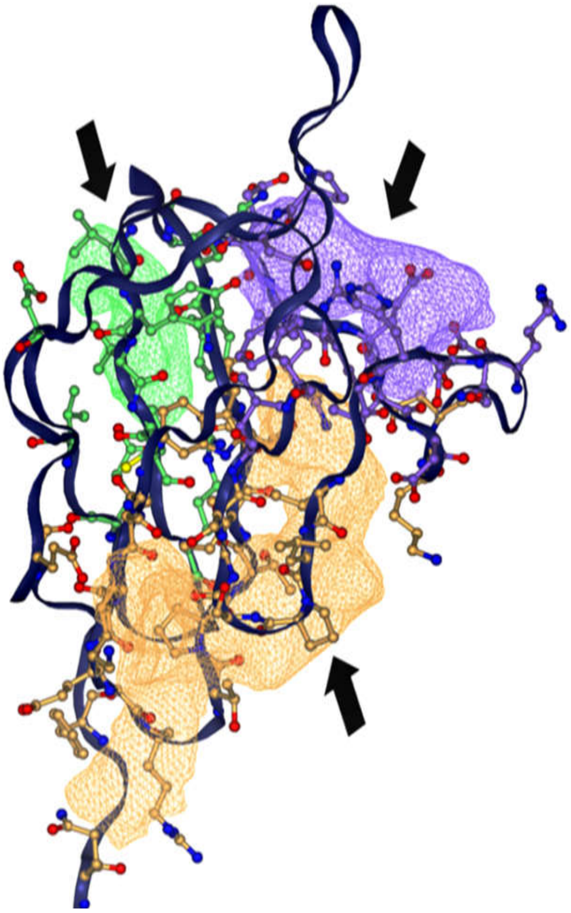

[a]

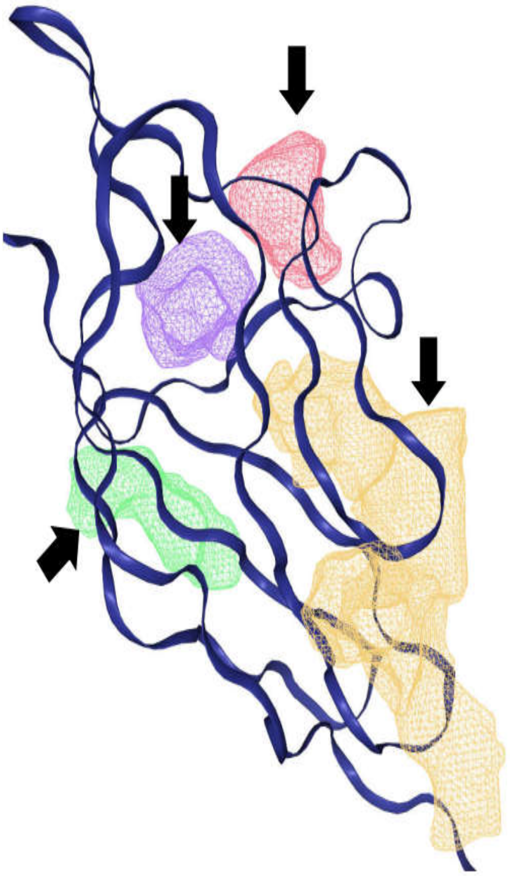

[a’]

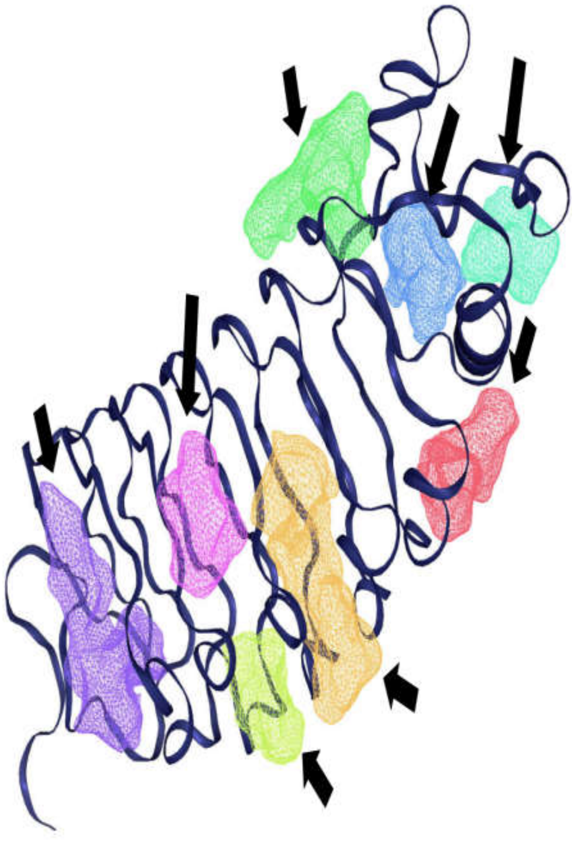

[b]

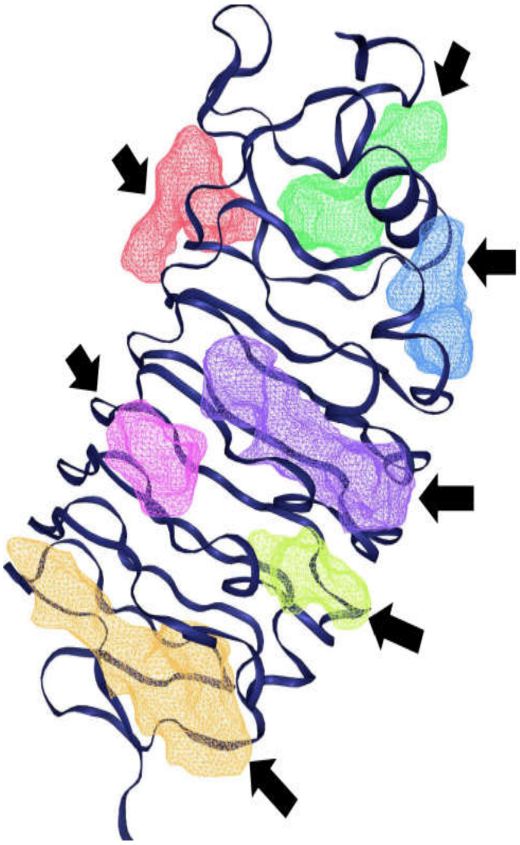

[b’]

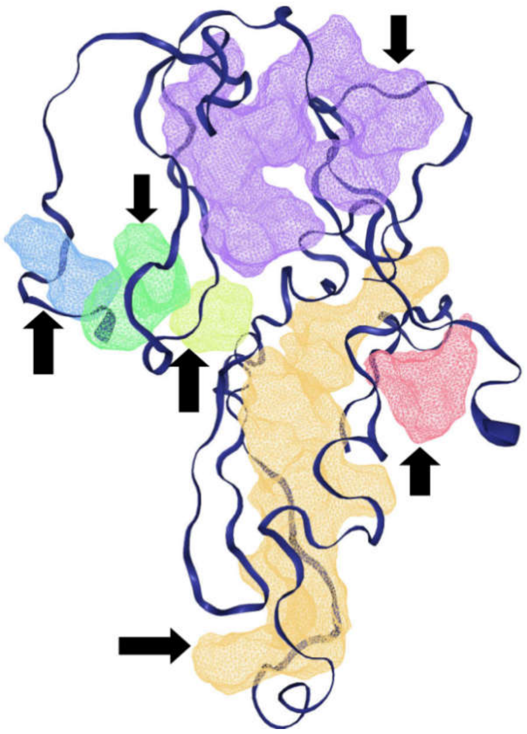

[c]

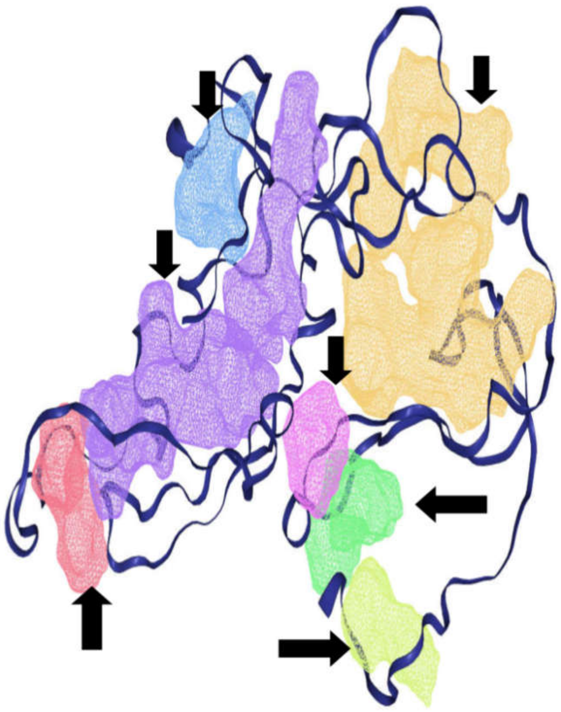

[c’]

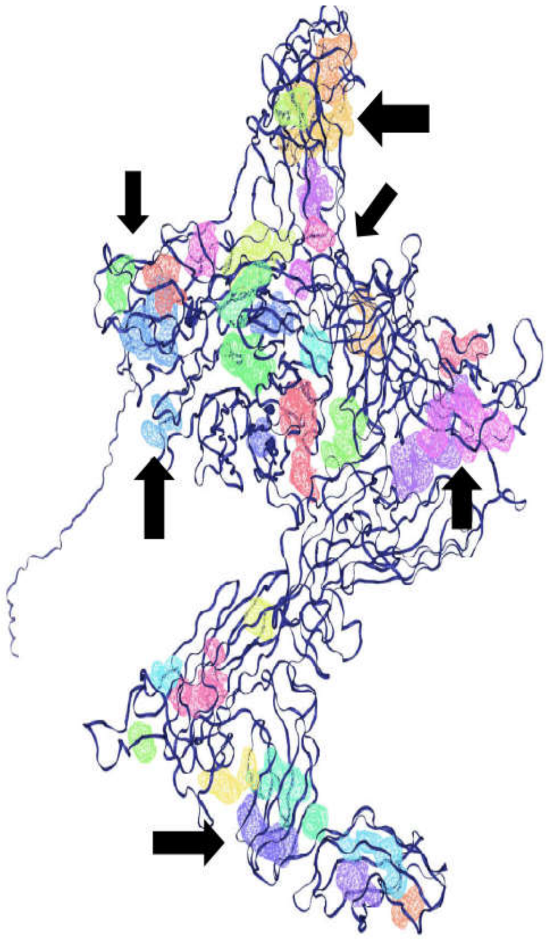

[d]

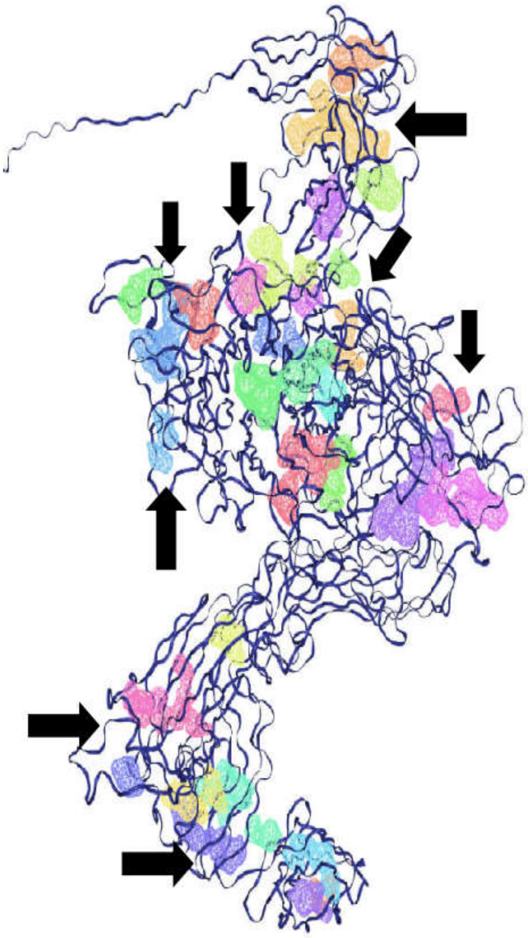

[d’]

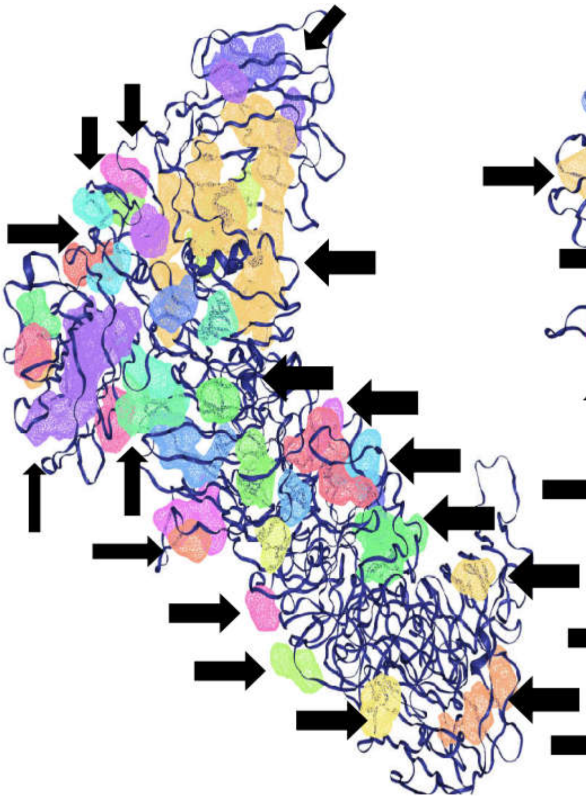

[e]

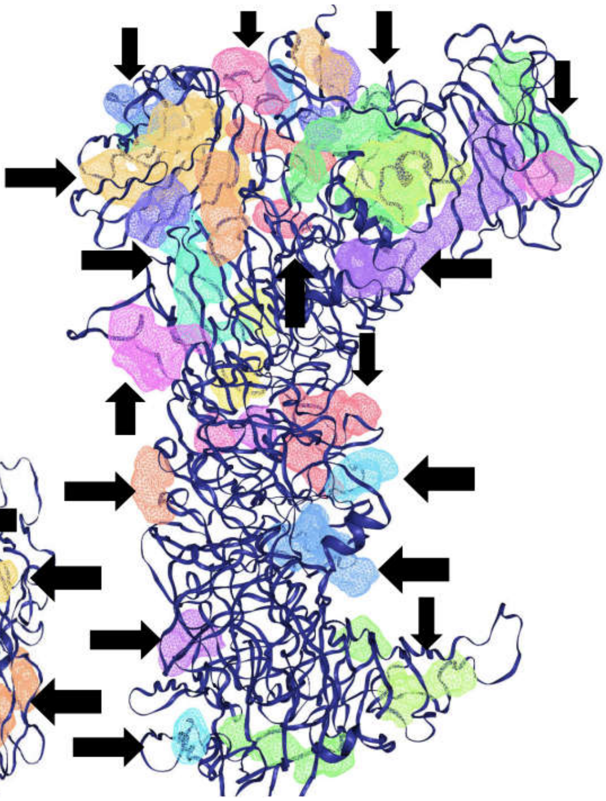

[e']

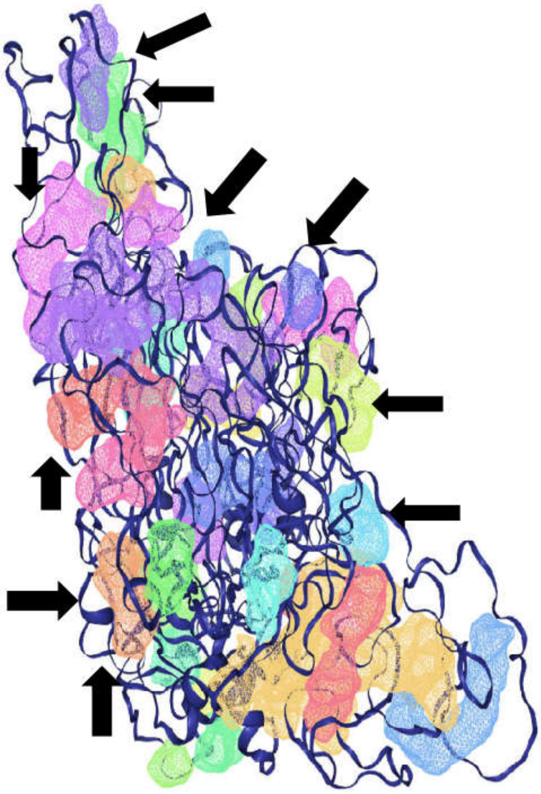

[f]

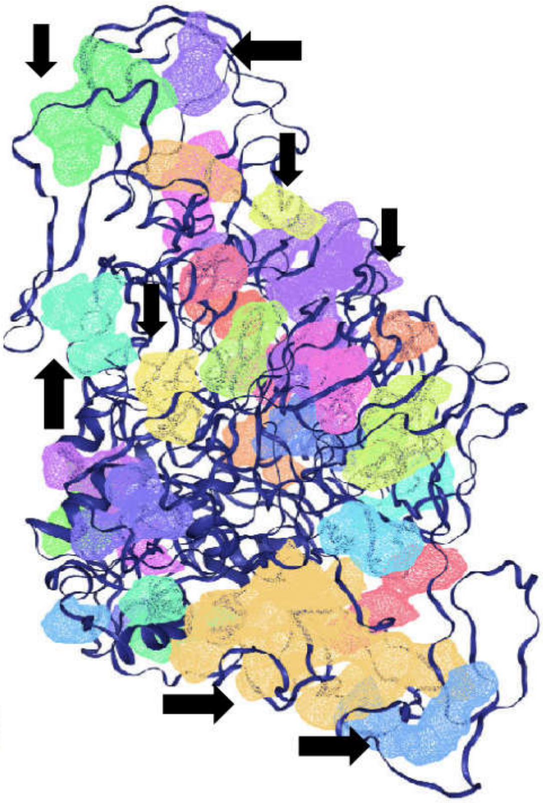

[f']

Fig. S6

Fig. S6. Representative images of druggable/ligand binding pockets in mutants compared to respective wild types. [a] ROBO1.IG1 (wild type) with 3 pockets, **[a']** ROBO1.IG1 (K103N) with 4 pockets, **[b]** SLIT2.D2 (wild type) with 8 pockets, **[b']** SLIT2.D2 (Y323H) with 7 pockets, **[c]** ROBO4.IG1-2 (wild type) with 6 pockets, **[c']** ROBO4.IG1-2 (G102C) with 7 pockets, **[d]** ROBO1-full protein (wild type) with 34 pockets, **[d']** ROBO1-full protein (P635S) with 32 pockets, **[e]** SLIT2-full protein (wild type) with 37 pockets, **[e']** SLIT2-full protein (P54R) with 35 pockets, **[f]** ROBO4-full protein (wild type) with 32 pockets, **[f']** ROBO4-full protein (W367C) with 31 pockets. *Black arrows (bold) indicate the position of drug/ligand binding pockets*. The images were obtained from the DoGSiteScorer webserver (https://proteins.plus/).

Table S1. The entire list of missense mutations of ROBO1, ROBO4, and SLIT2, prioritised by sequence-based parameters.

GENES	MUTATIONS	PROVEAN	SIFT	POLYPHEN2	SNPs&GO	iMUTANT 2.0	FATHMM	TOTAL SCORE
**[I] Lung Cancer Dataset**								
**[A] Mutations within docking domains**								
ROBO1	p.F129L	1	0	2	1	1	0	5
ROBO1	p.R119Q	1	1	2	1	1	0	6
ROBO1	p.K103N	1	1	2	1	1	0	6
ROBO1	p.S75P	0	0	2	0	1	0	3
SLIT2	p.D435V	1	1	2	1	1	0	6
SLIT2	p.E300V	1	1	1	0	0	0	3
SLIT2	p.I305T	1	1	1	1	1	0	5
SLIT2	p.I433V	0	0	0	0	1	0	1
SLIT2	p.K439M	1	1	2	0	0	0	4
SLIT2	p.R306L	1	1	2	1	1	0	6
SLIT2	p.T301N	0	0	1	0	1	0	2
SLIT2	p.Y323H	1	0	2	1	1	0	5
ROBO4	p.D78N	1	0	0	0	1	0	2
ROBO4	p.R136L	1	0	0	1	1	0	3
ROBO4	p.G102C	1	1	2	0	1	0	5
ROBO4	p.L42M	0	0	2	0	1	0	3
ROBO4	p.T210N	0	0	1	0	1	0	2
ROBO4	p.C207S	1	1	2	1	1	1	7

GENES	MUTATIONS	PROVEAN	SIFT	POLYPHEN2	SNPs&GO	iMUTANT 2.0	FATHMM	TOTAL SCORE
**[I] Lung Cancer Dataset**								
**[B] Mutations outside docking domains**								
ROBO1	p.E1637K	0	1	0	0	1	0	2
ROBO1	p.S1605N	0	1	1	0	1	0	3
ROBO1	p.P1593T	1	1	2	0	1	0	5
ROBO1	p.V1454L	0	0	0	0	0	0	0
ROBO1	p.R1420P	0	1	1	0	1	0	3
ROBO1	p.E1412V	0	1	1	0	0	0	2
ROBO1	p.P1217S	1	1	0	0	1	0	3
ROBO1	p.A1183S	0	1	0	0	0	0	1
ROBO1	p.Y1138H	0	0	0	0	1	0	1
ROBO1	p.G1103R	0	0	0	0	1	0	1
ROBO1	p.A1074S	0	0	1	0	1	0	2
ROBO1	p.L1006I	0	0	0	0	1	0	1
ROBO1	p.W816C	1	1	2	1	1	0	6
ROBO1	p.W816S	1	1	2	1	1	0	6
ROBO1	p.K750N	1	0	2	1	1	0	5
ROBO1	p.V737L	0	1	0	0	1	0	2
ROBO1	p.T730M	0	1	2	0	1	0	4
ROBO1	p.D722E	0	0	0	0	1	0	1
ROBO1	p.R635S	1	1	2	1	1	0	6
ROBO1	p.R420L	1	1	1	1	1	0	5
ROBO1	p.R318S	0	0	0	0	1	0	1
ROBO1	p.D312V	1	1	2	1	1	0	6
ROBO1	p.R306I	1	1	2	1	1	0	6
ROBO1	p.R296M	1	1	2	1	1	0	6
ROBO1	p.D276G	1	0	2	0	1	0	4
ROBO1	p.E260D	1	0	2	0	1	0	4
ROBO1	p.D213N	1	1	2	0	1	0	5
ROBO1	p.R60C	1	1	2	1	1	0	6
ROBO1	p.D51V	0	1	2	0	1	0	4
ROBO1	p.P31T	0	1	0	0	1	0	2

SLIT2	p.A1207T	1	1	2	0	1	0	5
SLIT2	p.C1482F	1	1	2	0	1	0	5
SLIT2	p.C500S	1	1	1	0	1	0	4
SLIT2	p.C510F	1	1	2	1	1	0	6
SLIT2	p.D1000N	1	1	0	0	0	0	2
SLIT2	p.D1445H	0	0	1	0	1	0	2
SLIT2	p.D491G	1	1	1	1	1	0	5
SLIT2	p.D503N	1	1	0	0	1	0	3
SLIT2	p.D704N	1	1	2	0	1	0	5
SLIT2	p.D939Y	0	1	0	1	0	0	2
SLIT2	p.E1399K	0	0	0	0	1	0	1
SLIT2	p.E529Q	0	0	0	0	1	0	1
SLIT2	p.G1306C	1	1	2	0	1	0	5
SLIT2	p.G1403C	1	1	2	0	1	0	5
SLIT2	p.G1424W	1	1	2	0	1	0	5
SLIT2	p.G43R	1	1	2	0	1	0	5
SLIT2	p.G97A	1	1	1	0	1	0	4
SLIT2	p.H197L	1	1	0	1	0	0	3
SLIT2	p.K1396N	0	1	0	0	1	0	2
SLIT2	p.K1518I	0	0	2	0	0	0	2
SLIT2	p.L548F	1	1	2	1	1	0	6
SLIT2	p.M8I	0	0	0	0	1	0	1
SLIT2	p.N1087T	1	1	2	0	0	0	4
SLIT2	p.N1300K	1	1	2	0	1	0	5
SLIT2	p.N1372Y	0	0	1	0	1	0	2
SLIT2	p.N161K	1	1	2	1	1	0	6
SLIT2	p.N866K	0	0	0	0	1	0	1
SLIT2	p.P1374H	1	1	2	0	1	0	5
SLIT2	p.P1494R	0	0	2	0	1	0	3
SLIT2	p.P54R	1	1	2	1	1	0	6
SLIT2	p.P665S	1	1	2	1	1	0	6
SLIT2	p.P946T	1	0	1	0	1	0	3
SLIT2	p.Q1463E	0	0	1	0	0	0	1
SLIT2	p.Q235R	0	0	0	0	1	0	1

SLIT2	p.Q251E	0	0	1	0	0	0	1
SLIT2	p.Q261R	0	0	0	0	1	0	1
SLIT2	p.R1427S	0	0	0	0	1	0	1
SLIT2	p.R144M	1	1	2	1	1	0	6
SLIT2	p.R1526T	0	0	0	0	1	0	1
SLIT2	p.R227S	1	0	1	0	1	0	3
SLIT2	p.R55C	1	1	2	1	1	0	6
SLIT2	p.R633C	1	1	2	1	1	0	6
SLIT2	p.S607G	0	0	0	0	1	0	1
SLIT2	p.T1302N	0	0	0	0	1	0	1
SLIT2	p.T546I	1	1	0	1	1	0	4
SLIT2	p.T686R	1	1	2	1	1	0	6
SLIT2	p.V1243A	1	0	1	0	1	0	3
SLIT2	p.W219L	1	1	2	1	1	0	6
SLIT2	p.Y1442F	0	0	0	0	1	0	1
ROBO4	p.G829W	1	1	2	1	1	0	6
ROBO4	p.N374S	0	0	0	0	1	0	1
ROBO4	p.R627H	0	0	0	0	1	0	1
ROBO4	p.C994Y	0	0	0	0	1	0	1
ROBO4	p.G920S	0	0	1	0	1	0	2
ROBO4	p.R491C	0	1	2	1	1	0	5
ROBO4	p.D622E	0	0	2	0	0	0	2
ROBO4	p.P290T	0	0	1	0	1	0	2
ROBO4	p.L574P	0	1	2	1	1	0	5
ROBO4	p.W367C	1	1	2	1	1	1	7
ROBO4	p.V337E	1	1	2	1	1	0	6
ROBO4	p.G311V	0	0	2	1	1	0	4
ROBO4	p.L624F	0	1	2	0	1	0	4
ROBO4	p.S451R	0	0	0	0	0	0	0
ROBO4	p.K647Q	0	0	1	0	0	0	1
ROBO4	p.P716S	1	0	0	0	1	0	2
ROBO4	p.A295V	0	1	2	0	0	0	3
ROBO4	p.G328R	0	1	2	0	1	0	4
ROBO4	p.D903H	1	1	2	1	1	0	6

ROBO4	p.R530S	0	1	2	1	1	0	5
ROBO4	p.L17Q	0	1	2	0	0	0	3
ROBO4	p.Q229K	0	0	0	0	0	0	0
ROBO4	p.R560C	0	1	0	1	1	0	3
ROBO4	p.G975R	0	0	0	0	0	0	0
ROBO4	p.L20M	0	1	1	0	1	0	3
ROBO4	p.W397C	1	1	0	1	1	0	4
ROBO4	p.Q306H	0	0	2	0	1	0	3
ROBO4	p.R462K	0	0	0	0	1	0	1

GENES	MUTATIONS	PROVEAN	SIFT	POLYPHEN2	SNPs&GO	iMUTANT 2.0	FATHMM	TOTAL SCORE
**[I] Non-Lung Cancer Dataset**								
**[A] Mutations within docking domains**								
ROBO1	p.R62H	1	1	2	0	1	0	5
ROBO1	p.R69H	1	1	2	0	1	0	5
ROBO1	p.I78S	1	1	2	1	1	0	6
ROBO1	p.G82V	1	1	2	1	1	0	6
ROBO1	p.A85T	0	0	2	0	1	0	3
ROBO1	p.A91G	1	1	2	1	1	0	6
ROBO1	p.R94C	1	1	2	1	1	0	6
ROBO1	p.R94H	1	1	2	0	1	0	5
ROBO1	p.G104V	0	1	2	1	1	0	5
ROBO1	p.E106D	0	0	0	0	1	0	1
ROBO1	p.T110I	1	1	2	0	1	0	5
ROBO1	p.R116S	1	1	2	0	1	0	5
ROBO1	p.P123S	1	1	2	0	1	0	5
ROBO1	p.P123L	1	1	2	1	1	0	6
ROBO1	p.R131C	1	1	2	1	1	0	6
ROBO1	p.G135E	1	1	2	0	1	0	5
ROBO1	p.G135V	1	1	2	1	1	0	6
ROBO1	p.G143R	1	1	2	1	1	0	6
ROBO1	p.V146F	1	0	2	1	1	0	5
ROBO1	p.C147Y	1	1	2	1	1	1	7
ROBO1	p.R150K	0	0	2	1	1	0	4

ROBO1	p.G154E	1	1	2	1	1	0	6
SLIT2	p.L400I	0	1	0	0	1	0	2
SLIT2	p.S417L	1	0	0	1	0	0	2
SLIT2	p.F372C	0	0	2	0	1	0	3
SLIT2	p.C436F	1	1	2	1	1	0	6
SLIT2	p.R467I	1	1	2	0	1	0	5
SLIT2	p.E304K	1	1	2	0	1	0	5
SLIT2	p.R388W	1	1	2	1	1	0	6
SLIT2	p.R456H	1	1	2	1	1	0	6
SLIT2	p.A276T	0	0	1	0	1	0	2
SLIT2	p.A412T	0	1	1	0	1	0	3
SLIT2	p.N430K	1	1	2	1	1	0	6
SLIT2	p.L298I	0	0	0	0	1	0	1
SLIT2	p.K474N	1	1	0	0	1	0	3
SLIT2	p.D405N	0	0	0	0	1	0	1
SLIT2	p.R461C	1	1	2	1	1	0	6
SLIT2	p.A421T	0	1	1	0	1	0	3
SLIT2	p.A275V	0	0	1	0	0	0	1
SLIT2	p.R456C	1	1	2	1	1	0	6
SLIT2	p.R348C	1	1	2	1	1	0	6
SLIT2	p.Q393R	0	0	0	1	0	0	1
SLIT2	p.E362K	1	1	1	0	1	0	4
SLIT2	p.R420W	1	1	2	1	1	0	6
SLIT2	p.S373F	1	1	1	1	0	0	4
SLIT2	p.I305M	0	1	0	1	1	0	3
SLIT2	p.L350V	1	1	2	1	1	0	6
SLIT2	p.L376I	0	1	2	0	1	0	4
SLIT2	p.T311A	0	0	0	0	1	0	1
SLIT2	p.N406S	1	1	2	1	1	0	6
SLIT2	p.P431L	1	1	2	1	1	0	6
SLIT2	p.L331P	1	1	2	1	1	0	6
SLIT2	p.L398F	1	1	2	1	1	0	6
SLIT2	p.L371P	1	1	2	1	0	0	5
SLIT2	p.S352L	1	1	2	1	1	0	6

SLIT2	p.A455V	1	1	2	0	0	0	4
SLIT2	p.S349F	1	1	2	1	0	0	5
SLIT2	p.S417A	0	0	0	0	1	0	1
SLIT2	p.Q345L	1	1	0	1	1	0	4
SLIT2	p.S366G	0	0	0	0	1	0	1
SLIT2	p.G346R	1	1	2	1	1	0	6
SLIT2	p.N448S	1	1	0	1	1	0	4
SLIT2	p.S402F	1	1	2	1	0	0	5
SLIT2	p.L376F	1	1	2	0	1	0	5
SLIT2	p.R462C	1	1	2	1	1	0	6
SLIT2	p.A340T	1	1	2	0	1	0	5
SLIT2	p.P322L	1	1	2	0	1	0	5
ROBO4	p.P46S	0	0	0	0	1	0	1
ROBO4	p.P60L	0	0	0	0	1	0	1
ROBO4	p.P71L	0	0	2	0	1	0	3
ROBO4	p.M74I	0	0	0	0	1	0	1
ROBO4	p.H80Y	0	0	0	0	0	0	0
ROBO4	p.D85H	0	1	1	0	1	0	3
ROBO4	p.G86E	1	1	2	1	0	0	5
ROBO4	p.Q92P	0	1	0	0	1	0	2
ROBO4	p.R96W	0	1	2	0	1	0	4
ROBO4	p.D108N	1	0	0	0	0	0	1
ROBO4	p.R119W	0	0	2	1	1	0	4
ROBO4	p.E137K	0	0	0	0	1	0	1
ROBO4	p.M146T	0	0	0	0	1	0	1
ROBO4	p.H164Y	1	0	0	0	0	0	1
ROBO4	p.P165S	1	1	2	1	1	0	6
ROBO4	p.T168I	1	0	0	0	0	0	1
ROBO4	p.A179T	0	0	0	0	1	0	1
ROBO4	p.P182L	1	0	0	0	1	0	2
ROBO4	p.G183R	0	1	2	0	0	0	3
ROBO4	p.G189W	1	1	2	1	1	0	6
ROBO4	p.S191F	1	1	1	0	0	0	3
ROBO4	p.A197V	0	0	0	0	1	0	1

ROBO4	p.C207F	1	1	2	1	1	1	7
ROBO4	p.R219C	1	1	2	1	1	0	6
ROBO4	p.A221S	0	1	2	0	1	0	4
ROBO4	p.R222Q	0	0	1	0	1	0	2
ROBO4	p.S224F	0	0	0	0	0	0	0

Table S2. The list of missense mutations of ROBO1, ROBO4, and SLIT2, prioritised by structure-based parameters.

PROTEINS	VARIANTS	PROVEAN	SIFT	POLYPHEN2	SNPs&GO	iMUTANT	FATHMM	H-BOND	STERIC CLASH	ASA	TOTAL SCORE
**[A] Lung Cancer Dataset**											
**[I] Mutations within Docking Domain**											
ROBO1	p.R119Q	1	1	2	1	1	0	1	0	1	8
ROBO1	p.K103N	1	1	2	1	1	0	1	0	1	8
SLIT2	p.D435V	1	1	2	1	1	0	1	1	1	9
SLIT2	p.R306L	1	1	2	1	1	0	1	1	1	9
SLIT2	p.Y323H	1	0	2	1	1	0	1	1	1	8
ROBO4	p.G102C	1	1	2	0	1	0	1	1	1	8
ROBO4	p.C207S	1	1	2	1	1	1	1	0	0	8
**[II] Mutations outside Docking Domain**											
ROBO1	p.R635S	1	1	2	1	1	0	1	1	1	9
ROBO1	p.D312V	1	1	2	1	1	0	1	1	1	9
ROBO1	p.R306I	1	1	2	1	1	0	1	1	1	9
SLIT2	p.P54R	1	1	2	1	1	0	1	1	1	9
SLIT2	p.P665S	1	1	2	1	1	0	1	1	0	8
ROBO4	p.G829W	1	1	2	1	1	0	1	1	1	9
ROBO4	p.W367C	1	1	2	1	1	1	1	0	1	9
**[B] Non-Lung Cancer Dataset**											
**[I] Mutations within Docking Domain**											
ROBO1	p.I78S	1	1	2	1	1	0	1	0	1	8
ROBO1	p.G82V	1	1	2	1	1	0	1	0	1	8
ROBO1	p.P123L	1	1	2	1	1	0	1	1	0	8
ROBO1	p.R131C	1	1	2	1	1	0	1	0	1	8
ROBO1	p.G143R	1	1	2	1	1	0	1	0	1	8
ROBO1	p.G154E	1	1	2	1	1	0	1	0	1	8
SLIT2	p.C436F	1	1	2	1	1	0	1	1	1	9

PROTEINS	VARIANTS	PROVEAN	SIFT	POLYPHEN2	SNPs&GO	iMUTANT	FATHMM	H-BOND	STERIC CLASH	ASA	TOTAL SCORE
**[B] Non-Lung Cancer Dataset**											
**[I] Mutations within Docking Domain**											
SLIT2	p.R388W	1	1	2	1	1	0	1	1	1	9
SLIT2	p.R456H	1	1	2	1	1	0	1	1	1	9
SLIT2	p.N430K	1	1	2	1	1	0	1	1	1	9
SLIT2	p.R420W	1	1	2	1	1	0	0	1	1	8
SLIT2	p.L350V	1	1	2	1	1	0	0	1	1	8
SLIT2	p.P431L	1	1	2	1	1	0	1	1	1	9
SLIT2	p.L331P	1	1	2	1	1	0	1	1	0	8
SLIT2	p.L398F	1	1	2	1	1	0	1	1	0	8
SLIT2	p.L371P	1	1	2	1	0	0	1	1	1	8
SLIT2	p.S352L	1	1	2	1	1	0	1	1	1	9
SLIT2	p.R462C	1	1	2	1	1	0	1	1	1	9
SLIT2	p.A340T	1	1	2	0	1	0	1	1	1	8
SLIT2	p.P322L	1	1	2	0	1	0	1	1	1	8
SLIT2	p.R461C	1	1	2	1	1	0	1	0	1	8
ROBO4	p.G86E	1	1	2	1	0	0	1	1	1	8
ROBO4	p.C207F	1	1	2	1	1	1	0	1	1	9
**[II] Mutations outside Docking Domain**											
ROBO1	p.P176L	1	1	2	1	1	0	1	1	1	9
ROBO1	p.G337V	1	1	2	1	1	0	1	1	0	8
ROBO1	p.C476Y	1	1	2	1	1	0	1	1	0	8
SLIT2	p.C215Y	1	1	2	1	0	1	1	1	0	8
SLIT2	p.G576R	1	1	2	1	1	0	1	1	0	8
SLIT2	p.S636C	1	1	2	1	1	0	1	0	1	8
ROBO4	p.G425V	1	1	2	1	1	0	1	1	0	8
ROBO4	p.G876V	1	1	2	1	1	0	1	1	0	8

Table S3. List of Q-Mean scores of the wild type and mutant models of the three proteins ROBO1, ROBO4, and SLIT2 for both the interacting domains and whole proteins. *The Q-Mean score values of the structures as obtained from https://swissmodel.expasy.org/qmean/.*

ROBO1	Q-MEAN SCORE	ROBO4	Q-MEAN SCORE	SLIT2	Q-MEAN SCORE
**[I] DOMAINS**		**[I] DOMAINS**		**[I] DOMAINS**	
WILD TYPE	0.76	WILD TYPE	-12.17	WILD TYPE	-0.48
**[A] MUTANTS FROM LUNG CANCER DATASET**		**[A] MUTANTS FROM LUNG CANCER DATASET**		**[A] MUTANTS FROM LUNG CANCER DATASET**	
p.R119Q	0.74	G102C	-12.13	D435V	-0.61
p.K103N	0.78	C207S	-11.99	R306L	-0.53
				Y323H	-0.47
**[B] MUTANTS FROM NON-LUNG CANCER DATASET**		**[B] MUTANTS FROM NON-LUNG CANCER DATASET**		**[B] MUTANTS FROM NON-LUNG CANCER DATASET**	
p.I78S	0.65	G86E	-12.21	C436F	-0.66
p.G82V	-0.06	C207F	-11.95	R388W	-0.46
p.P123L	0.27			R456H	-0.48
p.R131C	0.56			N430K	-0.87
p.G143R	0.14			R461C	-0.59
p.G154E	0.04			R420W	-0.47
				L350V	-0.39
				P431L	-0.56
				L331P	-1.67
				L398F	-1.28
				L371P	-0.64
				S352L	-0.47
				R462C	-0.59
				A340T	-0.53
				P322L	-0.5
**ROBO1**	**Q-MEAN SCORE**	**ROBO4**	**Q-MEAN SCORE**	**SLIT2**	**Q-MEAN SCORE**
**[II] FULL PROTEINS**		**[II] FULL PROTEINS**		**[II] FULL PROTEINS**	
WILD TYPE	-12.45	WILD TYPE	-15.89	WILD TYPE	-19.52
**[A] MUTANTS FROM LUNG CANCER DATASET**		**[A] MUTANTS FROM LUNG CANCER DATASET**		**[A] MUTANTS FROM LUNG CANCER DATASET**	
p.R635S	-12.46	G829W	-15.97	P54R	-19.53
p.D312V	-12.48	W367C	-15.9	P665S	-19.43
p.R306I	-12.45				
**[B] MUTANTS FROM NON-LUNG CANCER DATASET**		**[B] MUTANTS FROM NON-LUNG CANCER DATASET**		**[B] MUTANTS FROM NON-LUNG CANCER DATASET**	
p.P176L	-12.46	G425V	-16	C215Y	-19.51
p.G337V	-12.55	G876V	-16.07	G576R	-19.6
p.C476Y	-12.48			S636C	-19.46

Table S4. Assessment of alteration in ligand-binding potential and the number of ligand-binding pockets in the protein structures.

GENES	MUTATIONS	DRUGGABILITY	NUMBER OF POCKETS
**[A] Lung Cancer Dataset**			
**[I] Mutations within Docking Domain**			
ROBO1	p.R119Q	Unfavourable	Neutral
ROBO1	p.K103N	Unfavourable	Increase
SLIT2	p.D435V	Unfavourable	Neutral
SLIT2	p.R306L	Favourable	Neutral
SLIT2	p.Y323H	Favourable	Decrease
ROBO4	p.G102C	Favourable	Increase
ROBO4	p.C207S	Favourable	Neutral
**[II] Mutations outside Docking Domain**			
ROBO1	p.R635S	Favourable	Decrease
ROBO1	p.D312V	Favourable	Neutral
ROBO1	p.R306I	Favourable	Neutral
SLIT2	p.P54R	Favourable	Decrease
SLIT2	p.P665S	Favourable	Decrease
ROBO4	p.G829W	Favourable	Neutral
ROBO4	p.W367C	Favourable	Decrease
**[B] Non-Lung Cancer Dataset**			
**[I] Mutations within Docking Domain**			
ROBO1	p.I78S	Unfavourable	Neutral
ROBO1	p.G82V	Unfavourable	Neutral
ROBO1	p.P123L	Unfavourable	Neutral
ROBO1	p.R131C	Unfavourable	Increase
ROBO1	p.G143R	Unfavourable	Increase
ROBO1	p.G154E	Unfavourable	Increase
SLIT2	p.C436F	Favourable	Neutral
SLIT2	p.R388W	Favourable	Neutral
SLIT2	p.R456H	Favourable	Decrease
SLIT2	p.N430K	Favourable	Decrease
SLIT2	p.R420W	Favourable	Neutral
SLIT2	p.L350V	Favourable	Decrease
SLIT2	p.P431L	Favourable	Neutral
SLIT2	p.L331P	Unfavourable	Neutral
SLIT2	p.L398F	Favourable	Decrease
SLIT2	p.L371P	Favourable	Decrease
SLIT2	p.S352L	Favourable	Decrease
SLIT2	p.R462C	Favourable	Neutral
SLIT2	p.A340T	Favourable	Neutral
SLIT2	p.P322L	Favourable	Decrease
SLIT2	p.R461C	Favourable	Neutral
ROBO4	p.G86E	Favourable	Neutral
ROBO4	p.C207F	Unfavourable	Increase
**[II] Mutations outside Docking Domain**			
ROBO1	p.P176L	Unfavourable	Neutral
ROBO1	p.G337V	Favourable	Neutral
ROBO1	p.C476Y	Unfavourable	Neutral
SLIT2	p.C215Y	Favourable	Decrease
SLIT2	p.G576R	Favourable	Decrease
SLIT2	p.S636C	Favourable	Decrease
ROBO4	p.G425V	Neutral	Neutral
ROBO4	p.G876V	Favourable	Neutral

Table S5. The residue-specific interaction between the cognate docking domains of ROBO1/4 and SLIT2. The interaction files are ordered following the order of the complexes in Fig 2.

[A] ROBO1.IG1 (WT) + SLIT2.D2 (WT)

List of atom-atom interactions across protein-protein interface
----

Hydrogen bonds

	<----- A T O M 1 ----->						<----- A T O M 2 -------->					
	Atom no.	Atom name	Res name	Res no.	Chain		Atom no.	Atom name	Res name	Res no.	Chain	Distance
1.	109	OE1	GLU	72	A	<-->	1124	NH1	ARG	306	B	2.82
2.	110	OE2	GLU	72	A	<-->	1125	NH2	ARG	306	B	2.93
3.	133	OG	SER	75	A	<-->	979	NH1	ARG	287	B	3.25
4.	210	OG1	THR	86	A	<-->	1526	OH	TYR	356	B	2.67
5.	210	OG1	THR	86	A	<-->	1526	OH	TYR	356	B	2.67
6.	226	OD1	ASN	88	A	<-->	1309	NH2	ARG	328	B	3.15
7.	242	NZ	LYS	90	A	<-->	1105	OE1	GLU	304	B	2.74

Non-bonded contacts

	<----- A T O M 1 ----->						<----- A T O M 2 -------->					
	Atom no.	Atom name	Res name	Res no.	Chain		Atom no.	Atom name	Res name	Res no.	Chain	Distance
1.	108	CD	GLU	72	A	<-->	1124	NH1	ARG	306	B	3.42
2.	108	CD	GLU	72	A	<-->	1125	NH2	ARG	306	B	3.77
3.	109	OE1	GLU	72	A	<-->	1123	CZ	ARG	306	B	3.79
4.	109	OE1	GLU	72	A	<-->	1124	NH1	ARG	306	B	2.82
5.	109	OE1	GLU	72	A	<-->	1125	NH2	ARG	306	B	3.87
6.	110	OE2	GLU	72	A	<-->	978	CZ	ARG	287	B	3.63
7.	110	OE2	GLU	72	A	<-->	979	NH1	ARG	287	B	3.58
8.	110	OE2	GLU	72	A	<-->	980	NH2	ARG	287	B	3.70
9.	110	OE2	GLU	72	A	<-->	1123	CZ	ARG	306	B	3.57
10.	110	OE2	GLU	72	A	<-->	1124	NH1	ARG	306	B	3.36
11.	110	OE2	GLU	72	A	<-->	1125	NH2	ARG	306	B	2.93
12.	133	OG	SER	75	A	<-->	979	NH1	ARG	287	B	3.25
13.	148	CD1	LEU	77	A	<-->	1151	NE2	GLN	309	B	3.51
14.	197	CB	PRO	84	A	<-->	1713	ND2	ASN	380	B	3.42
15.	198	CG	PRO	84	A	<-->	1521	CD1	TYR	356	B	3.46
16.	198	CG	PRO	84	A	<-->	1713	ND2	ASN	380	B	3.55
17.	198	CG	PRO	84	A	<-->	1718	CB	ALA	381	B	3.82
18.	199	CD	PRO	84	A	<-->	1718	CB	ALA	381	B	3.83
19.	202	C	ALA	85	A	<-->	1526	OH	TYR	356	B	3.77
20.	205	N	THR	86	A	<-->	1526	OH	TYR	356	B	3.41
21.	209	CB	THR	86	A	<-->	1526	OH	TYR	356	B	3.34
22.	210	OG1	THR	86	A	<-->	1524	CE2	TYR	356	B	3.58
23.	210	OG1	THR	86	A	<-->	1525	CZ	TYR	356	B	3.51
24.	210	OG1	THR	86	A	<-->	1526	OH	TYR	356	B	2.67
25.	225	CG	ASN	88	A	<-->	1125	NH2	ARG	306	B	3.35
26.	226	OD1	ASN	88	A	<-->	1125	NH2	ARG	306	B	3.64
27.	226	OD1	ASN	88	A	<-->	1309	NH2	ARG	328	B	3.15
28.	227	ND2	ASN	88	A	<-->	1125	NH2	ARG	306	B	3.21
29.	240	CD	LYS	90	A	<-->	948	CD1	ILE	283	B	3.77

30.	241	CE	LYS	90	A	<-->	948	CD1	ILE	283	B	3.59
31.	241	CE	LYS	90	A	<-->	1105	OE1	GLU	304	B	3.45
32.	242	NZ	LYS	90	A	<-->	1104	CD	GLU	304	B	3.20
33.	242	NZ	LYS	90	A	<-->	1105	OE1	GLU	304	B	2.74
34.	242	NZ	LYS	90	A	<-->	1106	OE2	GLU	304	B	2.91
35.	242	NZ	LYS	90	A	<-->	1308	NH1	ARG	328	B	3.19
36.	481	SD	MET	120	A	<-->	2083	CE1	HIS	426	B	3.54
37.	481	SD	MET	120	A	<-->	2084	NE2	HIS	426	B	3.61
38.	482	CE	MET	120	A	<-->	1878	CD2	LEU	400	B	3.70
39.	521	OG	SER	126	A	<-->	1309	NH2	ARG	328	B	3.77
40.	538	CE1	PHE	128	A	<-->	1524	CE2	TYR	356	B	3.85
41.	557	CG	LEU	130	A	<-->	1906	CG	TYR	404	B	3.88
42.	557	CG	LEU	130	A	<-->	1908	CD2	TYR	404	B	3.84
43.	558	CD1	LEU	130	A	<-->	1892	OG	SER	402	B	3.44
44.	558	CD1	LEU	130	A	<-->	1905	CB	TYR	404	B	3.71
45.	558	CD1	LEU	130	A	<-->	1906	CG	TYR	404	B	3.83
46.	558	CD1	LEU	130	A	<-->	1908	CD2	TYR	404	B	3.81
47.	558	CD1	LEU	130	A	<-->	2081	ND1	HIS	426	B	3.64
48.	559	CD2	LEU	130	A	<-->	2080	CG	HIS	426	B	3.64
49.	559	CD2	LEU	130	A	<-->	2081	ND1	HIS	426	B	3.72
50.	559	CD2	LEU	130	A	<-->	2303	CB	SER	453	B	3.83
51.	564	CB	ARG	131	A	<-->	1912	OH	TYR	404	B	3.82
52.	566	CD	ARG	131	A	<-->	1911	CZ	TYR	404	B	3.79
53.	566	CD	ARG	131	A	<-->	1912	OH	TYR	404	B	3.01

Salt bridges

<----- A T O M 1 -----> <----- A T O M 2 -------- >

Atom Atom Res Res Atom Atom Res Res
no. name name no. Chain no. name name no. Chain Distance 1. 110 OE2 GLU
72 A <--> 979 NH1 ARG 287 B 3.58
2. 109 OE1 GLU 72 A <--> 1124 NH1 ARG 306 B 2.82
3. 242 NZ LYS 90 A <--> 1105 OE1 GLU 304 B 2.74

Number of salt bridges: 3

Number of hydrogen bonds: 7

Number of non-bonded contacts: 53

[B] ROBO4.IG1-2 + SLIT2.D2 WILD TYPES

Hydrogen bonds

	<----- A T O M 1 ----->						<----- A T O M 2 ----->					
	Atom no.	Atom name	Res name	Res no.	Chain		Atom no.	Atom name	Res name	Res no.	Chain	Distance
1.	1163	O	GLY	189	A	<-->	42	N	ALA	275	B	3.27
2.	1258	OE1	GLU	202	A	<-->	5	N	SER	270	B	2.20
3.	1258	OE1	GLU	202	A	<-->	25	ND1	HIS	272	B	2.82
4.	1267	O	THR	204	A	<-->	233	OG1	THR	301	B	3.24
5.	1316	N	ASN	211	A	<-->	74	O	SER	280	B	3.32
6.	1322	OD1	ASN	211	A	<-->	76	OG	SER	280	B	3.29
7.	1330	N	ALA	213	A	<-->	115	OD2	ASP	285	B	2.73
8.	1335	N	GLY	214	A	<-->	63	OG1	THR	278	B	2.51
9.	1338	O	GLY	214	A	<-->	145	NZ	LYS	289	B	2.87

Non-bonded contacts

	<----- A T O M 1 ----->						<----- A T O M 2 ----->					
	Atom no.	Atom name	Res name	Res no.	Chain		Atom no.	Atom name	Res name	Res no.	Chain	Distance
1.	932	CB	PRO	160	A	<-->	4	O	GLY	269	B	3.51
2.	943	CA	TRP	162	A	<-->	1	N	GLY	269	B	3.49
3.	1098	CD2	LEU	180	A	<-->	46	CB	ALA	275	B	3.64
4.	1141	CA	THR	186	A	<-->	173	OE2	GLU	293	B	3.84
5.	1142	C	THR	186	A	<-->	194	OG1	THR	296	B	3.22
6.	1143	O	THR	186	A	<-->	194	OG1	THR	296	B	3.40
7.	1144	CB	THR	186	A	<-->	170	CG	GLU	293	B	3.04
8.	1144	CB	THR	186	A	<-->	171	CD	GLU	293	B	2.81
9.	1144	CB	THR	186	A	<-->	172	OE1	GLU	293	B	3.22
10.	1144	CB	THR	186	A	<-->	173	OE2	GLU	293	B	3.08
11.	1145	OG1	THR	186	A	<-->	169	CB	GLU	293	B	3.73
12.	1145	OG1	THR	186	A	<-->	170	CG	GLU	293	B	2.45
13.	1145	OG1	THR	186	A	<-->	171	CD	GLU	293	B	1.65
14.	1145	OG1	THR	186	A	<-->	172	OE1	GLU	293	B	2.24
15.	1145	OG1	THR	186	A	<-->	173	OE2	GLU	293	B	1.70
16.	1146	CG2	THR	186	A	<-->	169	CB	GLU	293	B	3.65
17.	1146	CG2	THR	186	A	<-->	170	CG	GLU	293	B	2.58
18.	1146	CG2	THR	186	A	<-->	171	CD	GLU	293	B	3.16
19.	1146	CG2	THR	186	A	<-->	172	OE1	GLU	293	B	3.78
20.	1146	CG2	THR	186	A	<-->	173	OE2	GLU	293	B	3.65
21.	1146	CG2	THR	186	A	<-->	176	C	ILE	294	B	3.85
22.	1146	CG2	THR	186	A	<-->	177	O	ILE	294	B	3.04
23.	1147	N	VAL	187	A	<-->	189	N	THR	296	B	3.17
24.	1147	N	VAL	187	A	<-->	190	CA	THR	296	B	3.79
25.	1147	N	VAL	187	A	<-->	193	CB	THR	296	B	3.25
26.	1147	N	VAL	187	A	<-->	194	OG1	THR	296	B	2.25
27.	1147	N	VAL	187	A	<-->	195	CG2	THR	296	B	3.46
28.	1148	CA	VAL	187	A	<-->	189	N	THR	296	B	2.77
29.	1148	CA	VAL	187	A	<-->	190	CA	THR	296	B	2.88
30.	1148	CA	VAL	187	A	<-->	191	C	THR	296	B	3.47
31.	1148	CA	VAL	187	A	<-->	192	O	THR	296	B	3.61
32.	1148	CA	VAL	187	A	<-->	193	CB	THR	296	B	2.19
33.	1148	CA	VAL	187	A	<-->	194	OG1	THR	296	B	0.92
34.	1148	CA	VAL	187	A	<-->	195	CG2	THR	296	B	2.97

35. 1149 C VAL 187 A <--> 189 N THR 296 B 3.57
36. 1149 C VAL 187 A <--> 190 CA THR 296 B 3.70
37. 1149 C VAL 187 A <--> 191 C THR 296 B 3.69
38. 1149 C VAL 187 A <--> 192 O THR 296 B 3.42
39. 1149 C VAL 187 A <--> 193 CB THR 296 B 3.30
40. 1149 C VAL 187 A <--> 194 OG1 THR 296 B 1.98
41. 1150 O VAL 187 A <--> 192 O THR 296 B 3.88
42. 1150 O VAL 187 A <--> 193 CB THR 296 B 3.79
43. 1150 O VAL 187 A <--> 194 OG1 THR 296 B 2.53
44. 1151 CB VAL 187 A <--> 189 N THR 296 B 3.81
45. 1151 CB VAL 187 A <--> 190 CA THR 296 B 3.42
46. 1151 CB VAL 187 A <--> 193 CB THR 296 B 2.10
47. 1151 CB VAL 187 A <--> 194 OG1 THR 296 B 1.15
48. 1151 CB VAL 187 A <--> 195 CG2 THR 296 B 2.64
49. 1152 CG1 VAL 187 A <--> 190 CA THR 296 B 3.36
50. 1152 CG1 VAL 187 A <--> 191 C THR 296 B 3.53
51. 1152 CG1 VAL 187 A <--> 193 CB THR 296 B 2.11
52. 1152 CG1 VAL 187 A <--> 194 OG1 THR 296 B 1.73
53. 1152 CG1 VAL 187 A <--> 195 CG2 THR 296 B 3.15
54. 1152 CG1 VAL 187 A <--> 196 N ASN 297 B 3.74
55. 1153 CG2 VAL 187 A <--> 190 CA THR 296 B 3.63
56. 1153 CG2 VAL 187 A <--> 193 CB THR 296 B 2.25
57. 1153 CG2 VAL 187 A <--> 194 OG1 THR 296 B 2.14
58. 1153 CG2 VAL 187 A <--> 195 CG2 THR 296 B 1.70
59. 1154 N SER 188 A <--> 189 N THR 296 B 3.69
60. 1154 N SER 188 A <--> 192 O THR 296 B 3.50
61. 1154 N SER 188 A <--> 194 OG1 THR 296 B 3.07
62. 1156 C SER 188 A <--> 192 O THR 296 B 3.70
63. 1157 O SER 188 A <--> 36 CA PRO 274 B 3.63
64. 1157 O SER 188 A <--> 39 CB PRO 274 B 3.42
65. 1157 O SER 188 A <--> 40 CG PRO 274 B 3.43
66. 1157 O SER 188 A <--> 192 O THR 296 B 3.63
67. 1160 N GLY 189 A <--> 203 ND2 ASN 297 B 3.90
68. 1161 CA GLY 189 A <--> 31 C CYS 273 B 3.61
69. 1161 CA GLY 189 A <--> 32 O CYS 273 B 3.58
70. 1161 CA GLY 189 A <--> 35 N PRO 274 B 3.58
71. 1161 CA GLY 189 A <--> 36 CA PRO 274 B 3.69
72. 1162 C GLY 189 A <--> 31 C CYS 273 B 2.96
73. 1162 C GLY 189 A <--> 32 O CYS 273 B 2.56
74. 1162 C GLY 189 A <--> 35 N PRO 274 B 3.06
75. 1162 C GLY 189 A <--> 36 CA PRO 274 B 2.85
76. 1162 C GLY 189 A <--> 37 C PRO 274 B 3.80
77. 1163 O GLY 189 A <--> 31 C CYS 273 B 3.59
78. 1163 O GLY 189 A <--> 32 O CYS 273 B 3.15
79. 1163 O GLY 189 A <--> 35 N PRO 274 B 3.37
80. 1163 O GLY 189 A <--> 36 CA PRO 274 B 2.59
81. 1163 O GLY 189 A <--> 37 C PRO 274 B 3.38
82. 1163 O GLY 189 A <--> 39 CB PRO 274 B 3.56
83. 1163 O GLY 189 A <--> 42 N ALA 275 B 3.27
84. 1164 N GLY 190 A <--> 16 CG LEU 271 B 3.47
85. 1164 N GLY 190 A <--> 18 CD2 LEU 271 B 3.52
86. 1164 N GLY 190 A <--> 29 N CYS 273 B 3.24
87. 1164 N GLY 190 A <--> 30 CA CYS 273 B 3.41
88. 1164 N GLY 190 A <--> 31 C CYS 273 B 2.37
89. 1164 N GLY 190 A <--> 32 O CYS 273 B 1.58
90. 1164 N GLY 190 A <--> 35 N PRO 274 B 3.04
91. 1164 N GLY 190 A <--> 36 CA PRO 274 B 3.19
92. 1164 N GLY 190 A <--> 37 C PRO 274 B 3.78
93. 1165 CA GLY 190 A <--> 16 CG LEU 271 B 3.55
94. 1165 CA GLY 190 A <--> 18 CD2 LEU 271 B 3.14

95.	1165	CA	GLY	190	A	<-->	30	CA	CYS	273	B	3.85
96.	1165	CA	GLY	190	A	<-->	31	C	CYS	273	B	2.67
97.	1165	CA	GLY	190	A	<-->	32	O	CYS	273	B	1.48
98.	1165	CA	GLY	190	A	<-->	35	N	PRO	274	B	3.41
99.	1165	CA	GLY	190	A	<-->	36	CA	PRO	274	B	3.29
100.	1165	CA	GLY	190	A	<-->	37	C	PRO	274	B	3.25
101.	1165	CA	GLY	190	A	<-->	42	N	ALA	275	B	3.19
102.	1166	C	GLY	190	A	<-->	16	CG	LEU	271	B	2.62
103.	1166	C	GLY	190	A	<-->	17	CD1	LEU	271	B	2.81
104.	1166	C	GLY	190	A	<-->	18	CD2	LEU	271	B	2.33
105.	1166	C	GLY	190	A	<-->	29	N	CYS	273	B	3.77
106.	1166	C	GLY	190	A	<-->	30	CA	CYS	273	B	3.75
107.	1166	C	GLY	190	A	<-->	31	C	CYS	273	B	3.08
108.	1166	C	GLY	190	A	<-->	32	O	CYS	273	B	1.93
109.	1166	C	GLY	190	A	<-->	33	CB	CYS	273	B	3.81
110.	1167	O	GLY	190	A	<-->	15	CB	LEU	271	B	2.98
111.	1167	O	GLY	190	A	<-->	16	CG	LEU	271	B	1.51
112.	1167	O	GLY	190	A	<-->	17	CD1	LEU	271	B	2.02
113.	1167	O	GLY	190	A	<-->	18	CD2	LEU	271	B	1.23
114.	1167	O	GLY	190	A	<-->	32	O	CYS	273	B	2.90
115.	1168	N	SER	191	A	<-->	16	CG	LEU	271	B	3.55
116.	1168	N	SER	191	A	<-->	17	CD1	LEU	271	B	3.13
117.	1168	N	SER	191	A	<-->	18	CD2	LEU	271	B	3.37
118.	1168	N	SER	191	A	<-->	30	CA	CYS	273	B	3.65
119.	1168	N	SER	191	A	<-->	31	C	CYS	273	B	3.18
120.	1168	N	SER	191	A	<-->	32	O	CYS	273	B	2.32
121.	1168	N	SER	191	A	<-->	33	CB	CYS	273	B	3.21
122.	1169	CA	SER	191	A	<-->	16	CG	LEU	271	B	3.69
123.	1169	CA	SER	191	A	<-->	17	CD1	LEU	271	B	2.76
124.	1169	CA	SER	191	A	<-->	18	CD2	LEU	271	B	3.67
125.	1169	CA	SER	191	A	<-->	32	O	CYS	273	B	3.61
126.	1169	CA	SER	191	A	<-->	33	CB	CYS	273	B	3.49
127.	1170	C	SER	191	A	<-->	17	CD1	LEU	271	B	3.48
128.	1170	C	SER	191	A	<-->	33	CB	CYS	273	B	2.76
129.	1170	C	SER	191	A	<-->	34	SG	CYS	273	B	3.42
130.	1171	O	SER	191	A	<-->	30	CA	CYS	273	B	3.21
131.	1171	O	SER	191	A	<-->	31	C	CYS	273	B	3.67
132.	1171	O	SER	191	A	<-->	32	O	CYS	273	B	3.71
133.	1171	O	SER	191	A	<-->	33	CB	CYS	273	B	1.79
134.	1171	O	SER	191	A	<-->	34	SG	CYS	273	B	2.20
135.	1171	O	SER	191	A	<-->	70	SG	CYS	279	B	3.83
136.	1174	N	LEU	192	A	<-->	33	CB	CYS	273	B	3.77
137.	1179	CG	LEU	192	A	<-->	68	O	CYS	279	B	3.87
138.	1181	CD2	LEU	192	A	<-->	68	O	CYS	279	B	3.76
139.	1182	N	LEU	193	A	<-->	68	O	CYS	279	B	3.37
140.	1185	O	LEU	193	A	<-->	68	O	CYS	279	B	3.40
141.	1185	O	LEU	193	A	<-->	76	OG	SER	280	B	3.52
142.	1187	CG	LEU	193	A	<-->	62	CB	THR	278	B	3.90
143.	1187	CG	LEU	193	A	<-->	64	CG2	THR	278	B	3.81
144.	1188	CD1	LEU	193	A	<-->	59	CA	THR	278	B	3.70
145.	1188	CD1	LEU	193	A	<-->	62	CB	THR	278	B	2.90
146.	1188	CD1	LEU	193	A	<-->	64	CG2	THR	278	B	2.95
147.	1189	CD2	LEU	193	A	<-->	64	CG2	THR	278	B	3.74
148.	1194	CB	MET	194	A	<-->	76	OG	SER	280	B	3.80
149.	1197	CE	MET	194	A	<-->	67	C	CYS	279	B	3.57
150.	1197	CE	MET	194	A	<-->	68	O	CYS	279	B	2.84
151.	1197	CE	MET	194	A	<-->	71	N	SER	280	B	3.56
152.	1197	CE	MET	194	A	<-->	72	CA	SER	280	B	2.68
153.	1197	CE	MET	194	A	<-->	73	C	SER	280	B	3.43
154.	1197	CE	MET	194	A	<-->	75	CB	SER	280	B	3.47

155.	1197	CE	MET	194	A	<-->	77	N	ASN	281	B	3.19
156.	1209	CD	ARG	196	A	<-->	81	CB	ASN	281	B	3.37
157.	1210	NE	ARG	196	A	<-->	81	CB	ASN	281	B	3.49
158.	1246	O	ASP	201	A	<-->	216	CB	PRO	299	B	3.29
159.	1246	O	ASP	201	A	<-->	217	CG	PRO	299	B	3.40
160.	1250	OD2	ASP	201	A	<-->	68	O	CYS	279	B	3.82
161.	1252	CA	GLU	202	A	<-->	23	CB	HIS	272	B	3.75
162.	1252	CA	GLU	202	A	<-->	25	ND1	HIS	272	B	3.41
163.	1253	C	GLU	202	A	<-->	23	CB	HIS	272	B	3.61
164.	1253	C	GLU	202	A	<-->	24	CG	HIS	272	B	3.75
165.	1253	C	GLU	202	A	<-->	25	ND1	HIS	272	B	3.25
166.	1253	C	GLU	202	A	<-->	213	CA	PRO	299	B	3.70
167.	1253	C	GLU	202	A	<-->	216	CB	PRO	299	B	3.62
168.	1254	O	GLU	202	A	<-->	23	CB	HIS	272	B	2.75
169.	1254	O	GLU	202	A	<-->	24	CG	HIS	272	B	2.92
170.	1254	O	GLU	202	A	<-->	25	ND1	HIS	272	B	2.81
171.	1254	O	GLU	202	A	<-->	27	CE1	HIS	272	B	3.85
172.	1254	O	GLU	202	A	<-->	212	N	PRO	299	B	3.73
173.	1254	O	GLU	202	A	<-->	213	CA	PRO	299	B	2.89
174.	1254	O	GLU	202	A	<-->	216	CB	PRO	299	B	3.25
175.	1254	O	GLU	202	A	<-->	217	CG	PRO	299	B	3.76
176.	1255	CB	GLU	202	A	<-->	8	O	SER	270	B	2.84
177.	1255	CB	GLU	202	A	<-->	24	CG	HIS	272	B	3.78
178.	1255	CB	GLU	202	A	<-->	25	ND1	HIS	272	B	2.78
179.	1255	CB	GLU	202	A	<-->	27	CE1	HIS	272	B	3.66
180.	1256	CG	GLU	202	A	<-->	2	CA	GLY	269	B	3.83
181.	1256	CG	GLU	202	A	<-->	8	O	SER	270	B	3.54
182.	1256	CG	GLU	202	A	<-->	25	ND1	HIS	272	B	3.44
183.	1256	CG	GLU	202	A	<-->	27	CE1	HIS	272	B	3.89
184.	1257	CD	GLU	202	A	<-->	1	N	GLY	269	B	3.67
185.	1257	CD	GLU	202	A	<-->	2	CA	GLY	269	B	2.51
186.	1257	CD	GLU	202	A	<-->	3	C	GLY	269	B	2.99
187.	1257	CD	GLU	202	A	<-->	5	N	SER	270	B	2.98
188.	1257	CD	GLU	202	A	<-->	8	O	SER	270	B	3.01
189.	1257	CD	GLU	202	A	<-->	25	ND1	HIS	272	B	3.51
190.	1257	CD	GLU	202	A	<-->	27	CE1	HIS	272	B	3.48
191.	1258	OE1	GLU	202	A	<-->	2	CA	GLY	269	B	2.87
192.	1258	OE1	GLU	202	A	<-->	3	C	GLY	269	B	2.75
193.	1258	OE1	GLU	202	A	<-->	4	O	GLY	269	B	3.81
194.	1258	OE1	GLU	202	A	<-->	5	N	SER	270	B	2.20
195.	1258	OE1	GLU	202	A	<-->	6	CA	SER	270	B	3.08
196.	1258	OE1	GLU	202	A	<-->	7	C	SER	270	B	2.90
197.	1258	OE1	GLU	202	A	<-->	8	O	SER	270	B	2.10
198.	1258	OE1	GLU	202	A	<-->	9	CB	SER	270	B	3.87
199.	1258	OE1	GLU	202	A	<-->	10	OG	SER	270	B	3.50
200.	1258	OE1	GLU	202	A	<-->	25	ND1	HIS	272	B	2.82
201.	1258	OE1	GLU	202	A	<-->	27	CE1	HIS	272	B	2.61
202.	1258	OE1	GLU	202	A	<-->	28	NE2	HIS	272	B	3.85
203.	1259	OE2	GLU	202	A	<-->	1	N	GLY	269	B	2.72
204.	1259	OE2	GLU	202	A	<-->	2	CA	GLY	269	B	1.71
205.	1259	OE2	GLU	202	A	<-->	3	C	GLY	269	B	2.79
206.	1259	OE2	GLU	202	A	<-->	4	O	GLY	269	B	3.79
207.	1259	OE2	GLU	202	A	<-->	5	N	SER	270	B	3.18
208.	1261	CA	GLY	203	A	<-->	219	N	GLU	300	B	3.49
209.	1261	CA	GLY	203	A	<-->	224	CG	GLU	300	B	3.59
210.	1261	CA	GLY	203	A	<-->	233	OG1	THR	301	B	3.90
211.	1262	C	GLY	203	A	<-->	224	CG	GLU	300	B	3.71
212.	1262	C	GLY	203	A	<-->	227	OE2	GLU	300	B	3.88
213.	1263	O	GLY	203	A	<-->	224	CG	GLU	300	B	3.51
214.	1263	O	GLY	203	A	<-->	225	CD	GLU	300	B	3.66

215.	1263	O	GLY	203	A	<-->	227	OE2	GLU	300	B	3.03
216.	1264	N	THR	204	A	<-->	233	OG1	THR	301	B	3.74
217.	1264	N	THR	204	A	<-->	234	CG2	THR	301	B	3.69
218.	1265	CA	THR	204	A	<-->	234	CG2	THR	301	B	3.86
219.	1266	C	THR	204	A	<-->	234	CG2	THR	301	B	3.44
220.	1267	O	THR	204	A	<-->	232	CB	THR	301	B	3.82
221.	1267	O	THR	204	A	<-->	233	OG1	THR	301	B	3.24
222.	1267	O	THR	204	A	<-->	234	CG2	THR	301	B	3.46
223.	1271	N	TYR	205	A	<-->	234	CG2	THR	301	B	3.75
224.	1277	CD1	TYR	205	A	<-->	427	NZ	LYS	325	B	3.89
225.	1278	CD2	TYR	205	A	<-->	427	NZ	LYS	325	B	3.25
226.	1279	CE1	TYR	205	A	<-->	234	CG2	THR	301	B	3.87
227.	1279	CE1	TYR	205	A	<-->	427	NZ	LYS	325	B	2.89
228.	1280	CE2	TYR	205	A	<-->	426	CE	LYS	325	B	3.18
229.	1280	CE2	TYR	205	A	<-->	427	NZ	LYS	325	B	1.95
230.	1281	CZ	TYR	205	A	<-->	426	CE	LYS	325	B	3.11
231.	1281	CZ	TYR	205	A	<-->	427	NZ	LYS	325	B	1.63
232.	1282	OH	TYR	205	A	<-->	425	CD	LYS	325	B	3.38
233.	1282	OH	TYR	205	A	<-->	426	CE	LYS	325	B	2.31
234.	1282	OH	TYR	205	A	<-->	427	NZ	LYS	325	B	1.25
235.	1298	CA	VAL	208	A	<-->	83	OD1	ASN	281	B	3.04
236.	1298	CA	VAL	208	A	<-->	92	ND2	ASN	282	B	3.38
237.	1299	C	VAL	208	A	<-->	78	CA	ASN	281	B	3.77
238.	1299	C	VAL	208	A	<-->	81	CB	ASN	281	B	3.87
239.	1299	C	VAL	208	A	<-->	82	CG	ASN	281	B	3.10
240.	1299	C	VAL	208	A	<-->	83	OD1	ASN	281	B	1.92
241.	1300	O	VAL	208	A	<-->	82	CG	ASN	281	B	3.76
242.	1300	O	VAL	208	A	<-->	83	OD1	ASN	281	B	2.60
243.	1301	CB	VAL	208	A	<-->	83	OD1	ASN	281	B	3.65
244.	1301	CB	VAL	208	A	<-->	90	CG	ASN	282	B	3.82
245.	1301	CB	VAL	208	A	<-->	92	ND2	ASN	282	B	2.62
246.	1302	CG1	VAL	208	A	<-->	90	CG	ASN	282	B	3.16
247.	1302	CG1	VAL	208	A	<-->	91	OD1	ASN	282	B	3.74
248.	1302	CG1	VAL	208	A	<-->	92	ND2	ASN	282	B	1.93
249.	1304	N	ALA	209	A	<-->	78	CA	ASN	281	B	2.68
250.	1304	N	ALA	209	A	<-->	79	C	ASN	281	B	3.38
251.	1304	N	ALA	209	A	<-->	80	O	ASN	281	B	3.65
252.	1304	N	ALA	209	A	<-->	81	CB	ASN	281	B	2.54
253.	1304	N	ALA	209	A	<-->	82	CG	ASN	281	B	1.84
254.	1304	N	ALA	209	A	<-->	83	OD1	ASN	281	B	0.88
255.	1304	N	ALA	209	A	<-->	84	ND2	ASN	281	B	3.04
256.	1305	CA	ALA	209	A	<-->	78	CA	ASN	281	B	3.20
257.	1305	CA	ALA	209	A	<-->	81	CB	ASN	281	B	2.29
258.	1305	CA	ALA	209	A	<-->	82	CG	ASN	281	B	1.24
259.	1305	CA	ALA	209	A	<-->	83	OD1	ASN	281	B	1.07
260.	1305	CA	ALA	209	A	<-->	84	ND2	ASN	281	B	2.09
261.	1306	C	ALA	209	A	<-->	78	CA	ASN	281	B	2.75
262.	1306	C	ALA	209	A	<-->	79	C	ASN	281	B	3.25
263.	1306	C	ALA	209	A	<-->	80	O	ASN	281	B	2.94
264.	1306	C	ALA	209	A	<-->	81	CB	ASN	281	B	1.51
265.	1306	C	ALA	209	A	<-->	82	CG	ASN	281	B	0.48
266.	1306	C	ALA	209	A	<-->	83	OD1	ASN	281	B	1.69
267.	1306	C	ALA	209	A	<-->	84	ND2	ASN	281	B	1.02
268.	1307	O	ALA	209	A	<-->	73	C	SER	280	B	3.72
269.	1307	O	ALA	209	A	<-->	74	O	SER	280	B	3.47
270.	1307	O	ALA	209	A	<-->	77	N	ASN	281	B	3.22
271.	1307	O	ALA	209	A	<-->	78	CA	ASN	281	B	1.95
272.	1307	O	ALA	209	A	<-->	79	C	ASN	281	B	2.07
273.	1307	O	ALA	209	A	<-->	80	O	ASN	281	B	1.75
274.	1307	O	ALA	209	A	<-->	81	CB	ASN	281	B	1.30

275.	1307	O	ALA	209	A	<-->	82	CG	ASN	281	B	1.18
276.	1307	O	ALA	209	A	<-->	83	OD1	ASN	281	B	2.04
277.	1307	O	ALA	209	A	<-->	84	ND2	ASN	281	B	1.86
278.	1307	O	ALA	209	A	<-->	85	N	ASN	282	B	3.39
279.	1308	CB	ALA	209	A	<-->	78	CA	ASN	281	B	3.69
280.	1308	CB	ALA	209	A	<-->	81	CB	ASN	281	B	2.68
281.	1308	CB	ALA	209	A	<-->	82	CG	ASN	281	B	2.40
282.	1308	CB	ALA	209	A	<-->	83	OD1	ASN	281	B	2.52
283.	1308	CB	ALA	209	A	<-->	84	ND2	ASN	281	B	3.09
284.	1309	N	THR	210	A	<-->	78	CA	ASN	281	B	3.86
285.	1309	N	THR	210	A	<-->	80	O	ASN	281	B	3.73
286.	1309	N	THR	210	A	<-->	81	CB	ASN	281	B	2.42
287.	1309	N	THR	210	A	<-->	82	CG	ASN	281	B	1.81
288.	1309	N	THR	210	A	<-->	83	OD1	ASN	281	B	2.89
289.	1309	N	THR	210	A	<-->	84	ND2	ASN	281	B	0.91
290.	1310	CA	THR	210	A	<-->	80	O	ASN	281	B	3.58
291.	1310	CA	THR	210	A	<-->	81	CB	ASN	281	B	3.00
292.	1310	CA	THR	210	A	<-->	82	CG	ASN	281	B	2.82
293.	1310	CA	THR	210	A	<-->	84	ND2	ASN	281	B	1.90
294.	1310	CA	THR	210	A	<-->	100	CD1	ILE	283	B	3.42
295.	1311	C	THR	210	A	<-->	81	CB	ASN	281	B	3.46
296.	1311	C	THR	210	A	<-->	82	CG	ASN	281	B	3.81
297.	1311	C	THR	210	A	<-->	84	ND2	ASN	281	B	3.15
298.	1311	C	THR	210	A	<-->	100	CD1	ILE	283	B	3.51
299.	1312	O	THR	210	A	<-->	84	ND2	ASN	281	B	3.73
300.	1313	CB	THR	210	A	<-->	84	ND2	ASN	281	B	2.71
301.	1313	CB	THR	210	A	<-->	100	CD1	ILE	283	B	3.41
302.	1314	OG1	THR	210	A	<-->	98	CG1	ILE	283	B	3.71
303.	1314	OG1	THR	210	A	<-->	100	CD1	ILE	283	B	2.36
304.	1315	CG2	THR	210	A	<-->	84	ND2	ASN	281	B	3.48
305.	1316	N	ASN	211	A	<-->	74	O	SER	280	B	3.32
306.	1316	N	ASN	211	A	<-->	81	CB	ASN	281	B	3.72
307.	1316	N	ASN	211	A	<-->	100	CD1	ILE	283	B	2.95
308.	1317	CA	ASN	211	A	<-->	74	O	SER	280	B	3.90
309.	1317	CA	ASN	211	A	<-->	75	CB	SER	280	B	3.83
310.	1317	CA	ASN	211	A	<-->	100	CD1	ILE	283	B	3.87
311.	1318	C	ASN	211	A	<-->	97	CB	ILE	283	B	3.84
312.	1318	C	ASN	211	A	<-->	99	CG2	ILE	283	B	3.80
313.	1318	C	ASN	211	A	<-->	100	CD1	ILE	283	B	3.55
314.	1319	O	ASN	211	A	<-->	74	O	SER	280	B	3.76
315.	1319	O	ASN	211	A	<-->	97	CB	ILE	283	B	2.67
316.	1319	O	ASN	211	A	<-->	98	CG1	ILE	283	B	3.13
317.	1319	O	ASN	211	A	<-->	99	CG2	ILE	283	B	2.65
318.	1319	O	ASN	211	A	<-->	100	CD1	ILE	283	B	2.67
319.	1320	CB	ASN	211	A	<-->	72	CA	SER	280	B	3.86
320.	1320	CB	ASN	211	A	<-->	73	C	SER	280	B	3.86
321.	1320	CB	ASN	211	A	<-->	74	O	SER	280	B	3.65
322.	1320	CB	ASN	211	A	<-->	75	CB	SER	280	B	2.62
323.	1320	CB	ASN	211	A	<-->	76	OG	SER	280	B	3.39
324.	1321	CG	ASN	211	A	<-->	75	CB	SER	280	B	3.39
325.	1321	CG	ASN	211	A	<-->	76	OG	SER	280	B	3.66
326.	1322	OD1	ASN	211	A	<-->	75	CB	SER	280	B	3.32
327.	1322	OD1	ASN	211	A	<-->	76	OG	SER	280	B	3.29
328.	1330	N	ALA	213	A	<-->	112	CB	ASP	285	B	3.45
329.	1330	N	ALA	213	A	<-->	113	CG	ASP	285	B	3.49
330.	1330	N	ALA	213	A	<-->	115	OD2	ASP	285	B	2.73
331.	1331	CA	ALA	213	A	<-->	63	OG1	THR	278	B	3.39
332.	1331	CA	ALA	213	A	<-->	112	CB	ASP	285	B	3.43
333.	1331	CA	ALA	213	A	<-->	113	CG	ASP	285	B	3.33
334.	1331	CA	ALA	213	A	<-->	115	OD2	ASP	285	B	2.50

335.	1332	C	ALA	213	A	<-->	63	OG1	THR	278	B	3.16
336.	1332	C	ALA	213	A	<-->	109	CA	ASP	285	B	3.85
337.	1332	C	ALA	213	A	<-->	112	CB	ASP	285	B	2.38
338.	1332	C	ALA	213	A	<-->	113	CG	ASP	285	B	2.06
339.	1332	C	ALA	213	A	<-->	114	OD1	ASP	285	B	3.16
340.	1332	C	ALA	213	A	<-->	115	OD2	ASP	285	B	1.53
341.	1333	O	ALA	213	A	<-->	109	CA	ASP	285	B	3.30
342.	1333	O	ALA	213	A	<-->	110	C	ASP	285	B	3.89
343.	1333	O	ALA	213	A	<-->	112	CB	ASP	285	B	1.92
344.	1333	O	ALA	213	A	<-->	113	CG	ASP	285	B	1.01
345.	1333	O	ALA	213	A	<-->	114	OD1	ASP	285	B	2.14
346.	1333	O	ALA	213	A	<-->	115	OD2	ASP	285	B	0.55
347.	1333	O	ALA	213	A	<-->	129	NE	ARG	287	B	3.64
348.	1333	O	ALA	213	A	<-->	132	NH2	ARG	287	B	3.49
349.	1334	CB	ALA	213	A	<-->	62	CB	THR	278	B	3.54
350.	1334	CB	ALA	213	A	<-->	63	OG1	THR	278	B	2.43
351.	1334	CB	ALA	213	A	<-->	115	OD2	ASP	285	B	3.82
352.	1335	N	GLY	214	A	<-->	62	CB	THR	278	B	3.62
353.	1335	N	GLY	214	A	<-->	63	OG1	THR	278	B	2.51
354.	1335	N	GLY	214	A	<-->	64	CG2	THR	278	B	3.75
355.	1335	N	GLY	214	A	<-->	111	O	ASP	285	B	3.59
356.	1335	N	GLY	214	A	<-->	112	CB	ASP	285	B	2.84
357.	1335	N	GLY	214	A	<-->	113	CG	ASP	285	B	2.73
358.	1335	N	GLY	214	A	<-->	114	OD1	ASP	285	B	3.56
359.	1335	N	GLY	214	A	<-->	115	OD2	ASP	285	B	2.60
360.	1336	CA	GLY	214	A	<-->	58	N	THR	278	B	3.76
361.	1336	CA	GLY	214	A	<-->	63	OG1	THR	278	B	3.23
362.	1336	CA	GLY	214	A	<-->	109	CA	ASP	285	B	3.67
363.	1336	CA	GLY	214	A	<-->	110	C	ASP	285	B	3.20
364.	1336	CA	GLY	214	A	<-->	111	O	ASP	285	B	2.59
365.	1336	CA	GLY	214	A	<-->	112	CB	ASP	285	B	2.94
366.	1336	CA	GLY	214	A	<-->	113	CG	ASP	285	B	2.73
367.	1336	CA	GLY	214	A	<-->	114	OD1	ASP	285	B	3.08
368.	1336	CA	GLY	214	A	<-->	115	OD2	ASP	285	B	3.08
369.	1337	C	GLY	214	A	<-->	111	O	ASP	285	B	3.71
370.	1337	C	GLY	214	A	<-->	113	CG	ASP	285	B	3.69
371.	1337	C	GLY	214	A	<-->	114	OD1	ASP	285	B	3.77
372.	1337	C	GLY	214	A	<-->	115	OD2	ASP	285	B	3.78
373.	1338	O	GLY	214	A	<-->	111	O	ASP	285	B	3.86
374.	1338	O	GLY	214	A	<-->	125	O	ARG	287	B	3.78
375.	1338	O	GLY	214	A	<-->	144	CE	LYS	289	B	3.29
376.	1338	O	GLY	214	A	<-->	145	NZ	LYS	289	B	2.87
377.	1339	N	HIS	215	A	<-->	115	OD2	ASP	285	B	3.79
378.	1340	CA	HIS	215	A	<-->	145	NZ	LYS	289	B	3.71
379.	1341	C	HIS	215	A	<-->	145	NZ	LYS	289	B	3.76
380.	1349	N	ARG	216	A	<-->	145	NZ	LYS	289	B	3.26
381.	1353	CB	ARG	216	A	<-->	144	CE	LYS	289	B	3.65
382.	1353	CB	ARG	216	A	<-->	145	NZ	LYS	289	B	3.86
383.	1354	CG	ARG	216	A	<-->	144	CE	LYS	289	B	3.75
384.	1355	CD	ARG	216	A	<-->	45	O	ALA	275	B	3.85
385.	1355	CD	ARG	216	A	<-->	50	O	ALA	276	B	3.63
386.	1355	CD	ARG	216	A	<-->	144	CE	LYS	289	B	3.84
387.	1356	NE	ARG	216	A	<-->	45	O	ALA	275	B	3.78
388.	1356	NE	ARG	216	A	<-->	48	CA	ALA	276	B	3.55
389.	1356	NE	ARG	216	A	<-->	49	C	ALA	276	B	3.13
390.	1356	NE	ARG	216	A	<-->	50	O	ALA	276	B	2.44
391.	1356	NE	ARG	216	A	<-->	143	CD	LYS	289	B	3.56
392.	1356	NE	ARG	216	A	<-->	144	CE	LYS	289	B	2.73
393.	1357	CZ	ARG	216	A	<-->	44	C	ALA	275	B	3.47
394.	1357	CZ	ARG	216	A	<-->	45	O	ALA	275	B	2.87

395.	1357	CZ	ARG	216	A	<-->	47	N	ALA	276	B	3.28
396.	1357	CZ	ARG	216	A	<-->	48	CA	ALA	276	B	2.23
397.	1357	CZ	ARG	216	A	<-->	49	C	ALA	276	B	1.87
398.	1357	CZ	ARG	216	A	<-->	50	O	ALA	276	B	1.59
399.	1357	CZ	ARG	216	A	<-->	51	CB	ALA	276	B	3.36
400.	1357	CZ	ARG	216	A	<-->	52	N	CYS	277	B	2.99
401.	1357	CZ	ARG	216	A	<-->	53	CA	CYS	277	B	3.80
402.	1357	CZ	ARG	216	A	<-->	143	CD	LYS	289	B	3.85
403.	1357	CZ	ARG	216	A	<-->	144	CE	LYS	289	B	3.43
404.	1358	NH1	ARG	216	A	<-->	43	CA	ALA	275	B	3.82
405.	1358	NH1	ARG	216	A	<-->	44	C	ALA	275	B	2.39
406.	1358	NH1	ARG	216	A	<-->	45	O	ALA	275	B	1.63
407.	1358	NH1	ARG	216	A	<-->	47	N	ALA	276	B	2.63
408.	1358	NH1	ARG	216	A	<-->	48	CA	ALA	276	B	2.23
409.	1358	NH1	ARG	216	A	<-->	49	C	ALA	276	B	1.97
410.	1358	NH1	ARG	216	A	<-->	50	O	ALA	276	B	2.36
411.	1358	NH1	ARG	216	A	<-->	51	CB	ALA	276	B	3.71
412.	1358	NH1	ARG	216	A	<-->	52	N	CYS	277	B	2.58
413.	1358	NH1	ARG	216	A	<-->	53	CA	CYS	277	B	3.52
414.	1358	NH1	ARG	216	A	<-->	54	C	CYS	277	B	3.64
415.	1358	NH1	ARG	216	A	<-->	55	O	CYS	277	B	3.16
416.	1359	NH2	ARG	216	A	<-->	44	C	ALA	275	B	3.82
417.	1359	NH2	ARG	216	A	<-->	45	O	ALA	275	B	3.64
418.	1359	NH2	ARG	216	A	<-->	47	N	ALA	276	B	3.13
419.	1359	NH2	ARG	216	A	<-->	48	CA	ALA	276	B	1.71
420.	1359	NH2	ARG	216	A	<-->	49	C	ALA	276	B	1.55
421.	1359	NH2	ARG	216	A	<-->	50	O	ALA	276	B	1.28
422.	1359	NH2	ARG	216	A	<-->	51	CB	ALA	276	B	2.26
423.	1359	NH2	ARG	216	A	<-->	52	N	CYS	277	B	2.87
424.	1359	NH2	ARG	216	A	<-->	53	CA	CYS	277	B	3.79
425.	1359	NH2	ARG	216	A	<-->	142	CG	LYS	289	B	3.35
426.	1359	NH2	ARG	216	A	<-->	143	CD	LYS	289	B	3.22
427.	1359	NH2	ARG	216	A	<-->	144	CE	LYS	289	B	3.32
428.	1377	C	ARG	219	A	<-->	136	O	GLY	288	B	3.75
429.	1386	N	ALA	220	A	<-->	136	O	GLY	288	B	3.10
430.	1387	CA	ALA	220	A	<-->	136	O	GLY	288	B	3.52
431.	1387	CA	ALA	220	A	<-->	138	CA	LYS	289	B	3.81
432.	1387	CA	ALA	220	A	<-->	139	C	LYS	289	B	3.74
433.	1388	C	ALA	220	A	<-->	136	O	GLY	288	B	3.55
434.	1388	C	ALA	220	A	<-->	137	N	LYS	289	B	3.75
435.	1388	C	ALA	220	A	<-->	138	CA	LYS	289	B	2.70
436.	1388	C	ALA	220	A	<-->	139	C	LYS	289	B	2.41
437.	1388	C	ALA	220	A	<-->	140	O	LYS	289	B	2.44
438.	1388	C	ALA	220	A	<-->	141	CB	LYS	289	B	3.78
439.	1388	C	ALA	220	A	<-->	146	N	GLY	290	B	3.07
440.	1388	C	ALA	220	A	<-->	147	CA	GLY	290	B	3.75
441.	1389	O	ALA	220	A	<-->	136	O	GLY	288	B	3.58
442.	1389	O	ALA	220	A	<-->	137	N	LYS	289	B	3.59
443.	1389	O	ALA	220	A	<-->	138	CA	LYS	289	B	2.31
444.	1389	O	ALA	220	A	<-->	139	C	LYS	289	B	2.48
445.	1389	O	ALA	220	A	<-->	140	O	LYS	289	B	2.38
446.	1389	O	ALA	220	A	<-->	141	CB	LYS	289	B	2.95
447.	1389	O	ALA	220	A	<-->	142	CG	LYS	289	B	3.56
448.	1389	O	ALA	220	A	<-->	143	CD	LYS	289	B	2.98
449.	1389	O	ALA	220	A	<-->	146	N	GLY	290	B	3.55
450.	1391	N	ALA	221	A	<-->	138	CA	LYS	289	B	2.95
451.	1391	N	ALA	221	A	<-->	139	C	LYS	289	B	1.95
452.	1391	N	ALA	221	A	<-->	140	O	LYS	289	B	1.90
453.	1391	N	ALA	221	A	<-->	146	N	GLY	290	B	2.22
454.	1391	N	ALA	221	A	<-->	147	CA	GLY	290	B	2.51

455.	1391	N	ALA	221	A	<-->	148	C	GLY	290	B	3.55
456.	1392	CA	ALA	221	A	<-->	138	CA	LYS	289	B	2.92
457.	1392	CA	ALA	221	A	<-->	139	C	LYS	289	B	1.49
458.	1392	CA	ALA	221	A	<-->	140	O	LYS	289	B	0.93
459.	1392	CA	ALA	221	A	<-->	141	CB	LYS	289	B	3.69
460.	1392	CA	ALA	221	A	<-->	146	N	GLY	290	B	1.93
461.	1392	CA	ALA	221	A	<-->	147	CA	GLY	290	B	2.01
462.	1392	CA	ALA	221	A	<-->	148	C	GLY	290	B	2.46
463.	1392	CA	ALA	221	A	<-->	149	O	GLY	290	B	3.03
464.	1392	CA	ALA	221	A	<-->	150	N	LEU	291	B	3.09
465.	1393	C	ALA	221	A	<-->	139	C	LYS	289	B	2.82
466.	1393	C	ALA	221	A	<-->	140	O	LYS	289	B	1.70
467.	1393	C	ALA	221	A	<-->	146	N	GLY	290	B	3.41
468.	1393	C	ALA	221	A	<-->	147	CA	GLY	290	B	3.18
469.	1393	C	ALA	221	A	<-->	148	C	GLY	290	B	3.14
470.	1393	C	ALA	221	A	<-->	149	O	GLY	290	B	3.16
471.	1393	C	ALA	221	A	<-->	150	N	LEU	291	B	3.83
472.	1394	O	ALA	221	A	<-->	139	C	LYS	289	B	3.67
473.	1394	O	ALA	221	A	<-->	140	O	LYS	289	B	2.64
474.	1395	CB	ALA	221	A	<-->	138	CA	LYS	289	B	3.82
475.	1395	CB	ALA	221	A	<-->	139	C	LYS	289	B	2.32
476.	1395	CB	ALA	221	A	<-->	140	O	LYS	289	B	2.27
477.	1395	CB	ALA	221	A	<-->	146	N	GLY	290	B	1.82
478.	1395	CB	ALA	221	A	<-->	147	CA	GLY	290	B	0.90
479.	1395	CB	ALA	221	A	<-->	148	C	GLY	290	B	1.07
480.	1395	CB	ALA	221	A	<-->	149	O	GLY	290	B	1.81
481.	1395	CB	ALA	221	A	<-->	150	N	LEU	291	B	2.11
482.	1395	CB	ALA	221	A	<-->	151	CA	LEU	291	B	3.35
483.	1396	N	ARG	222	A	<-->	139	C	LYS	289	B	3.48
484.	1396	N	ARG	222	A	<-->	140	O	LYS	289	B	2.35
485.	1396	N	ARG	222	A	<-->	147	CA	GLY	290	B	3.86
486.	1396	N	ARG	222	A	<-->	148	C	GLY	290	B	3.26
487.	1396	N	ARG	222	A	<-->	149	O	GLY	290	B	3.11
488.	1396	N	ARG	222	A	<-->	150	N	LEU	291	B	3.63
489.	1396	N	ARG	222	A	<-->	157	CD2	LEU	291	B	3.30
490.	1397	CA	ARG	222	A	<-->	140	O	LYS	289	B	3.70
491.	1397	CA	ARG	222	A	<-->	157	CD2	LEU	291	B	3.79
492.	1399	O	ARG	222	A	<-->	140	O	LYS	289	B	3.62
493.	1412	CG1	VAL	223	A	<-->	47	N	ALA	276	B	3.43
494.	1412	CG1	VAL	223	A	<-->	48	CA	ALA	276	B	3.67
495.	1412	CG1	VAL	223	A	<-->	51	CB	ALA	276	B	3.31

Salt bridges

	<----- A T O M 1 ----->						<----- A T O M 2 ----->					
	Atom no.	Atom name	Res name	Res no.	Chain		Atom no.	Atom name	Res name	Res no.	Chain	Distance
1.	1258	OE1	GLU	202	A	<-->	25	ND1	HIS	272	B	2.82

Number of salt bridges: 1

Number of hydrogen bonds: 9

Number of non-bonded contacts: 495

[C] R119Q + SLIT2.D2 (WT)

Hydrogen bonds

<----- A T O M 1 -----> <----- A T O M 2 ----->

	Atom no.	Atom name	Res name	Res no.	Chain		Atom no.	Atom name	Res name	Res no.	Chain	Distance
1.	4	OG	SER	60	A	<-->	63	OG1	THR	278	B	2.30
2.	4	OG	SER	60	A	<-->	63	OG1	THR	278	B	2.30
3.	41	OE2	GLU	64	A	<-->	456	NH1	ARG	328	B	3.26
4.	301	N	ILE	99	A	<-->	1058	OH	TYR	404	B	1.72
5.	308	O	ILE	99	A	<-->	1058	OH	TYR	404	B	2.65
6.	308	O	ILE	99	A	<-->	1252	NE2	GLN	429	B	3.21
7.	411	O	ASP	111	A	<-->	1482	OG1	THR	458	B	3.21

Non-bonded contacts

<----- A T O M 1 -----> <----- A T O M 2 ----->

	Atom no.	Atom name	Res name	Res no.	Chain		Atom no.	Atom name	Res name	Res no.	Chain	Distance
1.	1	N	SER	60	A	<-->	75	CB	SER	280	B	3.55
2.	1	N	SER	60	A	<-->	76	OG	SER	280	B	3.13
3.	2	CA	SER	60	A	<-->	63	OG1	THR	278	B	3.55
4.	2	CA	SER	60	A	<-->	76	OG	SER	280	B	3.58
5.	5	C	SER	60	A	<-->	115	OD2	ASP	285	B	3.30
6.	6	O	SER	60	A	<-->	115	OD2	ASP	285	B	3.45
7.	3	CB	SER	60	A	<-->	61	O	THR	278	B	3.89
8.	3	CB	SER	60	A	<-->	63	OG1	THR	278	B	2.98
9.	3	CB	SER	60	A	<-->	108	N	ASP	285	B	3.90
10.	3	CB	SER	60	A	<-->	109	CA	ASP	285	B	3.86
11.	3	CB	SER	60	A	<-->	112	CB	ASP	285	B	2.64
12.	3	CB	SER	60	A	<-->	113	CG	ASP	285	B	3.51
13.	3	CB	SER	60	A	<-->	115	OD2	ASP	285	B	3.42
14.	4	OG	SER	60	A	<-->	61	O	THR	278	B	3.75
15.	4	OG	SER	60	A	<-->	62	CB	THR	278	B	3.74
16.	4	OG	SER	60	A	<-->	63	OG1	THR	278	B	2.30
17.	4	OG	SER	60	A	<-->	108	N	ASP	285	B	3.62
18.	4	OG	SER	60	A	<-->	109	CA	ASP	285	B	3.24
19.	4	OG	SER	60	A	<-->	111	O	ASP	285	B	3.85
20.	4	OG	SER	60	A	<-->	112	CB	ASP	285	B	1.86
21.	4	OG	SER	60	A	<-->	113	CG	ASP	285	B	2.56
22.	4	OG	SER	60	A	<-->	114	OD1	ASP	285	B	3.74
23.	4	OG	SER	60	A	<-->	115	OD2	ASP	285	B	2.59
24.	7	N	LEU	61	A	<-->	115	OD2	ASP	285	B	3.30
25.	8	CA	LEU	61	A	<-->	115	OD2	ASP	285	B	3.54
26.	8	CA	LEU	61	A	<-->	132	NH2	ARG	287	B	3.32
27.	13	C	LEU	61	A	<-->	115	OD2	ASP	285	B	3.78
28.	13	C	LEU	61	A	<-->	130	CZ	ARG	287	B	3.80
29.	13	C	LEU	61	A	<-->	132	NH2	ARG	287	B	3.32
30.	13	C	LEU	61	A	<-->	276	NH1	ARG	306	B	3.56
31.	14	O	LEU	61	A	<-->	113	CG	ASP	285	B	3.77
32.	14	O	LEU	61	A	<-->	115	OD2	ASP	285	B	3.21
33.	14	O	LEU	61	A	<-->	129	NE	ARG	287	B	3.07
34.	14	O	LEU	61	A	<-->	130	CZ	ARG	287	B	2.95
35.	14	O	LEU	61	A	<-->	131	NH1	ARG	287	B	3.75
36.	14	O	LEU	61	A	<-->	132	NH2	ARG	287	B	2.81

37.	14	O	LEU	61	A	<-->	275	CZ	ARG	306	B	3.48
38.	14	O	LEU	61	A	<-->	276	NH1	ARG	306	B	3.21
39.	14	O	LEU	61	A	<-->	277	NH2	ARG	306	B	3.42
40.	9	CB	LEU	61	A	<-->	115	OD2	ASP	285	B	3.08
41.	9	CB	LEU	61	A	<-->	130	CZ	ARG	287	B	3.43
42.	9	CB	LEU	61	A	<-->	132	NH2	ARG	287	B	2.22
43.	10	CG	LEU	61	A	<-->	130	CZ	ARG	287	B	2.92
44.	10	CG	LEU	61	A	<-->	131	NH1	ARG	287	B	3.32
45.	10	CG	LEU	61	A	<-->	132	NH2	ARG	287	B	1.86
46.	11	CD1	LEU	61	A	<-->	132	NH2	ARG	287	B	3.33
47.	12	CD2	LEU	61	A	<-->	130	CZ	ARG	287	B	3.20
48.	12	CD2	LEU	61	A	<-->	131	NH1	ARG	287	B	3.51
49.	12	CD2	LEU	61	A	<-->	132	NH2	ARG	287	B	2.16
50.	15	N	ARG	62	A	<-->	276	NH1	ARG	306	B	3.18
51.	16	CA	ARG	62	A	<-->	258	OE2	GLU	304	B	3.79
52.	16	CA	ARG	62	A	<-->	275	CZ	ARG	306	B	3.07
53.	16	CA	ARG	62	A	<-->	276	NH1	ARG	306	B	2.08
54.	16	CA	ARG	62	A	<-->	277	NH2	ARG	306	B	3.26
55.	24	C	ARG	62	A	<-->	275	CZ	ARG	306	B	3.25
56.	24	C	ARG	62	A	<-->	276	NH1	ARG	306	B	2.77
57.	24	C	ARG	62	A	<-->	277	NH2	ARG	306	B	2.91
58.	25	O	ARG	62	A	<-->	277	NH2	ARG	306	B	3.88
59.	17	CB	ARG	62	A	<-->	258	OE2	GLU	304	B	3.26
60.	17	CB	ARG	62	A	<-->	276	NH1	ARG	306	B	2.78
61.	17	CB	ARG	62	A	<-->	456	NH1	ARG	328	B	3.52
62.	26	N	GLN	63	A	<-->	274	NE	ARG	306	B	3.84
63.	26	N	GLN	63	A	<-->	275	CZ	ARG	306	B	2.65
64.	26	N	GLN	63	A	<-->	276	NH1	ARG	306	B	2.63
65.	26	N	GLN	63	A	<-->	277	NH2	ARG	306	B	2.15
66.	26	N	GLN	63	A	<-->	457	NH2	ARG	328	B	3.03
67.	27	CA	GLN	63	A	<-->	275	CZ	ARG	306	B	3.80
68.	27	CA	GLN	63	A	<-->	277	NH2	ARG	306	B	3.04
69.	27	CA	GLN	63	A	<-->	457	NH2	ARG	328	B	3.29
70.	33	C	GLN	63	A	<-->	455	CZ	ARG	328	B	3.80
71.	33	C	GLN	63	A	<-->	457	NH2	ARG	328	B	2.59
72.	34	O	GLN	63	A	<-->	454	NE	ARG	328	B	3.89
73.	34	O	GLN	63	A	<-->	455	CZ	ARG	328	B	3.07
74.	34	O	GLN	63	A	<-->	457	NH2	ARG	328	B	1.84
75.	28	CB	GLN	63	A	<-->	277	NH2	ARG	306	B	2.97
76.	35	N	GLU	64	A	<-->	457	NH2	ARG	328	B	3.51
77.	41	OE2	GLU	64	A	<-->	456	NH1	ARG	328	B	3.26
78.	48	OD1	ASP	65	A	<-->	831	CD2	LEU	376	B	3.64
79.	49	OD2	ASP	65	A	<-->	831	CD2	LEU	376	B	3.58
80.	267	NE	ARG	94	A	<-->	473	OD2	ASP	330	B	3.30
81.	268	CZ	ARG	94	A	<-->	471	CG	ASP	330	B	3.48
82.	268	CZ	ARG	94	A	<-->	472	OD1	ASP	330	B	3.89
83.	268	CZ	ARG	94	A	<-->	473	OD2	ASP	330	B	2.39
84.	268	CZ	ARG	94	A	<-->	653	CG1	VAL	354	B	3.63
85.	269	NH1	ARG	94	A	<-->	473	OD2	ASP	330	B	3.06
86.	269	NH1	ARG	94	A	<-->	487	OG	SER	332	B	3.80
87.	269	NH1	ARG	94	A	<-->	672	CE2	TYR	356	B	3.70
88.	269	NH1	ARG	94	A	<-->	673	CZ	TYR	356	B	3.69
89.	269	NH1	ARG	94	A	<-->	674	OH	TYR	356	B	3.07
90.	270	NH2	ARG	94	A	<-->	470	CB	ASP	330	B	3.62
91.	270	NH2	ARG	94	A	<-->	471	CG	ASP	330	B	2.34
92.	270	NH2	ARG	94	A	<-->	472	OD1	ASP	330	B	2.77
93.	270	NH2	ARG	94	A	<-->	473	OD2	ASP	330	B	1.48
94.	270	NH2	ARG	94	A	<-->	487	OG	SER	332	B	3.86
95.	270	NH2	ARG	94	A	<-->	652	CB	VAL	354	B	3.19
96.	270	NH2	ARG	94	A	<-->	653	CG1	VAL	354	B	2.88

97.	270	NH2	ARG	94	A	<-->	654	CG2	VAL	354	B	3.34
98.	273	N	PRO	95	A	<-->	846	CD1	LEU	378	B	3.81
99.	274	CA	PRO	95	A	<-->	846	CD1	LEU	378	B	3.07
100.	276	CB	PRO	95	A	<-->	845	CG	LEU	378	B	3.49
101.	276	CB	PRO	95	A	<-->	846	CD1	LEU	378	B	2.95
102.	276	CB	PRO	95	A	<-->	847	CD2	LEU	378	B	2.91
103.	277	CG	PRO	95	A	<-->	845	CG	LEU	378	B	3.59
104.	277	CG	PRO	95	A	<-->	846	CD1	LEU	378	B	3.16
105.	277	CG	PRO	95	A	<-->	847	CD2	LEU	378	B	2.93
106.	277	CG	PRO	95	A	<-->	1024	CD2	LEU	400	B	3.22
107.	280	N	THR	96	A	<-->	670	CD2	TYR	356	B	3.82
108.	280	N	THR	96	A	<-->	672	CE2	TYR	356	B	3.76
109.	280	N	THR	96	A	<-->	863	ND2	ASN	380	B	3.45
110.	281	CA	THR	96	A	<-->	863	ND2	ASN	380	B	3.24
111.	285	C	THR	96	A	<-->	863	ND2	ASN	380	B	3.67
112.	286	O	THR	96	A	<-->	863	ND2	ASN	380	B	3.30
113.	282	CB	THR	96	A	<-->	668	CG	TYR	356	B	3.88
114.	282	CB	THR	96	A	<-->	861	CG	ASN	380	B	3.47
115.	282	CB	THR	96	A	<-->	863	ND2	ASN	380	B	2.20
116.	283	OG1	THR	96	A	<-->	667	CB	TYR	356	B	3.26
117.	283	OG1	THR	96	A	<-->	668	CG	TYR	356	B	2.53
118.	283	OG1	THR	96	A	<-->	669	CD1	TYR	356	B	2.62
119.	283	OG1	THR	96	A	<-->	670	CD2	TYR	356	B	2.75
120.	283	OG1	THR	96	A	<-->	671	CE1	TYR	356	B	3.02
121.	283	OG1	THR	96	A	<-->	672	CE2	TYR	356	B	3.12
122.	283	OG1	THR	96	A	<-->	673	CZ	TYR	356	B	3.22
123.	283	OG1	THR	96	A	<-->	861	CG	ASN	380	B	3.63
124.	283	OG1	THR	96	A	<-->	863	ND2	ASN	380	B	2.54
125.	284	CG2	THR	96	A	<-->	863	ND2	ASN	380	B	3.23
126.	293	O	PRO	97	A	<-->	1056	CE2	TYR	404	B	3.54
127.	294	N	THR	98	A	<-->	1054	CD2	TYR	404	B	3.88
128.	294	N	THR	98	A	<-->	1056	CE2	TYR	404	B	3.57
129.	294	N	THR	98	A	<-->	1057	CZ	TYR	404	B	3.75
130.	295	CA	THR	98	A	<-->	1052	CG	TYR	404	B	3.65
131.	295	CA	THR	98	A	<-->	1053	CD1	TYR	404	B	3.53
132.	295	CA	THR	98	A	<-->	1054	CD2	TYR	404	B	3.20
133.	295	CA	THR	98	A	<-->	1055	CE1	TYR	404	B	2.95
134.	295	CA	THR	98	A	<-->	1056	CE2	TYR	404	B	2.54
135.	295	CA	THR	98	A	<-->	1057	CZ	TYR	404	B	2.39
136.	295	CA	THR	98	A	<-->	1058	OH	TYR	404	B	2.79
137.	299	C	THR	98	A	<-->	1055	CE1	TYR	404	B	3.57
138.	299	C	THR	98	A	<-->	1056	CE2	TYR	404	B	3.48
139.	299	C	THR	98	A	<-->	1057	CZ	TYR	404	B	2.93
140.	299	C	THR	98	A	<-->	1058	OH	TYR	404	B	2.57
141.	300	O	THR	98	A	<-->	1058	OH	TYR	404	B	3.71
142.	296	CB	THR	98	A	<-->	1052	CG	TYR	404	B	2.86
143.	296	CB	THR	98	A	<-->	1053	CD1	TYR	404	B	2.35
144.	296	CB	THR	98	A	<-->	1054	CD2	TYR	404	B	3.01
145.	296	CB	THR	98	A	<-->	1055	CE1	TYR	404	B	1.99
146.	296	CB	THR	98	A	<-->	1056	CE2	TYR	404	B	2.74
147.	296	CB	THR	98	A	<-->	1057	CZ	TYR	404	B	2.23
148.	296	CB	THR	98	A	<-->	1058	OH	TYR	404	B	3.03
149.	297	OG1	THR	98	A	<-->	1053	CD1	TYR	404	B	3.29
150.	297	OG1	THR	98	A	<-->	1055	CE1	TYR	404	B	3.05
151.	297	OG1	THR	98	A	<-->	1057	CZ	TYR	404	B	3.55
152.	297	OG1	THR	98	A	<-->	1449	CB	SER	453	B	3.18
153.	298	CG2	THR	98	A	<-->	1051	CB	TYR	404	B	2.59
154.	298	CG2	THR	98	A	<-->	1052	CG	TYR	404	B	1.68
155.	298	CG2	THR	98	A	<-->	1053	CD1	TYR	404	B	1.78
156.	298	CG2	THR	98	A	<-->	1054	CD2	TYR	404	B	2.20

157. 298 CG2 THR 98 A <--> 1055 CE1 TYR 404 B 2.38
158. 298 CG2 THR 98 A <--> 1056 CE2 TYR 404 B 2.71
159. 298 CG2 THR 98 A <--> 1057 CZ TYR 404 B 2.78
160. 301 N ILE 99 A <--> 1055 CE1 TYR 404 B 3.57
161. 301 N ILE 99 A <--> 1056 CE2 TYR 404 B 3.37
162. 301 N ILE 99 A <--> 1057 CZ TYR 404 B 2.67
163. 301 N ILE 99 A <--> 1058 OH TYR 404 B 1.72
164. 302 CA ILE 99 A <--> 1058 OH TYR 404 B 2.74
165. 307 C ILE 99 A <--> 1058 OH TYR 404 B 3.02
166. 308 O ILE 99 A <--> 1057 CZ TYR 404 B 3.90
167. 308 O ILE 99 A <--> 1058 OH TYR 404 B 2.65
168. 308 O ILE 99 A <--> 1252 NE2 GLN 429 B 3.21
169. 303 CB ILE 99 A <--> 1058 OH TYR 404 B 3.40
170. 313 CD GLU 100 A <--> 1448 O SER 453 B 3.46
171. 314 OE1 GLU 100 A <--> 1448 O SER 453 B 3.55
172. 315 OE2 GLU 100 A <--> 1448 O SER 453 B 3.52
173. 315 OE2 GLU 100 A <--> 1470 NH2 ARG 456 B 3.58
174. 380 O ARG 107 A <--> 1539 ND2 ASN 465 B 3.62
175. 372 CB ARG 107 A <--> 1470 NH2 ARG 456 B 3.66
176. 373 CG ARG 107 A <--> 1467 NE ARG 456 B 3.79
177. 373 CG ARG 107 A <--> 1468 CZ ARG 456 B 3.18
178. 373 CG ARG 107 A <--> 1469 NH1 ARG 456 B 3.54
179. 373 CG ARG 107 A <--> 1470 NH2 ARG 456 B 2.93
180. 374 CD ARG 107 A <--> 1468 CZ ARG 456 B 3.81
181. 374 CD ARG 107 A <--> 1470 NH2 ARG 456 B 3.27
182. 375 NE ARG 107 A <--> 1467 NE ARG 456 B 3.47
183. 375 NE ARG 107 A <--> 1468 CZ ARG 456 B 3.61
184. 375 NE ARG 107 A <--> 1470 NH2 ARG 456 B 3.31
185. 376 CZ ARG 107 A <--> 1466 CD ARG 456 B 3.57
186. 376 CZ ARG 107 A <--> 1467 NE ARG 456 B 2.43
187. 376 CZ ARG 107 A <--> 1468 CZ ARG 456 B 2.81
188. 376 CZ ARG 107 A <--> 1470 NH2 ARG 456 B 2.62
189. 377 NH1 ARG 107 A <--> 1466 CD ARG 456 B 3.39
190. 377 NH1 ARG 107 A <--> 1467 NE ARG 456 B 1.97
191. 377 NH1 ARG 107 A <--> 1468 CZ ARG 456 B 1.86
192. 377 NH1 ARG 107 A <--> 1469 NH1 ARG 456 B 3.15
193. 377 NH1 ARG 107 A <--> 1470 NH2 ARG 456 B 1.37
194. 378 NH2 ARG 107 A <--> 1466 CD ARG 456 B 3.46
195. 378 NH2 ARG 107 A <--> 1467 NE ARG 456 B 2.68
196. 378 NH2 ARG 107 A <--> 1468 CZ ARG 456 B 3.55
197. 378 NH2 ARG 107 A <--> 1470 NH2 ARG 456 B 3.63
198. 388 N GLU 109 A <--> 1537 CG ASN 465 B 3.80
199. 388 N GLU 109 A <--> 1538 OD1 ASN 465 B 3.71
200. 388 N GLU 109 A <--> 1539 ND2 ASN 465 B 3.31
201. 389 CA GLU 109 A <--> 1537 CG ASN 465 B 3.33
202. 389 CA GLU 109 A <--> 1538 OD1 ASN 465 B 3.17
203. 389 CA GLU 109 A <--> 1539 ND2 ASN 465 B 3.30
204. 390 CB GLU 109 A <--> 1536 CB ASN 465 B 3.52
205. 390 CB GLU 109 A <--> 1537 CG ASN 465 B 2.97
206. 390 CB GLU 109 A <--> 1538 OD1 ASN 465 B 3.34
207. 390 CB GLU 109 A <--> 1539 ND2 ASN 465 B 2.90
208. 391 CG GLU 109 A <--> 1533 CA ASN 465 B 3.77
209. 391 CG GLU 109 A <--> 1536 CB ASN 465 B 2.36
210. 391 CG GLU 109 A <--> 1537 CG ASN 465 B 1.90
211. 391 CG GLU 109 A <--> 1538 OD1 ASN 465 B 2.77
212. 391 CG GLU 109 A <--> 1539 ND2 ASN 465 B 1.66
213. 392 CD GLU 109 A <--> 1536 CB ASN 465 B 2.83
214. 392 CD GLU 109 A <--> 1537 CG ASN 465 B 3.05
215. 392 CD GLU 109 A <--> 1539 ND2 ASN 465 B 2.65
216. 393 OE1 GLU 109 A <--> 1539 ND2 ASN 465 B 3.60

217.	394	OE2	GLU	109	A	<-->	1529	C	ALA	464	B	3.79
218.	394	OE2	GLU	109	A	<-->	1530	O	ALA	464	B	3.21
219.	394	OE2	GLU	109	A	<-->	1533	CA	ASN	465	B	3.84
220.	394	OE2	GLU	109	A	<-->	1536	CB	ASN	465	B	2.50
221.	394	OE2	GLU	109	A	<-->	1537	CG	ASN	465	B	3.25
222.	394	OE2	GLU	109	A	<-->	1539	ND2	ASN	465	B	3.06
223.	411	O	ASP	111	A	<-->	1482	OG1	THR	458	B	3.21
224.	411	O	ASP	111	A	<-->	1483	CG2	THR	458	B	3.73
225.	419	C	LYS	112	A	<-->	1256	O	ASN	430	B	3.78
226.	419	C	LYS	112	A	<-->	1482	OG1	THR	458	B	3.21
227.	420	O	LYS	112	A	<-->	1253	N	ASN	430	B	3.87
228.	420	O	LYS	112	A	<-->	1255	C	ASN	430	B	3.29
229.	420	O	LYS	112	A	<-->	1256	O	ASN	430	B	2.63
230.	420	O	LYS	112	A	<-->	1261	N	PRO	431	B	3.78
231.	420	O	LYS	112	A	<-->	1262	CA	PRO	431	B	3.76
232.	420	O	LYS	112	A	<-->	1482	OG1	THR	458	B	3.23
233.	414	CB	LYS	112	A	<-->	1245	CA	GLN	429	B	3.58
234.	414	CB	LYS	112	A	<-->	1246	C	GLN	429	B	3.89
235.	414	CB	LYS	112	A	<-->	1250	CD	GLN	429	B	3.59
236.	414	CB	LYS	112	A	<-->	1251	OE1	GLN	429	B	2.69
237.	415	CG	LYS	112	A	<-->	1250	CD	GLN	429	B	3.76
238.	415	CG	LYS	112	A	<-->	1251	OE1	GLN	429	B	2.62
239.	416	CD	LYS	112	A	<-->	1247	O	GLN	429	B	3.76
240.	417	CE	LYS	112	A	<-->	1083	NZ	LYS	407	B	3.69
241.	418	NZ	LYS	112	A	<-->	1081	CD	LYS	407	B	3.20
242.	418	NZ	LYS	112	A	<-->	1082	CE	LYS	407	B	2.99
243.	418	NZ	LYS	112	A	<-->	1083	NZ	LYS	407	B	2.38
244.	421	N	ASP	113	A	<-->	1482	OG1	THR	458	B	3.26
245.	422	CA	ASP	113	A	<-->	1474	O	CYS	457	B	3.42
246.	422	CA	ASP	113	A	<-->	1478	CA	THR	458	B	3.83
247.	422	CA	ASP	113	A	<-->	1482	OG1	THR	458	B	3.22
248.	427	C	ASP	113	A	<-->	1473	C	CYS	457	B	3.43
249.	427	C	ASP	113	A	<-->	1474	O	CYS	457	B	2.91
250.	427	C	ASP	113	A	<-->	1477	N	THR	458	B	3.33
251.	427	C	ASP	113	A	<-->	1478	CA	THR	458	B	2.70
252.	427	C	ASP	113	A	<-->	1479	C	THR	458	B	3.84
253.	427	C	ASP	113	A	<-->	1481	CB	THR	458	B	3.22
254.	427	C	ASP	113	A	<-->	1482	OG1	THR	458	B	2.76
255.	427	C	ASP	113	A	<-->	1483	CG2	THR	458	B	3.85
256.	428	O	ASP	113	A	<-->	1472	CA	CYS	457	B	3.87
257.	428	O	ASP	113	A	<-->	1473	C	CYS	457	B	2.37
258.	428	O	ASP	113	A	<-->	1474	O	CYS	457	B	1.91
259.	428	O	ASP	113	A	<-->	1477	N	THR	458	B	2.32
260.	428	O	ASP	113	A	<-->	1478	CA	THR	458	B	1.84
261.	428	O	ASP	113	A	<-->	1479	C	THR	458	B	2.90
262.	428	O	ASP	113	A	<-->	1480	O	THR	458	B	3.22
263.	428	O	ASP	113	A	<-->	1481	CB	THR	458	B	2.90
264.	428	O	ASP	113	A	<-->	1482	OG1	THR	458	B	2.92
265.	428	O	ASP	113	A	<-->	1483	CG2	THR	458	B	3.75
266.	428	O	ASP	113	A	<-->	1532	N	ASN	465	B	3.58
267.	423	CB	ASP	113	A	<-->	1464	CB	ARG	456	B	3.74
268.	423	CB	ASP	113	A	<-->	1466	CD	ARG	456	B	3.68
269.	423	CB	ASP	113	A	<-->	1474	O	CYS	457	B	3.71
270.	423	CB	ASP	113	A	<-->	1533	CA	ASN	465	B	3.69
271.	423	CB	ASP	113	A	<-->	1538	OD1	ASN	465	B	3.51
272.	424	CG	ASP	113	A	<-->	1464	CB	ARG	456	B	3.79
273.	424	CG	ASP	113	A	<-->	1466	CD	ARG	456	B	3.11
274.	424	CG	ASP	113	A	<-->	1538	OD1	ASN	465	B	3.53
275.	425	OD1	ASP	113	A	<-->	1464	CB	ARG	456	B	3.28
276.	425	OD1	ASP	113	A	<-->	1465	CG	ARG	456	B	3.74

277. 425 OD1 ASP 113 A <--> 1466 CD ARG 456 B 2.95
278. 426 OD2 ASP 113 A <--> 1466 CD ARG 456 B 3.55
279. 426 OD2 ASP 113 A <--> 1538 OD1 ASN 465 B 3.14
280. 429 N ASP 114 A <--> 1478 CA THR 458 B 3.32
281. 429 N ASP 114 A <--> 1481 CB THR 458 B 3.54
282. 429 N ASP 114 A <--> 1482 OG1 THR 458 B 3.16
283. 429 N ASP 114 A <--> 1483 CG2 THR 458 B 3.61
284. 430 CA ASP 114 A <--> 1478 CA THR 458 B 3.22
285. 430 CA ASP 114 A <--> 1479 C THR 458 B 3.57
286. 430 CA ASP 114 A <--> 1480 O THR 458 B 3.11
287. 430 CA ASP 114 A <--> 1481 CB THR 458 B 3.37
288. 430 CA ASP 114 A <--> 1482 OG1 THR 458 B 3.52
289. 430 CA ASP 114 A <--> 1483 CG2 THR 458 B 2.90
290. 435 C ASP 114 A <--> 1481 CB THR 458 B 3.88
291. 435 C ASP 114 A <--> 1483 CG2 THR 458 B 2.90
292. 431 CB ASP 114 A <--> 1479 C THR 458 B 3.88
293. 431 CB ASP 114 A <--> 1480 O THR 458 B 3.00
294. 431 CB ASP 114 A <--> 1531 CB ALA 464 B 3.79
295. 434 OD2 ASP 114 A <--> 1531 CB ALA 464 B 3.79
296. 437 N PRO 115 A <--> 1481 CB THR 458 B 3.44
297. 437 N PRO 115 A <--> 1482 OG1 THR 458 B 3.68
298. 437 N PRO 115 A <--> 1483 CG2 THR 458 B 2.13
299. 438 CA PRO 115 A <--> 1483 CG2 THR 458 B 3.21
300. 440 CB PRO 115 A <--> 1483 CG2 THR 458 B 3.07
301. 441 CG PRO 115 A <--> 1481 CB THR 458 B 3.19
302. 441 CG PRO 115 A <--> 1482 OG1 THR 458 B 3.67
303. 441 CG PRO 115 A <--> 1483 CG2 THR 458 B 1.99
304. 439 CD PRO 115 A <--> 1478 CA THR 458 B 3.29
305. 439 CD PRO 115 A <--> 1479 C THR 458 B 3.55
306. 439 CD PRO 115 A <--> 1480 O THR 458 B 3.67
307. 439 CD PRO 115 A <--> 1481 CB THR 458 B 2.25
308. 439 CD PRO 115 A <--> 1482 OG1 THR 458 B 2.85
309. 439 CD PRO 115 A <--> 1483 CG2 THR 458 B 0.78
310. 449 NE ARG 116 A <--> 1502 CG ARG 461 B 3.63
311. 450 CZ ARG 116 A <--> 1498 CA ARG 461 B 3.47
312. 450 CZ ARG 116 A <--> 1501 CB ARG 461 B 3.83
313. 450 CZ ARG 116 A <--> 1502 CG ARG 461 B 3.26
314. 450 CZ ARG 116 A <--> 1531 CB ALA 464 B 3.61
315. 451 NH1 ARG 116 A <--> 1480 O THR 458 B 3.38
316. 451 NH1 ARG 116 A <--> 1497 N ARG 461 B 3.79
317. 451 NH1 ARG 116 A <--> 1498 CA ARG 461 B 3.65
318. 451 NH1 ARG 116 A <--> 1502 CG ARG 461 B 3.60
319. 452 NH2 ARG 116 A <--> 1497 N ARG 461 B 3.74
320. 452 NH2 ARG 116 A <--> 1498 CA ARG 461 B 2.78
321. 452 NH2 ARG 116 A <--> 1499 C ARG 461 B 3.43
322. 452 NH2 ARG 116 A <--> 1500 O ARG 461 B 3.16
323. 452 NH2 ARG 116 A <--> 1501 CB ARG 461 B 3.35
324. 452 NH2 ARG 116 A <--> 1502 CG ARG 461 B 3.32
325. 452 NH2 ARG 116 A <--> 1531 CB ALA 464 B 2.76
326. 490 CB LEU 121 A <--> 1250 CD GLN 429 B 3.72
327. 490 CB LEU 121 A <--> 1251 OE1 GLN 429 B 3.12
328. 490 CB LEU 121 A <--> 1252 NE2 GLN 429 B 3.82
329. 491 CG LEU 121 A <--> 1250 CD GLN 429 B 3.30
330. 491 CG LEU 121 A <--> 1251 OE1 GLN 429 B 3.13
331. 491 CG LEU 121 A <--> 1252 NE2 GLN 429 B 3.37
332. 493 CD2 LEU 121 A <--> 1249 CG GLN 429 B 2.89
333. 493 CD2 LEU 121 A <--> 1250 CD GLN 429 B 1.94
334. 493 CD2 LEU 121 A <--> 1251 OE1 GLN 429 B 2.25
335. 493 CD2 LEU 121 A <--> 1252 NE2 GLN 429 B 1.95
336. 505 CA PRO 123 A <--> 1066 OD2 ASP 405 B 3.09

337.	509	C	PRO	123	A	<-->	1066	OD2	ASP	405	B	3.70
338.	510	O	PRO	123	A	<-->	1066	OD2	ASP	405	B	3.37
339.	507	CB	PRO	123	A	<-->	1066	OD2	ASP	405	B	3.64
340.	508	CG	PRO	123	A	<-->	885	NZ	LYS	383	B	3.45
341.	718	CD	ARG	150	A	<-->	1441	O	THR	452	B	3.69
342.	733	N	TYR	152	A	<-->	1229	CE1	HIS	426	B	3.84
343.	733	N	TYR	152	A	<-->	1230	NE2	HIS	426	B	3.47
344.	735	CB	TYR	152	A	<-->	1211	OG1	THR	424	B	3.83
345.	735	CB	TYR	152	A	<-->	1212	CG2	THR	424	B	3.30
346.	737	CD1	TYR	152	A	<-->	1024	CD2	LEU	400	B	3.03
347.	739	CE1	TYR	152	A	<-->	1024	CD2	LEU	400	B	3.14

Salt bridges

<----- A T O M 1 -----> <----- A T O M 2 ----->

	Atom no.	Atom name	Res name	Res no.	Chain		Atom no.	Atom name	Res name	Res no.	Chain	Distance
1.	41	OE2	GLU	64	A	<-->	456	NH1	ARG	328	B	3.26
2.	270	NH2	ARG	94	A	<-->	473	OD2	ASP	330	B	1.48
3.	315	OE2	GLU	100	A	<-->	1470	NH2	ARG	456	B	3.58
4.	425	OD1	ASP	113	A	<-->	1467	NE	ARG	456	B	3.95

Number of salt bridges: 4

Number of hydrogen bonds: 7

Number of non-bonded contacts: 347

[D] ROBO1.IG1 (WT) + Y323H

Hydrogen bonds

<----- A T O M 1 -----> <----- A T O M 2 ----->

	Atom no.	Atom name	Res name	Res no.	Chain		Atom no.	Atom name	Res name	Res no.	Chain	Distance
1.	134	OG	SER	75	A	<-->	1224	NE2	HIS	426	B	2.25
2.	138	O	ASP	76	A	<-->	1439	OG1	THR	452	B	2.51
3.	256	OE1	GLU	92	A	<-->	301	NE2	GLN	309	B	1.46
4.	261	O	GLY	93	A	<-->	129	NH1	ARG	287	B	2.94
5.	261	O	GLY	93	A	<-->	130	NH2	ARG	287	B	2.31

Non-bonded contacts

<----- A T O M 1 -----> <----- A T O M 2 ----->

	Atom no.	Atom name	Res name	Res no.	Chain		Atom no.	Atom name	Res name	Res no.	Chain	Distance
1.	10	O	LEU	61	A	<-->	145	O	LYS	289	B	3.62
2.	10	O	LEU	61	A	<-->	141	CD	LYS	289	B	3.81
3.	11	CB	LEU	61	A	<-->	145	O	LYS	289	B	3.61
4.	12	CG	LEU	61	A	<-->	144	C	LYS	289	B	3.39
5.	12	CG	LEU	61	A	<-->	145	O	LYS	289	B	3.45
6.	12	CG	LEU	61	A	<-->	146	N	GLY	290	B	3.48
7.	12	CG	LEU	61	A	<-->	147	CA	GLY	290	B	3.68
8.	14	CD2	LEU	61	A	<-->	144	C	LYS	289	B	3.09
9.	14	CD2	LEU	61	A	<-->	145	O	LYS	289	B	3.32
10.	14	CD2	LEU	61	A	<-->	146	N	GLY	290	B	2.62
11.	14	CD2	LEU	61	A	<-->	147	CA	GLY	290	B	2.43
12.	14	CD2	LEU	61	A	<-->	148	C	GLY	290	B	3.81
13.	26	N	GLN	63	A	<-->	141	CD	LYS	289	B	3.55
14.	26	N	GLN	63	A	<-->	142	CE	LYS	289	B	3.26
15.	26	N	GLN	63	A	<-->	143	NZ	LYS	289	B	3.06
16.	27	CA	GLN	63	A	<-->	142	CE	LYS	289	B	3.59
17.	27	CA	GLN	63	A	<-->	143	NZ	LYS	289	B	2.77
18.	28	C	GLN	63	A	<-->	143	NZ	LYS	289	B	3.61
19.	29	O	GLN	63	A	<-->	143	NZ	LYS	289	B	3.59
20.	30	CB	GLN	63	A	<-->	136	O	GLY	288	B	3.72
21.	30	CB	GLN	63	A	<-->	141	CD	LYS	289	B	3.50
22.	30	CB	GLN	63	A	<-->	142	CE	LYS	289	B	2.87
23.	30	CB	GLN	63	A	<-->	143	NZ	LYS	289	B	1.61
24.	31	CG	GLN	63	A	<-->	143	NZ	LYS	289	B	2.72
25.	32	CD	GLN	63	A	<-->	136	O	GLY	288	B	3.88
26.	33	OE1	GLN	63	A	<-->	134	CA	GLY	288	B	3.66
27.	33	OE1	GLN	63	A	<-->	135	C	GLY	288	B	3.70
28.	33	OE1	GLN	63	A	<-->	136	O	GLY	288	B	3.00
29.	87	NH2	ARG	69	A	<-->	301	NE2	GLN	309	B	3.02
30.	98	C	VAL	71	A	<-->	670	OH	TYR	356	B	3.37
31.	99	O	VAL	71	A	<-->	669	CZ	TYR	356	B	3.89
32.	99	O	VAL	71	A	<-->	670	OH	TYR	356	B	3.35
33.	100	CB	VAL	71	A	<-->	670	OH	TYR	356	B	3.35
34.	102	CG2	VAL	71	A	<-->	670	OH	TYR	356	B	2.82
35.	103	N	GLU	72	A	<-->	670	OH	TYR	356	B	3.67
36.	107	CB	GLU	72	A	<-->	670	OH	TYR	356	B	3.89
37.	109	CD	GLU	72	A	<-->	842	CD1	LEU	378	B	2.98

38.	110	OE1	GLU	72	A	<-->	668	CE2	TYR	356	B	3.78
39.	110	OE1	GLU	72	A	<-->	842	CD1	LEU	378	B	2.46
40.	111	OE2	GLU	72	A	<-->	842	CD1	LEU	378	B	3.01
41.	111	OE2	GLU	72	A	<-->	843	CD2	LEU	378	B	3.68
42.	133	CB	SER	75	A	<-->	1226	CE1	HIS	426	B	3.51
43.	133	CB	SER	75	A	<-->	1224	NE2	HIS	426	B	3.05
44.	134	OG	SER	75	A	<-->	1221	ND1	HIS	426	B	3.85
45.	134	OG	SER	75	A	<-->	1225	CD2	HIS	426	B	3.53
46.	134	OG	SER	75	A	<-->	1226	CE1	HIS	426	B	2.60
47.	134	OG	SER	75	A	<-->	1224	NE2	HIS	426	B	2.25
48.	137	C	ASP	76	A	<-->	1438	CB	THR	452	B	3.42
49.	137	C	ASP	76	A	<-->	1439	OG1	THR	452	B	3.68
50.	137	C	ASP	76	A	<-->	1440	CG2	THR	452	B	3.20
51.	138	O	ASP	76	A	<-->	1438	CB	THR	452	B	2.52
52.	138	O	ASP	76	A	<-->	1439	OG1	THR	452	B	2.51
53.	138	O	ASP	76	A	<-->	1440	CG2	THR	452	B	2.50
54.	143	N	LEU	77	A	<-->	1440	CG2	THR	452	B	3.52
55.	144	CA	LEU	77	A	<-->	1440	CG2	THR	452	B	3.26
56.	145	C	LEU	77	A	<-->	1440	CG2	THR	452	B	3.85
57.	147	CB	LEU	77	A	<-->	1207	OG1	THR	424	B	3.14
58.	148	CG	LEU	77	A	<-->	1203	O	GLN	423	B	3.71
59.	148	CG	LEU	77	A	<-->	1206	CB	THR	424	B	3.54
60.	148	CG	LEU	77	A	<-->	1207	OG1	THR	424	B	2.21
61.	149	CD1	LEU	77	A	<-->	1202	C	GLN	423	B	3.24
62.	149	CD1	LEU	77	A	<-->	1203	O	GLN	423	B	2.89
63.	149	CD1	LEU	77	A	<-->	1204	N	THR	424	B	3.20
64.	149	CD1	LEU	77	A	<-->	1205	CA	THR	424	B	2.72
65.	149	CD1	LEU	77	A	<-->	1206	CB	THR	424	B	2.21
66.	149	CD1	LEU	77	A	<-->	1207	OG1	THR	424	B	1.19
67.	149	CD1	LEU	77	A	<-->	1208	CG2	THR	424	B	2.85
68.	150	CD2	LEU	77	A	<-->	1206	CB	THR	424	B	3.87
69.	150	CD2	LEU	77	A	<-->	1207	OG1	THR	424	B	2.48
70.	151	N	ILE	78	A	<-->	1440	CG2	THR	452	B	3.68
71.	154	O	ILE	78	A	<-->	1203	O	GLN	423	B	2.81
72.	154	O	ILE	78	A	<-->	1420	CA	ILE	450	B	3.79
73.	154	O	ILE	78	A	<-->	1423	CG2	ILE	450	B	3.82
74.	155	CB	ILE	78	A	<-->	1418	O	PRO	449	B	2.83
75.	156	CG1	ILE	78	A	<-->	1418	O	PRO	449	B	3.67
76.	157	CG2	ILE	78	A	<-->	1418	O	PRO	449	B	3.01
77.	158	CD1	ILE	78	A	<-->	1418	O	PRO	449	B	3.47
78.	163	CB	VAL	79	A	<-->	1200	OE1	GLN	423	B	3.32
79.	165	CG2	VAL	79	A	<-->	1198	CG	GLN	423	B	3.90
80.	165	CG2	VAL	79	A	<-->	1199	CD	GLN	423	B	3.14
81.	165	CG2	VAL	79	A	<-->	1200	OE1	GLN	423	B	1.96
82.	186	CA	GLU	83	A	<-->	1199	CD	GLN	423	B	3.89
83.	186	CA	GLU	83	A	<-->	1200	OE1	GLN	423	B	3.60
84.	186	CA	GLU	83	A	<-->	1201	NE2	GLN	423	B	3.75
85.	187	C	GLU	83	A	<-->	1199	CD	GLN	423	B	3.48
86.	187	C	GLU	83	A	<-->	1200	OE1	GLN	423	B	3.29
87.	187	C	GLU	83	A	<-->	1201	NE2	GLN	423	B	3.71
88.	188	O	GLU	83	A	<-->	1200	OE1	GLN	423	B	3.56
89.	189	CB	GLU	83	A	<-->	1199	CD	GLN	423	B	3.10
90.	189	CB	GLU	83	A	<-->	1200	OE1	GLN	423	B	2.67
91.	189	CB	GLU	83	A	<-->	1201	NE2	GLN	423	B	2.93
92.	190	CG	GLU	83	A	<-->	1200	OE1	GLN	423	B	3.82
93.	190	CG	GLU	83	A	<-->	1201	NE2	GLN	423	B	3.73
94.	191	CD	GLU	83	A	<-->	1200	OE1	GLN	423	B	3.87
95.	191	CD	GLU	83	A	<-->	1201	NE2	GLN	423	B	3.87
96.	192	OE1	GLU	83	A	<-->	1199	CD	GLN	423	B	3.82
97.	192	OE1	GLU	83	A	<-->	1200	OE1	GLN	423	B	3.20

98.	192	OE1	GLU	83	A	<-->	1201	NE2	GLN	423	B	3.71
99.	194	N	PRO	84	A	<-->	1198	CG	GLN	423	B	3.66
100.	194	N	PRO	84	A	<-->	1199	CD	GLN	423	B	3.20
101.	194	N	PRO	84	A	<-->	1200	OE1	GLN	423	B	3.49
102.	194	N	PRO	84	A	<-->	1201	NE2	GLN	423	B	3.29
103.	195	CA	PRO	84	A	<-->	1198	CG	GLN	423	B	3.48
104.	195	CA	PRO	84	A	<-->	1199	CD	GLN	423	B	3.63
105.	196	C	PRO	84	A	<-->	1197	CB	GLN	423	B	3.60
106.	196	C	PRO	84	A	<-->	1198	CG	GLN	423	B	2.40
107.	196	C	PRO	84	A	<-->	1199	CD	GLN	423	B	2.97
108.	196	C	PRO	84	A	<-->	1200	OE1	GLN	423	B	3.33
109.	196	C	PRO	84	A	<-->	1201	NE2	GLN	423	B	3.79
110.	197	O	PRO	84	A	<-->	1196	CA	GLN	423	B	3.75
111.	197	O	PRO	84	A	<-->	1197	CB	GLN	423	B	2.53
112.	197	O	PRO	84	A	<-->	1198	CG	GLN	423	B	1.26
113.	197	O	PRO	84	A	<-->	1199	CD	GLN	423	B	1.84
114.	197	O	PRO	84	A	<-->	1200	OE1	GLN	423	B	2.32
115.	197	O	PRO	84	A	<-->	1201	NE2	GLN	423	B	2.85
116.	198	CB	PRO	84	A	<-->	1010	CG	ASN	399	B	3.57
117.	198	CB	PRO	84	A	<-->	1011	OD1	ASN	399	B	3.12
118.	198	CB	PRO	84	A	<-->	1012	ND2	ASN	399	B	3.53
119.	199	CG	PRO	84	A	<-->	1010	CG	ASN	399	B	3.83
120.	199	CG	PRO	84	A	<-->	1011	OD1	ASN	399	B	2.92
121.	199	CG	PRO	84	A	<-->	1186	O	ALA	421	B	3.23
122.	199	CG	PRO	84	A	<-->	1198	CG	GLN	423	B	3.79
123.	199	CG	PRO	84	A	<-->	1199	CD	GLN	423	B	3.88
124.	199	CG	PRO	84	A	<-->	1201	NE2	GLN	423	B	3.51
125.	200	CD	PRO	84	A	<-->	1186	O	ALA	421	B	3.87
126.	200	CD	PRO	84	A	<-->	1199	CD	GLN	423	B	3.48
127.	200	CD	PRO	84	A	<-->	1201	NE2	GLN	423	B	3.00
128.	201	N	ALA	85	A	<-->	1198	CG	GLN	423	B	3.23
129.	202	CA	ALA	85	A	<-->	1197	CB	GLN	423	B	3.61
130.	202	CA	ALA	85	A	<-->	1198	CG	GLN	423	B	3.37
131.	211	OG1	THR	86	A	<-->	1009	CB	ASN	399	B	3.57
132.	211	OG1	THR	86	A	<-->	1012	ND2	ASN	399	B	3.59
133.	226	CG	ASN	88	A	<-->	826	CD1	LEU	376	B	3.50
134.	226	CG	ASN	88	A	<-->	827	CD2	LEU	376	B	3.83
135.	226	CG	ASN	88	A	<-->	1020	CD2	LEU	400	B	3.00
136.	227	OD1	ASN	88	A	<-->	825	CG	LEU	376	B	3.50
137.	227	OD1	ASN	88	A	<-->	826	CD1	LEU	376	B	3.06
138.	227	OD1	ASN	88	A	<-->	827	CD2	LEU	376	B	2.80
139.	227	OD1	ASN	88	A	<-->	1020	CD2	LEU	400	B	3.59
140.	228	ND2	ASN	88	A	<-->	826	CD1	LEU	376	B	3.47
141.	228	ND2	ASN	88	A	<-->	1018	CG	LEU	400	B	3.33
142.	228	ND2	ASN	88	A	<-->	1020	CD2	LEU	400	B	1.82
143.	241	CD	LYS	90	A	<-->	469	OD2	ASP	330	B	3.03
144.	241	CD	LYS	90	A	<-->	483	OG	SER	332	B	3.84
145.	241	CD	LYS	90	A	<-->	670	OH	TYR	356	B	3.28
146.	242	CE	LYS	90	A	<-->	467	CG	ASP	330	B	2.74
147.	242	CE	LYS	90	A	<-->	468	OD1	ASP	330	B	3.19
148.	242	CE	LYS	90	A	<-->	469	OD2	ASP	330	B	1.66
149.	242	CE	LYS	90	A	<-->	483	OG	SER	332	B	3.80
150.	242	CE	LYS	90	A	<-->	649	CG1	VAL	354	B	3.78
151.	243	NZ	LYS	90	A	<-->	467	CG	ASP	330	B	3.42
152.	243	NZ	LYS	90	A	<-->	468	OD1	ASP	330	B	3.77
153.	243	NZ	LYS	90	A	<-->	469	OD2	ASP	330	B	2.50
154.	243	NZ	LYS	90	A	<-->	648	CB	VAL	354	B	3.35
155.	243	NZ	LYS	90	A	<-->	649	CG1	VAL	354	B	2.87
156.	243	NZ	LYS	90	A	<-->	650	CG2	VAL	354	B	3.19
157.	243	NZ	LYS	90	A	<-->	668	CE2	TYR	356	B	3.90

158.	247	O	ALA	91	A	<-->	129	NH1	ARG	287	B	3.82
159.	250	CA	GLU	92	A	<-->	129	NH1	ARG	287	B	3.36
160.	251	C	GLU	92	A	<-->	129	NH1	ARG	287	B	3.60
161.	253	CB	GLU	92	A	<-->	300	OE1	GLN	309	B	3.61
162.	254	CG	GLU	92	A	<-->	299	CD	GLN	309	B	3.27
163.	254	CG	GLU	92	A	<-->	300	OE1	GLN	309	B	2.61
164.	254	CG	GLU	92	A	<-->	301	NE2	GLN	309	B	3.41
165.	255	CD	GLU	92	A	<-->	298	CG	GLN	309	B	3.36
166.	255	CD	GLU	92	A	<-->	299	CD	GLN	309	B	2.17
167.	255	CD	GLU	92	A	<-->	300	OE1	GLN	309	B	2.12
168.	255	CD	GLU	92	A	<-->	301	NE2	GLN	309	B	2.12
169.	256	OE1	GLU	92	A	<-->	298	CG	GLN	309	B	3.54
170.	256	OE1	GLU	92	A	<-->	299	CD	GLN	309	B	2.25
171.	256	OE1	GLU	92	A	<-->	300	OE1	GLN	309	B	2.57
172.	256	OE1	GLU	92	A	<-->	301	NE2	GLN	309	B	1.46
173.	257	OE2	GLU	92	A	<-->	298	CG	GLN	309	B	2.82
174.	257	OE2	GLU	92	A	<-->	299	CD	GLN	309	B	2.19
175.	257	OE2	GLU	92	A	<-->	300	OE1	GLN	309	B	2.37
176.	257	OE2	GLU	92	A	<-->	301	NE2	GLN	309	B	2.51
177.	258	N	GLY	93	A	<-->	129	NH1	ARG	287	B	3.05
178.	260	C	GLY	93	A	<-->	129	NH1	ARG	287	B	3.85
179.	260	C	GLY	93	A	<-->	130	NH2	ARG	287	B	3.56
180.	261	O	GLY	93	A	<-->	128	CZ	ARG	287	B	2.94
181.	261	O	GLY	93	A	<-->	129	NH1	ARG	287	B	2.94
182.	261	O	GLY	93	A	<-->	130	NH2	ARG	287	B	2.31
183.	271	NH1	ARG	94	A	<-->	143	NZ	LYS	289	B	3.71
184.	272	NH2	ARG	94	A	<-->	62	CG2	THR	278	B	3.06
185.	280	N	THR	96	A	<-->	130	NH2	ARG	287	B	3.85
186.	281	CA	THR	96	A	<-->	130	NH2	ARG	287	B	3.56
187.	285	OG1	THR	96	A	<-->	113	OD2	ASP	285	B	3.41
188.	285	OG1	THR	96	A	<-->	130	NH2	ARG	287	B	3.48
189.	504	CD1	LEU	122	A	<-->	628	OD1	ASN	351	B	3.38
190.	507	CA	PRO	123	A	<-->	452	NH1	ARG	328	B	3.37
191.	508	C	PRO	123	A	<-->	451	CZ	ARG	328	B	3.26
192.	508	C	PRO	123	A	<-->	452	NH1	ARG	328	B	2.09
193.	509	O	PRO	123	A	<-->	451	CZ	ARG	328	B	3.11
194.	509	O	PRO	123	A	<-->	452	NH1	ARG	328	B	1.94
195.	509	O	PRO	123	A	<-->	453	NH2	ARG	328	B	3.54
196.	510	CB	PRO	123	A	<-->	255	OE1	GLU	304	B	2.97
197.	510	CB	PRO	123	A	<-->	452	NH1	ARG	328	B	3.60
198.	513	N	SER	124	A	<-->	449	CD	ARG	328	B	3.70
199.	513	N	SER	124	A	<-->	450	NE	ARG	328	B	3.53
200.	513	N	SER	124	A	<-->	451	CZ	ARG	328	B	2.86
201.	513	N	SER	124	A	<-->	452	NH1	ARG	328	B	2.15
202.	513	N	SER	124	A	<-->	453	NH2	ARG	328	B	3.57
203.	514	CA	SER	124	A	<-->	449	CD	ARG	328	B	3.66
204.	514	CA	SER	124	A	<-->	450	NE	ARG	328	B	2.95
205.	514	CA	SER	124	A	<-->	451	CZ	ARG	328	B	2.19
206.	514	CA	SER	124	A	<-->	452	NH1	ARG	328	B	2.23
207.	514	CA	SER	124	A	<-->	453	NH2	ARG	328	B	2.47
208.	515	C	SER	124	A	<-->	451	CZ	ARG	328	B	3.28
209.	515	C	SER	124	A	<-->	452	NH1	ARG	328	B	3.31
210.	515	C	SER	124	A	<-->	453	NH2	ARG	328	B	2.97
211.	516	O	SER	124	A	<-->	451	CZ	ARG	328	B	3.62
212.	516	O	SER	124	A	<-->	453	NH2	ARG	328	B	2.86
213.	517	CB	SER	124	A	<-->	449	CD	ARG	328	B	3.69
214.	517	CB	SER	124	A	<-->	450	NE	ARG	328	B	3.10
215.	517	CB	SER	124	A	<-->	451	CZ	ARG	328	B	2.96
216.	517	CB	SER	124	A	<-->	452	NH1	ARG	328	B	3.38
217.	517	CB	SER	124	A	<-->	453	NH2	ARG	328	B	3.24

218.	528	OG	SER	126	A	<-->	827	CD2	LEU	376	B	3.65
219.	543	CD1	PHE	128	A	<-->	819	NE2	GLN	375	B	3.68
220.	543	CD1	PHE	128	A	<-->	1012	ND2	ASN	399	B	3.28
221.	545	CE1	PHE	128	A	<-->	817	CD	GLN	375	B	3.73
222.	545	CE1	PHE	128	A	<-->	819	NE2	GLN	375	B	2.76
223.	545	CE1	PHE	128	A	<-->	1010	CG	ASN	399	B	3.89
224.	545	CE1	PHE	128	A	<-->	1012	ND2	ASN	399	B	2.58
225.	546	CE2	PHE	128	A	<-->	818	OE1	GLN	375	B	3.59
226.	546	CE2	PHE	128	A	<-->	819	NE2	GLN	375	B	3.39
227.	547	CZ	PHE	128	A	<-->	817	CD	GLN	375	B	3.52
228.	547	CZ	PHE	128	A	<-->	818	OE1	GLN	375	B	3.65
229.	547	CZ	PHE	128	A	<-->	819	NE2	GLN	375	B	2.59
230.	547	CZ	PHE	128	A	<-->	1012	ND2	ASN	399	B	3.62

Salt bridges

	<----- A T O M 1 ----->						<----- A T O M 2 ----->					
	Atom no.	Atom name	Res name	Res no.	Chain		Atom no.	Atom name	Res name	Res no.	Chain	Distance
1.	243	NZ	LYS	90	A	<-->	469	OD2	ASP	330	B	2.50

Number of salt bridges: 1

Number of hydrogen bonds: 5

Number of non-bonded contacts: 230

[E] C207S + SLIT2.D2 (WT)

Hydrogen bonds

	<----- A T O M 1 ----->					<----- A T O M 2 ----->					
	Atom no.	Atom name	Res name	Res no.	Chain		Atom no.	Atom name	Res name	Res no.	Chain	Distance
1.	133	N	ALA	49	A	<-->	1058	OH	TYR	404	B	2.98
2.	208	O	PRO	59	A	<-->	674	OH	TYR	356	B	2.38
3.	237	O	ILE	63	A	<-->	456	NH1	ARG	328	B	2.21
4.	532	OD2	ASP	101	A	<-->	674	OH	TYR	356	B	2.88

Non-bonded contacts

	<----- A T O M 1 ----->					<----- A T O M 2 ----->					
	Atom no.	Atom name	Res name	Res no.	Chain		Atom no.	Atom name	Res name	Res no.	Chain	Distance
1.	126	N	PRO	48	A	<-->	1252	NE2	GLN	429	B	3.39
2.	127	CA	PRO	48	A	<-->	1058	OH	TYR	404	B	2.83
3.	127	CA	PRO	48	A	<-->	1252	NE2	GLN	429	B	3.46
4.	131	C	PRO	48	A	<-->	1058	OH	TYR	404	B	3.00
5.	132	O	PRO	48	A	<-->	1058	OH	TYR	404	B	3.82
6.	129	CB	PRO	48	A	<-->	1057	CZ	TYR	404	B	3.30
7.	129	CB	PRO	48	A	<-->	1058	OH	TYR	404	B	2.05
8.	129	CB	PRO	48	A	<-->	1249	CG	GLN	429	B	3.40
9.	129	CB	PRO	48	A	<-->	1250	CD	GLN	429	B	3.55
10.	129	CB	PRO	48	A	<-->	1252	NE2	GLN	429	B	3.28
11.	130	CG	PRO	48	A	<-->	1058	OH	TYR	404	B	3.34
12.	130	CG	PRO	48	A	<-->	1249	CG	GLN	429	B	3.11
13.	130	CG	PRO	48	A	<-->	1250	CD	GLN	429	B	2.67
14.	130	CG	PRO	48	A	<-->	1251	OE1	GLN	429	B	3.28
15.	130	CG	PRO	48	A	<-->	1252	NE2	GLN	429	B	2.48
16.	128	CD	PRO	48	A	<-->	1250	CD	GLN	429	B	3.63
17.	128	CD	PRO	48	A	<-->	1252	NE2	GLN	429	B	2.95
18.	133	N	ALA	49	A	<-->	1058	OH	TYR	404	B	2.98
19.	137	O	ALA	49	A	<-->	1058	OH	TYR	404	B	3.63
20.	162	O	SER	52	A	<-->	863	ND2	ASN	380	B	3.76
21.	159	CB	SER	52	A	<-->	1052	CG	TYR	404	B	3.19
22.	159	CB	SER	52	A	<-->	1053	CD1	TYR	404	B	3.26
23.	159	CB	SER	52	A	<-->	1054	CD2	TYR	404	B	3.29
24.	159	CB	SER	52	A	<-->	1055	CE1	TYR	404	B	3.45
25.	159	CB	SER	52	A	<-->	1056	CE2	TYR	404	B	3.48
26.	159	CB	SER	52	A	<-->	1057	CZ	TYR	404	B	3.56
27.	160	OG	SER	52	A	<-->	1052	CG	TYR	404	B	3.53
28.	160	OG	SER	52	A	<-->	1053	CD1	TYR	404	B	3.58
29.	160	OG	SER	52	A	<-->	1054	CD2	TYR	404	B	3.13
30.	160	OG	SER	52	A	<-->	1055	CE1	TYR	404	B	3.27
31.	160	OG	SER	52	A	<-->	1056	CE2	TYR	404	B	2.76
32.	160	OG	SER	52	A	<-->	1057	CZ	TYR	404	B	2.85
33.	160	OG	SER	52	A	<-->	1058	OH	TYR	404	B	3.44
34.	168	O	CYS	53	A	<-->	863	ND2	ASN	380	B	3.39
35.	172	CG	GLN	54	A	<-->	672	CE2	TYR	356	B	3.70
36.	172	CG	GLN	54	A	<-->	846	CD1	LEU	378	B	3.49
37.	173	CD	GLN	54	A	<-->	670	CD2	TYR	356	B	3.65
38.	173	CD	GLN	54	A	<-->	672	CE2	TYR	356	B	3.48

39.	173	CD	GLN	54	A	<-->	845	CG	LEU	378	B	3.35
40.	173	CD	GLN	54	A	<-->	846	CD1	LEU	378	B	2.05
41.	173	CD	GLN	54	A	<-->	847	CD2	LEU	378	B	3.71
42.	174	OE1	GLN	54	A	<-->	844	CB	LEU	378	B	3.76
43.	174	OE1	GLN	54	A	<-->	845	CG	LEU	378	B	2.77
44.	174	OE1	GLN	54	A	<-->	846	CD1	LEU	378	B	1.85
45.	174	OE1	GLN	54	A	<-->	847	CD2	LEU	378	B	2.74
46.	175	NE2	GLN	54	A	<-->	653	CG1	VAL	354	B	3.89
47.	175	NE2	GLN	54	A	<-->	668	CG	TYR	356	B	3.68
48.	175	NE2	GLN	54	A	<-->	670	CD2	TYR	356	B	2.38
49.	175	NE2	GLN	54	A	<-->	672	CE2	TYR	356	B	2.41
50.	175	NE2	GLN	54	A	<-->	673	CZ	TYR	356	B	3.70
51.	175	NE2	GLN	54	A	<-->	844	CB	LEU	378	B	3.41
52.	175	NE2	GLN	54	A	<-->	845	CG	LEU	378	B	3.18
53.	175	NE2	GLN	54	A	<-->	846	CD1	LEU	378	B	1.95
54.	178	N	ALA	55	A	<-->	1024	CD2	LEU	400	B	3.71
55.	178	N	ALA	55	A	<-->	1229	CE1	HIS	426	B	3.40
56.	179	CA	ALA	55	A	<-->	1023	CD1	LEU	400	B	3.78
57.	179	CA	ALA	55	A	<-->	1024	CD2	LEU	400	B	3.35
58.	179	CA	ALA	55	A	<-->	1229	CE1	HIS	426	B	3.06
59.	179	CA	ALA	55	A	<-->	1230	NE2	HIS	426	B	3.61
60.	180	CB	ALA	55	A	<-->	1021	CB	LEU	400	B	3.85
61.	180	CB	ALA	55	A	<-->	1022	CG	LEU	400	B	2.72
62.	180	CB	ALA	55	A	<-->	1023	CD1	LEU	400	B	2.42
63.	180	CB	ALA	55	A	<-->	1024	CD2	LEU	400	B	2.03
64.	180	CB	ALA	55	A	<-->	1229	CE1	HIS	426	B	3.38
65.	207	C	PRO	59	A	<-->	674	OH	TYR	356	B	3.56
66.	208	O	PRO	59	A	<-->	672	CE2	TYR	356	B	3.85
67.	208	O	PRO	59	A	<-->	673	CZ	TYR	356	B	3.31
68.	208	O	PRO	59	A	<-->	674	OH	TYR	356	B	2.38
69.	210	CA	PRO	60	A	<-->	674	OH	TYR	356	B	3.57
70.	214	C	PRO	60	A	<-->	674	OH	TYR	356	B	3.82
71.	216	N	PRO	61	A	<-->	674	OH	TYR	356	B	3.09
72.	222	O	PRO	61	A	<-->	457	NH2	ARG	328	B	3.79
73.	222	O	PRO	61	A	<-->	473	OD2	ASP	330	B	3.81
74.	219	CB	PRO	61	A	<-->	653	CG1	VAL	354	B	3.84
75.	219	CB	PRO	61	A	<-->	672	CE2	TYR	356	B	2.98
76.	219	CB	PRO	61	A	<-->	673	CZ	TYR	356	B	3.70
77.	219	CB	PRO	61	A	<-->	674	OH	TYR	356	B	3.55
78.	219	CB	PRO	61	A	<-->	846	CD1	LEU	378	B	3.71
79.	220	CG	PRO	61	A	<-->	473	OD2	ASP	330	B	3.52
80.	220	CG	PRO	61	A	<-->	487	OG	SER	332	B	3.88
81.	220	CG	PRO	61	A	<-->	653	CG1	VAL	354	B	3.55
82.	220	CG	PRO	61	A	<-->	672	CE2	TYR	356	B	2.73
83.	220	CG	PRO	61	A	<-->	673	CZ	TYR	356	B	3.03
84.	220	CG	PRO	61	A	<-->	674	OH	TYR	356	B	2.73
85.	218	CD	PRO	61	A	<-->	672	CE2	TYR	356	B	3.34
86.	218	CD	PRO	61	A	<-->	673	CZ	TYR	356	B	3.06
87.	218	CD	PRO	61	A	<-->	674	OH	TYR	356	B	2.10
88.	228	C	THR	62	A	<-->	455	CZ	ARG	328	B	3.86
89.	228	C	THR	62	A	<-->	457	NH2	ARG	328	B	3.21
90.	229	O	THR	62	A	<-->	454	NE	ARG	328	B	3.34
91.	229	O	THR	62	A	<-->	455	CZ	ARG	328	B	2.91
92.	229	O	THR	62	A	<-->	456	NH1	ARG	328	B	3.58
93.	229	O	THR	62	A	<-->	457	NH2	ARG	328	B	2.61
94.	225	CB	THR	62	A	<-->	831	CD2	LEU	376	B	3.15
95.	226	OG1	THR	62	A	<-->	654	CG2	VAL	354	B	3.58
96.	226	OG1	THR	62	A	<-->	831	CD2	LEU	376	B	3.39
97.	227	CG2	THR	62	A	<-->	829	CG	LEU	376	B	3.71
98.	227	CG2	THR	62	A	<-->	831	CD2	LEU	376	B	2.41

99.	230	N	ILE	63	A	<-->	457	NH2	ARG	328	B	3.25
100.	231	CA	ILE	63	A	<-->	455	CZ	ARG	328	B	3.35
101.	231	CA	ILE	63	A	<-->	456	NH1	ARG	328	B	3.60
102.	231	CA	ILE	63	A	<-->	457	NH2	ARG	328	B	2.50
103.	236	C	ILE	63	A	<-->	455	CZ	ARG	328	B	3.56
104.	236	C	ILE	63	A	<-->	456	NH1	ARG	328	B	3.22
105.	236	C	ILE	63	A	<-->	457	NH2	ARG	328	B	3.23
106.	237	O	ILE	63	A	<-->	455	CZ	ARG	328	B	2.90
107.	237	O	ILE	63	A	<-->	456	NH1	ARG	328	B	2.21
108.	237	O	ILE	63	A	<-->	457	NH2	ARG	328	B	3.03
109.	232	CB	ILE	63	A	<-->	455	CZ	ARG	328	B	3.87
110.	232	CB	ILE	63	A	<-->	457	NH2	ARG	328	B	2.79
111.	234	CG2	ILE	63	A	<-->	275	CZ	ARG	306	B	3.45
112.	234	CG2	ILE	63	A	<-->	276	NH1	ARG	306	B	3.70
113.	234	CG2	ILE	63	A	<-->	277	NH2	ARG	306	B	2.99
114.	234	CG2	ILE	63	A	<-->	455	CZ	ARG	328	B	3.51
115.	234	CG2	ILE	63	A	<-->	457	NH2	ARG	328	B	2.24
116.	246	NH2	ARG	64	A	<-->	100	CD1	ILE	283	B	2.85
117.	465	NE2	GLN	92	A	<-->	303	NE2	GLN	309	B	3.89
118.	503	CA	HIS	98	A	<-->	131	NH1	ARG	287	B	3.83
119.	503	CA	HIS	98	A	<-->	132	NH2	ARG	287	B	3.86
120.	510	C	HIS	98	A	<-->	130	CZ	ARG	287	B	3.14
121.	510	C	HIS	98	A	<-->	131	NH1	ARG	287	B	2.53
122.	510	C	HIS	98	A	<-->	132	NH2	ARG	287	B	2.94
123.	511	O	HIS	98	A	<-->	130	CZ	ARG	287	B	2.98
124.	511	O	HIS	98	A	<-->	131	NH1	ARG	287	B	2.68
125.	511	O	HIS	98	A	<-->	132	NH2	ARG	287	B	2.63
126.	506	CB	HIS	98	A	<-->	132	NH2	ARG	287	B	3.62
127.	505	CG	HIS	98	A	<-->	130	CZ	ARG	287	B	3.61
128.	505	CG	HIS	98	A	<-->	132	NH2	ARG	287	B	2.48
129.	504	ND1	HIS	98	A	<-->	115	OD2	ASP	285	B	3.57
130.	504	ND1	HIS	98	A	<-->	129	NE	ARG	287	B	3.56
131.	504	ND1	HIS	98	A	<-->	130	CZ	ARG	287	B	2.56
132.	504	ND1	HIS	98	A	<-->	131	NH1	ARG	287	B	3.15
133.	504	ND1	HIS	98	A	<-->	132	NH2	ARG	287	B	1.56
134.	508	CD2	HIS	98	A	<-->	132	NH2	ARG	287	B	2.92
135.	509	CE1	HIS	98	A	<-->	113	CG	ASP	285	B	3.50
136.	509	CE1	HIS	98	A	<-->	115	OD2	ASP	285	B	2.33
137.	509	CE1	HIS	98	A	<-->	129	NE	ARG	287	B	3.40
138.	509	CE1	HIS	98	A	<-->	130	CZ	ARG	287	B	2.82
139.	509	CE1	HIS	98	A	<-->	131	NH1	ARG	287	B	3.84
140.	509	CE1	HIS	98	A	<-->	132	NH2	ARG	287	B	1.69
141.	507	NE2	HIS	98	A	<-->	115	OD2	ASP	285	B	2.67
142.	507	NE2	HIS	98	A	<-->	130	CZ	ARG	287	B	3.87
143.	507	NE2	HIS	98	A	<-->	132	NH2	ARG	287	B	2.59
144.	512	N	ALA	99	A	<-->	130	CZ	ARG	287	B	2.93
145.	512	N	ALA	99	A	<-->	131	NH1	ARG	287	B	1.88
146.	512	N	ALA	99	A	<-->	132	NH2	ARG	287	B	3.26
147.	513	CA	ALA	99	A	<-->	128	CD	ARG	287	B	3.71
148.	513	CA	ALA	99	A	<-->	129	NE	ARG	287	B	3.45
149.	513	CA	ALA	99	A	<-->	130	CZ	ARG	287	B	2.51
150.	513	CA	ALA	99	A	<-->	131	NH1	ARG	287	B	1.23
151.	513	CA	ALA	99	A	<-->	132	NH2	ARG	287	B	3.31
152.	515	C	ALA	99	A	<-->	131	NH1	ARG	287	B	2.70
153.	516	O	ALA	99	A	<-->	131	NH1	ARG	287	B	3.61
154.	514	CB	ALA	99	A	<-->	128	CD	ARG	287	B	2.88
155.	514	CB	ALA	99	A	<-->	129	NE	ARG	287	B	3.15
156.	514	CB	ALA	99	A	<-->	130	CZ	ARG	287	B	2.71
157.	514	CB	ALA	99	A	<-->	131	NH1	ARG	287	B	1.60
158.	514	CB	ALA	99	A	<-->	132	NH2	ARG	287	B	3.87

159.	514	CB	ALA	99	A	<-->	277	NH2	ARG	306	B	3.58
160.	514	CB	ALA	99	A	<-->	294	OE2	GLU	308	B	3.67
161.	517	N	HIS	100	A	<-->	131	NH1	ARG	287	B	3.39
162.	517	N	HIS	100	A	<-->	302	OE1	GLN	309	B	3.25
163.	517	N	HIS	100	A	<-->	303	NE2	GLN	309	B	3.89
164.	518	CA	HIS	100	A	<-->	301	CD	GLN	309	B	3.65
165.	518	CA	HIS	100	A	<-->	302	OE1	GLN	309	B	3.36
166.	518	CA	HIS	100	A	<-->	303	NE2	GLN	309	B	3.10
167.	525	C	HIS	100	A	<-->	300	CG	GLN	309	B	3.87
168.	525	C	HIS	100	A	<-->	301	CD	GLN	309	B	2.44
169.	525	C	HIS	100	A	<-->	302	OE1	GLN	309	B	2.37
170.	525	C	HIS	100	A	<-->	303	NE2	GLN	309	B	1.95
171.	526	O	HIS	100	A	<-->	300	CG	GLN	309	B	2.98
172.	526	O	HIS	100	A	<-->	301	CD	GLN	309	B	1.52
173.	526	O	HIS	100	A	<-->	302	OE1	GLN	309	B	1.15
174.	526	O	HIS	100	A	<-->	303	NE2	GLN	309	B	1.78
175.	521	CB	HIS	100	A	<-->	303	NE2	GLN	309	B	3.18
176.	527	N	ASP	101	A	<-->	301	CD	GLN	309	B	3.01
177.	527	N	ASP	101	A	<-->	302	OE1	GLN	309	B	3.31
178.	527	N	ASP	101	A	<-->	303	NE2	GLN	309	B	2.24
179.	528	CA	ASP	101	A	<-->	300	CG	GLN	309	B	3.56
180.	528	CA	ASP	101	A	<-->	301	CD	GLN	309	B	2.89
181.	528	CA	ASP	101	A	<-->	302	OE1	GLN	309	B	3.42
182.	528	CA	ASP	101	A	<-->	303	NE2	GLN	309	B	2.45
183.	533	C	ASP	101	A	<-->	301	CD	GLN	309	B	3.77
184.	533	C	ASP	101	A	<-->	303	NE2	GLN	309	B	3.07
185.	533	C	ASP	101	A	<-->	495	ND2	ASN	333	B	3.82
186.	534	O	ASP	101	A	<-->	303	NE2	GLN	309	B	3.68
187.	529	CB	ASP	101	A	<-->	303	NE2	GLN	309	B	3.90
188.	529	CB	ASP	101	A	<-->	495	ND2	ASN	333	B	3.67
189.	530	CG	ASP	101	A	<-->	302	OE1	GLN	309	B	3.85
190.	531	OD1	ASP	101	A	<-->	301	CD	GLN	309	B	3.65
191.	531	OD1	ASP	101	A	<-->	302	OE1	GLN	309	B	3.13
192.	532	OD2	ASP	101	A	<-->	674	OH	TYR	356	B	2.88
193.	535	N	GLY	102	A	<-->	300	CG	GLN	309	B	3.87
194.	535	N	GLY	102	A	<-->	303	NE2	GLN	309	B	3.66
195.	535	N	GLY	102	A	<-->	493	CG	ASN	333	B	3.52
196.	535	N	GLY	102	A	<-->	495	ND2	ASN	333	B	2.83
197.	536	CA	GLY	102	A	<-->	493	CG	ASN	333	B	3.73
198.	536	CA	GLY	102	A	<-->	495	ND2	ASN	333	B	3.41
199.	537	C	GLY	102	A	<-->	493	CG	ASN	333	B	2.98
200.	537	C	GLY	102	A	<-->	494	OD1	ASN	333	B	2.87
201.	537	C	GLY	102	A	<-->	495	ND2	ASN	333	B	2.86
202.	538	O	GLY	102	A	<-->	492	CB	ASN	333	B	3.19
203.	538	O	GLY	102	A	<-->	493	CG	ASN	333	B	1.84
204.	538	O	GLY	102	A	<-->	494	OD1	ASN	333	B	1.79
205.	538	O	GLY	102	A	<-->	495	ND2	ASN	333	B	1.83
206.	539	N	GLN	103	A	<-->	494	OD1	ASN	333	B	3.55
207.	540	CA	GLN	103	A	<-->	494	OD1	ASN	333	B	3.50
208.	542	CG	GLN	103	A	<-->	493	CG	ASN	333	B	3.90
209.	542	CG	GLN	103	A	<-->	494	OD1	ASN	333	B	3.41
210.	542	CG	GLN	103	A	<-->	495	ND2	ASN	333	B	3.54
211.	542	CG	GLN	103	A	<-->	671	CE1	TYR	356	B	3.88
212.	543	CD	GLN	103	A	<-->	671	CE1	TYR	356	B	3.49
213.	544	OE1	GLN	103	A	<-->	669	CD1	TYR	356	B	3.59
214.	544	OE1	GLN	103	A	<-->	671	CE1	TYR	356	B	3.54
215.	545	NE2	GLN	103	A	<-->	671	CE1	TYR	356	B	3.83
216.	684	N	ALA	123	A	<-->	1538	OD1	ASN	465	B	3.52
217.	687	C	ALA	123	A	<-->	1467	NE	ARG	456	B	3.65
218.	687	C	ALA	123	A	<-->	1468	CZ	ARG	456	B	3.73

219.	687	C	ALA	123	A	<-->	1538	OD1	ASN	465	B	3.76
220.	688	O	ALA	123	A	<-->	1466	CD	ARG	456	B	3.08
221.	688	O	ALA	123	A	<-->	1467	NE	ARG	456	B	2.80
222.	688	O	ALA	123	A	<-->	1468	CZ	ARG	456	B	2.97
223.	688	O	ALA	123	A	<-->	1469	NH1	ARG	456	B	3.31
224.	688	O	ALA	123	A	<-->	1470	NH2	ARG	456	B	3.60
225.	688	O	ALA	123	A	<-->	1537	CG	ASN	465	B	3.81
226.	688	O	ALA	123	A	<-->	1538	OD1	ASN	465	B	2.75
227.	689	N	VAL	124	A	<-->	1467	NE	ARG	456	B	3.77
228.	689	N	VAL	124	A	<-->	1468	CZ	ARG	456	B	3.77
229.	689	N	VAL	124	A	<-->	1470	NH2	ARG	456	B	3.58
230.	690	CA	VAL	124	A	<-->	1467	NE	ARG	456	B	2.98
231.	690	CA	VAL	124	A	<-->	1468	CZ	ARG	456	B	2.94
232.	690	CA	VAL	124	A	<-->	1470	NH2	ARG	456	B	2.49
233.	694	C	VAL	124	A	<-->	1467	NE	ARG	456	B	3.20
234.	694	C	VAL	124	A	<-->	1468	CZ	ARG	456	B	2.51
235.	694	C	VAL	124	A	<-->	1469	NH1	ARG	456	B	3.37
236.	694	C	VAL	124	A	<-->	1470	NH2	ARG	456	B	1.63
237.	695	O	VAL	124	A	<-->	1468	CZ	ARG	456	B	3.47
238.	695	O	VAL	124	A	<-->	1470	NH2	ARG	456	B	2.62
239.	691	CB	VAL	124	A	<-->	1470	NH2	ARG	456	B	3.35
240.	692	CG1	VAL	124	A	<-->	1470	NH2	ARG	456	B	3.08
241.	692	CG1	VAL	124	A	<-->	1559	NH2	ARG	467	B	3.87
242.	696	N	SER	125	A	<-->	1467	NE	ARG	456	B	2.72
243.	696	N	SER	125	A	<-->	1468	CZ	ARG	456	B	1.76
244.	696	N	SER	125	A	<-->	1469	NH1	ARG	456	B	2.65
245.	696	N	SER	125	A	<-->	1470	NH2	ARG	456	B	0.47
246.	696	N	SER	125	A	<-->	1556	NE	ARG	467	B	3.68
247.	696	N	SER	125	A	<-->	1557	CZ	ARG	467	B	3.66
248.	696	N	SER	125	A	<-->	1559	NH2	ARG	467	B	3.48
249.	697	CA	SER	125	A	<-->	1467	NE	ARG	456	B	3.74
250.	697	CA	SER	125	A	<-->	1468	CZ	ARG	456	B	2.51
251.	697	CA	SER	125	A	<-->	1469	NH1	ARG	456	B	2.78
252.	697	CA	SER	125	A	<-->	1470	NH2	ARG	456	B	1.60
253.	697	CA	SER	125	A	<-->	1554	CG	ARG	467	B	3.84
254.	697	CA	SER	125	A	<-->	1555	CD	ARG	467	B	3.79
255.	697	CA	SER	125	A	<-->	1556	NE	ARG	467	B	2.80
256.	697	CA	SER	125	A	<-->	1557	CZ	ARG	467	B	3.14
257.	697	CA	SER	125	A	<-->	1559	NH2	ARG	467	B	3.03
258.	700	C	SER	125	A	<-->	1468	CZ	ARG	456	B	3.84
259.	700	C	SER	125	A	<-->	1470	NH2	ARG	456	B	2.69
260.	700	C	SER	125	A	<-->	1555	CD	ARG	467	B	3.69
261.	700	C	SER	125	A	<-->	1556	NE	ARG	467	B	2.32
262.	700	C	SER	125	A	<-->	1557	CZ	ARG	467	B	2.27
263.	700	C	SER	125	A	<-->	1558	NH1	ARG	467	B	3.52
264.	700	C	SER	125	A	<-->	1559	NH2	ARG	467	B	1.74
265.	701	O	SER	125	A	<-->	1470	NH2	ARG	456	B	2.98
266.	701	O	SER	125	A	<-->	1556	NE	ARG	467	B	2.87
267.	701	O	SER	125	A	<-->	1557	CZ	ARG	467	B	2.18
268.	701	O	SER	125	A	<-->	1558	NH1	ARG	467	B	3.15
269.	701	O	SER	125	A	<-->	1559	NH2	ARG	467	B	1.15
270.	698	CB	SER	125	A	<-->	1467	NE	ARG	456	B	3.79
271.	698	CB	SER	125	A	<-->	1468	CZ	ARG	456	B	2.65
272.	698	CB	SER	125	A	<-->	1469	NH1	ARG	456	B	2.54
273.	698	CB	SER	125	A	<-->	1470	NH2	ARG	456	B	2.36
274.	698	CB	SER	125	A	<-->	1553	CB	ARG	467	B	3.87
275.	698	CB	SER	125	A	<-->	1554	CG	ARG	467	B	2.54
276.	698	CB	SER	125	A	<-->	1555	CD	ARG	467	B	2.66
277.	698	CB	SER	125	A	<-->	1556	NE	ARG	467	B	2.21
278.	698	CB	SER	125	A	<-->	1557	CZ	ARG	467	B	3.09

279.	698	CB	SER	125	A	<-->	1559	NH2	ARG	467	B	3.58
280.	699	OG	SER	125	A	<-->	1468	CZ	ARG	456	B	3.16
281.	699	OG	SER	125	A	<-->	1469	NH1	ARG	456	B	3.45
282.	699	OG	SER	125	A	<-->	1470	NH2	ARG	456	B	2.79
283.	699	OG	SER	125	A	<-->	1553	CB	ARG	467	B	3.01
284.	699	OG	SER	125	A	<-->	1554	CG	ARG	467	B	1.51
285.	699	OG	SER	125	A	<-->	1555	CD	ARG	467	B	1.70
286.	699	OG	SER	125	A	<-->	1556	NE	ARG	467	B	1.53
287.	699	OG	SER	125	A	<-->	1557	CZ	ARG	467	B	2.26
288.	699	OG	SER	125	A	<-->	1558	NH1	ARG	467	B	2.86
289.	699	OG	SER	125	A	<-->	1559	NH2	ARG	467	B	3.18
290.	702	N	ARG	126	A	<-->	1470	NH2	ARG	456	B	3.87
291.	702	N	ARG	126	A	<-->	1555	CD	ARG	467	B	3.65
292.	702	N	ARG	126	A	<-->	1556	NE	ARG	467	B	2.34
293.	702	N	ARG	126	A	<-->	1557	CZ	ARG	467	B	2.65
294.	702	N	ARG	126	A	<-->	1559	NH2	ARG	467	B	2.26
295.	703	CA	ARG	126	A	<-->	1556	NE	ARG	467	B	2.99
296.	703	CA	ARG	126	A	<-->	1557	CZ	ARG	467	B	2.95
297.	703	CA	ARG	126	A	<-->	1559	NH2	ARG	467	B	2.39
298.	711	C	ARG	126	A	<-->	1555	CD	ARG	467	B	3.48
299.	711	C	ARG	126	A	<-->	1556	NE	ARG	467	B	2.48
300.	711	C	ARG	126	A	<-->	1557	CZ	ARG	467	B	2.40
301.	711	C	ARG	126	A	<-->	1558	NH1	ARG	467	B	3.27
302.	711	C	ARG	126	A	<-->	1559	NH2	ARG	467	B	2.41
303.	712	O	ARG	126	A	<-->	1554	CG	ARG	467	B	3.71
304.	712	O	ARG	126	A	<-->	1555	CD	ARG	467	B	2.28
305.	712	O	ARG	126	A	<-->	1556	NE	ARG	467	B	1.56
306.	712	O	ARG	126	A	<-->	1557	CZ	ARG	467	B	1.89
307.	712	O	ARG	126	A	<-->	1558	NH1	ARG	467	B	2.66
308.	712	O	ARG	126	A	<-->	1559	NH2	ARG	467	B	2.56
309.	713	N	GLY	127	A	<-->	1556	NE	ARG	467	B	3.65
310.	713	N	GLY	127	A	<-->	1557	CZ	ARG	467	B	3.31
311.	713	N	GLY	127	A	<-->	1558	NH1	ARG	467	B	3.80
312.	713	N	GLY	127	A	<-->	1559	NH2	ARG	467	B	3.23
313.	714	CA	GLY	127	A	<-->	1557	CZ	ARG	467	B	3.76
314.	714	CA	GLY	127	A	<-->	1558	NH1	ARG	467	B	3.80
315.	715	C	GLY	127	A	<-->	1557	CZ	ARG	467	B	3.40
316.	715	C	GLY	127	A	<-->	1558	NH1	ARG	467	B	2.98
317.	715	C	GLY	127	A	<-->	1559	NH2	ARG	467	B	3.76
318.	716	O	GLY	127	A	<-->	1558	NH1	ARG	467	B	3.34
319.	717	N	ALA	128	A	<-->	1557	CZ	ARG	467	B	2.91
320.	717	N	ALA	128	A	<-->	1558	NH1	ARG	467	B	2.60
321.	717	N	ALA	128	A	<-->	1559	NH2	ARG	467	B	2.88
322.	718	CA	ALA	128	A	<-->	1557	CZ	ARG	467	B	3.40
323.	718	CA	ALA	128	A	<-->	1558	NH1	ARG	467	B	2.72
324.	718	CA	ALA	128	A	<-->	1559	NH2	ARG	467	B	3.32
325.	721	O	ALA	128	A	<-->	1440	C	THR	452	B	3.65
326.	721	O	ALA	128	A	<-->	1441	O	THR	452	B	2.78
327.	719	CB	ALA	128	A	<-->	1557	CZ	ARG	467	B	3.65
328.	719	CB	ALA	128	A	<-->	1558	NH1	ARG	467	B	3.31
329.	719	CB	ALA	128	A	<-->	1559	NH2	ARG	467	B	3.09
330.	739	C	LEU	130	A	<-->	1432	O	GLU	451	B	3.83
331.	740	O	LEU	130	A	<-->	1417	O	PRO	449	B	3.77
332.	740	O	LEU	130	A	<-->	1444	CG2	THR	452	B	3.34
333.	736	CG	LEU	130	A	<-->	1417	O	PRO	449	B	3.81
334.	737	CD1	LEU	130	A	<-->	1417	O	PRO	449	B	3.78
335.	738	CD2	LEU	130	A	<-->	1418	CB	PRO	449	B	3.49
336.	741	N	SER	131	A	<-->	1431	C	GLU	451	B	3.67
337.	741	N	SER	131	A	<-->	1432	O	GLU	451	B	2.82
338.	741	N	SER	131	A	<-->	1439	CA	THR	452	B	3.25

339.	742	CA	SER	131	A	<-->	1429	N	GLU	451	B	3.83
340.	742	CA	SER	131	A	<-->	1430	CA	GLU	451	B	3.61
341.	742	CA	SER	131	A	<-->	1431	C	GLU	451	B	2.25
342.	742	CA	SER	131	A	<-->	1432	O	GLU	451	B	1.60
343.	742	CA	SER	131	A	<-->	1438	N	THR	452	B	2.56
344.	742	CA	SER	131	A	<-->	1439	CA	THR	452	B	2.27
345.	742	CA	SER	131	A	<-->	1440	C	THR	452	B	3.31
346.	742	CA	SER	131	A	<-->	1441	O	THR	452	B	3.83
347.	742	CA	SER	131	A	<-->	1442	CB	THR	452	B	3.29
348.	742	CA	SER	131	A	<-->	1444	CG2	THR	452	B	3.32
349.	745	C	SER	131	A	<-->	1430	CA	GLU	451	B	3.78
350.	745	C	SER	131	A	<-->	1431	C	GLU	451	B	2.26
351.	745	C	SER	131	A	<-->	1432	O	GLU	451	B	2.11
352.	745	C	SER	131	A	<-->	1438	N	THR	452	B	1.88
353.	745	C	SER	131	A	<-->	1439	CA	THR	452	B	1.10
354.	745	C	SER	131	A	<-->	1440	C	THR	452	B	1.83
355.	745	C	SER	131	A	<-->	1441	O	THR	452	B	2.49
356.	745	C	SER	131	A	<-->	1442	CB	THR	452	B	2.50
357.	745	C	SER	131	A	<-->	1443	OG1	THR	452	B	3.53
358.	745	C	SER	131	A	<-->	1444	CG2	THR	452	B	3.23
359.	745	C	SER	131	A	<-->	1445	N	SER	453	B	2.72
360.	746	O	SER	131	A	<-->	1431	C	GLU	451	B	3.39
361.	746	O	SER	131	A	<-->	1432	O	GLU	451	B	3.08
362.	746	O	SER	131	A	<-->	1438	N	THR	452	B	2.93
363.	746	O	SER	131	A	<-->	1439	CA	THR	452	B	1.85
364.	746	O	SER	131	A	<-->	1440	C	THR	452	B	1.54
365.	746	O	SER	131	A	<-->	1441	O	THR	452	B	1.63
366.	746	O	SER	131	A	<-->	1442	CB	THR	452	B	3.02
367.	746	O	SER	131	A	<-->	1445	N	SER	453	B	2.55
368.	746	O	SER	131	A	<-->	1446	CA	SER	453	B	3.51
369.	746	O	SER	131	A	<-->	1451	N	GLY	454	B	3.62
370.	743	CB	SER	131	A	<-->	1429	N	GLU	451	B	3.18
371.	743	CB	SER	131	A	<-->	1430	CA	GLU	451	B	2.91
372.	743	CB	SER	131	A	<-->	1431	C	GLU	451	B	1.88
373.	743	CB	SER	131	A	<-->	1432	O	GLU	451	B	0.69
374.	743	CB	SER	131	A	<-->	1433	CB	GLU	451	B	3.39
375.	743	CB	SER	131	A	<-->	1436	OE1	GLU	451	B	3.71
376.	743	CB	SER	131	A	<-->	1438	N	THR	452	B	2.89
377.	743	CB	SER	131	A	<-->	1439	CA	THR	452	B	3.24
378.	744	OG	SER	131	A	<-->	1423	C	ILE	450	B	3.32
379.	744	OG	SER	131	A	<-->	1424	O	ILE	450	B	3.83
380.	744	OG	SER	131	A	<-->	1429	N	GLU	451	B	2.22
381.	744	OG	SER	131	A	<-->	1430	CA	GLU	451	B	1.54
382.	744	OG	SER	131	A	<-->	1431	C	GLU	451	B	0.94
383.	744	OG	SER	131	A	<-->	1432	O	GLU	451	B	0.95
384.	744	OG	SER	131	A	<-->	1433	CB	GLU	451	B	2.11
385.	744	OG	SER	131	A	<-->	1434	CG	GLU	451	B	3.13
386.	744	OG	SER	131	A	<-->	1435	CD	GLU	451	B	3.18
387.	744	OG	SER	131	A	<-->	1436	OE1	GLU	451	B	3.14
388.	744	OG	SER	131	A	<-->	1438	N	THR	452	B	2.26
389.	744	OG	SER	131	A	<-->	1439	CA	THR	452	B	3.23
390.	747	N	GLU	137	A	<-->	1244	N	GLN	429	B	3.76
391.	747	N	GLU	137	A	<-->	1245	CA	GLN	429	B	3.35
392.	747	N	GLU	137	A	<-->	1246	C	GLN	429	B	3.40
393.	747	N	GLU	137	A	<-->	1253	N	ASN	430	B	2.98
394.	747	N	GLU	137	A	<-->	1254	CA	ASN	430	B	3.62
395.	747	N	GLU	137	A	<-->	1255	C	ASN	430	B	3.09
396.	747	N	GLU	137	A	<-->	1256	O	ASN	430	B	2.65
397.	747	N	GLU	137	A	<-->	1261	N	PRO	431	B	3.79
398.	748	CA	GLU	137	A	<-->	1256	O	ASN	430	B	3.64

399.	748	CA	GLU	137	A	<-->	1482	OG1	THR	458	B	3.61
400.	754	C	GLU	137	A	<-->	1482	OG1	THR	458	B	3.87
401.	755	O	GLU	137	A	<-->	1462	C	ARG	456	B	3.74
402.	755	O	GLU	137	A	<-->	1463	O	ARG	456	B	3.36
403.	755	O	GLU	137	A	<-->	1464	CB	ARG	456	B	3.10
404.	749	CB	GLU	137	A	<-->	1256	O	ASN	430	B	3.66
405.	749	CB	GLU	137	A	<-->	1261	N	PRO	431	B	3.87
406.	749	CB	GLU	137	A	<-->	1262	CA	PRO	431	B	3.41
407.	749	CB	GLU	137	A	<-->	1481	CB	THR	458	B	3.70
408.	749	CB	GLU	137	A	<-->	1482	OG1	THR	458	B	2.28
409.	750	CG	GLU	137	A	<-->	1255	C	ASN	430	B	3.65
410.	750	CG	GLU	137	A	<-->	1256	O	ASN	430	B	3.83
411.	750	CG	GLU	137	A	<-->	1261	N	PRO	431	B	3.15
412.	750	CG	GLU	137	A	<-->	1262	CA	PRO	431	B	2.80
413.	750	CG	GLU	137	A	<-->	1265	CB	PRO	431	B	2.95
414.	750	CG	GLU	137	A	<-->	1267	CD	PRO	431	B	3.73
415.	750	CG	GLU	137	A	<-->	1482	OG1	THR	458	B	2.96
416.	751	CD	GLU	137	A	<-->	1261	N	PRO	431	B	3.50
417.	751	CD	GLU	137	A	<-->	1262	CA	PRO	431	B	2.60
418.	751	CD	GLU	137	A	<-->	1263	C	PRO	431	B	3.79
419.	751	CD	GLU	137	A	<-->	1265	CB	PRO	431	B	2.20
420.	751	CD	GLU	137	A	<-->	1266	CG	PRO	431	B	3.56
421.	751	CD	GLU	137	A	<-->	1481	CB	THR	458	B	3.48
422.	751	CD	GLU	137	A	<-->	1482	OG1	THR	458	B	2.62
423.	751	CD	GLU	137	A	<-->	1483	CG2	THR	458	B	3.45
424.	752	OE1	GLU	137	A	<-->	1262	CA	PRO	431	B	3.74
425.	752	OE1	GLU	137	A	<-->	1265	CB	PRO	431	B	3.26
426.	752	OE1	GLU	137	A	<-->	1481	CB	THR	458	B	3.34
427.	752	OE1	GLU	137	A	<-->	1482	OG1	THR	458	B	2.74
428.	752	OE1	GLU	137	A	<-->	1483	CG2	THR	458	B	2.84
429.	753	OE2	GLU	137	A	<-->	1261	N	PRO	431	B	2.96
430.	753	OE2	GLU	137	A	<-->	1262	CA	PRO	431	B	1.92
431.	753	OE2	GLU	137	A	<-->	1263	C	PRO	431	B	2.96
432.	753	OE2	GLU	137	A	<-->	1264	O	PRO	431	B	3.20
433.	753	OE2	GLU	137	A	<-->	1265	CB	PRO	431	B	1.04
434.	753	OE2	GLU	137	A	<-->	1266	CG	PRO	431	B	2.52
435.	753	OE2	GLU	137	A	<-->	1267	CD	PRO	431	B	3.22
436.	753	OE2	GLU	137	A	<-->	1481	CB	THR	458	B	3.68
437.	753	OE2	GLU	137	A	<-->	1482	OG1	THR	458	B	3.11
438.	753	OE2	GLU	137	A	<-->	1483	CG2	THR	458	B	3.73
439.	757	CA	ASP	138	A	<-->	1466	CD	ARG	456	B	3.53
440.	757	CA	ASP	138	A	<-->	1538	OD1	ASN	465	B	3.40
441.	762	C	ASP	138	A	<-->	1537	CG	ASN	465	B	3.79
442.	762	C	ASP	138	A	<-->	1538	OD1	ASN	465	B	3.14
443.	763	O	ASP	138	A	<-->	1536	CB	ASN	465	B	3.66
444.	763	O	ASP	138	A	<-->	1537	CG	ASN	465	B	2.96
445.	763	O	ASP	138	A	<-->	1538	OD1	ASN	465	B	2.43
446.	763	O	ASP	138	A	<-->	1539	ND2	ASN	465	B	3.66
447.	758	CB	ASP	138	A	<-->	1466	CD	ARG	456	B	3.05
448.	758	CB	ASP	138	A	<-->	1467	NE	ARG	456	B	3.76
449.	758	CB	ASP	138	A	<-->	1538	OD1	ASN	465	B	3.13
450.	759	CG	ASP	138	A	<-->	1465	CG	ARG	456	B	3.84
451.	759	CG	ASP	138	A	<-->	1466	CD	ARG	456	B	2.63
452.	759	CG	ASP	138	A	<-->	1467	NE	ARG	456	B	3.13
453.	760	OD1	ASP	138	A	<-->	1464	CB	ARG	456	B	2.94
454.	760	OD1	ASP	138	A	<-->	1465	CG	ARG	456	B	3.24
455.	760	OD1	ASP	138	A	<-->	1466	CD	ARG	456	B	2.47
456.	760	OD1	ASP	138	A	<-->	1467	NE	ARG	456	B	3.27
457.	761	OD2	ASP	138	A	<-->	1252	NE2	GLN	429	B	3.68
458.	761	OD2	ASP	138	A	<-->	1466	CD	ARG	456	B	3.30

459. 761 OD2 ASP 138 A <--> 1467 NE ARG 456 B 3.24
460. 773 C PHE 139 A <--> 1536 CB ASN 465 B 3.81
461. 774 O PHE 139 A <--> 1528 CA ALA 464 B 3.76
462. 774 O PHE 139 A <--> 1529 C ALA 464 B 3.48
463. 774 O PHE 139 A <--> 1531 CB ALA 464 B 3.48
464. 774 O PHE 139 A <--> 1532 N ASN 465 B 3.00
465. 774 O PHE 139 A <--> 1533 CA ASN 465 B 3.55
466. 774 O PHE 139 A <--> 1536 CB ASN 465 B 3.51
467. 775 N GLN 140 A <--> 1536 CB ASN 465 B 3.85
468. 776 CA GLN 140 A <--> 1531 CB ALA 464 B 3.82
469. 776 CA GLN 140 A <--> 1536 CB ASN 465 B 3.60
470. 777 CB GLN 140 A <--> 1530 O ALA 464 B 3.83
471. 777 CB GLN 140 A <--> 1536 CB ASN 465 B 2.91
472. 777 CB GLN 140 A <--> 1537 CG ASN 465 B 3.51
473. 777 CB GLN 140 A <--> 1539 ND2 ASN 465 B 3.35
474. 778 CG GLN 140 A <--> 1539 ND2 ASN 465 B 3.33
475. 779 CD GLN 140 A <--> 1539 ND2 ASN 465 B 3.14
476. 780 OE1 GLN 140 A <--> 1539 ND2 ASN 465 B 3.59
477. 781 NE2 GLN 140 A <--> 1536 CB ASN 465 B 3.87
478. 781 NE2 GLN 140 A <--> 1539 ND2 ASN 465 B 3.30
479. 1127 NH2 ARG 184 A <--> 884 CE LYS 383 B 3.85
480. 1127 NH2 ARG 184 A <--> 885 NZ LYS 383 B 2.76
481. 1131 CA HIS 185 A <--> 695 NZ LYS 359 B 3.42
482. 1138 C HIS 185 A <--> 695 NZ LYS 359 B 3.42
483. 1134 CB HIS 185 A <--> 692 CG LYS 359 B 3.61
484. 1134 CB HIS 185 A <--> 693 CD LYS 359 B 3.73
485. 1134 CB HIS 185 A <--> 694 CE LYS 359 B 3.75
486. 1134 CB HIS 185 A <--> 695 NZ LYS 359 B 2.66
487. 1133 CG HIS 185 A <--> 692 CG LYS 359 B 2.92
488. 1133 CG HIS 185 A <--> 693 CD LYS 359 B 3.47
489. 1133 CG HIS 185 A <--> 695 NZ LYS 359 B 3.40
490. 1132 ND1 HIS 185 A <--> 681 C ASN 358 B 3.50
491. 1132 ND1 HIS 185 A <--> 682 O ASN 358 B 2.67
492. 1132 ND1 HIS 185 A <--> 688 CA LYS 359 B 3.78
493. 1132 ND1 HIS 185 A <--> 691 CB LYS 359 B 3.84
494. 1132 ND1 HIS 185 A <--> 692 CG LYS 359 B 2.89
495. 1136 CD2 HIS 185 A <--> 692 CG LYS 359 B 3.13
496. 1136 CD2 HIS 185 A <--> 693 CD LYS 359 B 3.54
497. 1137 CE1 HIS 185 A <--> 679 N ASN 358 B 3.68
498. 1137 CE1 HIS 185 A <--> 680 CA ASN 358 B 3.41
499. 1137 CE1 HIS 185 A <--> 681 C ASN 358 B 2.65
500. 1137 CE1 HIS 185 A <--> 682 O ASN 358 B 2.04
501. 1137 CE1 HIS 185 A <--> 687 N LYS 359 B 3.44
502. 1137 CE1 HIS 185 A <--> 688 CA LYS 359 B 3.74
503. 1137 CE1 HIS 185 A <--> 692 CG LYS 359 B 3.08
504. 1135 NE2 HIS 185 A <--> 678 O GLY 357 B 3.87
505. 1135 NE2 HIS 185 A <--> 680 CA ASN 358 B 3.78
506. 1135 NE2 HIS 185 A <--> 681 C ASN 358 B 3.41
507. 1135 NE2 HIS 185 A <--> 682 O ASN 358 B 3.17
508. 1135 NE2 HIS 185 A <--> 692 CG LYS 359 B 3.26
509. 1140 N THR 186 A <--> 694 CE LYS 359 B 3.78
510. 1140 N THR 186 A <--> 695 NZ LYS 359 B 2.78
511. 1141 CA THR 186 A <--> 695 NZ LYS 359 B 3.61
512. 1145 C THR 186 A <--> 695 NZ LYS 359 B 3.72
513. 1146 O THR 186 A <--> 695 NZ LYS 359 B 3.34
514. 1142 CB THR 186 A <--> 695 NZ LYS 359 B 3.80

Salt bridges

	<----- A T O M 1 ----->						<----- A T O M 2 ----->					
	Atom no.	Atom name	Res name	Res no.	Chain		Atom no.	Atom name	Res name	Res no.	Chain	Distance
1.	507	NE2	HIS	98	A	<-->	114	OD1	ASP	285	B	2.67
2.	761	OD2	ASP	138	A	<-->	1467	NE	ARG	456	B	3.24

Number of salt bridges: 2

Number of hydrogen bonds: 4

Number of non-bonded contacts: 514

[F] ROBO4.IG1-2 (WT) + R388W

Hydrogen bonds

	<----- A T O M 1 ----->						<----- A T O M 2 ----->					
	Atom no.	Atom name	Res name	Res no.	Chain		Atom no.	Atom name	Res name	Res no.	Chain	Distance
1.	1157	O	SER	188	A	<-->	246	OG1	THR	303	B	2.73
2.	1164	N	GLY	190	A	<-->	84	O	ASN	281	B	2.69
3.	1282	OH	TYR	205	A	<-->	51	O	ALA	276	B	2.76
4.	1304	N	ALA	209	A	<-->	34	O	CYS	273	B	2.71
5.	1307	O	ALA	209	A	<-->	29	N	CYS	273	B	1.57
6.	1312	O	THR	210	A	<-->	8	OG	SER	270	B	3.18
7.	1314	OG1	THR	210	A	<-->	201	ND2	ASN	297	B	1.57
8.	1316	N	ASN	211	A	<-->	203	O	ASN	297	B	3.23
9.	1323	ND2	ASN	211	A	<-->	8	OG	SER	270	B	2.72
10.	1329	OG	SER	212	A	<-->	200	OD1	ASN	297	B	2.24
11.	1396	N	ARG	222	A	<-->	615	OG	SER	349	B	1.50

Non-bonded contacts

	<----- A T O M 1 ----->						<----- A T O M 2 ----->					
	Atom no.	Atom name	Res name	Res no.	Chain		Atom no.	Atom name	Res name	Res no.	Chain	Distance
1.	1018	CG	TRP	171	A	<-->	1	N	GLY	269	B	3.83
2.	1020	CD2	TRP	171	A	<-->	1	N	GLY	269	B	3.20
3.	1022	CE2	TRP	171	A	<-->	1	N	GLY	269	B	3.43
4.	1023	CE3	TRP	171	A	<-->	1	N	GLY	269	B	3.23
5.	1024	CZ2	TRP	171	A	<-->	1	N	GLY	269	B	3.67
6.	1025	CZ3	TRP	171	A	<-->	1	N	GLY	269	B	3.46
7.	1026	CH2	TRP	171	A	<-->	1	N	GLY	269	B	3.66
8.	1140	N	THR	186	A	<-->	628	CB	ASN	351	B	3.57
9.	1140	N	THR	186	A	<-->	629	CG	ASN	351	B	2.72
10.	1140	N	THR	186	A	<-->	630	OD1	ASN	351	B	2.64
11.	1140	N	THR	186	A	<-->	631	ND2	ASN	351	B	2.96
12.	1141	CA	THR	186	A	<-->	628	CB	ASN	351	B	2.89
13.	1141	CA	THR	186	A	<-->	629	CG	ASN	351	B	2.54
14.	1141	CA	THR	186	A	<-->	630	OD1	ASN	351	B	2.39
15.	1141	CA	THR	186	A	<-->	631	ND2	ASN	351	B	3.34
16.	1142	C	THR	186	A	<-->	629	CG	ASN	351	B	3.72
17.	1142	C	THR	186	A	<-->	630	OD1	ASN	351	B	3.11
18.	1143	O	THR	186	A	<-->	630	OD1	ASN	351	B	3.74
19.	1144	CB	THR	186	A	<-->	446	O	ARG	327	B	3.54
20.	1144	CB	THR	186	A	<-->	626	N	ASN	351	B	3.88
21.	1144	CB	THR	186	A	<-->	627	CA	ASN	351	B	3.04
22.	1144	CB	THR	186	A	<-->	632	C	ASN	351	B	3.33
23.	1144	CB	THR	186	A	<-->	628	CB	ASN	351	B	1.73
24.	1144	CB	THR	186	A	<-->	629	CG	ASN	351	B	1.50
25.	1144	CB	THR	186	A	<-->	630	OD1	ASN	351	B	1.39
26.	1144	CB	THR	186	A	<-->	631	ND2	ASN	351	B	2.73
27.	1144	CB	THR	186	A	<-->	634	N	SER	352	B	3.64
28.	1145	OG1	THR	186	A	<-->	626	N	ASN	351	B	3.40
29.	1145	OG1	THR	186	A	<-->	627	CA	ASN	351	B	2.30
30.	1145	OG1	THR	186	A	<-->	632	C	ASN	351	B	2.99
31.	1145	OG1	THR	186	A	<-->	633	O	ASN	351	B	3.47

32. 1145 OG1 THR 186 A <--> 628 CB ASN 351 B 0.96
33. 1145 OG1 THR 186 A <--> 629 CG ASN 351 B 0.74
34. 1145 OG1 THR 186 A <--> 630 OD1 ASN 351 B 1.81
35. 1145 OG1 THR 186 A <--> 631 ND2 ASN 351 B 1.65
36. 1145 OG1 THR 186 A <--> 634 N SER 352 B 3.77
37. 1146 CG2 THR 186 A <--> 437 CA ARG 327 B 3.60
38. 1146 CG2 THR 186 A <--> 445 C ARG 327 B 2.91
39. 1146 CG2 THR 186 A <--> 446 O ARG 327 B 2.01
40. 1146 CG2 THR 186 A <--> 438 CB ARG 327 B 3.45
41. 1146 CG2 THR 186 A <--> 447 N ARG 328 B 3.86
42. 1146 CG2 THR 186 A <--> 626 N ASN 351 B 3.12
43. 1146 CG2 THR 186 A <--> 627 CA ASN 351 B 2.87
44. 1146 CG2 THR 186 A <--> 632 C ASN 351 B 3.18
45. 1146 CG2 THR 186 A <--> 628 CB ASN 351 B 1.98
46. 1146 CG2 THR 186 A <--> 629 CG ASN 351 B 2.70
47. 1146 CG2 THR 186 A <--> 630 OD1 ASN 351 B 2.68
48. 1146 CG2 THR 186 A <--> 634 N SER 352 B 3.02
49. 1146 CG2 THR 186 A <--> 636 CB SER 352 B 3.86
50. 1147 N VAL 187 A <--> 449 CB ARG 328 B 3.84
51. 1147 N VAL 187 A <--> 630 OD1 ASN 351 B 3.72
52. 1148 CA VAL 187 A <--> 255 OE1 GLU 304 B 3.72
53. 1148 CA VAL 187 A <--> 449 CB ARG 328 B 3.61
54. 1148 CA VAL 187 A <--> 451 CD ARG 328 B 3.18
55. 1148 CA VAL 187 A <--> 454 NH1 ARG 328 B 3.78
56. 1149 C VAL 187 A <--> 255 OE1 GLU 304 B 3.47
57. 1150 O VAL 187 A <--> 255 OE1 GLU 304 B 3.66
58. 1150 O VAL 187 A <--> 454 NH1 ARG 328 B 3.81
59. 1151 CB VAL 187 A <--> 451 CD ARG 328 B 3.14
60. 1151 CB VAL 187 A <--> 452 NE ARG 328 B 3.63
61. 1151 CB VAL 187 A <--> 453 CZ ARG 328 B 3.63
62. 1151 CB VAL 187 A <--> 454 NH1 ARG 328 B 3.15
63. 1152 CG1 VAL 187 A <--> 255 OE1 GLU 304 B 3.70
64. 1152 CG1 VAL 187 A <--> 451 CD ARG 328 B 2.95
65. 1152 CG1 VAL 187 A <--> 452 NE ARG 328 B 2.99
66. 1152 CG1 VAL 187 A <--> 453 CZ ARG 328 B 2.50
67. 1152 CG1 VAL 187 A <--> 454 NH1 ARG 328 B 1.72
68. 1152 CG1 VAL 187 A <--> 455 NH2 ARG 328 B 3.48
69. 1153 CG2 VAL 187 A <--> 450 CG ARG 328 B 3.61
70. 1153 CG2 VAL 187 A <--> 451 CD ARG 328 B 3.00
71. 1153 CG2 VAL 187 A <--> 452 NE ARG 328 B 3.38
72. 1153 CG2 VAL 187 A <--> 453 CZ ARG 328 B 3.77
73. 1153 CG2 VAL 187 A <--> 454 NH1 ARG 328 B 3.84
74. 1153 CG2 VAL 187 A <--> 636 CB SER 352 B 3.75
75. 1153 CG2 VAL 187 A <--> 637 OG SER 352 B 3.25
76. 1154 N SER 188 A <--> 255 OE1 GLU 304 B 3.81
77. 1156 C SER 188 A <--> 246 OG1 THR 303 B 3.67
78. 1157 O SER 188 A <--> 245 CB THR 303 B 3.21
79. 1157 O SER 188 A <--> 246 OG1 THR 303 B 2.73
80. 1157 O SER 188 A <--> 441 NE ARG 327 B 3.45
81. 1158 CB SER 188 A <--> 440 CD ARG 327 B 3.86
82. 1158 CB SER 188 A <--> 441 NE ARG 327 B 3.60
83. 1160 N GLY 189 A <--> 98 CD1 ILE 283 B 3.59
84. 1160 N GLY 189 A <--> 246 OG1 THR 303 B 3.86
85. 1161 CA GLY 189 A <--> 96 CG1 ILE 283 B 3.15
86. 1161 CA GLY 189 A <--> 98 CD1 ILE 283 B 2.85
87. 1161 CA GLY 189 A <--> 246 OG1 THR 303 B 3.12
88. 1162 C GLY 189 A <--> 84 O ASN 281 B 3.75
89. 1162 C GLY 189 A <--> 246 OG1 THR 303 B 3.09
90. 1163 O GLY 189 A <--> 245 CB THR 303 B 3.78
91. 1163 O GLY 189 A <--> 246 OG1 THR 303 B 3.19

92.	1163	O	GLY	189	A	<-->	247	CG2	THR	303	B	3.77
93.	1163	O	GLY	189	A	<-->	444	NH2	ARG	327	B	3.42
94.	1164	N	GLY	190	A	<-->	83	C	ASN	281	B	3.84
95.	1164	N	GLY	190	A	<-->	84	O	ASN	281	B	2.69
96.	1164	N	GLY	190	A	<-->	80	CG	ASN	281	B	3.41
97.	1164	N	GLY	190	A	<-->	81	OD1	ASN	281	B	3.81
98.	1164	N	GLY	190	A	<-->	82	ND2	ASN	281	B	2.89
99.	1164	N	GLY	190	A	<-->	246	OG1	THR	303	B	3.74
100.	1165	CA	GLY	190	A	<-->	84	O	ASN	281	B	3.17
101.	1165	CA	GLY	190	A	<-->	80	CG	ASN	281	B	3.33
102.	1165	CA	GLY	190	A	<-->	81	OD1	ASN	281	B	3.24
103.	1165	CA	GLY	190	A	<-->	82	ND2	ASN	281	B	3.09
104.	1166	C	GLY	190	A	<-->	78	CA	ASN	281	B	3.62
105.	1166	C	GLY	190	A	<-->	83	C	ASN	281	B	3.27
106.	1166	C	GLY	190	A	<-->	84	O	ASN	281	B	2.75
107.	1166	C	GLY	190	A	<-->	79	CB	ASN	281	B	3.39
108.	1166	C	GLY	190	A	<-->	80	CG	ASN	281	B	2.20
109.	1166	C	GLY	190	A	<-->	81	OD1	ASN	281	B	1.82
110.	1166	C	GLY	190	A	<-->	82	ND2	ASN	281	B	2.52
111.	1167	O	GLY	190	A	<-->	78	CA	ASN	281	B	3.32
112.	1167	O	GLY	190	A	<-->	83	C	ASN	281	B	3.45
113.	1167	O	GLY	190	A	<-->	84	O	ASN	281	B	3.03
114.	1167	O	GLY	190	A	<-->	79	CB	ASN	281	B	2.63
115.	1167	O	GLY	190	A	<-->	80	CG	ASN	281	B	1.17
116.	1167	O	GLY	190	A	<-->	81	OD1	ASN	281	B	1.07
117.	1167	O	GLY	190	A	<-->	82	ND2	ASN	281	B	1.54
118.	1168	N	SER	191	A	<-->	78	CA	ASN	281	B	3.55
119.	1168	N	SER	191	A	<-->	83	C	ASN	281	B	3.14
120.	1168	N	SER	191	A	<-->	84	O	ASN	281	B	3.03
121.	1168	N	SER	191	A	<-->	79	CB	ASN	281	B	3.84
122.	1168	N	SER	191	A	<-->	80	CG	ASN	281	B	2.98
123.	1168	N	SER	191	A	<-->	81	OD1	ASN	281	B	2.22
124.	1168	N	SER	191	A	<-->	82	ND2	ASN	281	B	3.70
125.	1168	N	SER	191	A	<-->	85	N	ASN	282	B	3.79
126.	1168	N	SER	191	A	<-->	87	CB	ASN	282	B	3.47
127.	1168	N	SER	191	A	<-->	88	CG	ASN	282	B	3.62
128.	1168	N	SER	191	A	<-->	90	ND2	ASN	282	B	2.96
129.	1169	CA	SER	191	A	<-->	78	CA	ASN	281	B	3.14
130.	1169	CA	SER	191	A	<-->	83	C	ASN	281	B	3.27
131.	1169	CA	SER	191	A	<-->	84	O	ASN	281	B	3.64
132.	1169	CA	SER	191	A	<-->	79	CB	ASN	281	B	3.64
133.	1169	CA	SER	191	A	<-->	80	CG	ASN	281	B	3.06
134.	1169	CA	SER	191	A	<-->	81	OD1	ASN	281	B	2.07
135.	1169	CA	SER	191	A	<-->	85	N	ASN	282	B	3.82
136.	1169	CA	SER	191	A	<-->	90	ND2	ASN	282	B	3.16
137.	1170	C	SER	191	A	<-->	78	CA	ASN	281	B	3.05
138.	1170	C	SER	191	A	<-->	83	C	ASN	281	B	3.01
139.	1170	C	SER	191	A	<-->	84	O	ASN	281	B	3.76
140.	1170	C	SER	191	A	<-->	81	OD1	ASN	281	B	3.33
141.	1170	C	SER	191	A	<-->	85	N	ASN	282	B	3.03
142.	1170	C	SER	191	A	<-->	86	CA	ASN	282	B	3.84
143.	1170	C	SER	191	A	<-->	87	CB	ASN	282	B	3.52
144.	1170	C	SER	191	A	<-->	88	CG	ASN	282	B	2.98
145.	1170	C	SER	191	A	<-->	89	OD1	ASN	282	B	3.64
146.	1170	C	SER	191	A	<-->	90	ND2	ASN	282	B	2.49
147.	1171	O	SER	191	A	<-->	78	CA	ASN	281	B	3.30
148.	1171	O	SER	191	A	<-->	83	C	ASN	281	B	2.69
149.	1171	O	SER	191	A	<-->	84	O	ASN	281	B	3.39
150.	1171	O	SER	191	A	<-->	85	N	ASN	282	B	2.31
151.	1171	O	SER	191	A	<-->	86	CA	ASN	282	B	2.77

152.	1171	O	SER	191	A	<-->	87	CB	ASN	282	B	2.38
153.	1171	O	SER	191	A	<-->	88	CG	ASN	282	B	1.83
154.	1171	O	SER	191	A	<-->	89	OD1	ASN	282	B	2.55
155.	1171	O	SER	191	A	<-->	90	ND2	ASN	282	B	1.78
156.	1172	CB	SER	191	A	<-->	81	OD1	ASN	281	B	2.95
157.	1172	CB	SER	191	A	<-->	90	ND2	ASN	282	B	3.70
158.	1173	OG	SER	191	A	<-->	81	OD1	ASN	281	B	3.62
159.	1174	N	LEU	192	A	<-->	78	CA	ASN	281	B	3.49
160.	1174	N	LEU	192	A	<-->	83	C	ASN	281	B	3.87
161.	1174	N	LEU	192	A	<-->	85	N	ASN	282	B	3.77
162.	1174	N	LEU	192	A	<-->	90	ND2	ASN	282	B	3.48
163.	1187	CG	LEU	193	A	<-->	231	OG1	THR	301	B	3.48
164.	1187	CG	LEU	193	A	<-->	232	CG2	THR	301	B	3.58
165.	1188	CD1	LEU	193	A	<-->	230	CB	THR	301	B	3.86
166.	1188	CD1	LEU	193	A	<-->	231	OG1	THR	301	B	3.25
167.	1188	CD1	LEU	193	A	<-->	232	CG2	THR	301	B	3.25
168.	1189	CD2	LEU	193	A	<-->	232	CG2	THR	301	B	3.20
169.	1195	CG	MET	194	A	<-->	10	O	SER	270	B	3.71
170.	1198	N	ALA	195	A	<-->	1	N	GLY	269	B	2.78
171.	1198	N	ALA	195	A	<-->	2	CA	GLY	269	B	3.14
172.	1198	N	ALA	195	A	<-->	3	C	GLY	269	B	3.65
173.	1198	N	ALA	195	A	<-->	4	O	GLY	269	B	3.71
174.	1199	CA	ALA	195	A	<-->	1	N	GLY	269	B	2.80
175.	1199	CA	ALA	195	A	<-->	2	CA	GLY	269	B	3.51
176.	1199	CA	ALA	195	A	<-->	3	C	GLY	269	B	3.66
177.	1199	CA	ALA	195	A	<-->	4	O	GLY	269	B	3.24
178.	1200	C	ALA	195	A	<-->	1	N	GLY	269	B	3.66
179.	1200	C	ALA	195	A	<-->	3	C	GLY	269	B	3.50
180.	1200	C	ALA	195	A	<-->	4	O	GLY	269	B	2.90
181.	1201	O	ALA	195	A	<-->	1	N	GLY	269	B	3.58
182.	1201	O	ALA	195	A	<-->	2	CA	GLY	269	B	3.43
183.	1201	O	ALA	195	A	<-->	3	C	GLY	269	B	2.63
184.	1201	O	ALA	195	A	<-->	4	O	GLY	269	B	2.10
185.	1201	O	ALA	195	A	<-->	5	N	SER	270	B	3.33
186.	1201	O	ALA	195	A	<-->	6	CA	SER	270	B	3.55
187.	1201	O	ALA	195	A	<-->	9	C	SER	270	B	3.37
188.	1201	O	ALA	195	A	<-->	10	O	SER	270	B	3.28
189.	1202	CB	ALA	195	A	<-->	1	N	GLY	269	B	1.88
190.	1202	CB	ALA	195	A	<-->	2	CA	GLY	269	B	2.98
191.	1202	CB	ALA	195	A	<-->	3	C	GLY	269	B	3.17
192.	1202	CB	ALA	195	A	<-->	4	O	GLY	269	B	2.61
193.	1208	CG	ARG	196	A	<-->	9	C	SER	270	B	2.99
194.	1208	CG	ARG	196	A	<-->	10	O	SER	270	B	3.11
195.	1208	CG	ARG	196	A	<-->	11	N	LEU	271	B	2.81
196.	1208	CG	ARG	196	A	<-->	12	CA	LEU	271	B	2.72
197.	1208	CG	ARG	196	A	<-->	13	CB	LEU	271	B	3.27
198.	1208	CG	ARG	196	A	<-->	15	CD1	LEU	271	B	3.77
199.	1209	CD	ARG	196	A	<-->	6	CA	SER	270	B	3.79
200.	1209	CD	ARG	196	A	<-->	9	C	SER	270	B	2.36
201.	1209	CD	ARG	196	A	<-->	10	O	SER	270	B	2.16
202.	1209	CD	ARG	196	A	<-->	11	N	LEU	271	B	2.19
203.	1209	CD	ARG	196	A	<-->	12	CA	LEU	271	B	1.71
204.	1209	CD	ARG	196	A	<-->	17	C	LEU	271	B	2.74
205.	1209	CD	ARG	196	A	<-->	18	O	LEU	271	B	3.83
206.	1209	CD	ARG	196	A	<-->	13	CB	LEU	271	B	2.84
207.	1209	CD	ARG	196	A	<-->	14	CG	LEU	271	B	3.64
208.	1209	CD	ARG	196	A	<-->	15	CD1	LEU	271	B	3.40
209.	1209	CD	ARG	196	A	<-->	19	N	HIS	272	B	2.98
210.	1210	NE	ARG	196	A	<-->	6	CA	SER	270	B	2.94
211.	1210	NE	ARG	196	A	<-->	9	C	SER	270	B	1.53

212.	1210	NE	ARG	196	A	<-->	10	O	SER	270	B	2.02
213.	1210	NE	ARG	196	A	<-->	7	CB	SER	270	B	3.66
214.	1210	NE	ARG	196	A	<-->	11	N	LEU	271	B	0.78
215.	1210	NE	ARG	196	A	<-->	12	CA	LEU	271	B	0.93
216.	1210	NE	ARG	196	A	<-->	17	C	LEU	271	B	2.09
217.	1210	NE	ARG	196	A	<-->	18	O	LEU	271	B	2.83
218.	1210	NE	ARG	196	A	<-->	13	CB	LEU	271	B	2.24
219.	1210	NE	ARG	196	A	<-->	14	CG	LEU	271	B	3.47
220.	1210	NE	ARG	196	A	<-->	15	CD1	LEU	271	B	3.86
221.	1210	NE	ARG	196	A	<-->	19	N	HIS	272	B	2.96
222.	1211	CZ	ARG	196	A	<-->	3	C	GLY	269	B	3.62
223.	1211	CZ	ARG	196	A	<-->	5	N	SER	270	B	2.70
224.	1211	CZ	ARG	196	A	<-->	6	CA	SER	270	B	1.66
225.	1211	CZ	ARG	196	A	<-->	9	C	SER	270	B	0.48
226.	1211	CZ	ARG	196	A	<-->	10	O	SER	270	B	1.58
227.	1211	CZ	ARG	196	A	<-->	7	CB	SER	270	B	2.38
228.	1211	CZ	ARG	196	A	<-->	8	OG	SER	270	B	2.79
229.	1211	CZ	ARG	196	A	<-->	11	N	LEU	271	B	0.99
230.	1211	CZ	ARG	196	A	<-->	12	CA	LEU	271	B	2.22
231.	1211	CZ	ARG	196	A	<-->	17	C	LEU	271	B	2.92
232.	1211	CZ	ARG	196	A	<-->	18	O	LEU	271	B	3.29
233.	1211	CZ	ARG	196	A	<-->	13	CB	LEU	271	B	3.41
234.	1211	CZ	ARG	196	A	<-->	19	N	HIS	272	B	3.82
235.	1212	NH1	ARG	196	A	<-->	2	CA	GLY	269	B	3.53
236.	1212	NH1	ARG	196	A	<-->	3	C	GLY	269	B	2.42
237.	1212	NH1	ARG	196	A	<-->	4	O	GLY	269	B	2.95
238.	1212	NH1	ARG	196	A	<-->	5	N	SER	270	B	1.63
239.	1212	NH1	ARG	196	A	<-->	6	CA	SER	270	B	1.40
240.	1212	NH1	ARG	196	A	<-->	9	C	SER	270	B	0.93
241.	1212	NH1	ARG	196	A	<-->	10	O	SER	270	B	1.07
242.	1212	NH1	ARG	196	A	<-->	7	CB	SER	270	B	2.63
243.	1212	NH1	ARG	196	A	<-->	8	OG	SER	270	B	2.94
244.	1212	NH1	ARG	196	A	<-->	11	N	LEU	271	B	2.25
245.	1212	NH1	ARG	196	A	<-->	12	CA	LEU	271	B	3.27
246.	1212	NH1	ARG	196	A	<-->	17	C	LEU	271	B	3.89
247.	1212	NH1	ARG	196	A	<-->	21	ND1	HIS	272	B	3.75
248.	1212	NH1	ARG	196	A	<-->	26	CE1	HIS	272	B	3.68
249.	1213	NH2	ARG	196	A	<-->	5	N	SER	270	B	3.08
250.	1213	NH2	ARG	196	A	<-->	6	CA	SER	270	B	1.79
251.	1213	NH2	ARG	196	A	<-->	9	C	SER	270	B	1.75
252.	1213	NH2	ARG	196	A	<-->	10	O	SER	270	B	2.80
253.	1213	NH2	ARG	196	A	<-->	7	CB	SER	270	B	1.48
254.	1213	NH2	ARG	196	A	<-->	8	OG	SER	270	B	2.08
255.	1213	NH2	ARG	196	A	<-->	11	N	LEU	271	B	1.69
256.	1213	NH2	ARG	196	A	<-->	12	CA	LEU	271	B	2.98
257.	1213	NH2	ARG	196	A	<-->	17	C	LEU	271	B	3.44
258.	1213	NH2	ARG	196	A	<-->	18	O	LEU	271	B	3.31
259.	1213	NH2	ARG	196	A	<-->	13	CB	LEU	271	B	3.80
260.	1239	C	SER	200	A	<-->	70	O	CYS	279	B	3.47
261.	1242	OG	SER	200	A	<-->	73	CB	SER	280	B	3.67
262.	1242	OG	SER	200	A	<-->	74	OG	SER	280	B	3.68
263.	1243	N	ASP	201	A	<-->	69	C	CYS	279	B	3.31
264.	1243	N	ASP	201	A	<-->	70	O	CYS	279	B	2.16
265.	1244	CA	ASP	201	A	<-->	65	N	CYS	279	B	2.97
266.	1244	CA	ASP	201	A	<-->	66	CA	CYS	279	B	3.14
267.	1244	CA	ASP	201	A	<-->	69	C	CYS	279	B	2.41
268.	1244	CA	ASP	201	A	<-->	70	O	CYS	279	B	1.43
269.	1244	CA	ASP	201	A	<-->	67	CB	CYS	279	B	3.67
270.	1244	CA	ASP	201	A	<-->	71	N	SER	280	B	3.44
271.	1244	CA	ASP	201	A	<-->	72	CA	SER	280	B	3.88

272.	1245	C	ASP	201	A	<-->	63	C	THR	278	B	3.53
273.	1245	C	ASP	201	A	<-->	65	N	CYS	279	B	2.53
274.	1245	C	ASP	201	A	<-->	66	CA	CYS	279	B	2.35
275.	1245	C	ASP	201	A	<-->	69	C	CYS	279	B	1.24
276.	1245	C	ASP	201	A	<-->	70	O	CYS	279	B	0.96
277.	1245	C	ASP	201	A	<-->	67	CB	CYS	279	B	3.37
278.	1245	C	ASP	201	A	<-->	71	N	SER	280	B	2.01
279.	1245	C	ASP	201	A	<-->	72	CA	SER	280	B	2.63
280.	1245	C	ASP	201	A	<-->	73	CB	SER	280	B	3.29
281.	1245	C	ASP	201	A	<-->	74	OG	SER	280	B	2.92
282.	1246	O	ASP	201	A	<-->	63	C	THR	278	B	2.98
283.	1246	O	ASP	201	A	<-->	64	O	THR	278	B	3.40
284.	1246	O	ASP	201	A	<-->	65	N	CYS	279	B	1.97
285.	1246	O	ASP	201	A	<-->	66	CA	CYS	279	B	1.23
286.	1246	O	ASP	201	A	<-->	69	C	CYS	279	B	0.45
287.	1246	O	ASP	201	A	<-->	70	O	CYS	279	B	1.41
288.	1246	O	ASP	201	A	<-->	67	CB	CYS	279	B	2.41
289.	1246	O	ASP	201	A	<-->	71	N	SER	280	B	1.54
290.	1246	O	ASP	201	A	<-->	72	CA	SER	280	B	2.76
291.	1246	O	ASP	201	A	<-->	75	C	SER	280	B	3.89
292.	1246	O	ASP	201	A	<-->	73	CB	SER	280	B	3.64
293.	1246	O	ASP	201	A	<-->	74	OG	SER	280	B	3.38
294.	1246	O	ASP	201	A	<-->	100	O	ILE	283	B	3.62
295.	1247	CB	ASP	201	A	<-->	65	N	CYS	279	B	3.12
296.	1247	CB	ASP	201	A	<-->	66	CA	CYS	279	B	3.01
297.	1247	CB	ASP	201	A	<-->	69	C	CYS	279	B	2.70
298.	1247	CB	ASP	201	A	<-->	70	O	CYS	279	B	1.86
299.	1247	CB	ASP	201	A	<-->	67	CB	CYS	279	B	2.84
300.	1248	CG	ASP	201	A	<-->	66	CA	CYS	279	B	3.23
301.	1248	CG	ASP	201	A	<-->	69	C	CYS	279	B	2.65
302.	1248	CG	ASP	201	A	<-->	70	O	CYS	279	B	1.94
303.	1248	CG	ASP	201	A	<-->	67	CB	CYS	279	B	2.83
304.	1248	CG	ASP	201	A	<-->	71	N	SER	280	B	3.67
305.	1249	OD1	ASP	201	A	<-->	66	CA	CYS	279	B	3.62
306.	1249	OD1	ASP	201	A	<-->	69	C	CYS	279	B	2.54
307.	1249	OD1	ASP	201	A	<-->	70	O	CYS	279	B	1.82
308.	1249	OD1	ASP	201	A	<-->	67	CB	CYS	279	B	3.61
309.	1249	OD1	ASP	201	A	<-->	71	N	SER	280	B	3.17
310.	1249	OD1	ASP	201	A	<-->	72	CA	SER	280	B	3.24
311.	1249	OD1	ASP	201	A	<-->	75	C	SER	280	B	3.89
312.	1249	OD1	ASP	201	A	<-->	77	N	ASN	281	B	3.72
313.	1250	OD2	ASP	201	A	<-->	66	CA	CYS	279	B	3.73
314.	1250	OD2	ASP	201	A	<-->	69	C	CYS	279	B	3.53
315.	1250	OD2	ASP	201	A	<-->	70	O	CYS	279	B	3.10
316.	1250	OD2	ASP	201	A	<-->	67	CB	CYS	279	B	2.81
317.	1251	N	GLU	202	A	<-->	65	N	CYS	279	B	3.57
318.	1251	N	GLU	202	A	<-->	66	CA	CYS	279	B	3.45
319.	1251	N	GLU	202	A	<-->	69	C	CYS	279	B	2.20
320.	1251	N	GLU	202	A	<-->	70	O	CYS	279	B	2.05
321.	1251	N	GLU	202	A	<-->	71	N	SER	280	B	2.15
322.	1251	N	GLU	202	A	<-->	72	CA	SER	280	B	2.09
323.	1251	N	GLU	202	A	<-->	75	C	SER	280	B	3.61
324.	1251	N	GLU	202	A	<-->	73	CB	SER	280	B	2.21
325.	1251	N	GLU	202	A	<-->	74	OG	SER	280	B	1.79
326.	1252	CA	GLU	202	A	<-->	69	C	CYS	279	B	2.77
327.	1252	CA	GLU	202	A	<-->	70	O	CYS	279	B	3.13
328.	1252	CA	GLU	202	A	<-->	71	N	SER	280	B	1.97
329.	1252	CA	GLU	202	A	<-->	72	CA	SER	280	B	1.61
330.	1252	CA	GLU	202	A	<-->	75	C	SER	280	B	2.93
331.	1252	CA	GLU	202	A	<-->	76	O	SER	280	B	3.36

332.	1252	CA	GLU	202	A	<-->	73	CB	SER	280	B	0.99
333.	1252	CA	GLU	202	A	<-->	74	OG	SER	280	B	0.83
334.	1253	C	GLU	202	A	<-->	63	C	THR	278	B	3.84
335.	1253	C	GLU	202	A	<-->	64	O	THR	278	B	3.81
336.	1253	C	GLU	202	A	<-->	61	OG1	THR	278	B	3.48
337.	1253	C	GLU	202	A	<-->	65	N	CYS	279	B	3.85
338.	1253	C	GLU	202	A	<-->	66	CA	CYS	279	B	3.82
339.	1253	C	GLU	202	A	<-->	69	C	CYS	279	B	3.21
340.	1253	C	GLU	202	A	<-->	70	O	CYS	279	B	3.86
341.	1253	C	GLU	202	A	<-->	71	N	SER	280	B	2.52
342.	1253	C	GLU	202	A	<-->	72	CA	SER	280	B	2.85
343.	1253	C	GLU	202	A	<-->	73	CB	SER	280	B	2.17
344.	1253	C	GLU	202	A	<-->	74	OG	SER	280	B	1.18
345.	1253	C	GLU	202	A	<-->	100	O	ILE	283	B	3.63
346.	1254	O	GLU	202	A	<-->	64	O	THR	278	B	3.82
347.	1254	O	GLU	202	A	<-->	61	OG1	THR	278	B	3.73
348.	1254	O	GLU	202	A	<-->	69	C	CYS	279	B	3.88
349.	1254	O	GLU	202	A	<-->	71	N	SER	280	B	2.98
350.	1254	O	GLU	202	A	<-->	72	CA	SER	280	B	3.39
351.	1254	O	GLU	202	A	<-->	76	O	SER	280	B	3.80
352.	1254	O	GLU	202	A	<-->	73	CB	SER	280	B	2.73
353.	1254	O	GLU	202	A	<-->	74	OG	SER	280	B	2.19
354.	1254	O	GLU	202	A	<-->	100	O	ILE	283	B	2.99
355.	1255	CB	GLU	202	A	<-->	71	N	SER	280	B	3.41
356.	1255	CB	GLU	202	A	<-->	72	CA	SER	280	B	2.53
357.	1255	CB	GLU	202	A	<-->	75	C	SER	280	B	3.47
358.	1255	CB	GLU	202	A	<-->	76	O	SER	280	B	3.84
359.	1255	CB	GLU	202	A	<-->	73	CB	SER	280	B	1.12
360.	1255	CB	GLU	202	A	<-->	74	OG	SER	280	B	1.36
361.	1256	CG	GLU	202	A	<-->	72	CA	SER	280	B	3.88
362.	1256	CG	GLU	202	A	<-->	73	CB	SER	280	B	2.58
363.	1256	CG	GLU	202	A	<-->	74	OG	SER	280	B	1.95
364.	1257	CD	GLU	202	A	<-->	73	CB	SER	280	B	3.57
365.	1257	CD	GLU	202	A	<-->	74	OG	SER	280	B	3.30
366.	1258	OE1	GLU	202	A	<-->	73	CB	SER	280	B	3.59
367.	1258	OE1	GLU	202	A	<-->	74	OG	SER	280	B	3.69
368.	1260	N	GLY	203	A	<-->	59	CA	THR	278	B	3.62
369.	1260	N	GLY	203	A	<-->	63	C	THR	278	B	3.23
370.	1260	N	GLY	203	A	<-->	64	O	THR	278	B	3.49
371.	1260	N	GLY	203	A	<-->	60	CB	THR	278	B	2.79
372.	1260	N	GLY	203	A	<-->	61	OG1	THR	278	B	2.46
373.	1260	N	GLY	203	A	<-->	65	N	CYS	279	B	3.41
374.	1260	N	GLY	203	A	<-->	66	CA	CYS	279	B	3.88
375.	1260	N	GLY	203	A	<-->	69	C	CYS	279	B	3.52
376.	1260	N	GLY	203	A	<-->	71	N	SER	280	B	3.31
377.	1260	N	GLY	203	A	<-->	72	CA	SER	280	B	3.82
378.	1260	N	GLY	203	A	<-->	73	CB	SER	280	B	3.22
379.	1260	N	GLY	203	A	<-->	74	OG	SER	280	B	1.89
380.	1261	CA	GLY	203	A	<-->	59	CA	THR	278	B	3.12
381.	1261	CA	GLY	203	A	<-->	63	C	THR	278	B	3.13
382.	1261	CA	GLY	203	A	<-->	64	O	THR	278	B	3.24
383.	1261	CA	GLY	203	A	<-->	60	CB	THR	278	B	2.05
384.	1261	CA	GLY	203	A	<-->	61	OG1	THR	278	B	1.17
385.	1261	CA	GLY	203	A	<-->	62	CG2	THR	278	B	3.23
386.	1261	CA	GLY	203	A	<-->	65	N	CYS	279	B	3.82
387.	1261	CA	GLY	203	A	<-->	74	OG	SER	280	B	3.20
388.	1262	C	GLY	203	A	<-->	59	CA	THR	278	B	3.29
389.	1262	C	GLY	203	A	<-->	63	C	THR	278	B	3.87
390.	1262	C	GLY	203	A	<-->	60	CB	THR	278	B	1.80
391.	1262	C	GLY	203	A	<-->	61	OG1	THR	278	B	1.69

392.	1262	C	GLY	203	A	<-->	62	CG2	THR	278	B	2.30
393.	1263	O	GLY	203	A	<-->	60	CB	THR	278	B	2.85
394.	1263	O	GLY	203	A	<-->	61	OG1	THR	278	B	2.54
395.	1263	O	GLY	203	A	<-->	62	CG2	THR	278	B	2.79
396.	1264	N	THR	204	A	<-->	58	N	THR	278	B	3.89
397.	1264	N	THR	204	A	<-->	59	CA	THR	278	B	2.77
398.	1264	N	THR	204	A	<-->	63	C	THR	278	B	3.55
399.	1264	N	THR	204	A	<-->	60	CB	THR	278	B	1.45
400.	1264	N	THR	204	A	<-->	61	OG1	THR	278	B	2.34
401.	1264	N	THR	204	A	<-->	62	CG2	THR	278	B	1.81
402.	1265	CA	THR	204	A	<-->	59	CA	THR	278	B	3.60
403.	1265	CA	THR	204	A	<-->	60	CB	THR	278	B	2.52
404.	1265	CA	THR	204	A	<-->	61	OG1	THR	278	B	3.57
405.	1265	CA	THR	204	A	<-->	62	CG2	THR	278	B	1.91
406.	1266	C	THR	204	A	<-->	57	O	CYS	277	B	3.77
407.	1266	C	THR	204	A	<-->	59	CA	THR	278	B	3.26
408.	1266	C	THR	204	A	<-->	60	CB	THR	278	B	2.82
409.	1266	C	THR	204	A	<-->	62	CG2	THR	278	B	2.28
410.	1267	O	THR	204	A	<-->	57	O	CYS	277	B	3.40
411.	1267	O	THR	204	A	<-->	58	N	THR	278	B	3.77
412.	1267	O	THR	204	A	<-->	59	CA	THR	278	B	2.63
413.	1267	O	THR	204	A	<-->	63	C	THR	278	B	3.55
414.	1267	O	THR	204	A	<-->	60	CB	THR	278	B	2.67
415.	1267	O	THR	204	A	<-->	62	CG2	THR	278	B	2.77
416.	1267	O	THR	204	A	<-->	65	N	CYS	279	B	3.45
417.	1268	CB	THR	204	A	<-->	60	CB	THR	278	B	3.81
418.	1268	CB	THR	204	A	<-->	62	CG2	THR	278	B	3.39
419.	1271	N	TYR	205	A	<-->	57	O	CYS	277	B	3.88
420.	1271	N	TYR	205	A	<-->	62	CG2	THR	278	B	2.99
421.	1272	CA	TYR	205	A	<-->	57	O	CYS	277	B	3.75
422.	1276	CG	TYR	205	A	<-->	46	O	ALA	275	B	3.38
423.	1278	CD2	TYR	205	A	<-->	45	C	ALA	275	B	3.35
424.	1278	CD2	TYR	205	A	<-->	46	O	ALA	275	B	2.13
425.	1278	CD2	TYR	205	A	<-->	57	O	CYS	277	B	3.86
426.	1280	CE2	TYR	205	A	<-->	45	C	ALA	275	B	3.27
427.	1280	CE2	TYR	205	A	<-->	46	O	ALA	275	B	2.15
428.	1280	CE2	TYR	205	A	<-->	47	N	ALA	276	B	3.81
429.	1280	CE2	TYR	205	A	<-->	48	CA	ALA	276	B	3.42
430.	1280	CE2	TYR	205	A	<-->	50	C	ALA	276	B	3.40
431.	1280	CE2	TYR	205	A	<-->	51	O	ALA	276	B	3.43
432.	1281	CZ	TYR	205	A	<-->	46	O	ALA	275	B	3.41
433.	1281	CZ	TYR	205	A	<-->	50	C	ALA	276	B	3.66
434.	1281	CZ	TYR	205	A	<-->	51	O	ALA	276	B	3.21
435.	1281	CZ	TYR	205	A	<-->	142	CE	LYS	289	B	3.75
436.	1282	OH	TYR	205	A	<-->	48	CA	ALA	276	B	3.88
437.	1282	OH	TYR	205	A	<-->	50	C	ALA	276	B	3.52
438.	1282	OH	TYR	205	A	<-->	51	O	ALA	276	B	2.76
439.	1282	OH	TYR	205	A	<-->	141	CD	LYS	289	B	3.31
440.	1282	OH	TYR	205	A	<-->	142	CE	LYS	289	B	2.45
441.	1282	OH	TYR	205	A	<-->	143	NZ	LYS	289	B	3.66
442.	1294	O	CYS	207	A	<-->	31	CB	CYS	273	B	3.27
443.	1298	CA	VAL	208	A	<-->	15	CD1	LEU	271	B	3.36
444.	1298	CA	VAL	208	A	<-->	34	O	CYS	273	B	3.44
445.	1298	CA	VAL	208	A	<-->	31	CB	CYS	273	B	3.16
446.	1298	CA	VAL	208	A	<-->	32	SG	CYS	273	B	3.78
447.	1299	C	VAL	208	A	<-->	14	CG	LEU	271	B	2.94
448.	1299	C	VAL	208	A	<-->	15	CD1	LEU	271	B	2.35
449.	1299	C	VAL	208	A	<-->	16	CD2	LEU	271	B	2.74
450.	1299	C	VAL	208	A	<-->	34	O	CYS	273	B	3.20
451.	1299	C	VAL	208	A	<-->	31	CB	CYS	273	B	3.82

452.	1300	O	VAL	208	A	<-->	14	CG	LEU	271	B	3.25
453.	1300	O	VAL	208	A	<-->	15	CD1	LEU	271	B	2.97
454.	1300	O	VAL	208	A	<-->	16	CD2	LEU	271	B	2.50
455.	1301	CB	VAL	208	A	<-->	33	C	CYS	273	B	3.79
456.	1301	CB	VAL	208	A	<-->	34	O	CYS	273	B	3.22
457.	1301	CB	VAL	208	A	<-->	31	CB	CYS	273	B	3.60
458.	1301	CB	VAL	208	A	<-->	32	SG	CYS	273	B	3.67
459.	1301	CB	VAL	208	A	<-->	40	C	PRO	274	B	3.81
460.	1301	CB	VAL	208	A	<-->	41	O	PRO	274	B	3.43
461.	1301	CB	VAL	208	A	<-->	43	CA	ALA	275	B	3.80
462.	1302	CG1	VAL	208	A	<-->	31	CB	CYS	273	B	3.70
463.	1302	CG1	VAL	208	A	<-->	32	SG	CYS	273	B	3.05
464.	1302	CG1	VAL	208	A	<-->	41	O	PRO	274	B	3.26
465.	1302	CG1	VAL	208	A	<-->	57	O	CYS	277	B	3.13
466.	1303	CG2	VAL	208	A	<-->	43	CA	ALA	275	B	3.46
467.	1304	N	ALA	209	A	<-->	13	CB	LEU	271	B	3.38
468.	1304	N	ALA	209	A	<-->	14	CG	LEU	271	B	1.90
469.	1304	N	ALA	209	A	<-->	15	CD1	LEU	271	B	1.45
470.	1304	N	ALA	209	A	<-->	16	CD2	LEU	271	B	2.32
471.	1304	N	ALA	209	A	<-->	29	N	CYS	273	B	3.32
472.	1304	N	ALA	209	A	<-->	30	CA	CYS	273	B	3.61
473.	1304	N	ALA	209	A	<-->	33	C	CYS	273	B	3.54
474.	1304	N	ALA	209	A	<-->	34	O	CYS	273	B	2.71
475.	1304	N	ALA	209	A	<-->	31	CB	CYS	273	B	3.42
476.	1305	CA	ALA	209	A	<-->	12	CA	LEU	271	B	3.13
477.	1305	CA	ALA	209	A	<-->	17	C	LEU	271	B	3.36
478.	1305	CA	ALA	209	A	<-->	18	O	LEU	271	B	3.87
479.	1305	CA	ALA	209	A	<-->	13	CB	LEU	271	B	2.11
480.	1305	CA	ALA	209	A	<-->	14	CG	LEU	271	B	0.59
481.	1305	CA	ALA	209	A	<-->	15	CD1	LEU	271	B	1.54
482.	1305	CA	ALA	209	A	<-->	16	CD2	LEU	271	B	1.36
483.	1305	CA	ALA	209	A	<-->	19	N	HIS	272	B	3.70
484.	1305	CA	ALA	209	A	<-->	29	N	CYS	273	B	3.70
485.	1305	CA	ALA	209	A	<-->	34	O	CYS	273	B	3.31
486.	1306	C	ALA	209	A	<-->	12	CA	LEU	271	B	2.93
487.	1306	C	ALA	209	A	<-->	17	C	LEU	271	B	2.44
488.	1306	C	ALA	209	A	<-->	18	O	LEU	271	B	2.75
489.	1306	C	ALA	209	A	<-->	13	CB	LEU	271	B	2.47
490.	1306	C	ALA	209	A	<-->	14	CG	LEU	271	B	1.56
491.	1306	C	ALA	209	A	<-->	15	CD1	LEU	271	B	2.48
492.	1306	C	ALA	209	A	<-->	16	CD2	LEU	271	B	2.55
493.	1306	C	ALA	209	A	<-->	19	N	HIS	272	B	2.72
494.	1306	C	ALA	209	A	<-->	20	CA	HIS	272	B	3.34
495.	1306	C	ALA	209	A	<-->	27	C	HIS	272	B	3.46
496.	1306	C	ALA	209	A	<-->	29	N	CYS	273	B	2.76
497.	1306	C	ALA	209	A	<-->	30	CA	CYS	273	B	3.74
498.	1306	C	ALA	209	A	<-->	33	C	CYS	273	B	3.63
499.	1306	C	ALA	209	A	<-->	34	O	CYS	273	B	2.99
500.	1307	O	ALA	209	A	<-->	12	CA	LEU	271	B	3.67
501.	1307	O	ALA	209	A	<-->	17	C	LEU	271	B	2.86
502.	1307	O	ALA	209	A	<-->	18	O	LEU	271	B	3.25
503.	1307	O	ALA	209	A	<-->	13	CB	LEU	271	B	3.55
504.	1307	O	ALA	209	A	<-->	14	CG	LEU	271	B	2.56
505.	1307	O	ALA	209	A	<-->	15	CD1	LEU	271	B	2.89
506.	1307	O	ALA	209	A	<-->	16	CD2	LEU	271	B	3.54
507.	1307	O	ALA	209	A	<-->	19	N	HIS	272	B	2.54
508.	1307	O	ALA	209	A	<-->	20	CA	HIS	272	B	2.74
509.	1307	O	ALA	209	A	<-->	27	C	HIS	272	B	2.40
510.	1307	O	ALA	209	A	<-->	28	O	HIS	272	B	3.47
511.	1307	O	ALA	209	A	<-->	29	N	CYS	273	B	1.57

512.	1307	O	ALA	209	A	<-->	30	CA	CYS	273	B	2.57
513.	1307	O	ALA	209	A	<-->	33	C	CYS	273	B	2.68
514.	1307	O	ALA	209	A	<-->	34	O	CYS	273	B	2.41
515.	1307	O	ALA	209	A	<-->	31	CB	CYS	273	B	3.41
516.	1307	O	ALA	209	A	<-->	35	N	PRO	274	B	3.83
517.	1308	CB	ALA	209	A	<-->	11	N	LEU	271	B	3.32
518.	1308	CB	ALA	209	A	<-->	12	CA	LEU	271	B	2.19
519.	1308	CB	ALA	209	A	<-->	17	C	LEU	271	B	3.10
520.	1308	CB	ALA	209	A	<-->	18	O	LEU	271	B	3.84
521.	1308	CB	ALA	209	A	<-->	13	CB	LEU	271	B	1.17
522.	1308	CB	ALA	209	A	<-->	14	CG	LEU	271	B	1.05
523.	1308	CB	ALA	209	A	<-->	15	CD1	LEU	271	B	1.47
524.	1308	CB	ALA	209	A	<-->	16	CD2	LEU	271	B	2.08
525.	1308	CB	ALA	209	A	<-->	19	N	HIS	272	B	3.64
526.	1309	N	THR	210	A	<-->	11	N	LEU	271	B	3.75
527.	1309	N	THR	210	A	<-->	12	CA	LEU	271	B	2.77
528.	1309	N	THR	210	A	<-->	17	C	LEU	271	B	2.12
529.	1309	N	THR	210	A	<-->	18	O	LEU	271	B	1.82
530.	1309	N	THR	210	A	<-->	13	CB	LEU	271	B	2.43
531.	1309	N	THR	210	A	<-->	14	CG	LEU	271	B	2.28
532.	1309	N	THR	210	A	<-->	15	CD1	LEU	271	B	3.55
533.	1309	N	THR	210	A	<-->	16	CD2	LEU	271	B	3.06
534.	1309	N	THR	210	A	<-->	19	N	HIS	272	B	2.90
535.	1309	N	THR	210	A	<-->	20	CA	HIS	272	B	3.45
536.	1309	N	THR	210	A	<-->	29	N	CYS	273	B	3.68
537.	1310	CA	THR	210	A	<-->	12	CA	LEU	271	B	3.56
538.	1310	CA	THR	210	A	<-->	17	C	LEU	271	B	2.35
539.	1310	CA	THR	210	A	<-->	18	O	LEU	271	B	1.54
540.	1310	CA	THR	210	A	<-->	13	CB	LEU	271	B	3.69
541.	1310	CA	THR	210	A	<-->	14	CG	LEU	271	B	3.64
542.	1310	CA	THR	210	A	<-->	19	N	HIS	272	B	2.90
543.	1310	CA	THR	210	A	<-->	20	CA	HIS	272	B	2.86
544.	1310	CA	THR	210	A	<-->	27	C	HIS	272	B	3.54
545.	1310	CA	THR	210	A	<-->	29	N	CYS	273	B	3.69
546.	1310	CA	THR	210	A	<-->	201	ND2	ASN	297	B	3.11
547.	1311	C	THR	210	A	<-->	11	N	LEU	271	B	3.66
548.	1311	C	THR	210	A	<-->	12	CA	LEU	271	B	3.24
549.	1311	C	THR	210	A	<-->	17	C	LEU	271	B	1.94
550.	1311	C	THR	210	A	<-->	18	O	LEU	271	B	0.90
551.	1311	C	THR	210	A	<-->	19	N	HIS	272	B	2.61
552.	1311	C	THR	210	A	<-->	20	CA	HIS	272	B	2.66
553.	1311	C	THR	210	A	<-->	27	C	HIS	272	B	3.88
554.	1311	C	THR	210	A	<-->	23	CB	HIS	272	B	3.45
555.	1311	C	THR	210	A	<-->	22	CG	HIS	272	B	3.15
556.	1311	C	THR	210	A	<-->	21	ND1	HIS	272	B	3.89
557.	1311	C	THR	210	A	<-->	25	CD2	HIS	272	B	2.99
558.	1311	C	THR	210	A	<-->	24	NE2	HIS	272	B	3.68
559.	1311	C	THR	210	A	<-->	199	CG	ASN	297	B	3.83
560.	1311	C	THR	210	A	<-->	201	ND2	ASN	297	B	2.78
561.	1312	O	THR	210	A	<-->	9	C	SER	270	B	3.50
562.	1312	O	THR	210	A	<-->	10	O	SER	270	B	3.89
563.	1312	O	THR	210	A	<-->	7	CB	SER	270	B	3.69
564.	1312	O	THR	210	A	<-->	8	OG	SER	270	B	3.18
565.	1312	O	THR	210	A	<-->	11	N	LEU	271	B	3.00
566.	1312	O	THR	210	A	<-->	12	CA	LEU	271	B	3.08
567.	1312	O	THR	210	A	<-->	17	C	LEU	271	B	2.28
568.	1312	O	THR	210	A	<-->	18	O	LEU	271	B	1.28
569.	1312	O	THR	210	A	<-->	13	CB	LEU	271	B	3.78
570.	1312	O	THR	210	A	<-->	19	N	HIS	272	B	3.31
571.	1312	O	THR	210	A	<-->	20	CA	HIS	272	B	3.71

572. 1312 O THR 210 A <--> 22 CG HIS 272 B 3.68
573. 1312 O THR 210 A <--> 25 CD2 HIS 272 B 3.36
574. 1312 O THR 210 A <--> 24 NE2 HIS 272 B 3.59
575. 1312 O THR 210 A <--> 201 ND2 ASN 297 B 3.29
576. 1313 CB THR 210 A <--> 17 C LEU 271 B 3.76
577. 1313 CB THR 210 A <--> 18 O LEU 271 B 2.73
578. 1313 CB THR 210 A <--> 199 CG ASN 297 B 3.89
579. 1313 CB THR 210 A <--> 201 ND2 ASN 297 B 2.59
580. 1314 OG1 THR 210 A <--> 18 O LEU 271 B 3.51
581. 1314 OG1 THR 210 A <--> 203 O ASN 297 B 3.77
582. 1314 OG1 THR 210 A <--> 198 CB ASN 297 B 3.10
583. 1314 OG1 THR 210 A <--> 199 CG ASN 297 B 2.67
584. 1314 OG1 THR 210 A <--> 200 OD1 ASN 297 B 3.79
585. 1314 OG1 THR 210 A <--> 201 ND2 ASN 297 B 1.57
586. 1315 CG2 THR 210 A <--> 18 O LEU 271 B 3.01
587. 1315 CG2 THR 210 A <--> 201 ND2 ASN 297 B 3.04
588. 1316 N ASN 211 A <--> 17 C LEU 271 B 2.47
589. 1316 N ASN 211 A <--> 18 O LEU 271 B 2.01
590. 1316 N ASN 211 A <--> 19 N HIS 272 B 2.48
591. 1316 N ASN 211 A <--> 20 CA HIS 272 B 1.98
592. 1316 N ASN 211 A <--> 27 C HIS 272 B 3.35
593. 1316 N ASN 211 A <--> 23 CB HIS 272 B 2.28
594. 1316 N ASN 211 A <--> 22 CG HIS 272 B 1.96
595. 1316 N ASN 211 A <--> 21 ND1 HIS 272 B 2.98
596. 1316 N ASN 211 A <--> 25 CD2 HIS 272 B 1.89
597. 1316 N ASN 211 A <--> 26 CE1 HIS 272 B 3.43
598. 1316 N ASN 211 A <--> 24 NE2 HIS 272 B 2.92
599. 1316 N ASN 211 A <--> 203 O ASN 297 B 3.23
600. 1316 N ASN 211 A <--> 199 CG ASN 297 B 3.42
601. 1316 N ASN 211 A <--> 200 OD1 ASN 297 B 3.65
602. 1316 N ASN 211 A <--> 201 ND2 ASN 297 B 2.81
603. 1317 CA ASN 211 A <--> 10 O SER 270 B 3.88
604. 1317 CA ASN 211 A <--> 8 OG SER 270 B 3.72
605. 1317 CA ASN 211 A <--> 17 C LEU 271 B 3.25
606. 1317 CA ASN 211 A <--> 18 O LEU 271 B 2.96
607. 1317 CA ASN 211 A <--> 19 N HIS 272 B 3.24
608. 1317 CA ASN 211 A <--> 20 CA HIS 272 B 2.92
609. 1317 CA ASN 211 A <--> 23 CB HIS 272 B 2.43
610. 1317 CA ASN 211 A <--> 22 CG HIS 272 B 1.20
611. 1317 CA ASN 211 A <--> 21 ND1 HIS 272 B 1.91
612. 1317 CA ASN 211 A <--> 25 CD2 HIS 272 B 0.78
613. 1317 CA ASN 211 A <--> 26 CE1 HIS 272 B 2.07
614. 1317 CA ASN 211 A <--> 24 NE2 HIS 272 B 1.55
615. 1317 CA ASN 211 A <--> 203 O ASN 297 B 3.70
616. 1317 CA ASN 211 A <--> 199 CG ASN 297 B 3.74
617. 1317 CA ASN 211 A <--> 200 OD1 ASN 297 B 3.47
618. 1317 CA ASN 211 A <--> 201 ND2 ASN 297 B 3.48
619. 1318 C ASN 211 A <--> 20 CA HIS 272 B 3.52
620. 1318 C ASN 211 A <--> 23 CB HIS 272 B 2.60
621. 1318 C ASN 211 A <--> 22 CG HIS 272 B 1.59
622. 1318 C ASN 211 A <--> 21 ND1 HIS 272 B 2.54
623. 1318 C ASN 211 A <--> 25 CD2 HIS 272 B 0.76
624. 1318 C ASN 211 A <--> 26 CE1 HIS 272 B 2.64
625. 1318 C ASN 211 A <--> 24 NE2 HIS 272 B 1.83
626. 1318 C ASN 211 A <--> 202 C ASN 297 B 3.79
627. 1318 C ASN 211 A <--> 203 O ASN 297 B 2.88
628. 1318 C ASN 211 A <--> 199 CG ASN 297 B 3.29
629. 1318 C ASN 211 A <--> 200 OD1 ASN 297 B 2.76
630. 1318 C ASN 211 A <--> 201 ND2 ASN 297 B 3.59
631. 1318 C ASN 211 A <--> 211 O LEU 298 B 3.88

632.	1319	O	ASN	211	A	<-->	20	CA	HIS	272	B	3.32
633.	1319	O	ASN	211	A	<-->	23	CB	HIS	272	B	2.51
634.	1319	O	ASN	211	A	<-->	22	CG	HIS	272	B	2.23
635.	1319	O	ASN	211	A	<-->	21	ND1	HIS	272	B	3.46
636.	1319	O	ASN	211	A	<-->	25	CD2	HIS	272	B	1.82
637.	1319	O	ASN	211	A	<-->	26	CE1	HIS	272	B	3.81
638.	1319	O	ASN	211	A	<-->	24	NE2	HIS	272	B	3.05
639.	1319	O	ASN	211	A	<-->	197	CA	ASN	297	B	3.48
640.	1319	O	ASN	211	A	<-->	202	C	ASN	297	B	2.62
641.	1319	O	ASN	211	A	<-->	203	O	ASN	297	B	1.70
642.	1319	O	ASN	211	A	<-->	198	CB	ASN	297	B	3.69
643.	1319	O	ASN	211	A	<-->	199	CG	ASN	297	B	2.94
644.	1319	O	ASN	211	A	<-->	200	OD1	ASN	297	B	2.71
645.	1319	O	ASN	211	A	<-->	201	ND2	ASN	297	B	3.42
646.	1319	O	ASN	211	A	<-->	204	N	LEU	298	B	3.52
647.	1319	O	ASN	211	A	<-->	205	CA	LEU	298	B	3.84
648.	1319	O	ASN	211	A	<-->	210	C	LEU	298	B	3.39
649.	1319	O	ASN	211	A	<-->	211	O	LEU	298	B	3.03
650.	1320	CB	ASN	211	A	<-->	9	C	SER	270	B	3.66
651.	1320	CB	ASN	211	A	<-->	10	O	SER	270	B	2.87
652.	1320	CB	ASN	211	A	<-->	8	OG	SER	270	B	3.85
653.	1320	CB	ASN	211	A	<-->	17	C	LEU	271	B	3.26
654.	1320	CB	ASN	211	A	<-->	18	O	LEU	271	B	3.53
655.	1320	CB	ASN	211	A	<-->	19	N	HIS	272	B	2.99
656.	1320	CB	ASN	211	A	<-->	20	CA	HIS	272	B	3.06
657.	1320	CB	ASN	211	A	<-->	23	CB	HIS	272	B	2.27
658.	1320	CB	ASN	211	A	<-->	22	CG	HIS	272	B	1.13
659.	1320	CB	ASN	211	A	<-->	21	ND1	HIS	272	B	0.57
660.	1320	CB	ASN	211	A	<-->	25	CD2	HIS	272	B	1.98
661.	1320	CB	ASN	211	A	<-->	26	CE1	HIS	272	B	1.55
662.	1320	CB	ASN	211	A	<-->	24	NE2	HIS	272	B	2.13
663.	1321	CG	ASN	211	A	<-->	5	N	SER	270	B	3.79
664.	1321	CG	ASN	211	A	<-->	9	C	SER	270	B	3.74
665.	1321	CG	ASN	211	A	<-->	10	O	SER	270	B	2.95
666.	1321	CG	ASN	211	A	<-->	8	OG	SER	270	B	3.39
667.	1321	CG	ASN	211	A	<-->	23	CB	HIS	272	B	3.69
668.	1321	CG	ASN	211	A	<-->	22	CG	HIS	272	B	2.39
669.	1321	CG	ASN	211	A	<-->	21	ND1	HIS	272	B	1.17
670.	1321	CG	ASN	211	A	<-->	25	CD2	HIS	272	B	2.70
671.	1321	CG	ASN	211	A	<-->	26	CE1	HIS	272	B	0.67
672.	1321	CG	ASN	211	A	<-->	24	NE2	HIS	272	B	1.99
673.	1322	OD1	ASN	211	A	<-->	5	N	SER	270	B	3.67
674.	1322	OD1	ASN	211	A	<-->	10	O	SER	270	B	3.07
675.	1322	OD1	ASN	211	A	<-->	22	CG	HIS	272	B	3.30
676.	1322	OD1	ASN	211	A	<-->	21	ND1	HIS	272	B	1.95
677.	1322	OD1	ASN	211	A	<-->	25	CD2	HIS	272	B	3.85
678.	1322	OD1	ASN	211	A	<-->	26	CE1	HIS	272	B	1.86
679.	1322	OD1	ASN	211	A	<-->	24	NE2	HIS	272	B	3.17
680.	1323	ND2	ASN	211	A	<-->	5	N	SER	270	B	3.88
681.	1323	ND2	ASN	211	A	<-->	10	O	SER	270	B	3.72
682.	1323	ND2	ASN	211	A	<-->	8	OG	SER	270	B	2.72
683.	1323	ND2	ASN	211	A	<-->	22	CG	HIS	272	B	3.06
684.	1323	ND2	ASN	211	A	<-->	21	ND1	HIS	272	B	2.27
685.	1323	ND2	ASN	211	A	<-->	25	CD2	HIS	272	B	2.71
686.	1323	ND2	ASN	211	A	<-->	26	CE1	HIS	272	B	0.95
687.	1323	ND2	ASN	211	A	<-->	24	NE2	HIS	272	B	1.45
688.	1324	N	SER	212	A	<-->	23	CB	HIS	272	B	3.69
689.	1324	N	SER	212	A	<-->	22	CG	HIS	272	B	2.51
690.	1324	N	SER	212	A	<-->	21	ND1	HIS	272	B	2.87
691.	1324	N	SER	212	A	<-->	25	CD2	HIS	272	B	1.72

692.	1324	N	SER	212	A	<-->	26	CE1	HIS	272	B	2.50
693.	1324	N	SER	212	A	<-->	24	NE2	HIS	272	B	1.73
694.	1324	N	SER	212	A	<-->	203	O	ASN	297	B	3.88
695.	1324	N	SER	212	A	<-->	200	OD1	ASN	297	B	3.07
696.	1325	CA	SER	212	A	<-->	22	CG	HIS	272	B	3.66
697.	1325	CA	SER	212	A	<-->	25	CD2	HIS	272	B	3.01
698.	1325	CA	SER	212	A	<-->	26	CE1	HIS	272	B	3.81
699.	1325	CA	SER	212	A	<-->	24	NE2	HIS	272	B	3.14
700.	1325	CA	SER	212	A	<-->	200	OD1	ASN	297	B	3.42
701.	1325	CA	SER	212	A	<-->	211	O	LEU	298	B	3.49
702.	1326	C	SER	212	A	<-->	24	NE2	HIS	272	B	3.81
703.	1327	O	SER	212	A	<-->	26	CE1	HIS	272	B	3.50
704.	1327	O	SER	212	A	<-->	24	NE2	HIS	272	B	3.59
705.	1328	CB	SER	212	A	<-->	24	NE2	HIS	272	B	3.89
706.	1328	CB	SER	212	A	<-->	200	OD1	ASN	297	B	3.30
707.	1329	OG	SER	212	A	<-->	197	CA	ASN	297	B	3.66
708.	1329	OG	SER	212	A	<-->	199	CG	ASN	297	B	3.37
709.	1329	OG	SER	212	A	<-->	200	OD1	ASN	297	B	2.24
710.	1330	N	ALA	213	A	<-->	211	O	LEU	298	B	3.71
711.	1330	N	ALA	213	A	<-->	219	N	GLU	300	B	3.12
712.	1330	N	ALA	213	A	<-->	220	CA	GLU	300	B	3.41
713.	1330	N	ALA	213	A	<-->	221	CB	GLU	300	B	2.90
714.	1330	N	ALA	213	A	<-->	222	CG	GLU	300	B	3.46
715.	1331	CA	ALA	213	A	<-->	219	N	GLU	300	B	2.97
716.	1331	CA	ALA	213	A	<-->	220	CA	GLU	300	B	2.92
717.	1331	CA	ALA	213	A	<-->	221	CB	GLU	300	B	1.91
718.	1331	CA	ALA	213	A	<-->	222	CG	GLU	300	B	2.11
719.	1331	CA	ALA	213	A	<-->	223	CD	GLU	300	B	2.69
720.	1331	CA	ALA	213	A	<-->	224	OE1	GLU	300	B	2.93
721.	1331	CA	ALA	213	A	<-->	225	OE2	GLU	300	B	3.63
722.	1332	C	ALA	213	A	<-->	217	C	PRO	299	B	3.85
723.	1332	C	ALA	213	A	<-->	219	N	GLU	300	B	2.62
724.	1332	C	ALA	213	A	<-->	220	CA	GLU	300	B	1.86
725.	1332	C	ALA	213	A	<-->	226	C	GLU	300	B	3.08
726.	1332	C	ALA	213	A	<-->	227	O	GLU	300	B	3.65
727.	1332	C	ALA	213	A	<-->	221	CB	GLU	300	B	0.65
728.	1332	C	ALA	213	A	<-->	222	CG	GLU	300	B	1.87
729.	1332	C	ALA	213	A	<-->	223	CD	GLU	300	B	2.72
730.	1332	C	ALA	213	A	<-->	224	OE1	GLU	300	B	2.81
731.	1332	C	ALA	213	A	<-->	225	OE2	GLU	300	B	3.90
732.	1333	O	ALA	213	A	<-->	219	N	GLU	300	B	3.07
733.	1333	O	ALA	213	A	<-->	220	CA	GLU	300	B	2.15
734.	1333	O	ALA	213	A	<-->	226	C	GLU	300	B	3.40
735.	1333	O	ALA	213	A	<-->	227	O	GLU	300	B	3.72
736.	1333	O	ALA	213	A	<-->	221	CB	GLU	300	B	1.63
737.	1333	O	ALA	213	A	<-->	222	CG	GLU	300	B	3.06
738.	1333	O	ALA	213	A	<-->	223	CD	GLU	300	B	3.77
739.	1333	O	ALA	213	A	<-->	224	OE1	GLU	300	B	3.54
740.	1334	CB	ALA	213	A	<-->	219	N	GLU	300	B	2.76
741.	1334	CB	ALA	213	A	<-->	220	CA	GLU	300	B	3.13
742.	1334	CB	ALA	213	A	<-->	221	CB	GLU	300	B	2.47
743.	1334	CB	ALA	213	A	<-->	222	CG	GLU	300	B	1.82
744.	1334	CB	ALA	213	A	<-->	223	CD	GLU	300	B	2.69
745.	1334	CB	ALA	213	A	<-->	224	OE1	GLU	300	B	3.51
746.	1334	CB	ALA	213	A	<-->	225	OE2	GLU	300	B	3.21
747.	1335	N	GLY	214	A	<-->	219	N	GLU	300	B	2.80
748.	1335	N	GLY	214	A	<-->	220	CA	GLU	300	B	1.79
749.	1335	N	GLY	214	A	<-->	226	C	GLU	300	B	2.32
750.	1335	N	GLY	214	A	<-->	227	O	GLU	300	B	2.84
751.	1335	N	GLY	214	A	<-->	221	CB	GLU	300	B	0.73

752.	1335	N	GLY	214	A	<-->	222	CG	GLU	300	B	1.17
753.	1335	N	GLY	214	A	<-->	223	CD	GLU	300	B	2.26
754.	1335	N	GLY	214	A	<-->	224	OE1	GLU	300	B	2.70
755.	1335	N	GLY	214	A	<-->	225	OE2	GLU	300	B	3.31
756.	1335	N	GLY	214	A	<-->	228	N	THR	301	B	3.12
757.	1336	CA	GLY	214	A	<-->	219	N	GLU	300	B	3.48
758.	1336	CA	GLY	214	A	<-->	220	CA	GLU	300	B	2.11
759.	1336	CA	GLY	214	A	<-->	226	C	GLU	300	B	1.83
760.	1336	CA	GLY	214	A	<-->	227	O	GLU	300	B	1.70
761.	1336	CA	GLY	214	A	<-->	221	CB	GLU	300	B	1.86
762.	1336	CA	GLY	214	A	<-->	222	CG	GLU	300	B	2.46
763.	1336	CA	GLY	214	A	<-->	223	CD	GLU	300	B	3.29
764.	1336	CA	GLY	214	A	<-->	224	OE1	GLU	300	B	3.51
765.	1336	CA	GLY	214	A	<-->	228	N	THR	301	B	2.90
766.	1336	CA	GLY	214	A	<-->	229	CA	THR	301	B	3.76
767.	1337	C	GLY	214	A	<-->	220	CA	GLU	300	B	3.56
768.	1337	C	GLY	214	A	<-->	226	C	GLU	300	B	3.22
769.	1337	C	GLY	214	A	<-->	227	O	GLU	300	B	2.77
770.	1337	C	GLY	214	A	<-->	221	CB	GLU	300	B	2.85
771.	1337	C	GLY	214	A	<-->	222	CG	GLU	300	B	3.07
772.	1337	C	GLY	214	A	<-->	223	CD	GLU	300	B	3.20
773.	1337	C	GLY	214	A	<-->	224	OE1	GLU	300	B	3.14
774.	1338	O	GLY	214	A	<-->	226	C	GLU	300	B	3.80
775.	1338	O	GLY	214	A	<-->	227	O	GLU	300	B	3.01
776.	1339	N	HIS	215	A	<-->	221	CB	GLU	300	B	2.97
777.	1339	N	HIS	215	A	<-->	222	CG	GLU	300	B	2.86
778.	1339	N	HIS	215	A	<-->	223	CD	GLU	300	B	2.33
779.	1339	N	HIS	215	A	<-->	224	OE1	GLU	300	B	2.02
780.	1339	N	HIS	215	A	<-->	225	OE2	GLU	300	B	3.13
781.	1340	CA	HIS	215	A	<-->	223	CD	GLU	300	B	3.21
782.	1340	CA	HIS	215	A	<-->	224	OE1	GLU	300	B	2.70
783.	1340	CA	HIS	215	A	<-->	225	OE2	GLU	300	B	3.64
784.	1341	C	HIS	215	A	<-->	223	CD	GLU	300	B	3.52
785.	1341	C	HIS	215	A	<-->	224	OE1	GLU	300	B	3.47
786.	1341	C	HIS	215	A	<-->	225	OE2	GLU	300	B	3.46
787.	1342	O	HIS	215	A	<-->	222	CG	GLU	300	B	3.87
788.	1342	O	HIS	215	A	<-->	223	CD	GLU	300	B	3.00
789.	1342	O	HIS	215	A	<-->	224	OE1	GLU	300	B	3.38
790.	1342	O	HIS	215	A	<-->	225	OE2	GLU	300	B	2.61
791.	1343	CB	HIS	215	A	<-->	223	CD	GLU	300	B	3.45
792.	1343	CB	HIS	215	A	<-->	224	OE1	GLU	300	B	2.57
793.	1343	CB	HIS	215	A	<-->	225	OE2	GLU	300	B	3.78
794.	1353	CB	ARG	216	A	<-->	425	NZ	LYS	325	B	3.32
795.	1354	CG	ARG	216	A	<-->	424	CE	LYS	325	B	3.31
796.	1354	CG	ARG	216	A	<-->	425	NZ	LYS	325	B	2.20
797.	1355	CD	ARG	216	A	<-->	423	CD	LYS	325	B	3.18
798.	1355	CD	ARG	216	A	<-->	424	CE	LYS	325	B	2.12
799.	1355	CD	ARG	216	A	<-->	425	NZ	LYS	325	B	0.83
800.	1356	NE	ARG	216	A	<-->	422	CG	LYS	325	B	3.09
801.	1356	NE	ARG	216	A	<-->	423	CD	LYS	325	B	2.05
802.	1356	NE	ARG	216	A	<-->	424	CE	LYS	325	B	0.94
803.	1356	NE	ARG	216	A	<-->	425	NZ	LYS	325	B	1.19
804.	1357	CZ	ARG	216	A	<-->	421	CB	LYS	325	B	3.36
805.	1357	CZ	ARG	216	A	<-->	422	CG	LYS	325	B	2.16
806.	1357	CZ	ARG	216	A	<-->	423	CD	LYS	325	B	1.06
807.	1357	CZ	ARG	216	A	<-->	424	CE	LYS	325	B	0.56
808.	1357	CZ	ARG	216	A	<-->	425	NZ	LYS	325	B	1.75
809.	1358	NH1	ARG	216	A	<-->	421	CB	LYS	325	B	3.78
810.	1358	NH1	ARG	216	A	<-->	422	CG	LYS	325	B	3.02
811.	1358	NH1	ARG	216	A	<-->	423	CD	LYS	325	B	1.82

812.	1358	NH1	ARG	216	A	<-->	424	CE	LYS	325	B	1.71
813.	1358	NH1	ARG	216	A	<-->	425	NZ	LYS	325	B	1.95
814.	1359	NH2	ARG	216	A	<-->	420	CA	LYS	325	B	3.42
815.	1359	NH2	ARG	216	A	<-->	421	CB	LYS	325	B	2.38
816.	1359	NH2	ARG	216	A	<-->	422	CG	LYS	325	B	0.97
817.	1359	NH2	ARG	216	A	<-->	423	CD	LYS	325	B	1.21
818.	1359	NH2	ARG	216	A	<-->	424	CE	LYS	325	B	1.65
819.	1359	NH2	ARG	216	A	<-->	425	NZ	LYS	325	B	3.08
820.	1392	CA	ALA	221	A	<-->	603	CB	ARG	348	B	3.80
821.	1392	CA	ALA	221	A	<-->	615	OG	SER	349	B	2.62
822.	1393	C	ALA	221	A	<-->	614	CB	SER	349	B	3.80
823.	1393	C	ALA	221	A	<-->	615	OG	SER	349	B	2.39
824.	1394	O	ALA	221	A	<-->	615	OG	SER	349	B	3.56
825.	1395	CB	ALA	221	A	<-->	601	N	ARG	348	B	3.39
826.	1395	CB	ALA	221	A	<-->	602	CA	ARG	348	B	2.85
827.	1395	CB	ALA	221	A	<-->	610	C	ARG	348	B	2.65
828.	1395	CB	ALA	221	A	<-->	611	O	ARG	348	B	2.99
829.	1395	CB	ALA	221	A	<-->	603	CB	ARG	348	B	2.35
830.	1395	CB	ALA	221	A	<-->	604	CG	ARG	348	B	3.71
831.	1395	CB	ALA	221	A	<-->	612	N	SER	349	B	3.10
832.	1395	CB	ALA	221	A	<-->	613	CA	SER	349	B	3.81
833.	1395	CB	ALA	221	A	<-->	614	CB	SER	349	B	3.84
834.	1395	CB	ALA	221	A	<-->	615	OG	SER	349	B	2.82
835.	1396	N	ARG	222	A	<-->	613	CA	SER	349	B	3.48
836.	1396	N	ARG	222	A	<-->	614	CB	SER	349	B	2.74
837.	1396	N	ARG	222	A	<-->	615	OG	SER	349	B	1.50
838.	1397	CA	ARG	222	A	<-->	614	CB	SER	349	B	3.31
839.	1397	CA	ARG	222	A	<-->	615	OG	SER	349	B	2.54
840.	1398	C	ARG	222	A	<-->	615	OG	SER	349	B	3.50
841.	1399	O	ARG	222	A	<-->	615	OG	SER	349	B	3.83
842.	1400	CB	ARG	222	A	<-->	615	OG	SER	349	B	3.71
843.	1411	CB	VAL	223	A	<-->	443	NH1	ARG	327	B	3.37
844.	1412	CG1	VAL	223	A	<-->	442	CZ	ARG	327	B	3.30
845.	1412	CG1	VAL	223	A	<-->	443	NH1	ARG	327	B	2.82
846.	1412	CG1	VAL	223	A	<-->	444	NH2	ARG	327	B	3.27
847.	1413	CG2	VAL	223	A	<-->	443	NH1	ARG	327	B	2.80

Number of hydrogen bonds: 11

Number of non-bonded contacts: 847

Table S6. The residue-specific interaction between the cognate full proteins, ROBO1/4 and SLIT2. The interaction files are ordered following the order of the complexes in Fig 3.

[A] ROBO1 (WT) + SLIT2 (WT) FULL PROTEIN

Hydrogen bonds

	<----- A T O M 1 ----->						<----- A T O M 2 ----->					
	Atom no.	Atom name	Res name	Res no.	Chain		Atom no.	Atom name	Res name	Res no.	Chain	Distance
1.	442	OH	TYR	56	A	<-->	14032	O	ILE	166	B	3.35
2.	459	OG	SER	59	A	<-->	14040	O	GLU	167	B	2.98
3.	460	N	ARG	60	A	<-->	14032	O	ILE	166	B	2.55
4.	744	N	THR	96	A	<-->	15837	OD1	ASP	394	B	3.27
5.	749	OG1	THR	96	A	<-->	15834	O	ASP	394	B	2.79
6.	754	O	PRO	97	A	<-->	15856	NE2	HIS	396	B	2.66
7.	899	OD1	ASP	114	A	<-->	16993	NH2	ARG	539	B	2.61
8.	917	NH1	ARG	116	A	<-->	16997	O	LEU	540	B	2.51
9.	924	OG	SER	117	A	<-->	15863	OD1	ASN	397	B	3.34
10.	924	OG	SER	117	A	<-->	17018	N	ASN	543	B	2.92
11.	1997	OE2	GLU	255	A	<-->	13124	NH2	ARG	55	B	1.61
12.	2298	NH2	ARG	294	A	<-->	13803	OD1	ASN	137	B	3.31

Non-bonded contacts

	<----- A T O M 1 ----->						<----- A T O M 2 ----->					
	Atom no.	Atom name	Res name	Res no.	Chain		Atom no.	Atom name	Res name	Res no.	Chain	Distance
1.	437	CD1	TYR	56	A	<-->	14036	CD1	ILE	166	B	3.85
2.	439	CE1	TYR	56	A	<-->	14036	CD1	ILE	166	B	3.42
3.	441	CZ	TYR	56	A	<-->	14033	CB	ILE	166	B	3.75
4.	441	CZ	TYR	56	A	<-->	14036	CD1	ILE	166	B	3.55
5.	442	OH	TYR	56	A	<-->	14029	N	ILE	166	B	3.40
6.	442	OH	TYR	56	A	<-->	14030	CA	ILE	166	B	3.75
7.	442	OH	TYR	56	A	<-->	14032	O	ILE	166	B	3.35
8.	442	OH	TYR	56	A	<-->	14033	CB	ILE	166	B	3.27
9.	442	OH	TYR	56	A	<-->	14034	CG1	ILE	166	B	3.75
10.	450	N	GLY	58	A	<-->	14219	CZ	ARG	189	B	3.78
11.	450	N	GLY	58	A	<-->	14220	NH1	ARG	189	B	3.86
12.	450	N	GLY	58	A	<-->	14221	NH2	ARG	189	B	3.46
13.	451	CA	GLY	58	A	<-->	14220	NH1	ARG	189	B	3.68
14.	455	CA	SER	59	A	<-->	14032	O	ILE	166	B	3.07
15.	455	CA	SER	59	A	<-->	14040	O	GLU	167	B	3.31
16.	455	CA	SER	59	A	<-->	14050	CB	ASP	168	B	3.59
17.	456	C	SER	59	A	<-->	14032	O	ILE	166	B	3.21
18.	456	C	SER	59	A	<-->	14039	C	GLU	167	B	3.54
19.	456	C	SER	59	A	<-->	14040	O	GLU	167	B	2.72
20.	457	O	SER	59	A	<-->	14040	O	GLU	167	B	3.05
21.	458	CB	SER	59	A	<-->	14032	O	ILE	166	B	3.53
22.	458	CB	SER	59	A	<-->	14039	C	GLU	167	B	3.47
23.	458	CB	SER	59	A	<-->	14040	O	GLU	167	B	2.88
24.	458	CB	SER	59	A	<-->	14046	N	ASP	168	B	3.68
25.	458	CB	SER	59	A	<-->	14047	CA	ASP	168	B	3.40
26.	458	CB	SER	59	A	<-->	14050	CB	ASP	168	B	2.15

27.	458	CB	SER	59	A	<-->	14051	CG	ASP	168	B	3.16
28.	458	CB	SER	59	A	<-->	14053	OD2	ASP	168	B	3.25
29.	459	OG	SER	59	A	<-->	14040	O	GLU	167	B	2.98
30.	459	OG	SER	59	A	<-->	14047	CA	ASP	168	B	3.66
31.	459	OG	SER	59	A	<-->	14048	C	ASP	168	B	3.80
32.	459	OG	SER	59	A	<-->	14049	O	ASP	168	B	3.48
33.	459	OG	SER	59	A	<-->	14050	CB	ASP	168	B	2.51
34.	459	OG	SER	59	A	<-->	14051	CG	ASP	168	B	3.17
35.	459	OG	SER	59	A	<-->	14053	OD2	ASP	168	B	3.24
36.	459	OG	SER	59	A	<-->	14215	CB	ARG	189	B	3.49
37.	459	OG	SER	59	A	<-->	14216	CG	ARG	189	B	3.27
38.	459	OG	SER	59	A	<-->	14217	CD	ARG	189	B	3.14
39.	459	OG	SER	59	A	<-->	14218	NE	ARG	189	B	3.80
40.	460	N	ARG	60	A	<-->	14031	C	ILE	166	B	3.50
41.	460	N	ARG	60	A	<-->	14032	O	ILE	166	B	2.55
42.	460	N	ARG	60	A	<-->	14037	N	GLU	167	B	3.82
43.	460	N	ARG	60	A	<-->	14038	CA	GLU	167	B	3.25
44.	460	N	ARG	60	A	<-->	14039	C	GLU	167	B	3.25
45.	460	N	ARG	60	A	<-->	14040	O	GLU	167	B	2.76
46.	461	CA	ARG	60	A	<-->	14032	O	ILE	166	B	3.62
47.	461	CA	ARG	60	A	<-->	14038	CA	GLU	167	B	3.36
48.	461	CA	ARG	60	A	<-->	14039	C	GLU	167	B	3.67
49.	461	CA	ARG	60	A	<-->	14040	O	GLU	167	B	3.16
50.	461	CA	ARG	60	A	<-->	14043	CD	GLU	167	B	3.36
51.	461	CA	ARG	60	A	<-->	14044	OE1	GLU	167	B	2.99
52.	461	CA	ARG	60	A	<-->	14045	OE2	GLU	167	B	3.79
53.	462	C	ARG	60	A	<-->	14031	C	ILE	166	B	3.84
54.	462	C	ARG	60	A	<-->	14032	O	ILE	166	B	3.61
55.	462	C	ARG	60	A	<-->	14037	N	GLU	167	B	3.37
56.	462	C	ARG	60	A	<-->	14038	CA	GLU	167	B	2.31
57.	462	C	ARG	60	A	<-->	14039	C	GLU	167	B	3.03
58.	462	C	ARG	60	A	<-->	14040	O	GLU	167	B	2.91
59.	462	C	ARG	60	A	<-->	14041	CB	GLU	167	B	2.89
60.	462	C	ARG	60	A	<-->	14042	CG	GLU	167	B	2.79
61.	462	C	ARG	60	A	<-->	14043	CD	GLU	167	B	2.08
62.	462	C	ARG	60	A	<-->	14044	OE1	GLU	167	B	1.65
63.	462	C	ARG	60	A	<-->	14045	OE2	GLU	167	B	2.86
64.	463	O	ARG	60	A	<-->	14031	C	ILE	166	B	3.02
65.	463	O	ARG	60	A	<-->	14032	O	ILE	166	B	3.11
66.	463	O	ARG	60	A	<-->	14037	N	GLU	167	B	2.46
67.	463	O	ARG	60	A	<-->	14038	CA	GLU	167	B	1.81
68.	463	O	ARG	60	A	<-->	14039	C	GLU	167	B	3.02
69.	463	O	ARG	60	A	<-->	14040	O	GLU	167	B	3.36
70.	463	O	ARG	60	A	<-->	14041	CB	GLU	167	B	2.49
71.	463	O	ARG	60	A	<-->	14042	CG	GLU	167	B	2.99
72.	463	O	ARG	60	A	<-->	14043	CD	GLU	167	B	2.52
73.	463	O	ARG	60	A	<-->	14044	OE1	GLU	167	B	1.65
74.	463	O	ARG	60	A	<-->	14045	OE2	GLU	167	B	3.59
75.	464	CB	ARG	60	A	<-->	14044	OE1	GLU	167	B	3.35
76.	471	N	LEU	61	A	<-->	14038	CA	GLU	167	B	2.80
77.	471	N	LEU	61	A	<-->	14039	C	GLU	167	B	3.27
78.	471	N	LEU	61	A	<-->	14040	O	GLU	167	B	3.03
79.	471	N	LEU	61	A	<-->	14041	CB	GLU	167	B	2.74
80.	471	N	LEU	61	A	<-->	14042	CG	GLU	167	B	1.94
81.	471	N	LEU	61	A	<-->	14043	CD	GLU	167	B	1.14
82.	471	N	LEU	61	A	<-->	14044	OE1	GLU	167	B	1.67
83.	471	N	LEU	61	A	<-->	14045	OE2	GLU	167	B	1.69
84.	472	CA	LEU	61	A	<-->	14038	CA	GLU	167	B	2.95
85.	472	CA	LEU	61	A	<-->	14039	C	GLU	167	B	3.64
86.	472	CA	LEU	61	A	<-->	14040	O	GLU	167	B	3.77

87.	472	CA	LEU	61	A	<-->	14041	CB	GLU	167	B	2.12
88.	472	CA	LEU	61	A	<-->	14042	CG	GLU	167	B	1.00
89.	472	CA	LEU	61	A	<-->	14043	CD	GLU	167	B	0.57
90.	472	CA	LEU	61	A	<-->	14044	OE1	GLU	167	B	1.66
91.	472	CA	LEU	61	A	<-->	14045	OE2	GLU	167	B	1.54
92.	473	C	LEU	61	A	<-->	14041	CB	GLU	167	B	3.48
93.	473	C	LEU	61	A	<-->	14042	CG	GLU	167	B	2.37
94.	473	C	LEU	61	A	<-->	14043	CD	GLU	167	B	1.33
95.	473	C	LEU	61	A	<-->	14044	OE1	GLU	167	B	2.13
96.	473	C	LEU	61	A	<-->	14045	OE2	GLU	167	B	1.01
97.	474	O	LEU	61	A	<-->	14042	CG	GLU	167	B	3.07
98.	474	O	LEU	61	A	<-->	14043	CD	GLU	167	B	2.28
99.	474	O	LEU	61	A	<-->	14044	OE1	GLU	167	B	3.25
100.	474	O	LEU	61	A	<-->	14045	OE2	GLU	167	B	1.26
101.	475	CB	LEU	61	A	<-->	14038	CA	GLU	167	B	3.46
102.	475	CB	LEU	61	A	<-->	14039	C	GLU	167	B	3.65
103.	475	CB	LEU	61	A	<-->	14040	O	GLU	167	B	3.76
104.	475	CB	LEU	61	A	<-->	14041	CB	GLU	167	B	2.40
105.	475	CB	LEU	61	A	<-->	14042	CG	GLU	167	B	1.00
106.	475	CB	LEU	61	A	<-->	14043	CD	GLU	167	B	2.06
107.	475	CB	LEU	61	A	<-->	14044	OE1	GLU	167	B	3.18
108.	475	CB	LEU	61	A	<-->	14045	OE2	GLU	167	B	2.43
109.	475	CB	LEU	61	A	<-->	14054	N	GLY	169	B	3.32
110.	475	CB	LEU	61	A	<-->	14055	CA	GLY	169	B	3.07
111.	475	CB	LEU	61	A	<-->	14056	C	GLY	169	B	3.60
112.	475	CB	LEU	61	A	<-->	14057	O	GLY	169	B	3.51
113.	476	CG	LEU	61	A	<-->	13960	CG	GLN	157	B	3.53
114.	476	CG	LEU	61	A	<-->	14041	CB	GLU	167	B	2.89
115.	476	CG	LEU	61	A	<-->	14042	CG	GLU	167	B	2.08
116.	476	CG	LEU	61	A	<-->	14043	CD	GLU	167	B	3.17
117.	476	CG	LEU	61	A	<-->	14045	OE2	GLU	167	B	3.56
118.	476	CG	LEU	61	A	<-->	14054	N	GLY	169	B	3.76
119.	476	CG	LEU	61	A	<-->	14055	CA	GLY	169	B	3.39
120.	476	CG	LEU	61	A	<-->	14056	C	GLY	169	B	3.23
121.	476	CG	LEU	61	A	<-->	14057	O	GLY	169	B	2.76
122.	477	CD1	LEU	61	A	<-->	14042	CG	GLU	167	B	3.28
123.	477	CD1	LEU	61	A	<-->	14054	N	GLY	169	B	3.47
124.	477	CD1	LEU	61	A	<-->	14055	CA	GLY	169	B	2.69
125.	477	CD1	LEU	61	A	<-->	14056	C	GLY	169	B	2.03
126.	477	CD1	LEU	61	A	<-->	14057	O	GLY	169	B	1.78
127.	477	CD1	LEU	61	A	<-->	14058	N	ALA	170	B	2.82
128.	477	CD1	LEU	61	A	<-->	14059	CA	ALA	170	B	3.36
129.	477	CD1	LEU	61	A	<-->	14060	C	ALA	170	B	3.61
130.	478	CD2	LEU	61	A	<-->	14041	CB	GLU	167	B	3.88
131.	478	CD2	LEU	61	A	<-->	14042	CG	GLU	167	B	3.02
132.	478	CD2	LEU	61	A	<-->	14043	CD	GLU	167	B	3.51
133.	478	CD2	LEU	61	A	<-->	14045	OE2	GLU	167	B	3.61
134.	479	N	ARG	62	A	<-->	14042	CG	GLU	167	B	3.31
135.	479	N	ARG	62	A	<-->	14043	CD	GLU	167	B	2.16
136.	479	N	ARG	62	A	<-->	14044	OE1	GLU	167	B	2.19
137.	479	N	ARG	62	A	<-->	14045	OE2	GLU	167	B	2.16
138.	480	CA	ARG	62	A	<-->	14043	CD	GLU	167	B	3.47
139.	480	CA	ARG	62	A	<-->	14044	OE1	GLU	167	B	3.51
140.	480	CA	ARG	62	A	<-->	14045	OE2	GLU	167	B	3.06
141.	486	NE	ARG	62	A	<-->	14022	OG	SER	164	B	3.81
142.	487	CZ	ARG	62	A	<-->	14021	CB	SER	164	B	3.69
143.	487	CZ	ARG	62	A	<-->	14022	OG	SER	164	B	3.00
144.	488	NH1	ARG	62	A	<-->	14022	OG	SER	164	B	3.71
145.	489	NH2	ARG	62	A	<-->	14018	CA	SER	164	B	3.86
146.	489	NH2	ARG	62	A	<-->	14021	CB	SER	164	B	2.41

147.	489	NH2	ARG	62	A	<-->	14022	OG	SER	164	B	2.10
148.	496	CD	GLN	63	A	<-->	14067	CB	PHE	171	B	3.82
149.	496	CD	GLN	63	A	<-->	14068	CG	PHE	171	B	3.76
150.	496	CD	GLN	63	A	<-->	14069	CD1	PHE	171	B	3.51
151.	497	OE1	GLN	63	A	<-->	14069	CD1	PHE	171	B	3.63
152.	498	NE2	GLN	63	A	<-->	14064	CA	PHE	171	B	3.80
153.	498	NE2	GLN	63	A	<-->	14067	CB	PHE	171	B	2.50
154.	498	NE2	GLN	63	A	<-->	14068	CG	PHE	171	B	2.65
155.	498	NE2	GLN	63	A	<-->	14069	CD1	PHE	171	B	2.62
156.	498	NE2	GLN	63	A	<-->	14070	CD2	PHE	171	B	3.72
157.	498	NE2	GLN	63	A	<-->	14071	CE1	PHE	171	B	3.67
158.	734	CZ	ARG	94	A	<-->	14073	CZ	PHE	171	B	3.57
159.	736	NH2	ARG	94	A	<-->	14071	CE1	PHE	171	B	3.43
160.	736	NH2	ARG	94	A	<-->	14072	CE2	PHE	171	B	3.28
161.	736	NH2	ARG	94	A	<-->	14073	CZ	PHE	171	B	2.61
162.	738	CA	PRO	95	A	<-->	15837	OD1	ASP	394	B	3.73
163.	741	CB	PRO	95	A	<-->	15818	CD2	PHE	392	B	3.68
164.	741	CB	PRO	95	A	<-->	15837	OD1	ASP	394	B	3.06
165.	744	N	THR	96	A	<-->	15837	OD1	ASP	394	B	3.27
166.	747	O	THR	96	A	<-->	15820	CE2	PHE	392	B	3.70
167.	748	CB	THR	96	A	<-->	15855	CE1	HIS	396	B	3.41
168.	749	OG1	THR	96	A	<-->	15820	CE2	PHE	392	B	3.90
169.	749	OG1	THR	96	A	<-->	15832	CA	ASP	394	B	3.56
170.	749	OG1	THR	96	A	<-->	15833	C	ASP	394	B	3.57
171.	749	OG1	THR	96	A	<-->	15834	O	ASP	394	B	2.79
172.	749	OG1	THR	96	A	<-->	15835	CB	ASP	394	B	3.76
173.	749	OG1	THR	96	A	<-->	15837	OD1	ASP	394	B	3.60
174.	749	OG1	THR	96	A	<-->	15853	ND1	HIS	396	B	3.87
175.	749	OG1	THR	96	A	<-->	15855	CE1	HIS	396	B	3.24
176.	749	OG1	THR	96	A	<-->	15856	NE2	HIS	396	B	3.73
177.	753	C	PRO	97	A	<-->	15856	NE2	HIS	396	B	3.50
178.	754	O	PRO	97	A	<-->	15855	CE1	HIS	396	B	3.07
179.	754	O	PRO	97	A	<-->	15856	NE2	HIS	396	B	2.66
180.	759	CA	THR	98	A	<-->	15856	NE2	HIS	396	B	3.77
181.	759	CA	THR	98	A	<-->	16210	CZ3	TRP	440	B	3.45
182.	759	CA	THR	98	A	<-->	16211	CH2	TRP	440	B	3.77
183.	760	C	THR	98	A	<-->	16210	CZ3	TRP	440	B	3.70
184.	760	C	THR	98	A	<-->	16211	CH2	TRP	440	B	3.48
185.	762	CB	THR	98	A	<-->	16208	CE3	TRP	440	B	3.23
186.	762	CB	THR	98	A	<-->	16210	CZ3	TRP	440	B	2.18
187.	762	CB	THR	98	A	<-->	16211	CH2	TRP	440	B	2.92
188.	762	CB	THR	98	A	<-->	17039	CB	PHE	545	B	3.63
189.	762	CB	THR	98	A	<-->	17049	O	THR	546	B	3.68
190.	762	CB	THR	98	A	<-->	17059	CG2	VAL	547	B	2.84
191.	763	OG1	THR	98	A	<-->	16205	CD2	TRP	440	B	3.48
192.	763	OG1	THR	98	A	<-->	16207	CE2	TRP	440	B	3.79
193.	763	OG1	THR	98	A	<-->	16208	CE3	TRP	440	B	2.47
194.	763	OG1	THR	98	A	<-->	16209	CZ2	TRP	440	B	3.25
195.	763	OG1	THR	98	A	<-->	16210	CZ3	TRP	440	B	1.42
196.	763	OG1	THR	98	A	<-->	16211	CH2	TRP	440	B	2.05
197.	763	OG1	THR	98	A	<-->	17036	CA	PHE	545	B	3.67
198.	763	OG1	THR	98	A	<-->	17037	C	PHE	545	B	3.89
199.	763	OG1	THR	98	A	<-->	17039	CB	PHE	545	B	2.42
200.	763	OG1	THR	98	A	<-->	17040	CG	PHE	545	B	3.04
201.	763	OG1	THR	98	A	<-->	17042	CD2	PHE	545	B	3.11
202.	763	OG1	THR	98	A	<-->	17059	CG2	VAL	547	B	3.51
203.	764	CG2	THR	98	A	<-->	16208	CE3	TRP	440	B	3.31
204.	764	CG2	THR	98	A	<-->	16210	CZ3	TRP	440	B	2.28
205.	764	CG2	THR	98	A	<-->	16211	CH2	TRP	440	B	2.86
206.	764	CG2	THR	98	A	<-->	16218	CD1	LEU	441	B	3.73

207.	764	CG2	THR	98	A	<-->	17057	CB	VAL	547	B	3.76
208.	764	CG2	THR	98	A	<-->	17059	CG2	VAL	547	B	2.47
209.	765	N	ILE	99	A	<-->	16210	CZ3	TRP	440	B	3.86
210.	765	N	ILE	99	A	<-->	16211	CH2	TRP	440	B	3.27
211.	765	N	ILE	99	A	<-->	17042	CD2	PHE	545	B	3.63
212.	766	CA	ILE	99	A	<-->	16211	CH2	TRP	440	B	3.84
213.	767	C	ILE	99	A	<-->	16209	CZ2	TRP	440	B	3.66
214.	767	C	ILE	99	A	<-->	16211	CH2	TRP	440	B	3.37
215.	768	O	ILE	99	A	<-->	16209	CZ2	TRP	440	B	3.02
216.	768	O	ILE	99	A	<-->	16211	CH2	TRP	440	B	2.97
217.	777	CB	GLU	100	A	<-->	16209	CZ2	TRP	440	B	3.53
218.	779	CD	GLU	100	A	<-->	16216	CB	LEU	441	B	3.70
219.	779	CD	GLU	100	A	<-->	16219	CD2	LEU	441	B	3.87
220.	780	OE1	GLU	100	A	<-->	16216	CB	LEU	441	B	3.30
221.	780	OE1	GLU	100	A	<-->	16223	O	ALA	442	B	3.48
222.	781	OE2	GLU	100	A	<-->	16216	CB	LEU	441	B	3.36
223.	781	OE2	GLU	100	A	<-->	16217	CG	LEU	441	B	3.61
224.	781	OE2	GLU	100	A	<-->	16219	CD2	LEU	441	B	2.79
225.	837	O	ARG	107	A	<-->	16226	CA	ASP	443	B	3.84
226.	837	O	ARG	107	A	<-->	16227	C	ASP	443	B	3.66
227.	837	O	ARG	107	A	<-->	16233	N	TYR	444	B	3.12
228.	837	O	ARG	107	A	<-->	16234	CA	TYR	444	B	3.74
229.	837	O	ARG	107	A	<-->	16237	CB	TYR	444	B	3.17
230.	838	CB	ARG	107	A	<-->	16222	C	ALA	442	B	3.68
231.	838	CB	ARG	107	A	<-->	16223	O	ALA	442	B	2.83
232.	838	CB	ARG	107	A	<-->	16225	N	ASP	443	B	3.80
233.	838	CB	ARG	107	A	<-->	16226	CA	ASP	443	B	3.10
234.	838	CB	ARG	107	A	<-->	16229	CB	ASP	443	B	3.83
235.	839	CG	ARG	107	A	<-->	16223	O	ALA	442	B	3.29
236.	839	CG	ARG	107	A	<-->	16225	N	ASP	443	B	3.86
237.	839	CG	ARG	107	A	<-->	16226	CA	ASP	443	B	3.07
238.	839	CG	ARG	107	A	<-->	16229	CB	ASP	443	B	3.15
239.	840	CD	ARG	107	A	<-->	16215	O	LEU	441	B	3.80
240.	840	CD	ARG	107	A	<-->	16222	C	ALA	442	B	3.08
241.	840	CD	ARG	107	A	<-->	16223	O	ALA	442	B	2.74
242.	840	CD	ARG	107	A	<-->	16225	N	ASP	443	B	2.91
243.	840	CD	ARG	107	A	<-->	16226	CA	ASP	443	B	2.48
244.	840	CD	ARG	107	A	<-->	16229	CB	ASP	443	B	2.38
245.	840	CD	ARG	107	A	<-->	16230	CG	ASP	443	B	3.74
246.	841	NE	ARG	107	A	<-->	16206	NE1	TRP	440	B	3.00
247.	841	NE	ARG	107	A	<-->	16207	CE2	TRP	440	B	3.72
248.	841	NE	ARG	107	A	<-->	16209	CZ2	TRP	440	B	3.81
249.	841	NE	ARG	107	A	<-->	16226	CA	ASP	443	B	3.73
250.	841	NE	ARG	107	A	<-->	16229	CB	ASP	443	B	3.07
251.	842	CZ	ARG	107	A	<-->	16206	NE1	TRP	440	B	3.30
252.	842	CZ	ARG	107	A	<-->	16229	CB	ASP	443	B	3.05
253.	843	NH1	ARG	107	A	<-->	16226	CA	ASP	443	B	3.72
254.	843	NH1	ARG	107	A	<-->	16229	CB	ASP	443	B	2.36
255.	843	NH1	ARG	107	A	<-->	16230	CG	ASP	443	B	3.16
256.	843	NH1	ARG	107	A	<-->	16232	OD2	ASP	443	B	3.10
257.	844	NH2	ARG	107	A	<-->	16206	NE1	TRP	440	B	3.19
258.	844	NH2	ARG	107	A	<-->	16207	CE2	TRP	440	B	3.77
259.	844	NH2	ARG	107	A	<-->	16209	CZ2	TRP	440	B	3.77
260.	852	N	GLU	109	A	<-->	16236	O	TYR	444	B	3.59
261.	853	CA	GLU	109	A	<-->	16236	O	TYR	444	B	3.76
262.	855	O	GLU	109	A	<-->	16236	O	TYR	444	B	3.76
263.	856	CB	GLU	109	A	<-->	16236	O	TYR	444	B	3.01
264.	857	CG	GLU	109	A	<-->	16235	C	TYR	444	B	2.91
265.	857	CG	GLU	109	A	<-->	16236	O	TYR	444	B	1.71
266.	857	CG	GLU	109	A	<-->	16245	N	LEU	445	B	3.72

267.	857	CG	GLU	109	A	<-->	16246	CA	LEU	445	B	3.75
268.	857	CG	GLU	109	A	<-->	16249	CB	LEU	445	B	2.98
269.	857	CG	GLU	109	A	<-->	16250	CG	LEU	445	B	3.51
270.	857	CG	GLU	109	A	<-->	16251	CD1	LEU	445	B	3.46
271.	858	CD	GLU	109	A	<-->	16235	C	TYR	444	B	3.76
272.	858	CD	GLU	109	A	<-->	16236	O	TYR	444	B	2.69
273.	858	CD	GLU	109	A	<-->	16246	CA	LEU	445	B	3.64
274.	858	CD	GLU	109	A	<-->	16249	CB	LEU	445	B	2.65
275.	858	CD	GLU	109	A	<-->	16250	CG	LEU	445	B	3.54
276.	859	OE1	GLU	109	A	<-->	16236	O	TYR	444	B	3.86
277.	859	OE1	GLU	109	A	<-->	16249	CB	LEU	445	B	3.65
278.	860	OE2	GLU	109	A	<-->	16235	C	TYR	444	B	3.59
279.	860	OE2	GLU	109	A	<-->	16236	O	TYR	444	B	2.81
280.	860	OE2	GLU	109	A	<-->	16245	N	LEU	445	B	3.63
281.	860	OE2	GLU	109	A	<-->	16246	CA	LEU	445	B	2.79
282.	860	OE2	GLU	109	A	<-->	16249	CB	LEU	445	B	2.06
283.	860	OE2	GLU	109	A	<-->	16250	CG	LEU	445	B	3.38
284.	888	O	ASP	113	A	<-->	16993	NH2	ARG	539	B	3.74
285.	894	CA	ASP	114	A	<-->	16993	NH2	ARG	539	B	3.85
286.	898	CG	ASP	114	A	<-->	16993	NH2	ARG	539	B	3.31
287.	899	OD1	ASP	114	A	<-->	16990	NE	ARG	539	B	3.52
288.	899	OD1	ASP	114	A	<-->	16991	CZ	ARG	539	B	3.19
289.	899	OD1	ASP	114	A	<-->	16993	NH2	ARG	539	B	2.61
290.	900	OD2	ASP	114	A	<-->	17001	CD2	LEU	540	B	3.46
291.	904	O	PRO	115	A	<-->	17009	ND2	ASN	541	B	3.67
292.	915	NE	ARG	116	A	<-->	17017	ND2	ASN	542	B	3.63
293.	916	CZ	ARG	116	A	<-->	16997	O	LEU	540	B	3.82
294.	916	CZ	ARG	116	A	<-->	17015	CG	ASN	542	B	3.38
295.	916	CZ	ARG	116	A	<-->	17017	ND2	ASN	542	B	2.46
296.	917	NH1	ARG	116	A	<-->	16995	CA	LEU	540	B	3.83
297.	917	NH1	ARG	116	A	<-->	16996	C	LEU	540	B	3.31
298.	917	NH1	ARG	116	A	<-->	16997	O	LEU	540	B	2.51
299.	917	NH1	ARG	116	A	<-->	16998	CB	LEU	540	B	3.57
300.	917	NH1	ARG	116	A	<-->	17015	CG	ASN	542	B	3.16
301.	917	NH1	ARG	116	A	<-->	17016	OD1	ASN	542	B	3.46
302.	917	NH1	ARG	116	A	<-->	17017	ND2	ASN	542	B	2.76
303.	918	NH2	ARG	116	A	<-->	17015	CG	ASN	542	B	2.99
304.	918	NH2	ARG	116	A	<-->	17016	OD1	ASN	542	B	3.68
305.	918	NH2	ARG	116	A	<-->	17017	ND2	ASN	542	B	1.72
306.	920	CA	SER	117	A	<-->	17025	ND2	ASN	543	B	3.56
307.	923	CB	SER	117	A	<-->	17022	CB	ASN	543	B	3.36
308.	923	CB	SER	117	A	<-->	17023	CG	ASN	543	B	2.40
309.	923	CB	SER	117	A	<-->	17024	OD1	ASN	543	B	2.67
310.	923	CB	SER	117	A	<-->	17025	ND2	ASN	543	B	2.18
311.	924	OG	SER	117	A	<-->	15863	OD1	ASN	397	B	3.34
312.	924	OG	SER	117	A	<-->	17012	C	ASN	542	B	3.58
313.	924	OG	SER	117	A	<-->	17014	CB	ASN	542	B	3.32
314.	924	OG	SER	117	A	<-->	17018	N	ASN	543	B	2.92
315.	924	OG	SER	117	A	<-->	17019	CA	ASN	543	B	3.39
316.	924	OG	SER	117	A	<-->	17022	CB	ASN	543	B	2.81
317.	924	OG	SER	117	A	<-->	17023	CG	ASN	543	B	2.47
318.	924	OG	SER	117	A	<-->	17024	OD1	ASN	543	B	2.81
319.	924	OG	SER	117	A	<-->	17025	ND2	ASN	543	B	2.82
320.	927	C	HIS	118	A	<-->	15862	CG	ASN	397	B	3.11
321.	927	C	HIS	118	A	<-->	15863	OD1	ASN	397	B	3.02
322.	927	C	HIS	118	A	<-->	15864	ND2	ASN	397	B	2.43
323.	928	O	HIS	118	A	<-->	15861	CB	ASN	397	B	3.82
324.	928	O	HIS	118	A	<-->	15862	CG	ASN	397	B	2.30
325.	928	O	HIS	118	A	<-->	15863	OD1	ASN	397	B	1.96
326.	928	O	HIS	118	A	<-->	15864	ND2	ASN	397	B	2.10

327.	935	N	ARG	119	A	<-->	15862	CG	ASN	397	B	3.23
328.	935	N	ARG	119	A	<-->	15863	OD1	ASN	397	B	3.57
329.	935	N	ARG	119	A	<-->	15864	ND2	ASN	397	B	2.19
330.	936	CA	ARG	119	A	<-->	15861	CB	ASN	397	B	3.39
331.	936	CA	ARG	119	A	<-->	15862	CG	ASN	397	B	2.48
332.	936	CA	ARG	119	A	<-->	15863	OD1	ASN	397	B	3.24
333.	936	CA	ARG	119	A	<-->	15864	ND2	ASN	397	B	1.39
334.	937	C	ARG	119	A	<-->	15861	CB	ASN	397	B	2.96
335.	937	C	ARG	119	A	<-->	15862	CG	ASN	397	B	2.19
336.	937	C	ARG	119	A	<-->	15863	OD1	ASN	397	B	3.17
337.	937	C	ARG	119	A	<-->	15864	ND2	ASN	397	B	0.94
338.	938	O	ARG	119	A	<-->	15857	N	ASN	397	B	3.77
339.	938	O	ARG	119	A	<-->	15858	CA	ASN	397	B	3.38
340.	938	O	ARG	119	A	<-->	15861	CB	ASN	397	B	1.99
341.	938	O	ARG	119	A	<-->	15862	CG	ASN	397	B	1.78
342.	938	O	ARG	119	A	<-->	15863	OD1	ASN	397	B	2.94
343.	938	O	ARG	119	A	<-->	15864	ND2	ASN	397	B	1.35
344.	938	O	ARG	119	A	<-->	15872	CD2	LEU	398	B	3.42
345.	939	CB	ARG	119	A	<-->	15862	CG	ASN	397	B	3.76
346.	939	CB	ARG	119	A	<-->	15864	ND2	ASN	397	B	2.74
347.	940	CG	ARG	119	A	<-->	15861	CB	ASN	397	B	3.89
348.	940	CG	ARG	119	A	<-->	15864	ND2	ASN	397	B	3.31
349.	940	CG	ARG	119	A	<-->	15872	CD2	LEU	398	B	3.34
350.	941	CD	ARG	119	A	<-->	14393	OD1	ASN	210	B	3.66
351.	942	NE	ARG	119	A	<-->	14392	CG	ASN	210	B	3.81
352.	942	NE	ARG	119	A	<-->	14393	OD1	ASN	210	B	3.04
353.	943	CZ	ARG	119	A	<-->	14386	ND2	ASN	209	B	3.72
354.	943	CZ	ARG	119	A	<-->	14392	CG	ASN	210	B	3.13
355.	943	CZ	ARG	119	A	<-->	14393	OD1	ASN	210	B	2.08
356.	943	CZ	ARG	119	A	<-->	14394	ND2	ASN	210	B	3.73
357.	944	NH1	ARG	119	A	<-->	14384	CG	ASN	209	B	3.06
358.	944	NH1	ARG	119	A	<-->	14385	OD1	ASN	209	B	2.93
359.	944	NH1	ARG	119	A	<-->	14386	ND2	ASN	209	B	2.43
360.	944	NH1	ARG	119	A	<-->	14392	CG	ASN	210	B	2.91
361.	944	NH1	ARG	119	A	<-->	14393	OD1	ASN	210	B	1.75
362.	944	NH1	ARG	119	A	<-->	14394	ND2	ASN	210	B	3.60
363.	944	NH1	ARG	119	A	<-->	15870	CG	LEU	398	B	3.72
364.	944	NH1	ARG	119	A	<-->	15872	CD2	LEU	398	B	3.26
365.	945	NH2	ARG	119	A	<-->	14392	CG	ASN	210	B	3.44
366.	945	NH2	ARG	119	A	<-->	14393	OD1	ASN	210	B	2.50
367.	945	NH2	ARG	119	A	<-->	15174	OD1	ASN	310	B	3.82
368.	946	N	MET	120	A	<-->	15862	CG	ASN	397	B	3.31
369.	946	N	MET	120	A	<-->	15864	ND2	ASN	397	B	2.08
370.	947	CA	MET	120	A	<-->	15864	ND2	ASN	397	B	3.08
371.	948	C	MET	120	A	<-->	15862	CG	ASN	397	B	3.71
372.	948	C	MET	120	A	<-->	15864	ND2	ASN	397	B	3.22
373.	949	O	MET	120	A	<-->	15862	CG	ASN	397	B	3.31
374.	949	O	MET	120	A	<-->	15863	OD1	ASN	397	B	3.43
375.	949	O	MET	120	A	<-->	15864	ND2	ASN	397	B	2.97
376.	955	CA	LEU	121	A	<-->	15851	CB	HIS	396	B	3.80
377.	955	CA	LEU	121	A	<-->	15852	CG	HIS	396	B	3.70
378.	955	CA	LEU	121	A	<-->	15853	ND1	HIS	396	B	2.82
379.	955	CA	LEU	121	A	<-->	15855	CE1	HIS	396	B	3.87
380.	956	C	LEU	121	A	<-->	15852	CG	HIS	396	B	3.64
381.	956	C	LEU	121	A	<-->	15853	ND1	HIS	396	B	2.59
382.	956	C	LEU	121	A	<-->	15855	CE1	HIS	396	B	3.28
383.	957	O	LEU	121	A	<-->	15851	CB	HIS	396	B	3.75
384.	957	O	LEU	121	A	<-->	15852	CG	HIS	396	B	2.88
385.	957	O	LEU	121	A	<-->	15853	ND1	HIS	396	B	1.79
386.	957	O	LEU	121	A	<-->	15854	CD2	HIS	396	B	3.57

387.	957	O	LEU	121	A	<-->	15855	CE1	HIS	396	B	2.18
388.	957	O	LEU	121	A	<-->	15856	NE2	HIS	396	B	3.27
389.	958	CB	LEU	121	A	<-->	15853	ND1	HIS	396	B	3.04
390.	958	CB	LEU	121	A	<-->	15855	CE1	HIS	396	B	3.82
391.	959	CG	LEU	121	A	<-->	15834	O	ASP	394	B	3.68
392.	959	CG	LEU	121	A	<-->	15840	CA	LEU	395	B	3.47
393.	959	CG	LEU	121	A	<-->	15845	CD1	LEU	395	B	3.43
394.	959	CG	LEU	121	A	<-->	15847	N	HIS	396	B	3.56
395.	960	CD1	LEU	121	A	<-->	15833	C	ASP	394	B	3.55
396.	960	CD1	LEU	121	A	<-->	15834	O	ASP	394	B	3.34
397.	960	CD1	LEU	121	A	<-->	15835	CB	ASP	394	B	3.83
398.	960	CD1	LEU	121	A	<-->	15839	N	LEU	395	B	3.72
399.	960	CD1	LEU	121	A	<-->	15840	CA	LEU	395	B	3.41
400.	960	CD1	LEU	121	A	<-->	15845	CD1	LEU	395	B	2.70
401.	961	CD2	LEU	121	A	<-->	15833	C	ASP	394	B	3.84
402.	961	CD2	LEU	121	A	<-->	15834	O	ASP	394	B	3.35
403.	961	CD2	LEU	121	A	<-->	15839	N	LEU	395	B	3.59
404.	961	CD2	LEU	121	A	<-->	15840	CA	LEU	395	B	2.44
405.	961	CD2	LEU	121	A	<-->	15841	C	LEU	395	B	2.70
406.	961	CD2	LEU	121	A	<-->	15842	O	LEU	395	B	3.89
407.	961	CD2	LEU	121	A	<-->	15843	CB	LEU	395	B	3.11
408.	961	CD2	LEU	121	A	<-->	15844	CG	LEU	395	B	3.88
409.	961	CD2	LEU	121	A	<-->	15845	CD1	LEU	395	B	3.44
410.	961	CD2	LEU	121	A	<-->	15847	N	HIS	396	B	2.23
411.	961	CD2	LEU	121	A	<-->	15848	CA	HIS	396	B	3.43
412.	961	CD2	LEU	121	A	<-->	15857	N	ASN	397	B	3.62
413.	962	N	LEU	122	A	<-->	15853	ND1	HIS	396	B	3.88
414.	1150	CD2	TYR	145	A	<-->	16219	CD2	LEU	441	B	3.88
415.	1995	CD	GLU	255	A	<-->	13124	NH2	ARG	55	B	2.82
416.	1996	OE1	GLU	255	A	<-->	13124	NH2	ARG	55	B	3.50
417.	1997	OE2	GLU	255	A	<-->	13121	NE	ARG	55	B	3.69
418.	1997	OE2	GLU	255	A	<-->	13122	CZ	ARG	55	B	2.95
419.	1997	OE2	GLU	255	A	<-->	13124	NH2	ARG	55	B	1.61
420.	2243	C	GLY	287	A	<-->	13303	CD1	LEU	78	B	3.65
421.	2245	N	ASP	288	A	<-->	13294	CA	GLY	77	B	3.61
422.	2245	N	ASP	288	A	<-->	13295	C	GLY	77	B	3.43
423.	2245	N	ASP	288	A	<-->	13296	O	GLY	77	B	3.41
424.	2245	N	ASP	288	A	<-->	13303	CD1	LEU	78	B	2.54
425.	2246	CA	ASP	288	A	<-->	13294	CA	GLY	77	B	3.56
426.	2246	CA	ASP	288	A	<-->	13295	C	GLY	77	B	3.00
427.	2246	CA	ASP	288	A	<-->	13296	O	GLY	77	B	3.20
428.	2246	CA	ASP	288	A	<-->	13297	N	LEU	78	B	3.21
429.	2246	CA	ASP	288	A	<-->	13298	CA	LEU	78	B	3.52
430.	2246	CA	ASP	288	A	<-->	13301	CB	LEU	78	B	3.75
431.	2246	CA	ASP	288	A	<-->	13302	CG	LEU	78	B	3.11
432.	2246	CA	ASP	288	A	<-->	13303	CD1	LEU	78	B	1.87
433.	2247	C	ASP	288	A	<-->	13293	N	GLY	77	B	3.60
434.	2247	C	ASP	288	A	<-->	13294	CA	GLY	77	B	3.56
435.	2247	C	ASP	288	A	<-->	13295	C	GLY	77	B	3.37
436.	2247	C	ASP	288	A	<-->	13297	N	LEU	78	B	3.26
437.	2247	C	ASP	288	A	<-->	13298	CA	LEU	78	B	3.79
438.	2247	C	ASP	288	A	<-->	13303	CD1	LEU	78	B	3.25
439.	2248	O	ASP	288	A	<-->	13303	CD1	LEU	78	B	3.87
440.	2249	CB	ASP	288	A	<-->	13293	N	GLY	77	B	3.72
441.	2249	CB	ASP	288	A	<-->	13294	CA	GLY	77	B	3.02
442.	2249	CB	ASP	288	A	<-->	13295	C	GLY	77	B	1.89
443.	2249	CB	ASP	288	A	<-->	13296	O	GLY	77	B	1.99
444.	2249	CB	ASP	288	A	<-->	13297	N	LEU	78	B	2.01
445.	2249	CB	ASP	288	A	<-->	13298	CA	LEU	78	B	2.19
446.	2249	CB	ASP	288	A	<-->	13299	C	LEU	78	B	3.67

447.	2249	CB	ASP	288	A	<-->	13301	CB	LEU	78	B	2.35
448.	2249	CB	ASP	288	A	<-->	13302	CG	LEU	78	B	2.19
449.	2249	CB	ASP	288	A	<-->	13303	CD1	LEU	78	B	1.47
450.	2249	CB	ASP	288	A	<-->	13304	CD2	LEU	78	B	3.60
451.	2250	CG	ASP	288	A	<-->	13295	C	GLY	77	B	2.72
452.	2250	CG	ASP	288	A	<-->	13296	O	GLY	77	B	2.05
453.	2250	CG	ASP	288	A	<-->	13297	N	LEU	78	B	3.06
454.	2250	CG	ASP	288	A	<-->	13298	CA	LEU	78	B	2.82
455.	2250	CG	ASP	288	A	<-->	13301	CB	LEU	78	B	2.00
456.	2250	CG	ASP	288	A	<-->	13302	CG	LEU	78	B	1.44
457.	2250	CG	ASP	288	A	<-->	13303	CD1	LEU	78	B	0.97
458.	2250	CG	ASP	288	A	<-->	13304	CD2	LEU	78	B	2.38
459.	2250	CG	ASP	288	A	<-->	13786	CB	SER	135	B	3.41
460.	2251	OD1	ASP	288	A	<-->	13295	C	GLY	77	B	3.89
461.	2251	OD1	ASP	288	A	<-->	13296	O	GLY	77	B	3.20
462.	2251	OD1	ASP	288	A	<-->	13298	CA	LEU	78	B	3.83
463.	2251	OD1	ASP	288	A	<-->	13301	CB	LEU	78	B	2.90
464.	2251	OD1	ASP	288	A	<-->	13302	CG	LEU	78	B	1.65
465.	2251	OD1	ASP	288	A	<-->	13303	CD1	LEU	78	B	0.98
466.	2251	OD1	ASP	288	A	<-->	13304	CD2	LEU	78	B	2.00
467.	2251	OD1	ASP	288	A	<-->	13786	CB	SER	135	B	3.90
468.	2252	OD2	ASP	288	A	<-->	13295	C	GLY	77	B	2.77
469.	2252	OD2	ASP	288	A	<-->	13296	O	GLY	77	B	1.79
470.	2252	OD2	ASP	288	A	<-->	13297	N	LEU	78	B	3.19
471.	2252	OD2	ASP	288	A	<-->	13298	CA	LEU	78	B	2.94
472.	2252	OD2	ASP	288	A	<-->	13301	CB	LEU	78	B	1.84
473.	2252	OD2	ASP	288	A	<-->	13302	CG	LEU	78	B	2.07
474.	2252	OD2	ASP	288	A	<-->	13303	CD1	LEU	78	B	2.22
475.	2252	OD2	ASP	288	A	<-->	13304	CD2	LEU	78	B	2.57
476.	2252	OD2	ASP	288	A	<-->	13782	N	SER	135	B	3.77
477.	2252	OD2	ASP	288	A	<-->	13783	CA	SER	135	B	3.10
478.	2252	OD2	ASP	288	A	<-->	13784	C	SER	135	B	2.93
479.	2252	OD2	ASP	288	A	<-->	13785	O	SER	135	B	3.34
480.	2252	OD2	ASP	288	A	<-->	13786	CB	SER	135	B	2.34
481.	2252	OD2	ASP	288	A	<-->	13787	OG	SER	135	B	3.53
482.	2252	OD2	ASP	288	A	<-->	13788	N	GLU	136	B	3.23
483.	2252	OD2	ASP	288	A	<-->	13792	CB	GLU	136	B	3.55
484.	2253	N	PRO	289	A	<-->	13139	CG2	THR	57	B	3.80
485.	2253	N	PRO	289	A	<-->	13290	C	ALA	76	B	3.65
486.	2253	N	PRO	289	A	<-->	13293	N	GLY	77	B	2.65
487.	2253	N	PRO	289	A	<-->	13294	CA	GLY	77	B	2.88
488.	2253	N	PRO	289	A	<-->	13295	C	GLY	77	B	3.24
489.	2253	N	PRO	289	A	<-->	13297	N	LEU	78	B	3.29
490.	2254	CA	PRO	289	A	<-->	13137	CB	THR	57	B	3.65
491.	2254	CA	PRO	289	A	<-->	13138	OG1	THR	57	B	3.67
492.	2254	CA	PRO	289	A	<-->	13139	CG2	THR	57	B	2.55
493.	2254	CA	PRO	289	A	<-->	13289	CA	ALA	76	B	3.85
494.	2254	CA	PRO	289	A	<-->	13290	C	ALA	76	B	3.71
495.	2254	CA	PRO	289	A	<-->	13293	N	GLY	77	B	3.08
496.	2254	CA	PRO	289	A	<-->	13294	CA	GLY	77	B	3.84
497.	2255	C	PRO	289	A	<-->	13137	CB	THR	57	B	3.76
498.	2255	C	PRO	289	A	<-->	13138	OG1	THR	57	B	3.22
499.	2255	C	PRO	289	A	<-->	13139	CG2	THR	57	B	3.21
500.	2257	CB	PRO	289	A	<-->	13137	CB	THR	57	B	2.91
501.	2257	CB	PRO	289	A	<-->	13138	OG1	THR	57	B	3.43
502.	2257	CB	PRO	289	A	<-->	13139	CG2	THR	57	B	1.84
503.	2257	CB	PRO	289	A	<-->	13275	OD1	ASP	74	B	3.74
504.	2257	CB	PRO	289	A	<-->	13288	N	ALA	76	B	3.60
505.	2257	CB	PRO	289	A	<-->	13289	CA	ALA	76	B	2.38
506.	2257	CB	PRO	289	A	<-->	13290	C	ALA	76	B	2.51

507.	2257	CB	PRO	289	A	<-->	13291	O	ALA	76	B	3.44
508.	2257	CB	PRO	289	A	<-->	13292	CB	ALA	76	B	3.01
509.	2257	CB	PRO	289	A	<-->	13293	N	GLY	77	B	2.38
510.	2257	CB	PRO	289	A	<-->	13294	CA	GLY	77	B	3.55
511.	2258	CG	PRO	289	A	<-->	13139	CG2	THR	57	B	3.30
512.	2258	CG	PRO	289	A	<-->	13288	N	ALA	76	B	3.55
513.	2258	CG	PRO	289	A	<-->	13289	CA	ALA	76	B	2.30
514.	2258	CG	PRO	289	A	<-->	13290	C	ALA	76	B	1.49
515.	2258	CG	PRO	289	A	<-->	13291	O	ALA	76	B	2.18
516.	2258	CG	PRO	289	A	<-->	13292	CB	ALA	76	B	3.00
517.	2258	CG	PRO	289	A	<-->	13293	N	GLY	77	B	1.39
518.	2258	CG	PRO	289	A	<-->	13294	CA	GLY	77	B	2.31
519.	2258	CG	PRO	289	A	<-->	13295	C	GLY	77	B	3.51
520.	2258	CG	PRO	289	A	<-->	13297	N	LEU	78	B	3.77
521.	2259	CD	PRO	289	A	<-->	13289	CA	ALA	76	B	3.53
522.	2259	CD	PRO	289	A	<-->	13290	C	ALA	76	B	2.51
523.	2259	CD	PRO	289	A	<-->	13291	O	ALA	76	B	3.15
524.	2259	CD	PRO	289	A	<-->	13293	N	GLY	77	B	1.42
525.	2259	CD	PRO	289	A	<-->	13294	CA	GLY	77	B	1.49
526.	2259	CD	PRO	289	A	<-->	13295	C	GLY	77	B	2.31
527.	2259	CD	PRO	289	A	<-->	13296	O	GLY	77	B	3.30
528.	2259	CD	PRO	289	A	<-->	13297	N	LEU	78	B	2.73
529.	2260	N	VAL	290	A	<-->	13137	CB	THR	57	B	3.49
530.	2260	N	VAL	290	A	<-->	13138	OG1	THR	57	B	2.59
531.	2260	N	VAL	290	A	<-->	13139	CG2	THR	57	B	3.26
532.	2261	CA	VAL	290	A	<-->	13138	OG1	THR	57	B	2.94
533.	2262	C	VAL	290	A	<-->	13137	CB	THR	57	B	3.81
534.	2262	C	VAL	290	A	<-->	13138	OG1	THR	57	B	2.53
535.	2263	O	VAL	290	A	<-->	13138	OG1	THR	57	B	3.66
536.	2264	CB	VAL	290	A	<-->	13138	OG1	THR	57	B	3.61
537.	2266	CG2	VAL	290	A	<-->	13138	OG1	THR	57	B	3.15
538.	2267	N	PRO	291	A	<-->	13133	N	THR	57	B	3.85
539.	2267	N	PRO	291	A	<-->	13134	CA	THR	57	B	3.79
540.	2267	N	PRO	291	A	<-->	13137	CB	THR	57	B	2.72
541.	2267	N	PRO	291	A	<-->	13138	OG1	THR	57	B	1.59
542.	2267	N	PRO	291	A	<-->	13139	CG2	THR	57	B	3.66
543.	2268	CA	PRO	291	A	<-->	13137	CB	THR	57	B	3.33
544.	2268	CA	PRO	291	A	<-->	13138	OG1	THR	57	B	2.62
545.	2269	C	PRO	291	A	<-->	13138	OG1	THR	57	B	3.73
546.	2271	CB	PRO	291	A	<-->	13129	CB	ASN	56	B	3.41
547.	2271	CB	PRO	291	A	<-->	13130	CG	ASN	56	B	3.86
548.	2271	CB	PRO	291	A	<-->	13131	OD1	ASN	56	B	3.73
549.	2271	CB	PRO	291	A	<-->	13133	N	THR	57	B	2.88
550.	2271	CB	PRO	291	A	<-->	13134	CA	THR	57	B	3.34
551.	2271	CB	PRO	291	A	<-->	13137	CB	THR	57	B	2.79
552.	2271	CB	PRO	291	A	<-->	13138	OG1	THR	57	B	2.66
553.	2272	CG	PRO	291	A	<-->	13126	CA	ASN	56	B	3.26
554.	2272	CG	PRO	291	A	<-->	13127	C	ASN	56	B	2.74
555.	2272	CG	PRO	291	A	<-->	13128	O	ASN	56	B	3.77
556.	2272	CG	PRO	291	A	<-->	13129	CB	ASN	56	B	3.04
557.	2272	CG	PRO	291	A	<-->	13130	CG	ASN	56	B	3.75
558.	2272	CG	PRO	291	A	<-->	13131	OD1	ASN	56	B	3.59
559.	2272	CG	PRO	291	A	<-->	13133	N	THR	57	B	1.58
560.	2272	CG	PRO	291	A	<-->	13134	CA	THR	57	B	2.14
561.	2272	CG	PRO	291	A	<-->	13135	C	THR	57	B	3.35
562.	2272	CG	PRO	291	A	<-->	13137	CB	THR	57	B	2.04
563.	2272	CG	PRO	291	A	<-->	13138	OG1	THR	57	B	1.85
564.	2272	CG	PRO	291	A	<-->	13139	CG2	THR	57	B	3.56
565.	2272	CG	PRO	291	A	<-->	13140	N	GLU	58	B	3.71
566.	2273	CD	PRO	291	A	<-->	13127	C	ASN	56	B	3.73

567.	2273	CD	PRO	291	A	<-->	13133	N	THR	57	B	2.62
568.	2273	CD	PRO	291	A	<-->	13134	CA	THR	57	B	2.47
569.	2273	CD	PRO	291	A	<-->	13135	C	THR	57	B	3.86
570.	2273	CD	PRO	291	A	<-->	13137	CB	THR	57	B	1.73
571.	2273	CD	PRO	291	A	<-->	13138	OG1	THR	57	B	0.50
572.	2273	CD	PRO	291	A	<-->	13139	CG2	THR	57	B	2.87
573.	2274	N	THR	292	A	<-->	13138	OG1	THR	57	B	3.85
574.	2280	CG2	THR	292	A	<-->	13290	C	ALA	76	B	3.36
575.	2280	CG2	THR	292	A	<-->	13291	O	ALA	76	B	2.74
576.	2280	CG2	THR	292	A	<-->	13292	CB	ALA	76	B	3.27
577.	2296	CZ	ARG	294	A	<-->	13796	OE2	GLU	136	B	3.86
578.	2297	NH1	ARG	294	A	<-->	13793	CG	GLU	136	B	3.85
579.	2297	NH1	ARG	294	A	<-->	13794	CD	GLU	136	B	3.14
580.	2297	NH1	ARG	294	A	<-->	13795	OE1	GLU	136	B	3.28
581.	2297	NH1	ARG	294	A	<-->	13796	OE2	GLU	136	B	3.20
582.	2298	NH2	ARG	294	A	<-->	13793	CG	GLU	136	B	3.84
583.	2298	NH2	ARG	294	A	<-->	13794	CD	GLU	136	B	3.82
584.	2298	NH2	ARG	294	A	<-->	13796	OE2	GLU	136	B	3.59
585.	2298	NH2	ARG	294	A	<-->	13803	OD1	ASN	137	B	3.31
586.	2298	NH2	ARG	294	A	<-->	13813	NE2	GLN	138	B	3.88
587.	6192	CB	GLN	799	A	<-->	13796	OE2	GLU	136	B	3.86
588.	6193	CG	GLN	799	A	<-->	13794	CD	GLU	136	B	3.12
589.	6193	CG	GLN	799	A	<-->	13795	OE1	GLU	136	B	3.27
590.	6193	CG	GLN	799	A	<-->	13796	OE2	GLU	136	B	2.36
591.	6194	CD	GLN	799	A	<-->	13794	CD	GLU	136	B	3.36
592.	6194	CD	GLN	799	A	<-->	13795	OE1	GLU	136	B	3.51
593.	6194	CD	GLN	799	A	<-->	13796	OE2	GLU	136	B	2.50
594.	6195	OE1	GLN	799	A	<-->	13794	CD	GLU	136	B	3.78
595.	6195	OE1	GLN	799	A	<-->	13795	OE1	GLU	136	B	3.57
596.	6195	OE1	GLN	799	A	<-->	13796	OE2	GLU	136	B	3.24
597.	6196	NE2	GLN	799	A	<-->	13794	CD	GLU	136	B	3.85
598.	6196	NE2	GLN	799	A	<-->	13796	OE2	GLU	136	B	2.74
599.	7808	NH2	ARG	1011	A	<-->	13795	OE1	GLU	136	B	3.50

Salt bridges

	<----- A T O M 1 ----->						<----- A T O M 2 ----->					
	Atom no.	Atom name	Res name	Res no.	Chain		Atom no.	Atom name	Res name	Res no.	Chain	Distance
1.	843	NH1	ARG	107	A	<-->	16232	OD2	ASP	443	B	3.10
2.	899	OD1	ASP	114	A	<-->	16993	NH2	ARG	539	B	2.61
3.	1997	OE2	GLU	255	A	<-->	13124	NH2	ARG	55	B	1.61
4.	2297	NH1	ARG	294	A	<-->	13796	OE2	GLU	136	B	3.20
5.	7808	NH2	ARG	1011	A	<-->	13795	OE1	GLU	136	B	3.50

Number of salt bridges: 5

Number of hydrogen bonds: 12

Number of non-bonded contacts: 599

[B] ROBO4 (WT) + SLIT2 (WT) FULL PROTEIN

Hydrogen bonds

	<----- A T O M 1 ----->						<----- A T O M 2 ----->					
	Atom no.	Atom name	Res name	Res no.	Chain		Atom no.	Atom name	Res name	Res no.	Chain	Distance
1.	2320	O	LEU	310	A	<-->	14148	NE2	HIS	833	B	2.90
2.	2329	N	GLY	312	A	<-->	14145	ND1	HIS	833	B	2.71
3.	2702	N	ASN	360	A	<-->	14566	O	PRO	889	B	2.96
4.	2710	N	GLY	361	A	<-->	14562	O	GLY	888	B	2.82
5.	2916	OG	SER	386	A	<-->	14172	O	ILE	837	B	2.27
6.	3406	NE2	HIS	453	A	<-->	12896	N	GLU	677	B	2.84
7.	3499	O	LEU	465	A	<-->	11788	OH	TYR	534	B	3.24
8.	3507	O	LYS	466	A	<-->	11788	OH	TYR	534	B	3.29

Non-bonded contacts

	<----- A T O M 1 ----->						<----- A T O M 2 ----->					
	Atom no.	Atom name	Res name	Res no.	Chain		Atom no.	Atom name	Res name	Res no.	Chain	Distance
1.	2139	CD	ARG	285	A	<-->	14147	CE1	HIS	833	B	2.92
2.	2139	CD	ARG	285	A	<-->	14148	NE2	HIS	833	B	2.97
3.	2140	NE	ARG	285	A	<-->	14145	ND1	HIS	833	B	3.23
4.	2140	NE	ARG	285	A	<-->	14147	CE1	HIS	833	B	2.07
5.	2140	NE	ARG	285	A	<-->	14148	NE2	HIS	833	B	2.78
6.	2141	CZ	ARG	285	A	<-->	14144	CG	HIS	833	B	2.96
7.	2141	CZ	ARG	285	A	<-->	14145	ND1	HIS	833	B	1.92
8.	2141	CZ	ARG	285	A	<-->	14146	CD2	HIS	833	B	2.91
9.	2141	CZ	ARG	285	A	<-->	14147	CE1	HIS	833	B	0.82
10.	2141	CZ	ARG	285	A	<-->	14148	NE2	HIS	833	B	1.90
11.	2142	NH1	ARG	285	A	<-->	14143	CB	HIS	833	B	3.81
12.	2142	NH1	ARG	285	A	<-->	14144	CG	HIS	833	B	2.33
13.	2142	NH1	ARG	285	A	<-->	14145	ND1	HIS	833	B	1.98
14.	2142	NH1	ARG	285	A	<-->	14146	CD2	HIS	833	B	1.76
15.	2142	NH1	ARG	285	A	<-->	14147	CE1	HIS	833	B	0.91
16.	2142	NH1	ARG	285	A	<-->	14148	NE2	HIS	833	B	0.65
17.	2143	NH2	ARG	285	A	<-->	14144	CG	HIS	833	B	2.87
18.	2143	NH2	ARG	285	A	<-->	14145	ND1	HIS	833	B	1.52
19.	2143	NH2	ARG	285	A	<-->	14146	CD2	HIS	833	B	3.41
20.	2143	NH2	ARG	285	A	<-->	14147	CE1	HIS	833	B	1.57
21.	2143	NH2	ARG	285	A	<-->	14148	NE2	HIS	833	B	2.87
22.	2307	CB	ALA	308	A	<-->	14146	CD2	HIS	833	B	3.60
23.	2307	CB	ALA	308	A	<-->	14148	NE2	HIS	833	B	3.05
24.	2320	O	LEU	310	A	<-->	14146	CD2	HIS	833	B	3.76
25.	2320	O	LEU	310	A	<-->	14147	CE1	HIS	833	B	3.17
26.	2320	O	LEU	310	A	<-->	14148	NE2	HIS	833	B	2.90
27.	2323	CD1	LEU	310	A	<-->	14571	CA	GLY	890	B	3.77
28.	2325	N	GLY	311	A	<-->	14142	O	HIS	833	B	3.55
29.	2326	CA	GLY	311	A	<-->	14139	N	HIS	833	B	3.43
30.	2326	CA	GLY	311	A	<-->	14141	C	HIS	833	B	3.69
31.	2326	CA	GLY	311	A	<-->	14142	O	HIS	833	B	2.72
32.	2326	CA	GLY	311	A	<-->	14144	CG	HIS	833	B	3.56
33.	2326	CA	GLY	311	A	<-->	14145	ND1	HIS	833	B	3.27
34.	2326	CA	GLY	311	A	<-->	14146	CD2	HIS	833	B	3.76

35.	2326	CA	GLY	311	A	<-->	14147	CE1	HIS	833	B	3.32
36.	2326	CA	GLY	311	A	<-->	14148	NE2	HIS	833	B	3.64
37.	2327	C	GLY	311	A	<-->	14139	N	HIS	833	B	3.65
38.	2327	C	GLY	311	A	<-->	14145	ND1	HIS	833	B	3.13
39.	2327	C	GLY	311	A	<-->	14147	CE1	HIS	833	B	3.26
40.	2328	O	GLY	311	A	<-->	14147	CE1	HIS	833	B	3.83
41.	2329	N	GLY	312	A	<-->	14133	C	LEU	832	B	3.17
42.	2329	N	GLY	312	A	<-->	14134	O	LEU	832	B	3.01
43.	2329	N	GLY	312	A	<-->	14139	N	HIS	833	B	2.92
44.	2329	N	GLY	312	A	<-->	14144	CG	HIS	833	B	3.60
45.	2329	N	GLY	312	A	<-->	14145	ND1	HIS	833	B	2.71
46.	2329	N	GLY	312	A	<-->	14147	CE1	HIS	833	B	3.32
47.	2330	CA	GLY	312	A	<-->	14133	C	LEU	832	B	3.69
48.	2330	CA	GLY	312	A	<-->	14134	O	LEU	832	B	3.19
49.	2330	CA	GLY	312	A	<-->	14145	ND1	HIS	833	B	3.55
50.	2331	C	GLY	312	A	<-->	14133	C	LEU	832	B	3.26
51.	2331	C	GLY	312	A	<-->	14134	O	LEU	832	B	2.33
52.	2331	C	GLY	312	A	<-->	14139	N	HIS	833	B	3.88
53.	2332	O	GLY	312	A	<-->	14131	N	LEU	832	B	3.51
54.	2332	O	GLY	312	A	<-->	14132	CA	LEU	832	B	3.20
55.	2332	O	GLY	312	A	<-->	14133	C	LEU	832	B	2.32
56.	2332	O	GLY	312	A	<-->	14134	O	LEU	832	B	1.32
57.	2332	O	GLY	312	A	<-->	14135	CB	LEU	832	B	3.26
58.	2332	O	GLY	312	A	<-->	14139	N	HIS	833	B	3.28
59.	2333	N	LEU	313	A	<-->	14134	O	LEU	832	B	3.31
60.	2334	CA	LEU	313	A	<-->	14134	O	LEU	832	B	3.54
61.	2337	CB	LEU	313	A	<-->	14578	CB	GLU	891	B	3.67
62.	2337	CB	LEU	313	A	<-->	14579	CG	GLU	891	B	2.44
63.	2337	CB	LEU	313	A	<-->	14580	CD	GLU	891	B	2.98
64.	2337	CB	LEU	313	A	<-->	14582	OE2	GLU	891	B	3.01
65.	2338	CG	LEU	313	A	<-->	14134	O	LEU	832	B	3.28
66.	2338	CG	LEU	313	A	<-->	14575	CA	GLU	891	B	3.78
67.	2338	CG	LEU	313	A	<-->	14578	CB	GLU	891	B	2.77
68.	2338	CG	LEU	313	A	<-->	14579	CG	GLU	891	B	1.70
69.	2338	CG	LEU	313	A	<-->	14580	CD	GLU	891	B	1.73
70.	2338	CG	LEU	313	A	<-->	14581	OE1	GLU	891	B	2.51
71.	2338	CG	LEU	313	A	<-->	14582	OE2	GLU	891	B	2.17
72.	2339	CD1	LEU	313	A	<-->	14134	O	LEU	832	B	3.12
73.	2339	CD1	LEU	313	A	<-->	14139	N	HIS	833	B	3.89
74.	2339	CD1	LEU	313	A	<-->	14575	CA	GLU	891	B	3.57
75.	2339	CD1	LEU	313	A	<-->	14578	CB	GLU	891	B	2.72
76.	2339	CD1	LEU	313	A	<-->	14579	CG	GLU	891	B	2.49
77.	2339	CD1	LEU	313	A	<-->	14580	CD	GLU	891	B	2.71
78.	2339	CD1	LEU	313	A	<-->	14581	OE1	GLU	891	B	2.87
79.	2339	CD1	LEU	313	A	<-->	14582	OE2	GLU	891	B	3.50
80.	2340	CD2	LEU	313	A	<-->	14575	CA	GLU	891	B	3.22
81.	2340	CD2	LEU	313	A	<-->	14576	C	GLU	891	B	3.62
82.	2340	CD2	LEU	313	A	<-->	14578	CB	GLU	891	B	1.98
83.	2340	CD2	LEU	313	A	<-->	14579	CG	GLU	891	B	0.73
84.	2340	CD2	LEU	313	A	<-->	14580	CD	GLU	891	B	0.82
85.	2340	CD2	LEU	313	A	<-->	14581	OE1	GLU	891	B	1.87
86.	2340	CD2	LEU	313	A	<-->	14582	OE2	GLU	891	B	1.71
87.	2340	CD2	LEU	313	A	<-->	14583	N	MET	892	B	3.89
88.	2340	CD2	LEU	313	A	<-->	14591	N	ALA	893	B	3.72
89.	2341	N	HIS	314	A	<-->	14588	CG	MET	892	B	3.61
90.	2342	CA	HIS	314	A	<-->	14588	CG	MET	892	B	3.48
91.	2345	CB	HIS	314	A	<-->	14587	CB	MET	892	B	3.47
92.	2345	CB	HIS	314	A	<-->	14588	CG	MET	892	B	2.30
93.	2345	CB	HIS	314	A	<-->	14589	SD	MET	892	B	3.33
94.	2345	CB	HIS	314	A	<-->	14590	CE	MET	892	B	3.76

95.	2346	CG	HIS	314	A	<-->	14587	CB	MET	892	B	3.08
96.	2346	CG	HIS	314	A	<-->	14588	CG	MET	892	B	1.58
97.	2346	CG	HIS	314	A	<-->	14589	SD	MET	892	B	1.92
98.	2346	CG	HIS	314	A	<-->	14590	CE	MET	892	B	2.77
99.	2347	ND1	HIS	314	A	<-->	14588	CG	MET	892	B	2.66
100.	2347	ND1	HIS	314	A	<-->	14589	SD	MET	892	B	1.92
101.	2347	ND1	HIS	314	A	<-->	14590	CE	MET	892	B	2.46
102.	2348	CD2	HIS	314	A	<-->	14583	N	MET	892	B	3.54
103.	2348	CD2	HIS	314	A	<-->	14584	CA	MET	892	B	3.15
104.	2348	CD2	HIS	314	A	<-->	14585	C	MET	892	B	3.04
105.	2348	CD2	HIS	314	A	<-->	14587	CB	MET	892	B	2.51
106.	2348	CD2	HIS	314	A	<-->	14588	CG	MET	892	B	1.14
107.	2348	CD2	HIS	314	A	<-->	14589	SD	MET	892	B	1.35
108.	2348	CD2	HIS	314	A	<-->	14590	CE	MET	892	B	2.89
109.	2348	CD2	HIS	314	A	<-->	14591	N	ALA	893	B	2.65
110.	2348	CD2	HIS	314	A	<-->	14592	CA	ALA	893	B	3.43
111.	2348	CD2	HIS	314	A	<-->	14595	CB	ALA	893	B	3.74
112.	2349	CE1	HIS	314	A	<-->	14588	CG	MET	892	B	2.92
113.	2349	CE1	HIS	314	A	<-->	14589	SD	MET	892	B	1.38
114.	2349	CE1	HIS	314	A	<-->	14590	CE	MET	892	B	2.42
115.	2349	CE1	HIS	314	A	<-->	14591	N	ALA	893	B	3.84
116.	2349	CE1	HIS	314	A	<-->	14592	CA	ALA	893	B	3.84
117.	2349	CE1	HIS	314	A	<-->	14595	CB	ALA	893	B	3.87
118.	2350	NE2	HIS	314	A	<-->	14584	CA	MET	892	B	3.77
119.	2350	NE2	HIS	314	A	<-->	14585	C	MET	892	B	3.09
120.	2350	NE2	HIS	314	A	<-->	14586	O	MET	892	B	3.70
121.	2350	NE2	HIS	314	A	<-->	14587	CB	MET	892	B	3.38
122.	2350	NE2	HIS	314	A	<-->	14588	CG	MET	892	B	2.30
123.	2350	NE2	HIS	314	A	<-->	14589	SD	MET	892	B	0.96
124.	2350	NE2	HIS	314	A	<-->	14590	CE	MET	892	B	2.72
125.	2350	NE2	HIS	314	A	<-->	14591	N	ALA	893	B	2.52
126.	2350	NE2	HIS	314	A	<-->	14592	CA	ALA	893	B	2.69
127.	2350	NE2	HIS	314	A	<-->	14595	CB	ALA	893	B	2.94
128.	2358	CD2	TRP	315	A	<-->	11320	NZ	LYS	475	B	2.89
129.	2359	NE1	TRP	315	A	<-->	11320	NZ	LYS	475	B	3.38
130.	2360	CE2	TRP	315	A	<-->	11319	CE	LYS	475	B	3.20
131.	2360	CE2	TRP	315	A	<-->	11320	NZ	LYS	475	B	2.32
132.	2361	CE3	TRP	315	A	<-->	11319	CE	LYS	475	B	3.76
133.	2361	CE3	TRP	315	A	<-->	11320	NZ	LYS	475	B	2.96
134.	2362	CZ2	TRP	315	A	<-->	11318	CD	LYS	475	B	3.44
135.	2362	CZ2	TRP	315	A	<-->	11319	CE	LYS	475	B	1.97
136.	2362	CZ2	TRP	315	A	<-->	11320	NZ	LYS	475	B	1.65
137.	2363	CZ3	TRP	315	A	<-->	11318	CD	LYS	475	B	3.42
138.	2363	CZ3	TRP	315	A	<-->	11319	CE	LYS	475	B	2.74
139.	2363	CZ3	TRP	315	A	<-->	11320	NZ	LYS	475	B	2.42
140.	2364	CH2	TRP	315	A	<-->	11317	CG	LYS	475	B	3.75
141.	2364	CH2	TRP	315	A	<-->	11318	CD	LYS	475	B	2.69
142.	2364	CH2	TRP	315	A	<-->	11319	CE	LYS	475	B	1.57
143.	2364	CH2	TRP	315	A	<-->	11320	NZ	LYS	475	B	1.70
144.	2416	CE2	PHE	321	A	<-->	14574	N	GLU	891	B	3.76
145.	2416	CE2	PHE	321	A	<-->	14575	CA	GLU	891	B	3.73
146.	2666	CG2	VAL	354	A	<-->	14584	CA	MET	892	B	3.85
147.	2666	CG2	VAL	354	A	<-->	14587	CB	MET	892	B	3.43
148.	2680	CD1	LEU	356	A	<-->	12055	CG	ASN	567	B	3.76
149.	2680	CD1	LEU	356	A	<-->	12057	ND2	ASN	567	B	3.07
150.	2681	CD2	LEU	356	A	<-->	11324	O	PHE	476	B	3.75
151.	2681	CD2	LEU	356	A	<-->	11343	N	CYS	478	B	3.47
152.	2691	N	PRO	358	A	<-->	14328	OD1	ASN	859	B	3.36
153.	2692	CA	PRO	358	A	<-->	14326	CB	ASN	859	B	3.36
154.	2692	CA	PRO	358	A	<-->	14327	CG	ASN	859	B	3.28

155. 2692 CA PRO 358 A <--> 14328 OD1 ASN 859 B 2.85
156. 2695 CB PRO 358 A <--> 14323 CA ASN 859 B 3.86
157. 2695 CB PRO 358 A <--> 14326 CB ASN 859 B 2.40
158. 2695 CB PRO 358 A <--> 14327 CG ASN 859 B 1.89
159. 2695 CB PRO 358 A <--> 14328 OD1 ASN 859 B 1.70
160. 2695 CB PRO 358 A <--> 14329 ND2 ASN 859 B 2.85
161. 2696 CG PRO 358 A <--> 14326 CB ASN 859 B 3.71
162. 2696 CG PRO 358 A <--> 14327 CG ASN 859 B 2.71
163. 2696 CG PRO 358 A <--> 14328 OD1 ASN 859 B 2.28
164. 2696 CG PRO 358 A <--> 14329 ND2 ASN 859 B 3.13
165. 2696 CG PRO 358 A <--> 14577 O GLU 891 B 3.48
166. 2697 CD PRO 358 A <--> 14327 CG ASN 859 B 3.84
167. 2697 CD PRO 358 A <--> 14328 OD1 ASN 859 B 3.01
168. 2698 N GLY 359 A <--> 14326 CB ASN 859 B 3.61
169. 2702 N ASN 360 A <--> 14565 C PRO 889 B 3.81
170. 2702 N ASN 360 A <--> 14566 O PRO 889 B 2.96
171. 2703 CA ASN 360 A <--> 14566 O PRO 889 B 3.38
172. 2703 CA ASN 360 A <--> 14571 CA GLY 890 B 3.19
173. 2704 C ASN 360 A <--> 14565 C PRO 889 B 3.81
174. 2704 C ASN 360 A <--> 14566 O PRO 889 B 3.68
175. 2704 C ASN 360 A <--> 14570 N GLY 890 B 3.47
176. 2704 C ASN 360 A <--> 14571 CA GLY 890 B 2.92
177. 2705 O ASN 360 A <--> 14571 CA GLY 890 B 3.80
178. 2710 N GLY 361 A <--> 14561 C GLY 888 B 3.76
179. 2710 N GLY 361 A <--> 14562 O GLY 888 B 2.82
180. 2710 N GLY 361 A <--> 14564 CA PRO 889 B 3.77
181. 2710 N GLY 361 A <--> 14565 C PRO 889 B 2.78
182. 2710 N GLY 361 A <--> 14566 O PRO 889 B 2.99
183. 2710 N GLY 361 A <--> 14570 N GLY 890 B 2.46
184. 2710 N GLY 361 A <--> 14571 CA GLY 890 B 2.44
185. 2710 N GLY 361 A <--> 14572 C GLY 890 B 3.88
186. 2711 CA GLY 361 A <--> 14562 O GLY 888 B 2.82
187. 2711 CA GLY 361 A <--> 14565 C PRO 889 B 3.48
188. 2711 CA GLY 361 A <--> 14570 N GLY 890 B 2.91
189. 2711 CA GLY 361 A <--> 14571 CA GLY 890 B 3.17
190. 2712 C GLY 361 A <--> 14570 N GLY 890 B 3.10
191. 2712 C GLY 361 A <--> 14571 CA GLY 890 B 2.98
192. 2713 O GLY 361 A <--> 14565 C PRO 889 B 3.88
193. 2713 O GLY 361 A <--> 14570 N GLY 890 B 2.65
194. 2713 O GLY 361 A <--> 14571 CA GLY 890 B 2.15
195. 2713 O GLY 361 A <--> 14572 C GLY 890 B 3.29
196. 2713 O GLY 361 A <--> 14574 N GLU 891 B 3.88
197. 2822 ND1 HIS 375 A <--> 15927 CG2 VAL 1069 B 3.00
198. 2823 CD2 HIS 375 A <--> 15927 CG2 VAL 1069 B 3.71
199. 2824 CE1 HIS 375 A <--> 15925 CB VAL 1069 B 3.06
200. 2824 CE1 HIS 375 A <--> 15927 CG2 VAL 1069 B 2.04
201. 2825 NE2 HIS 375 A <--> 15925 CB VAL 1069 B 3.82
202. 2825 NE2 HIS 375 A <--> 15927 CG2 VAL 1069 B 2.64
203. 2892 C VAL 384 A <--> 14176 CD1 ILE 837 B 3.75
204. 2893 O VAL 384 A <--> 14174 CG1 ILE 837 B 3.48
205. 2893 O VAL 384 A <--> 14176 CD1 ILE 837 B 2.55
206. 2903 CD1 TRP 385 A <--> 14187 CB VAL 839 B 3.34
207. 2903 CD1 TRP 385 A <--> 14188 CG1 VAL 839 B 3.20
208. 2904 CD2 TRP 385 A <--> 14188 CG1 VAL 839 B 3.33
209. 2904 CD2 TRP 385 A <--> 14545 CZ ARG 885 B 3.69
210. 2904 CD2 TRP 385 A <--> 14546 NH1 ARG 885 B 3.16
211. 2904 CD2 TRP 385 A <--> 14547 NH2 ARG 885 B 3.31
212. 2905 NE1 TRP 385 A <--> 14183 N VAL 839 B 3.57
213. 2905 NE1 TRP 385 A <--> 14184 CA VAL 839 B 3.40
214. 2905 NE1 TRP 385 A <--> 14187 CB VAL 839 B 2.22

215.	2905	NE1	TRP	385	A	<-->	14188	CG1	VAL	839	B	1.83
216.	2905	NE1	TRP	385	A	<-->	14189	CG2	VAL	839	B	3.27
217.	2905	NE1	TRP	385	A	<-->	14546	NH1	ARG	885	B	3.82
218.	2906	CE2	TRP	385	A	<-->	14183	N	VAL	839	B	3.77
219.	2906	CE2	TRP	385	A	<-->	14184	CA	VAL	839	B	3.89
220.	2906	CE2	TRP	385	A	<-->	14187	CB	VAL	839	B	3.02
221.	2906	CE2	TRP	385	A	<-->	14188	CG1	VAL	839	B	1.94
222.	2906	CE2	TRP	385	A	<-->	14545	CZ	ARG	885	B	3.55
223.	2906	CE2	TRP	385	A	<-->	14546	NH1	ARG	885	B	2.61
224.	2906	CE2	TRP	385	A	<-->	14547	NH2	ARG	885	B	3.64
225.	2907	CE3	TRP	385	A	<-->	14545	CZ	ARG	885	B	3.18
226.	2907	CE3	TRP	385	A	<-->	14546	NH1	ARG	885	B	2.99
227.	2907	CE3	TRP	385	A	<-->	14547	NH2	ARG	885	B	2.62
228.	2908	CZ2	TRP	385	A	<-->	14183	N	VAL	839	B	3.75
229.	2908	CZ2	TRP	385	A	<-->	14184	CA	VAL	839	B	3.86
230.	2908	CZ2	TRP	385	A	<-->	14186	O	VAL	839	B	3.68
231.	2908	CZ2	TRP	385	A	<-->	14187	CB	VAL	839	B	3.30
232.	2908	CZ2	TRP	385	A	<-->	14188	CG1	VAL	839	B	1.88
233.	2908	CZ2	TRP	385	A	<-->	14545	CZ	ARG	885	B	2.81
234.	2908	CZ2	TRP	385	A	<-->	14546	NH1	ARG	885	B	1.56
235.	2908	CZ2	TRP	385	A	<-->	14547	NH2	ARG	885	B	3.40
236.	2909	CZ3	TRP	385	A	<-->	14544	NE	ARG	885	B	3.44
237.	2909	CZ3	TRP	385	A	<-->	14545	CZ	ARG	885	B	2.26
238.	2909	CZ3	TRP	385	A	<-->	14546	NH1	ARG	885	B	2.06
239.	2909	CZ3	TRP	385	A	<-->	14547	NH2	ARG	885	B	2.21
240.	2910	CH2	TRP	385	A	<-->	14188	CG1	VAL	839	B	3.25
241.	2910	CH2	TRP	385	A	<-->	14543	CD	ARG	885	B	3.56
242.	2910	CH2	TRP	385	A	<-->	14544	NE	ARG	885	B	3.08
243.	2910	CH2	TRP	385	A	<-->	14545	CZ	ARG	885	B	2.01
244.	2910	CH2	TRP	385	A	<-->	14546	NH1	ARG	885	B	1.03
245.	2910	CH2	TRP	385	A	<-->	14547	NH2	ARG	885	B	2.68
246.	2911	N	SER	386	A	<-->	14172	O	ILE	837	B	3.79
247.	2911	N	SER	386	A	<-->	14547	NH2	ARG	885	B	3.59
248.	2912	CA	SER	386	A	<-->	14172	O	ILE	837	B	3.85
249.	2912	CA	SER	386	A	<-->	14547	NH2	ARG	885	B	3.25
250.	2913	C	SER	386	A	<-->	14545	CZ	ARG	885	B	3.44
251.	2913	C	SER	386	A	<-->	14547	NH2	ARG	885	B	2.09
252.	2914	O	SER	386	A	<-->	14544	NE	ARG	885	B	3.02
253.	2914	O	SER	386	A	<-->	14545	CZ	ARG	885	B	2.34
254.	2914	O	SER	386	A	<-->	14546	NH1	ARG	885	B	3.42
255.	2914	O	SER	386	A	<-->	14547	NH2	ARG	885	B	1.08
256.	2915	CB	SER	386	A	<-->	14172	O	ILE	837	B	2.79
257.	2915	CB	SER	386	A	<-->	14547	NH2	ARG	885	B	3.61
258.	2916	OG	SER	386	A	<-->	14169	N	ILE	837	B	3.72
259.	2916	OG	SER	386	A	<-->	14171	C	ILE	837	B	3.43
260.	2916	OG	SER	386	A	<-->	14172	O	ILE	837	B	2.27
261.	2917	N	LEU	387	A	<-->	14547	NH2	ARG	885	B	2.83
262.	2918	CA	LEU	387	A	<-->	14545	CZ	ARG	885	B	3.78
263.	2918	CA	LEU	387	A	<-->	14547	NH2	ARG	885	B	2.84
264.	2919	C	LEU	387	A	<-->	14544	NE	ARG	885	B	3.88
265.	2919	C	LEU	387	A	<-->	14545	CZ	ARG	885	B	3.67
266.	2919	C	LEU	387	A	<-->	14547	NH2	ARG	885	B	3.01
267.	2920	O	LEU	387	A	<-->	14544	NE	ARG	885	B	3.04
268.	2920	O	LEU	387	A	<-->	14545	CZ	ARG	885	B	3.09
269.	2920	O	LEU	387	A	<-->	14547	NH2	ARG	885	B	2.91
270.	2934	CG	ASN	389	A	<-->	14555	CA	ALA	887	B	3.54
271.	2934	CG	ASN	389	A	<-->	14556	C	ALA	887	B	3.83
272.	2934	CG	ASN	389	A	<-->	14559	N	GLY	888	B	3.17
273.	2936	ND2	ASN	389	A	<-->	14554	N	ALA	887	B	3.79
274.	2936	ND2	ASN	389	A	<-->	14555	CA	ALA	887	B	2.53

275.	2936	ND2	ASN	389	A	<-->	14556	C	ALA	887	B	2.53
276.	2936	ND2	ASN	389	A	<-->	14557	O	ALA	887	B	3.73
277.	2936	ND2	ASN	389	A	<-->	14558	CB	ALA	887	B	3.16
278.	2936	ND2	ASN	389	A	<-->	14559	N	GLY	888	B	1.85
279.	2936	ND2	ASN	389	A	<-->	14560	CA	GLY	888	B	3.04
280.	2936	ND2	ASN	389	A	<-->	14561	C	GLY	888	B	3.47
281.	2936	ND2	ASN	389	A	<-->	14562	O	GLY	888	B	3.34
282.	2957	CD2	LEU	392	A	<-->	14356	OH	TYR	862	B	3.75
283.	3141	OH	TYR	416	A	<-->	12607	OH	TYR	638	B	3.86
284.	3226	N	PRO	431	A	<-->	14530	CG2	ILE	883	B	3.69
285.	3227	CA	PRO	431	A	<-->	14530	CG2	ILE	883	B	3.12
286.	3230	CB	PRO	431	A	<-->	14526	C	ILE	883	B	3.81
287.	3230	CB	PRO	431	A	<-->	14527	O	ILE	883	B	3.49
288.	3230	CB	PRO	431	A	<-->	14528	CB	ILE	883	B	2.96
289.	3230	CB	PRO	431	A	<-->	14529	CG1	ILE	883	B	3.42
290.	3230	CB	PRO	431	A	<-->	14530	CG2	ILE	883	B	1.62
291.	3230	CB	PRO	431	A	<-->	14531	CD1	ILE	883	B	3.61
292.	3231	CG	PRO	431	A	<-->	14527	O	ILE	883	B	3.73
293.	3231	CG	PRO	431	A	<-->	14528	CB	ILE	883	B	3.47
294.	3231	CG	PRO	431	A	<-->	14530	CG2	ILE	883	B	1.96
295.	3232	CD	PRO	431	A	<-->	14530	CG2	ILE	883	B	3.24
296.	3235	C	SER	432	A	<-->	14531	CD1	ILE	883	B	3.65
297.	3238	OG	SER	432	A	<-->	14553	SG	CYS	886	B	3.63
298.	3239	N	ARG	433	A	<-->	14529	CG1	ILE	883	B	3.25
299.	3239	N	ARG	433	A	<-->	14531	CD1	ILE	883	B	2.47
300.	3240	CA	ARG	433	A	<-->	14529	CG1	ILE	883	B	3.01
301.	3240	CA	ARG	433	A	<-->	14531	CD1	ILE	883	B	2.01
302.	3241	C	ARG	433	A	<-->	14529	CG1	ILE	883	B	3.68
303.	3241	C	ARG	433	A	<-->	14531	CD1	ILE	883	B	2.23
304.	3242	O	ARG	433	A	<-->	14531	CD1	ILE	883	B	3.29
305.	3243	CB	ARG	433	A	<-->	14528	CB	ILE	883	B	3.39
306.	3243	CB	ARG	433	A	<-->	14529	CG1	ILE	883	B	1.90
307.	3243	CB	ARG	433	A	<-->	14531	CD1	ILE	883	B	1.53
308.	3244	CG	ARG	433	A	<-->	14529	CG1	ILE	883	B	2.88
309.	3244	CG	ARG	433	A	<-->	14531	CD1	ILE	883	B	2.53
310.	3245	CD	ARG	433	A	<-->	14529	CG1	ILE	883	B	3.25
311.	3245	CD	ARG	433	A	<-->	14531	CD1	ILE	883	B	2.69
312.	3246	NE	ARG	433	A	<-->	14525	CA	ILE	883	B	3.67
313.	3246	NE	ARG	433	A	<-->	14528	CB	ILE	883	B	2.95
314.	3246	NE	ARG	433	A	<-->	14529	CG1	ILE	883	B	2.66
315.	3246	NE	ARG	433	A	<-->	14531	CD1	ILE	883	B	2.65
316.	3247	CZ	ARG	433	A	<-->	14525	CA	ILE	883	B	3.68
317.	3247	CZ	ARG	433	A	<-->	14528	CB	ILE	883	B	3.50
318.	3247	CZ	ARG	433	A	<-->	14529	CG1	ILE	883	B	3.49
319.	3247	CZ	ARG	433	A	<-->	14531	CD1	ILE	883	B	3.89
320.	3248	NH1	ARG	433	A	<-->	14499	CB	LYS	879	B	3.39
321.	3248	NH1	ARG	433	A	<-->	14500	CG	LYS	879	B	3.24
322.	3249	NH2	ARG	433	A	<-->	14507	O	GLU	880	B	3.79
323.	3249	NH2	ARG	433	A	<-->	14522	C	GLY	882	B	3.21
324.	3249	NH2	ARG	433	A	<-->	14523	O	GLY	882	B	2.98
325.	3249	NH2	ARG	433	A	<-->	14524	N	ILE	883	B	3.22
326.	3249	NH2	ARG	433	A	<-->	14525	CA	ILE	883	B	3.04
327.	3249	NH2	ARG	433	A	<-->	14528	CB	ILE	883	B	3.27
328.	3249	NH2	ARG	433	A	<-->	14529	CG1	ILE	883	B	3.79
329.	3250	N	PRO	434	A	<-->	14529	CG1	ILE	883	B	3.67
330.	3250	N	PRO	434	A	<-->	14531	CD1	ILE	883	B	2.17
331.	3251	CA	PRO	434	A	<-->	14531	CD1	ILE	883	B	3.40
332.	3254	CB	PRO	434	A	<-->	14531	CD1	ILE	883	B	3.81
333.	3255	CG	PRO	434	A	<-->	14528	CB	ILE	883	B	3.59
334.	3255	CG	PRO	434	A	<-->	14529	CG1	ILE	883	B	3.78

335. 3255 CG PRO 434 A <--> 14530 CG2 ILE 883 B 3.45
336. 3255 CG PRO 434 A <--> 14531 CD1 ILE 883 B 2.84
337. 3256 CD PRO 434 A <--> 14528 CB ILE 883 B 3.13
338. 3256 CD PRO 434 A <--> 14529 CG1 ILE 883 B 2.75
339. 3256 CD PRO 434 A <--> 14530 CG2 ILE 883 B 3.14
340. 3256 CD PRO 434 A <--> 14531 CD1 ILE 883 B 1.56
341. 3390 C GLU 452 A <--> 12904 OE2 GLU 677 B 3.50
342. 3391 O GLU 452 A <--> 12904 OE2 GLU 677 B 3.60
343. 3392 CB GLU 452 A <--> 12917 CZ3 TRP 678 B 3.69
344. 3392 CB GLU 452 A <--> 12918 CH2 TRP 678 B 3.64
345. 3393 CG GLU 452 A <--> 12901 CG GLU 677 B 3.76
346. 3394 CD GLU 452 A <--> 12901 CG GLU 677 B 3.79
347. 3394 CD GLU 452 A <--> 12910 CG TRP 678 B 3.67
348. 3394 CD GLU 452 A <--> 12911 CD1 TRP 678 B 3.82
349. 3394 CD GLU 452 A <--> 12912 CD2 TRP 678 B 2.94
350. 3394 CD GLU 452 A <--> 12913 NE1 TRP 678 B 3.30
351. 3394 CD GLU 452 A <--> 12914 CE2 TRP 678 B 2.68
352. 3394 CD GLU 452 A <--> 12915 CE3 TRP 678 B 3.28
353. 3394 CD GLU 452 A <--> 12916 CZ2 TRP 678 B 2.80
354. 3394 CD GLU 452 A <--> 12917 CZ3 TRP 678 B 3.34
355. 3394 CD GLU 452 A <--> 12918 CH2 TRP 678 B 3.12
356. 3395 OE1 GLU 452 A <--> 12910 CG TRP 678 B 3.56
357. 3395 OE1 GLU 452 A <--> 12911 CD1 TRP 678 B 3.80
358. 3395 OE1 GLU 452 A <--> 12912 CD2 TRP 678 B 2.43
359. 3395 OE1 GLU 452 A <--> 12913 NE1 TRP 678 B 3.06
360. 3395 OE1 GLU 452 A <--> 12914 CE2 TRP 678 B 2.01
361. 3395 OE1 GLU 452 A <--> 12915 CE3 TRP 678 B 2.59
362. 3395 OE1 GLU 452 A <--> 12916 CZ2 TRP 678 B 1.71
363. 3395 OE1 GLU 452 A <--> 12917 CZ3 TRP 678 B 2.31
364. 3395 OE1 GLU 452 A <--> 12918 CH2 TRP 678 B 1.87
365. 3396 OE2 GLU 452 A <--> 12900 CB GLU 677 B 3.44
366. 3396 OE2 GLU 452 A <--> 12901 CG GLU 677 B 3.33
367. 3396 OE2 GLU 452 A <--> 12910 CG TRP 678 B 3.19
368. 3396 OE2 GLU 452 A <--> 12911 CD1 TRP 678 B 3.08
369. 3396 OE2 GLU 452 A <--> 12912 CD2 TRP 678 B 2.98
370. 3396 OE2 GLU 452 A <--> 12913 NE1 TRP 678 B 2.81
371. 3396 OE2 GLU 452 A <--> 12914 CE2 TRP 678 B 2.73
372. 3396 OE2 GLU 452 A <--> 12915 CE3 TRP 678 B 3.72
373. 3396 OE2 GLU 452 A <--> 12916 CZ2 TRP 678 B 3.33
374. 3397 N HIS 453 A <--> 12902 CD GLU 677 B 3.70
375. 3397 N HIS 453 A <--> 12904 OE2 GLU 677 B 2.73
376. 3398 CA HIS 453 A <--> 12901 CG GLU 677 B 3.74
377. 3398 CA HIS 453 A <--> 12902 CD GLU 677 B 2.90
378. 3398 CA HIS 453 A <--> 12903 OE1 GLU 677 B 3.80
379. 3398 CA HIS 453 A <--> 12904 OE2 GLU 677 B 1.75
380. 3399 C HIS 453 A <--> 12904 OE2 GLU 677 B 2.91
381. 3400 O HIS 453 A <--> 12904 OE2 GLU 677 B 3.27
382. 3401 CB HIS 453 A <--> 12901 CG GLU 677 B 3.68
383. 3401 CB HIS 453 A <--> 12902 CD GLU 677 B 2.70
384. 3401 CB HIS 453 A <--> 12903 OE1 GLU 677 B 3.24
385. 3401 CB HIS 453 A <--> 12904 OE2 GLU 677 B 2.03
386. 3402 CG HIS 453 A <--> 12900 CB GLU 677 B 3.00
387. 3402 CG HIS 453 A <--> 12901 CG GLU 677 B 2.77
388. 3402 CG HIS 453 A <--> 12902 CD GLU 677 B 2.24
389. 3402 CG HIS 453 A <--> 12903 OE1 GLU 677 B 2.79
390. 3402 CG HIS 453 A <--> 12904 OE2 GLU 677 B 2.28
391. 3403 ND1 HIS 453 A <--> 12900 CB GLU 677 B 3.17
392. 3403 ND1 HIS 453 A <--> 12901 CG GLU 677 B 3.21
393. 3403 ND1 HIS 453 A <--> 12902 CD GLU 677 B 3.29
394. 3403 ND1 HIS 453 A <--> 12904 OE2 GLU 677 B 3.37

395.	3404	CD2	HIS	453	A	<-->	12896	N	GLU	677	B	3.56
396.	3404	CD2	HIS	453	A	<-->	12897	CA	GLU	677	B	2.95
397.	3404	CD2	HIS	453	A	<-->	12900	CB	GLU	677	B	2.02
398.	3404	CD2	HIS	453	A	<-->	12901	CG	GLU	677	B	2.27
399.	3404	CD2	HIS	453	A	<-->	12902	CD	GLU	677	B	1.87
400.	3404	CD2	HIS	453	A	<-->	12903	OE1	GLU	677	B	1.96
401.	3404	CD2	HIS	453	A	<-->	12904	OE2	GLU	677	B	2.61
402.	3405	CE1	HIS	453	A	<-->	12897	CA	GLU	677	B	3.75
403.	3405	CE1	HIS	453	A	<-->	12900	CB	GLU	677	B	2.41
404.	3405	CE1	HIS	453	A	<-->	12901	CG	GLU	677	B	3.06
405.	3405	CE1	HIS	453	A	<-->	12902	CD	GLU	677	B	3.54
406.	3406	NE2	HIS	453	A	<-->	12896	N	GLU	677	B	2.84
407.	3406	NE2	HIS	453	A	<-->	12897	CA	GLU	677	B	2.58
408.	3406	NE2	HIS	453	A	<-->	12898	C	GLU	677	B	3.82
409.	3406	NE2	HIS	453	A	<-->	12900	CB	GLU	677	B	1.51
410.	3406	NE2	HIS	453	A	<-->	12901	CG	GLU	677	B	2.53
411.	3406	NE2	HIS	453	A	<-->	12902	CD	GLU	677	B	2.90
412.	3406	NE2	HIS	453	A	<-->	12903	OE1	GLU	677	B	3.03
413.	3406	NE2	HIS	453	A	<-->	12904	OE2	GLU	677	B	3.75
414.	3417	CD	PRO	455	A	<-->	14553	SG	CYS	886	B	3.74
415.	3437	OG1	THR	457	A	<-->	12621	CG	ASN	640	B	3.32
416.	3437	OG1	THR	457	A	<-->	12622	OD1	ASN	640	B	3.81
417.	3437	OG1	THR	457	A	<-->	12623	ND2	ASN	640	B	2.68
418.	3442	O	LEU	458	A	<-->	12623	ND2	ASN	640	B	3.82
419.	3444	CG	LEU	458	A	<-->	12604	CE1	TYR	638	B	3.57
420.	3444	CG	LEU	458	A	<-->	12606	CZ	TYR	638	B	3.79
421.	3444	CG	LEU	458	A	<-->	12607	OH	TYR	638	B	3.28
422.	3445	CD1	LEU	458	A	<-->	12604	CE1	TYR	638	B	3.50
423.	3445	CD1	LEU	458	A	<-->	12606	CZ	TYR	638	B	3.70
424.	3445	CD1	LEU	458	A	<-->	12607	OH	TYR	638	B	2.99
425.	3445	CD1	LEU	458	A	<-->	12891	CD2	LEU	675	B	2.79
426.	3446	CD2	LEU	458	A	<-->	12602	CD1	TYR	638	B	3.72
427.	3446	CD2	LEU	458	A	<-->	12604	CE1	TYR	638	B	2.66
428.	3446	CD2	LEU	458	A	<-->	12605	CE2	TYR	638	B	3.70
429.	3446	CD2	LEU	458	A	<-->	12606	CZ	TYR	638	B	2.65
430.	3446	CD2	LEU	458	A	<-->	12607	OH	TYR	638	B	2.43
431.	3450	O	GLU	459	A	<-->	11385	OE1	GLN	483	B	3.56
432.	3456	N	GLN	460	A	<-->	9339	CE3	TRP	223	B	3.89
433.	3457	CA	GLN	460	A	<-->	11383	CG	GLN	483	B	3.33
434.	3460	CB	GLN	460	A	<-->	9330	CA	TRP	223	B	3.68
435.	3460	CB	GLN	460	A	<-->	9333	CB	TRP	223	B	2.59
436.	3460	CB	GLN	460	A	<-->	9334	CG	TRP	223	B	2.93
437.	3460	CB	GLN	460	A	<-->	9336	CD2	TRP	223	B	3.00
438.	3460	CB	GLN	460	A	<-->	9339	CE3	TRP	223	B	2.85
439.	3460	CB	GLN	460	A	<-->	11382	CB	GLN	483	B	3.58
440.	3460	CB	GLN	460	A	<-->	11383	CG	GLN	483	B	3.14
441.	3461	CG	GLN	460	A	<-->	9330	CA	TRP	223	B	3.38
442.	3461	CG	GLN	460	A	<-->	9333	CB	TRP	223	B	2.94
443.	3461	CG	GLN	460	A	<-->	9334	CG	TRP	223	B	3.53
444.	3461	CG	GLN	460	A	<-->	9336	CD2	TRP	223	B	3.42
445.	3461	CG	GLN	460	A	<-->	9339	CE3	TRP	223	B	2.78
446.	3461	CG	GLN	460	A	<-->	9341	CZ3	TRP	223	B	3.83
447.	3461	CG	GLN	460	A	<-->	11379	CA	GLN	483	B	3.89
448.	3461	CG	GLN	460	A	<-->	11382	CB	GLN	483	B	2.41
449.	3461	CG	GLN	460	A	<-->	11383	CG	GLN	483	B	2.62
450.	3462	CD	GLN	460	A	<-->	9336	CD2	TRP	223	B	3.30
451.	3462	CD	GLN	460	A	<-->	9339	CE3	TRP	223	B	2.16
452.	3462	CD	GLN	460	A	<-->	9341	CZ3	TRP	223	B	2.75
453.	3462	CD	GLN	460	A	<-->	11382	CB	GLN	483	B	3.38
454.	3462	CD	GLN	460	A	<-->	11383	CG	GLN	483	B	3.87

455.	3463	OE1	GLN	460	A	<-->	9339	CE3	TRP	223	B	3.05
456.	3463	OE1	GLN	460	A	<-->	9341	CZ3	TRP	223	B	3.33
457.	3463	OE1	GLN	460	A	<-->	11382	CB	GLN	483	B	3.44
458.	3464	NE2	GLN	460	A	<-->	9334	CG	TRP	223	B	3.66
459.	3464	NE2	GLN	460	A	<-->	9336	CD2	TRP	223	B	2.62
460.	3464	NE2	GLN	460	A	<-->	9338	CE2	TRP	223	B	3.52
461.	3464	NE2	GLN	460	A	<-->	9339	CE3	TRP	223	B	1.46
462.	3464	NE2	GLN	460	A	<-->	9340	CZ2	TRP	223	B	3.64
463.	3464	NE2	GLN	460	A	<-->	9341	CZ3	TRP	223	B	1.67
464.	3464	NE2	GLN	460	A	<-->	9342	CH2	TRP	223	B	2.87
465.	3470	CG	LEU	461	A	<-->	9328	OD2	ASP	222	B	3.89
466.	3470	CG	LEU	461	A	<-->	10798	OE1	GLN	409	B	3.46
467.	3471	CD1	LEU	461	A	<-->	9328	OD2	ASP	222	B	3.28
468.	3471	CD1	LEU	461	A	<-->	11774	CD	GLN	533	B	3.83
469.	3471	CD1	LEU	461	A	<-->	11775	OE1	GLN	533	B	3.50
470.	3471	CD1	LEU	461	A	<-->	11776	NE2	GLN	533	B	3.58
471.	3472	CD2	LEU	461	A	<-->	9326	CG	ASP	222	B	3.51
472.	3472	CD2	LEU	461	A	<-->	9327	OD1	ASP	222	B	3.08
473.	3472	CD2	LEU	461	A	<-->	9328	OD2	ASP	222	B	3.54
474.	3472	CD2	LEU	461	A	<-->	9329	N	TRP	223	B	3.84
475.	3472	CD2	LEU	461	A	<-->	9330	CA	TRP	223	B	3.85
476.	3472	CD2	LEU	461	A	<-->	9333	CB	TRP	223	B	2.71
477.	3472	CD2	LEU	461	A	<-->	9334	CG	TRP	223	B	3.55
478.	3472	CD2	LEU	461	A	<-->	10798	OE1	GLN	409	B	2.92
479.	3498	C	LEU	465	A	<-->	11776	NE2	GLN	533	B	3.81
480.	3499	O	LEU	465	A	<-->	11785	CE1	TYR	534	B	3.58
481.	3499	O	LEU	465	A	<-->	11787	CZ	TYR	534	B	3.77
482.	3499	O	LEU	465	A	<-->	11788	OH	TYR	534	B	3.24
483.	3500	CB	LEU	465	A	<-->	11776	NE2	GLN	533	B	3.43
484.	3503	CD2	LEU	465	A	<-->	11776	NE2	GLN	533	B	3.86
485.	3504	N	LYS	466	A	<-->	11774	CD	GLN	533	B	3.23
486.	3504	N	LYS	466	A	<-->	11775	OE1	GLN	533	B	3.38
487.	3504	N	LYS	466	A	<-->	11776	NE2	GLN	533	B	2.79
488.	3504	N	LYS	466	A	<-->	11785	CE1	TYR	534	B	3.66
489.	3505	CA	LYS	466	A	<-->	11774	CD	GLN	533	B	3.19
490.	3505	CA	LYS	466	A	<-->	11775	OE1	GLN	533	B	2.98
491.	3505	CA	LYS	466	A	<-->	11776	NE2	GLN	533	B	3.36
492.	3505	CA	LYS	466	A	<-->	11783	CD1	TYR	534	B	3.10
493.	3505	CA	LYS	466	A	<-->	11785	CE1	TYR	534	B	2.56
494.	3505	CA	LYS	466	A	<-->	11787	CZ	TYR	534	B	3.69
495.	3506	C	LYS	466	A	<-->	11775	OE1	GLN	533	B	3.76
496.	3506	C	LYS	466	A	<-->	11783	CD1	TYR	534	B	3.65
497.	3506	C	LYS	466	A	<-->	11785	CE1	TYR	534	B	2.89
498.	3506	C	LYS	466	A	<-->	11787	CZ	TYR	534	B	3.85
499.	3507	O	LYS	466	A	<-->	11783	CD1	TYR	534	B	3.36
500.	3507	O	LYS	466	A	<-->	11785	CE1	TYR	534	B	2.47
501.	3507	O	LYS	466	A	<-->	11787	CZ	TYR	534	B	3.20
502.	3507	O	LYS	466	A	<-->	11788	OH	TYR	534	B	3.29
503.	3508	CB	LYS	466	A	<-->	11769	CA	GLN	533	B	3.58
504.	3508	CB	LYS	466	A	<-->	11772	CB	GLN	533	B	3.74
505.	3508	CB	LYS	466	A	<-->	11773	CG	GLN	533	B	2.99
506.	3508	CB	LYS	466	A	<-->	11774	CD	GLN	533	B	2.11
507.	3508	CB	LYS	466	A	<-->	11775	OE1	GLN	533	B	1.65
508.	3508	CB	LYS	466	A	<-->	11776	NE2	GLN	533	B	2.85
509.	3508	CB	LYS	466	A	<-->	11777	N	TYR	534	B	3.79
510.	3508	CB	LYS	466	A	<-->	11783	CD1	TYR	534	B	3.09
511.	3508	CB	LYS	466	A	<-->	11785	CE1	TYR	534	B	3.22
512.	3509	CG	LYS	466	A	<-->	11769	CA	GLN	533	B	2.98
513.	3509	CG	LYS	466	A	<-->	11770	C	GLN	533	B	3.20
514.	3509	CG	LYS	466	A	<-->	11772	CB	GLN	533	B	2.76

515.	3509	CG	LYS	466	A	<-->	11773	CG	GLN	533	B	1.69
516.	3509	CG	LYS	466	A	<-->	11774	CD	GLN	533	B	1.57
517.	3509	CG	LYS	466	A	<-->	11775	OE1	GLN	533	B	1.98
518.	3509	CG	LYS	466	A	<-->	11776	NE2	GLN	533	B	2.47
519.	3509	CG	LYS	466	A	<-->	11777	N	TYR	534	B	2.79
520.	3509	CG	LYS	466	A	<-->	11780	O	TYR	534	B	3.89
521.	3509	CG	LYS	466	A	<-->	11783	CD1	TYR	534	B	3.22
522.	3509	CG	LYS	466	A	<-->	11785	CE1	TYR	534	B	3.69
523.	3510	CD	LYS	466	A	<-->	9416	CG	LEU	232	B	3.83
524.	3510	CD	LYS	466	A	<-->	9418	CD2	LEU	232	B	3.52
525.	3510	CD	LYS	466	A	<-->	11769	CA	GLN	533	B	3.60
526.	3510	CD	LYS	466	A	<-->	11770	C	GLN	533	B	3.09
527.	3510	CD	LYS	466	A	<-->	11772	CB	GLN	533	B	3.61
528.	3510	CD	LYS	466	A	<-->	11773	CG	GLN	533	B	2.62
529.	3510	CD	LYS	466	A	<-->	11774	CD	GLN	533	B	3.04
530.	3510	CD	LYS	466	A	<-->	11775	OE1	GLN	533	B	3.47
531.	3510	CD	LYS	466	A	<-->	11776	NE2	GLN	533	B	3.71
532.	3510	CD	LYS	466	A	<-->	11777	N	TYR	534	B	2.09
533.	3510	CD	LYS	466	A	<-->	11778	CA	TYR	534	B	2.91
534.	3510	CD	LYS	466	A	<-->	11779	C	TYR	534	B	3.02
535.	3510	CD	LYS	466	A	<-->	11780	O	TYR	534	B	2.38
536.	3510	CD	LYS	466	A	<-->	11781	CB	TYR	534	B	3.47
537.	3510	CD	LYS	466	A	<-->	11782	CG	TYR	534	B	3.06
538.	3510	CD	LYS	466	A	<-->	11783	CD1	TYR	534	B	2.37
539.	3510	CD	LYS	466	A	<-->	11785	CE1	TYR	534	B	3.03
540.	3511	CE	LYS	466	A	<-->	9416	CG	LEU	232	B	2.55
541.	3511	CE	LYS	466	A	<-->	9417	CD1	LEU	232	B	3.19
542.	3511	CE	LYS	466	A	<-->	9418	CD2	LEU	232	B	1.99
543.	3511	CE	LYS	466	A	<-->	11770	C	GLN	533	B	3.31
544.	3511	CE	LYS	466	A	<-->	11772	CB	GLN	533	B	3.66
545.	3511	CE	LYS	466	A	<-->	11773	CG	GLN	533	B	2.72
546.	3511	CE	LYS	466	A	<-->	11774	CD	GLN	533	B	3.72
547.	3511	CE	LYS	466	A	<-->	11777	N	TYR	534	B	2.11
548.	3511	CE	LYS	466	A	<-->	11778	CA	TYR	534	B	2.73
549.	3511	CE	LYS	466	A	<-->	11779	C	TYR	534	B	2.43
550.	3511	CE	LYS	466	A	<-->	11780	O	TYR	534	B	1.90
551.	3511	CE	LYS	466	A	<-->	11781	CB	TYR	534	B	3.85
552.	3511	CE	LYS	466	A	<-->	11782	CG	TYR	534	B	3.82
553.	3511	CE	LYS	466	A	<-->	11783	CD1	TYR	534	B	3.60
554.	3511	CE	LYS	466	A	<-->	11789	N	THR	535	B	3.55
555.	3512	NZ	LYS	466	A	<-->	9416	CG	LEU	232	B	3.03
556.	3512	NZ	LYS	466	A	<-->	9418	CD2	LEU	232	B	2.07
557.	3512	NZ	LYS	466	A	<-->	11777	N	TYR	534	B	2.83
558.	3512	NZ	LYS	466	A	<-->	11778	CA	TYR	534	B	2.62
559.	3512	NZ	LYS	466	A	<-->	11779	C	TYR	534	B	1.52
560.	3512	NZ	LYS	466	A	<-->	11780	O	TYR	534	B	0.80
561.	3512	NZ	LYS	466	A	<-->	11781	CB	TYR	534	B	3.62
562.	3512	NZ	LYS	466	A	<-->	11782	CG	TYR	534	B	3.56
563.	3512	NZ	LYS	466	A	<-->	11783	CD1	TYR	534	B	3.79
564.	3512	NZ	LYS	466	A	<-->	11789	N	THR	535	B	2.37
565.	3512	NZ	LYS	466	A	<-->	11790	CA	THR	535	B	2.60
566.	3512	NZ	LYS	466	A	<-->	11793	CB	THR	535	B	2.80
567.	3512	NZ	LYS	466	A	<-->	11794	OG1	THR	535	B	3.45
568.	3521	CZ	ARG	467	A	<-->	10799	NE2	GLN	409	B	2.87
569.	3521	CZ	ARG	467	A	<-->	11765	CB	PRO	532	B	3.73
570.	3521	CZ	ARG	467	A	<-->	11766	CG	PRO	532	B	3.90
571.	3522	NH1	ARG	467	A	<-->	10797	CD	GLN	409	B	3.10
572.	3522	NH1	ARG	467	A	<-->	10798	OE1	GLN	409	B	3.62
573.	3522	NH1	ARG	467	A	<-->	10799	NE2	GLN	409	B	2.01
574.	3522	NH1	ARG	467	A	<-->	11765	CB	PRO	532	B	3.66

575.	3523	NH2	ARG	467	A	<-->	9366	CB	GLN	226	B	3.81
576.	3523	NH2	ARG	467	A	<-->	9367	CG	GLN	226	B	2.70
577.	3523	NH2	ARG	467	A	<-->	9368	CD	GLN	226	B	3.65
578.	3523	NH2	ARG	467	A	<-->	9369	OE1	GLN	226	B	3.65
579.	3523	NH2	ARG	467	A	<-->	10799	NE2	GLN	409	B	3.08
580.	3523	NH2	ARG	467	A	<-->	11765	CB	PRO	532	B	2.96
581.	3523	NH2	ARG	467	A	<-->	11766	CG	PRO	532	B	2.87
582.	3850	CB	GLU	509	A	<-->	11817	CD2	LEU	538	B	3.80
583.	3853	OE1	GLU	509	A	<-->	11815	CG	LEU	538	B	3.85
584.	3853	OE1	GLU	509	A	<-->	11816	CD1	LEU	538	B	3.59
585.	3853	OE1	GLU	509	A	<-->	11817	CD2	LEU	538	B	3.54
586.	5216	CB	GLN	690	A	<-->	9340	CZ2	TRP	223	B	3.78
587.	5216	CB	GLN	690	A	<-->	9341	CZ3	TRP	223	B	3.74
588.	5216	CB	GLN	690	A	<-->	9342	CH2	TRP	223	B	3.68
589.	5217	CG	GLN	690	A	<-->	9336	CD2	TRP	223	B	3.50
590.	5217	CG	GLN	690	A	<-->	9337	NE1	TRP	223	B	3.62
591.	5217	CG	GLN	690	A	<-->	9338	CE2	TRP	223	B	2.93
592.	5217	CG	GLN	690	A	<-->	9339	CE3	TRP	223	B	3.77
593.	5217	CG	GLN	690	A	<-->	9340	CZ2	TRP	223	B	2.61
594.	5217	CG	GLN	690	A	<-->	9341	CZ3	TRP	223	B	3.49
595.	5217	CG	GLN	690	A	<-->	9342	CH2	TRP	223	B	2.92
596.	5218	CD	GLN	690	A	<-->	9338	CE2	TRP	223	B	3.70
597.	5218	CD	GLN	690	A	<-->	9340	CZ2	TRP	223	B	2.80
598.	5218	CD	GLN	690	A	<-->	9341	CZ3	TRP	223	B	3.67
599.	5218	CD	GLN	690	A	<-->	9342	CH2	TRP	223	B	2.77
600.	5220	NE2	GLN	690	A	<-->	9338	CE2	TRP	223	B	3.55
601.	5220	NE2	GLN	690	A	<-->	9340	CZ2	TRP	223	B	2.34
602.	5220	NE2	GLN	690	A	<-->	9341	CZ3	TRP	223	B	2.94
603.	5220	NE2	GLN	690	A	<-->	9342	CH2	TRP	223	B	1.86
604.	5465	CD2	HIS	722	A	<-->	9361	NH2	ARG	225	B	3.51
605.	5491	N	PRO	726	A	<-->	9340	CZ2	TRP	223	B	2.88
606.	5491	N	PRO	726	A	<-->	9342	CH2	TRP	223	B	3.14
607.	5492	CA	PRO	726	A	<-->	9340	CZ2	TRP	223	B	3.40
608.	5492	CA	PRO	726	A	<-->	9342	CH2	TRP	223	B	3.27
609.	5493	C	PRO	726	A	<-->	9342	CH2	TRP	223	B	3.71
610.	5494	O	PRO	726	A	<-->	9342	CH2	TRP	223	B	3.23
611.	5495	CB	PRO	726	A	<-->	9336	CD2	TRP	223	B	3.87
612.	5495	CB	PRO	726	A	<-->	9338	CE2	TRP	223	B	3.41
613.	5495	CB	PRO	726	A	<-->	9339	CE3	TRP	223	B	3.78
614.	5495	CB	PRO	726	A	<-->	9340	CZ2	TRP	223	B	2.74
615.	5495	CB	PRO	726	A	<-->	9341	CZ3	TRP	223	B	3.14
616.	5495	CB	PRO	726	A	<-->	9342	CH2	TRP	223	B	2.56
617.	5496	CG	PRO	726	A	<-->	9336	CD2	TRP	223	B	2.77
618.	5496	CG	PRO	726	A	<-->	9337	NE1	TRP	223	B	3.04
619.	5496	CG	PRO	726	A	<-->	9338	CE2	TRP	223	B	2.00
620.	5496	CG	PRO	726	A	<-->	9339	CE3	TRP	223	B	2.99
621.	5496	CG	PRO	726	A	<-->	9340	CZ2	TRP	223	B	1.23
622.	5496	CG	PRO	726	A	<-->	9341	CZ3	TRP	223	B	2.48
623.	5496	CG	PRO	726	A	<-->	9342	CH2	TRP	223	B	1.59
624.	5497	CD	PRO	726	A	<-->	9336	CD2	TRP	223	B	3.90
625.	5497	CD	PRO	726	A	<-->	9337	NE1	TRP	223	B	3.30
626.	5497	CD	PRO	726	A	<-->	9338	CE2	TRP	223	B	2.70
627.	5497	CD	PRO	726	A	<-->	9340	CZ2	TRP	223	B	1.73
628.	5497	CD	PRO	726	A	<-->	9341	CZ3	TRP	223	B	3.79
629.	5497	CD	PRO	726	A	<-->	9342	CH2	TRP	223	B	2.56
630.	5700	CA	SER	755	A	<-->	9380	NH1	ARG	227	B	3.20
631.	5701	C	SER	755	A	<-->	9380	NH1	ARG	227	B	3.47
632.	5703	CB	SER	755	A	<-->	9379	CZ	ARG	227	B	2.91
633.	5703	CB	SER	755	A	<-->	9380	NH1	ARG	227	B	1.93
634.	5703	CB	SER	755	A	<-->	9381	NH2	ARG	227	B	3.35

635. 5704 OG SER 755 A <--> 9379 CZ ARG 227 B 3.72
636. 5704 OG SER 755 A <--> 9380 NH1 ARG 227 B 2.83
637. 5704 OG SER 755 A <--> 9381 NH2 ARG 227 B 3.75
638. 5705 N PRO 756 A <--> 9380 NH1 ARG 227 B 3.49
639. 5709 CB PRO 756 A <--> 9376 CG ARG 227 B 3.39
640. 5709 CB PRO 756 A <--> 9377 CD ARG 227 B 3.45
641. 5709 CB PRO 756 A <--> 9387 CG PRO 228 B 3.49
642. 5709 CB PRO 756 A <--> 9388 CD PRO 228 B 3.67
643. 5710 CG PRO 756 A <--> 9376 CG ARG 227 B 2.85
644. 5710 CG PRO 756 A <--> 9377 CD ARG 227 B 2.29
645. 5710 CG PRO 756 A <--> 9378 NE ARG 227 B 3.45
646. 5710 CG PRO 756 A <--> 9379 CZ ARG 227 B 3.82
647. 5710 CG PRO 756 A <--> 9380 NH1 ARG 227 B 3.30
648. 5711 CD PRO 756 A <--> 9377 CD ARG 227 B 3.17
649. 5711 CD PRO 756 A <--> 9378 NE ARG 227 B 3.83
650. 5711 CD PRO 756 A <--> 9379 CZ ARG 227 B 3.64
651. 5711 CD PRO 756 A <--> 9380 NH1 ARG 227 B 2.67
652. 5715 O CYS 757 A <--> 9387 CG PRO 228 B 3.42
653. 5739 CA SER 761 A <--> 9397 CZ ARG 229 B 3.84
654. 5739 CA SER 761 A <--> 9399 NH2 ARG 229 B 3.45
655. 5740 C SER 761 A <--> 9397 CZ ARG 229 B 3.27
656. 5740 C SER 761 A <--> 9398 NH1 ARG 229 B 3.34
657. 5740 C SER 761 A <--> 9399 NH2 ARG 229 B 2.62
658. 5741 O SER 761 A <--> 9399 NH2 ARG 229 B 3.14
659. 5742 CB SER 761 A <--> 9396 NE ARG 229 B 3.65
660. 5742 CB SER 761 A <--> 9397 CZ ARG 229 B 3.17
661. 5742 CB SER 761 A <--> 9398 NH1 ARG 229 B 3.62
662. 5742 CB SER 761 A <--> 9399 NH2 ARG 229 B 3.05
663. 5743 OG SER 761 A <--> 9396 NE ARG 229 B 3.29
664. 5743 OG SER 761 A <--> 9397 CZ ARG 229 B 2.97
665. 5743 OG SER 761 A <--> 9398 NH1 ARG 229 B 3.89
666. 5743 OG SER 761 A <--> 9399 NH2 ARG 229 B 2.46
667. 5744 N PRO 762 A <--> 9396 NE ARG 229 B 3.68
668. 5744 N PRO 762 A <--> 9397 CZ ARG 229 B 2.39
669. 5744 N PRO 762 A <--> 9398 NH1 ARG 229 B 2.13
670. 5744 N PRO 762 A <--> 9399 NH2 ARG 229 B 2.15
671. 5745 CA PRO 762 A <--> 9397 CZ ARG 229 B 2.67
672. 5745 CA PRO 762 A <--> 9398 NH1 ARG 229 B 2.32
673. 5745 CA PRO 762 A <--> 9399 NH2 ARG 229 B 2.33
674. 5746 C PRO 762 A <--> 9396 NE ARG 229 B 3.68
675. 5746 C PRO 762 A <--> 9397 CZ ARG 229 B 2.52
676. 5746 C PRO 762 A <--> 9398 NH1 ARG 229 B 2.84
677. 5746 C PRO 762 A <--> 9399 NH2 ARG 229 B 1.75
678. 5747 O PRO 762 A <--> 9396 NE ARG 229 B 2.76
679. 5747 O PRO 762 A <--> 9397 CZ ARG 229 B 1.87
680. 5747 O PRO 762 A <--> 9398 NH1 ARG 229 B 2.76
681. 5747 O PRO 762 A <--> 9399 NH2 ARG 229 B 0.90
682. 5748 CB PRO 762 A <--> 8150 NH2 ARG 79 B 3.49
683. 5748 CB PRO 762 A <--> 9397 CZ ARG 229 B 2.94
684. 5748 CB PRO 762 A <--> 9398 NH1 ARG 229 B 1.98
685. 5748 CB PRO 762 A <--> 9399 NH2 ARG 229 B 3.26
686. 5749 CG PRO 762 A <--> 8150 NH2 ARG 79 B 3.40
687. 5749 CG PRO 762 A <--> 9397 CZ ARG 229 B 3.40
688. 5749 CG PRO 762 A <--> 9398 NH1 ARG 229 B 2.14
689. 5750 CD PRO 762 A <--> 9397 CZ ARG 229 B 2.84
690. 5750 CD PRO 762 A <--> 9398 NH1 ARG 229 B 1.97
691. 5750 CD PRO 762 A <--> 9399 NH2 ARG 229 B 3.23
692. 5751 N GLN 763 A <--> 9397 CZ ARG 229 B 3.79
693. 5751 N GLN 763 A <--> 9399 NH2 ARG 229 B 3.00
694. 5752 CA GLN 763 A <--> 9399 NH2 ARG 229 B 3.66

```
695.  5761  CA  ALA  764   A  <-->  8168  CD2 LEU   81   B     3.15
696.  5762  C   ALA  764   A  <-->  8168  CD2 LEU   81   B     3.63
697.  5763  O   ALA  764   A  <-->  8168  CD2 LEU   81   B     3.24
698.  5764  CB  ALA  764   A  <-->  8168  CD2 LEU   81   B     3.76

Salt bridges
------------

     <----- A T O M  1 ----->     <----- A T O M  2 ----->

     Atom Atom Res  Res           Atom Atom Res  Res
      no. name name no.  Chain      no. name name no.  Chain  Distance
  1.  3406  NE2 HIS  453   A  <--> 12903  OE1 GLU  677   B     3.03

Number of salt bridges:          1

Number of hydrogen bonds:        8

Number of non-bonded contacts:   698
```

[C] D312V + SLIT2 (WT) _FULL Protein

Hydrogen bonds

<----- A T O M 1 -----> <----- A T O M 2 ----->

	Atom no.	Atom name	Res name	Res no.	Chain		Atom no.	Atom name	Res name	Res no.	Chain	Distance
1.	550	OG1	THR	57	A	<-->	21431	OG	SER	1114	B	2.33
2.	1047	NH1	ARG	107	A	<-->	17772	OH	TYR	638	B	2.55
3.	1071	OE2	GLU	109	A	<-->	16551	NE2	GLN	483	B	2.61
4.	1078	OG1	THR	110	A	<-->	16941	NE2	GLN	533	B	1.77
5.	1085	O	ASP	111	A	<-->	16506	NH1	ARG	477	B	2.18
6.	1085	O	ASP	111	A	<-->	16507	NH2	ARG	477	B	3.16
7.	1088	OD1	ASP	111	A	<-->	16506	NH1	ARG	477	B	1.22
8.	1099	NZ	LYS	112	A	<-->	16945	O	TYR	534	B	3.12
9.	1151	OG	SER	117	A	<-->	19731	O	PRO	889	B	2.56
10.	2526	OG1	THR	257	A	<-->	19296	N	LEU	832	B	3.01
11.	2541	O	LEU	259	A	<-->	16268	OG1	THR	447	B	3.27
12.	2555	OE2	GLU	260	A	<-->	16268	OG1	THR	447	B	2.95

Non-bonded contacts

<----- A T O M 1 -----> <----- A T O M 2 ----->

	Atom no.	Atom name	Res name	Res no.	Chain		Atom no.	Atom name	Res name	Res no.	Chain	Distance
1.	529	C	TYR	56	A	<-->	21796	N	ASN	1163	B	3.81
2.	530	O	TYR	56	A	<-->	21431	OG	SER	1114	B	3.89
3.	530	O	TYR	56	A	<-->	21796	N	ASN	1163	B	3.80
4.	534	CD2	TYR	56	A	<-->	21796	N	ASN	1163	B	3.86
5.	534	CD2	TYR	56	A	<-->	21800	CB	ASN	1163	B	3.60
6.	536	CE2	TYR	56	A	<-->	21796	N	ASN	1163	B	3.87
7.	536	CE2	TYR	56	A	<-->	21800	CB	ASN	1163	B	3.65
8.	545	N	THR	57	A	<-->	21790	CA	VAL	1162	B	2.93
9.	545	N	THR	57	A	<-->	21791	C	VAL	1162	B	3.58
10.	545	N	THR	57	A	<-->	21793	CB	VAL	1162	B	3.12
11.	545	N	THR	57	A	<-->	21794	CG1	VAL	1162	B	3.41
12.	545	N	THR	57	A	<-->	21796	N	ASN	1163	B	3.25
13.	546	CA	THR	57	A	<-->	21785	C	SER	1161	B	3.74
14.	546	CA	THR	57	A	<-->	21786	O	SER	1161	B	3.60
15.	546	CA	THR	57	A	<-->	21789	N	VAL	1162	B	3.03
16.	546	CA	THR	57	A	<-->	21790	CA	VAL	1162	B	1.64
17.	546	CA	THR	57	A	<-->	21791	C	VAL	1162	B	2.20
18.	546	CA	THR	57	A	<-->	21792	O	VAL	1162	B	3.38
19.	546	CA	THR	57	A	<-->	21793	CB	VAL	1162	B	1.91
20.	546	CA	THR	57	A	<-->	21794	CG1	VAL	1162	B	2.75
21.	546	CA	THR	57	A	<-->	21795	CG2	VAL	1162	B	3.25
22.	546	CA	THR	57	A	<-->	21796	N	ASN	1163	B	2.19
23.	546	CA	THR	57	A	<-->	21797	CA	ASN	1163	B	3.57
24.	546	CA	THR	57	A	<-->	21799	O	ASN	1163	B	3.89
25.	547	C	THR	57	A	<-->	21785	C	SER	1161	B	2.84
26.	547	C	THR	57	A	<-->	21786	O	SER	1161	B	2.84
27.	547	C	THR	57	A	<-->	21789	N	VAL	1162	B	2.24
28.	547	C	THR	57	A	<-->	21790	CA	VAL	1162	B	1.30
29.	547	C	THR	57	A	<-->	21791	C	VAL	1162	B	1.61

30.	547	C	THR	57	A	<-->	21792	O	VAL	1162	B	2.73
31.	547	C	THR	57	A	<-->	21793	CB	VAL	1162	B	2.59
32.	547	C	THR	57	A	<-->	21794	CG1	VAL	1162	B	3.47
33.	547	C	THR	57	A	<-->	21795	CG2	VAL	1162	B	3.62
34.	547	C	THR	57	A	<-->	21796	N	ASN	1163	B	1.85
35.	547	C	THR	57	A	<-->	21797	CA	ASN	1163	B	3.20
36.	548	O	THR	57	A	<-->	21784	CA	SER	1161	B	3.65
37.	548	O	THR	57	A	<-->	21785	C	SER	1161	B	2.31
38.	548	O	THR	57	A	<-->	21786	O	SER	1161	B	1.95
39.	548	O	THR	57	A	<-->	21789	N	VAL	1162	B	2.36
40.	548	O	THR	57	A	<-->	21790	CA	VAL	1162	B	2.04
41.	548	O	THR	57	A	<-->	21791	C	VAL	1162	B	2.71
42.	548	O	THR	57	A	<-->	21792	O	VAL	1162	B	3.64
43.	548	O	THR	57	A	<-->	21793	CB	VAL	1162	B	3.37
44.	548	O	THR	57	A	<-->	21794	CG1	VAL	1162	B	3.81
45.	548	O	THR	57	A	<-->	21796	N	ASN	1163	B	2.98
46.	549	CB	THR	57	A	<-->	21418	O	PHE	1113	B	3.63
47.	549	CB	THR	57	A	<-->	21430	CB	SER	1114	B	3.45
48.	549	CB	THR	57	A	<-->	21431	OG	SER	1114	B	3.71
49.	549	CB	THR	57	A	<-->	21785	C	SER	1161	B	3.54
50.	549	CB	THR	57	A	<-->	21786	O	SER	1161	B	3.59
51.	549	CB	THR	57	A	<-->	21789	N	VAL	1162	B	2.78
52.	549	CB	THR	57	A	<-->	21790	CA	VAL	1162	B	1.70
53.	549	CB	THR	57	A	<-->	21791	C	VAL	1162	B	2.76
54.	549	CB	THR	57	A	<-->	21792	O	VAL	1162	B	3.63
55.	549	CB	THR	57	A	<-->	21793	CB	VAL	1162	B	0.78
56.	549	CB	THR	57	A	<-->	21794	CG1	VAL	1162	B	1.32
57.	549	CB	THR	57	A	<-->	21795	CG2	VAL	1162	B	2.29
58.	549	CB	THR	57	A	<-->	21796	N	ASN	1163	B	3.32
59.	550	OG1	THR	57	A	<-->	21418	O	PHE	1113	B	3.17
60.	550	OG1	THR	57	A	<-->	21427	CA	SER	1114	B	3.52
61.	550	OG1	THR	57	A	<-->	21430	CB	SER	1114	B	2.10
62.	550	OG1	THR	57	A	<-->	21431	OG	SER	1114	B	2.33
63.	550	OG1	THR	57	A	<-->	21438	CD	PRO	1115	B	3.89
64.	550	OG1	THR	57	A	<-->	21790	CA	VAL	1162	B	3.11
65.	550	OG1	THR	57	A	<-->	21793	CB	VAL	1162	B	1.93
66.	550	OG1	THR	57	A	<-->	21794	CG1	VAL	1162	B	1.85
67.	550	OG1	THR	57	A	<-->	21795	CG2	VAL	1162	B	3.04
68.	551	CG2	THR	57	A	<-->	21418	O	PHE	1113	B	2.90
69.	551	CG2	THR	57	A	<-->	21430	CB	SER	1114	B	3.89
70.	551	CG2	THR	57	A	<-->	21785	C	SER	1161	B	3.73
71.	551	CG2	THR	57	A	<-->	21789	N	VAL	1162	B	2.59
72.	551	CG2	THR	57	A	<-->	21790	CA	VAL	1162	B	1.91
73.	551	CG2	THR	57	A	<-->	21791	C	VAL	1162	B	2.47
74.	551	CG2	THR	57	A	<-->	21792	O	VAL	1162	B	2.83
75.	551	CG2	THR	57	A	<-->	21793	CB	VAL	1162	B	0.78
76.	551	CG2	THR	57	A	<-->	21794	CG1	VAL	1162	B	1.99
77.	551	CG2	THR	57	A	<-->	21795	CG2	VAL	1162	B	0.76
78.	551	CG2	THR	57	A	<-->	21796	N	ASN	1163	B	3.41
79.	554	N	GLY	58	A	<-->	21785	C	SER	1161	B	3.40
80.	554	N	GLY	58	A	<-->	21786	O	SER	1161	B	3.79
81.	554	N	GLY	58	A	<-->	21789	N	VAL	1162	B	2.48
82.	554	N	GLY	58	A	<-->	21790	CA	VAL	1162	B	1.81
83.	554	N	GLY	58	A	<-->	21791	C	VAL	1162	B	0.82
84.	554	N	GLY	58	A	<-->	21792	O	VAL	1162	B	1.65
85.	554	N	GLY	58	A	<-->	21793	CB	VAL	1162	B	3.01
86.	554	N	GLY	58	A	<-->	21795	CG2	VAL	1162	B	3.50
87.	554	N	GLY	58	A	<-->	21796	N	ASN	1163	B	1.23
88.	554	N	GLY	58	A	<-->	21797	CA	ASN	1163	B	2.23
89.	554	N	GLY	58	A	<-->	21798	C	ASN	1163	B	3.09

90.	554	N	GLY	58	A	<-->	21799	O	ASN	1163	B	3.62
91.	554	N	GLY	58	A	<-->	21800	CB	ASN	1163	B	3.46
92.	554	N	GLY	58	A	<-->	21804	N	PHE	1164	B	3.87
93.	555	CA	GLY	58	A	<-->	21785	C	SER	1161	B	3.55
94.	555	CA	GLY	58	A	<-->	21789	N	VAL	1162	B	2.86
95.	555	CA	GLY	58	A	<-->	21790	CA	VAL	1162	B	2.90
96.	555	CA	GLY	58	A	<-->	21791	C	VAL	1162	B	2.04
97.	555	CA	GLY	58	A	<-->	21792	O	VAL	1162	B	1.99
98.	555	CA	GLY	58	A	<-->	21796	N	ASN	1163	B	2.47
99.	555	CA	GLY	58	A	<-->	21797	CA	ASN	1163	B	2.83
100.	555	CA	GLY	58	A	<-->	21798	C	ASN	1163	B	3.75
101.	556	C	GLY	58	A	<-->	21791	C	VAL	1162	B	3.22
102.	556	C	GLY	58	A	<-->	21792	O	VAL	1162	B	3.38
103.	556	C	GLY	58	A	<-->	21796	N	ASN	1163	B	3.04
104.	556	C	GLY	58	A	<-->	21797	CA	ASN	1163	B	3.06
105.	556	C	GLY	58	A	<-->	21800	CB	ASN	1163	B	3.77
106.	557	O	GLY	58	A	<-->	21791	C	VAL	1162	B	3.79
107.	557	O	GLY	58	A	<-->	21796	N	ASN	1163	B	3.47
108.	557	O	GLY	58	A	<-->	21797	CA	ASN	1163	B	3.75
109.	559	N	SER	59	A	<-->	21796	N	ASN	1163	B	3.63
110.	559	N	SER	59	A	<-->	21797	CA	ASN	1163	B	3.09
111.	559	N	SER	59	A	<-->	21800	CB	ASN	1163	B	3.59
112.	559	N	SER	59	A	<-->	21801	CG	ASN	1163	B	3.67
113.	559	N	SER	59	A	<-->	21803	ND2	ASN	1163	B	3.90
114.	560	CA	SER	59	A	<-->	21803	ND2	ASN	1163	B	3.63
115.	562	O	SER	59	A	<-->	21645	OE2	GLU	1142	B	2.86
116.	563	CB	SER	59	A	<-->	21800	CB	ASN	1163	B	3.78
117.	563	CB	SER	59	A	<-->	21801	CG	ASN	1163	B	3.05
118.	563	CB	SER	59	A	<-->	21802	OD1	ASN	1163	B	3.66
119.	563	CB	SER	59	A	<-->	21803	ND2	ASN	1163	B	2.41
120.	564	OG	SER	59	A	<-->	21801	CG	ASN	1163	B	3.00
121.	564	OG	SER	59	A	<-->	21802	OD1	ASN	1163	B	3.10
122.	564	OG	SER	59	A	<-->	21803	ND2	ASN	1163	B	2.69
123.	574	NE	ARG	60	A	<-->	21640	O	GLU	1142	B	3.68
124.	575	CZ	ARG	60	A	<-->	21639	C	GLU	1142	B	3.65
125.	575	CZ	ARG	60	A	<-->	21640	O	GLU	1142	B	3.24
126.	575	CZ	ARG	60	A	<-->	21642	CG	GLU	1142	B	3.73
127.	575	CZ	ARG	60	A	<-->	21646	N	PRO	1143	B	3.74
128.	575	CZ	ARG	60	A	<-->	21647	CA	PRO	1143	B	3.47
129.	576	NH1	ARG	60	A	<-->	21642	CG	GLU	1142	B	3.29
130.	577	NH2	ARG	60	A	<-->	21639	C	GLU	1142	B	2.86
131.	577	NH2	ARG	60	A	<-->	21640	O	GLU	1142	B	2.75
132.	577	NH2	ARG	60	A	<-->	21642	CG	GLU	1142	B	3.84
133.	577	NH2	ARG	60	A	<-->	21646	N	PRO	1143	B	2.59
134.	577	NH2	ARG	60	A	<-->	21647	CA	PRO	1143	B	2.20
135.	577	NH2	ARG	60	A	<-->	21648	C	PRO	1143	B	3.42
136.	577	NH2	ARG	60	A	<-->	21650	CB	PRO	1143	B	2.86
137.	577	NH2	ARG	60	A	<-->	21651	CG	PRO	1143	B	3.56
138.	577	NH2	ARG	60	A	<-->	21652	CD	PRO	1143	B	3.06
139.	919	CB	PRO	95	A	<-->	19712	NH2	ARG	885	B	3.77
140.	1044	CD	ARG	107	A	<-->	17772	OH	TYR	638	B	3.27
141.	1045	NE	ARG	107	A	<-->	17772	OH	TYR	638	B	3.59
142.	1046	CZ	ARG	107	A	<-->	17772	OH	TYR	638	B	3.33
143.	1047	NH1	ARG	107	A	<-->	17770	CE2	TYR	638	B	3.54
144.	1047	NH1	ARG	107	A	<-->	17771	CZ	TYR	638	B	3.34
145.	1047	NH1	ARG	107	A	<-->	17772	OH	TYR	638	B	2.55
146.	1064	CA	GLU	109	A	<-->	17768	CD2	TYR	638	B	3.84
147.	1065	C	GLU	109	A	<-->	16540	CD	GLU	482	B	3.82
148.	1065	C	GLU	109	A	<-->	16542	OE2	GLU	482	B	2.72
149.	1066	O	GLU	109	A	<-->	16540	CD	GLU	482	B	2.94

150.	1066	O	GLU	109	A	<-->	16541	OE1	GLU	482	B	3.49
151.	1066	O	GLU	109	A	<-->	16542	OE2	GLU	482	B	2.10
152.	1066	O	GLU	109	A	<-->	16551	NE2	GLN	483	B	3.25
153.	1067	CB	GLU	109	A	<-->	17765	CB	TYR	638	B	3.29
154.	1067	CB	GLU	109	A	<-->	17766	CG	TYR	638	B	2.78
155.	1067	CB	GLU	109	A	<-->	17767	CD1	TYR	638	B	3.58
156.	1067	CB	GLU	109	A	<-->	17768	CD2	TYR	638	B	2.34
157.	1067	CB	GLU	109	A	<-->	17770	CE2	TYR	638	B	2.88
158.	1067	CB	GLU	109	A	<-->	17771	CZ	TYR	638	B	3.65
159.	1068	CG	GLU	109	A	<-->	16551	NE2	GLN	483	B	3.56
160.	1068	CG	GLU	109	A	<-->	17762	CA	TYR	638	B	3.21
161.	1068	CG	GLU	109	A	<-->	17765	CB	TYR	638	B	1.77
162.	1068	CG	GLU	109	A	<-->	17766	CG	TYR	638	B	1.50
163.	1068	CG	GLU	109	A	<-->	17767	CD1	TYR	638	B	2.53
164.	1068	CG	GLU	109	A	<-->	17768	CD2	TYR	638	B	1.75
165.	1068	CG	GLU	109	A	<-->	17769	CE1	TYR	638	B	3.37
166.	1068	CG	GLU	109	A	<-->	17770	CE2	TYR	638	B	2.83
167.	1068	CG	GLU	109	A	<-->	17771	CZ	TYR	638	B	3.49
168.	1069	CD	GLU	109	A	<-->	16540	CD	GLU	482	B	3.89
169.	1069	CD	GLU	109	A	<-->	16542	OE2	GLU	482	B	3.02
170.	1069	CD	GLU	109	A	<-->	16551	NE2	GLN	483	B	3.33
171.	1069	CD	GLU	109	A	<-->	17755	C	LEU	637	B	3.47
172.	1069	CD	GLU	109	A	<-->	17756	O	LEU	637	B	3.68
173.	1069	CD	GLU	109	A	<-->	17761	N	TYR	638	B	3.05
174.	1069	CD	GLU	109	A	<-->	17762	CA	TYR	638	B	2.77
175.	1069	CD	GLU	109	A	<-->	17765	CB	TYR	638	B	1.59
176.	1069	CD	GLU	109	A	<-->	17766	CG	TYR	638	B	2.45
177.	1069	CD	GLU	109	A	<-->	17767	CD1	TYR	638	B	3.64
178.	1069	CD	GLU	109	A	<-->	17768	CD2	TYR	638	B	2.79
179.	1070	OE1	GLU	109	A	<-->	16540	CD	GLU	482	B	3.88
180.	1070	OE1	GLU	109	A	<-->	16542	OE2	GLU	482	B	2.81
181.	1070	OE1	GLU	109	A	<-->	17755	C	LEU	637	B	3.72
182.	1070	OE1	GLU	109	A	<-->	17756	O	LEU	637	B	3.82
183.	1070	OE1	GLU	109	A	<-->	17761	N	TYR	638	B	3.71
184.	1070	OE1	GLU	109	A	<-->	17762	CA	TYR	638	B	3.73
185.	1070	OE1	GLU	109	A	<-->	17765	CB	TYR	638	B	2.78
186.	1070	OE1	GLU	109	A	<-->	17766	CG	TYR	638	B	3.50
187.	1070	OE1	GLU	109	A	<-->	17768	CD2	TYR	638	B	3.43
188.	1071	OE2	GLU	109	A	<-->	16539	CG	GLU	482	B	3.21
189.	1071	OE2	GLU	109	A	<-->	16540	CD	GLU	482	B	3.55
190.	1071	OE2	GLU	109	A	<-->	16542	OE2	GLU	482	B	3.07
191.	1071	OE2	GLU	109	A	<-->	16549	CD	GLN	483	B	3.79
192.	1071	OE2	GLU	109	A	<-->	16551	NE2	GLN	483	B	2.61
193.	1071	OE2	GLU	109	A	<-->	17754	CA	LEU	637	B	3.75
194.	1071	OE2	GLU	109	A	<-->	17755	C	LEU	637	B	2.92
195.	1071	OE2	GLU	109	A	<-->	17756	O	LEU	637	B	3.50
196.	1071	OE2	GLU	109	A	<-->	17757	CB	LEU	637	B	3.85
197.	1071	OE2	GLU	109	A	<-->	17761	N	TYR	638	B	2.13
198.	1071	OE2	GLU	109	A	<-->	17762	CA	TYR	638	B	2.11
199.	1071	OE2	GLU	109	A	<-->	17763	C	TYR	638	B	3.13
200.	1071	OE2	GLU	109	A	<-->	17764	O	TYR	638	B	3.25
201.	1071	OE2	GLU	109	A	<-->	17765	CB	TYR	638	B	1.32
202.	1071	OE2	GLU	109	A	<-->	17766	CG	TYR	638	B	2.73
203.	1071	OE2	GLU	109	A	<-->	17767	CD1	TYR	638	B	3.71
204.	1071	OE2	GLU	109	A	<-->	17768	CD2	TYR	638	B	3.50
205.	1073	N	THR	110	A	<-->	16542	OE2	GLU	482	B	2.91
206.	1074	CA	THR	110	A	<-->	16540	CD	GLU	482	B	3.34
207.	1074	CA	THR	110	A	<-->	16541	OE1	GLU	482	B	3.60
208.	1074	CA	THR	110	A	<-->	16542	OE2	GLU	482	B	2.41
209.	1074	CA	THR	110	A	<-->	16941	NE2	GLN	533	B	3.84

210.	1075	C	THR	110	A	<-->	16542	OE2	GLU	482	B	3.45
211.	1077	CB	THR	110	A	<-->	16540	CD	GLU	482	B	3.75
212.	1077	CB	THR	110	A	<-->	16541	OE1	GLU	482	B	3.47
213.	1077	CB	THR	110	A	<-->	16542	OE2	GLU	482	B	3.24
214.	1077	CB	THR	110	A	<-->	16939	CD	GLN	533	B	3.87
215.	1077	CB	THR	110	A	<-->	16941	NE2	GLN	533	B	2.57
216.	1078	OG1	THR	110	A	<-->	16540	CD	GLU	482	B	3.76
217.	1078	OG1	THR	110	A	<-->	16541	OE1	GLU	482	B	3.21
218.	1078	OG1	THR	110	A	<-->	16542	OE2	GLU	482	B	3.63
219.	1078	OG1	THR	110	A	<-->	16938	CG	GLN	533	B	3.18
220.	1078	OG1	THR	110	A	<-->	16939	CD	GLN	533	B	2.81
221.	1078	OG1	THR	110	A	<-->	16941	NE2	GLN	533	B	1.77
222.	1079	CG2	THR	110	A	<-->	16941	NE2	GLN	533	B	3.33
223.	1082	N	ASP	111	A	<-->	16542	OE2	GLU	482	B	3.72
224.	1083	CA	ASP	111	A	<-->	16506	NH1	ARG	477	B	3.33
225.	1083	CA	ASP	111	A	<-->	16511	O	CYS	478	B	3.51
226.	1083	CA	ASP	111	A	<-->	16515	CA	SER	479	B	3.65
227.	1084	C	ASP	111	A	<-->	16505	CZ	ARG	477	B	3.80
228.	1084	C	ASP	111	A	<-->	16506	NH1	ARG	477	B	2.71
229.	1085	O	ASP	111	A	<-->	16505	CZ	ARG	477	B	3.07
230.	1085	O	ASP	111	A	<-->	16506	NH1	ARG	477	B	2.18
231.	1085	O	ASP	111	A	<-->	16507	NH2	ARG	477	B	3.16
232.	1086	CB	ASP	111	A	<-->	14583	CD2	LEU	232	B	3.43
233.	1086	CB	ASP	111	A	<-->	16506	NH1	ARG	477	B	3.28
234.	1086	CB	ASP	111	A	<-->	16511	O	CYS	478	B	3.61
235.	1086	CB	ASP	111	A	<-->	16514	N	SER	479	B	3.79
236.	1086	CB	ASP	111	A	<-->	16515	CA	SER	479	B	2.63
237.	1086	CB	ASP	111	A	<-->	16516	C	SER	479	B	3.33
238.	1086	CB	ASP	111	A	<-->	16518	CB	SER	479	B	2.93
239.	1086	CB	ASP	111	A	<-->	16519	OG	SER	479	B	2.41
240.	1086	CB	ASP	111	A	<-->	16520	N	ALA	480	B	3.12
241.	1086	CB	ASP	111	A	<-->	16959	OG1	THR	535	B	3.83
242.	1087	CG	ASP	111	A	<-->	16503	CD	ARG	477	B	3.52
243.	1087	CG	ASP	111	A	<-->	16505	CZ	ARG	477	B	3.63
244.	1087	CG	ASP	111	A	<-->	16506	NH1	ARG	477	B	2.42
245.	1087	CG	ASP	111	A	<-->	16510	C	CYS	478	B	3.40
246.	1087	CG	ASP	111	A	<-->	16511	O	CYS	478	B	3.20
247.	1087	CG	ASP	111	A	<-->	16514	N	SER	479	B	3.11
248.	1087	CG	ASP	111	A	<-->	16515	CA	SER	479	B	2.28
249.	1087	CG	ASP	111	A	<-->	16516	C	SER	479	B	3.52
250.	1087	CG	ASP	111	A	<-->	16518	CB	SER	479	B	2.19
251.	1087	CG	ASP	111	A	<-->	16519	OG	SER	479	B	2.14
252.	1087	CG	ASP	111	A	<-->	16520	N	ALA	480	B	3.83
253.	1087	CG	ASP	111	A	<-->	16958	CB	THR	535	B	3.81
254.	1087	CG	ASP	111	A	<-->	16959	OG1	THR	535	B	3.24
255.	1088	OD1	ASP	111	A	<-->	16501	CB	ARG	477	B	3.62
256.	1088	OD1	ASP	111	A	<-->	16502	CG	ARG	477	B	3.51
257.	1088	OD1	ASP	111	A	<-->	16503	CD	ARG	477	B	2.74
258.	1088	OD1	ASP	111	A	<-->	16504	NE	ARG	477	B	2.94
259.	1088	OD1	ASP	111	A	<-->	16505	CZ	ARG	477	B	2.39
260.	1088	OD1	ASP	111	A	<-->	16506	NH1	ARG	477	B	1.22
261.	1088	OD1	ASP	111	A	<-->	16507	NH2	ARG	477	B	3.55
262.	1088	OD1	ASP	111	A	<-->	16511	O	CYS	478	B	3.66
263.	1088	OD1	ASP	111	A	<-->	16515	CA	SER	479	B	3.42
264.	1088	OD1	ASP	111	A	<-->	16518	CB	SER	479	B	3.20
265.	1088	OD1	ASP	111	A	<-->	16519	OG	SER	479	B	3.11
266.	1088	OD1	ASP	111	A	<-->	16958	CB	THR	535	B	3.87
267.	1088	OD1	ASP	111	A	<-->	16959	OG1	THR	535	B	2.88
268.	1089	OD2	ASP	111	A	<-->	16503	CD	ARG	477	B	3.66
269.	1089	OD2	ASP	111	A	<-->	16506	NH1	ARG	477	B	3.41

270.	1089	OD2	ASP	111	A	<-->	16510	C	CYS	478	B	2.89
271.	1089	OD2	ASP	111	A	<-->	16511	O	CYS	478	B	3.15
272.	1089	OD2	ASP	111	A	<-->	16514	N	SER	479	B	2.21
273.	1089	OD2	ASP	111	A	<-->	16515	CA	SER	479	B	1.48
274.	1089	OD2	ASP	111	A	<-->	16516	C	SER	479	B	2.83
275.	1089	OD2	ASP	111	A	<-->	16517	O	SER	479	B	3.69
276.	1089	OD2	ASP	111	A	<-->	16518	CB	SER	479	B	1.11
277.	1089	OD2	ASP	111	A	<-->	16519	OG	SER	479	B	1.83
278.	1089	OD2	ASP	111	A	<-->	16520	N	ALA	480	B	3.47
279.	1089	OD2	ASP	111	A	<-->	16959	OG1	THR	535	B	3.80
280.	1091	N	LYS	112	A	<-->	16506	NH1	ARG	477	B	3.63
281.	1096	CG	LYS	112	A	<-->	16945	O	TYR	534	B	3.13
282.	1096	CG	LYS	112	A	<-->	16948	CD1	TYR	534	B	3.71
283.	1096	CG	LYS	112	A	<-->	16950	CE1	TYR	534	B	3.22
284.	1096	CG	LYS	112	A	<-->	16952	CZ	TYR	534	B	3.34
285.	1096	CG	LYS	112	A	<-->	16953	OH	TYR	534	B	3.68
286.	1097	CD	LYS	112	A	<-->	16945	O	TYR	534	B	3.58
287.	1097	CD	LYS	112	A	<-->	16947	CG	TYR	534	B	3.62
288.	1097	CD	LYS	112	A	<-->	16948	CD1	TYR	534	B	2.71
289.	1097	CD	LYS	112	A	<-->	16949	CD2	TYR	534	B	3.88
290.	1097	CD	LYS	112	A	<-->	16950	CE1	TYR	534	B	1.91
291.	1097	CD	LYS	112	A	<-->	16951	CE2	TYR	534	B	3.37
292.	1097	CD	LYS	112	A	<-->	16952	CZ	TYR	534	B	2.38
293.	1097	CD	LYS	112	A	<-->	16953	OH	TYR	534	B	2.79
294.	1098	CE	LYS	112	A	<-->	16942	N	TYR	534	B	3.67
295.	1098	CE	LYS	112	A	<-->	16943	CA	TYR	534	B	3.78
296.	1098	CE	LYS	112	A	<-->	16944	C	TYR	534	B	3.69
297.	1098	CE	LYS	112	A	<-->	16945	O	TYR	534	B	2.96
298.	1098	CE	LYS	112	A	<-->	16946	CB	TYR	534	B	3.41
299.	1098	CE	LYS	112	A	<-->	16947	CG	TYR	534	B	2.12
300.	1098	CE	LYS	112	A	<-->	16948	CD1	TYR	534	B	1.24
301.	1098	CE	LYS	112	A	<-->	16949	CD2	TYR	534	B	2.61
302.	1098	CE	LYS	112	A	<-->	16950	CE1	TYR	534	B	0.95
303.	1098	CE	LYS	112	A	<-->	16951	CE2	TYR	534	B	2.49
304.	1098	CE	LYS	112	A	<-->	16952	CZ	TYR	534	B	1.81
305.	1098	CE	LYS	112	A	<-->	16953	OH	TYR	534	B	2.92
306.	1099	NZ	LYS	112	A	<-->	16944	C	TYR	534	B	3.67
307.	1099	NZ	LYS	112	A	<-->	16945	O	TYR	534	B	3.12
308.	1099	NZ	LYS	112	A	<-->	16946	CB	TYR	534	B	3.30
309.	1099	NZ	LYS	112	A	<-->	16947	CG	TYR	534	B	1.86
310.	1099	NZ	LYS	112	A	<-->	16948	CD1	TYR	534	B	1.93
311.	1099	NZ	LYS	112	A	<-->	16949	CD2	TYR	534	B	1.46
312.	1099	NZ	LYS	112	A	<-->	16950	CE1	TYR	534	B	1.66
313.	1099	NZ	LYS	112	A	<-->	16951	CE2	TYR	534	B	1.09
314.	1099	NZ	LYS	112	A	<-->	16952	CZ	TYR	534	B	1.22
315.	1099	NZ	LYS	112	A	<-->	16953	OH	TYR	534	B	2.52
316.	1135	CD	ARG	116	A	<-->	19749	CA	MET	892	B	3.62
317.	1135	CD	ARG	116	A	<-->	19752	CB	MET	892	B	2.69
318.	1135	CD	ARG	116	A	<-->	19753	CG	MET	892	B	3.83
319.	1136	NE	ARG	116	A	<-->	19748	N	MET	892	B	3.27
320.	1136	NE	ARG	116	A	<-->	19749	CA	MET	892	B	2.78
321.	1136	NE	ARG	116	A	<-->	19752	CB	MET	892	B	1.70
322.	1136	NE	ARG	116	A	<-->	19753	CG	MET	892	B	2.66
323.	1137	CZ	ARG	116	A	<-->	19741	C	GLU	891	B	3.75
324.	1137	CZ	ARG	116	A	<-->	19748	N	MET	892	B	2.45
325.	1137	CZ	ARG	116	A	<-->	19749	CA	MET	892	B	1.74
326.	1137	CZ	ARG	116	A	<-->	19750	C	MET	892	B	2.83
327.	1137	CZ	ARG	116	A	<-->	19751	O	MET	892	B	3.62
328.	1137	CZ	ARG	116	A	<-->	19752	CB	MET	892	B	0.45
329.	1137	CZ	ARG	116	A	<-->	19753	CG	MET	892	B	1.61

330. 1137 CZ ARG 116 A <--> 19754 SD MET 892 B 3.13
331. 1137 CZ ARG 116 A <--> 19755 CE MET 892 B 3.49
332. 1137 CZ ARG 116 A <--> 19756 N ALA 893 B 3.48
333. 1138 NH1 ARG 116 A <--> 19748 N MET 892 B 2.92
334. 1138 NH1 ARG 116 A <--> 19749 CA MET 892 B 1.63
335. 1138 NH1 ARG 116 A <--> 19750 C MET 892 B 2.15
336. 1138 NH1 ARG 116 A <--> 19751 O MET 892 B 2.58
337. 1138 NH1 ARG 116 A <--> 19752 CB MET 892 B 0.91
338. 1138 NH1 ARG 116 A <--> 19753 CG MET 892 B 2.01
339. 1138 NH1 ARG 116 A <--> 19754 SD MET 892 B 2.72
340. 1138 NH1 ARG 116 A <--> 19755 CE MET 892 B 2.88
341. 1138 NH1 ARG 116 A <--> 19756 N ALA 893 B 3.10
342. 1139 NH2 ARG 116 A <--> 19741 C GLU 891 B 3.23
343. 1139 NH2 ARG 116 A <--> 19748 N MET 892 B 1.91
344. 1139 NH2 ARG 116 A <--> 19749 CA MET 892 B 1.93
345. 1139 NH2 ARG 116 A <--> 19750 C MET 892 B 2.81
346. 1139 NH2 ARG 116 A <--> 19752 CB MET 892 B 1.48
347. 1139 NH2 ARG 116 A <--> 19753 CG MET 892 B 1.45
348. 1139 NH2 ARG 116 A <--> 19754 SD MET 892 B 3.08
349. 1139 NH2 ARG 116 A <--> 19756 N ALA 893 B 2.97
350. 1146 N SER 117 A <--> 19739 N GLU 891 B 3.77
351. 1147 CA SER 117 A <--> 19736 CA GLY 890 B 3.44
352. 1147 CA SER 117 A <--> 19737 C GLY 890 B 3.63
353. 1147 CA SER 117 A <--> 19739 N GLU 891 B 3.01
354. 1148 C SER 117 A <--> 19736 CA GLY 890 B 3.27
355. 1148 C SER 117 A <--> 19737 C GLY 890 B 3.08
356. 1148 C SER 117 A <--> 19739 N GLU 891 B 2.09
357. 1148 C SER 117 A <--> 19740 CA GLU 891 B 2.91
358. 1148 C SER 117 A <--> 19741 C GLU 891 B 3.50
359. 1149 O SER 117 A <--> 19736 CA GLY 890 B 3.84
360. 1149 O SER 117 A <--> 19737 C GLY 890 B 3.15
361. 1149 O SER 117 A <--> 19739 N GLU 891 B 1.85
362. 1149 O SER 117 A <--> 19740 CA GLU 891 B 2.01
363. 1149 O SER 117 A <--> 19741 C GLU 891 B 2.58
364. 1149 O SER 117 A <--> 19742 O GLU 891 B 3.38
365. 1149 O SER 117 A <--> 19743 CB GLU 891 B 3.48
366. 1149 O SER 117 A <--> 19748 N MET 892 B 2.98
367. 1150 CB SER 117 A <--> 19730 C PRO 889 B 3.69
368. 1150 CB SER 117 A <--> 19731 O PRO 889 B 3.11
369. 1150 CB SER 117 A <--> 19735 N GLY 890 B 3.50
370. 1150 CB SER 117 A <--> 19736 CA GLY 890 B 2.50
371. 1150 CB SER 117 A <--> 19737 C GLY 890 B 2.97
372. 1150 CB SER 117 A <--> 19739 N GLU 891 B 2.95
373. 1150 CB SER 117 A <--> 19742 O GLU 891 B 3.79
374. 1151 OG SER 117 A <--> 19730 C PRO 889 B 3.22
375. 1151 OG SER 117 A <--> 19731 O PRO 889 B 2.56
376. 1151 OG SER 117 A <--> 19735 N GLY 890 B 3.24
377. 1151 OG SER 117 A <--> 19736 CA GLY 890 B 2.44
378. 1151 OG SER 117 A <--> 19737 C GLY 890 B 2.28
379. 1151 OG SER 117 A <--> 19738 O GLY 890 B 2.99
380. 1151 OG SER 117 A <--> 19739 N GLU 891 B 2.40
381. 1151 OG SER 117 A <--> 19740 CA GLU 891 B 3.29
382. 1151 OG SER 117 A <--> 19741 C GLU 891 B 3.01
383. 1151 OG SER 117 A <--> 19742 O GLU 891 B 2.47
384. 1154 N HIS 118 A <--> 19736 CA GLY 890 B 3.39
385. 1154 N HIS 118 A <--> 19737 C GLY 890 B 3.51
386. 1154 N HIS 118 A <--> 19739 N GLU 891 B 2.73
387. 1154 N HIS 118 A <--> 19740 CA GLU 891 B 3.68
388. 1155 CA HIS 118 A <--> 19737 C GLY 890 B 3.76
389. 1155 CA HIS 118 A <--> 19739 N GLU 891 B 2.98

390. 1155 CA HIS 118 A <--> 19740 CA GLU 891 B 3.63
391. 1156 C HIS 118 A <--> 19736 CA GLY 890 B 3.16
392. 1156 C HIS 118 A <--> 19737 C GLY 890 B 2.79
393. 1156 C HIS 118 A <--> 19738 O GLY 890 B 3.57
394. 1156 C HIS 118 A <--> 19739 N GLU 891 B 2.39
395. 1156 C HIS 118 A <--> 19740 CA GLU 891 B 3.11
396. 1156 C HIS 118 A <--> 19743 CB GLU 891 B 3.46
397. 1157 O HIS 118 A <--> 19735 N GLY 890 B 3.03
398. 1157 O HIS 118 A <--> 19736 CA GLY 890 B 2.05
399. 1157 O HIS 118 A <--> 19737 C GLY 890 B 2.03
400. 1157 O HIS 118 A <--> 19738 O GLY 890 B 2.84
401. 1157 O HIS 118 A <--> 19739 N GLU 891 B 2.22
402. 1157 O HIS 118 A <--> 19740 CA GLU 891 B 3.32
403. 1157 O HIS 118 A <--> 19743 CB GLU 891 B 3.70
404. 1162 CE1 HIS 118 A <--> 19310 ND1 HIS 833 B 3.66
405. 1162 CE1 HIS 118 A <--> 19312 CE1 HIS 833 B 2.89
406. 1162 CE1 HIS 118 A <--> 19313 NE2 HIS 833 B 3.13
407. 1163 NE2 HIS 118 A <--> 19312 CE1 HIS 833 B 3.26
408. 1163 NE2 HIS 118 A <--> 19313 NE2 HIS 833 B 3.84
409. 1166 N ARG 119 A <--> 19737 C GLY 890 B 3.48
410. 1166 N ARG 119 A <--> 19738 O GLY 890 B 3.88
411. 1166 N ARG 119 A <--> 19739 N GLU 891 B 3.07
412. 1166 N ARG 119 A <--> 19740 CA GLU 891 B 3.24
413. 1166 N ARG 119 A <--> 19743 CB GLU 891 B 3.05
414. 1167 CA ARG 119 A <--> 19307 O HIS 833 B 3.80
415. 1167 CA ARG 119 A <--> 19737 C GLY 890 B 3.61
416. 1167 CA ARG 119 A <--> 19738 O GLY 890 B 3.55
417. 1167 CA ARG 119 A <--> 19739 N GLU 891 B 3.65
418. 1167 CA ARG 119 A <--> 19740 CA GLU 891 B 3.71
419. 1167 CA ARG 119 A <--> 19743 CB GLU 891 B 2.94
420. 1167 CA ARG 119 A <--> 19746 OE1 GLU 891 B 3.29
421. 1168 C ARG 119 A <--> 19307 O HIS 833 B 3.42
422. 1168 C ARG 119 A <--> 19317 O GLY 834 B 3.77
423. 1169 O ARG 119 A <--> 19317 O GLY 834 B 3.25
424. 1169 O ARG 119 A <--> 19331 CG ASP 836 B 3.73
425. 1169 O ARG 119 A <--> 19332 OD1 ASP 836 B 3.09
426. 1169 O ARG 119 A <--> 19333 OD2 ASP 836 B 3.70
427. 1169 O ARG 119 A <--> 19738 O GLY 890 B 3.87
428. 1170 CB ARG 119 A <--> 19298 C LEU 832 B 3.80
429. 1170 CB ARG 119 A <--> 19299 O LEU 832 B 3.78
430. 1170 CB ARG 119 A <--> 19304 N HIS 833 B 3.00
431. 1170 CB ARG 119 A <--> 19305 CA HIS 833 B 3.84
432. 1170 CB ARG 119 A <--> 19306 C HIS 833 B 3.53
433. 1170 CB ARG 119 A <--> 19307 O HIS 833 B 3.03
434. 1170 CB ARG 119 A <--> 19743 CB GLU 891 B 3.44
435. 1170 CB ARG 119 A <--> 19745 CD GLU 891 B 3.56
436. 1170 CB ARG 119 A <--> 19746 OE1 GLU 891 B 2.72
437. 1171 CG ARG 119 A <--> 19304 N HIS 833 B 3.19
438. 1171 CG ARG 119 A <--> 19305 CA HIS 833 B 3.76
439. 1171 CG ARG 119 A <--> 19306 C HIS 833 B 3.37
440. 1171 CG ARG 119 A <--> 19307 O HIS 833 B 3.28
441. 1171 CG ARG 119 A <--> 19314 N GLY 834 B 3.89
442. 1171 CG ARG 119 A <--> 19317 O GLY 834 B 3.17
443. 1171 CG ARG 119 A <--> 19745 CD GLU 891 B 3.40
444. 1171 CG ARG 119 A <--> 19746 OE1 GLU 891 B 2.24
445. 1172 CD ARG 119 A <--> 19295 OG SER 831 B 3.51
446. 1172 CD ARG 119 A <--> 19297 CA LEU 832 B 3.81
447. 1172 CD ARG 119 A <--> 19298 C LEU 832 B 2.91
448. 1172 CD ARG 119 A <--> 19299 O LEU 832 B 3.31
449. 1172 CD ARG 119 A <--> 19304 N HIS 833 B 2.42

450.	1172	CD	ARG	119	A	<-->	19305	CA	HIS	833	B	2.91
451.	1172	CD	ARG	119	A	<-->	19306	C	HIS	833	B	3.03
452.	1172	CD	ARG	119	A	<-->	19307	O	HIS	833	B	3.47
453.	1172	CD	ARG	119	A	<-->	19314	N	GLY	834	B	3.49
454.	1172	CD	ARG	119	A	<-->	19317	O	GLY	834	B	3.66
455.	1172	CD	ARG	119	A	<-->	19745	CD	GLU	891	B	3.74
456.	1172	CD	ARG	119	A	<-->	19746	OE1	GLU	891	B	2.80
457.	1173	NE	ARG	119	A	<-->	19297	CA	LEU	832	B	3.61
458.	1173	NE	ARG	119	A	<-->	19298	C	LEU	832	B	3.03
459.	1173	NE	ARG	119	A	<-->	19299	O	LEU	832	B	3.89
460.	1173	NE	ARG	119	A	<-->	19304	N	HIS	833	B	2.19
461.	1173	NE	ARG	119	A	<-->	19305	CA	HIS	833	B	1.87
462.	1173	NE	ARG	119	A	<-->	19306	C	HIS	833	B	1.88
463.	1173	NE	ARG	119	A	<-->	19307	O	HIS	833	B	2.74
464.	1173	NE	ARG	119	A	<-->	19308	CB	HIS	833	B	3.38
465.	1173	NE	ARG	119	A	<-->	19314	N	GLY	834	B	2.14
466.	1173	NE	ARG	119	A	<-->	19315	CA	GLY	834	B	3.34
467.	1173	NE	ARG	119	A	<-->	19316	C	GLY	834	B	3.60
468.	1173	NE	ARG	119	A	<-->	19317	O	GLY	834	B	3.30
469.	1174	CZ	ARG	119	A	<-->	19304	N	HIS	833	B	3.45
470.	1174	CZ	ARG	119	A	<-->	19305	CA	HIS	833	B	2.70
471.	1174	CZ	ARG	119	A	<-->	19306	C	HIS	833	B	2.29
472.	1174	CZ	ARG	119	A	<-->	19307	O	HIS	833	B	3.27
473.	1174	CZ	ARG	119	A	<-->	19314	N	GLY	834	B	1.65
474.	1174	CZ	ARG	119	A	<-->	19315	CA	GLY	834	B	2.62
475.	1174	CZ	ARG	119	A	<-->	19316	C	GLY	834	B	2.75
476.	1174	CZ	ARG	119	A	<-->	19317	O	GLY	834	B	2.77
477.	1174	CZ	ARG	119	A	<-->	19318	N	ASN	835	B	3.71
478.	1175	NH1	ARG	119	A	<-->	19277	O	LEU	829	B	3.59
479.	1175	NH1	ARG	119	A	<-->	19306	C	HIS	833	B	3.48
480.	1175	NH1	ARG	119	A	<-->	19314	N	GLY	834	B	2.71
481.	1175	NH1	ARG	119	A	<-->	19315	CA	GLY	834	B	3.15
482.	1175	NH1	ARG	119	A	<-->	19316	C	GLY	834	B	2.62
483.	1175	NH1	ARG	119	A	<-->	19317	O	GLY	834	B	2.46
484.	1175	NH1	ARG	119	A	<-->	19318	N	ASN	835	B	3.28
485.	1175	NH1	ARG	119	A	<-->	19319	CA	ASN	835	B	3.83
486.	1176	NH2	ARG	119	A	<-->	19305	CA	HIS	833	B	2.82
487.	1176	NH2	ARG	119	A	<-->	19306	C	HIS	833	B	2.33
488.	1176	NH2	ARG	119	A	<-->	19307	O	HIS	833	B	3.42
489.	1176	NH2	ARG	119	A	<-->	19308	CB	HIS	833	B	3.64
490.	1176	NH2	ARG	119	A	<-->	19314	N	GLY	834	B	1.23
491.	1176	NH2	ARG	119	A	<-->	19315	CA	GLY	834	B	2.16
492.	1176	NH2	ARG	119	A	<-->	19316	C	GLY	834	B	2.84
493.	1176	NH2	ARG	119	A	<-->	19317	O	GLY	834	B	3.37
494.	1176	NH2	ARG	119	A	<-->	19318	N	ASN	835	B	3.60
495.	1183	N	MET	120	A	<-->	19307	O	HIS	833	B	3.13
496.	1184	CA	MET	120	A	<-->	19307	O	HIS	833	B	3.80
497.	1186	O	MET	120	A	<-->	19736	CA	GLY	890	B	3.79
498.	1192	N	LEU	121	A	<-->	19332	OD1	ASP	836	B	3.52
499.	1193	CA	LEU	121	A	<-->	19332	OD1	ASP	836	B	3.88
500.	1197	CG	LEU	121	A	<-->	19331	CG	ASP	836	B	3.62
501.	1197	CG	LEU	121	A	<-->	19332	OD1	ASP	836	B	3.18
502.	1199	CD2	LEU	121	A	<-->	19327	CA	ASP	836	B	2.99
503.	1199	CD2	LEU	121	A	<-->	19330	CB	ASP	836	B	2.71
504.	1199	CD2	LEU	121	A	<-->	19331	CG	ASP	836	B	2.35
505.	1199	CD2	LEU	121	A	<-->	19332	OD1	ASP	836	B	2.38
506.	1199	CD2	LEU	121	A	<-->	19333	OD2	ASP	836	B	2.99
507.	1256	CE2	PHE	128	A	<-->	16953	OH	TYR	534	B	3.41
508.	1257	CZ	PHE	128	A	<-->	16953	OH	TYR	534	B	2.83
509.	1269	CG	PHE	129	A	<-->	16980	CG	LEU	538	B	3.88

510. 1269 CG PHE 129 A <--> 16981 CD1 LEU 538 B 3.79
511. 1269 CG PHE 129 A <--> 16982 CD2 LEU 538 B 2.84
512. 1270 CD1 PHE 129 A <--> 16982 CD2 LEU 538 B 3.45
513. 1271 CD2 PHE 129 A <--> 16980 CG LEU 538 B 3.49
514. 1271 CD2 PHE 129 A <--> 16982 CD2 LEU 538 B 2.10
515. 1272 CE1 PHE 129 A <--> 16982 CD2 LEU 538 B 3.45
516. 1273 CE2 PHE 129 A <--> 16980 CG LEU 538 B 3.59
517. 1273 CE2 PHE 129 A <--> 16982 CD2 LEU 538 B 2.10
518. 1274 CZ PHE 129 A <--> 16982 CD2 LEU 538 B 2.85
519. 1284 O LEU 130 A <--> 16953 OH TYR 534 B 3.75
520. 1288 CD2 LEU 130 A <--> 16697 CE2 PHE 501 B 3.85
521. 1288 CD2 LEU 130 A <--> 16698 CZ PHE 501 B 3.76
522. 1307 N ILE 132 A <--> 16953 OH TYR 534 B 3.89
523. 1311 CB ILE 132 A <--> 16951 CE2 TYR 534 B 3.86
524. 1311 CB ILE 132 A <--> 16953 OH TYR 534 B 3.54
525. 1312 CG1 ILE 132 A <--> 16950 CE1 TYR 534 B 3.75
526. 1312 CG1 ILE 132 A <--> 16951 CE2 TYR 534 B 3.32
527. 1312 CG1 ILE 132 A <--> 16952 CZ TYR 534 B 2.95
528. 1312 CG1 ILE 132 A <--> 16953 OH TYR 534 B 2.59
529. 1313 CG2 ILE 132 A <--> 16951 CE2 TYR 534 B 3.26
530. 1313 CG2 ILE 132 A <--> 16952 CZ TYR 534 B 3.86
531. 1313 CG2 ILE 132 A <--> 16953 OH TYR 534 B 3.77
532. 2043 CG ASP 207 A <--> 19753 CG MET 892 B 3.36
533. 2044 OD1 ASP 207 A <--> 19753 CG MET 892 B 3.21
534. 2045 OD2 ASP 207 A <--> 19753 CG MET 892 B 3.06
535. 2045 OD2 ASP 207 A <--> 19754 SD MET 892 B 3.84
536. 2509 OE1 GLU 255 A <--> 19301 CG LEU 832 B 3.89
537. 2509 OE1 GLU 255 A <--> 19302 CD1 LEU 832 B 3.62
538. 2509 OE1 GLU 255 A <--> 19303 CD2 LEU 832 B 3.28
539. 2521 N THR 257 A <--> 19302 CD1 LEU 832 B 3.87
540. 2522 CA THR 257 A <--> 19302 CD1 LEU 832 B 3.24
541. 2525 CB THR 257 A <--> 19296 N LEU 832 B 3.74
542. 2525 CB THR 257 A <--> 19300 CB LEU 832 B 3.59
543. 2525 CB THR 257 A <--> 19301 CG LEU 832 B 3.01
544. 2525 CB THR 257 A <--> 19302 CD1 LEU 832 B 1.80
545. 2525 CB THR 257 A <--> 19303 CD2 LEU 832 B 3.52
546. 2526 OG1 THR 257 A <--> 19296 N LEU 832 B 3.01
547. 2526 OG1 THR 257 A <--> 19297 CA LEU 832 B 3.36
548. 2526 OG1 THR 257 A <--> 19299 O LEU 832 B 3.81
549. 2526 OG1 THR 257 A <--> 19300 CB LEU 832 B 2.72
550. 2526 OG1 THR 257 A <--> 19301 CG LEU 832 B 2.79
551. 2526 OG1 THR 257 A <--> 19302 CD1 LEU 832 B 2.13
552. 2526 OG1 THR 257 A <--> 19303 CD2 LEU 832 B 3.48
553. 2527 CG2 THR 257 A <--> 19300 CB LEU 832 B 3.87
554. 2527 CG2 THR 257 A <--> 19301 CG LEU 832 B 2.95
555. 2527 CG2 THR 257 A <--> 19302 CD1 LEU 832 B 2.25
556. 2527 CG2 THR 257 A <--> 19303 CD2 LEU 832 B 2.75
557. 2540 C LEU 259 A <--> 16267 CB THR 447 B 3.61
558. 2540 C LEU 259 A <--> 16269 CG2 THR 447 B 3.42
559. 2541 O LEU 259 A <--> 16267 CB THR 447 B 3.07
560. 2541 O LEU 259 A <--> 16268 OG1 THR 447 B 3.27
561. 2541 O LEU 259 A <--> 16269 CG2 THR 447 B 3.23
562. 2542 CB LEU 259 A <--> 16267 CB THR 447 B 3.50
563. 2542 CB LEU 259 A <--> 16269 CG2 THR 447 B 3.86
564. 2543 CG LEU 259 A <--> 16264 CA THR 447 B 3.22
565. 2543 CG LEU 259 A <--> 16267 CB THR 447 B 2.82
566. 2543 CG LEU 259 A <--> 16269 CG2 THR 447 B 2.84
567. 2544 CD1 LEU 259 A <--> 16269 CG2 THR 447 B 3.74
568. 2545 CD2 LEU 259 A <--> 16256 O HIS 446 B 3.75
569. 2545 CD2 LEU 259 A <--> 16263 N THR 447 B 3.47

570.	2545	CD2	LEU	259	A	<-->	16264	CA	THR	447	B	2.25
571.	2545	CD2	LEU	259	A	<-->	16265	C	THR	447	B	2.77
572.	2545	CD2	LEU	259	A	<-->	16266	O	THR	447	B	2.70
573.	2545	CD2	LEU	259	A	<-->	16267	CB	THR	447	B	2.59
574.	2545	CD2	LEU	259	A	<-->	16269	CG2	THR	447	B	3.08
575.	2547	N	GLU	260	A	<-->	16269	CG2	THR	447	B	3.68
576.	2548	CA	GLU	260	A	<-->	16269	CG2	THR	447	B	3.82
577.	2553	CD	GLU	260	A	<-->	16268	OG1	THR	447	B	3.75
578.	2555	OE2	GLU	260	A	<-->	16268	OG1	THR	447	B	2.95
579.	2564	NE	ARG	261	A	<-->	16476	NZ	LYS	474	B	3.39
580.	2564	NE	ARG	261	A	<-->	16484	CE	LYS	475	B	3.69
581.	2564	NE	ARG	261	A	<-->	16485	NZ	LYS	475	B	3.04
582.	2565	CZ	ARG	261	A	<-->	16484	CE	LYS	475	B	2.96
583.	2565	CZ	ARG	261	A	<-->	16485	NZ	LYS	475	B	1.92
584.	2566	NH1	ARG	261	A	<-->	16484	CE	LYS	475	B	3.89
585.	2566	NH1	ARG	261	A	<-->	16485	NZ	LYS	475	B	2.53
586.	2567	NH2	ARG	261	A	<-->	16483	CD	LYS	475	B	2.92
587.	2567	NH2	ARG	261	A	<-->	16484	CE	LYS	475	B	1.66
588.	2567	NH2	ARG	261	A	<-->	16485	NZ	LYS	475	B	0.78
589.	2726	OD2	ASP	277	A	<-->	16475	CE	LYS	474	B	3.28
590.	2726	OD2	ASP	277	A	<-->	16476	NZ	LYS	474	B	3.69

Salt bridges

<----- A T O M 1 -----> <----- A T O M 2 ----->

	Atom no.	Atom name	Res name	Res no.	Chain		Atom no.	Atom name	Res name	Res no.	Chain	Distance
1.	1088	OD1	ASP	111	A	<-->	16506	NH1	ARG	477	B	1.22
2.	2726	OD2	ASP	277	A	<-->	16476	NZ	LYS	474	B	3.69

Number of salt bridges: 2

Number of hydrogen bonds: 12

Number of non-bonded contacts: 590

[D] ROBO1 (WT) + P54R_FULL PROTEIN

Hydrogen bonds

<----- A T O M 1 -----> <----- A T O M 2 ----->

	Atom no.	Atom name	Res name	Res no.	Chain		Atom no.	Atom name	Res name	Res no.	Chain	Distance
1.	744	N	THR	96	A	<-->	4981	OG1	THR	489	B	2.93
2.	749	OG1	THR	96	A	<-->	4981	OG1	THR	489	B	2.91
3.	749	OG1	THR	96	A	<-->	4981	OG1	THR	489	B	2.91
4.	768	O	ILE	99	A	<-->	3694	NZ	LYS	365	B	2.11
5.	807	OH	TYR	102	A	<-->	6009	ND2	ASN	592	B	3.18
6.	859	OE1	GLU	109	A	<-->	2348	NH2	ARG	227	B	1.64
7.	899	OD1	ASP	114	A	<-->	2318	NH1	ARG	225	B	3.04
8.	899	OD1	ASP	114	A	<-->	2319	NH2	ARG	225	B	2.14
9.	904	O	PRO	115	A	<-->	2316	NE	ARG	225	B	2.41
10.	944	NH1	ARG	119	A	<-->	6495	OD2	ASP	639	B	2.84

Non-bonded contacts

<----- A T O M 1 -----> <----- A T O M 2 ----->

	Atom no.	Atom name	Res name	Res no.	Chain		Atom no.	Atom name	Res name	Res no.	Chain	Distance
1.	457	O	SER	59	A	<-->	6650	CD2	LEU	656	B	3.76
2.	469	NH1	ARG	60	A	<-->	6610	OG1	THR	652	B	3.48
3.	471	N	LEU	61	A	<-->	6650	CD2	LEU	656	B	3.56
4.	738	CA	PRO	95	A	<-->	4981	OG1	THR	489	B	3.77
5.	739	C	PRO	95	A	<-->	4981	OG1	THR	489	B	3.63
6.	741	CB	PRO	95	A	<-->	4981	OG1	THR	489	B	3.03
7.	741	CB	PRO	95	A	<-->	4982	CG2	THR	489	B	3.58
8.	742	CG	PRO	95	A	<-->	4227	OG	SER	417	B	3.75
9.	744	N	THR	96	A	<-->	4182	CB	LYS	413	B	3.67
10.	744	N	THR	96	A	<-->	4185	CE	LYS	413	B	3.83
11.	744	N	THR	96	A	<-->	4981	OG1	THR	489	B	2.93
12.	745	CA	THR	96	A	<-->	4182	CB	LYS	413	B	3.06
13.	745	CA	THR	96	A	<-->	4185	CE	LYS	413	B	3.06
14.	745	CA	THR	96	A	<-->	4186	NZ	LYS	413	B	3.50
15.	745	CA	THR	96	A	<-->	4981	OG1	THR	489	B	3.71
16.	746	C	THR	96	A	<-->	4179	CA	LYS	413	B	3.88
17.	746	C	THR	96	A	<-->	4180	C	LYS	413	B	3.29
18.	746	C	THR	96	A	<-->	4181	O	LYS	413	B	3.63
19.	746	C	THR	96	A	<-->	4182	CB	LYS	413	B	3.23
20.	746	C	THR	96	A	<-->	4191	N	GLY	414	B	3.27
21.	746	C	THR	96	A	<-->	4192	CA	GLY	414	B	3.40
22.	747	O	THR	96	A	<-->	4179	CA	LYS	413	B	3.29
23.	747	O	THR	96	A	<-->	4180	C	LYS	413	B	2.41
24.	747	O	THR	96	A	<-->	4181	O	LYS	413	B	2.91
25.	747	O	THR	96	A	<-->	4182	CB	LYS	413	B	3.08
26.	747	O	THR	96	A	<-->	4191	N	GLY	414	B	2.11
27.	747	O	THR	96	A	<-->	4192	CA	GLY	414	B	2.20
28.	747	O	THR	96	A	<-->	4193	C	GLY	414	B	3.67
29.	747	O	THR	96	A	<-->	4981	OG1	THR	489	B	3.44
30.	748	CB	THR	96	A	<-->	4179	CA	LYS	413	B	3.42
31.	748	CB	THR	96	A	<-->	4180	C	LYS	413	B	3.86

32.	748	CB	THR	96	A	<-->	4182	CB	LYS	413	B	2.00
33.	748	CB	THR	96	A	<-->	4183	CG	LYS	413	B	2.59
34.	748	CB	THR	96	A	<-->	4184	CD	LYS	413	B	2.53
35.	748	CB	THR	96	A	<-->	4185	CE	LYS	413	B	1.80
36.	748	CB	THR	96	A	<-->	4186	NZ	LYS	413	B	2.58
37.	748	CB	THR	96	A	<-->	4981	OG1	THR	489	B	3.76
38.	749	OG1	THR	96	A	<-->	4178	N	LYS	413	B	3.13
39.	749	OG1	THR	96	A	<-->	4179	CA	LYS	413	B	2.17
40.	749	OG1	THR	96	A	<-->	4180	C	LYS	413	B	2.88
41.	749	OG1	THR	96	A	<-->	4181	O	LYS	413	B	3.76
42.	749	OG1	THR	96	A	<-->	4182	CB	LYS	413	B	0.78
43.	749	OG1	THR	96	A	<-->	4183	CG	LYS	413	B	1.84
44.	749	OG1	THR	96	A	<-->	4184	CD	LYS	413	B	2.62
45.	749	OG1	THR	96	A	<-->	4185	CE	LYS	413	B	2.64
46.	749	OG1	THR	96	A	<-->	4186	NZ	LYS	413	B	3.70
47.	749	OG1	THR	96	A	<-->	4191	N	GLY	414	B	3.22
48.	749	OG1	THR	96	A	<-->	4973	C	GLY	488	B	3.85
49.	749	OG1	THR	96	A	<-->	4974	O	GLY	488	B	3.56
50.	749	OG1	THR	96	A	<-->	4980	CB	THR	489	B	3.61
51.	749	OG1	THR	96	A	<-->	4981	OG1	THR	489	B	2.91
52.	750	CG2	THR	96	A	<-->	4182	CB	LYS	413	B	3.00
53.	750	CG2	THR	96	A	<-->	4183	CG	LYS	413	B	2.82
54.	750	CG2	THR	96	A	<-->	4184	CD	LYS	413	B	2.38
55.	750	CG2	THR	96	A	<-->	4185	CE	LYS	413	B	1.13
56.	750	CG2	THR	96	A	<-->	4186	NZ	LYS	413	B	2.29
57.	752	CA	PRO	97	A	<-->	4181	O	LYS	413	B	3.81
58.	752	CA	PRO	97	A	<-->	4192	CA	GLY	414	B	3.86
59.	753	C	PRO	97	A	<-->	4180	C	LYS	413	B	3.65
60.	753	C	PRO	97	A	<-->	4181	O	LYS	413	B	2.90
61.	754	O	PRO	97	A	<-->	4180	C	LYS	413	B	3.67
62.	754	O	PRO	97	A	<-->	4181	O	LYS	413	B	2.91
63.	758	N	THR	98	A	<-->	4180	C	LYS	413	B	3.85
64.	758	N	THR	98	A	<-->	4181	O	LYS	413	B	2.89
65.	758	N	THR	98	A	<-->	4192	CA	GLY	414	B	3.80
66.	759	CA	THR	98	A	<-->	4181	O	LYS	413	B	3.03
67.	762	CB	THR	98	A	<-->	4181	O	LYS	413	B	3.30
68.	762	CB	THR	98	A	<-->	4202	CG2	THR	415	B	3.05
69.	763	OG1	THR	98	A	<-->	3692	CD	LYS	365	B	3.76
70.	763	OG1	THR	98	A	<-->	4202	CG2	THR	415	B	2.88
71.	764	CG2	THR	98	A	<-->	4202	CG2	THR	415	B	3.82
72.	767	C	ILE	99	A	<-->	3694	NZ	LYS	365	B	3.19
73.	768	O	ILE	99	A	<-->	3692	CD	LYS	365	B	3.68
74.	768	O	ILE	99	A	<-->	3693	CE	LYS	365	B	3.28
75.	768	O	ILE	99	A	<-->	3694	NZ	LYS	365	B	2.11
76.	773	N	GLU	100	A	<-->	3694	NZ	LYS	365	B	3.73
77.	774	CA	GLU	100	A	<-->	3694	NZ	LYS	365	B	3.38
78.	777	CB	GLU	100	A	<-->	3692	CD	LYS	365	B	3.83
79.	777	CB	GLU	100	A	<-->	3694	NZ	LYS	365	B	3.62
80.	780	OE1	GLU	100	A	<-->	3690	CB	LYS	365	B	3.83
81.	780	OE1	GLU	100	A	<-->	3704	OG	SER	366	B	3.54
82.	781	OE2	GLU	100	A	<-->	5991	CG2	THR	590	B	3.22
83.	807	OH	TYR	102	A	<-->	6009	ND2	ASN	592	B	3.18
84.	819	C	GLY	104	A	<-->	842	CB	MET	87	B	3.32
85.	819	C	GLY	104	A	<-->	843	CG	MET	87	B	3.61
86.	820	O	GLY	104	A	<-->	839	CA	MET	87	B	3.77
87.	820	O	GLY	104	A	<-->	842	CB	MET	87	B	2.47
88.	820	O	GLY	104	A	<-->	843	CG	MET	87	B	2.69
89.	823	C	GLY	105	A	<-->	842	CB	MET	87	B	3.87
90.	823	C	GLY	105	A	<-->	6037	CD2	LEU	594	B	3.85
91.	824	O	GLY	105	A	<-->	6035	CG	LEU	594	B	3.70

92.	824	O	GLY	105	A	<-->	6037	CD2	LEU	594	B	2.78
93.	825	N	GLU	106	A	<-->	839	CA	MET	87	B	3.88
94.	825	N	GLU	106	A	<-->	842	CB	MET	87	B	3.31
95.	826	CA	GLU	106	A	<-->	824	OE1	GLN	85	B	3.77
96.	826	CA	GLU	106	A	<-->	839	CA	MET	87	B	3.80
97.	826	CA	GLU	106	A	<-->	842	CB	MET	87	B	3.72
98.	827	C	GLU	106	A	<-->	824	OE1	GLN	85	B	3.12
99.	828	O	GLU	106	A	<-->	823	CD	GLN	85	B	3.83
100.	828	O	GLU	106	A	<-->	824	OE1	GLN	85	B	2.67
101.	829	CB	GLU	106	A	<-->	824	OE1	GLN	85	B	3.29
102.	829	CB	GLU	106	A	<-->	831	C	LEU	86	B	3.38
103.	829	CB	GLU	106	A	<-->	832	O	LEU	86	B	3.71
104.	829	CB	GLU	106	A	<-->	838	N	MET	87	B	2.97
105.	829	CB	GLU	106	A	<-->	839	CA	MET	87	B	2.87
106.	829	CB	GLU	106	A	<-->	842	CB	MET	87	B	3.08
107.	830	CG	GLU	106	A	<-->	830	CA	LEU	86	B	3.71
108.	830	CG	GLU	106	A	<-->	831	C	LEU	86	B	2.43
109.	830	CG	GLU	106	A	<-->	832	O	LEU	86	B	2.64
110.	830	CG	GLU	106	A	<-->	838	N	MET	87	B	1.95
111.	830	CG	GLU	106	A	<-->	839	CA	MET	87	B	1.53
112.	830	CG	GLU	106	A	<-->	840	C	MET	87	B	2.85
113.	830	CG	GLU	106	A	<-->	841	O	MET	87	B	3.35
114.	830	CG	GLU	106	A	<-->	842	CB	MET	87	B	2.37
115.	830	CG	GLU	106	A	<-->	843	CG	MET	87	B	3.81
116.	830	CG	GLU	106	A	<-->	847	N	GLU	88	B	3.88
117.	830	CG	GLU	106	A	<-->	6035	CG	LEU	594	B	3.57
118.	831	CD	GLU	106	A	<-->	819	C	GLN	85	B	3.83
119.	831	CD	GLU	106	A	<-->	829	N	LEU	86	B	3.06
120.	831	CD	GLU	106	A	<-->	830	CA	LEU	86	B	2.24
121.	831	CD	GLU	106	A	<-->	831	C	LEU	86	B	1.04
122.	831	CD	GLU	106	A	<-->	832	O	LEU	86	B	1.83
123.	831	CD	GLU	106	A	<-->	833	CB	LEU	86	B	3.55
124.	831	CD	GLU	106	A	<-->	836	CD2	LEU	86	B	3.89
125.	831	CD	GLU	106	A	<-->	838	N	MET	87	B	0.79
126.	831	CD	GLU	106	A	<-->	839	CA	MET	87	B	1.71
127.	831	CD	GLU	106	A	<-->	840	C	MET	87	B	2.85
128.	831	CD	GLU	106	A	<-->	841	O	MET	87	B	3.73
129.	831	CD	GLU	106	A	<-->	842	CB	MET	87	B	2.85
130.	831	CD	GLU	106	A	<-->	843	CG	MET	87	B	3.88
131.	831	CD	GLU	106	A	<-->	847	N	GLU	88	B	3.40
132.	832	OE1	GLU	106	A	<-->	818	CA	GLN	85	B	3.81
133.	832	OE1	GLU	106	A	<-->	819	C	GLN	85	B	2.74
134.	832	OE1	GLU	106	A	<-->	820	O	GLN	85	B	3.00
135.	832	OE1	GLU	106	A	<-->	821	CB	GLN	85	B	3.73
136.	832	OE1	GLU	106	A	<-->	822	CG	GLN	85	B	3.90
137.	832	OE1	GLU	106	A	<-->	829	N	LEU	86	B	2.29
138.	832	OE1	GLU	106	A	<-->	830	CA	LEU	86	B	1.82
139.	832	OE1	GLU	106	A	<-->	831	C	LEU	86	B	1.61
140.	832	OE1	GLU	106	A	<-->	832	O	LEU	86	B	2.67
141.	832	OE1	GLU	106	A	<-->	833	CB	LEU	86	B	3.29
142.	832	OE1	GLU	106	A	<-->	836	CD2	LEU	86	B	3.84
143.	832	OE1	GLU	106	A	<-->	838	N	MET	87	B	1.48
144.	832	OE1	GLU	106	A	<-->	839	CA	MET	87	B	2.71
145.	832	OE1	GLU	106	A	<-->	842	CB	MET	87	B	3.40
146.	833	OE2	GLU	106	A	<-->	829	N	LEU	86	B	3.39
147.	833	OE2	GLU	106	A	<-->	830	CA	LEU	86	B	2.22
148.	833	OE2	GLU	106	A	<-->	831	C	LEU	86	B	0.87
149.	833	OE2	GLU	106	A	<-->	832	O	LEU	86	B	1.32
150.	833	OE2	GLU	106	A	<-->	833	CB	LEU	86	B	3.05
151.	833	OE2	GLU	106	A	<-->	834	CG	LEU	86	B	3.24

152.	833	OE2	GLU	106	A	<-->	835	CD1	LEU	86	B	3.02
153.	833	OE2	GLU	106	A	<-->	836	CD2	LEU	86	B	3.29
154.	833	OE2	GLU	106	A	<-->	838	N	MET	87	B	1.25
155.	833	OE2	GLU	106	A	<-->	839	CA	MET	87	B	1.98
156.	833	OE2	GLU	106	A	<-->	840	C	MET	87	B	2.37
157.	833	OE2	GLU	106	A	<-->	841	O	MET	87	B	3.28
158.	833	OE2	GLU	106	A	<-->	842	CB	MET	87	B	3.40
159.	833	OE2	GLU	106	A	<-->	847	N	GLU	88	B	2.64
160.	833	OE2	GLU	106	A	<-->	857	N	ASN	89	B	3.89
161.	834	N	ARG	107	A	<-->	824	OE1	GLN	85	B	3.77
162.	836	C	ARG	107	A	<-->	823	CD	GLN	85	B	3.81
163.	836	C	ARG	107	A	<-->	824	OE1	GLN	85	B	3.09
164.	836	C	ARG	107	A	<-->	825	NE2	GLN	85	B	3.85
165.	836	C	ARG	107	A	<-->	3456	CG	PRO	341	B	3.78
166.	837	O	ARG	107	A	<-->	823	CD	GLN	85	B	3.41
167.	837	O	ARG	107	A	<-->	824	OE1	GLN	85	B	2.90
168.	837	O	ARG	107	A	<-->	825	NE2	GLN	85	B	3.53
169.	837	O	ARG	107	A	<-->	3455	CB	PRO	341	B	3.36
170.	837	O	ARG	107	A	<-->	3456	CG	PRO	341	B	2.56
171.	838	CB	ARG	107	A	<-->	3704	OG	SER	366	B	3.49
172.	839	CG	ARG	107	A	<-->	3704	OG	SER	366	B	3.71
173.	839	CG	ARG	107	A	<-->	5374	CD	GLU	529	B	3.64
174.	839	CG	ARG	107	A	<-->	5375	OE1	GLU	529	B	3.07
175.	839	CG	ARG	107	A	<-->	5376	OE2	GLU	529	B	3.54
176.	840	CD	ARG	107	A	<-->	3704	OG	SER	366	B	2.84
177.	840	CD	ARG	107	A	<-->	5374	CD	GLU	529	B	2.41
178.	840	CD	ARG	107	A	<-->	5375	OE1	GLU	529	B	1.93
179.	840	CD	ARG	107	A	<-->	5376	OE2	GLU	529	B	2.36
180.	841	NE	ARG	107	A	<-->	3692	CD	LYS	365	B	3.39
181.	841	NE	ARG	107	A	<-->	3693	CE	LYS	365	B	3.56
182.	841	NE	ARG	107	A	<-->	3694	NZ	LYS	365	B	3.73
183.	841	NE	ARG	107	A	<-->	5373	CG	GLU	529	B	3.45
184.	841	NE	ARG	107	A	<-->	5374	CD	GLU	529	B	2.03
185.	841	NE	ARG	107	A	<-->	5375	OE1	GLU	529	B	2.39
186.	841	NE	ARG	107	A	<-->	5376	OE2	GLU	529	B	1.27
187.	842	CZ	ARG	107	A	<-->	3692	CD	LYS	365	B	3.84
188.	842	CZ	ARG	107	A	<-->	3693	CE	LYS	365	B	3.45
189.	842	CZ	ARG	107	A	<-->	3694	NZ	LYS	365	B	3.55
190.	842	CZ	ARG	107	A	<-->	5373	CG	GLU	529	B	2.78
191.	842	CZ	ARG	107	A	<-->	5374	CD	GLU	529	B	1.77
192.	842	CZ	ARG	107	A	<-->	5375	OE1	GLU	529	B	2.50
193.	842	CZ	ARG	107	A	<-->	5376	OE2	GLU	529	B	1.21
194.	843	NH1	ARG	107	A	<-->	5373	CG	GLU	529	B	2.57
195.	843	NH1	ARG	107	A	<-->	5374	CD	GLU	529	B	1.89
196.	843	NH1	ARG	107	A	<-->	5375	OE1	GLU	529	B	2.20
197.	843	NH1	ARG	107	A	<-->	5376	OE2	GLU	529	B	2.20
198.	844	NH2	ARG	107	A	<-->	3692	CD	LYS	365	B	3.44
199.	844	NH2	ARG	107	A	<-->	3693	CE	LYS	365	B	2.55
200.	844	NH2	ARG	107	A	<-->	3694	NZ	LYS	365	B	2.53
201.	844	NH2	ARG	107	A	<-->	5373	CG	GLU	529	B	3.19
202.	844	NH2	ARG	107	A	<-->	5374	CD	GLU	529	B	2.71
203.	844	NH2	ARG	107	A	<-->	5375	OE1	GLU	529	B	3.71
204.	844	NH2	ARG	107	A	<-->	5376	OE2	GLU	529	B	1.88
205.	845	N	VAL	108	A	<-->	823	CD	GLN	85	B	3.75
206.	845	N	VAL	108	A	<-->	824	OE1	GLN	85	B	3.20
207.	845	N	VAL	108	A	<-->	825	NE2	GLN	85	B	3.45
208.	846	CA	VAL	108	A	<-->	823	CD	GLN	85	B	3.08
209.	846	CA	VAL	108	A	<-->	824	OE1	GLN	85	B	3.02
210.	846	CA	VAL	108	A	<-->	825	NE2	GLN	85	B	2.35
211.	847	C	VAL	108	A	<-->	825	NE2	GLN	85	B	3.36

212.	849	CB	VAL	108	A	<-->	823	CD	GLN	85	B	2.78
213.	849	CB	VAL	108	A	<-->	824	OE1	GLN	85	B	2.88
214.	849	CB	VAL	108	A	<-->	825	NE2	GLN	85	B	2.04
215.	850	CG1	VAL	108	A	<-->	825	NE2	GLN	85	B	3.56
216.	851	CG2	VAL	108	A	<-->	823	CD	GLN	85	B	2.90
217.	851	CG2	VAL	108	A	<-->	824	OE1	GLN	85	B	3.56
218.	851	CG2	VAL	108	A	<-->	825	NE2	GLN	85	B	1.76
219.	852	N	GLU	109	A	<-->	825	NE2	GLN	85	B	3.43
220.	854	C	GLU	109	A	<-->	2348	NH2	ARG	227	B	3.82
221.	856	CB	GLU	109	A	<-->	5387	NE2	HIS	530	B	3.82
222.	857	CG	GLU	109	A	<-->	3442	CD1	LEU	339	B	2.83
223.	858	CD	GLU	109	A	<-->	2346	CZ	ARG	227	B	3.85
224.	858	CD	GLU	109	A	<-->	2348	NH2	ARG	227	B	2.81
225.	858	CD	GLU	109	A	<-->	3442	CD1	LEU	339	B	3.04
226.	859	OE1	GLU	109	A	<-->	2345	NE	ARG	227	B	3.52
227.	859	OE1	GLU	109	A	<-->	2346	CZ	ARG	227	B	2.84
228.	859	OE1	GLU	109	A	<-->	2347	NH1	ARG	227	B	3.77
229.	859	OE1	GLU	109	A	<-->	2348	NH2	ARG	227	B	1.64
230.	859	OE1	GLU	109	A	<-->	5387	NE2	HIS	530	B	3.83
231.	860	OE2	GLU	109	A	<-->	804	CB	VAL	83	B	3.69
232.	860	OE2	GLU	109	A	<-->	805	CG1	VAL	83	B	3.75
233.	860	OE2	GLU	109	A	<-->	806	CG2	VAL	83	B	3.05
234.	860	OE2	GLU	109	A	<-->	2348	NH2	ARG	227	B	3.45
235.	860	OE2	GLU	109	A	<-->	3441	CG	LEU	339	B	3.65
236.	860	OE2	GLU	109	A	<-->	3442	CD1	LEU	339	B	2.87
237.	861	N	THR	110	A	<-->	2348	NH2	ARG	227	B	3.60
238.	862	CA	THR	110	A	<-->	2348	NH2	ARG	227	B	3.66
239.	863	C	THR	110	A	<-->	2347	NH1	ARG	227	B	3.85
240.	863	C	THR	110	A	<-->	2348	NH2	ARG	227	B	3.46
241.	864	O	THR	110	A	<-->	2348	NH2	ARG	227	B	3.71
242.	868	N	ASP	111	A	<-->	2347	NH1	ARG	227	B	3.34
243.	868	N	ASP	111	A	<-->	2348	NH2	ARG	227	B	3.77
244.	869	CA	ASP	111	A	<-->	2347	NH1	ARG	227	B	3.33
245.	872	CB	ASP	111	A	<-->	2347	NH1	ARG	227	B	3.58
246.	894	CA	ASP	114	A	<-->	2319	NH2	ARG	225	B	3.23
247.	895	C	ASP	114	A	<-->	2317	CZ	ARG	225	B	3.63
248.	895	C	ASP	114	A	<-->	2319	NH2	ARG	225	B	2.62
249.	896	O	ASP	114	A	<-->	2319	NH2	ARG	225	B	3.48
250.	897	CB	ASP	114	A	<-->	2319	NH2	ARG	225	B	3.74
251.	898	CG	ASP	114	A	<-->	2319	NH2	ARG	225	B	3.11
252.	899	OD1	ASP	114	A	<-->	2317	CZ	ARG	225	B	2.97
253.	899	OD1	ASP	114	A	<-->	2318	NH1	ARG	225	B	3.04
254.	899	OD1	ASP	114	A	<-->	2319	NH2	ARG	225	B	2.14
255.	901	N	PRO	115	A	<-->	2316	NE	ARG	225	B	3.32
256.	901	N	PRO	115	A	<-->	2317	CZ	ARG	225	B	2.63
257.	901	N	PRO	115	A	<-->	2318	NH1	ARG	225	B	3.39
258.	901	N	PRO	115	A	<-->	2319	NH2	ARG	225	B	1.90
259.	902	CA	PRO	115	A	<-->	2316	NE	ARG	225	B	3.14
260.	902	CA	PRO	115	A	<-->	2317	CZ	ARG	225	B	3.02
261.	902	CA	PRO	115	A	<-->	2319	NH2	ARG	225	B	2.43
262.	903	C	PRO	115	A	<-->	2316	NE	ARG	225	B	2.99
263.	903	C	PRO	115	A	<-->	2317	CZ	ARG	225	B	3.03
264.	903	C	PRO	115	A	<-->	2319	NH2	ARG	225	B	2.31
265.	904	O	PRO	115	A	<-->	2306	CD1	LEU	224	B	3.70
266.	904	O	PRO	115	A	<-->	2307	CD2	LEU	224	B	3.87
267.	904	O	PRO	115	A	<-->	2315	CD	ARG	225	B	3.82
268.	904	O	PRO	115	A	<-->	2316	NE	ARG	225	B	2.41
269.	904	O	PRO	115	A	<-->	2317	CZ	ARG	225	B	2.33
270.	904	O	PRO	115	A	<-->	2318	NH1	ARG	225	B	3.59
271.	904	O	PRO	115	A	<-->	2319	NH2	ARG	225	B	1.69

272.	905	CB	PRO	115	A	<-->	2315	CD	ARG	225	B	3.66
273.	905	CB	PRO	115	A	<-->	2316	NE	ARG	225	B	2.78
274.	905	CB	PRO	115	A	<-->	2317	CZ	ARG	225	B	3.12
275.	905	CB	PRO	115	A	<-->	2319	NH2	ARG	225	B	3.18
276.	906	CG	PRO	115	A	<-->	2315	CD	ARG	225	B	3.08
277.	906	CG	PRO	115	A	<-->	2316	NE	ARG	225	B	2.42
278.	906	CG	PRO	115	A	<-->	2317	CZ	ARG	225	B	2.46
279.	906	CG	PRO	115	A	<-->	2318	NH1	ARG	225	B	3.02
280.	906	CG	PRO	115	A	<-->	2319	NH2	ARG	225	B	2.95
281.	907	CD	PRO	115	A	<-->	2316	NE	ARG	225	B	3.24
282.	907	CD	PRO	115	A	<-->	2317	CZ	ARG	225	B	2.58
283.	907	CD	PRO	115	A	<-->	2318	NH1	ARG	225	B	2.91
284.	907	CD	PRO	115	A	<-->	2319	NH2	ARG	225	B	2.55
285.	908	N	ARG	116	A	<-->	2319	NH2	ARG	225	B	3.61
286.	909	CA	ARG	116	A	<-->	2305	CG	LEU	224	B	3.82
287.	909	CA	ARG	116	A	<-->	2306	CD1	LEU	224	B	3.03
288.	909	CA	ARG	116	A	<-->	2307	CD2	LEU	224	B	3.53
289.	910	C	ARG	116	A	<-->	2306	CD1	LEU	224	B	3.37
290.	912	CB	ARG	116	A	<-->	2306	CD1	LEU	224	B	3.70
291.	912	CB	ARG	116	A	<-->	2307	CD2	LEU	224	B	3.27
292.	913	CG	ARG	116	A	<-->	2305	CG	LEU	224	B	3.77
293.	913	CG	ARG	116	A	<-->	2307	CD2	LEU	224	B	2.52
294.	914	CD	ARG	116	A	<-->	2304	CB	LEU	224	B	3.52
295.	914	CD	ARG	116	A	<-->	2305	CG	LEU	224	B	2.56
296.	914	CD	ARG	116	A	<-->	2306	CD1	LEU	224	B	3.22
297.	914	CD	ARG	116	A	<-->	2307	CD2	LEU	224	B	1.10
298.	915	NE	ARG	116	A	<-->	2304	CB	LEU	224	B	3.33
299.	915	NE	ARG	116	A	<-->	2305	CG	LEU	224	B	2.89
300.	915	NE	ARG	116	A	<-->	2306	CD1	LEU	224	B	3.36
301.	915	NE	ARG	116	A	<-->	2307	CD2	LEU	224	B	1.84
302.	916	CZ	ARG	116	A	<-->	2285	CD1	TRP	223	B	3.85
303.	916	CZ	ARG	116	A	<-->	2287	NE1	TRP	223	B	3.02
304.	916	CZ	ARG	116	A	<-->	2288	CE2	TRP	223	B	3.40
305.	916	CZ	ARG	116	A	<-->	2290	CZ2	TRP	223	B	3.64
306.	916	CZ	ARG	116	A	<-->	2304	CB	LEU	224	B	2.44
307.	916	CZ	ARG	116	A	<-->	2305	CG	LEU	224	B	2.67
308.	916	CZ	ARG	116	A	<-->	2306	CD1	LEU	224	B	3.35
309.	916	CZ	ARG	116	A	<-->	2307	CD2	LEU	224	B	2.19
310.	917	NH1	ARG	116	A	<-->	2285	CD1	TRP	223	B	3.31
311.	917	NH1	ARG	116	A	<-->	2287	NE1	TRP	223	B	2.90
312.	917	NH1	ARG	116	A	<-->	2288	CE2	TRP	223	B	3.68
313.	917	NH1	ARG	116	A	<-->	2300	N	LEU	224	B	3.49
314.	917	NH1	ARG	116	A	<-->	2301	CA	LEU	224	B	2.89
315.	917	NH1	ARG	116	A	<-->	2302	C	LEU	224	B	3.72
316.	917	NH1	ARG	116	A	<-->	2304	CB	LEU	224	B	1.42
317.	917	NH1	ARG	116	A	<-->	2305	CG	LEU	224	B	2.07
318.	917	NH1	ARG	116	A	<-->	2306	CD1	LEU	224	B	3.22
319.	917	NH1	ARG	116	A	<-->	2307	CD2	LEU	224	B	2.02
320.	918	NH2	ARG	116	A	<-->	2285	CD1	TRP	223	B	3.48
321.	918	NH2	ARG	116	A	<-->	2286	CD2	TRP	223	B	3.39
322.	918	NH2	ARG	116	A	<-->	2287	NE1	TRP	223	B	2.44
323.	918	NH2	ARG	116	A	<-->	2288	CE2	TRP	223	B	2.32
324.	918	NH2	ARG	116	A	<-->	2290	CZ2	TRP	223	B	2.31
325.	918	NH2	ARG	116	A	<-->	2292	CH2	TRP	223	B	3.40
326.	918	NH2	ARG	116	A	<-->	2304	CB	LEU	224	B	3.22
327.	918	NH2	ARG	116	A	<-->	2305	CG	LEU	224	B	3.80
328.	918	NH2	ARG	116	A	<-->	2307	CD2	LEU	224	B	3.54
329.	919	N	SER	117	A	<-->	2306	CD1	LEU	224	B	2.80
330.	923	CB	SER	117	A	<-->	4160	CG2	THR	410	B	3.79
331.	924	OG	SER	117	A	<-->	2306	CD1	LEU	224	B	3.80

332.	924	OG	SER	117	A	<-->	4158	CB	THR	410	B	3.86
333.	924	OG	SER	117	A	<-->	4160	CG2	THR	410	B	3.12
334.	936	CA	ARG	119	A	<-->	4926	CD1	TYR	484	B	3.25
335.	936	CA	ARG	119	A	<-->	4928	CE1	TYR	484	B	2.91
336.	937	C	ARG	119	A	<-->	4926	CD1	TYR	484	B	3.00
337.	937	C	ARG	119	A	<-->	4928	CE1	TYR	484	B	2.07
338.	937	C	ARG	119	A	<-->	4930	CZ	TYR	484	B	2.72
339.	937	C	ARG	119	A	<-->	4931	OH	TYR	484	B	2.85
340.	938	O	ARG	119	A	<-->	4925	CG	TYR	484	B	3.11
341.	938	O	ARG	119	A	<-->	4926	CD1	TYR	484	B	2.12
342.	938	O	ARG	119	A	<-->	4927	CD2	TYR	484	B	3.35
343.	938	O	ARG	119	A	<-->	4928	CE1	TYR	484	B	0.94
344.	938	O	ARG	119	A	<-->	4929	CE2	TYR	484	B	2.78
345.	938	O	ARG	119	A	<-->	4930	CZ	TYR	484	B	1.57
346.	938	O	ARG	119	A	<-->	4931	OH	TYR	484	B	2.09
347.	939	CB	ARG	119	A	<-->	4926	CD1	TYR	484	B	3.83
348.	939	CB	ARG	119	A	<-->	4928	CE1	TYR	484	B	3.55
349.	940	CG	ARG	119	A	<-->	4926	CD1	TYR	484	B	3.24
350.	940	CG	ARG	119	A	<-->	4928	CE1	TYR	484	B	3.19
351.	943	CZ	ARG	119	A	<-->	4946	CE1	PHE	485	B	3.07
352.	943	CZ	ARG	119	A	<-->	4948	CZ	PHE	485	B	3.68
353.	944	NH1	ARG	119	A	<-->	4944	CD1	PHE	485	B	3.35
354.	944	NH1	ARG	119	A	<-->	4946	CE1	PHE	485	B	2.20
355.	944	NH1	ARG	119	A	<-->	4948	CZ	PHE	485	B	2.77
356.	944	NH1	ARG	119	A	<-->	6493	CG	ASP	639	B	3.65
357.	944	NH1	ARG	119	A	<-->	6494	OD1	ASP	639	B	3.74
358.	944	NH1	ARG	119	A	<-->	6495	OD2	ASP	639	B	2.84
359.	945	NH2	ARG	119	A	<-->	4946	CE1	PHE	485	B	3.44
360.	945	NH2	ARG	119	A	<-->	4948	CZ	PHE	485	B	3.76
361.	945	NH2	ARG	119	A	<-->	6514	CD	GLN	641	B	3.55
362.	945	NH2	ARG	119	A	<-->	6516	NE2	GLN	641	B	3.09
363.	946	N	MET	120	A	<-->	4928	CE1	TYR	484	B	3.16
364.	946	N	MET	120	A	<-->	4930	CZ	TYR	484	B	3.41
365.	946	N	MET	120	A	<-->	4931	OH	TYR	484	B	2.97
366.	947	CA	MET	120	A	<-->	4928	CE1	TYR	484	B	3.53
367.	947	CA	MET	120	A	<-->	4930	CZ	TYR	484	B	3.26
368.	947	CA	MET	120	A	<-->	4931	OH	TYR	484	B	2.29
369.	948	C	MET	120	A	<-->	4928	CE1	TYR	484	B	3.71
370.	948	C	MET	120	A	<-->	4929	CE2	TYR	484	B	3.81
371.	948	C	MET	120	A	<-->	4930	CZ	TYR	484	B	3.00
372.	948	C	MET	120	A	<-->	4931	OH	TYR	484	B	2.09
373.	949	O	MET	120	A	<-->	4930	CZ	TYR	484	B	3.38
374.	949	O	MET	120	A	<-->	4931	OH	TYR	484	B	2.90
375.	950	CB	MET	120	A	<-->	4931	OH	TYR	484	B	3.63
376.	954	N	LEU	121	A	<-->	4929	CE2	TYR	484	B	3.76
377.	954	N	LEU	121	A	<-->	4930	CZ	TYR	484	B	3.23
378.	954	N	LEU	121	A	<-->	4931	OH	TYR	484	B	2.11
379.	955	CA	LEU	121	A	<-->	4184	CD	LYS	413	B	3.08
380.	955	CA	LEU	121	A	<-->	4929	CE2	TYR	484	B	3.84
381.	955	CA	LEU	121	A	<-->	4930	CZ	TYR	484	B	3.82
382.	955	CA	LEU	121	A	<-->	4931	OH	TYR	484	B	3.03
383.	956	C	LEU	121	A	<-->	4184	CD	LYS	413	B	3.37
384.	956	C	LEU	121	A	<-->	4186	NZ	LYS	413	B	3.58
385.	957	O	LEU	121	A	<-->	4176	CB	ALA	412	B	3.54
386.	957	O	LEU	121	A	<-->	4184	CD	LYS	413	B	3.27
387.	957	O	LEU	121	A	<-->	4186	NZ	LYS	413	B	3.51
388.	958	CB	LEU	121	A	<-->	4183	CG	LYS	413	B	2.97
389.	958	CB	LEU	121	A	<-->	4184	CD	LYS	413	B	1.82
390.	958	CB	LEU	121	A	<-->	4185	CE	LYS	413	B	2.94
391.	958	CB	LEU	121	A	<-->	4186	NZ	LYS	413	B	3.23

392.	958	CB	LEU	121	A	<-->	4931	OH	TYR	484	B	3.80
393.	959	CG	LEU	121	A	<-->	4183	CG	LYS	413	B	3.19
394.	959	CG	LEU	121	A	<-->	4184	CD	LYS	413	B	2.60
395.	959	CG	LEU	121	A	<-->	4185	CE	LYS	413	B	3.76
396.	959	CG	LEU	121	A	<-->	4931	OH	TYR	484	B	3.51
397.	960	CD1	LEU	121	A	<-->	4183	CG	LYS	413	B	2.90
398.	960	CD1	LEU	121	A	<-->	4184	CD	LYS	413	B	2.62
399.	960	CD1	LEU	121	A	<-->	4185	CE	LYS	413	B	3.39
400.	960	CD1	LEU	121	A	<-->	4972	CA	GLY	488	B	3.62
401.	960	CD1	LEU	121	A	<-->	6553	CG2	VAL	645	B	3.10
402.	961	CD2	LEU	121	A	<-->	4183	CG	LYS	413	B	3.46
403.	961	CD2	LEU	121	A	<-->	4184	CD	LYS	413	B	3.42
404.	961	CD2	LEU	121	A	<-->	4929	CE2	TYR	484	B	2.92
405.	961	CD2	LEU	121	A	<-->	4930	CZ	TYR	484	B	3.53
406.	961	CD2	LEU	121	A	<-->	4931	OH	TYR	484	B	3.33
407.	961	CD2	LEU	121	A	<-->	4941	O	PHE	485	B	3.10
408.	962	N	LEU	122	A	<-->	4186	NZ	LYS	413	B	3.89
409.	969	CD2	LEU	122	A	<-->	4186	NZ	LYS	413	B	2.73
410.	1152	CE2	TYR	145	A	<-->	5983	CD2	LEU	589	B	3.26
411.	1153	CZ	TYR	145	A	<-->	5983	CD2	LEU	589	B	3.77
412.	1154	OH	TYR	145	A	<-->	5983	CD2	LEU	589	B	3.38
413.	1162	N	CYS	147	A	<-->	4235	CG	PRO	418	B	2.96
414.	1162	N	CYS	147	A	<-->	4236	CD	PRO	418	B	3.33
415.	1163	CA	CYS	147	A	<-->	4234	CB	PRO	418	B	3.48
416.	1163	CA	CYS	147	A	<-->	4235	CG	PRO	418	B	2.19
417.	1163	CA	CYS	147	A	<-->	4236	CD	PRO	418	B	3.20
418.	1164	C	CYS	147	A	<-->	4235	CG	PRO	418	B	3.49
419.	1166	CB	CYS	147	A	<-->	4230	N	PRO	418	B	3.28
420.	1166	CB	CYS	147	A	<-->	4231	CA	PRO	418	B	3.39
421.	1166	CB	CYS	147	A	<-->	4234	CB	PRO	418	B	2.69
422.	1166	CB	CYS	147	A	<-->	4235	CG	PRO	418	B	1.46
423.	1166	CB	CYS	147	A	<-->	4236	CD	PRO	418	B	2.51
424.	1167	SG	CYS	147	A	<-->	4226	CB	SER	417	B	3.69
425.	1167	SG	CYS	147	A	<-->	4235	CG	PRO	418	B	3.10
426.	1167	SG	CYS	147	A	<-->	4236	CD	PRO	418	B	3.59
427.	1629	CG	ASP	207	A	<-->	2290	CZ2	TRP	223	B	3.75
428.	1631	OD2	ASP	207	A	<-->	2287	NE1	TRP	223	B	3.59
429.	1631	OD2	ASP	207	A	<-->	2288	CE2	TRP	223	B	3.37
430.	1631	OD2	ASP	207	A	<-->	2290	CZ2	TRP	223	B	2.66
431.	1631	OD2	ASP	207	A	<-->	2292	CH2	TRP	223	B	3.62
432.	4251	CB	VAL	547	A	<-->	11414	O	ILE	1144	B	3.88
433.	4251	CB	VAL	547	A	<-->	11549	CB	LYS	1158	B	3.18
434.	4251	CB	VAL	547	A	<-->	11550	CG	LYS	1158	B	3.85
435.	4251	CB	VAL	547	A	<-->	11561	O	LEU	1159	B	3.18
436.	4252	CG1	VAL	547	A	<-->	11546	CA	LYS	1158	B	3.63
437.	4252	CG1	VAL	547	A	<-->	11549	CB	LYS	1158	B	2.34
438.	4252	CG1	VAL	547	A	<-->	11550	CG	LYS	1158	B	2.86
439.	4252	CG1	VAL	547	A	<-->	11551	CD	LYS	1158	B	2.73
440.	4252	CG1	VAL	547	A	<-->	11552	CE	LYS	1158	B	3.61
441.	4253	CG2	VAL	547	A	<-->	11407	O	PRO	1143	B	3.27
442.	4253	CG2	VAL	547	A	<-->	11413	C	ILE	1144	B	3.51
443.	4253	CG2	VAL	547	A	<-->	11414	O	ILE	1144	B	2.41
444.	4253	CG2	VAL	547	A	<-->	11417	CG2	ILE	1144	B	3.32
445.	4253	CG2	VAL	547	A	<-->	11546	CA	LYS	1158	B	3.66
446.	4253	CG2	VAL	547	A	<-->	11549	CB	LYS	1158	B	3.26
447.	4253	CG2	VAL	547	A	<-->	11550	CG	LYS	1158	B	3.72
448.	4253	CG2	VAL	547	A	<-->	11558	N	LEU	1159	B	3.61
449.	4253	CG2	VAL	547	A	<-->	11561	O	LEU	1159	B	3.18
450.	4254	N	PRO	548	A	<-->	11407	O	PRO	1143	B	3.19
451.	4255	CA	PRO	548	A	<-->	11407	O	PRO	1143	B	3.76

452.	4257	O	PRO	548	A	<-->	11550	CG	LYS	1158	B	3.73
453.	4257	O	PRO	548	A	<-->	11551	CD	LYS	1158	B	3.36
454.	4258	CB	PRO	548	A	<-->	11405	CA	PRO	1143	B	3.60
455.	4258	CB	PRO	548	A	<-->	11406	C	PRO	1143	B	3.78
456.	4258	CB	PRO	548	A	<-->	11407	O	PRO	1143	B	3.09
457.	4258	CB	PRO	548	A	<-->	11408	CB	PRO	1143	B	2.92
458.	4259	CG	PRO	548	A	<-->	11405	CA	PRO	1143	B	2.94
459.	4259	CG	PRO	548	A	<-->	11406	C	PRO	1143	B	3.00
460.	4259	CG	PRO	548	A	<-->	11407	O	PRO	1143	B	2.36
461.	4259	CG	PRO	548	A	<-->	11408	CB	PRO	1143	B	2.98
462.	4260	CD	PRO	548	A	<-->	11405	CA	PRO	1143	B	3.88
463.	4260	CD	PRO	548	A	<-->	11406	C	PRO	1143	B	3.29
464.	4260	CD	PRO	548	A	<-->	11407	O	PRO	1143	B	2.25
465.	4260	CD	PRO	548	A	<-->	11417	CG2	ILE	1144	B	3.61
466.	4265	CB	VAL	549	A	<-->	11553	NZ	LYS	1158	B	3.72
467.	4267	CG2	VAL	549	A	<-->	11550	CG	LYS	1158	B	3.72
468.	4267	CG2	VAL	549	A	<-->	11551	CD	LYS	1158	B	2.39
469.	4267	CG2	VAL	549	A	<-->	11552	CE	LYS	1158	B	3.04
470.	4267	CG2	VAL	549	A	<-->	11553	NZ	LYS	1158	B	2.71
471.	4925	CA	ALA	636	A	<-->	11578	O	SER	1161	B	3.65
472.	4926	C	ALA	636	A	<-->	11576	CA	SER	1161	B	3.86
473.	4926	C	ALA	636	A	<-->	11577	C	SER	1161	B	2.89
474.	4926	C	ALA	636	A	<-->	11578	O	SER	1161	B	2.28
475.	4926	C	ALA	636	A	<-->	11579	CB	SER	1161	B	3.56
476.	4926	C	ALA	636	A	<-->	11583	N	VAL	1162	B	3.54
477.	4926	C	ALA	636	A	<-->	11584	CA	VAL	1162	B	3.64
478.	4927	O	ALA	636	A	<-->	11575	N	SER	1161	B	3.69
479.	4927	O	ALA	636	A	<-->	11576	CA	SER	1161	B	2.66
480.	4927	O	ALA	636	A	<-->	11577	C	SER	1161	B	1.78
481.	4927	O	ALA	636	A	<-->	11578	O	SER	1161	B	1.33
482.	4927	O	ALA	636	A	<-->	11579	CB	SER	1161	B	2.52
483.	4927	O	ALA	636	A	<-->	11583	N	VAL	1162	B	2.72
484.	4927	O	ALA	636	A	<-->	11584	CA	VAL	1162	B	3.25
485.	4928	CB	ALA	636	A	<-->	11585	C	VAL	1162	B	3.81
486.	4928	CB	ALA	636	A	<-->	11591	N	ASN	1163	B	3.51
487.	4929	N	ALA	637	A	<-->	11577	C	SER	1161	B	3.63
488.	4929	N	ALA	637	A	<-->	11578	O	SER	1161	B	2.74
489.	4929	N	ALA	637	A	<-->	11584	CA	VAL	1162	B	3.84
490.	4930	CA	ALA	637	A	<-->	11577	C	SER	1161	B	3.71
491.	4930	CA	ALA	637	A	<-->	11578	O	SER	1161	B	2.64
492.	4930	CA	ALA	637	A	<-->	11584	CA	VAL	1162	B	3.72
493.	4931	C	ALA	637	A	<-->	11577	C	SER	1161	B	3.73
494.	4931	C	ALA	637	A	<-->	11578	O	SER	1161	B	2.50
495.	4932	O	ALA	637	A	<-->	11572	CG1	VAL	1160	B	3.44
496.	4932	O	ALA	637	A	<-->	11578	O	SER	1161	B	2.87
497.	4934	N	ASN	638	A	<-->	11578	O	SER	1161	B	2.97
498.	4938	CB	ASN	638	A	<-->	11572	CG1	VAL	1160	B	3.52

Salt bridges

<----- A T O M 1 -----> <----- A T O M 2 ----->

	Atom no.	Atom name	Res name	Res no.	Chain		Atom no.	Atom name	Res name	Res no.	Chain	Distance
1.	844	NH2	ARG	107	A	<-->	3667	OE1	GLU	362	B	3.94
2.	841	NE	ARG	107	A	<-->	5376	OE2	GLU	529	B	1.27
3.	859	OE1	GLU	109	A	<-->	2348	NH2	ARG	227	B	1.64
4.	859	OE1	GLU	109	A	<-->	5387	NE2	HIS	530	B	3.83
5.	899	OD1	ASP	114	A	<-->	2319	NH2	ARG	225	B	2.14

6. 944 NH1 ARG 119 A <--> 6495 OD2 ASP 639 B 2.84

Number of salt bridges: 6

Number of hydrogen bonds: 10

Number of non-bonded contacts: 498

[E] G829W + SLIT2 (WT) _FULL PROTEIN

Hydrogen bonds

	<----- A T O M 1 ----->						<----- A T O M 2 ----->					
	Atom no.	Atom name	Res name	Res no.	Chain		Atom no.	Atom name	Res name	Res no.	Chain	Distance
1.	272	NE2	GLN	39	A	<-->	11081	O	TYR	444	B	3.08
2.	288	OE1	GLN	41	A	<-->	11312	OG	SER	473	B	2.94
3.	3248	NH1	ARG	433	A	<-->	18066	OG	SER	1349	B	3.10
4.	3387	OG	SER	451	A	<-->	18057	O	PRO	1348	B	2.82
5.	3395	OE1	GLU	452	A	<-->	18082	N	ALA	1352	B	2.03
6.	3675	O	ILE	489	A	<-->	13311	NH2	ARG	726	B	1.74

Non-bonded contacts

	<----- A T O M 1 ----->						<----- A T O M 2 ----->					
	Atom no.	Atom name	Res name	Res no.	Chain		Atom no.	Atom name	Res name	Res no.	Chain	Distance
1.	261	CB	PRO	38	A	<-->	11114	CG2	THR	447	B	3.57
2.	268	CB	GLN	39	A	<-->	11094	CB	LEU	445	B	3.67
3.	269	CG	GLN	39	A	<-->	11094	CB	LEU	445	B	3.13
4.	269	CG	GLN	39	A	<-->	11095	CG	LEU	445	B	3.41
5.	270	CD	GLN	39	A	<-->	11081	O	TYR	444	B	3.43
6.	270	CD	GLN	39	A	<-->	11091	CA	LEU	445	B	3.16
7.	270	CD	GLN	39	A	<-->	11094	CB	LEU	445	B	1.66
8.	270	CD	GLN	39	A	<-->	11095	CG	LEU	445	B	2.09
9.	270	CD	GLN	39	A	<-->	11096	CD1	LEU	445	B	3.21
10.	270	CD	GLN	39	A	<-->	11097	CD2	LEU	445	B	3.17
11.	271	OE1	GLN	39	A	<-->	11080	C	TYR	444	B	3.76
12.	271	OE1	GLN	39	A	<-->	11081	O	TYR	444	B	3.28
13.	271	OE1	GLN	39	A	<-->	11090	N	LEU	445	B	3.39
14.	271	OE1	GLN	39	A	<-->	11091	CA	LEU	445	B	2.24
15.	271	OE1	GLN	39	A	<-->	11092	C	LEU	445	B	3.11
16.	271	OE1	GLN	39	A	<-->	11094	CB	LEU	445	B	1.06
17.	271	OE1	GLN	39	A	<-->	11095	CG	LEU	445	B	2.29
18.	271	OE1	GLN	39	A	<-->	11096	CD1	LEU	445	B	3.47
19.	271	OE1	GLN	39	A	<-->	11097	CD2	LEU	445	B	3.15
20.	271	OE1	GLN	39	A	<-->	11098	N	HIS	446	B	3.56
21.	272	NE2	GLN	39	A	<-->	11077	OD2	ASP	443	B	3.77
22.	272	NE2	GLN	39	A	<-->	11080	C	TYR	444	B	3.72
23.	272	NE2	GLN	39	A	<-->	11081	O	TYR	444	B	3.08
24.	272	NE2	GLN	39	A	<-->	11090	N	LEU	445	B	3.83
25.	272	NE2	GLN	39	A	<-->	11091	CA	LEU	445	B	3.42
26.	272	NE2	GLN	39	A	<-->	11094	CB	LEU	445	B	2.02
27.	272	NE2	GLN	39	A	<-->	11095	CG	LEU	445	B	1.59
28.	272	NE2	GLN	39	A	<-->	11096	CD1	LEU	445	B	2.19
29.	272	NE2	GLN	39	A	<-->	11097	CD2	LEU	445	B	2.98
30.	278	CG	ASP	40	A	<-->	11081	O	TYR	444	B	3.63
31.	280	OD2	ASP	40	A	<-->	11081	O	TYR	444	B	3.13
32.	282	CA	GLN	41	A	<-->	11836	CZ	ARG	539	B	3.47
33.	282	CA	GLN	41	A	<-->	11837	NH1	ARG	539	B	3.21
34.	282	CA	GLN	41	A	<-->	11838	NH2	ARG	539	B	2.92
35.	283	C	GLN	41	A	<-->	11836	CZ	ARG	539	B	3.16
36.	283	C	GLN	41	A	<-->	11837	NH1	ARG	539	B	3.14

37.	283	C	GLN	41	A	<-->	11838	NH2	ARG	539	B	2.76
38.	284	O	GLN	41	A	<-->	11319	CD	LYS	474	B	3.40
39.	284	O	GLN	41	A	<-->	11320	CE	LYS	474	B	3.85
40.	284	O	GLN	41	A	<-->	11835	NE	ARG	539	B	3.35
41.	284	O	GLN	41	A	<-->	11836	CZ	ARG	539	B	2.44
42.	284	O	GLN	41	A	<-->	11837	NH1	ARG	539	B	2.38
43.	284	O	GLN	41	A	<-->	11838	NH2	ARG	539	B	2.56
44.	285	CB	GLN	41	A	<-->	11836	CZ	ARG	539	B	2.65
45.	285	CB	GLN	41	A	<-->	11837	NH1	ARG	539	B	2.08
46.	285	CB	GLN	41	A	<-->	11838	NH2	ARG	539	B	2.51
47.	286	CG	GLN	41	A	<-->	11834	CD	ARG	539	B	3.80
48.	286	CG	GLN	41	A	<-->	11835	NE	ARG	539	B	2.77
49.	286	CG	GLN	41	A	<-->	11836	CZ	ARG	539	B	1.48
50.	286	CG	GLN	41	A	<-->	11837	NH1	ARG	539	B	1.66
51.	286	CG	GLN	41	A	<-->	11838	NH2	ARG	539	B	1.31
52.	287	CD	GLN	41	A	<-->	11834	CD	ARG	539	B	3.34
53.	287	CD	GLN	41	A	<-->	11835	NE	ARG	539	B	2.77
54.	287	CD	GLN	41	A	<-->	11836	CZ	ARG	539	B	1.96
55.	287	CD	GLN	41	A	<-->	11837	NH1	ARG	539	B	1.77
56.	287	CD	GLN	41	A	<-->	11838	NH2	ARG	539	B	2.51
57.	287	CD	GLN	41	A	<-->	11852	CG	ASN	541	B	3.65
58.	287	CD	GLN	41	A	<-->	11853	OD1	ASN	541	B	3.59
59.	287	CD	GLN	41	A	<-->	11854	ND2	ASN	541	B	2.88
60.	288	OE1	GLN	41	A	<-->	11310	O	SER	473	B	3.63
61.	288	OE1	GLN	41	A	<-->	11312	OG	SER	473	B	2.94
62.	288	OE1	GLN	41	A	<-->	11834	CD	ARG	539	B	3.41
63.	288	OE1	GLN	41	A	<-->	11835	NE	ARG	539	B	3.34
64.	288	OE1	GLN	41	A	<-->	11836	CZ	ARG	539	B	2.64
65.	288	OE1	GLN	41	A	<-->	11837	NH1	ARG	539	B	1.80
66.	288	OE1	GLN	41	A	<-->	11838	NH2	ARG	539	B	3.49
67.	288	OE1	GLN	41	A	<-->	11852	CG	ASN	541	B	3.04
68.	288	OE1	GLN	41	A	<-->	11853	OD1	ASN	541	B	2.81
69.	288	OE1	GLN	41	A	<-->	11854	ND2	ASN	541	B	2.52
70.	289	NE2	GLN	41	A	<-->	11834	CD	ARG	539	B	3.64
71.	289	NE2	GLN	41	A	<-->	11835	NE	ARG	539	B	3.04
72.	289	NE2	GLN	41	A	<-->	11836	CZ	ARG	539	B	2.69
73.	289	NE2	GLN	41	A	<-->	11837	NH1	ARG	539	B	3.00
74.	289	NE2	GLN	41	A	<-->	11838	NH2	ARG	539	B	3.03
75.	289	NE2	GLN	41	A	<-->	11852	CG	ASN	541	B	3.64
76.	289	NE2	GLN	41	A	<-->	11854	ND2	ASN	541	B	2.58
77.	290	N	LEU	42	A	<-->	11838	NH2	ARG	539	B	3.66
78.	295	CG	LEU	42	A	<-->	11319	CD	LYS	474	B	3.87
79.	295	CG	LEU	42	A	<-->	11320	CE	LYS	474	B	3.13
80.	295	CG	LEU	42	A	<-->	11321	NZ	LYS	474	B	3.32
81.	296	CD1	LEU	42	A	<-->	11319	CD	LYS	474	B	3.03
82.	296	CD1	LEU	42	A	<-->	11320	CE	LYS	474	B	1.86
83.	296	CD1	LEU	42	A	<-->	11321	NZ	LYS	474	B	2.22
84.	297	CD2	LEU	42	A	<-->	11319	CD	LYS	474	B	3.52
85.	297	CD2	LEU	42	A	<-->	11320	CE	LYS	474	B	3.43
86.	297	CD2	LEU	42	A	<-->	11321	NZ	LYS	474	B	3.70
87.	620	CG	ASP	85	A	<-->	14134	CD2	LEU	830	B	3.69
88.	622	OD2	ASP	85	A	<-->	14132	CG	LEU	830	B	3.03
89.	622	OD2	ASP	85	A	<-->	14133	CD1	LEU	830	B	3.29
90.	622	OD2	ASP	85	A	<-->	14134	CD2	LEU	830	B	2.66
91.	632	OG1	THR	87	A	<-->	14147	CD1	LEU	832	B	3.23
92.	793	CB	LEU	109	A	<-->	14148	CD2	LEU	832	B	3.45
93.	794	CG	LEU	109	A	<-->	14146	CG	LEU	832	B	3.71
94.	794	CG	LEU	109	A	<-->	14147	CD1	LEU	832	B	3.11
95.	794	CG	LEU	109	A	<-->	14148	CD2	LEU	832	B	3.54
96.	796	CD2	LEU	109	A	<-->	14146	CG	LEU	832	B	3.71

97.	796	CD2	LEU	109	A	<-->	14147	CD1	LEU	832	B	2.72
98.	796	CD2	LEU	109	A	<-->	14148	CD2	LEU	832	B	3.63
99.	832	SG	CYS	114	A	<-->	11112	CB	THR	447	B	2.96
100.	832	SG	CYS	114	A	<-->	11114	CG2	THR	447	B	2.61
101.	838	CG	GLU	115	A	<-->	11114	CG2	THR	447	B	3.62
102.	839	CD	GLU	115	A	<-->	11114	CG2	THR	447	B	3.61
103.	840	OE1	GLU	115	A	<-->	11114	CG2	THR	447	B	2.78
104.	1296	CA	ASP	174	A	<-->	14166	O	ASN	835	B	3.62
105.	1297	C	ASP	174	A	<-->	14166	O	ASN	835	B	3.20
106.	1298	O	ASP	174	A	<-->	14166	O	ASN	835	B	3.68
107.	1299	CB	ASP	174	A	<-->	14165	C	ASN	835	B	3.57
108.	1299	CB	ASP	174	A	<-->	14166	O	ASN	835	B	2.76
109.	1299	CB	ASP	174	A	<-->	14171	N	ASP	836	B	3.63
110.	1299	CB	ASP	174	A	<-->	14172	CA	ASP	836	B	2.79
111.	1299	CB	ASP	174	A	<-->	14173	C	ASP	836	B	3.43
112.	1299	CB	ASP	174	A	<-->	14175	CB	ASP	836	B	3.62
113.	1299	CB	ASP	174	A	<-->	14176	CG	ASP	836	B	3.74
114.	1299	CB	ASP	174	A	<-->	14177	OD1	ASP	836	B	3.25
115.	1299	CB	ASP	174	A	<-->	14179	N	ILE	837	B	3.19
116.	1300	CG	ASP	174	A	<-->	14172	CA	ASP	836	B	2.92
117.	1300	CG	ASP	174	A	<-->	14173	C	ASP	836	B	3.23
118.	1300	CG	ASP	174	A	<-->	14175	CB	ASP	836	B	3.29
119.	1300	CG	ASP	174	A	<-->	14176	CG	ASP	836	B	3.67
120.	1300	CG	ASP	174	A	<-->	14177	OD1	ASP	836	B	3.67
121.	1300	CG	ASP	174	A	<-->	14179	N	ILE	837	B	2.66
122.	1300	CG	ASP	174	A	<-->	14180	CA	ILE	837	B	3.85
123.	1300	CG	ASP	174	A	<-->	14182	O	ILE	837	B	3.54
124.	1301	OD1	ASP	174	A	<-->	14179	N	ILE	837	B	3.39
125.	1301	OD1	ASP	174	A	<-->	14182	O	ILE	837	B	3.32
126.	1302	OD2	ASP	174	A	<-->	14171	N	ASP	836	B	3.73
127.	1302	OD2	ASP	174	A	<-->	14172	CA	ASP	836	B	2.31
128.	1302	OD2	ASP	174	A	<-->	14173	C	ASP	836	B	2.58
129.	1302	OD2	ASP	174	A	<-->	14174	O	ASP	836	B	3.72
130.	1302	OD2	ASP	174	A	<-->	14175	CB	ASP	836	B	2.26
131.	1302	OD2	ASP	174	A	<-->	14176	CG	ASP	836	B	2.96
132.	1302	OD2	ASP	174	A	<-->	14177	OD1	ASP	836	B	3.36
133.	1302	OD2	ASP	174	A	<-->	14178	OD2	ASP	836	B	3.75
134.	1302	OD2	ASP	174	A	<-->	14179	N	ILE	837	B	2.28
135.	1302	OD2	ASP	174	A	<-->	14180	CA	ILE	837	B	3.55
136.	1302	OD2	ASP	174	A	<-->	14181	C	ILE	837	B	3.83
137.	1302	OD2	ASP	174	A	<-->	14182	O	ILE	837	B	3.33
138.	1303	N	GLY	175	A	<-->	14166	O	ASN	835	B	3.11
139.	1304	CA	GLY	175	A	<-->	14160	CA	GLY	834	B	3.80
140.	1304	CA	GLY	175	A	<-->	14166	O	ASN	835	B	3.57
141.	1305	C	GLY	175	A	<-->	14160	CA	GLY	834	B	3.39
142.	1306	O	GLY	175	A	<-->	14152	O	HIS	833	B	3.52
143.	1306	O	GLY	175	A	<-->	14160	CA	GLY	834	B	3.52
144.	1307	N	LYS	176	A	<-->	14160	CA	GLY	834	B	3.65
145.	1308	CA	LYS	176	A	<-->	14152	O	HIS	833	B	3.55
146.	1308	CA	LYS	176	A	<-->	14156	CD2	HIS	833	B	2.78
147.	1308	CA	LYS	176	A	<-->	14158	NE2	HIS	833	B	3.16
148.	1309	C	LYS	176	A	<-->	14151	C	HIS	833	B	3.67
149.	1309	C	LYS	176	A	<-->	14152	O	HIS	833	B	3.50
150.	1309	C	LYS	176	A	<-->	14153	CB	HIS	833	B	3.67
151.	1309	C	LYS	176	A	<-->	14154	CG	HIS	833	B	3.04
152.	1309	C	LYS	176	A	<-->	14156	CD2	HIS	833	B	1.95
153.	1309	C	LYS	176	A	<-->	14158	NE2	HIS	833	B	2.84
154.	1310	O	LYS	176	A	<-->	14154	CG	HIS	833	B	3.84
155.	1310	O	LYS	176	A	<-->	14156	CD2	HIS	833	B	2.92
156.	1310	O	LYS	176	A	<-->	14158	NE2	HIS	833	B	3.87

157.	1311	CB	LYS	176	A	<-->	14156	CD2	HIS	833	B	3.38
158.	1311	CB	LYS	176	A	<-->	14158	NE2	HIS	833	B	3.20
159.	1316	N	PRO	177	A	<-->	14150	CA	HIS	833	B	3.43
160.	1316	N	PRO	177	A	<-->	14151	C	HIS	833	B	3.11
161.	1316	N	PRO	177	A	<-->	14152	O	HIS	833	B	3.01
162.	1316	N	PRO	177	A	<-->	14153	CB	HIS	833	B	2.56
163.	1316	N	PRO	177	A	<-->	14154	CG	HIS	833	B	1.73
164.	1316	N	PRO	177	A	<-->	14155	ND1	HIS	833	B	2.83
165.	1316	N	PRO	177	A	<-->	14156	CD2	HIS	833	B	0.77
166.	1316	N	PRO	177	A	<-->	14157	CE1	HIS	833	B	2.91
167.	1316	N	PRO	177	A	<-->	14158	NE2	HIS	833	B	2.06
168.	1316	N	PRO	177	A	<-->	14159	N	GLY	834	B	3.76
169.	1317	CA	PRO	177	A	<-->	14150	CA	HIS	833	B	3.22
170.	1317	CA	PRO	177	A	<-->	14151	C	HIS	833	B	3.48
171.	1317	CA	PRO	177	A	<-->	14152	O	HIS	833	B	3.81
172.	1317	CA	PRO	177	A	<-->	14153	CB	HIS	833	B	1.87
173.	1317	CA	PRO	177	A	<-->	14154	CG	HIS	833	B	1.56
174.	1317	CA	PRO	177	A	<-->	14155	ND1	HIS	833	B	2.74
175.	1317	CA	PRO	177	A	<-->	14156	CD2	HIS	833	B	1.62
176.	1317	CA	PRO	177	A	<-->	14157	CE1	HIS	833	B	3.25
177.	1317	CA	PRO	177	A	<-->	14158	NE2	HIS	833	B	2.81
178.	1318	C	PRO	177	A	<-->	14153	CB	HIS	833	B	3.02
179.	1318	C	PRO	177	A	<-->	14154	CG	HIS	833	B	2.42
180.	1318	C	PRO	177	A	<-->	14155	ND1	HIS	833	B	3.00
181.	1318	C	PRO	177	A	<-->	14156	CD2	HIS	833	B	2.34
182.	1318	C	PRO	177	A	<-->	14157	CE1	HIS	833	B	3.22
183.	1318	C	PRO	177	A	<-->	14158	NE2	HIS	833	B	2.91
184.	1319	O	PRO	177	A	<-->	14153	CB	HIS	833	B	3.83
185.	1319	O	PRO	177	A	<-->	14154	CG	HIS	833	B	2.78
186.	1319	O	PRO	177	A	<-->	14155	ND1	HIS	833	B	2.94
187.	1319	O	PRO	177	A	<-->	14156	CD2	HIS	833	B	2.35
188.	1319	O	PRO	177	A	<-->	14157	CE1	HIS	833	B	2.64
189.	1319	O	PRO	177	A	<-->	14158	NE2	HIS	833	B	2.28
190.	1320	CB	PRO	177	A	<-->	14142	CA	LEU	832	B	3.58
191.	1320	CB	PRO	177	A	<-->	14143	C	LEU	832	B	3.54
192.	1320	CB	PRO	177	A	<-->	14145	CB	LEU	832	B	3.56
193.	1320	CB	PRO	177	A	<-->	14149	N	HIS	833	B	2.88
194.	1320	CB	PRO	177	A	<-->	14150	CA	HIS	833	B	2.07
195.	1320	CB	PRO	177	A	<-->	14151	C	HIS	833	B	2.91
196.	1320	CB	PRO	177	A	<-->	14152	O	HIS	833	B	3.42
197.	1320	CB	PRO	177	A	<-->	14153	CB	HIS	833	B	0.55
198.	1320	CB	PRO	177	A	<-->	14154	CG	HIS	833	B	1.20
199.	1320	CB	PRO	177	A	<-->	14155	ND1	HIS	833	B	2.21
200.	1320	CB	PRO	177	A	<-->	14156	CD2	HIS	833	B	2.33
201.	1320	CB	PRO	177	A	<-->	14157	CE1	HIS	833	B	3.28
202.	1320	CB	PRO	177	A	<-->	14158	NE2	HIS	833	B	3.39
203.	1320	CB	PRO	177	A	<-->	14159	N	GLY	834	B	3.74
204.	1321	CG	PRO	177	A	<-->	14142	CA	LEU	832	B	3.86
205.	1321	CG	PRO	177	A	<-->	14143	C	LEU	832	B	3.19
206.	1321	CG	PRO	177	A	<-->	14149	N	HIS	833	B	2.22
207.	1321	CG	PRO	177	A	<-->	14150	CA	HIS	833	B	2.00
208.	1321	CG	PRO	177	A	<-->	14151	C	HIS	833	B	2.51
209.	1321	CG	PRO	177	A	<-->	14152	O	HIS	833	B	2.51
210.	1321	CG	PRO	177	A	<-->	14153	CB	HIS	833	B	1.55
211.	1321	CG	PRO	177	A	<-->	14154	CG	HIS	833	B	0.98
212.	1321	CG	PRO	177	A	<-->	14155	ND1	HIS	833	B	1.49
213.	1321	CG	PRO	177	A	<-->	14156	CD2	HIS	833	B	2.07
214.	1321	CG	PRO	177	A	<-->	14157	CE1	HIS	833	B	2.50
215.	1321	CG	PRO	177	A	<-->	14158	NE2	HIS	833	B	2.80
216.	1321	CG	PRO	177	A	<-->	14159	N	GLY	834	B	3.73

217. 1322 CD PRO 177 A <--> 14149 N HIS 833 B 3.26
218. 1322 CD PRO 177 A <--> 14150 CA HIS 833 B 2.70
219. 1322 CD PRO 177 A <--> 14151 C HIS 833 B 2.26
220. 1322 CD PRO 177 A <--> 14152 O HIS 833 B 1.82
221. 1322 CD PRO 177 A <--> 14153 CB HIS 833 B 2.40
222. 1322 CD PRO 177 A <--> 14154 CG HIS 833 B 1.57
223. 1322 CD PRO 177 A <--> 14155 ND1 HIS 833 B 2.41
224. 1322 CD PRO 177 A <--> 14156 CD2 HIS 833 B 1.40
225. 1322 CD PRO 177 A <--> 14157 CE1 HIS 833 B 2.69
226. 1322 CD PRO 177 A <--> 14158 NE2 HIS 833 B 2.27
227. 1322 CD PRO 177 A <--> 14159 N GLY 834 B 3.31
228. 1323 N LEU 178 A <--> 14153 CB HIS 833 B 3.62
229. 1323 N LEU 178 A <--> 14154 CG HIS 833 B 3.48
230. 1323 N LEU 178 A <--> 14156 CD2 HIS 833 B 3.65
231. 1335 CB ALA 179 A <--> 14148 CD2 LEU 832 B 3.31
232. 1756 CG2 VAL 235 A <--> 14146 CG LEU 832 B 3.84
233. 2014 NE1 TRP 268 A <--> 15112 N CYS 961 B 3.57
234. 2014 NE1 TRP 268 A <--> 15113 CA CYS 961 B 3.16
235. 2014 NE1 TRP 268 A <--> 15116 CB CYS 961 B 3.52
236. 2014 NE1 TRP 268 A <--> 15117 SG CYS 961 B 2.98
237. 2015 CE2 TRP 268 A <--> 15117 SG CYS 961 B 2.82
238. 2017 CZ2 TRP 268 A <--> 15116 CB CYS 961 B 3.78
239. 2017 CZ2 TRP 268 A <--> 15117 SG CYS 961 B 2.17
240. 2019 CH2 TRP 268 A <--> 13955 O SER 809 B 3.70
241. 2019 CH2 TRP 268 A <--> 15117 SG CYS 961 B 3.32
242. 2506 N ASP 334 A <--> 17650 CZ ARG 1294 B 3.37
243. 2506 N ASP 334 A <--> 17651 NH1 ARG 1294 B 3.10
244. 2506 N ASP 334 A <--> 17652 NH2 ARG 1294 B 3.74
245. 2507 CA ASP 334 A <--> 17649 NE ARG 1294 B 3.50
246. 2507 CA ASP 334 A <--> 17650 CZ ARG 1294 B 2.48
247. 2507 CA ASP 334 A <--> 17651 NH1 ARG 1294 B 2.31
248. 2507 CA ASP 334 A <--> 17652 NH2 ARG 1294 B 2.54
249. 2508 C ASP 334 A <--> 17650 CZ ARG 1294 B 3.26
250. 2508 C ASP 334 A <--> 17651 NH1 ARG 1294 B 3.53
251. 2508 C ASP 334 A <--> 17652 NH2 ARG 1294 B 2.66
252. 2509 O ASP 334 A <--> 15111 CB ALA 960 B 3.41
253. 2509 O ASP 334 A <--> 17652 NH2 ARG 1294 B 3.05
254. 2510 CB ASP 334 A <--> 17648 CD ARG 1294 B 3.01
255. 2510 CB ASP 334 A <--> 17649 NE ARG 1294 B 2.07
256. 2510 CB ASP 334 A <--> 17650 CZ ARG 1294 B 0.94
257. 2510 CB ASP 334 A <--> 17651 NH1 ARG 1294 B 1.30
258. 2510 CB ASP 334 A <--> 17652 NH2 ARG 1294 B 1.49
259. 2511 CG ASP 334 A <--> 17648 CD ARG 1294 B 2.75
260. 2511 CG ASP 334 A <--> 17649 NE ARG 1294 B 1.51
261. 2511 CG ASP 334 A <--> 17650 CZ ARG 1294 B 1.46
262. 2511 CG ASP 334 A <--> 17651 NH1 ARG 1294 B 2.58
263. 2511 CG ASP 334 A <--> 17652 NH2 ARG 1294 B 1.74
264. 2512 OD1 ASP 334 A <--> 17648 CD ARG 1294 B 3.27
265. 2512 OD1 ASP 334 A <--> 17649 NE ARG 1294 B 2.43
266. 2512 OD1 ASP 334 A <--> 17650 CZ ARG 1294 B 2.62
267. 2512 OD1 ASP 334 A <--> 17651 NH1 ARG 1294 B 3.47
268. 2512 OD1 ASP 334 A <--> 17652 NH2 ARG 1294 B 2.86
269. 2513 OD2 ASP 334 A <--> 17452 CD1 LEU 1267 B 3.09
270. 2513 OD2 ASP 334 A <--> 17646 CB ARG 1294 B 3.84
271. 2513 OD2 ASP 334 A <--> 17647 CG ARG 1294 B 3.67
272. 2513 OD2 ASP 334 A <--> 17648 CD ARG 1294 B 2.86
273. 2513 OD2 ASP 334 A <--> 17649 NE ARG 1294 B 1.42
274. 2513 OD2 ASP 334 A <--> 17650 CZ ARG 1294 B 1.82
275. 2513 OD2 ASP 334 A <--> 17651 NH1 ARG 1294 B 3.18
276. 2513 OD2 ASP 334 A <--> 17652 NH2 ARG 1294 B 1.86

277.	2514	N	SER	335	A	<-->	17650	CZ	ARG	1294	B	3.83
278.	2514	N	SER	335	A	<-->	17652	NH2	ARG	1294	B	3.25
279.	2517	O	SER	335	A	<-->	17652	NH2	ARG	1294	B	3.62
280.	2519	OG	SER	335	A	<-->	15111	CB	ALA	960	B	3.52
281.	2527	ND2	ASN	336	A	<-->	15108	CA	ALA	960	B	3.86
282.	2798	CB	ALA	372	A	<-->	17705	CG2	THR	1302	B	3.50
283.	2806	OE1	GLU	373	A	<-->	17702	O	THR	1302	B	3.20
284.	3062	CB	LEU	406	A	<-->	17648	CD	ARG	1294	B	3.79
285.	3067	CA	GLU	407	A	<-->	17638	CB	LEU	1293	B	3.28
286.	3067	CA	GLU	407	A	<-->	17639	CG	LEU	1293	B	3.42
287.	3067	CA	GLU	407	A	<-->	17640	CD1	LEU	1293	B	3.06
288.	3067	CA	GLU	407	A	<-->	17641	CD2	LEU	1293	B	3.62
289.	3068	C	GLU	407	A	<-->	17635	CA	LEU	1293	B	3.05
290.	3068	C	GLU	407	A	<-->	17636	C	LEU	1293	B	3.62
291.	3068	C	GLU	407	A	<-->	17638	CB	LEU	1293	B	2.46
292.	3068	C	GLU	407	A	<-->	17639	CG	LEU	1293	B	2.81
293.	3068	C	GLU	407	A	<-->	17640	CD1	LEU	1293	B	3.16
294.	3068	C	GLU	407	A	<-->	17641	CD2	LEU	1293	B	2.73
295.	3068	C	GLU	407	A	<-->	17642	N	ARG	1294	B	3.61
296.	3069	O	GLU	407	A	<-->	17634	N	LEU	1293	B	3.31
297.	3069	O	GLU	407	A	<-->	17635	CA	LEU	1293	B	1.93
298.	3069	O	GLU	407	A	<-->	17636	C	LEU	1293	B	2.46
299.	3069	O	GLU	407	A	<-->	17637	O	LEU	1293	B	3.39
300.	3069	O	GLU	407	A	<-->	17638	CB	LEU	1293	B	1.46
301.	3069	O	GLU	407	A	<-->	17639	CG	LEU	1293	B	2.39
302.	3069	O	GLU	407	A	<-->	17640	CD1	LEU	1293	B	3.14
303.	3069	O	GLU	407	A	<-->	17641	CD2	LEU	1293	B	2.70
304.	3069	O	GLU	407	A	<-->	17642	N	ARG	1294	B	2.75
305.	3069	O	GLU	407	A	<-->	17647	CG	ARG	1294	B	3.89
306.	3070	CB	GLU	407	A	<-->	17638	CB	LEU	1293	B	3.75
307.	3070	CB	GLU	407	A	<-->	17639	CG	LEU	1293	B	3.22
308.	3070	CB	GLU	407	A	<-->	17640	CD1	LEU	1293	B	2.40
309.	3070	CB	GLU	407	A	<-->	17641	CD2	LEU	1293	B	3.32
310.	3071	CG	GLU	407	A	<-->	17640	CD1	LEU	1293	B	3.38
311.	3075	N	ILE	408	A	<-->	17635	CA	LEU	1293	B	3.73
312.	3075	N	ILE	408	A	<-->	17638	CB	LEU	1293	B	3.52
313.	3075	N	ILE	408	A	<-->	17639	CG	LEU	1293	B	3.60
314.	3075	N	ILE	408	A	<-->	17641	CD2	LEU	1293	B	2.90
315.	3076	CA	ILE	408	A	<-->	17635	CA	LEU	1293	B	3.87
316.	3076	CA	ILE	408	A	<-->	17641	CD2	LEU	1293	B	3.34
317.	3079	CB	ILE	408	A	<-->	17631	O	SER	1292	B	3.57
318.	3079	CB	ILE	408	A	<-->	17641	CD2	LEU	1293	B	2.95
319.	3080	CG1	ILE	408	A	<-->	17619	O	VAL	1290	B	3.73
320.	3080	CG1	ILE	408	A	<-->	17630	C	SER	1292	B	3.22
321.	3080	CG1	ILE	408	A	<-->	17631	O	SER	1292	B	2.38
322.	3080	CG1	ILE	408	A	<-->	17634	N	LEU	1293	B	3.52
323.	3080	CG1	ILE	408	A	<-->	17635	CA	LEU	1293	B	3.11
324.	3080	CG1	ILE	408	A	<-->	17638	CB	LEU	1293	B	3.61
325.	3080	CG1	ILE	408	A	<-->	17639	CG	LEU	1293	B	3.24
326.	3080	CG1	ILE	408	A	<-->	17641	CD2	LEU	1293	B	1.93
327.	3081	CG2	ILE	408	A	<-->	17631	O	SER	1292	B	3.83
328.	3082	CD1	ILE	408	A	<-->	17618	C	VAL	1290	B	3.48
329.	3082	CD1	ILE	408	A	<-->	17619	O	VAL	1290	B	2.32
330.	3082	CD1	ILE	408	A	<-->	17625	C	ALA	1291	B	3.79
331.	3082	CD1	ILE	408	A	<-->	17626	O	ALA	1291	B	3.66
332.	3082	CD1	ILE	408	A	<-->	17630	C	SER	1292	B	3.53
333.	3082	CD1	ILE	408	A	<-->	17631	O	SER	1292	B	2.73
334.	3082	CD1	ILE	408	A	<-->	17639	CG	LEU	1293	B	3.85
335.	3082	CD1	ILE	408	A	<-->	17641	CD2	LEU	1293	B	2.52
336.	3193	OG1	THR	424	A	<-->	17621	CG1	VAL	1290	B	3.87

337. 3205 CA GLY 427 A <--> 17644 C ARG 1294 B 3.84
338. 3206 C GLY 427 A <--> 17657 CB GLN 1295 B 3.36
339. 3206 C GLY 427 A <--> 17660 OE1 GLN 1295 B 3.36
340. 3207 O GLY 427 A <--> 17660 OE1 GLN 1295 B 3.25
341. 3208 N ALA 428 A <--> 17644 C ARG 1294 B 3.64
342. 3208 N ALA 428 A <--> 17645 O ARG 1294 B 3.78
343. 3208 N ALA 428 A <--> 17653 N GLN 1295 B 3.27
344. 3208 N ALA 428 A <--> 17654 CA GLN 1295 B 2.98
345. 3208 N ALA 428 A <--> 17657 CB GLN 1295 B 2.18
346. 3208 N ALA 428 A <--> 17658 CG GLN 1295 B 2.97
347. 3208 N ALA 428 A <--> 17659 CD GLN 1295 B 3.21
348. 3208 N ALA 428 A <--> 17660 OE1 GLN 1295 B 2.70
349. 3209 CA ALA 428 A <--> 17653 N GLN 1295 B 3.90
350. 3209 CA ALA 428 A <--> 17654 CA GLN 1295 B 3.22
351. 3209 CA ALA 428 A <--> 17657 CB GLN 1295 B 1.86
352. 3209 CA ALA 428 A <--> 17658 CG GLN 1295 B 2.05
353. 3209 CA ALA 428 A <--> 17659 CD GLN 1295 B 1.83
354. 3209 CA ALA 428 A <--> 17660 OE1 GLN 1295 B 1.34
355. 3209 CA ALA 428 A <--> 17661 NE2 GLN 1295 B 3.16
356. 3210 C ALA 428 A <--> 17657 CB GLN 1295 B 3.29
357. 3210 C ALA 428 A <--> 17658 CG GLN 1295 B 3.01
358. 3210 C ALA 428 A <--> 17659 CD GLN 1295 B 2.27
359. 3210 C ALA 428 A <--> 17660 OE1 GLN 1295 B 1.26
360. 3210 C ALA 428 A <--> 17661 NE2 GLN 1295 B 3.40
361. 3210 C ALA 428 A <--> 17716 CB PHE 1304 B 3.80
362. 3210 C ALA 428 A <--> 17718 CD1 PHE 1304 B 3.88
363. 3211 O ALA 428 A <--> 17658 CG GLN 1295 B 3.75
364. 3211 O ALA 428 A <--> 17659 CD GLN 1295 B 2.55
365. 3211 O ALA 428 A <--> 17660 OE1 GLN 1295 B 1.43
366. 3211 O ALA 428 A <--> 17661 NE2 GLN 1295 B 3.28
367. 3211 O ALA 428 A <--> 17718 CD1 PHE 1304 B 3.78
368. 3212 CB ALA 428 A <--> 17653 N GLN 1295 B 3.85
369. 3212 CB ALA 428 A <--> 17654 CA GLN 1295 B 2.69
370. 3212 CB ALA 428 A <--> 17655 C GLN 1295 B 3.34
371. 3212 CB ALA 428 A <--> 17657 CB GLN 1295 B 1.53
372. 3212 CB ALA 428 A <--> 17658 CG GLN 1295 B 0.63
373. 3212 CB ALA 428 A <--> 17659 CD GLN 1295 B 1.44
374. 3212 CB ALA 428 A <--> 17660 OE1 GLN 1295 B 2.01
375. 3212 CB ALA 428 A <--> 17661 NE2 GLN 1295 B 2.63
376. 3212 CB ALA 428 A <--> 17662 N ALA 1296 B 3.75
377. 3212 CB ALA 428 A <--> 17716 CB PHE 1304 B 2.96
378. 3213 N GLY 429 A <--> 17658 CG GLN 1295 B 3.64
379. 3213 N GLY 429 A <--> 17659 CD GLN 1295 B 3.29
380. 3213 N GLY 429 A <--> 17660 OE1 GLN 1295 B 2.52
381. 3213 N GLY 429 A <--> 17716 CB PHE 1304 B 3.86
382. 3213 N GLY 429 A <--> 17717 CG PHE 1304 B 3.70
383. 3213 N GLY 429 A <--> 17718 CD1 PHE 1304 B 3.71
384. 3214 CA GLY 429 A <--> 17660 OE1 GLN 1295 B 3.51
385. 3214 CA GLY 429 A <--> 17717 CG PHE 1304 B 3.77
386. 3214 CA GLY 429 A <--> 17718 CD1 PHE 1304 B 3.40
387. 3214 CA GLY 429 A <--> 17720 CE1 PHE 1304 B 3.52
388. 3215 C GLY 429 A <--> 17717 CG PHE 1304 B 3.81
389. 3215 C GLY 429 A <--> 17718 CD1 PHE 1304 B 3.74
390. 3215 C GLY 429 A <--> 17719 CD2 PHE 1304 B 3.71
391. 3215 C GLY 429 A <--> 17720 CE1 PHE 1304 B 3.58
392. 3215 C GLY 429 A <--> 17721 CE2 PHE 1304 B 3.54
393. 3215 C GLY 429 A <--> 17722 CZ PHE 1304 B 3.48
394. 3216 O GLY 429 A <--> 17717 CG PHE 1304 B 3.07
395. 3216 O GLY 429 A <--> 17718 CD1 PHE 1304 B 3.28
396. 3216 O GLY 429 A <--> 17719 CD2 PHE 1304 B 2.63

397.	3216	O	GLY	429	A	<-->	17720	CE1	PHE	1304	B	3.13
398.	3216	O	GLY	429	A	<-->	17721	CE2	PHE	1304	B	2.44
399.	3216	O	GLY	429	A	<-->	17722	CZ	PHE	1304	B	2.72
400.	3218	CA	GLU	430	A	<-->	17613	CG	ASN	1289	B	3.75
401.	3218	CA	GLU	430	A	<-->	17614	OD1	ASN	1289	B	3.65
402.	3218	CA	GLU	430	A	<-->	17615	ND2	ASN	1289	B	3.49
403.	3221	CB	GLU	430	A	<-->	17612	CB	ASN	1289	B	3.59
404.	3221	CB	GLU	430	A	<-->	17613	CG	ASN	1289	B	2.33
405.	3221	CB	GLU	430	A	<-->	17614	OD1	ASN	1289	B	2.43
406.	3221	CB	GLU	430	A	<-->	17615	ND2	ASN	1289	B	1.99
407.	3222	CG	GLU	430	A	<-->	17613	CG	ASN	1289	B	2.86
408.	3222	CG	GLU	430	A	<-->	17614	OD1	ASN	1289	B	3.24
409.	3222	CG	GLU	430	A	<-->	17615	ND2	ASN	1289	B	1.79
410.	3223	CD	GLU	430	A	<-->	17611	O	ASN	1289	B	3.69
411.	3223	CD	GLU	430	A	<-->	17612	CB	ASN	1289	B	3.53
412.	3223	CD	GLU	430	A	<-->	17613	CG	ASN	1289	B	2.32
413.	3223	CD	GLU	430	A	<-->	17614	OD1	ASN	1289	B	2.86
414.	3223	CD	GLU	430	A	<-->	17615	ND2	ASN	1289	B	1.23
415.	3224	OE1	GLU	430	A	<-->	17610	C	ASN	1289	B	3.53
416.	3224	OE1	GLU	430	A	<-->	17611	O	ASN	1289	B	2.91
417.	3224	OE1	GLU	430	A	<-->	17612	CB	ASN	1289	B	3.53
418.	3224	OE1	GLU	430	A	<-->	17613	CG	ASN	1289	B	2.20
419.	3224	OE1	GLU	430	A	<-->	17614	OD1	ASN	1289	B	2.27
420.	3224	OE1	GLU	430	A	<-->	17615	ND2	ASN	1289	B	1.86
421.	3225	OE2	GLU	430	A	<-->	17611	O	ASN	1289	B	3.83
422.	3225	OE2	GLU	430	A	<-->	17612	CB	ASN	1289	B	3.72
423.	3225	OE2	GLU	430	A	<-->	17613	CG	ASN	1289	B	2.95
424.	3225	OE2	GLU	430	A	<-->	17614	OD1	ASN	1289	B	3.79
425.	3225	OE2	GLU	430	A	<-->	17615	ND2	ASN	1289	B	1.79
426.	3244	CG	ARG	433	A	<-->	18066	OG	SER	1349	B	2.95
427.	3245	CD	ARG	433	A	<-->	18065	CB	SER	1349	B	3.20
428.	3245	CD	ARG	433	A	<-->	18066	OG	SER	1349	B	2.19
429.	3246	NE	ARG	433	A	<-->	18066	OG	SER	1349	B	3.32
430.	3247	CZ	ARG	433	A	<-->	18066	OG	SER	1349	B	3.65
431.	3247	CZ	ARG	433	A	<-->	18081	NE2	GLN	1351	B	3.36
432.	3248	NH1	ARG	433	A	<-->	18066	OG	SER	1349	B	3.10
433.	3248	NH1	ARG	433	A	<-->	18078	CG	GLN	1351	B	3.29
434.	3248	NH1	ARG	433	A	<-->	18079	CD	GLN	1351	B	3.04
435.	3248	NH1	ARG	433	A	<-->	18081	NE2	GLN	1351	B	2.02
436.	3253	O	PRO	434	A	<-->	17622	CG2	VAL	1290	B	2.90
437.	3254	CB	PRO	434	A	<-->	17612	CB	ASN	1289	B	2.97
438.	3254	CB	PRO	434	A	<-->	17613	CG	ASN	1289	B	3.66
439.	3254	CB	PRO	434	A	<-->	17615	ND2	ASN	1289	B	3.47
440.	3255	CG	PRO	434	A	<-->	17592	O	GLY	1286	B	3.78
441.	3386	CB	SER	451	A	<-->	18057	O	PRO	1348	B	2.94
442.	3386	CB	SER	451	A	<-->	18062	CA	SER	1349	B	3.84
443.	3387	OG	SER	451	A	<-->	18056	C	PRO	1348	B	3.69
444.	3387	OG	SER	451	A	<-->	18057	O	PRO	1348	B	2.82
445.	3387	OG	SER	451	A	<-->	18062	CA	SER	1349	B	3.19
446.	3387	OG	SER	451	A	<-->	18065	CB	SER	1349	B	3.43
447.	3387	OG	SER	451	A	<-->	18066	OG	SER	1349	B	3.47
448.	3393	CG	GLU	452	A	<-->	18085	O	ALA	1352	B	3.83
449.	3394	CD	GLU	452	A	<-->	18082	N	ALA	1352	B	2.98
450.	3394	CD	GLU	452	A	<-->	18083	CA	ALA	1352	B	3.49
451.	3394	CD	GLU	452	A	<-->	18084	C	ALA	1352	B	3.24
452.	3394	CD	GLU	452	A	<-->	18085	O	ALA	1352	B	2.33
453.	3394	CD	GLU	452	A	<-->	18086	CB	ALA	1352	B	3.61
454.	3395	OE1	GLU	452	A	<-->	18074	CA	GLN	1351	B	3.23
455.	3395	OE1	GLU	452	A	<-->	18075	C	GLN	1351	B	3.00
456.	3395	OE1	GLU	452	A	<-->	18077	CB	GLN	1351	B	3.54

457. 3395 OE1 GLU 452 A <--> 18082 N ALA 1352 B 2.03
458. 3395 OE1 GLU 452 A <--> 18083 CA ALA 1352 B 2.80
459. 3395 OE1 GLU 452 A <--> 18084 C ALA 1352 B 2.61
460. 3395 OE1 GLU 452 A <--> 18085 O ALA 1352 B 1.96
461. 3395 OE1 GLU 452 A <--> 18086 CB ALA 1352 B 3.44
462. 3395 OE1 GLU 452 A <--> 18087 N GLY 1353 B 3.84
463. 3396 OE2 GLU 452 A <--> 18082 N ALA 1352 B 3.60
464. 3396 OE2 GLU 452 A <--> 18083 CA ALA 1352 B 3.62
465. 3396 OE2 GLU 452 A <--> 18084 C ALA 1352 B 3.15
466. 3396 OE2 GLU 452 A <--> 18085 O ALA 1352 B 2.17
467. 3396 OE2 GLU 452 A <--> 18086 CB ALA 1352 B 3.39
468. 3662 O VAL 487 A <--> 13664 OE2 GLU 772 B 3.79
469. 3666 N CYS 488 A <--> 13664 OE2 GLU 772 B 3.85
470. 3667 CA CYS 488 A <--> 13662 CD GLU 772 B 3.36
471. 3667 CA CYS 488 A <--> 13663 OE1 GLU 772 B 3.69
472. 3667 CA CYS 488 A <--> 13664 OE2 GLU 772 B 2.70
473. 3668 C CYS 488 A <--> 13661 CG GLU 772 B 3.07
474. 3668 C CYS 488 A <--> 13662 CD GLU 772 B 2.20
475. 3668 C CYS 488 A <--> 13663 OE1 GLU 772 B 2.94
476. 3668 C CYS 488 A <--> 13664 OE2 GLU 772 B 1.55
477. 3669 O CYS 488 A <--> 13308 NE ARG 726 B 3.61
478. 3669 O CYS 488 A <--> 13309 CZ ARG 726 B 3.46
479. 3669 O CYS 488 A <--> 13310 NH1 ARG 726 B 3.80
480. 3669 O CYS 488 A <--> 13311 NH2 ARG 726 B 3.75
481. 3669 O CYS 488 A <--> 13661 CG GLU 772 B 2.68
482. 3669 O CYS 488 A <--> 13662 CD GLU 772 B 1.65
483. 3669 O CYS 488 A <--> 13663 OE1 GLU 772 B 2.59
484. 3669 O CYS 488 A <--> 13664 OE2 GLU 772 B 0.47
485. 3670 CB CYS 488 A <--> 13662 CD GLU 772 B 3.56
486. 3670 CB CYS 488 A <--> 13663 OE1 GLU 772 B 3.38
487. 3670 CB CYS 488 A <--> 13664 OE2 GLU 772 B 3.39
488. 3672 N ILE 489 A <--> 13661 CG GLU 772 B 2.83
489. 3672 N ILE 489 A <--> 13662 CD GLU 772 B 2.62
490. 3672 N ILE 489 A <--> 13663 OE1 GLU 772 B 3.42
491. 3672 N ILE 489 A <--> 13664 OE2 GLU 772 B 2.46
492. 3672 N ILE 489 A <--> 13665 N LEU 773 B 3.75
493. 3672 N ILE 489 A <--> 13670 CG LEU 773 B 3.80
494. 3672 N ILE 489 A <--> 13672 CD2 LEU 773 B 3.13
495. 3673 CA ILE 489 A <--> 13294 CD2 LEU 724 B 3.85
496. 3673 CA ILE 489 A <--> 13311 NH2 ARG 726 B 3.79
497. 3673 CA ILE 489 A <--> 13660 CB GLU 772 B 3.42
498. 3673 CA ILE 489 A <--> 13661 CG GLU 772 B 2.02
499. 3673 CA ILE 489 A <--> 13662 CD GLU 772 B 2.60
500. 3673 CA ILE 489 A <--> 13663 OE1 GLU 772 B 3.60
501. 3673 CA ILE 489 A <--> 13664 OE2 GLU 772 B 2.76
502. 3673 CA ILE 489 A <--> 13665 N LEU 773 B 3.21
503. 3673 CA ILE 489 A <--> 13666 CA LEU 773 B 3.84
504. 3673 CA ILE 489 A <--> 13669 CB LEU 773 B 3.83
505. 3673 CA ILE 489 A <--> 13670 CG LEU 773 B 2.78
506. 3673 CA ILE 489 A <--> 13671 CD1 LEU 773 B 3.80
507. 3673 CA ILE 489 A <--> 13672 CD2 LEU 773 B 2.68
508. 3674 C ILE 489 A <--> 13309 CZ ARG 726 B 3.63
509. 3674 C ILE 489 A <--> 13311 NH2 ARG 726 B 2.76
510. 3674 C ILE 489 A <--> 13661 CG GLU 772 B 2.73
511. 3674 C ILE 489 A <--> 13662 CD GLU 772 B 3.35
512. 3674 C ILE 489 A <--> 13664 OE2 GLU 772 B 3.11
513. 3674 C ILE 489 A <--> 13670 CG LEU 773 B 3.39
514. 3674 C ILE 489 A <--> 13671 CD1 LEU 773 B 3.83
515. 3674 C ILE 489 A <--> 13672 CD2 LEU 773 B 3.44
516. 3675 O ILE 489 A <--> 13294 CD2 LEU 724 B 3.69

517.	3675	O	ILE	489	A	<-->	13308	NE	ARG	726	B	3.46
518.	3675	O	ILE	489	A	<-->	13309	CZ	ARG	726	B	2.83
519.	3675	O	ILE	489	A	<-->	13310	NH1	ARG	726	B	3.83
520.	3675	O	ILE	489	A	<-->	13311	NH2	ARG	726	B	1.74
521.	3675	O	ILE	489	A	<-->	13660	CB	GLU	772	B	3.84
522.	3675	O	ILE	489	A	<-->	13661	CG	GLU	772	B	2.73
523.	3675	O	ILE	489	A	<-->	13662	CD	GLU	772	B	3.60
524.	3675	O	ILE	489	A	<-->	13664	OE2	GLU	772	B	3.46
525.	3675	O	ILE	489	A	<-->	13670	CG	LEU	773	B	3.87
526.	3676	CB	ILE	489	A	<-->	13294	CD2	LEU	724	B	3.28
527.	3676	CB	ILE	489	A	<-->	13658	C	GLU	772	B	3.52
528.	3676	CB	ILE	489	A	<-->	13660	CB	GLU	772	B	3.87
529.	3676	CB	ILE	489	A	<-->	13661	CG	GLU	772	B	2.88
530.	3676	CB	ILE	489	A	<-->	13662	CD	GLU	772	B	3.80
531.	3676	CB	ILE	489	A	<-->	13665	N	LEU	773	B	2.63
532.	3676	CB	ILE	489	A	<-->	13666	CA	LEU	773	B	2.73
533.	3676	CB	ILE	489	A	<-->	13669	CB	LEU	773	B	2.43
534.	3676	CB	ILE	489	A	<-->	13670	CG	LEU	773	B	1.33
535.	3676	CB	ILE	489	A	<-->	13671	CD1	LEU	773	B	2.61
536.	3676	CB	ILE	489	A	<-->	13672	CD2	LEU	773	B	1.48
537.	3677	CG1	ILE	489	A	<-->	13665	N	LEU	773	B	3.82
538.	3677	CG1	ILE	489	A	<-->	13666	CA	LEU	773	B	3.45
539.	3677	CG1	ILE	489	A	<-->	13669	CB	LEU	773	B	2.96
540.	3677	CG1	ILE	489	A	<-->	13670	CG	LEU	773	B	1.72
541.	3677	CG1	ILE	489	A	<-->	13671	CD1	LEU	773	B	2.50
542.	3677	CG1	ILE	489	A	<-->	13672	CD2	LEU	773	B	0.57
543.	3678	CG2	ILE	489	A	<-->	13294	CD2	LEU	724	B	3.52
544.	3678	CG2	ILE	489	A	<-->	13657	CA	GLU	772	B	3.38
545.	3678	CG2	ILE	489	A	<-->	13658	C	GLU	772	B	2.45
546.	3678	CG2	ILE	489	A	<-->	13659	O	GLU	772	B	3.26
547.	3678	CG2	ILE	489	A	<-->	13660	CB	GLU	772	B	3.61
548.	3678	CG2	ILE	489	A	<-->	13661	CG	GLU	772	B	3.06
549.	3678	CG2	ILE	489	A	<-->	13662	CD	GLU	772	B	3.84
550.	3678	CG2	ILE	489	A	<-->	13665	N	LEU	773	B	1.25
551.	3678	CG2	ILE	489	A	<-->	13666	CA	LEU	773	B	1.41
552.	3678	CG2	ILE	489	A	<-->	13667	C	LEU	773	B	2.83
553.	3678	CG2	ILE	489	A	<-->	13668	O	LEU	773	B	3.66
554.	3678	CG2	ILE	489	A	<-->	13669	CB	LEU	773	B	1.99
555.	3678	CG2	ILE	489	A	<-->	13670	CG	LEU	773	B	2.02
556.	3678	CG2	ILE	489	A	<-->	13671	CD1	LEU	773	B	3.55
557.	3678	CG2	ILE	489	A	<-->	13672	CD2	LEU	773	B	2.18
558.	3678	CG2	ILE	489	A	<-->	13673	N	SER	774	B	3.55
559.	3679	CD1	ILE	489	A	<-->	13666	CA	LEU	773	B	3.78
560.	3679	CD1	ILE	489	A	<-->	13669	CB	LEU	773	B	3.83
561.	3679	CD1	ILE	489	A	<-->	13670	CG	LEU	773	B	3.00
562.	3679	CD1	ILE	489	A	<-->	13672	CD2	LEU	773	B	1.48
563.	3680	N	HIS	490	A	<-->	13311	NH2	ARG	726	B	3.52
564.	3680	N	HIS	490	A	<-->	13664	OE2	GLU	772	B	3.79
565.	3680	N	HIS	490	A	<-->	13672	CD2	LEU	773	B	3.67
566.	3681	CA	HIS	490	A	<-->	13311	NH2	ARG	726	B	3.63
567.	3682	C	HIS	490	A	<-->	13309	CZ	ARG	726	B	3.81
568.	3682	C	HIS	490	A	<-->	13310	NH1	ARG	726	B	3.76
569.	3682	C	HIS	490	A	<-->	13311	NH2	ARG	726	B	3.07
570.	3683	O	HIS	490	A	<-->	13310	NH1	ARG	726	B	3.78
571.	3683	O	HIS	490	A	<-->	13311	NH2	ARG	726	B	3.24
572.	3690	N	ARG	491	A	<-->	13309	CZ	ARG	726	B	3.53
573.	3690	N	ARG	491	A	<-->	13310	NH1	ARG	726	B	3.26
574.	3690	N	ARG	491	A	<-->	13311	NH2	ARG	726	B	3.24
575.	3690	N	ARG	491	A	<-->	13664	OE2	GLU	772	B	3.61
576.	3691	CA	ARG	491	A	<-->	13309	CZ	ARG	726	B	3.47

577. 3691 CA ARG 491 A <--> 13310 NH1 ARG 726 B 2.68
578. 3691 CA ARG 491 A <--> 13311 NH2 ARG 726 B 3.68
579. 3691 CA ARG 491 A <--> 13664 OE2 GLU 772 B 3.86
580. 3692 C ARG 491 A <--> 13310 NH1 ARG 726 B 3.73
581. 3693 O ARG 491 A <--> 13310 NH1 ARG 726 B 3.87
582. 3694 CB ARG 491 A <--> 13308 NE ARG 726 B 3.56
583. 3694 CB ARG 491 A <--> 13309 CZ ARG 726 B 2.47
584. 3694 CB ARG 491 A <--> 13310 NH1 ARG 726 B 1.30
585. 3694 CB ARG 491 A <--> 13311 NH2 ARG 726 B 3.08
586. 3695 CG ARG 491 A <--> 13308 NE ARG 726 B 3.85
587. 3695 CG ARG 491 A <--> 13309 CZ ARG 726 B 2.61
588. 3695 CG ARG 491 A <--> 13310 NH1 ARG 726 B 1.72
589. 3695 CG ARG 491 A <--> 13311 NH2 ARG 726 B 2.87
590. 3696 CD ARG 491 A <--> 13308 NE ARG 726 B 3.86
591. 3696 CD ARG 491 A <--> 13309 CZ ARG 726 B 2.98
592. 3696 CD ARG 491 A <--> 13310 NH1 ARG 726 B 2.16
593. 3696 CD ARG 491 A <--> 13311 NH2 ARG 726 B 3.56
594. 3697 NE ARG 491 A <--> 13310 NH1 ARG 726 B 3.46
595. 3699 NH1 ARG 491 A <--> 13308 NE ARG 726 B 3.67
596. 3699 NH1 ARG 491 A <--> 13309 CZ ARG 726 B 3.23
597. 3699 NH1 ARG 491 A <--> 13310 NH1 ARG 726 B 3.62
598. 3699 NH1 ARG 491 A <--> 13311 NH2 ARG 726 B 3.21
599. 4126 CB LEU 544 A <--> 17412 CD LYS 1262 B 3.51
600. 4127 CG LEU 544 A <--> 17411 CG LYS 1262 B 3.27
601. 4127 CG LEU 544 A <--> 17412 CD LYS 1262 B 2.15
602. 4127 CG LEU 544 A <--> 17413 CE LYS 1262 B 2.66
603. 4127 CG LEU 544 A <--> 17414 NZ LYS 1262 B 2.67
604. 4128 CD1 LEU 544 A <--> 17411 CG LYS 1262 B 3.25
605. 4128 CD1 LEU 544 A <--> 17412 CD LYS 1262 B 1.76
606. 4128 CD1 LEU 544 A <--> 17413 CE LYS 1262 B 2.18
607. 4128 CD1 LEU 544 A <--> 17414 NZ LYS 1262 B 1.86
608. 4129 CD2 LEU 544 A <--> 17412 CD LYS 1262 B 2.92
609. 4129 CD2 LEU 544 A <--> 17413 CE LYS 1262 B 2.63
610. 4129 CD2 LEU 544 A <--> 17414 NZ LYS 1262 B 2.55
611. 4369 C PRO 576 A <--> 11136 CG2 ILE 450 B 3.60
612. 4370 O PRO 576 A <--> 11134 CB ILE 450 B 3.66
613. 4370 O PRO 576 A <--> 11135 CG1 ILE 450 B 3.46
614. 4370 O PRO 576 A <--> 11136 CG2 ILE 450 B 2.93
615. 4370 O PRO 576 A <--> 11137 CD1 ILE 450 B 3.71
616. 4371 CB PRO 576 A <--> 11136 CG2 ILE 450 B 3.70
617. 4372 CG PRO 576 A <--> 13957 OG SER 809 B 3.31
618. 4375 CA ASP 577 A <--> 11135 CG1 ILE 450 B 3.43
619. 4376 C ASP 577 A <--> 11135 CG1 ILE 450 B 3.75
620. 4376 C ASP 577 A <--> 11137 CD1 ILE 450 B 3.87
621. 4382 N THR 578 A <--> 11135 CG1 ILE 450 B 3.09
622. 4382 N THR 578 A <--> 11137 CD1 ILE 450 B 2.78
623. 4383 CA THR 578 A <--> 11137 CD1 ILE 450 B 3.18
624. 4386 CB THR 578 A <--> 11135 CG1 ILE 450 B 3.83
625. 4386 CB THR 578 A <--> 11137 CD1 ILE 450 B 2.54
626. 4387 OG1 THR 578 A <--> 11084 CD1 TYR 444 B 3.76
627. 4387 OG1 THR 578 A <--> 11086 CE1 TYR 444 B 3.66
628. 4387 OG1 THR 578 A <--> 11135 CG1 ILE 450 B 3.63
629. 4387 OG1 THR 578 A <--> 11137 CD1 ILE 450 B 2.48
630. 4387 OG1 THR 578 A <--> 11141 O GLU 451 B 3.43
631. 4388 CG2 THR 578 A <--> 11133 O ILE 450 B 3.68
632. 4388 CG2 THR 578 A <--> 11135 CG1 ILE 450 B 3.22
633. 4388 CG2 THR 578 A <--> 11137 CD1 ILE 450 B 1.82
634. 4393 CB SER 579 A <--> 11084 CD1 TYR 444 B 3.62
635. 4526 CB PRO 597 A <--> 11069 CB ALA 442 B 3.62
636. 4527 CG PRO 597 A <--> 11068 O ALA 442 B 3.42

637.	4527	CG	PRO	597	A	<-->	11069	CB	ALA	442	B	3.81
638.	4539	CB	PRO	599	A	<-->	11152	OG1	THR	452	B	3.20
639.	4540	CG	PRO	599	A	<-->	11152	OG1	THR	452	B	2.91
640.	4549	OE1	GLN	600	A	<-->	12011	CB	ARG	561	B	3.64
641.	4550	NE2	GLN	600	A	<-->	11065	N	ALA	442	B	3.74
642.	4551	N	VAL	601	A	<-->	12006	CD2	LEU	560	B	3.79
643.	4552	CA	VAL	601	A	<-->	12006	CD2	LEU	560	B	3.79
644.	4555	CB	VAL	601	A	<-->	12004	CG	LEU	560	B	2.84
645.	4555	CB	VAL	601	A	<-->	12005	CD1	LEU	560	B	3.03
646.	4555	CB	VAL	601	A	<-->	12006	CD2	LEU	560	B	3.10
647.	4556	CG1	VAL	601	A	<-->	12004	CG	LEU	560	B	3.63
648.	4557	CG2	VAL	601	A	<-->	12000	CA	LEU	560	B	3.73
649.	4557	CG2	VAL	601	A	<-->	12001	C	LEU	560	B	3.79
650.	4557	CG2	VAL	601	A	<-->	12003	CB	LEU	560	B	3.07
651.	4557	CG2	VAL	601	A	<-->	12004	CG	LEU	560	B	1.64
652.	4557	CG2	VAL	601	A	<-->	12005	CD1	LEU	560	B	2.42
653.	4557	CG2	VAL	601	A	<-->	12006	CD2	LEU	560	B	1.61
654.	4557	CG2	VAL	601	A	<-->	12007	N	ARG	561	B	3.32
655.	4563	CG	PRO	602	A	<-->	12006	CD2	LEU	560	B	3.78
656.	4563	CG	PRO	602	A	<-->	12016	NH1	ARG	561	B	3.45
657.	4564	CD	PRO	602	A	<-->	12004	CG	LEU	560	B	3.73
658.	4564	CD	PRO	602	A	<-->	12005	CD1	LEU	560	B	3.53
659.	4564	CD	PRO	602	A	<-->	12006	CD2	LEU	560	B	2.82
660.	4597	NH1	ARG	606	A	<-->	11068	O	ALA	442	B	3.46
661.	4597	NH1	ARG	606	A	<-->	11071	CA	ASP	443	B	3.88

Salt bridges

	<----- A T O M 1 ----->						<----- A T O M 2 ----->					
	Atom no.	Atom name	Res name	Res no.	Chain		Atom no.	Atom name	Res name	Res no.	Chain	Distance
1.	2513	OD2	ASP	334	A	<-->	17649	NE	ARG	1294	B	1.42

Number of salt bridges: 1

Number of hydrogen bonds: 6

Number of non-bonded contacts: 661

[F] ROBO4 (WT) + P665S _FULL PROTEIN

Hydrogen bonds

<----- A T O M 1 -----> <----- A T O M 2 ----->

	Atom no.	Atom name	Res name	Res no.	Chain		Atom no.	Atom name	Res name	Res no.	Chain	Distance
1.	388	O	ALA	55	A	<-->	13499	NH1	ARG	754	B	3.05
2.	435	OG1	THR	62	A	<-->	14856	ND2	ASN	929	B	3.14
3.	953	OG	SER	131	A	<-->	13167	OD2	ASP	709	B	3.11
4.	953	OG	SER	131	A	<-->	13439	NZ	LYS	746	B	3.02
5.	1356	O	PRO	182	A	<-->	16397	ND2	ASN	1132	B	2.98
6.	1408	O	GLY	189	A	<-->	16334	N	CYS	1125	B	1.44
7.	1408	O	GLY	189	A	<-->	16339	SG	CYS	1125	B	3.16
8.	1604	NH2	ARG	216	A	<-->	16197	OH	TYR	1106	B	3.13
9.	1634	O	ALA	220	A	<-->	16355	ND2	ASN	1127	B	3.28
10.	1641	N	ARG	222	A	<-->	16373	OD1	ASP	1129	B	2.28
11.	1838	OE2	GLU	245	A	<-->	15700	SG	CYS	1040	B	2.24
12.	1857	O	THR	248	A	<-->	15544	OH	TYR	1020	B	2.71
13.	1872	O	LEU	250	A	<-->	15532	ND2	ASN	1019	B	1.26
14.	1920	OE2	GLU	256	A	<-->	19187	NH1	ARG	1500	B	2.81
15.	2212	N	TRP	297	A	<-->	16396	OD1	ASN	1132	B	3.19
16.	2856	NH2	ARG	380	A	<-->	19393	O	CYS	1527	B	3.24
17.	7260	O	GLN	970	A	<-->	15550	OG1	THR	1021	B	2.23
18.	7265	NE2	GLN	970	A	<-->	15029	O	GLN	952	B	1.95
19.	7405	NE2	GLN	988	A	<-->	17364	OD2	ASP	1257	B	3.05
20.	7413	NE	ARG	989	A	<-->	14872	O	THR	932	B	2.94
21.	7413	NE	ARG	989	A	<-->	14888	OD1	ASN	934	B	2.31
22.	7422	OG	SER	990	A	<-->	15302	OD2	ASP	988	B	3.25

Non-bonded contacts

<----- A T O M 1 -----> <----- A T O M 2 ----->

	Atom no.	Atom name	Res name	Res no.	Chain		Atom no.	Atom name	Res name	Res no.	Chain	Distance
1.	373	O	CYS	53	A	<-->	15693	CE2	PHE	1039	B	3.31
2.	373	O	CYS	53	A	<-->	15694	CZ	PHE	1039	B	3.66
3.	379	O	GLN	54	A	<-->	13508	OD2	ASP	755	B	3.82
4.	382	CD	GLN	54	A	<-->	15689	CG	PHE	1039	B	3.28
5.	382	CD	GLN	54	A	<-->	15690	CD1	PHE	1039	B	3.67
6.	382	CD	GLN	54	A	<-->	15691	CD2	PHE	1039	B	3.07
7.	382	CD	GLN	54	A	<-->	15692	CE1	PHE	1039	B	3.85
8.	382	CD	GLN	54	A	<-->	15693	CE2	PHE	1039	B	3.29
9.	382	CD	GLN	54	A	<-->	15694	CZ	PHE	1039	B	3.68
10.	383	OE1	GLN	54	A	<-->	15689	CG	PHE	1039	B	3.77
11.	383	OE1	GLN	54	A	<-->	15690	CD1	PHE	1039	B	3.75
12.	383	OE1	GLN	54	A	<-->	15691	CD2	PHE	1039	B	3.89
13.	383	OE1	GLN	54	A	<-->	15692	CE1	PHE	1039	B	3.86
14.	384	NE2	GLN	54	A	<-->	15688	CB	PHE	1039	B	2.79
15.	384	NE2	GLN	54	A	<-->	15689	CG	PHE	1039	B	2.03
16.	384	NE2	GLN	54	A	<-->	15690	CD1	PHE	1039	B	2.75
17.	384	NE2	GLN	54	A	<-->	15691	CD2	PHE	1039	B	1.78
18.	384	NE2	GLN	54	A	<-->	15692	CE1	PHE	1039	B	3.17

19.	384	NE2	GLN	54	A	<-->	15693	CE2	PHE	1039	B	2.38
20.	384	NE2	GLN	54	A	<-->	15694	CZ	PHE	1039	B	3.02
21.	385	N	ALA	55	A	<-->	13506	CG	ASP	755	B	3.88
22.	385	N	ALA	55	A	<-->	13507	OD1	ASP	755	B	3.72
23.	385	N	ALA	55	A	<-->	13508	OD2	ASP	755	B	3.69
24.	386	CA	ALA	55	A	<-->	13505	CB	ASP	755	B	3.58
25.	386	CA	ALA	55	A	<-->	13506	CG	ASP	755	B	2.48
26.	386	CA	ALA	55	A	<-->	13507	OD1	ASP	755	B	2.55
27.	386	CA	ALA	55	A	<-->	13508	OD2	ASP	755	B	2.38
28.	386	CA	ALA	55	A	<-->	13512	O	VAL	756	B	3.75
29.	387	C	ALA	55	A	<-->	13499	NH1	ARG	754	B	3.63
30.	387	C	ALA	55	A	<-->	13505	CB	ASP	755	B	3.70
31.	387	C	ALA	55	A	<-->	13506	CG	ASP	755	B	2.25
32.	387	C	ALA	55	A	<-->	13507	OD1	ASP	755	B	2.30
33.	387	C	ALA	55	A	<-->	13508	OD2	ASP	755	B	1.76
34.	388	O	ALA	55	A	<-->	13499	NH1	ARG	754	B	3.05
35.	388	O	ALA	55	A	<-->	13505	CB	ASP	755	B	3.42
36.	388	O	ALA	55	A	<-->	13506	CG	ASP	755	B	2.12
37.	388	O	ALA	55	A	<-->	13507	OD1	ASP	755	B	2.69
38.	388	O	ALA	55	A	<-->	13508	OD2	ASP	755	B	1.06
39.	389	CB	ALA	55	A	<-->	13503	C	ASP	755	B	3.82
40.	389	CB	ALA	55	A	<-->	13505	CB	ASP	755	B	3.05
41.	389	CB	ALA	55	A	<-->	13506	CG	ASP	755	B	2.24
42.	389	CB	ALA	55	A	<-->	13507	OD1	ASP	755	B	1.85
43.	389	CB	ALA	55	A	<-->	13508	OD2	ASP	755	B	2.89
44.	389	CB	ALA	55	A	<-->	13509	N	VAL	756	B	3.43
45.	389	CB	ALA	55	A	<-->	13511	C	VAL	756	B	3.36
46.	389	CB	ALA	55	A	<-->	13512	O	VAL	756	B	2.29
47.	390	N	SER	56	A	<-->	13499	NH1	ARG	754	B	3.84
48.	390	N	SER	56	A	<-->	13500	NH2	ARG	754	B	3.75
49.	390	N	SER	56	A	<-->	13506	CG	ASP	755	B	3.19
50.	390	N	SER	56	A	<-->	13507	OD1	ASP	755	B	2.74
51.	390	N	SER	56	A	<-->	13508	OD2	ASP	755	B	2.92
52.	391	CA	SER	56	A	<-->	13498	CZ	ARG	754	B	3.54
53.	391	CA	SER	56	A	<-->	13499	NH1	ARG	754	B	3.42
54.	391	CA	SER	56	A	<-->	13500	NH2	ARG	754	B	3.45
55.	391	CA	SER	56	A	<-->	13507	OD1	ASP	755	B	3.48
56.	391	CA	SER	56	A	<-->	13508	OD2	ASP	755	B	3.54
57.	394	CB	SER	56	A	<-->	13498	CZ	ARG	754	B	3.77
58.	394	CB	SER	56	A	<-->	13500	NH2	ARG	754	B	3.38
59.	395	OG	SER	56	A	<-->	13498	CZ	ARG	754	B	3.24
60.	395	OG	SER	56	A	<-->	13500	NH2	ARG	754	B	2.42
61.	395	OG	SER	56	A	<-->	13507	OD1	ASP	755	B	3.66
62.	395	OG	SER	56	A	<-->	13521	OG1	THR	757	B	3.38
63.	395	OG	SER	56	A	<-->	14680	CD1	PHE	905	B	3.89
64.	395	OG	SER	56	A	<-->	14682	CE1	PHE	905	B	3.27
65.	395	OG	SER	56	A	<-->	14683	CE2	PHE	905	B	3.31
66.	395	OG	SER	56	A	<-->	14684	CZ	PHE	905	B	2.93
67.	427	CB	PRO	61	A	<-->	15688	CB	PHE	1039	B	3.59
68.	427	CB	PRO	61	A	<-->	15691	CD2	PHE	1039	B	3.88
69.	428	CG	PRO	61	A	<-->	15687	O	PHE	1039	B	3.36
70.	428	CG	PRO	61	A	<-->	15688	CB	PHE	1039	B	3.75
71.	434	CB	THR	62	A	<-->	13522	CG2	THR	757	B	3.65
72.	434	CB	THR	62	A	<-->	14856	ND2	ASN	929	B	3.59
73.	435	OG1	THR	62	A	<-->	14856	ND2	ASN	929	B	3.14
74.	436	CG2	THR	62	A	<-->	13520	CB	THR	757	B	3.58
75.	436	CG2	THR	62	A	<-->	13521	OG1	THR	757	B	3.49

76.	436	CG2	THR	62	A	<-->	13522	CG2	THR	757	B	2.46
77.	455	NH2	ARG	64	A	<-->	15551	CG2	THR	1021	B	3.89
78.	715	ND1	HIS	98	A	<-->	15531	OD1	ASN	1019	B	3.67
79.	716	CD2	HIS	98	A	<-->	15531	OD1	ASN	1019	B	3.50
80.	717	CE1	HIS	98	A	<-->	15526	CA	ASN	1019	B	3.28
81.	717	CE1	HIS	98	A	<-->	15527	C	ASN	1019	B	3.83
82.	717	CE1	HIS	98	A	<-->	15528	O	ASN	1019	B	3.48
83.	717	CE1	HIS	98	A	<-->	15529	CB	ASN	1019	B	3.53
84.	717	CE1	HIS	98	A	<-->	15530	CG	ASN	1019	B	3.49
85.	717	CE1	HIS	98	A	<-->	15531	OD1	ASN	1019	B	2.75
86.	718	NE2	HIS	98	A	<-->	15526	CA	ASN	1019	B	3.79
87.	718	NE2	HIS	98	A	<-->	15530	CG	ASN	1019	B	3.67
88.	718	NE2	HIS	98	A	<-->	15531	OD1	ASN	1019	B	2.60
89.	759	CB	ALA	104	A	<-->	16539	OH	TYR	1151	B	3.60
90.	897	CA	VAL	124	A	<-->	14420	CB	SER	871	B	3.51
91.	897	CA	VAL	124	A	<-->	14421	OG	SER	871	B	3.28
92.	898	C	VAL	124	A	<-->	14420	CB	SER	871	B	3.17
93.	898	C	VAL	124	A	<-->	14421	OG	SER	871	B	3.55
94.	900	CB	VAL	124	A	<-->	14421	OG	SER	871	B	3.45
95.	901	CG1	VAL	124	A	<-->	14420	CB	SER	871	B	3.14
96.	901	CG1	VAL	124	A	<-->	14421	OG	SER	871	B	2.65
97.	903	N	SER	125	A	<-->	14417	CA	SER	871	B	3.30
98.	903	N	SER	125	A	<-->	14418	C	SER	871	B	3.48
99.	903	N	SER	125	A	<-->	14420	CB	SER	871	B	2.05
100.	903	N	SER	125	A	<-->	14421	OG	SER	871	B	2.87
101.	903	N	SER	125	A	<-->	14422	N	ASP	872	B	3.01
102.	903	N	SER	125	A	<-->	14425	O	ASP	872	B	3.78
103.	904	CA	SER	125	A	<-->	14417	CA	SER	871	B	3.42
104.	904	CA	SER	125	A	<-->	14418	C	SER	871	B	2.97
105.	904	CA	SER	125	A	<-->	14420	CB	SER	871	B	2.72
106.	904	CA	SER	125	A	<-->	14422	N	ASP	872	B	2.11
107.	904	CA	SER	125	A	<-->	14423	CA	ASP	872	B	2.73
108.	904	CA	SER	125	A	<-->	14424	C	ASP	872	B	3.08
109.	904	CA	SER	125	A	<-->	14425	O	ASP	872	B	2.66
110.	904	CA	SER	125	A	<-->	14426	CB	ASP	872	B	2.79
111.	905	C	SER	125	A	<-->	14417	CA	SER	871	B	3.72
112.	905	C	SER	125	A	<-->	14418	C	SER	871	B	3.21
113.	905	C	SER	125	A	<-->	14420	CB	SER	871	B	3.39
114.	905	C	SER	125	A	<-->	14422	N	ASP	872	B	1.99
115.	905	C	SER	125	A	<-->	14423	CA	ASP	872	B	2.47
116.	905	C	SER	125	A	<-->	14424	C	ASP	872	B	3.46
117.	905	C	SER	125	A	<-->	14425	O	ASP	872	B	3.54
118.	905	C	SER	125	A	<-->	14426	CB	ASP	872	B	2.14
119.	905	C	SER	125	A	<-->	14427	CG	ASP	872	B	3.64
120.	906	O	SER	125	A	<-->	14417	CA	SER	871	B	3.83
121.	906	O	SER	125	A	<-->	14418	C	SER	871	B	3.72
122.	906	O	SER	125	A	<-->	14420	CB	SER	871	B	3.45
123.	906	O	SER	125	A	<-->	14422	N	ASP	872	B	2.72
124.	906	O	SER	125	A	<-->	14423	CA	ASP	872	B	3.46
125.	906	O	SER	125	A	<-->	14426	CB	ASP	872	B	3.17
126.	907	CB	SER	125	A	<-->	14416	N	SER	871	B	3.72
127.	907	CB	SER	125	A	<-->	14417	CA	SER	871	B	2.78
128.	907	CB	SER	125	A	<-->	14418	C	SER	871	B	1.88
129.	907	CB	SER	125	A	<-->	14419	O	SER	871	B	2.53
130.	907	CB	SER	125	A	<-->	14420	CB	SER	871	B	2.63
131.	907	CB	SER	125	A	<-->	14422	N	ASP	872	B	1.46
132.	907	CB	SER	125	A	<-->	14423	CA	ASP	872	B	2.04

133. 907 CB SER 125 A <--> 14424 C ASP 872 B 1.98
134. 907 CB SER 125 A <--> 14425 O ASP 872 B 1.68
135. 907 CB SER 125 A <--> 14426 CB ASP 872 B 2.89
136. 907 CB SER 125 A <--> 14430 N TRP 873 B 3.22
137. 908 OG SER 125 A <--> 14410 C LEU 870 B 3.39
138. 908 OG SER 125 A <--> 14411 O LEU 870 B 3.49
139. 908 OG SER 125 A <--> 14416 N SER 871 B 2.61
140. 908 OG SER 125 A <--> 14417 CA SER 871 B 1.56
141. 908 OG SER 125 A <--> 14418 C SER 871 B 0.56
142. 908 OG SER 125 A <--> 14419 O SER 871 B 1.68
143. 908 OG SER 125 A <--> 14420 CB SER 871 B 2.14
144. 908 OG SER 125 A <--> 14421 OG SER 871 B 3.49
145. 908 OG SER 125 A <--> 14422 N ASP 872 B 1.05
146. 908 OG SER 125 A <--> 14423 CA ASP 872 B 2.27
147. 908 OG SER 125 A <--> 14424 C ASP 872 B 2.70
148. 908 OG SER 125 A <--> 14425 O ASP 872 B 2.88
149. 908 OG SER 125 A <--> 14426 CB ASP 872 B 3.46
150. 908 OG SER 125 A <--> 14430 N TRP 873 B 3.67
151. 909 N ARG 126 A <--> 14418 C SER 871 B 3.62
152. 909 N ARG 126 A <--> 14422 N ASP 872 B 2.27
153. 909 N ARG 126 A <--> 14423 CA ASP 872 B 1.89
154. 909 N ARG 126 A <--> 14424 C ASP 872 B 3.02
155. 909 N ARG 126 A <--> 14425 O ASP 872 B 3.43
156. 909 N ARG 126 A <--> 14426 CB ASP 872 B 0.92
157. 909 N ARG 126 A <--> 14427 CG ASP 872 B 2.34
158. 909 N ARG 126 A <--> 14428 OD1 ASP 872 B 3.29
159. 909 N ARG 126 A <--> 14429 OD2 ASP 872 B 2.94
160. 910 CA ARG 126 A <--> 14422 N ASP 872 B 3.25
161. 910 CA ARG 126 A <--> 14423 CA ASP 872 B 2.76
162. 910 CA ARG 126 A <--> 14426 CB ASP 872 B 1.77
163. 910 CA ARG 126 A <--> 14427 CG ASP 872 B 2.41
164. 910 CA ARG 126 A <--> 14428 OD1 ASP 872 B 3.44
165. 910 CA ARG 126 A <--> 14429 OD2 ASP 872 B 2.61
166. 911 C ARG 126 A <--> 14422 N ASP 872 B 3.06
167. 911 C ARG 126 A <--> 14423 CA ASP 872 B 2.68
168. 911 C ARG 126 A <--> 14426 CB ASP 872 B 2.51
169. 911 C ARG 126 A <--> 14427 CG ASP 872 B 3.01
170. 911 C ARG 126 A <--> 14428 OD1 ASP 872 B 3.61
171. 911 C ARG 126 A <--> 14429 OD2 ASP 872 B 3.51
172. 912 O ARG 126 A <--> 14418 C SER 871 B 3.01
173. 912 O ARG 126 A <--> 14419 O SER 871 B 3.42
174. 912 O ARG 126 A <--> 14422 N ASP 872 B 2.27
175. 912 O ARG 126 A <--> 14423 CA ASP 872 B 2.02
176. 912 O ARG 126 A <--> 14424 C ASP 872 B 3.41
177. 912 O ARG 126 A <--> 14426 CB ASP 872 B 2.59
178. 912 O ARG 126 A <--> 14427 CG ASP 872 B 3.27
179. 912 O ARG 126 A <--> 14428 OD1 ASP 872 B 3.54
180. 913 CB ARG 126 A <--> 14423 CA ASP 872 B 3.50
181. 913 CB ARG 126 A <--> 14426 CB ASP 872 B 2.29
182. 913 CB ARG 126 A <--> 14427 CG ASP 872 B 1.83
183. 913 CB ARG 126 A <--> 14428 OD1 ASP 872 B 2.87
184. 913 CB ARG 126 A <--> 14429 OD2 ASP 872 B 1.39
185. 914 CG ARG 126 A <--> 14426 CB ASP 872 B 3.15
186. 914 CG ARG 126 A <--> 14427 CG ASP 872 B 2.67
187. 914 CG ARG 126 A <--> 14428 OD1 ASP 872 B 3.80
188. 914 CG ARG 126 A <--> 14429 OD2 ASP 872 B 1.62
189. 915 CD ARG 126 A <--> 14429 OD2 ASP 872 B 2.91

190.	916	NE	ARG	126	A	<-->	14429	OD2	ASP	872	B	3.13
191.	920	N	GLY	127	A	<-->	14426	CB	ASP	872	B	3.76
192.	923	O	GLY	127	A	<-->	14402	NE1	TRP	869	B	3.79
193.	941	CA	LEU	130	A	<-->	13175	CD2	PHE	710	B	3.87
194.	941	CA	LEU	130	A	<-->	13177	CE2	PHE	710	B	2.89
195.	941	CA	LEU	130	A	<-->	13178	CZ	PHE	710	B	3.10
196.	942	C	LEU	130	A	<-->	13175	CD2	PHE	710	B	3.71
197.	942	C	LEU	130	A	<-->	13176	CE1	PHE	710	B	3.68
198.	942	C	LEU	130	A	<-->	13177	CE2	PHE	710	B	2.41
199.	942	C	LEU	130	A	<-->	13178	CZ	PHE	710	B	2.38
200.	943	O	LEU	130	A	<-->	13175	CD2	PHE	710	B	3.43
201.	943	O	LEU	130	A	<-->	13177	CE2	PHE	710	B	2.18
202.	943	O	LEU	130	A	<-->	13178	CZ	PHE	710	B	2.61
203.	943	O	LEU	130	A	<-->	13445	CG1	VAL	747	B	3.90
204.	943	O	LEU	130	A	<-->	13446	CG2	VAL	747	B	3.75
205.	944	CB	LEU	130	A	<-->	12768	CD1	LEU	661	B	3.89
206.	944	CB	LEU	130	A	<-->	13173	CG	PHE	710	B	3.82
207.	944	CB	LEU	130	A	<-->	13174	CD1	PHE	710	B	3.81
208.	944	CB	LEU	130	A	<-->	13175	CD2	PHE	710	B	3.25
209.	944	CB	LEU	130	A	<-->	13176	CE1	PHE	710	B	3.29
210.	944	CB	LEU	130	A	<-->	13177	CE2	PHE	710	B	2.63
211.	944	CB	LEU	130	A	<-->	13178	CZ	PHE	710	B	2.66
212.	945	CG	LEU	130	A	<-->	12768	CD1	LEU	661	B	3.39
213.	945	CG	LEU	130	A	<-->	13172	CB	PHE	710	B	3.53
214.	945	CG	LEU	130	A	<-->	13173	CG	PHE	710	B	2.31
215.	945	CG	LEU	130	A	<-->	13174	CD1	PHE	710	B	2.57
216.	945	CG	LEU	130	A	<-->	13175	CD2	PHE	710	B	1.77
217.	945	CG	LEU	130	A	<-->	13176	CE1	PHE	710	B	2.44
218.	945	CG	LEU	130	A	<-->	13177	CE2	PHE	710	B	1.56
219.	945	CG	LEU	130	A	<-->	13178	CZ	PHE	710	B	1.97
220.	946	CD1	LEU	130	A	<-->	12763	CA	LEU	661	B	3.65
221.	946	CD1	LEU	130	A	<-->	12766	CB	LEU	661	B	2.63
222.	946	CD1	LEU	130	A	<-->	12767	CG	LEU	661	B	2.45
223.	946	CD1	LEU	130	A	<-->	12768	CD1	LEU	661	B	2.08
224.	946	CD1	LEU	130	A	<-->	13172	CB	PHE	710	B	3.40
225.	946	CD1	LEU	130	A	<-->	13173	CG	PHE	710	B	2.59
226.	946	CD1	LEU	130	A	<-->	13174	CD1	PHE	710	B	3.50
227.	946	CD1	LEU	130	A	<-->	13175	CD2	PHE	710	B	1.58
228.	946	CD1	LEU	130	A	<-->	13176	CE1	PHE	710	B	3.70
229.	946	CD1	LEU	130	A	<-->	13177	CE2	PHE	710	B	1.99
230.	946	CD1	LEU	130	A	<-->	13178	CZ	PHE	710	B	3.10
231.	947	CD2	LEU	130	A	<-->	12937	CE	LYS	681	B	2.99
232.	947	CD2	LEU	130	A	<-->	12938	NZ	LYS	681	B	3.06
233.	947	CD2	LEU	130	A	<-->	13172	CB	PHE	710	B	2.81
234.	947	CD2	LEU	130	A	<-->	13173	CG	PHE	710	B	1.89
235.	947	CD2	LEU	130	A	<-->	13174	CD1	PHE	710	B	1.80
236.	947	CD2	LEU	130	A	<-->	13175	CD2	PHE	710	B	2.34
237.	947	CD2	LEU	130	A	<-->	13176	CE1	PHE	710	B	2.25
238.	947	CD2	LEU	130	A	<-->	13177	CE2	PHE	710	B	2.71
239.	947	CD2	LEU	130	A	<-->	13178	CZ	PHE	710	B	2.67
240.	948	N	SER	131	A	<-->	13176	CE1	PHE	710	B	3.63
241.	948	N	SER	131	A	<-->	13177	CE2	PHE	710	B	3.21
242.	948	N	SER	131	A	<-->	13178	CZ	PHE	710	B	2.56
243.	949	CA	SER	131	A	<-->	13176	CE1	PHE	710	B	3.81
244.	949	CA	SER	131	A	<-->	13177	CE2	PHE	710	B	3.63
245.	949	CA	SER	131	A	<-->	13178	CZ	PHE	710	B	2.86
246.	949	CA	SER	131	A	<-->	13446	CG2	VAL	747	B	3.43

247.	950	C	SER	131	A	<-->	13446	CG2	VAL	747	B	3.86
248.	952	CB	SER	131	A	<-->	13167	OD2	ASP	709	B	3.48
249.	952	CB	SER	131	A	<-->	13176	CE1	PHE	710	B	3.20
250.	952	CB	SER	131	A	<-->	13178	CZ	PHE	710	B	2.80
251.	953	OG	SER	131	A	<-->	13167	OD2	ASP	709	B	3.11
252.	953	OG	SER	131	A	<-->	13436	CG	LYS	746	B	3.78
253.	953	OG	SER	131	A	<-->	13437	CD	LYS	746	B	2.79
254.	953	OG	SER	131	A	<-->	13438	CE	LYS	746	B	3.14
255.	953	OG	SER	131	A	<-->	13439	NZ	LYS	746	B	3.02
256.	953	OG	SER	131	A	<-->	13446	CG2	VAL	747	B	3.48
257.	954	N	VAL	132	A	<-->	13446	CG2	VAL	747	B	3.36
258.	956	C	VAL	132	A	<-->	13439	NZ	LYS	746	B	3.90
259.	958	CB	VAL	132	A	<-->	13436	CG	LYS	746	B	3.73
260.	959	CG1	VAL	132	A	<-->	13436	CG	LYS	746	B	3.89
261.	960	CG2	VAL	132	A	<-->	13435	CB	LYS	746	B	3.77
262.	960	CG2	VAL	132	A	<-->	13436	CG	LYS	746	B	2.49
263.	960	CG2	VAL	132	A	<-->	13437	CD	LYS	746	B	3.15
264.	960	CG2	VAL	132	A	<-->	13438	CE	LYS	746	B	3.38
265.	960	CG2	VAL	132	A	<-->	13439	NZ	LYS	746	B	2.61
266.	961	N	ALA	133	A	<-->	13439	NZ	LYS	746	B	2.80
267.	962	CA	ALA	133	A	<-->	13439	NZ	LYS	746	B	3.30
268.	962	CA	ALA	133	A	<-->	14392	OE1	GLN	868	B	3.54
269.	965	CB	ALA	133	A	<-->	14397	O	TRP	869	B	3.72
270.	965	CB	ALA	133	A	<-->	14410	C	LEU	870	B	3.72
271.	965	CB	ALA	133	A	<-->	14411	O	LEU	870	B	3.74
272.	978	CG	LEU	135	A	<-->	14421	OG	SER	871	B	2.97
273.	979	CD1	LEU	135	A	<-->	14416	N	SER	871	B	3.86
274.	979	CD1	LEU	135	A	<-->	14420	CB	SER	871	B	3.22
275.	979	CD1	LEU	135	A	<-->	14421	OG	SER	871	B	1.92
276.	980	CD2	LEU	135	A	<-->	14421	OG	SER	871	B	3.62
277.	982	CA	ARG	136	A	<-->	14247	CD1	LEU	848	B	3.50
278.	982	CA	ARG	136	A	<-->	14248	CD2	LEU	848	B	3.80
279.	983	C	ARG	136	A	<-->	14247	CD1	LEU	848	B	3.87
280.	985	CB	ARG	136	A	<-->	14245	CB	LEU	848	B	3.34
281.	985	CB	ARG	136	A	<-->	14246	CG	LEU	848	B	3.05
282.	985	CB	ARG	136	A	<-->	14247	CD1	LEU	848	B	2.30
283.	985	CB	ARG	136	A	<-->	14248	CD2	LEU	848	B	3.37
284.	986	CG	ARG	136	A	<-->	14242	CA	LEU	848	B	3.24
285.	986	CG	ARG	136	A	<-->	14243	C	LEU	848	B	3.83
286.	986	CG	ARG	136	A	<-->	14245	CB	LEU	848	B	2.13
287.	986	CG	ARG	136	A	<-->	14246	CG	LEU	848	B	2.46
288.	986	CG	ARG	136	A	<-->	14247	CD1	LEU	848	B	2.48
289.	986	CG	ARG	136	A	<-->	14248	CD2	LEU	848	B	2.88
290.	986	CG	ARG	136	A	<-->	14249	N	SER	849	B	3.84
291.	987	CD	ARG	136	A	<-->	14241	N	LEU	848	B	3.19
292.	987	CD	ARG	136	A	<-->	14242	CA	LEU	848	B	2.18
293.	987	CD	ARG	136	A	<-->	14243	C	LEU	848	B	3.28
294.	987	CD	ARG	136	A	<-->	14245	CB	LEU	848	B	1.13
295.	987	CD	ARG	136	A	<-->	14246	CG	LEU	848	B	1.12
296.	987	CD	ARG	136	A	<-->	14247	CD1	LEU	848	B	2.02
297.	987	CD	ARG	136	A	<-->	14248	CD2	LEU	848	B	1.69
298.	987	CD	ARG	136	A	<-->	14249	N	SER	849	B	3.72
299.	988	NE	ARG	136	A	<-->	14228	O	ASN	846	B	3.79
300.	988	NE	ARG	136	A	<-->	14230	CG	ASN	846	B	3.26
301.	988	NE	ARG	136	A	<-->	14231	OD1	ASN	846	B	3.63
302.	988	NE	ARG	136	A	<-->	14232	ND2	ASN	846	B	2.64
303.	988	NE	ARG	136	A	<-->	14241	N	LEU	848	B	3.48

304. 988 NE ARG 136 A <--> 14242 CA LEU 848 B 2.96
305. 988 NE ARG 136 A <--> 14245 CB LEU 848 B 1.60
306. 988 NE ARG 136 A <--> 14246 CG LEU 848 B 0.88
307. 988 NE ARG 136 A <--> 14247 CD1 LEU 848 B 1.10
308. 988 NE ARG 136 A <--> 14248 CD2 LEU 848 B 2.37
309. 989 CZ ARG 136 A <--> 14230 CG ASN 846 B 3.74
310. 989 CZ ARG 136 A <--> 14232 ND2 ASN 846 B 2.90
311. 989 CZ ARG 136 A <--> 14245 CB LEU 848 B 2.93
312. 989 CZ ARG 136 A <--> 14246 CG LEU 848 B 1.67
313. 989 CZ ARG 136 A <--> 14247 CD1 LEU 848 B 0.75
314. 989 CZ ARG 136 A <--> 14248 CD2 LEU 848 B 2.64
315. 990 NH1 ARG 136 A <--> 14245 CB LEU 848 B 3.65
316. 990 NH1 ARG 136 A <--> 14246 CG LEU 848 B 2.24
317. 990 NH1 ARG 136 A <--> 14247 CD1 LEU 848 B 1.58
318. 990 NH1 ARG 136 A <--> 14248 CD2 LEU 848 B 2.33
319. 991 NH2 ARG 136 A <--> 14229 CB ASN 846 B 3.88
320. 991 NH2 ARG 136 A <--> 14230 CG ASN 846 B 3.45
321. 991 NH2 ARG 136 A <--> 14232 ND2 ASN 846 B 2.41
322. 991 NH2 ARG 136 A <--> 14246 CG LEU 848 B 2.87
323. 991 NH2 ARG 136 A <--> 14247 CD1 LEU 848 B 1.89
324. 1340 CB LEU 180 A <--> 16339 SG CYS 1125 B 3.31
325. 1341 CG LEU 180 A <--> 16338 CB CYS 1125 B 3.66
326. 1341 CG LEU 180 A <--> 16339 SG CYS 1125 B 3.07
327. 1343 CD2 LEU 180 A <--> 16338 CB CYS 1125 B 3.61
328. 1343 CD2 LEU 180 A <--> 16339 SG CYS 1125 B 3.61
329. 1349 CG GLN 181 A <--> 16539 OH TYR 1151 B 3.88
330. 1350 CD GLN 181 A <--> 16539 OH TYR 1151 B 3.85
331. 1352 NE2 GLN 181 A <--> 16539 OH TYR 1151 B 3.61
332. 1355 C PRO 182 A <--> 16397 ND2 ASN 1132 B 3.62
333. 1356 O PRO 182 A <--> 16380 SG CYS 1130 B 3.59
334. 1356 O PRO 182 A <--> 16397 ND2 ASN 1132 B 2.98
335. 1360 N GLY 183 A <--> 16397 ND2 ASN 1132 B 3.54
336. 1361 CA GLY 183 A <--> 16395 CG ASN 1132 B 3.59
337. 1361 CA GLY 183 A <--> 16397 ND2 ASN 1132 B 2.54
338. 1362 C GLY 183 A <--> 16397 ND2 ASN 1132 B 3.34
339. 1364 N ARG 184 A <--> 16397 ND2 ASN 1132 B 3.43
340. 1366 C ARG 184 A <--> 16401 O GLY 1133 B 3.43
341. 1367 O ARG 184 A <--> 16394 CB ASN 1132 B 2.95
342. 1367 O ARG 184 A <--> 16395 CG ASN 1132 B 3.87
343. 1367 O ARG 184 A <--> 16401 O GLY 1133 B 3.02
344. 1368 CB ARG 184 A <--> 16534 CD1 TYR 1151 B 3.80
345. 1368 CB ARG 184 A <--> 16536 CE1 TYR 1151 B 3.06
346. 1368 CB ARG 184 A <--> 16538 CZ TYR 1151 B 3.37
347. 1368 CB ARG 184 A <--> 16539 OH TYR 1151 B 3.47
348. 1369 CG ARG 184 A <--> 16534 CD1 TYR 1151 B 3.57
349. 1369 CG ARG 184 A <--> 16536 CE1 TYR 1151 B 3.32
350. 1369 CG ARG 184 A <--> 16538 CZ TYR 1151 B 3.58
351. 1370 CD ARG 184 A <--> 16533 CG TYR 1151 B 3.21
352. 1370 CD ARG 184 A <--> 16534 CD1 TYR 1151 B 3.20
353. 1370 CD ARG 184 A <--> 16535 CD2 TYR 1151 B 3.03
354. 1370 CD ARG 184 A <--> 16536 CE1 TYR 1151 B 3.02
355. 1370 CD ARG 184 A <--> 16537 CE2 TYR 1151 B 2.84
356. 1370 CD ARG 184 A <--> 16538 CZ TYR 1151 B 2.84
357. 1370 CD ARG 184 A <--> 16539 OH TYR 1151 B 3.57
358. 1371 NE ARG 184 A <--> 16532 CB TYR 1151 B 3.22
359. 1371 NE ARG 184 A <--> 16533 CG TYR 1151 B 1.92
360. 1371 NE ARG 184 A <--> 16534 CD1 TYR 1151 B 1.86

361.	1371	NE	ARG	184	A	<-->	16535	CD2	TYR	1151	B	1.89
362.	1371	NE	ARG	184	A	<-->	16536	CE1	TYR	1151	B	1.79
363.	1371	NE	ARG	184	A	<-->	16537	CE2	TYR	1151	B	1.81
364.	1371	NE	ARG	184	A	<-->	16538	CZ	TYR	1151	B	1.76
365.	1371	NE	ARG	184	A	<-->	16539	OH	TYR	1151	B	2.95
366.	1372	CZ	ARG	184	A	<-->	16529	CA	TYR	1151	B	3.87
367.	1372	CZ	ARG	184	A	<-->	16532	CB	TYR	1151	B	2.81
368.	1372	CZ	ARG	184	A	<-->	16533	CG	TYR	1151	B	1.54
369.	1372	CZ	ARG	184	A	<-->	16534	CD1	TYR	1151	B	2.27
370.	1372	CZ	ARG	184	A	<-->	16535	CD2	TYR	1151	B	0.59
371.	1372	CZ	ARG	184	A	<-->	16536	CE1	TYR	1151	B	2.45
372.	1372	CZ	ARG	184	A	<-->	16537	CE2	TYR	1151	B	1.09
373.	1372	CZ	ARG	184	A	<-->	16538	CZ	TYR	1151	B	2.00
374.	1372	CZ	ARG	184	A	<-->	16539	OH	TYR	1151	B	3.28
375.	1372	CZ	ARG	184	A	<-->	16540	N	GLN	1152	B	3.38
376.	1373	NH1	ARG	184	A	<-->	16532	CB	TYR	1151	B	3.46
377.	1373	NH1	ARG	184	A	<-->	16533	CG	TYR	1151	B	2.62
378.	1373	NH1	ARG	184	A	<-->	16534	CD1	TYR	1151	B	3.58
379.	1373	NH1	ARG	184	A	<-->	16535	CD2	TYR	1151	B	1.45
380.	1373	NH1	ARG	184	A	<-->	16536	CE1	TYR	1151	B	3.76
381.	1373	NH1	ARG	184	A	<-->	16537	CE2	TYR	1151	B	1.83
382.	1373	NH1	ARG	184	A	<-->	16538	CZ	TYR	1151	B	3.06
383.	1373	NH1	ARG	184	A	<-->	16540	N	GLN	1152	B	3.88
384.	1374	NH2	ARG	184	A	<-->	16529	CA	TYR	1151	B	3.13
385.	1374	NH2	ARG	184	A	<-->	16530	C	TYR	1151	B	3.04
386.	1374	NH2	ARG	184	A	<-->	16532	CB	TYR	1151	B	2.62
387.	1374	NH2	ARG	184	A	<-->	16533	CG	TYR	1151	B	1.47
388.	1374	NH2	ARG	184	A	<-->	16534	CD1	TYR	1151	B	2.18
389.	1374	NH2	ARG	184	A	<-->	16535	CD2	TYR	1151	B	1.00
390.	1374	NH2	ARG	184	A	<-->	16536	CE1	TYR	1151	B	2.51
391.	1374	NH2	ARG	184	A	<-->	16537	CE2	TYR	1151	B	1.59
392.	1374	NH2	ARG	184	A	<-->	16538	CZ	TYR	1151	B	2.27
393.	1374	NH2	ARG	184	A	<-->	16539	OH	TYR	1151	B	3.55
394.	1374	NH2	ARG	184	A	<-->	16540	N	GLN	1152	B	2.21
395.	1374	NH2	ARG	184	A	<-->	16541	CA	GLN	1152	B	3.29
396.	1374	NH2	ARG	184	A	<-->	16544	CB	GLN	1152	B	3.54
397.	1375	N	HIS	185	A	<-->	16401	O	GLY	1133	B	3.23
398.	1375	N	HIS	185	A	<-->	16534	CD1	TYR	1151	B	3.05
399.	1375	N	HIS	185	A	<-->	16536	CE1	TYR	1151	B	3.07
400.	1376	CA	HIS	185	A	<-->	16400	C	GLY	1133	B	3.47
401.	1376	CA	HIS	185	A	<-->	16401	O	GLY	1133	B	2.43
402.	1376	CA	HIS	185	A	<-->	16402	N	ALA	1134	B	3.84
403.	1376	CA	HIS	185	A	<-->	16403	CA	ALA	1134	B	3.26
404.	1376	CA	HIS	185	A	<-->	16406	CB	ALA	1134	B	3.82
405.	1376	CA	HIS	185	A	<-->	16534	CD1	TYR	1151	B	3.22
406.	1376	CA	HIS	185	A	<-->	16536	CE1	TYR	1151	B	3.74
407.	1377	C	HIS	185	A	<-->	16401	O	GLY	1133	B	3.37
408.	1377	C	HIS	185	A	<-->	16403	CA	ALA	1134	B	3.17
409.	1377	C	HIS	185	A	<-->	16406	CB	ALA	1134	B	3.13
410.	1377	C	HIS	185	A	<-->	16532	CB	TYR	1151	B	3.63
411.	1377	C	HIS	185	A	<-->	16533	CG	TYR	1151	B	3.76
412.	1377	C	HIS	185	A	<-->	16534	CD1	TYR	1151	B	3.19
413.	1378	O	HIS	185	A	<-->	16532	CB	TYR	1151	B	3.04
414.	1378	O	HIS	185	A	<-->	16533	CG	TYR	1151	B	3.07
415.	1378	O	HIS	185	A	<-->	16534	CD1	TYR	1151	B	2.80
416.	1378	O	HIS	185	A	<-->	16536	CE1	TYR	1151	B	3.72
417.	1379	CB	HIS	185	A	<-->	16400	C	GLY	1133	B	3.87

418.	1379	CB	HIS	185	A	<-->	16401	O	GLY	1133	B	3.04
419.	1379	CB	HIS	185	A	<-->	16403	CA	ALA	1134	B	3.33
420.	1379	CB	HIS	185	A	<-->	16404	C	ALA	1134	B	3.89
421.	1379	CB	HIS	185	A	<-->	16407	N	GLN	1135	B	3.54
422.	1379	CB	HIS	185	A	<-->	16534	CD1	TYR	1151	B	3.07
423.	1379	CB	HIS	185	A	<-->	16536	CE1	TYR	1151	B	3.67
424.	1380	CG	HIS	185	A	<-->	16400	C	GLY	1133	B	3.80
425.	1380	CG	HIS	185	A	<-->	16401	O	GLY	1133	B	3.02
426.	1382	CD2	HIS	185	A	<-->	16400	C	GLY	1133	B	3.27
427.	1382	CD2	HIS	185	A	<-->	16401	O	GLY	1133	B	2.51
428.	1383	CE1	HIS	185	A	<-->	15705	CB	ALA	1041	B	3.74
429.	1384	NE2	HIS	185	A	<-->	16401	O	GLY	1133	B	3.73
430.	1385	N	THR	186	A	<-->	16400	C	GLY	1133	B	3.80
431.	1385	N	THR	186	A	<-->	16401	O	GLY	1133	B	3.43
432.	1385	N	THR	186	A	<-->	16402	N	ALA	1134	B	3.38
433.	1385	N	THR	186	A	<-->	16403	CA	ALA	1134	B	2.22
434.	1385	N	THR	186	A	<-->	16404	C	ALA	1134	B	3.21
435.	1385	N	THR	186	A	<-->	16406	CB	ALA	1134	B	1.85
436.	1385	N	THR	186	A	<-->	16407	N	GLN	1135	B	3.39
437.	1386	CA	THR	186	A	<-->	16362	CD1	PHE	1128	B	3.06
438.	1386	CA	THR	186	A	<-->	16364	CE1	PHE	1128	B	2.73
439.	1386	CA	THR	186	A	<-->	16403	CA	ALA	1134	B	3.08
440.	1386	CA	THR	186	A	<-->	16404	C	ALA	1134	B	3.77
441.	1386	CA	THR	186	A	<-->	16406	CB	ALA	1134	B	2.00
442.	1387	C	THR	186	A	<-->	16362	CD1	PHE	1128	B	3.89
443.	1387	C	THR	186	A	<-->	16364	CE1	PHE	1128	B	3.01
444.	1387	C	THR	186	A	<-->	16366	CZ	PHE	1128	B	3.88
445.	1387	C	THR	186	A	<-->	16406	CB	ALA	1134	B	3.19
446.	1387	C	THR	186	A	<-->	16407	N	GLN	1135	B	3.90
447.	1387	C	THR	186	A	<-->	16410	O	GLN	1135	B	3.57
448.	1387	C	THR	186	A	<-->	16525	CA	GLY	1150	B	3.81
449.	1387	C	THR	186	A	<-->	16526	C	GLY	1150	B	3.31
450.	1387	C	THR	186	A	<-->	16527	O	GLY	1150	B	3.82
451.	1387	C	THR	186	A	<-->	16528	N	TYR	1151	B	3.01
452.	1387	C	THR	186	A	<-->	16529	CA	TYR	1151	B	3.49
453.	1387	C	THR	186	A	<-->	16532	CB	TYR	1151	B	3.11
454.	1388	O	THR	186	A	<-->	16406	CB	ALA	1134	B	3.77
455.	1388	O	THR	186	A	<-->	16407	N	GLN	1135	B	3.61
456.	1388	O	THR	186	A	<-->	16410	O	GLN	1135	B	3.35
457.	1388	O	THR	186	A	<-->	16525	CA	GLY	1150	B	3.73
458.	1388	O	THR	186	A	<-->	16526	C	GLY	1150	B	2.85
459.	1388	O	THR	186	A	<-->	16527	O	GLY	1150	B	3.38
460.	1388	O	THR	186	A	<-->	16528	N	TYR	1151	B	2.16
461.	1388	O	THR	186	A	<-->	16529	CA	TYR	1151	B	2.35
462.	1388	O	THR	186	A	<-->	16530	C	TYR	1151	B	3.81
463.	1388	O	THR	186	A	<-->	16532	CB	TYR	1151	B	2.17
464.	1388	O	THR	186	A	<-->	16533	CG	TYR	1151	B	3.33
465.	1388	O	THR	186	A	<-->	16534	CD1	TYR	1151	B	3.63
466.	1389	CB	THR	186	A	<-->	16361	CG	PHE	1128	B	3.90
467.	1389	CB	THR	186	A	<-->	16362	CD1	PHE	1128	B	2.61
468.	1389	CB	THR	186	A	<-->	16364	CE1	PHE	1128	B	2.52
469.	1389	CB	THR	186	A	<-->	16366	CZ	PHE	1128	B	3.78
470.	1389	CB	THR	186	A	<-->	16403	CA	ALA	1134	B	2.94
471.	1389	CB	THR	186	A	<-->	16404	C	ALA	1134	B	3.37
472.	1389	CB	THR	186	A	<-->	16405	O	ALA	1134	B	3.88
473.	1389	CB	THR	186	A	<-->	16406	CB	ALA	1134	B	1.52
474.	1389	CB	THR	186	A	<-->	16407	N	GLN	1135	B	3.86

475.	1390	OG1	THR	186	A	<-->	15987	O	ASP	1078	B	3.73
476.	1390	OG1	THR	186	A	<-->	16362	CD1	PHE	1128	B	2.86
477.	1390	OG1	THR	186	A	<-->	16364	CE1	PHE	1128	B	3.40
478.	1390	OG1	THR	186	A	<-->	16402	N	ALA	1134	B	3.24
479.	1390	OG1	THR	186	A	<-->	16403	CA	ALA	1134	B	2.68
480.	1390	OG1	THR	186	A	<-->	16404	C	ALA	1134	B	3.47
481.	1390	OG1	THR	186	A	<-->	16405	O	ALA	1134	B	3.80
482.	1390	OG1	THR	186	A	<-->	16406	CB	ALA	1134	B	1.26
483.	1391	CG2	THR	186	A	<-->	16360	CB	PHE	1128	B	3.85
484.	1391	CG2	THR	186	A	<-->	16361	CG	PHE	1128	B	2.77
485.	1391	CG2	THR	186	A	<-->	16362	CD1	PHE	1128	B	1.67
486.	1391	CG2	THR	186	A	<-->	16363	CD2	PHE	1128	B	3.42
487.	1391	CG2	THR	186	A	<-->	16364	CE1	PHE	1128	B	1.41
488.	1391	CG2	THR	186	A	<-->	16365	CE2	PHE	1128	B	3.31
489.	1391	CG2	THR	186	A	<-->	16366	CZ	PHE	1128	B	2.47
490.	1391	CG2	THR	186	A	<-->	16406	CB	ALA	1134	B	3.01
491.	1391	CG2	THR	186	A	<-->	16421	SG	CYS	1136	B	3.89
492.	1392	N	VAL	187	A	<-->	16362	CD1	PHE	1128	B	3.69
493.	1392	N	VAL	187	A	<-->	16364	CE1	PHE	1128	B	2.47
494.	1392	N	VAL	187	A	<-->	16366	CZ	PHE	1128	B	2.92
495.	1392	N	VAL	187	A	<-->	16410	O	GLN	1135	B	3.73
496.	1392	N	VAL	187	A	<-->	16525	CA	GLY	1150	B	3.08
497.	1392	N	VAL	187	A	<-->	16526	C	GLY	1150	B	2.98
498.	1392	N	VAL	187	A	<-->	16527	O	GLY	1150	B	3.45
499.	1392	N	VAL	187	A	<-->	16528	N	TYR	1151	B	3.22
500.	1392	N	VAL	187	A	<-->	16532	CB	TYR	1151	B	3.74
501.	1393	CA	VAL	187	A	<-->	16319	OG1	THR	1122	B	3.49
502.	1393	CA	VAL	187	A	<-->	16364	CE1	PHE	1128	B	3.65
503.	1393	CA	VAL	187	A	<-->	16366	CZ	PHE	1128	B	3.63
504.	1393	CA	VAL	187	A	<-->	16410	O	GLN	1135	B	3.74
505.	1393	CA	VAL	187	A	<-->	16524	N	GLY	1150	B	3.14
506.	1393	CA	VAL	187	A	<-->	16525	CA	GLY	1150	B	1.85
507.	1393	CA	VAL	187	A	<-->	16526	C	GLY	1150	B	1.89
508.	1393	CA	VAL	187	A	<-->	16527	O	GLY	1150	B	2.32
509.	1393	CA	VAL	187	A	<-->	16528	N	TYR	1151	B	2.67
510.	1393	CA	VAL	187	A	<-->	16529	CA	TYR	1151	B	3.66
511.	1393	CA	VAL	187	A	<-->	16532	CB	TYR	1151	B	3.61
512.	1394	C	VAL	187	A	<-->	16524	N	GLY	1150	B	3.57
513.	1394	C	VAL	187	A	<-->	16525	CA	GLY	1150	B	2.66
514.	1394	C	VAL	187	A	<-->	16526	C	GLY	1150	B	1.90
515.	1394	C	VAL	187	A	<-->	16527	O	GLY	1150	B	1.45
516.	1394	C	VAL	187	A	<-->	16528	N	TYR	1151	B	2.77
517.	1394	C	VAL	187	A	<-->	16529	CA	TYR	1151	B	3.21
518.	1394	C	VAL	187	A	<-->	16530	C	TYR	1151	B	3.82
519.	1394	C	VAL	187	A	<-->	16532	CB	TYR	1151	B	2.75
520.	1395	O	VAL	187	A	<-->	16524	N	GLY	1150	B	3.31
521.	1395	O	VAL	187	A	<-->	16525	CA	GLY	1150	B	2.77
522.	1395	O	VAL	187	A	<-->	16526	C	GLY	1150	B	1.60
523.	1395	O	VAL	187	A	<-->	16527	O	GLY	1150	B	0.53
524.	1395	O	VAL	187	A	<-->	16528	N	TYR	1151	B	2.45
525.	1395	O	VAL	187	A	<-->	16529	CA	TYR	1151	B	2.62
526.	1395	O	VAL	187	A	<-->	16530	C	TYR	1151	B	2.80
527.	1395	O	VAL	187	A	<-->	16531	O	TYR	1151	B	3.15
528.	1395	O	VAL	187	A	<-->	16532	CB	TYR	1151	B	2.42
529.	1395	O	VAL	187	A	<-->	16533	CG	TYR	1151	B	3.68
530.	1395	O	VAL	187	A	<-->	16540	N	GLN	1152	B	3.56
531.	1395	O	VAL	187	A	<-->	16549	N	GLY	1153	B	3.74

532.	1396	CB	VAL	187	A	<-->	16409	C	GLN	1135	B	3.84
533.	1396	CB	VAL	187	A	<-->	16410	O	GLN	1135	B	2.62
534.	1396	CB	VAL	187	A	<-->	16519	C	PRO	1149	B	3.19
535.	1396	CB	VAL	187	A	<-->	16520	O	PRO	1149	B	3.34
536.	1396	CB	VAL	187	A	<-->	16524	N	GLY	1150	B	2.29
537.	1396	CB	VAL	187	A	<-->	16525	CA	GLY	1150	B	0.92
538.	1396	CB	VAL	187	A	<-->	16526	C	GLY	1150	B	1.30
539.	1396	CB	VAL	187	A	<-->	16527	O	GLY	1150	B	2.40
540.	1396	CB	VAL	187	A	<-->	16528	N	TYR	1151	B	1.84
541.	1396	CB	VAL	187	A	<-->	16529	CA	TYR	1151	B	3.19
542.	1396	CB	VAL	187	A	<-->	16530	C	TYR	1151	B	3.87
543.	1396	CB	VAL	187	A	<-->	16531	O	TYR	1151	B	3.71
544.	1396	CB	VAL	187	A	<-->	16532	CB	TYR	1151	B	3.78
545.	1397	CG1	VAL	187	A	<-->	16410	O	GLN	1135	B	3.67
546.	1397	CG1	VAL	187	A	<-->	16518	CA	PRO	1149	B	3.20
547.	1397	CG1	VAL	187	A	<-->	16519	C	PRO	1149	B	2.01
548.	1397	CG1	VAL	187	A	<-->	16520	O	PRO	1149	B	2.66
549.	1397	CG1	VAL	187	A	<-->	16521	CB	PRO	1149	B	3.83
550.	1397	CG1	VAL	187	A	<-->	16524	N	GLY	1150	B	0.84
551.	1397	CG1	VAL	187	A	<-->	16525	CA	GLY	1150	B	0.81
552.	1397	CG1	VAL	187	A	<-->	16526	C	GLY	1150	B	1.51
553.	1397	CG1	VAL	187	A	<-->	16527	O	GLY	1150	B	2.22
554.	1397	CG1	VAL	187	A	<-->	16528	N	TYR	1151	B	2.50
555.	1397	CG1	VAL	187	A	<-->	16529	CA	TYR	1151	B	3.67
556.	1397	CG1	VAL	187	A	<-->	16530	C	TYR	1151	B	3.72
557.	1397	CG1	VAL	187	A	<-->	16531	O	TYR	1151	B	3.17
558.	1397	CG1	VAL	187	A	<-->	16552	O	GLY	1153	B	3.46
559.	1398	CG2	VAL	187	A	<-->	16409	C	GLN	1135	B	2.93
560.	1398	CG2	VAL	187	A	<-->	16410	O	GLN	1135	B	1.91
561.	1398	CG2	VAL	187	A	<-->	16416	N	CYS	1136	B	3.40
562.	1398	CG2	VAL	187	A	<-->	16417	CA	CYS	1136	B	3.03
563.	1398	CG2	VAL	187	A	<-->	16420	CB	CYS	1136	B	3.55
564.	1398	CG2	VAL	187	A	<-->	16421	SG	CYS	1136	B	3.17
565.	1398	CG2	VAL	187	A	<-->	16519	C	PRO	1149	B	3.40
566.	1398	CG2	VAL	187	A	<-->	16520	O	PRO	1149	B	2.96
567.	1398	CG2	VAL	187	A	<-->	16524	N	GLY	1150	B	3.06
568.	1398	CG2	VAL	187	A	<-->	16525	CA	GLY	1150	B	1.93
569.	1398	CG2	VAL	187	A	<-->	16526	C	GLY	1150	B	2.82
570.	1398	CG2	VAL	187	A	<-->	16528	N	TYR	1151	B	2.97
571.	1399	N	SER	188	A	<-->	16324	O	SER	1123	B	3.64
572.	1399	N	SER	188	A	<-->	16328	CA	PRO	1124	B	3.69
573.	1399	N	SER	188	A	<-->	16331	CB	PRO	1124	B	3.48
574.	1399	N	SER	188	A	<-->	16526	C	GLY	1150	B	3.24
575.	1399	N	SER	188	A	<-->	16527	O	GLY	1150	B	2.63
576.	1399	N	SER	188	A	<-->	16532	CB	TYR	1151	B	3.18
577.	1400	CA	SER	188	A	<-->	16324	O	SER	1123	B	3.36
578.	1400	CA	SER	188	A	<-->	16328	CA	PRO	1124	B	3.44
579.	1400	CA	SER	188	A	<-->	16331	CB	PRO	1124	B	3.63
580.	1400	CA	SER	188	A	<-->	16527	O	GLY	1150	B	3.19
581.	1400	CA	SER	188	A	<-->	16532	CB	TYR	1151	B	3.34
582.	1401	C	SER	188	A	<-->	16323	C	SER	1123	B	2.92
583.	1401	C	SER	188	A	<-->	16324	O	SER	1123	B	2.24
584.	1401	C	SER	188	A	<-->	16327	N	PRO	1124	B	2.94
585.	1401	C	SER	188	A	<-->	16328	CA	PRO	1124	B	2.22
586.	1401	C	SER	188	A	<-->	16329	C	PRO	1124	B	3.27
587.	1401	C	SER	188	A	<-->	16331	CB	PRO	1124	B	2.95
588.	1401	C	SER	188	A	<-->	16334	N	CYS	1125	B	3.51

589. 1402 O SER 188 A <--> 16323 C SER 1123 B 2.50
590. 1402 O SER 188 A <--> 16324 O SER 1123 B 2.37
591. 1402 O SER 188 A <--> 16327 N PRO 1124 B 2.00
592. 1402 O SER 188 A <--> 16328 CA PRO 1124 B 1.06
593. 1402 O SER 188 A <--> 16329 C PRO 1124 B 2.36
594. 1402 O SER 188 A <--> 16330 O PRO 1124 B 3.35
595. 1402 O SER 188 A <--> 16331 CB PRO 1124 B 1.82
596. 1402 O SER 188 A <--> 16332 CG PRO 1124 B 2.96
597. 1402 O SER 188 A <--> 16333 CD PRO 1124 B 2.91
598. 1402 O SER 188 A <--> 16334 N CYS 1125 B 2.92
599. 1403 CB SER 188 A <--> 16331 CB PRO 1124 B 3.87
600. 1403 CB SER 188 A <--> 16532 CB TYR 1151 B 3.48
601. 1403 CB SER 188 A <--> 16533 CG TYR 1151 B 3.73
602. 1403 CB SER 188 A <--> 16535 CD2 TYR 1151 B 3.74
603. 1404 OG SER 188 A <--> 16535 CD2 TYR 1151 B 3.71
604. 1405 N GLY 189 A <--> 16323 C SER 1123 B 3.06
605. 1405 N GLY 189 A <--> 16324 O SER 1123 B 2.09
606. 1405 N GLY 189 A <--> 16327 N PRO 1124 B 3.45
607. 1405 N GLY 189 A <--> 16328 CA PRO 1124 B 2.93
608. 1405 N GLY 189 A <--> 16329 C PRO 1124 B 3.59
609. 1405 N GLY 189 A <--> 16334 N CYS 1125 B 3.65
610. 1406 CA GLY 189 A <--> 16322 CA SER 1123 B 3.84
611. 1406 CA GLY 189 A <--> 16323 C SER 1123 B 2.71
612. 1406 CA GLY 189 A <--> 16324 O SER 1123 B 2.06
613. 1406 CA GLY 189 A <--> 16325 CB SER 1123 B 3.76
614. 1406 CA GLY 189 A <--> 16327 N PRO 1124 B 3.16
615. 1406 CA GLY 189 A <--> 16328 CA PRO 1124 B 2.91
616. 1406 CA GLY 189 A <--> 16329 C PRO 1124 B 3.11
617. 1406 CA GLY 189 A <--> 16330 O PRO 1124 B 3.80
618. 1406 CA GLY 189 A <--> 16334 N CYS 1125 B 3.28
619. 1407 C GLY 189 A <--> 16323 C SER 1123 B 3.78
620. 1407 C GLY 189 A <--> 16324 O SER 1123 B 3.41
621. 1407 C GLY 189 A <--> 16327 N PRO 1124 B 3.79
622. 1407 C GLY 189 A <--> 16328 CA PRO 1124 B 3.13
623. 1407 C GLY 189 A <--> 16329 C PRO 1124 B 2.66
624. 1407 C GLY 189 A <--> 16330 O PRO 1124 B 3.40
625. 1407 C GLY 189 A <--> 16334 N CYS 1125 B 2.29
626. 1407 C GLY 189 A <--> 16335 CA CYS 1125 B 3.06
627. 1407 C GLY 189 A <--> 16338 CB CYS 1125 B 3.43
628. 1408 O GLY 189 A <--> 16328 CA PRO 1124 B 2.86
629. 1408 O GLY 189 A <--> 16329 C PRO 1124 B 2.29
630. 1408 O GLY 189 A <--> 16330 O PRO 1124 B 3.29
631. 1408 O GLY 189 A <--> 16334 N CYS 1125 B 1.44
632. 1408 O GLY 189 A <--> 16335 CA CYS 1125 B 2.36
633. 1408 O GLY 189 A <--> 16336 C CYS 1125 B 3.65
634. 1408 O GLY 189 A <--> 16338 CB CYS 1125 B 2.67
635. 1408 O GLY 189 A <--> 16339 SG CYS 1125 B 3.16
636. 1409 N GLY 190 A <--> 16329 C PRO 1124 B 3.50
637. 1409 N GLY 190 A <--> 16330 O PRO 1124 B 3.89
638. 1409 N GLY 190 A <--> 16334 N CYS 1125 B 3.07
639. 1409 N GLY 190 A <--> 16335 CA CYS 1125 B 3.27
640. 1409 N GLY 190 A <--> 16338 CB CYS 1125 B 3.43
641. 1410 CA GLY 190 A <--> 16334 N CYS 1125 B 3.20
642. 1410 CA GLY 190 A <--> 16335 CA CYS 1125 B 2.79
643. 1410 CA GLY 190 A <--> 16338 CB CYS 1125 B 2.52
644. 1411 C GLY 190 A <--> 16338 CB CYS 1125 B 3.72
645. 1602 CZ ARG 216 A <--> 16197 OH TYR 1106 B 3.78

646. 1603 NH1 ARG 216 A <--> 16337 O CYS 1125 B 3.49
647. 1603 NH1 ARG 216 A <--> 16346 OD1 ASP 1126 B 3.45
648. 1604 NH2 ARG 216 A <--> 16197 OH TYR 1106 B 3.13
649. 1604 NH2 ARG 216 A <--> 16348 N ASN 1127 B 3.79
650. 1604 NH2 ARG 216 A <--> 16353 CG ASN 1127 B 3.84
651. 1604 NH2 ARG 216 A <--> 16355 ND2 ASN 1127 B 3.38
652. 1634 O ALA 220 A <--> 16355 ND2 ASN 1127 B 3.28
653. 1636 N ALA 221 A <--> 16373 OD1 ASP 1129 B 3.81
654. 1636 N ALA 221 A <--> 16374 OD2 ASP 1129 B 3.72
655. 1637 CA ALA 221 A <--> 16352 CB ASN 1127 B 3.80
656. 1637 CA ALA 221 A <--> 16372 CG ASP 1129 B 2.99
657. 1637 CA ALA 221 A <--> 16373 OD1 ASP 1129 B 2.43
658. 1637 CA ALA 221 A <--> 16374 OD2 ASP 1129 B 2.94
659. 1638 C ALA 221 A <--> 16370 O ASP 1129 B 3.40
660. 1638 C ALA 221 A <--> 16372 CG ASP 1129 B 3.43
661. 1638 C ALA 221 A <--> 16373 OD1 ASP 1129 B 2.65
662. 1638 C ALA 221 A <--> 16374 OD2 ASP 1129 B 3.77
663. 1639 O ALA 221 A <--> 16370 O ASP 1129 B 3.78
664. 1639 O ALA 221 A <--> 16373 OD1 ASP 1129 B 3.78
665. 1640 CB ALA 221 A <--> 16370 O ASP 1129 B 3.30
666. 1640 CB ALA 221 A <--> 16371 CB ASP 1129 B 3.14
667. 1640 CB ALA 221 A <--> 16372 CG ASP 1129 B 1.67
668. 1640 CB ALA 221 A <--> 16373 OD1 ASP 1129 B 1.75
669. 1640 CB ALA 221 A <--> 16374 OD2 ASP 1129 B 1.41
670. 1641 N ARG 222 A <--> 16350 C ASN 1127 B 3.57
671. 1641 N ARG 222 A <--> 16351 O ASN 1127 B 3.22
672. 1641 N ARG 222 A <--> 16352 CB ASN 1127 B 3.26
673. 1641 N ARG 222 A <--> 16367 N ASP 1129 B 3.61
674. 1641 N ARG 222 A <--> 16368 CA ASP 1129 B 3.88
675. 1641 N ARG 222 A <--> 16369 C ASP 1129 B 3.31
676. 1641 N ARG 222 A <--> 16370 O ASP 1129 B 3.18
677. 1641 N ARG 222 A <--> 16372 CG ASP 1129 B 3.33
678. 1641 N ARG 222 A <--> 16373 OD1 ASP 1129 B 2.28
679. 1641 N ARG 222 A <--> 16375 N CYS 1130 B 3.78
680. 1642 CA ARG 222 A <--> 16350 C ASN 1127 B 3.51
681. 1642 CA ARG 222 A <--> 16351 O ASN 1127 B 2.69
682. 1642 CA ARG 222 A <--> 16367 N ASP 1129 B 3.86
683. 1642 CA ARG 222 A <--> 16369 C ASP 1129 B 3.37
684. 1642 CA ARG 222 A <--> 16370 O ASP 1129 B 3.50
685. 1642 CA ARG 222 A <--> 16373 OD1 ASP 1129 B 3.44
686. 1642 CA ARG 222 A <--> 16375 N CYS 1130 B 3.28
687. 1642 CA ARG 222 A <--> 16376 CA CYS 1130 B 3.32
688. 1642 CA ARG 222 A <--> 16379 CB CYS 1130 B 2.97
689. 1643 C ARG 222 A <--> 16351 O ASN 1127 B 3.15
690. 1643 C ARG 222 A <--> 16379 CB CYS 1130 B 3.63
691. 1645 CB ARG 222 A <--> 16351 O ASN 1127 B 3.83
692. 1645 CB ARG 222 A <--> 16369 C ASP 1129 B 2.86
693. 1645 CB ARG 222 A <--> 16370 O ASP 1129 B 2.91
694. 1645 CB ARG 222 A <--> 16375 N CYS 1130 B 2.43
695. 1645 CB ARG 222 A <--> 16376 CA CYS 1130 B 1.93
696. 1645 CB ARG 222 A <--> 16377 C CYS 1130 B 3.38
697. 1645 CB ARG 222 A <--> 16379 CB CYS 1130 B 1.75
698. 1645 CB ARG 222 A <--> 16380 SG CYS 1130 B 3.34
699. 1646 CG ARG 222 A <--> 16369 C ASP 1129 B 3.62
700. 1646 CG ARG 222 A <--> 16370 O ASP 1129 B 3.15
701. 1646 CG ARG 222 A <--> 16375 N CYS 1130 B 3.42
702. 1646 CG ARG 222 A <--> 16376 CA CYS 1130 B 2.54

703.	1646	CG	ARG	222	A	<-->	16377	C	CYS	1130	B	3.68
704.	1646	CG	ARG	222	A	<-->	16379	CB	CYS	1130	B	2.64
705.	1647	CD	ARG	222	A	<-->	16369	C	ASP	1129	B	3.28
706.	1647	CD	ARG	222	A	<-->	16370	O	ASP	1129	B	2.42
707.	1647	CD	ARG	222	A	<-->	16375	N	CYS	1130	B	3.45
708.	1647	CD	ARG	222	A	<-->	16376	CA	CYS	1130	B	2.75
709.	1647	CD	ARG	222	A	<-->	16377	C	CYS	1130	B	3.60
710.	1647	CD	ARG	222	A	<-->	16379	CB	CYS	1130	B	3.56
711.	1648	NE	ARG	222	A	<-->	16370	O	ASP	1129	B	3.76
712.	1650	NH1	ARG	222	A	<-->	16370	O	ASP	1129	B	3.76
713.	1652	N	VAL	223	A	<-->	16344	CB	ASP	1126	B	3.32
714.	1652	N	VAL	223	A	<-->	16347	OD2	ASP	1126	B	3.72
715.	1652	N	VAL	223	A	<-->	16351	O	ASN	1127	B	3.14
716.	1652	N	VAL	223	A	<-->	16379	CB	CYS	1130	B	3.67
717.	1653	CA	VAL	223	A	<-->	16344	CB	ASP	1126	B	2.99
718.	1653	CA	VAL	223	A	<-->	16345	CG	ASP	1126	B	3.08
719.	1653	CA	VAL	223	A	<-->	16347	OD2	ASP	1126	B	2.53
720.	1654	C	VAL	223	A	<-->	16347	OD2	ASP	1126	B	3.31
721.	1655	O	VAL	223	A	<-->	16347	OD2	ASP	1126	B	3.54
722.	1656	CB	VAL	223	A	<-->	16341	CA	ASP	1126	B	3.75
723.	1656	CB	VAL	223	A	<-->	16344	CB	ASP	1126	B	2.22
724.	1656	CB	VAL	223	A	<-->	16345	CG	ASP	1126	B	2.09
725.	1656	CB	VAL	223	A	<-->	16346	OD1	ASP	1126	B	3.34
726.	1656	CB	VAL	223	A	<-->	16347	OD2	ASP	1126	B	1.48
727.	1657	CG1	VAL	223	A	<-->	16340	N	ASP	1126	B	3.02
728.	1657	CG1	VAL	223	A	<-->	16341	CA	ASP	1126	B	2.21
729.	1657	CG1	VAL	223	A	<-->	16342	C	ASP	1126	B	3.08
730.	1657	CG1	VAL	223	A	<-->	16344	CB	ASP	1126	B	0.69
731.	1657	CG1	VAL	223	A	<-->	16345	CG	ASP	1126	B	1.32
732.	1657	CG1	VAL	223	A	<-->	16346	OD1	ASP	1126	B	2.46
733.	1657	CG1	VAL	223	A	<-->	16347	OD2	ASP	1126	B	1.84
734.	1657	CG1	VAL	223	A	<-->	16348	N	ASN	1127	B	3.36
735.	1658	CG2	VAL	223	A	<-->	16344	CB	ASP	1126	B	3.12
736.	1658	CG2	VAL	223	A	<-->	16345	CG	ASP	1126	B	3.31
737.	1658	CG2	VAL	223	A	<-->	16347	OD2	ASP	1126	B	2.73
738.	1762	CG	GLU	236	A	<-->	16347	OD2	ASP	1126	B	3.15
739.	1763	CD	GLU	236	A	<-->	16345	CG	ASP	1126	B	3.70
740.	1763	CD	GLU	236	A	<-->	16347	OD2	ASP	1126	B	2.58
741.	1764	OE1	GLU	236	A	<-->	16347	OD2	ASP	1126	B	3.51
742.	1765	OE2	GLU	236	A	<-->	16345	CG	ASP	1126	B	2.73
743.	1765	OE2	GLU	236	A	<-->	16346	OD1	ASP	1126	B	3.19
744.	1765	OE2	GLU	236	A	<-->	16347	OD2	ASP	1126	B	1.86
745.	1832	C	GLU	245	A	<-->	15701	N	ALA	1041	B	3.76
746.	1832	C	GLU	245	A	<-->	15705	CB	ALA	1041	B	3.81
747.	1833	O	GLU	245	A	<-->	15701	N	ALA	1041	B	3.44
748.	1833	O	GLU	245	A	<-->	15702	CA	ALA	1041	B	3.57
749.	1833	O	GLU	245	A	<-->	15705	CB	ALA	1041	B	2.82
750.	1834	CB	GLU	245	A	<-->	15696	CA	CYS	1040	B	3.34
751.	1834	CB	GLU	245	A	<-->	15697	C	CYS	1040	B	3.63
752.	1834	CB	GLU	245	A	<-->	15701	N	ALA	1041	B	3.29
753.	1835	CG	GLU	245	A	<-->	15687	O	PHE	1039	B	3.77
754.	1835	CG	GLU	245	A	<-->	15696	CA	CYS	1040	B	2.97
755.	1835	CG	GLU	245	A	<-->	15697	C	CYS	1040	B	3.68
756.	1835	CG	GLU	245	A	<-->	15699	CB	CYS	1040	B	3.22
757.	1835	CG	GLU	245	A	<-->	15700	SG	CYS	1040	B	2.83
758.	1835	CG	GLU	245	A	<-->	15701	N	ALA	1041	B	3.43
759.	1836	CD	GLU	245	A	<-->	15686	C	PHE	1039	B	2.93

760. 1836 CD GLU 245 A <--> 15687 O PHE 1039 B 2.40
761. 1836 CD GLU 245 A <--> 15695 N CYS 1040 B 2.78
762. 1836 CD GLU 245 A <--> 15696 CA CYS 1040 B 1.84
763. 1836 CD GLU 245 A <--> 15697 C CYS 1040 B 3.04
764. 1836 CD GLU 245 A <--> 15699 CB CYS 1040 B 2.16
765. 1836 CD GLU 245 A <--> 15700 SG CYS 1040 B 2.67
766. 1836 CD GLU 245 A <--> 15701 N ALA 1041 B 3.35
767. 1837 OE1 GLU 245 A <--> 15685 CA PHE 1039 B 3.49
768. 1837 OE1 GLU 245 A <--> 15686 C PHE 1039 B 2.09
769. 1837 OE1 GLU 245 A <--> 15687 O PHE 1039 B 1.77
770. 1837 OE1 GLU 245 A <--> 15689 CG PHE 1039 B 3.80
771. 1837 OE1 GLU 245 A <--> 15691 CD2 PHE 1039 B 3.24
772. 1837 OE1 GLU 245 A <--> 15693 CE2 PHE 1039 B 3.84
773. 1837 OE1 GLU 245 A <--> 15695 N CYS 1040 B 2.06
774. 1837 OE1 GLU 245 A <--> 15696 CA CYS 1040 B 1.66
775. 1837 OE1 GLU 245 A <--> 15697 C CYS 1040 B 2.81
776. 1837 OE1 GLU 245 A <--> 15698 O CYS 1040 B 3.61
777. 1837 OE1 GLU 245 A <--> 15699 CB CYS 1040 B 2.71
778. 1837 OE1 GLU 245 A <--> 15700 SG CYS 1040 B 3.68
779. 1837 OE1 GLU 245 A <--> 15701 N ALA 1041 B 3.47
780. 1838 OE2 GLU 245 A <--> 15686 C PHE 1039 B 3.10
781. 1838 OE2 GLU 245 A <--> 15687 O PHE 1039 B 2.62
782. 1838 OE2 GLU 245 A <--> 15695 N CYS 1040 B 3.00
783. 1838 OE2 GLU 245 A <--> 15696 CA CYS 1040 B 2.19
784. 1838 OE2 GLU 245 A <--> 15697 C CYS 1040 B 3.48
785. 1838 OE2 GLU 245 A <--> 15699 CB CYS 1040 B 1.59
786. 1838 OE2 GLU 245 A <--> 15700 SG CYS 1040 B 2.24
787. 1838 OE2 GLU 245 A <--> 15701 N ALA 1041 B 3.87
788. 1841 C ASN 246 A <--> 15700 SG CYS 1040 B 3.26
789. 1842 O ASN 246 A <--> 15700 SG CYS 1040 B 2.21
790. 1842 O ASN 246 A <--> 15701 N ALA 1041 B 3.76
791. 1848 CA VAL 247 A <--> 15700 SG CYS 1040 B 3.88
792. 1851 CB VAL 247 A <--> 15667 NZ LYS 1036 B 3.86
793. 1852 CG1 VAL 247 A <--> 15666 CE LYS 1036 B 3.25
794. 1852 CG1 VAL 247 A <--> 15667 NZ LYS 1036 B 2.73
795. 1854 N THR 248 A <--> 15667 NZ LYS 1036 B 3.35
796. 1855 CA THR 248 A <--> 15544 OH TYR 1020 B 3.84
797. 1856 C THR 248 A <--> 15544 OH TYR 1020 B 3.41
798. 1857 O THR 248 A <--> 15543 CZ TYR 1020 B 3.82
799. 1857 O THR 248 A <--> 15544 OH TYR 1020 B 2.71
800. 1857 O THR 248 A <--> 15667 NZ LYS 1036 B 3.21
801. 1858 CB THR 248 A <--> 15544 OH TYR 1020 B 3.36
802. 1869 N LEU 250 A <--> 15529 CB ASN 1019 B 3.42
803. 1869 N LEU 250 A <--> 15530 CG ASN 1019 B 3.31
804. 1869 N LEU 250 A <--> 15532 ND2 ASN 1019 B 2.78
805. 1870 CA LEU 250 A <--> 15529 CB ASN 1019 B 2.69
806. 1870 CA LEU 250 A <--> 15530 CG ASN 1019 B 2.27
807. 1870 CA LEU 250 A <--> 15531 OD1 ASN 1019 B 2.99
808. 1870 CA LEU 250 A <--> 15532 ND2 ASN 1019 B 2.15
809. 1871 C LEU 250 A <--> 15529 CB ASN 1019 B 3.52
810. 1871 C LEU 250 A <--> 15530 CG ASN 1019 B 2.43
811. 1871 C LEU 250 A <--> 15531 OD1 ASN 1019 B 2.83
812. 1871 C LEU 250 A <--> 15532 ND2 ASN 1019 B 1.84
813. 1872 O LEU 250 A <--> 15529 CB ASN 1019 B 3.59
814. 1872 O LEU 250 A <--> 15530 CG ASN 1019 B 2.31
815. 1872 O LEU 250 A <--> 15531 OD1 ASN 1019 B 2.77
816. 1872 O LEU 250 A <--> 15532 ND2 ASN 1019 B 1.26

817. 1873 CB LEU 250 A <--> 15525 N ASN 1019 B 3.53
818. 1873 CB LEU 250 A <--> 15526 CA ASN 1019 B 2.61
819. 1873 CB LEU 250 A <--> 15527 C ASN 1019 B 3.49
820. 1873 CB LEU 250 A <--> 15529 CB ASN 1019 B 1.16
821. 1873 CB LEU 250 A <--> 15530 CG ASN 1019 B 1.24
822. 1873 CB LEU 250 A <--> 15531 OD1 ASN 1019 B 2.17
823. 1873 CB LEU 250 A <--> 15532 ND2 ASN 1019 B 1.93
824. 1874 CG LEU 250 A <--> 15525 N ASN 1019 B 2.64
825. 1874 CG LEU 250 A <--> 15526 CA ASN 1019 B 2.37
826. 1874 CG LEU 250 A <--> 15527 C ASN 1019 B 3.45
827. 1874 CG LEU 250 A <--> 15529 CB ASN 1019 B 1.28
828. 1874 CG LEU 250 A <--> 15530 CG ASN 1019 B 1.41
829. 1874 CG LEU 250 A <--> 15531 OD1 ASN 1019 B 2.59
830. 1874 CG LEU 250 A <--> 15532 ND2 ASN 1019 B 1.58
831. 1874 CG LEU 250 A <--> 15533 N TYR 1020 B 3.78
832. 1875 CD1 LEU 250 A <--> 15511 C ILE 1017 B 3.83
833. 1875 CD1 LEU 250 A <--> 15517 N ASN 1018 B 3.43
834. 1875 CD1 LEU 250 A <--> 15518 CA ASN 1018 B 3.46
835. 1875 CD1 LEU 250 A <--> 15519 C ASN 1018 B 2.50
836. 1875 CD1 LEU 250 A <--> 15520 O ASN 1018 B 3.31
837. 1875 CD1 LEU 250 A <--> 15525 N ASN 1019 B 1.15
838. 1875 CD1 LEU 250 A <--> 15526 CA ASN 1019 B 1.30
839. 1875 CD1 LEU 250 A <--> 15527 C ASN 1019 B 2.40
840. 1875 CD1 LEU 250 A <--> 15528 O ASN 1019 B 3.51
841. 1875 CD1 LEU 250 A <--> 15529 CB ASN 1019 B 1.52
842. 1875 CD1 LEU 250 A <--> 15530 CG ASN 1019 B 2.23
843. 1875 CD1 LEU 250 A <--> 15531 OD1 ASN 1019 B 2.97
844. 1875 CD1 LEU 250 A <--> 15532 ND2 ASN 1019 B 2.91
845. 1875 CD1 LEU 250 A <--> 15533 N TYR 1020 B 2.75
846. 1876 CD2 LEU 250 A <--> 15525 N ASN 1019 B 3.62
847. 1876 CD2 LEU 250 A <--> 15526 CA ASN 1019 B 3.53
848. 1876 CD2 LEU 250 A <--> 15529 CB ASN 1019 B 2.43
849. 1876 CD2 LEU 250 A <--> 15530 CG ASN 1019 B 2.85
850. 1876 CD2 LEU 250 A <--> 15532 ND2 ASN 1019 B 2.44
851. 1876 CD2 LEU 250 A <--> 15539 CD1 TYR 1020 B 3.76
852. 1876 CD2 LEU 250 A <--> 15541 CE1 TYR 1020 B 3.81
853. 1877 N ASN 251 A <--> 15530 CG ASN 1019 B 3.58
854. 1877 N ASN 251 A <--> 15531 OD1 ASN 1019 B 3.64
855. 1877 N ASN 251 A <--> 15532 ND2 ASN 1019 B 3.14
856. 1878 CA ASN 251 A <--> 15532 ND2 ASN 1019 B 3.85
857. 1885 N PRO 252 A <--> 15531 OD1 ASN 1019 B 3.50
858. 1886 CA PRO 252 A <--> 15531 OD1 ASN 1019 B 3.86
859. 1887 C PRO 252 A <--> 15531 OD1 ASN 1019 B 3.14
860. 1888 O PRO 252 A <--> 15530 CG ASN 1019 B 3.06
861. 1888 O PRO 252 A <--> 15531 OD1 ASN 1019 B 1.96
862. 1888 O PRO 252 A <--> 15532 ND2 ASN 1019 B 3.63
863. 1896 CB ASP 253 A <--> 19186 CZ ARG 1500 B 3.88
864. 1896 CB ASP 253 A <--> 19188 NH2 ARG 1500 B 3.90
865. 1897 CG ASP 253 A <--> 19183 CG ARG 1500 B 3.03
866. 1897 CG ASP 253 A <--> 19184 CD ARG 1500 B 3.85
867. 1897 CG ASP 253 A <--> 19185 NE ARG 1500 B 3.45
868. 1898 OD1 ASP 253 A <--> 19183 CG ARG 1500 B 3.73
869. 1899 OD2 ASP 253 A <--> 19179 CA ARG 1500 B 3.85
870. 1899 OD2 ASP 253 A <--> 19182 CB ARG 1500 B 3.00
871. 1899 OD2 ASP 253 A <--> 19183 CG ARG 1500 B 1.96
872. 1899 OD2 ASP 253 A <--> 19184 CD ARG 1500 B 3.12
873. 1899 OD2 ASP 253 A <--> 19185 NE ARG 1500 B 3.17

874. 1899 OD2 ASP 253 A <--> 19186 CZ ARG 1500 B 3.70
875. 1904 CB PRO 254 A <--> 15520 O ASN 1018 B 3.39
876. 1905 CG PRO 254 A <--> 15518 CA ASN 1018 B 3.65
877. 1905 CG PRO 254 A <--> 15519 C ASN 1018 B 2.78
878. 1905 CG PRO 254 A <--> 15520 O ASN 1018 B 2.25
879. 1905 CG PRO 254 A <--> 15521 CB ASN 1018 B 3.52
880. 1905 CG PRO 254 A <--> 15525 N ASN 1019 B 3.36
881. 1905 CG PRO 254 A <--> 15526 CA ASN 1019 B 3.43
882. 1906 CD PRO 254 A <--> 15518 CA ASN 1018 B 3.78
883. 1906 CD PRO 254 A <--> 15519 C ASN 1018 B 3.28
884. 1906 CD PRO 254 A <--> 15520 O ASN 1018 B 3.30
885. 1906 CD PRO 254 A <--> 15521 CB ASN 1018 B 3.05
886. 1906 CD PRO 254 A <--> 15525 N ASN 1019 B 3.62
887. 1906 CD PRO 254 A <--> 15531 OD1 ASN 1019 B 3.75
888. 1908 CA ALA 255 A <--> 19166 NZ LYS 1498 B 3.47
889. 1909 C ALA 255 A <--> 19164 CD LYS 1498 B 3.57
890. 1909 C ALA 255 A <--> 19165 CE LYS 1498 B 3.33
891. 1909 C ALA 255 A <--> 19166 NZ LYS 1498 B 2.17
892. 1910 O ALA 255 A <--> 19164 CD LYS 1498 B 3.57
893. 1910 O ALA 255 A <--> 19165 CE LYS 1498 B 2.97
894. 1910 O ALA 255 A <--> 19166 NZ LYS 1498 B 1.94
895. 1911 CB ALA 255 A <--> 19164 CD LYS 1498 B 3.30
896. 1911 CB ALA 255 A <--> 19166 NZ LYS 1498 B 3.69
897. 1911 CB ALA 255 A <--> 19185 NE ARG 1500 B 3.62
898. 1912 N GLU 256 A <--> 19164 CD LYS 1498 B 3.88
899. 1912 N GLU 256 A <--> 19165 CE LYS 1498 B 3.55
900. 1912 N GLU 256 A <--> 19166 NZ LYS 1498 B 2.22
901. 1913 CA GLU 256 A <--> 19165 CE LYS 1498 B 3.29
902. 1913 CA GLU 256 A <--> 19166 NZ LYS 1498 B 1.96
903. 1914 C GLU 256 A <--> 19166 NZ LYS 1498 B 2.96
904. 1915 O GLU 256 A <--> 19166 NZ LYS 1498 B 3.83
905. 1916 CB GLU 256 A <--> 19166 NZ LYS 1498 B 3.07
906. 1916 CB GLU 256 A <--> 19188 NH2 ARG 1500 B 3.15
907. 1917 CG GLU 256 A <--> 19164 CD LYS 1498 B 3.78
908. 1917 CG GLU 256 A <--> 19165 CE LYS 1498 B 3.75
909. 1917 CG GLU 256 A <--> 19166 NZ LYS 1498 B 3.25
910. 1917 CG GLU 256 A <--> 19185 NE ARG 1500 B 3.76
911. 1917 CG GLU 256 A <--> 19186 CZ ARG 1500 B 3.04
912. 1917 CG GLU 256 A <--> 19188 NH2 ARG 1500 B 1.70
913. 1918 CD GLU 256 A <--> 19186 CZ ARG 1500 B 2.82
914. 1918 CD GLU 256 A <--> 19187 NH1 ARG 1500 B 3.33
915. 1918 CD GLU 256 A <--> 19188 NH2 ARG 1500 B 1.67
916. 1919 OE1 GLU 256 A <--> 19186 CZ ARG 1500 B 3.55
917. 1919 OE1 GLU 256 A <--> 19188 NH2 ARG 1500 B 2.64
918. 1920 OE2 GLU 256 A <--> 19186 CZ ARG 1500 B 2.69
919. 1920 OE2 GLU 256 A <--> 19187 NH1 ARG 1500 B 2.81
920. 1920 OE2 GLU 256 A <--> 19188 NH2 ARG 1500 B 1.86
921. 1921 N GLY 257 A <--> 19166 NZ LYS 1498 B 3.47
922. 2205 N PRO 296 A <--> 16378 O CYS 1130 B 3.82
923. 2205 N PRO 296 A <--> 16379 CB CYS 1130 B 3.83
924. 2205 N PRO 296 A <--> 16397 ND2 ASN 1132 B 3.63
925. 2206 CA PRO 296 A <--> 16376 CA CYS 1130 B 3.64
926. 2206 CA PRO 296 A <--> 16377 C CYS 1130 B 3.39
927. 2206 CA PRO 296 A <--> 16378 O CYS 1130 B 2.46
928. 2206 CA PRO 296 A <--> 16379 CB CYS 1130 B 2.84
929. 2206 CA PRO 296 A <--> 16380 SG CYS 1130 B 3.33
930. 2206 CA PRO 296 A <--> 16395 CG ASN 1132 B 3.52

931.	2206	CA	PRO	296	A	<-->	16396	OD1	ASN	1132	B	3.83
932.	2206	CA	PRO	296	A	<-->	16397	ND2	ASN	1132	B	2.82
933.	2207	C	PRO	296	A	<-->	16376	CA	CYS	1130	B	3.08
934.	2207	C	PRO	296	A	<-->	16377	C	CYS	1130	B	2.51
935.	2207	C	PRO	296	A	<-->	16378	O	CYS	1130	B	1.56
936.	2207	C	PRO	296	A	<-->	16379	CB	CYS	1130	B	2.95
937.	2207	C	PRO	296	A	<-->	16380	SG	CYS	1130	B	3.81
938.	2207	C	PRO	296	A	<-->	16381	N	GLN	1131	B	3.67
939.	2207	C	PRO	296	A	<-->	16386	CG	GLN	1131	B	3.20
940.	2207	C	PRO	296	A	<-->	16397	ND2	ASN	1132	B	3.75
941.	2208	O	PRO	296	A	<-->	16376	CA	CYS	1130	B	2.52
942.	2208	O	PRO	296	A	<-->	16377	C	CYS	1130	B	2.35
943.	2208	O	PRO	296	A	<-->	16378	O	CYS	1130	B	1.97
944.	2208	O	PRO	296	A	<-->	16379	CB	CYS	1130	B	2.75
945.	2208	O	PRO	296	A	<-->	16381	N	GLN	1131	B	3.48
946.	2208	O	PRO	296	A	<-->	16386	CG	GLN	1131	B	3.39
947.	2209	CB	PRO	296	A	<-->	16376	CA	CYS	1130	B	3.05
948.	2209	CB	PRO	296	A	<-->	16377	C	CYS	1130	B	3.29
949.	2209	CB	PRO	296	A	<-->	16378	O	CYS	1130	B	2.82
950.	2209	CB	PRO	296	A	<-->	16379	CB	CYS	1130	B	1.76
951.	2209	CB	PRO	296	A	<-->	16380	SG	CYS	1130	B	2.09
952.	2209	CB	PRO	296	A	<-->	16395	CG	ASN	1132	B	3.79
953.	2209	CB	PRO	296	A	<-->	16397	ND2	ASN	1132	B	2.96
954.	2210	CG	PRO	296	A	<-->	16376	CA	CYS	1130	B	3.81
955.	2210	CG	PRO	296	A	<-->	16379	CB	CYS	1130	B	2.43
956.	2210	CG	PRO	296	A	<-->	16380	SG	CYS	1130	B	3.18
957.	2211	CD	PRO	296	A	<-->	16379	CB	CYS	1130	B	3.75
958.	2212	N	TRP	297	A	<-->	16377	C	CYS	1130	B	2.88
959.	2212	N	TRP	297	A	<-->	16378	O	CYS	1130	B	1.73
960.	2212	N	TRP	297	A	<-->	16381	N	GLN	1131	B	3.69
961.	2212	N	TRP	297	A	<-->	16382	CA	GLN	1131	B	3.75
962.	2212	N	TRP	297	A	<-->	16383	C	GLN	1131	B	3.54
963.	2212	N	TRP	297	A	<-->	16384	O	GLN	1131	B	3.62
964.	2212	N	TRP	297	A	<-->	16385	CB	GLN	1131	B	3.54
965.	2212	N	TRP	297	A	<-->	16386	CG	GLN	1131	B	2.32
966.	2212	N	TRP	297	A	<-->	16387	CD	GLN	1131	B	3.35
967.	2212	N	TRP	297	A	<-->	16389	NE2	GLN	1131	B	3.43
968.	2212	N	TRP	297	A	<-->	16395	CG	ASN	1132	B	3.56
969.	2212	N	TRP	297	A	<-->	16396	OD1	ASN	1132	B	3.19
970.	2212	N	TRP	297	A	<-->	16397	ND2	ASN	1132	B	3.71
971.	2213	CA	TRP	297	A	<-->	16377	C	CYS	1130	B	3.13
972.	2213	CA	TRP	297	A	<-->	16378	O	CYS	1130	B	2.32
973.	2213	CA	TRP	297	A	<-->	16381	N	GLN	1131	B	3.45
974.	2213	CA	TRP	297	A	<-->	16382	CA	GLN	1131	B	3.09
975.	2213	CA	TRP	297	A	<-->	16383	C	GLN	1131	B	3.13
976.	2213	CA	TRP	297	A	<-->	16384	O	GLN	1131	B	3.01
977.	2213	CA	TRP	297	A	<-->	16385	CB	GLN	1131	B	2.40
978.	2213	CA	TRP	297	A	<-->	16386	CG	GLN	1131	B	0.99
979.	2213	CA	TRP	297	A	<-->	16387	CD	GLN	1131	B	2.04
980.	2213	CA	TRP	297	A	<-->	16388	OE1	GLN	1131	B	3.15
981.	2213	CA	TRP	297	A	<-->	16389	NE2	GLN	1131	B	2.44
982.	2213	CA	TRP	297	A	<-->	16396	OD1	ASN	1132	B	3.76
983.	2214	C	TRP	297	A	<-->	16377	C	CYS	1130	B	3.71
984.	2214	C	TRP	297	A	<-->	16378	O	CYS	1130	B	2.89
985.	2214	C	TRP	297	A	<-->	16381	N	GLN	1131	B	3.78
986.	2214	C	TRP	297	A	<-->	16382	CA	GLN	1131	B	3.01
987.	2214	C	TRP	297	A	<-->	16383	C	GLN	1131	B	2.41

988. 2214 C TRP 297 A <--> 16384 O GLN 1131 B 1.83
989. 2214 C TRP 297 A <--> 16385 CB GLN 1131 B 2.61
990. 2214 C TRP 297 A <--> 16386 CG GLN 1131 B 1.79
991. 2214 C TRP 297 A <--> 16387 CD GLN 1131 B 2.79
992. 2214 C TRP 297 A <--> 16388 OE1 GLN 1131 B 3.79
993. 2214 C TRP 297 A <--> 16389 NE2 GLN 1131 B 3.14
994. 2214 C TRP 297 A <--> 16390 N ASN 1132 B 3.37
995. 2214 C TRP 297 A <--> 16391 CA ASN 1132 B 3.85
996. 2214 C TRP 297 A <--> 16395 CG ASN 1132 B 3.70
997. 2214 C TRP 297 A <--> 16396 OD1 ASN 1132 B 2.84
998. 2215 O TRP 297 A <--> 16378 O CYS 1130 B 3.06
999. 2215 O TRP 297 A <--> 16382 CA GLN 1131 B 3.74
1000. 2215 O TRP 297 A <--> 16383 C GLN 1131 B 2.74
1001. 2215 O TRP 297 A <--> 16384 O GLN 1131 B 2.16
1002. 2215 O TRP 297 A <--> 16385 CB GLN 1131 B 3.71
1003. 2215 O TRP 297 A <--> 16386 CG GLN 1131 B 2.92
1004. 2215 O TRP 297 A <--> 16390 N ASN 1132 B 3.24
1005. 2215 O TRP 297 A <--> 16391 CA ASN 1132 B 3.30
1006. 2215 O TRP 297 A <--> 16394 CB ASN 1132 B 3.59
1007. 2215 O TRP 297 A <--> 16395 CG ASN 1132 B 2.65
1008. 2215 O TRP 297 A <--> 16396 OD1 ASN 1132 B 1.65
1009. 2215 O TRP 297 A <--> 16397 ND2 ASN 1132 B 3.53
1010. 2216 CB TRP 297 A <--> 16378 O CYS 1130 B 3.75
1011. 2216 CB TRP 297 A <--> 16385 CB GLN 1131 B 3.25
1012. 2216 CB TRP 297 A <--> 16386 CG GLN 1131 B 1.80
1013. 2216 CB TRP 297 A <--> 16387 CD GLN 1131 B 1.39
1014. 2216 CB TRP 297 A <--> 16388 OE1 GLN 1131 B 2.58
1015. 2216 CB TRP 297 A <--> 16389 NE2 GLN 1131 B 0.96
1016. 2217 CG TRP 297 A <--> 16385 CB GLN 1131 B 3.12
1017. 2217 CG TRP 297 A <--> 16386 CG GLN 1131 B 2.20
1018. 2217 CG TRP 297 A <--> 16387 CD GLN 1131 B 0.85
1019. 2217 CG TRP 297 A <--> 16388 OE1 GLN 1131 B 1.50
1020. 2217 CG TRP 297 A <--> 16389 NE2 GLN 1131 B 0.99
1021. 2218 CD1 TRP 297 A <--> 16386 CG GLN 1131 B 3.24
1022. 2218 CD1 TRP 297 A <--> 16387 CD GLN 1131 B 2.14
1023. 2218 CD1 TRP 297 A <--> 16388 OE1 GLN 1131 B 2.35
1024. 2218 CD1 TRP 297 A <--> 16389 NE2 GLN 1131 B 1.96
1025. 2219 CD2 TRP 297 A <--> 16385 CB GLN 1131 B 3.03
1026. 2219 CD2 TRP 297 A <--> 16386 CG GLN 1131 B 2.66
1027. 2219 CD2 TRP 297 A <--> 16387 CD GLN 1131 B 1.34
1028. 2219 CD2 TRP 297 A <--> 16388 OE1 GLN 1131 B 0.38
1029. 2219 CD2 TRP 297 A <--> 16389 NE2 GLN 1131 B 2.16
1030. 2220 NE1 TRP 297 A <--> 16387 CD GLN 1131 B 2.84
1031. 2220 NE1 TRP 297 A <--> 16388 OE1 GLN 1131 B 2.39
1032. 2220 NE1 TRP 297 A <--> 16389 NE2 GLN 1131 B 3.02
1033. 2221 CE2 TRP 297 A <--> 16385 CB GLN 1131 B 3.83
1034. 2221 CE2 TRP 297 A <--> 16386 CG GLN 1131 B 3.77
1035. 2221 CE2 TRP 297 A <--> 16387 CD GLN 1131 B 2.53
1036. 2221 CE2 TRP 297 A <--> 16388 OE1 GLN 1131 B 1.55
1037. 2221 CE2 TRP 297 A <--> 16389 NE2 GLN 1131 B 3.12
1038. 2222 CE3 TRP 297 A <--> 16385 CB GLN 1131 B 3.09
1039. 2222 CE3 TRP 297 A <--> 16386 CG GLN 1131 B 2.93
1040. 2222 CE3 TRP 297 A <--> 16387 CD GLN 1131 B 2.06
1041. 2222 CE3 TRP 297 A <--> 16388 OE1 GLN 1131 B 1.31
1042. 2222 CE3 TRP 297 A <--> 16389 NE2 GLN 1131 B 3.01
1043. 2223 CZ2 TRP 297 A <--> 16387 CD GLN 1131 B 3.68
1044. 2223 CZ2 TRP 297 A <--> 16388 OE1 GLN 1131 B 2.50

1045. 2223 CZ2 TRP 297 A <--> 19254 CG ASP 1508 B 3.56
1046. 2223 CZ2 TRP 297 A <--> 19255 OD1 ASP 1508 B 3.84
1047. 2223 CZ2 TRP 297 A <--> 19256 OD2 ASP 1508 B 3.44
1048. 2224 CZ3 TRP 297 A <--> 16387 CD GLN 1131 B 3.35
1049. 2224 CZ3 TRP 297 A <--> 16388 OE1 GLN 1131 B 2.32
1050. 2225 CH2 TRP 297 A <--> 16013 OE1 GLN 1081 B 3.88
1051. 2225 CH2 TRP 297 A <--> 16388 OE1 GLN 1131 B 2.76
1052. 2225 CH2 TRP 297 A <--> 19254 CG ASP 1508 B 3.67
1053. 2225 CH2 TRP 297 A <--> 19256 OD2 ASP 1508 B 3.34
1054. 2226 N ALA 298 A <--> 16378 O CYS 1130 B 3.88
1055. 2226 N ALA 298 A <--> 16382 CA GLN 1131 B 2.89
1056. 2226 N ALA 298 A <--> 16383 C GLN 1131 B 2.39
1057. 2226 N ALA 298 A <--> 16384 O GLN 1131 B 1.46
1058. 2226 N ALA 298 A <--> 16385 CB GLN 1131 B 2.32
1059. 2226 N ALA 298 A <--> 16386 CG GLN 1131 B 2.16
1060. 2226 N ALA 298 A <--> 16387 CD GLN 1131 B 2.84
1061. 2226 N ALA 298 A <--> 16388 OE1 GLN 1131 B 3.50
1062. 2226 N ALA 298 A <--> 16389 NE2 GLN 1131 B 3.45
1063. 2226 N ALA 298 A <--> 16390 N ASN 1132 B 3.60
1064. 2226 N ALA 298 A <--> 16396 OD1 ASN 1132 B 3.73
1065. 2227 CA ALA 298 A <--> 16382 CA GLN 1131 B 3.71
1066. 2227 CA ALA 298 A <--> 16383 C GLN 1131 B 2.78
1067. 2227 CA ALA 298 A <--> 16384 O GLN 1131 B 1.56
1068. 2227 CA ALA 298 A <--> 16385 CB GLN 1131 B 3.52
1069. 2227 CA ALA 298 A <--> 16386 CG GLN 1131 B 3.56
1070. 2227 CA ALA 298 A <--> 16390 N ASN 1132 B 3.71
1071. 2227 CA ALA 298 A <--> 16391 CA ASN 1132 B 3.78
1072. 2227 CA ALA 298 A <--> 16396 OD1 ASN 1132 B 3.68
1073. 2228 C ALA 298 A <--> 16384 O GLN 1131 B 2.81
1074. 2228 C ALA 298 A <--> 16396 OD1 ASN 1132 B 3.66
1075. 2229 O ALA 298 A <--> 16384 O GLN 1131 B 3.65
1076. 2230 CB ALA 298 A <--> 15999 OD2 ASP 1079 B 3.50
1077. 2230 CB ALA 298 A <--> 16383 C GLN 1131 B 3.66
1078. 2230 CB ALA 298 A <--> 16384 O GLN 1131 B 2.66
1079. 2231 N GLU 299 A <--> 16384 O GLN 1131 B 3.46
1080. 2231 N GLU 299 A <--> 16396 OD1 ASN 1132 B 3.41
1081. 2246 CD GLU 300 A <--> 17118 CA PRO 1225 B 3.84
1082. 2246 CD GLU 300 A <--> 17119 C PRO 1225 B 3.87
1083. 2246 CD GLU 300 A <--> 17121 CB PRO 1225 B 2.74
1084. 2246 CD GLU 300 A <--> 17122 CG PRO 1225 B 3.52
1085. 2247 OE1 GLU 300 A <--> 17118 CA PRO 1225 B 3.59
1086. 2247 OE1 GLU 300 A <--> 17119 C PRO 1225 B 3.58
1087. 2247 OE1 GLU 300 A <--> 17120 O PRO 1225 B 3.29
1088. 2247 OE1 GLU 300 A <--> 17121 CB PRO 1225 B 2.78
1089. 2248 OE2 GLU 300 A <--> 17118 CA PRO 1225 B 3.29
1090. 2248 OE2 GLU 300 A <--> 17119 C PRO 1225 B 3.53
1091. 2248 OE2 GLU 300 A <--> 17121 CB PRO 1225 B 2.07
1092. 2248 OE2 GLU 300 A <--> 17122 CG PRO 1225 B 2.40
1093. 2248 OE2 GLU 300 A <--> 17123 CD PRO 1225 B 3.84
1094. 2382 CB ASP 318 A <--> 17953 SG CYS 1336 B 3.59
1095. 2383 CG ASP 318 A <--> 17952 CB CYS 1336 B 3.31
1096. 2383 CG ASP 318 A <--> 17953 SG CYS 1336 B 2.84
1097. 2384 OD1 ASP 318 A <--> 17952 CB CYS 1336 B 2.73
1098. 2384 OD1 ASP 318 A <--> 17953 SG CYS 1336 B 3.01
1099. 2385 OD2 ASP 318 A <--> 17952 CB CYS 1336 B 3.79
1100. 2385 OD2 ASP 318 A <--> 17953 SG CYS 1336 B 2.88
1101. 2852 CD ARG 380 A <--> 19406 O SER 1529 B 3.80

1102.	2853	NE	ARG	380	A	<-->	19405	C	SER	1529	B	3.73
1103.	2853	NE	ARG	380	A	<-->	19406	O	SER	1529	B	2.89
1104.	2854	CZ	ARG	380	A	<-->	19405	C	SER	1529	B	3.53
1105.	2854	CZ	ARG	380	A	<-->	19406	O	SER	1529	B	2.52
1106.	2854	CZ	ARG	380	A	<-->	19409	OXT	SER	1529	B	3.85
1107.	2855	NH1	ARG	380	A	<-->	19405	C	SER	1529	B	3.87
1108.	2855	NH1	ARG	380	A	<-->	19406	O	SER	1529	B	3.13
1109.	2855	NH1	ARG	380	A	<-->	19409	OXT	SER	1529	B	3.82
1110.	2856	NH2	ARG	380	A	<-->	19393	O	CYS	1527	B	3.24
1111.	2856	NH2	ARG	380	A	<-->	19399	O	VAL	1528	B	3.67
1112.	2856	NH2	ARG	380	A	<-->	19405	C	SER	1529	B	3.74
1113.	2856	NH2	ARG	380	A	<-->	19406	O	SER	1529	B	2.51
1114.	3873	CG	LEU	513	A	<-->	17960	ND1	HIS	1337	B	3.12
1115.	3873	CG	LEU	513	A	<-->	17962	CE1	HIS	1337	B	2.81
1116.	3874	CD1	LEU	513	A	<-->	17959	CG	HIS	1337	B	3.73
1117.	3874	CD1	LEU	513	A	<-->	17960	ND1	HIS	1337	B	2.49
1118.	3874	CD1	LEU	513	A	<-->	17962	CE1	HIS	1337	B	2.49
1119.	3874	CD1	LEU	513	A	<-->	17963	NE2	HIS	1337	B	3.68
1120.	3875	CD2	LEU	513	A	<-->	17960	ND1	HIS	1337	B	3.80
1121.	3875	CD2	LEU	513	A	<-->	17962	CE1	HIS	1337	B	2.92
1122.	3875	CD2	LEU	513	A	<-->	17963	NE2	HIS	1337	B	3.86
1123.	6919	O	VAL	929	A	<-->	17962	CE1	HIS	1337	B	3.08
1124.	6919	O	VAL	929	A	<-->	17963	NE2	HIS	1337	B	3.16
1125.	6921	CG1	VAL	929	A	<-->	18906	OH	TYR	1462	B	3.66
1126.	6924	CA	PHE	930	A	<-->	17963	NE2	HIS	1337	B	3.41
1127.	6925	C	PHE	930	A	<-->	17961	CD2	HIS	1337	B	3.25
1128.	6925	C	PHE	930	A	<-->	17962	CE1	HIS	1337	B	3.62
1129.	6925	C	PHE	930	A	<-->	17963	NE2	HIS	1337	B	2.48
1130.	6926	O	PHE	930	A	<-->	17957	O	HIS	1337	B	3.89
1131.	6926	O	PHE	930	A	<-->	17959	CG	HIS	1337	B	3.67
1132.	6926	O	PHE	930	A	<-->	17961	CD2	HIS	1337	B	2.33
1133.	6926	O	PHE	930	A	<-->	17962	CE1	HIS	1337	B	3.37
1134.	6926	O	PHE	930	A	<-->	17963	NE2	HIS	1337	B	2.08
1135.	6926	O	PHE	930	A	<-->	19157	OG	SER	1497	B	3.83
1136.	6928	CG	PHE	930	A	<-->	18904	CE2	TYR	1462	B	3.82
1137.	6929	CD1	PHE	930	A	<-->	18904	CE2	TYR	1462	B	3.90
1138.	6929	CD1	PHE	930	A	<-->	18906	OH	TYR	1462	B	3.76
1139.	6930	CD2	PHE	930	A	<-->	18902	CD2	TYR	1462	B	3.37
1140.	6930	CD2	PHE	930	A	<-->	18904	CE2	TYR	1462	B	2.86
1141.	6930	CD2	PHE	930	A	<-->	18905	CZ	TYR	1462	B	3.22
1142.	6930	CD2	PHE	930	A	<-->	18906	OH	TYR	1462	B	3.60
1143.	6931	CE1	PHE	930	A	<-->	18904	CE2	TYR	1462	B	3.09
1144.	6931	CE1	PHE	930	A	<-->	18905	CZ	TYR	1462	B	3.56
1145.	6931	CE1	PHE	930	A	<-->	18906	OH	TYR	1462	B	3.22
1146.	6932	CE2	PHE	930	A	<-->	18900	CG	TYR	1462	B	3.23
1147.	6932	CE2	PHE	930	A	<-->	18901	CD1	TYR	1462	B	3.67
1148.	6932	CE2	PHE	930	A	<-->	18902	CD2	TYR	1462	B	2.21
1149.	6932	CE2	PHE	930	A	<-->	18903	CE1	TYR	1462	B	3.34
1150.	6932	CE2	PHE	930	A	<-->	18904	CE2	TYR	1462	B	1.61
1151.	6932	CE2	PHE	930	A	<-->	18905	CZ	TYR	1462	B	2.39
1152.	6932	CE2	PHE	930	A	<-->	18906	OH	TYR	1462	B	3.03
1153.	6933	CZ	PHE	930	A	<-->	18902	CD2	TYR	1462	B	2.82
1154.	6933	CZ	PHE	930	A	<-->	18903	CE1	TYR	1462	B	3.90
1155.	6933	CZ	PHE	930	A	<-->	18904	CE2	TYR	1462	B	1.80
1156.	6933	CZ	PHE	930	A	<-->	18905	CZ	TYR	1462	B	2.62
1157.	6933	CZ	PHE	930	A	<-->	18906	OH	TYR	1462	B	2.81
1158.	6934	N	ILE	931	A	<-->	17961	CD2	HIS	1337	B	3.88

1159. 6934 N ILE 931 A <--> 17963 NE2 HIS 1337 B 2.99
1160. 6935 CA ILE 931 A <--> 17961 CD2 HIS 1337 B 3.69
1161. 6935 CA ILE 931 A <--> 17963 NE2 HIS 1337 B 3.13
1162. 6936 C ILE 931 A <--> 17959 CG HIS 1337 B 3.59
1163. 6936 C ILE 931 A <--> 17960 ND1 HIS 1337 B 3.73
1164. 6936 C ILE 931 A <--> 17961 CD2 HIS 1337 B 2.68
1165. 6936 C ILE 931 A <--> 17962 CE1 HIS 1337 B 2.98
1166. 6936 C ILE 931 A <--> 17963 NE2 HIS 1337 B 2.21
1167. 6937 O ILE 931 A <--> 17959 CG HIS 1337 B 3.16
1168. 6937 O ILE 931 A <--> 17960 ND1 HIS 1337 B 3.65
1169. 6937 O ILE 931 A <--> 17961 CD2 HIS 1337 B 2.44
1170. 6937 O ILE 931 A <--> 17962 CE1 HIS 1337 B 3.34
1171. 6937 O ILE 931 A <--> 17963 NE2 HIS 1337 B 2.62
1172. 6942 N ASP 932 A <--> 17959 CG HIS 1337 B 3.57
1173. 6942 N ASP 932 A <--> 17960 ND1 HIS 1337 B 3.20
1174. 6942 N ASP 932 A <--> 17961 CD2 HIS 1337 B 2.94
1175. 6942 N ASP 932 A <--> 17962 CE1 HIS 1337 B 2.20
1176. 6942 N ASP 932 A <--> 17963 NE2 HIS 1337 B 1.96
1177. 6943 CA ASP 932 A <--> 17959 CG HIS 1337 B 2.93
1178. 6943 CA ASP 932 A <--> 17960 ND1 HIS 1337 B 2.14
1179. 6943 CA ASP 932 A <--> 17961 CD2 HIS 1337 B 2.88
1180. 6943 CA ASP 932 A <--> 17962 CE1 HIS 1337 B 1.45
1181. 6943 CA ASP 932 A <--> 17963 NE2 HIS 1337 B 2.10
1182. 6944 C ASP 932 A <--> 17960 ND1 HIS 1337 B 3.09
1183. 6944 C ASP 932 A <--> 17962 CE1 HIS 1337 B 2.83
1184. 6944 C ASP 932 A <--> 17963 NE2 HIS 1337 B 3.57
1185. 6945 O ASP 932 A <--> 17960 ND1 HIS 1337 B 3.61
1186. 6945 O ASP 932 A <--> 17962 CE1 HIS 1337 B 3.65
1187. 6946 CB ASP 932 A <--> 17958 CB HIS 1337 B 2.70
1188. 6946 CB ASP 932 A <--> 17959 CG HIS 1337 B 1.50
1189. 6946 CB ASP 932 A <--> 17960 ND1 HIS 1337 B 1.05
1190. 6946 CB ASP 932 A <--> 17961 CD2 HIS 1337 B 1.99
1191. 6946 CB ASP 932 A <--> 17962 CE1 HIS 1337 B 1.48
1192. 6946 CB ASP 932 A <--> 17963 NE2 HIS 1337 B 2.03
1193. 6947 CG ASP 932 A <--> 17955 CA HIS 1337 B 3.30
1194. 6947 CG ASP 932 A <--> 17956 C HIS 1337 B 3.49
1195. 6947 CG ASP 932 A <--> 17957 O HIS 1337 B 2.89
1196. 6947 CG ASP 932 A <--> 17958 CB HIS 1337 B 2.50
1197. 6947 CG ASP 932 A <--> 17959 CG HIS 1337 B 1.04
1198. 6947 CG ASP 932 A <--> 17960 ND1 HIS 1337 B 1.28
1199. 6947 CG ASP 932 A <--> 17961 CD2 HIS 1337 B 1.03
1200. 6947 CG ASP 932 A <--> 17962 CE1 HIS 1337 B 1.41
1201. 6947 CG ASP 932 A <--> 17963 NE2 HIS 1337 B 1.36
1202. 6948 OD1 ASP 932 A <--> 17957 O HIS 1337 B 3.26
1203. 6948 OD1 ASP 932 A <--> 17958 CB HIS 1337 B 3.63
1204. 6948 OD1 ASP 932 A <--> 17959 CG HIS 1337 B 2.25
1205. 6948 OD1 ASP 932 A <--> 17960 ND1 HIS 1337 B 1.93
1206. 6948 OD1 ASP 932 A <--> 17961 CD2 HIS 1337 B 1.94
1207. 6948 OD1 ASP 932 A <--> 17962 CE1 HIS 1337 B 1.31
1208. 6948 OD1 ASP 932 A <--> 17963 NE2 HIS 1337 B 1.36
1209. 6949 OD2 ASP 932 A <--> 17954 N HIS 1337 B 3.88
1210. 6949 OD2 ASP 932 A <--> 17955 CA HIS 1337 B 2.55
1211. 6949 OD2 ASP 932 A <--> 17956 C HIS 1337 B 2.46
1212. 6949 OD2 ASP 932 A <--> 17957 O HIS 1337 B 1.93
1213. 6949 OD2 ASP 932 A <--> 17958 CB HIS 1337 B 1.87
1214. 6949 OD2 ASP 932 A <--> 17959 CG HIS 1337 B 1.06
1215. 6949 OD2 ASP 932 A <--> 17960 ND1 HIS 1337 B 2.24

1216.	6949	OD2	ASP	932	A	<-->	17961	CD2	HIS	1337	B	1.04
1217.	6949	OD2	ASP	932	A	<-->	17962	CE1	HIS	1337	B	2.67
1218.	6949	OD2	ASP	932	A	<-->	17963	NE2	HIS	1337	B	2.25
1219.	6949	OD2	ASP	932	A	<-->	17964	N	LYS	1338	B	3.73
1220.	6949	OD2	ASP	932	A	<-->	17967	O	LYS	1338	B	3.65
1221.	6949	OD2	ASP	932	A	<-->	17981	NZ	LYS	1339	B	3.66
1222.	6950	N	ALA	933	A	<-->	17962	CE1	HIS	1337	B	3.60
1223.	7258	CA	GLN	970	A	<-->	15551	CG2	THR	1021	B	3.59
1224.	7259	C	GLN	970	A	<-->	15549	CB	THR	1021	B	3.21
1225.	7259	C	GLN	970	A	<-->	15550	OG1	THR	1021	B	2.95
1226.	7259	C	GLN	970	A	<-->	15551	CG2	THR	1021	B	2.93
1227.	7260	O	GLN	970	A	<-->	15546	CA	THR	1021	B	3.74
1228.	7260	O	GLN	970	A	<-->	15549	CB	THR	1021	B	2.33
1229.	7260	O	GLN	970	A	<-->	15550	OG1	THR	1021	B	2.23
1230.	7260	O	GLN	970	A	<-->	15551	CG2	THR	1021	B	1.82
1231.	7261	CB	GLN	970	A	<-->	15030	CB	GLN	952	B	3.51
1232.	7261	CB	GLN	970	A	<-->	15031	CG	GLN	952	B	3.82
1233.	7262	CG	GLN	970	A	<-->	15030	CB	GLN	952	B	2.56
1234.	7262	CG	GLN	970	A	<-->	15031	CG	GLN	952	B	2.41
1235.	7262	CG	GLN	970	A	<-->	15032	CD	GLN	952	B	3.39
1236.	7262	CG	GLN	970	A	<-->	15034	NE2	GLN	952	B	3.50
1237.	7262	CG	GLN	970	A	<-->	15302	OD2	ASP	988	B	3.82
1238.	7263	CD	GLN	970	A	<-->	15025	O	GLY	951	B	3.78
1239.	7263	CD	GLN	970	A	<-->	15027	CA	GLN	952	B	3.37
1240.	7263	CD	GLN	970	A	<-->	15028	C	GLN	952	B	3.68
1241.	7263	CD	GLN	970	A	<-->	15029	O	GLN	952	B	3.12
1242.	7263	CD	GLN	970	A	<-->	15030	CB	GLN	952	B	2.12
1243.	7263	CD	GLN	970	A	<-->	15031	CG	GLN	952	B	2.27
1244.	7263	CD	GLN	970	A	<-->	15032	CD	GLN	952	B	3.69
1245.	7263	CD	GLN	970	A	<-->	15302	OD2	ASP	988	B	2.94
1246.	7264	OE1	GLN	970	A	<-->	14856	ND2	ASN	929	B	3.63
1247.	7264	OE1	GLN	970	A	<-->	15025	O	GLY	951	B	3.52
1248.	7264	OE1	GLN	970	A	<-->	15029	O	GLN	952	B	3.55
1249.	7264	OE1	GLN	970	A	<-->	15030	CB	GLN	952	B	3.02
1250.	7264	OE1	GLN	970	A	<-->	15031	CG	GLN	952	B	3.46
1251.	7264	OE1	GLN	970	A	<-->	15302	OD2	ASP	988	B	3.58
1252.	7265	NE2	GLN	970	A	<-->	14855	OD1	ASN	929	B	3.68
1253.	7265	NE2	GLN	970	A	<-->	15024	C	GLY	951	B	3.79
1254.	7265	NE2	GLN	970	A	<-->	15025	O	GLY	951	B	3.41
1255.	7265	NE2	GLN	970	A	<-->	15026	N	GLN	952	B	3.52
1256.	7265	NE2	GLN	970	A	<-->	15027	CA	GLN	952	B	2.59
1257.	7265	NE2	GLN	970	A	<-->	15028	C	GLN	952	B	2.52
1258.	7265	NE2	GLN	970	A	<-->	15029	O	GLN	952	B	1.95
1259.	7265	NE2	GLN	970	A	<-->	15030	CB	GLN	952	B	1.70
1260.	7265	NE2	GLN	970	A	<-->	15031	CG	GLN	952	B	1.53
1261.	7265	NE2	GLN	970	A	<-->	15032	CD	GLN	952	B	3.03
1262.	7265	NE2	GLN	970	A	<-->	15033	OE1	GLN	952	B	3.74
1263.	7265	NE2	GLN	970	A	<-->	15035	N	ASP	953	B	3.78
1264.	7265	NE2	GLN	970	A	<-->	15299	CB	ASP	988	B	3.73
1265.	7265	NE2	GLN	970	A	<-->	15300	CG	ASP	988	B	2.79
1266.	7265	NE2	GLN	970	A	<-->	15301	OD1	ASP	988	B	3.40
1267.	7265	NE2	GLN	970	A	<-->	15302	OD2	ASP	988	B	2.04
1268.	7266	N	ARG	971	A	<-->	15550	OG1	THR	1021	B	3.18
1269.	7267	CA	ARG	971	A	<-->	15549	CB	THR	1021	B	3.55
1270.	7267	CA	ARG	971	A	<-->	15550	OG1	THR	1021	B	2.40
1271.	7268	C	ARG	971	A	<-->	15550	OG1	THR	1021	B	3.36
1272.	7269	O	ARG	971	A	<-->	15550	OG1	THR	1021	B	3.90

1273. 7270 CB ARG 971 A <--> 15550 OG1 THR 1021 B 2.88
1274. 7271 CG ARG 971 A <--> 15549 CB THR 1021 B 3.49
1275. 7271 CG ARG 971 A <--> 15550 OG1 THR 1021 B 2.75
1276. 7272 CD ARG 971 A <--> 15528 O ASN 1019 B 3.65
1277. 7272 CD ARG 971 A <--> 15534 CA TYR 1020 B 3.84
1278. 7272 CD ARG 971 A <--> 15535 C TYR 1020 B 3.80
1279. 7272 CD ARG 971 A <--> 15545 N THR 1021 B 2.89
1280. 7272 CD ARG 971 A <--> 15546 CA THR 1021 B 3.58
1281. 7272 CD ARG 971 A <--> 15549 CB THR 1021 B 3.16
1282. 7272 CD ARG 971 A <--> 15550 OG1 THR 1021 B 2.57
1283. 7273 NE ARG 971 A <--> 15528 O ASN 1019 B 3.69
1284. 7273 NE ARG 971 A <--> 15534 CA TYR 1020 B 3.81
1285. 7273 NE ARG 971 A <--> 15545 N THR 1021 B 3.79
1286. 7274 CZ ARG 971 A <--> 15540 CD2 TYR 1020 B 3.54
1287. 7274 CZ ARG 971 A <--> 15542 CE2 TYR 1020 B 3.89
1288. 7275 NH1 ARG 971 A <--> 15540 CD2 TYR 1020 B 3.67
1289. 7276 NH2 ARG 971 A <--> 15540 CD2 TYR 1020 B 3.54
1290. 7276 NH2 ARG 971 A <--> 15542 CE2 TYR 1020 B 3.38
1291. 7298 NH1 ARG 974 A <--> 19165 CE LYS 1498 B 3.34
1292. 7298 NH1 ARG 974 A <--> 19166 NZ LYS 1498 B 3.02
1293. 7310 SD MET 976 A <--> 19155 O SER 1497 B 3.59
1294. 7311 CE MET 976 A <--> 19152 N SER 1497 B 3.65
1295. 7311 CE MET 976 A <--> 19154 C SER 1497 B 3.72
1296. 7311 CE MET 976 A <--> 19155 O SER 1497 B 3.33
1297. 7319 N PRO 978 A <--> 19150 NH1 ARG 1496 B 3.68
1298. 7320 CA PRO 978 A <--> 19149 CZ ARG 1496 B 3.36
1299. 7320 CA PRO 978 A <--> 19150 NH1 ARG 1496 B 2.44
1300. 7320 CA PRO 978 A <--> 19151 NH2 ARG 1496 B 3.51
1301. 7321 C PRO 978 A <--> 19149 CZ ARG 1496 B 2.97
1302. 7321 C PRO 978 A <--> 19150 NH1 ARG 1496 B 2.50
1303. 7321 C PRO 978 A <--> 19151 NH2 ARG 1496 B 2.64
1304. 7322 O PRO 978 A <--> 19149 CZ ARG 1496 B 2.90
1305. 7322 O PRO 978 A <--> 19150 NH1 ARG 1496 B 2.45
1306. 7322 O PRO 978 A <--> 19151 NH2 ARG 1496 B 2.69
1307. 7323 CB PRO 978 A <--> 19146 CG ARG 1496 B 3.71
1308. 7323 CB PRO 978 A <--> 19147 CD ARG 1496 B 3.88
1309. 7323 CB PRO 978 A <--> 19148 NE ARG 1496 B 3.63
1310. 7323 CB PRO 978 A <--> 19149 CZ ARG 1496 B 2.69
1311. 7323 CB PRO 978 A <--> 19150 NH1 ARG 1496 B 1.68
1312. 7323 CB PRO 978 A <--> 19151 NH2 ARG 1496 B 3.36
1313. 7324 CG PRO 978 A <--> 19136 O LEU 1495 B 3.56
1314. 7324 CG PRO 978 A <--> 19150 NH1 ARG 1496 B 3.15
1315. 7326 N TRP 979 A <--> 19149 CZ ARG 1496 B 3.51
1316. 7326 N TRP 979 A <--> 19150 NH1 ARG 1496 B 3.48
1317. 7326 N TRP 979 A <--> 19151 NH2 ARG 1496 B 2.70
1318. 7327 CA TRP 979 A <--> 19151 NH2 ARG 1496 B 2.76
1319. 7328 C TRP 979 A <--> 19151 NH2 ARG 1496 B 3.88
1320. 7330 CB TRP 979 A <--> 19151 NH2 ARG 1496 B 3.69
1321. 7331 CG TRP 979 A <--> 19151 NH2 ARG 1496 B 3.54
1322. 7333 CD2 TRP 979 A <--> 19149 CZ ARG 1496 B 3.82
1323. 7333 CD2 TRP 979 A <--> 19151 NH2 ARG 1496 B 2.71
1324. 7335 CE2 TRP 979 A <--> 19151 NH2 ARG 1496 B 3.72
1325. 7336 CE3 TRP 979 A <--> 19148 NE ARG 1496 B 3.34
1326. 7336 CE3 TRP 979 A <--> 19149 CZ ARG 1496 B 2.84
1327. 7336 CE3 TRP 979 A <--> 19151 NH2 ARG 1496 B 1.72
1328. 7338 CZ3 TRP 979 A <--> 19148 NE ARG 1496 B 2.67
1329. 7338 CZ3 TRP 979 A <--> 19149 CZ ARG 1496 B 2.77

1330. 7338 CZ3 TRP 979 A <--> 19151 NH2 ARG 1496 B 2.24
1331. 7339 CH2 TRP 979 A <--> 19148 NE ARG 1496 B 3.43
1332. 7339 CH2 TRP 979 A <--> 19149 CZ ARG 1496 B 3.70
1333. 7339 CH2 TRP 979 A <--> 19151 NH2 ARG 1496 B 3.38
1334. 7388 O SER 986 A <--> 15316 CE2 PHE 990 B 3.48
1335. 7388 O SER 986 A <--> 15317 CZ PHE 990 B 3.63
1336. 7392 CA SER 987 A <--> 14889 ND2 ASN 934 B 3.27
1337. 7392 CA SER 987 A <--> 15316 CE2 PHE 990 B 3.49
1338. 7393 C SER 987 A <--> 14887 CG ASN 934 B 3.79
1339. 7393 C SER 987 A <--> 14889 ND2 ASN 934 B 2.91
1340. 7394 O SER 987 A <--> 14889 ND2 ASN 934 B 3.90
1341. 7395 CB SER 987 A <--> 14886 CB ASN 934 B 3.30
1342. 7395 CB SER 987 A <--> 14887 CG ASN 934 B 3.20
1343. 7395 CB SER 987 A <--> 14888 OD1 ASN 934 B 3.86
1344. 7395 CB SER 987 A <--> 14889 ND2 ASN 934 B 3.15
1345. 7395 CB SER 987 A <--> 15316 CE2 PHE 990 B 3.89
1346. 7396 OG SER 987 A <--> 14886 CB ASN 934 B 3.52
1347. 7396 OG SER 987 A <--> 15316 CE2 PHE 990 B 3.73
1348. 7397 N GLN 988 A <--> 14887 CG ASN 934 B 3.29
1349. 7397 N GLN 988 A <--> 14889 ND2 ASN 934 B 2.10
1350. 7397 N GLN 988 A <--> 15314 CD2 PHE 990 B 3.80
1351. 7398 CA GLN 988 A <--> 14889 ND2 ASN 934 B 2.85
1352. 7399 C GLN 988 A <--> 14887 CG ASN 934 B 3.49
1353. 7399 C GLN 988 A <--> 14888 OD1 ASN 934 B 3.45
1354. 7399 C GLN 988 A <--> 14889 ND2 ASN 934 B 2.70
1355. 7400 O GLN 988 A <--> 14868 O GLY 931 B 3.12
1356. 7400 O GLN 988 A <--> 14887 CG ASN 934 B 2.52
1357. 7400 O GLN 988 A <--> 14888 OD1 ASN 934 B 2.45
1358. 7400 O GLN 988 A <--> 14889 ND2 ASN 934 B 1.99
1359. 7401 CB GLN 988 A <--> 14889 ND2 ASN 934 B 3.54
1360. 7403 CD GLN 988 A <--> 17362 CG ASP 1257 B 3.82
1361. 7403 CD GLN 988 A <--> 17363 OD1 ASP 1257 B 3.61
1362. 7403 CD GLN 988 A <--> 17364 OD2 ASP 1257 B 3.28
1363. 7404 OE1 GLN 988 A <--> 15034 NE2 GLN 952 B 3.60
1364. 7404 OE1 GLN 988 A <--> 15308 CA PHE 990 B 3.78
1365. 7404 OE1 GLN 988 A <--> 15311 CB PHE 990 B 3.15
1366. 7404 OE1 GLN 988 A <--> 17360 O ASP 1257 B 3.89
1367. 7404 OE1 GLN 988 A <--> 17362 CG ASP 1257 B 3.08
1368. 7404 OE1 GLN 988 A <--> 17363 OD1 ASP 1257 B 2.57
1369. 7404 OE1 GLN 988 A <--> 17364 OD2 ASP 1257 B 2.95
1370. 7405 NE2 GLN 988 A <--> 17362 CG ASP 1257 B 3.88
1371. 7405 NE2 GLN 988 A <--> 17364 OD2 ASP 1257 B 3.05
1372. 7407 CA ARG 989 A <--> 14867 C GLY 931 B 3.85
1373. 7407 CA ARG 989 A <--> 14868 O GLY 931 B 3.36
1374. 7410 CB ARG 989 A <--> 14868 O GLY 931 B 3.73
1375. 7410 CB ARG 989 A <--> 14875 CG2 THR 932 B 3.83
1376. 7410 CB ARG 989 A <--> 14888 OD1 ASN 934 B 3.83
1377. 7411 CG ARG 989 A <--> 14868 O GLY 931 B 3.45
1378. 7411 CG ARG 989 A <--> 14870 CA THR 932 B 3.47
1379. 7411 CG ARG 989 A <--> 14872 O THR 932 B 3.88
1380. 7411 CG ARG 989 A <--> 14875 CG2 THR 932 B 3.85
1381. 7411 CG ARG 989 A <--> 14887 CG ASN 934 B 3.43
1382. 7411 CG ARG 989 A <--> 14888 OD1 ASN 934 B 2.43
1383. 7411 CG ARG 989 A <--> 14889 ND2 ASN 934 B 3.81
1384. 7412 CD ARG 989 A <--> 14870 CA THR 932 B 3.69
1385. 7412 CD ARG 989 A <--> 14872 O THR 932 B 3.65
1386. 7412 CD ARG 989 A <--> 14873 CB THR 932 B 3.80

1387.	7412	CD	ARG	989	A	<-->	14875	CG2	THR	932	B	3.60
1388.	7412	CD	ARG	989	A	<-->	14888	OD1	ASN	934	B	2.95
1389.	7413	NE	ARG	989	A	<-->	14871	C	THR	932	B	3.79
1390.	7413	NE	ARG	989	A	<-->	14872	O	THR	932	B	2.94
1391.	7413	NE	ARG	989	A	<-->	14886	CB	ASN	934	B	3.86
1392.	7413	NE	ARG	989	A	<-->	14887	CG	ASN	934	B	3.18
1393.	7413	NE	ARG	989	A	<-->	14888	OD1	ASN	934	B	2.31
1394.	7414	CZ	ARG	989	A	<-->	14872	O	THR	932	B	3.53
1395.	7414	CZ	ARG	989	A	<-->	14888	OD1	ASN	934	B	3.45
1396.	7416	NH2	ARG	989	A	<-->	14872	O	THR	932	B	3.71
1397.	7416	NH2	ARG	989	A	<-->	14888	OD1	ASN	934	B	3.80
1398.	7417	N	SER	990	A	<-->	14866	CA	GLY	931	B	3.89
1399.	7422	OG	SER	990	A	<-->	14854	CG	ASN	929	B	3.44
1400.	7422	OG	SER	990	A	<-->	14855	OD1	ASN	929	B	3.87
1401.	7422	OG	SER	990	A	<-->	14856	ND2	ASN	929	B	3.04
1402.	7422	OG	SER	990	A	<-->	14866	CA	GLY	931	B	3.50
1403.	7422	OG	SER	990	A	<-->	15302	OD2	ASP	988	B	3.25
1404.	7431	NE2	GLN	991	A	<-->	14875	CG2	THR	932	B	3.75
1405.	7501	CA	ALA	1001	A	<-->	13496	CD	ARG	754	B	3.50
1406.	7504	CB	ALA	1001	A	<-->	13495	CG	ARG	754	B	3.62
1407.	7504	CB	ALA	1001	A	<-->	13496	CD	ARG	754	B	2.31
1408.	7504	CB	ALA	1001	A	<-->	13497	NE	ARG	754	B	3.14
1409.	7504	CB	ALA	1001	A	<-->	13498	CZ	ARG	754	B	3.88
1410.	7504	CB	ALA	1001	A	<-->	13499	NH1	ARG	754	B	3.86
1411.	7530	CG	ASP	1005	A	<-->	14689	CB	THR	906	B	3.23
1412.	7530	CG	ASP	1005	A	<-->	14691	CG2	THR	906	B	2.81
1413.	7531	OD1	ASP	1005	A	<-->	14689	CB	THR	906	B	2.87
1414.	7531	OD1	ASP	1005	A	<-->	14690	OG1	THR	906	B	3.39
1415.	7531	OD1	ASP	1005	A	<-->	14691	CG2	THR	906	B	2.85
1416.	7532	OD2	ASP	1005	A	<-->	14686	CA	THR	906	B	3.76
1417.	7532	OD2	ASP	1005	A	<-->	14689	CB	THR	906	B	2.90
1418.	7532	OD2	ASP	1005	A	<-->	14691	CG2	THR	906	B	2.11
1419.	7532	OD2	ASP	1005	A	<-->	14875	CG2	THR	932	B	3.77

Salt bridges

	<----- A T O M 1 ----->						<----- A T O M 2 ----->					
	Atom no.	Atom name	Res name	Res no.	Chain		Atom no.	Atom name	Res name	Res no.	Chain	Distance
1.	916	NE	ARG	126	A	<-->	14429	OD2	ASP	872	B	3.13
2.	1603	NH1	ARG	216	A	<-->	16346	OD1	ASP	1126	B	3.45
3.	1899	OD2	ASP	253	A	<-->	19185	NE	ARG	1500	B	3.17
4.	1920	OE2	GLU	256	A	<-->	19188	NH2	ARG	1500	B	1.86
5.	6948	OD1	ASP	932	A	<-->	17963	NE2	HIS	1337	B	1.36
6.	6949	OD2	ASP	932	A	<-->	17981	NZ	LYS	1339	B	3.66
7.	7275	NH1	ARG	971	A	<-->	15682	OD1	ASP	1038	B	3.93

Number of salt bridges: 7

Number of hydrogen bonds: 22

Number of non-bonded contacts: 1419

Table S7. The list of residue-specific RMSF values of the best model after MD simulation of the docked complexes in an explicit solvent system.

[A] ROBO1.IG1 (WT):SLIT2.D2 (WT)

Residue Position	Chain ID	RMSF Values
60	A	6.982
61	A	5.146
62	A	3.957
63	A	3.506
64	A	2.323
65	A	1.729
66	A	0.927
67	A	0.47
68	A	0.475
69	A	0.523
70	A	0.613
71	A	0.654
72	A	0.682
73	A	0.987
74	A	0.721
75	A	1.145
76	A	1.397
77	A	0.913
78	A	1.139
79	A	0.972
80	A	0.941
81	A	1.16
82	A	1.211
83	A	0.821
84	A	0.561
85	A	0.642
86	A	0.661
87	A	0.45
88	A	0.566
89	A	0.506
90	A	0.482
91	A	0.502
92	A	0.546
93	A	0.729
94	A	1.075
95	A	1.716
96	A	1.611
97	A	1.356
98	A	0.901
99	A	0.876
100	A	0.896
101	A	0.818
102	A	0.786
103	A	1.027
104	A	1.402
105	A	1.615

106	A	1.51
107	A	1.384
108	A	2.137
109	A	2.886
110	A	3.624
111	A	3.304
112	A	4.039
113	A	3.467
114	A	4.178
115	A	3.665
116	A	2.464
117	A	1.851
118	A	0.758
119	A	0.448
120	A	0.633
121	A	0.801
122	A	0.931
123	A	1.48
124	A	1.527
125	A	2.136
126	A	0.799
127	A	0.458
128	A	0.398
129	A	0.406
130	A	0.36
131	A	0.532
132	A	0.778
133	A	1.607
134	A	2.646
135	A	4.35
136	A	6.48
137	A	7.663
138	A	5.629
139	A	4.579
140	A	3.866
141	A	2.86
142	A	2.11
143	A	0.882
144	A	0.774
145	A	0.562
146	A	0.472
147	A	0.563
148	A	0.59
149	A	0.468
150	A	0.419
151	A	0.689
152	A	1.836
153	A	1.645
154	A	0.684
155	A	0.332

156	A	0.339
157	A	0.54
158	A	0.921
159	A	0.761
160	A	0.858
161	A	0.605
162	A	0.788
163	A	0.598
164	A	0.846
165	A	0.893
166	A	1.439
269	B	3.446
270	B	1.9
271	B	2.225
272	B	2.61
273	B	2.708
274	B	3.277
275	B	2.636
276	B	2.377
277	B	1.585
278	B	1.488
279	B	1.165
280	B	1.29
281	B	1.139
282	B	0.757
283	B	0.362
284	B	0.514
285	B	0.618
286	B	0.364
287	B	0.334
288	B	0.59
289	B	0.6
290	B	0.698
291	B	0.98
292	B	1.094
293	B	0.506
294	B	0.329
295	B	0.44
296	B	1.089
297	B	1.691
298	B	1.647
299	B	2.3
300	B	2.084
301	B	2.074
302	B	1.122
303	B	0.677
304	B	0.159
305	B	0.181
306	B	0.123
307	B	0.118

308	B	0.305
309	B	0.305
310	B	0.304
311	B	0.557
312	B	0.911
313	B	0.688
314	B	0.788
315	B	0.302
316	B	0.657
317	B	1.474
318	B	1.338
319	B	0.838
320	B	0.667
321	B	0.967
322	B	1.668
323	B	0.983
324	B	0.578
325	B	0.662
326	B	0.236
327	B	0.195
328	B	0.215
329	B	0.142
330	B	0.195
331	B	0.199
332	B	0.536
333	B	0.554
334	B	0.995
335	B	0.97
336	B	0.554
337	B	0.965
338	B	1.013
339	B	0.877
340	B	0.938
341	B	1.12
342	B	1.372
343	B	0.963
344	B	0.725
345	B	0.65
346	B	0.924
347	B	1.517
348	B	1.083
349	B	0.974
350	B	0.804
351	B	0.794
352	B	0.306
353	B	0.3
354	B	0.258
355	B	0.353
356	B	0.362
357	B	0.659

358	B	0.695
359	B	0.937
360	B	0.857
361	B	0.765
362	B	1.205
363	B	1.654
364	B	0.957
365	B	0.854
366	B	0.889
367	B	0.99
368	B	1.049
369	B	0.7
370	B	0.615
371	B	0.533
372	B	0.531
373	B	0.521
374	B	0.463
375	B	0.458
376	B	0.258
377	B	0.117
378	B	0.266
379	B	0.256
380	B	0.244
381	B	0.481
382	B	0.542
383	B	0.814
384	B	0.942
385	B	1.188
386	B	1.685
387	B	1.533
388	B	1.428
389	B	1.477
390	B	1.174
391	B	1.003
392	B	1.085
393	B	0.899
394	B	0.631
395	B	0.577
396	B	0.565
397	B	0.484
398	B	0.688
399	B	0.497
400	B	0.407
401	B	0.477
402	B	0.411
403	B	0.162
404	B	0.154
405	B	0.229
406	B	0.601
407	B	0.687

408	B	1.062
409	B	2.174
410	B	2.955
411	B	2.022
412	B	0.722
413	B	0.682
414	B	1.112
415	B	1.347
416	B	1.763
417	B	2.795
418	B	1.79
419	B	1.519
420	B	2.496
421	B	3.106
422	B	1.174
423	B	0.877
424	B	0.503
425	B	0.606
426	B	0.386
427	B	0.298
428	B	0.538
429	B	0.377
430	B	0.577
431	B	0.706
432	B	0.422
433	B	0.408
434	B	0.624
435	B	0.842
436	B	0.457
437	B	0.344
438	B	0.181
439	B	0.19
440	B	0.182
441	B	0.156
442	B	0.158
443	B	0.169
444	B	0.154
445	B	0.137
446	B	0.209
447	B	0.3
448	B	0.833
449	B	1.368
450	B	1.237
451	B	0.967
452	B	0.862
453	B	0.601
454	B	0.85
455	B	0.742
456	B	0.556
457	B	0.394

458	B	0.782
459	B	1.204
460	B	1.725
461	B	2.027
462	B	2.001
463	B	1.551
464	B	1.646
465	B	1.655
466	B	1.414
467	B	0.755
468	B	0.544
469	B	0.71
470	B	1.071
471	B	0.671
472	B	0.778
473	B	0.475
474	B	1.254
475	B	1.796
476	B	1.475
477	B	1.445
478	B	1.455

[B] G82V (ROBO1.IG1):SLIT2.D2 (WT)

Residue Position	Chain ID	RMSF Values
60	A	7.261
61	A	5.477
62	A	3.512
63	A	1.625
64	A	1.278
65	A	1.854
66	A	0.879
67	A	0.542
68	A	0.245
69	A	0.558
70	A	0.59
71	A	0.594
72	A	0.768
73	A	0.772
74	A	0.683
75	A	0.364
76	A	0.287
77	A	0.334
78	A	0.572
79	A	0.635
80	A	1.092
81	A	1.188
82	A	0.994
83	A	0.594
84	A	0.519
85	A	0.301
86	A	0.294
87	A	0.286
88	A	0.179
89	A	0.331
90	A	0.372
91	A	0.404
92	A	0.681
93	A	0.898
94	A	0.564
95	A	0.598
96	A	0.656
97	A	0.663
98	A	0.476
99	A	0.361
100	A	0.099
101	A	0.1
102	A	0.114
103	A	0.17
104	A	1.025
105	A	1.299
106	A	0.815
107	A	0.614

108	A	0.612
109	A	1.241
110	A	1.036
111	A	1.25
112	A	1.311
113	A	1.61
114	A	1.807
115	A	2.532
116	A	2.06
117	A	1
118	A	0.587
119	A	0.332
120	A	0.161
121	A	0.324
122	A	0.533
123	A	0.82
124	A	1
125	A	0.52
126	A	0.416
127	A	0.209
128	A	0.133
129	A	0.123
130	A	0.259
131	A	0.549
132	A	0.791
133	A	1.306
134	A	1.516
135	A	2.265
136	A	2.827
137	A	2.53
138	A	2.034
139	A	1.678
140	A	2.489
141	A	2.465
142	A	1.904
143	A	0.793
144	A	0.377
145	A	0.186
146	A	0.106
147	A	0.116
148	A	0.156
149	A	0.327
150	A	0.511
151	A	0.466
152	A	0.623
153	A	1.028
154	A	0.361
155	A	0.291
156	A	0.241
157	A	0.231

158	A	0.371
159	A	0.817
160	A	0.378
161	A	0.529
162	A	0.376
163	A	0.352
164	A	0.636
165	A	0.778
166	A	1.61
269	B	3.072
270	B	2.344
271	B	1.587
272	B	1.467
273	B	1.54
274	B	1.436
275	B	1.409
276	B	0.598
277	B	0.61
278	B	0.616
279	B	0.756
280	B	0.71
281	B	1.389
282	B	0.785
283	B	0.178
284	B	0.234
285	B	0.127
286	B	0.306
287	B	0.711
288	B	0.374
289	B	0.343
290	B	0.633
291	B	1.065
292	B	1.71
293	B	1.501
294	B	1.537
295	B	1.843
296	B	1.634
297	B	1.775
298	B	2.079
299	B	2.53
300	B	2.413
301	B	1.634
302	B	0.673
303	B	0.187
304	B	0.119
305	B	0.127
306	B	0.117
307	B	0.139
308	B	0.163
309	B	0.558

310	B	0.95
311	B	1.211
312	B	1.18
313	B	0.739
314	B	0.81
315	B	0.879
316	B	1.257
317	B	1.416
318	B	1.824
319	B	1.751
320	B	0.858
321	B	0.944
322	B	1.457
323	B	1.529
324	B	1.642
325	B	1.235
326	B	0.687
327	B	0.23
328	B	0.108
329	B	0.122
330	B	0.373
331	B	0.353
332	B	0.559
333	B	0.459
334	B	0.919
335	B	0.976
336	B	0.754
337	B	0.663
338	B	0.734
339	B	0.95
340	B	0.931
341	B	0.88
342	B	0.622
343	B	0.79
344	B	0.516
345	B	0.602
346	B	1.584
347	B	1.528
348	B	0.74
349	B	0.856
350	B	0.513
351	B	0.271
352	B	0.369
353	B	0.412
354	B	0.149
355	B	0.392
356	B	0.367
357	B	0.818
358	B	1.602
359	B	1.071

360	B	1.356
361	B	1.432
362	B	1.856
363	B	2.194
364	B	1.544
365	B	1.636
366	B	0.769
367	B	0.527
368	B	0.162
369	B	0.191
370	B	0.23
371	B	0.234
372	B	0.594
373	B	0.467
374	B	0.359
375	B	0.165
376	B	0.093
377	B	0.224
378	B	0.429
379	B	0.399
380	B	0.186
381	B	0.627
382	B	1.076
383	B	1.253
384	B	1.413
385	B	1.522
386	B	1.334
387	B	1.193
388	B	1.082
389	B	1.145
390	B	0.897
391	B	0.726
392	B	0.334
393	B	0.631
394	B	0.5
395	B	0.9
396	B	0.778
397	B	0.222
398	B	0.278
399	B	0.19
400	B	0.229
401	B	0.328
402	B	0.441
403	B	0.293
404	B	0.281
405	B	0.235
406	B	0.898
407	B	1.511
408	B	1.206
409	B	1.618

410	B	1.13
411	B	0.999
412	B	0.917
413	B	0.96
414	B	1.114
415	B	0.947
416	B	0.89
417	B	1.26
418	B	1.728
419	B	0.946
420	B	1.846
421	B	0.742
422	B	0.307
423	B	0.342
424	B	0.292
425	B	0.178
426	B	0.314
427	B	0.353
428	B	0.198
429	B	0.183
430	B	0.324
431	B	0.352
432	B	0.173
433	B	0.277
434	B	1.203
435	B	1.559
436	B	0.62
437	B	0.581
438	B	0.426
439	B	0.419
440	B	0.37
441	B	0.311
442	B	0.295
443	B	0.478
444	B	0.385
445	B	0.323
446	B	0.519
447	B	0.764
448	B	0.91
449	B	1.196
450	B	0.649
451	B	0.316
452	B	0.638
453	B	0.7
454	B	1.305
455	B	0.629
456	B	0.524
457	B	0.443
458	B	0.685
459	B	1.17

460	B	2.4
461	B	2.875
462	B	2.618
463	B	1.715
464	B	2.66
465	B	2.196
466	B	1.253
467	B	0.424
468	B	0.478
469	B	0.84
470	B	1.231
471	B	1.283
472	B	1.423
473	B	0.944
474	B	2.083
475	B	1.975
476	B	1.355
477	B	1.573
478	B	2.178

[C] ROBO1.IG1 (WT):Y323H (SLIT2.D2)

Residue Position	Chain ID	RMSF Values
60	A	3.422
61	A	2.012
62	A	1.017
63	A	1.152
64	A	0.68
65	A	0.806
66	A	0.524
67	A	0.459
68	A	0.271
69	A	0.224
70	A	0.136
71	A	0.408
72	A	0.585
73	A	0.739
74	A	0.618
75	A	0.723
76	A	0.618
77	A	0.334
78	A	0.591
79	A	0.589
80	A	1.488
81	A	2.303
82	A	2.201
83	A	1.149
84	A	0.53
85	A	0.536
86	A	0.297
87	A	0.358
88	A	0.436
89	A	0.563
90	A	0.286
91	A	0.17
92	A	0.155
93	A	0.122
94	A	0.41
95	A	0.577
96	A	0.913
97	A	0.916
98	A	0.896
99	A	0.602
100	A	0.465
101	A	0.203
102	A	0.169
103	A	0.488
104	A	0.891
105	A	1.442
106	A	0.876
107	A	0.489

108	A	1.09
109	A	1.134
110	A	2.301
111	A	2.199
112	A	3.605
113	A	4.066
114	A	4.132
115	A	3.674
116	A	2.706
117	A	2.338
118	A	1.645
119	A	0.829
120	A	0.969
121	A	0.999
122	A	1.352
123	A	3.024
124	A	2.486
125	A	1.379
126	A	1.002
127	A	0.537
128	A	0.535
129	A	0.491
130	A	0.713
131	A	1.019
132	A	0.946
133	A	1.315
134	A	1.551
135	A	2.685
136	A	2.318
137	A	2.661
138	A	2.791
139	A	2.612
140	A	2.856
141	A	2.092
142	A	2.342
143	A	0.919
144	A	0.601
145	A	0.199
146	A	0.144
147	A	0.121
148	A	0.349
149	A	0.124
150	A	0.568
151	A	0.606
152	A	0.755
153	A	1.28
154	A	0.793
155	A	0.535
156	A	0.39
157	A	0.301

158	A	0.669
159	A	0.893
160	A	0.789
161	A	0.575
162	A	0.554
163	A	0.499
164	A	0.572
165	A	0.739
166	A	1.695
269	B	3.079
270	B	1.854
271	B	1.415
272	B	1.13
273	B	0.892
274	B	1.1
275	B	1.407
276	B	0.836
277	B	0.475
278	B	0.276
279	B	0.358
280	B	0.475
281	B	1.199
282	B	0.528
283	B	0.288
284	B	0.163
285	B	0.16
286	B	0.413
287	B	0.316
288	B	0.12
289	B	0.131
290	B	0.388
291	B	1.158
292	B	1.418
293	B	0.458
294	B	0.421
295	B	0.891
296	B	0.744
297	B	0.761
298	B	0.593
299	B	0.776
300	B	0.788
301	B	0.871
302	B	0.547
303	B	0.303
304	B	0.253
305	B	0.096
306	B	0.091
307	B	0.091
308	B	0.101
309	B	0.127

310	B	0.404
311	B	0.693
312	B	0.653
313	B	0.689
314	B	0.446
315	B	0.514
316	B	1.13
317	B	1.231
318	B	1.391
319	B	1.135
320	B	0.836
321	B	1.137
322	B	1.178
323	B	1.112
324	B	0.978
325	B	0.745
326	B	0.538
327	B	0.354
328	B	0.321
329	B	0.275
330	B	0.213
331	B	0.31
332	B	0.453
333	B	0.374
334	B	0.393
335	B	1.133
336	B	0.962
337	B	0.764
338	B	0.965
339	B	0.68
340	B	1.286
341	B	1.954
342	B	1.719
343	B	1.3
344	B	0.849
345	B	1.17
346	B	1.063
347	B	1.034
348	B	0.81
349	B	0.755
350	B	0.413
351	B	0.376
352	B	0.567
353	B	0.36
354	B	0.235
355	B	0.263
356	B	0.139
357	B	0.608
358	B	0.878
359	B	0.915

360	B	0.842
361	B	0.782
362	B	0.741
363	B	0.866
364	B	1.2
365	B	1.507
366	B	1.616
367	B	0.814
368	B	0.587
369	B	1.071
370	B	1.458
371	B	1.063
372	B	1.641
373	B	0.751
374	B	0.528
375	B	0.231
376	B	0.381
377	B	0.305
378	B	0.421
379	B	0.372
380	B	0.324
381	B	0.52
382	B	0.442
383	B	0.558
384	B	0.481
385	B	0.492
386	B	0.659
387	B	0.444
388	B	0.603
389	B	1.473
390	B	2.525
391	B	1.608
392	B	0.693
393	B	0.998
394	B	1.064
395	B	0.753
396	B	0.64
397	B	1.485
398	B	0.421
399	B	0.157
400	B	0.404
401	B	0.205
402	B	0.333
403	B	0.433
404	B	0.576
405	B	0.304
406	B	1.349
407	B	0.86
408	B	0.396
409	B	0.584

410	B	0.722
411	B	0.504
412	B	1
413	B	1.111
414	B	1.286
415	B	1.451
416	B	0.905
417	B	1.081
418	B	1.103
419	B	0.399
420	B	0.506
421	B	0.903
422	B	0.613
423	B	0.351
424	B	0.204
425	B	0.251
426	B	0.429
427	B	0.39
428	B	0.726
429	B	0.423
430	B	0.673
431	B	1.432
432	B	0.399
433	B	0.446
434	B	1.106
435	B	1.072
436	B	0.783
437	B	0.507
438	B	0.508
439	B	0.515
440	B	0.456
441	B	0.364
442	B	0.371
443	B	0.508
444	B	0.399
445	B	0.377
446	B	0.597
447	B	0.652
448	B	1.035
449	B	1.24
450	B	0.831
451	B	0.549
452	B	1.241
453	B	1.001
454	B	1.33
455	B	0.867
456	B	0.67
457	B	0.522
458	B	0.805
459	B	1.024

460	B	2.199
461	B	3.46
462	B	3.358
463	B	2.443
464	B	2.166
465	B	1.962
466	B	1.152
467	B	0.657
468	B	0.539
469	B	0.993
470	B	1.04
471	B	0.966
472	B	1.135
473	B	1.178
474	B	1.645
475	B	1.898
476	B	1.274
477	B	1.421
478	B	1.878

[D] ROBO4.IG1-2 (WT):SLIT2.D2 (WT)

Residue Position	Chain ID	RMSF Values
32	A	1.596
33	A	1.179
34	A	0.802
35	A	1.168
36	A	1.681
37	A	1.136
38	A	1.759
39	A	2.228
40	A	1.773
41	A	2.116
42	A	1.987
43	A	1.83
44	A	1.461
45	A	1.472
46	A	1.17
47	A	1.264
48	A	1.562
49	A	2.033
50	A	1.817
51	A	1.527
52	A	2.369
53	A	3.344
54	A	3.856
55	A	4.049
56	A	3.723
57	A	3.775
58	A	3.299
59	A	3.192
60	A	3.583
61	A	3.482
62	A	2.684
63	A	2.501
64	A	2.727
65	A	3.065
66	A	3.908
67	A	3.476
68	A	2.898
69	A	3.337
70	A	3.135
71	A	3.418
72	A	3.092
73	A	2.874
74	A	2.997
75	A	3.579
76	A	3.721
77	A	3.527
78	A	2.939
79	A	2.084

80	A	1.261
81	A	1.104
82	A	1.238
83	A	1.299
84	A	1.781
85	A	2.026
86	A	2.217
87	A	1.378
88	A	0.786
89	A	0.632
90	A	0.383
91	A	0.601
92	A	1.05
93	A	1.692
94	A	1.821
95	A	1.954
96	A	2.067
97	A	2.676
98	A	2.643
99	A	2.772
100	A	2.555
101	A	3.164
102	A	2.742
103	A	1.83
104	A	1.25
105	A	0.661
106	A	0.915
107	A	1.063
108	A	0.952
109	A	1.063
110	A	1.143
111	A	0.692
112	A	0.365
113	A	0.713
114	A	1.221
115	A	1.487
116	A	1.194
117	A	1
118	A	0.797
119	A	0.694
120	A	0.763
121	A	0.616
122	A	0.392
123	A	0.901
124	A	1.44
125	A	1.653
126	A	1.795
127	A	2.573
128	A	2.538
129	A	2.146

130	A	3.718
131	A	4.993
137	A	1.896
138	A	1.257
139	A	1.356
140	A	2.461
141	A	3.048
142	A	2.448
143	A	3.369
144	A	1.593
145	A	1.403
146	A	1.544
147	A	1.518
148	A	1.051
149	A	1.028
150	A	1.073
151	A	1.177
152	A	1.12
153	A	1.422
154	A	1.692
155	A	2.208
156	A	1.012
157	A	0.291
158	A	0.42
159	A	0.699
160	A	0.732
161	A	0.798
162	A	0.835
163	A	0.947
164	A	1.175
165	A	1.434
166	A	1.324
167	A	1.34
168	A	1.112
169	A	0.703
170	A	0.87
171	A	0.974
172	A	1.418
173	A	3.399
174	A	2.702
175	A	2.589
176	A	1.63
177	A	1.618
178	A	1.301
179	A	1.586
180	A	1.418
181	A	1.781
182	A	2.288
183	A	3.376
184	A	2.214

185	A	1.911
186	A	1.542
187	A	1.11
188	A	0.11
189	A	0.101
190	A	0.106
191	A	0.108
192	A	0.103
193	A	0.112
194	A	0.689
195	A	0.539
196	A	0.348
197	A	0.459
198	A	0.299
199	A	0.432
200	A	0.505
201	A	0.685
202	A	1.126
203	A	1.781
204	A	1.181
205	A	0.587
206	A	0.431
207	A	0.139
208	A	0.134
209	A	0.129
210	A	0.116
211	A	0.104
212	A	0.092
213	A	2.014
214	A	2.215
215	A	1.463
216	A	1.669
217	A	1.726
218	A	1.9
219	A	1.834
220	A	2.699
221	A	3.132
222	A	1.514
223	A	2.402
224	A	4.083
269	B	3.589
270	B	2.315
271	B	0.919
272	B	0.103
273	B	0.091
274	B	0.093
275	B	0.109
276	B	1.034
277	B	0.985
278	B	0.646

279	B	0.082
280	B	0.095
281	B	0.109
282	B	0.098
283	B	0.087
284	B	0.093
285	B	0.551
286	B	0.885
287	B	0.704
288	B	0.679
289	B	0.454
290	B	0.945
291	B	0.848
292	B	1.176
293	B	2.456
294	B	1.522
295	B	1.868
296	B	2.181
297	B	2.347
298	B	1.758
299	B	1.853
300	B	2.153
301	B	1.576
302	B	0.857
303	B	0.249
304	B	0.114
305	B	0.109
306	B	0.412
307	B	0.57
308	B	0.436
309	B	0.538
310	B	0.71
311	B	0.994
312	B	0.687
313	B	0.651
314	B	0.774
315	B	0.569
316	B	0.895
317	B	2.307
318	B	3.015
319	B	2.237
320	B	2.048
321	B	3.312
322	B	2.6
323	B	1.497
324	B	1.229
325	B	0.799
326	B	0.532
327	B	0.624
328	B	0.457

329	B	0.323
330	B	0.232
331	B	0.385
332	B	0.442
333	B	0.677
334	B	0.631
335	B	0.985
336	B	0.828
337	B	0.809
338	B	1.139
339	B	0.735
340	B	0.808
341	B	1.164
342	B	1.191
343	B	0.811
344	B	0.885
345	B	1.199
346	B	1.156
347	B	0.834
348	B	0.771
349	B	0.684
350	B	0.445
351	B	0.474
352	B	0.33
353	B	0.563
354	B	0.46
355	B	0.469
356	B	1.328
357	B	1.229
358	B	1.096
359	B	0.916
360	B	0.666
361	B	0.957
362	B	1.119
363	B	1.273
364	B	1.946
365	B	1.67
366	B	1.265
367	B	0.776
368	B	0.666
369	B	1.11
370	B	1.575
371	B	1.689
372	B	1.597
373	B	0.933
374	B	0.794
375	B	0.596
376	B	0.312
377	B	0.462
378	B	0.411

379	B	0.681
380	B	0.776
381	B	0.913
382	B	1.007
383	B	1.095
384	B	1.127
385	B	0.915
386	B	1.202
387	B	1.216
388	B	1.189
389	B	1.31
390	B	1.328
391	B	1.217
392	B	1.31
393	B	1.709
394	B	1.456
395	B	1.409
396	B	1.403
397	B	0.8
398	B	0.881
399	B	0.765
400	B	0.403
401	B	0.397
402	B	0.811
403	B	0.808
404	B	0.841
405	B	0.875
406	B	0.787
407	B	0.929
408	B	1.113
409	B	1.933
410	B	2.156
411	B	1.823
412	B	2.1
413	B	2.59
414	B	2.248
415	B	2.544
416	B	1.864
417	B	1.632
418	B	2.001
419	B	1.915
420	B	1.728
421	B	1.293
422	B	1.213
423	B	0.862
424	B	0.603
425	B	0.454
426	B	0.504
427	B	0.661
428	B	0.847

429	B	1.03
430	B	0.871
431	B	1.019
432	B	0.621
433	B	0.849
434	B	1.048
435	B	1.443
436	B	1.795
437	B	1.806
438	B	1.15
439	B	0.589
440	B	0.565
441	B	0.727
442	B	0.666
443	B	0.624
444	B	0.64
445	B	0.724
446	B	0.831
447	B	0.789
448	B	1.014
449	B	2.09
450	B	1.126
451	B	0.82
452	B	1.26
453	B	1.285
454	B	1.517
455	B	0.988
456	B	0.817
457	B	0.854
458	B	1.137
459	B	1.299
460	B	1.82
461	B	2.667
462	B	2.843
463	B	1.557
464	B	1.8
465	B	2.369
466	B	1.131
467	B	0.775
468	B	0.815
469	B	1.036
470	B	1.375
471	B	1.449
472	B	1.66
473	B	0.997
474	B	2.192
475	B	3.613
476	B	4.171
477	B	5.559
478	B	6.133

[E] C207S (ROBO4.IG1-2):SLIT2.D2 (WT)

Residue Position	Chain ID	RMSF Values
32	A	2.315
33	A	0.722
34	A	1.118
35	A	0.678
36	A	0.767
37	A	2.168
38	A	2.149
39	A	1.751
40	A	1.931
41	A	1.767
42	A	1.881
43	A	2.116
44	A	2.415
45	A	1.989
46	A	1.169
47	A	0.945
48	A	1.158
49	A	0.778
50	A	0.783
51	A	0.672
52	A	0.785
53	A	0.749
54	A	1.13
55	A	1.309
56	A	2.094
57	A	1.66
58	A	1.409
59	A	1.625
60	A	1.272
61	A	1.234
62	A	0.7
63	A	0.309
64	A	0.327
65	A	0.433
66	A	0.442
67	A	1.242
68	A	1.397
69	A	1.61
70	A	1.625
71	A	2.77
72	A	2.266
73	A	2.552
74	A	1.245
75	A	1.024
76	A	1.246
77	A	0.936
78	A	0.987
79	A	0.92

80	A	0.89
81	A	1.437
82	A	1.434
83	A	1.798
84	A	2.374
85	A	2.263
86	A	1.43
87	A	1.311
88	A	0.362
89	A	0.297
90	A	0.808
91	A	0.736
92	A	0.855
93	A	0.927
94	A	1.107
95	A	0.775
96	A	0.922
97	A	1.43
98	A	0.831
99	A	0.802
100	A	1.205
101	A	0.994
102	A	0.714
103	A	0.776
104	A	0.809
105	A	0.229
106	A	0.187
107	A	0.23
108	A	0.507
109	A	0.33
110	A	0.556
111	A	0.504
112	A	0.54
113	A	0.66
114	A	0.889
115	A	1.383
116	A	1.008
117	A	0.645
118	A	0.294
119	A	0.251
120	A	0.362
121	A	0.224
122	A	0.317
123	A	1.574
124	A	1.464
125	A	1.51
126	A	1.562
127	A	2.265
128	A	2.325
129	A	1.796

130	A	1.611
131	A	3.729
137	A	0.788
138	A	0.596
139	A	0.216
140	A	0.233
141	A	0.267
142	A	1.608
143	A	1.957
144	A	2.029
145	A	2.653
146	A	1.817
147	A	1.278
148	A	1.037
149	A	1.435
150	A	1.157
151	A	1.515
152	A	1.417
153	A	1.735
154	A	1.075
155	A	0.438
156	A	0.266
157	A	0.242
158	A	0.244
159	A	0.354
160	A	0.499
161	A	0.854
162	A	0.804
163	A	1.109
164	A	1.309
165	A	1.89
166	A	1.861
167	A	1.987
168	A	1.57
169	A	1.287
170	A	1.46
171	A	1.185
172	A	1.306
173	A	1.297
174	A	1.29
175	A	0.958
176	A	0.992
177	A	0.649
178	A	0.601
179	A	1.073
180	A	1.085
181	A	0.967
182	A	0.708
183	A	0.869
184	A	0.857

185	A	2.359
186	A	1.579
187	A	1.993
188	A	1.512
189	A	1.654
190	A	1.542
191	A	1.354
192	A	1.197
193	A	1.249
194	A	1.431
195	A	1.315
196	A	1.022
197	A	0.941
198	A	0.885
199	A	0.931
200	A	1.071
201	A	1.309
202	A	1.084
203	A	1.431
204	A	1.488
205	A	1.507
206	A	2.162
207	A	2.312
208	A	1.951
209	A	2.152
210	A	2.029
211	A	2.112
212	A	2.346
213	A	2.51
214	A	2.428
215	A	2.185
216	A	2.07
217	A	1.79
218	A	2.305
219	A	1.948
220	A	2.37
221	A	2.786
222	A	2.708
223	A	4.04
224	A	5.657
269	B	5.488
270	B	4.335
271	B	3.811
272	B	3.993
273	B	2.76
274	B	2.476
275	B	2.832
276	B	1.652
277	B	0.821
278	B	0.459

279	B	0.548
280	B	0.798
281	B	1.223
282	B	0.613
283	B	0.343
284	B	0.36
285	B	0.402
286	B	0.427
287	B	0.414
288	B	0.194
289	B	0.798
290	B	1.488
291	B	0.675
292	B	0.801
293	B	0.558
294	B	0.905
295	B	1.607
296	B	1.741
297	B	2.361
298	B	2.389
299	B	2.806
300	B	2.516
301	B	1.876
302	B	0.97
303	B	0.618
304	B	0.388
305	B	0.346
306	B	0.393
307	B	0.268
308	B	0.133
309	B	0.138
310	B	0.268
311	B	1.272
312	B	0.831
313	B	0.608
314	B	0.632
315	B	0.541
316	B	0.86
317	B	1.473
318	B	1.665
319	B	0.983
320	B	0.672
321	B	0.999
322	B	1.981
323	B	1.803
324	B	2.347
325	B	1.345
326	B	1.4
327	B	0.453
328	B	0.321

329	B	0.398
330	B	0.389
331	B	0.279
332	B	0.12
333	B	0.129
334	B	0.373
335	B	0.385
336	B	0.515
337	B	0.612
338	B	0.574
339	B	0.628
340	B	1.09
341	B	2.367
342	B	2.584
343	B	2.846
344	B	1.856
345	B	1.787
346	B	2.063
347	B	1.753
348	B	1.257
349	B	0.998
350	B	0.675
351	B	0.437
352	B	0.218
353	B	0.4
354	B	0.583
355	B	0.423
356	B	0.429
357	B	0.733
358	B	1.273
359	B	0.708
360	B	1.067
361	B	0.812
362	B	1.116
363	B	1.1
364	B	1.253
365	B	1.067
366	B	0.867
367	B	1.001
368	B	0.444
369	B	0.51
370	B	1.005
371	B	0.786
372	B	0.922
373	B	0.932
374	B	0.672
375	B	0.444
376	B	0.341
377	B	0.141
378	B	0.148

379	B	0.484
380	B	0.36
381	B	0.94
382	B	1.022
383	B	1.658
384	B	1.305
385	B	1.859
386	B	1.72
387	B	1.107
388	B	0.831
389	B	0.638
390	B	0.796
391	B	0.611
392	B	0.532
393	B	0.695
394	B	0.871
395	B	0.613
396	B	0.663
397	B	0.684
398	B	0.413
399	B	0.563
400	B	0.256
401	B	0.199
402	B	0.115
403	B	0.533
404	B	0.252
405	B	0.235
406	B	0.703
407	B	1.493
408	B	1.358
409	B	2.243
410	B	1.098
411	B	1.029
412	B	1.073
413	B	1.132
414	B	1.098
415	B	0.75
416	B	0.659
417	B	1.046
418	B	1.025
419	B	0.831
420	B	1.747
421	B	1.012
422	B	0.364
423	B	0.345
424	B	0.371
425	B	0.168
426	B	0.137
427	B	0.139
428	B	0.153

429	B	0.293
430	B	0.427
431	B	0.666
432	B	0.475
433	B	0.635
434	B	0.651
435	B	0.609
436	B	0.687
437	B	0.495
438	B	0.399
439	B	0.33
440	B	0.33
441	B	0.305
442	B	0.346
443	B	0.378
444	B	0.334
445	B	0.441
446	B	0.455
447	B	0.65
448	B	1.055
449	B	0.782
450	B	0.769
451	B	0.567
452	B	0.553
453	B	0.594
454	B	0.69
455	B	0.669
456	B	0.697
457	B	0.642
458	B	0.667
459	B	0.793
460	B	0.926
461	B	0.991
462	B	1.639
463	B	2.537
464	B	2.071
465	B	0.933
466	B	0.594
467	B	0.531
468	B	0.519
469	B	0.671
470	B	0.765
471	B	0.709
472	B	1.015
473	B	0.965
474	B	1.807
475	B	1.77
476	B	1.327
477	B	2.704
478	B	3.396

[F] ROBO4.IG1-2 (WT):S352L (SLIT2.D2)

Residue Position	Chain ID	RMSF Values
32	A	4.373
33	A	4.172
34	A	2.753
35	A	4.102
36	A	2.099
37	A	2.304
38	A	1.55
39	A	2.107
40	A	3.458
41	A	3.372
42	A	2.295
43	A	1.877
44	A	1.944
45	A	1.373
46	A	1.406
47	A	1.253
48	A	1.249
49	A	1.536
50	A	1.367
51	A	1.393
52	A	1.51
53	A	1.823
54	A	1.603
55	A	2.194
56	A	2.325
57	A	2.797
58	A	2.249
59	A	3.381
60	A	4.934
61	A	6.326
62	A	7.946
63	A	6.96
64	A	6.36
65	A	4.932
66	A	6.048
67	A	5.749
68	A	4.749
69	A	5.153
70	A	4.411
71	A	4.422
72	A	3.331
73	A	3.201
74	A	2.008
75	A	1.907
76	A	1.819
77	A	1.621
78	A	1.349

79	A	1.409
80	A	1.247
81	A	1.24
82	A	1.202
83	A	1.232
84	A	1.476
85	A	2.687
86	A	2.522
87	A	0.871
88	A	0.753
89	A	0.708
90	A	0.671
91	A	0.786
92	A	0.94
93	A	0.821
94	A	0.961
95	A	0.825
96	A	1.814
97	A	3.556
98	A	3.169
99	A	2.817
100	A	1.786
101	A	1.59
102	A	1.315
103	A	0.99
104	A	0.912
105	A	0.695
106	A	0.631
107	A	0.658
108	A	0.885
109	A	1.02
110	A	1.213
111	A	0.944
112	A	0.704
113	A	1.168
114	A	1.581
115	A	2.687
116	A	3.081
117	A	3.023
118	A	2.479
119	A	1.206
120	A	0.882
121	A	0.981
122	A	1.087
123	A	1.107
124	A	1.504
125	A	1.825
126	A	1.671
127	A	1.672
128	A	2.295

129	A	2.62
130	A	3.439
131	A	3.991
137	A	1.81
138	A	1.14
139	A	1.28
140	A	1.024
141	A	1.078
142	A	1.233
143	A	1.913
144	A	2.232
145	A	2.63
146	A	1.958
147	A	2.954
148	A	2.434
149	A	2.312
150	A	2.147
151	A	2.656
152	A	2.481
153	A	2.839
154	A	2.188
155	A	1.482
156	A	1.162
157	A	1.178
158	A	1.109
159	A	1.103
160	A	1.49
161	A	1.5
162	A	1.212
163	A	1.183
164	A	1.069
165	A	1.367
166	A	1.54
167	A	1.458
168	A	1.002
169	A	0.887
170	A	1.14
171	A	1.228
172	A	1.121
173	A	1.502
174	A	1.52
175	A	1.868
176	A	1.783
177	A	1.772
178	A	1.154
179	A	1.112
180	A	1.026
181	A	1.066
182	A	1.346
183	A	2.054

184	A	1.47
185	A	1.756
186	A	1.747
187	A	1.626
188	A	2.511
189	A	2.382
190	A	1.787
191	A	1.493
192	A	1.971
193	A	0.987
194	A	0.993
195	A	0.857
196	A	0.585
197	A	0.542
198	A	0.66
199	A	0.624
200	A	0.985
201	A	1.622
202	A	2.555
203	A	3.07
204	A	2.734
205	A	2.001
206	A	2.21
207	A	2.603
208	A	1.871
209	A	1.069
210	A	0.879
211	A	1.72
212	A	1.802
213	A	2.443
214	A	3.183
215	A	2.112
216	A	2.137
217	A	1.395
218	A	1.763
219	A	1.937
220	A	2.731
221	A	2.435
222	A	1.097
223	A	0.95
224	A	2.248
269	B	2.82
270	B	2.106
271	B	1.796
272	B	1.369
273	B	1.397
274	B	1.751
275	B	1.616
276	B	1.163
277	B	0.523

278	B	0.537
279	B	0.515
280	B	0.484
281	B	0.961
282	B	0.594
283	B	0.314
284	B	0.282
285	B	0.234
286	B	0.554
287	B	0.882
288	B	0.655
289	B	1.224
290	B	0.829
291	B	1.495
292	B	1.404
293	B	1.281
294	B	0.929
295	B	1.304
296	B	1.844
297	B	1.849
298	B	1.791
299	B	1.542
300	B	1.486
301	B	1.712
302	B	0.889
303	B	0.49
304	B	0.384
305	B	0.294
306	B	0.198
307	B	0.537
308	B	0.977
309	B	0.504
310	B	0.394
311	B	0.38
312	B	0.567
313	B	0.26
314	B	0.238
315	B	0.239
316	B	0.651
317	B	1.349
318	B	1.759
319	B	1.749
320	B	1.095
321	B	0.966
322	B	0.689
323	B	0.905
324	B	0.625
325	B	0.927
326	B	0.564
327	B	0.458

328	B	0.247
329	B	0.202
330	B	0.136
331	B	0.225
332	B	0.362
333	B	0.533
334	B	0.764
335	B	0.702
336	B	0.421
337	B	0.592
338	B	0.551
339	B	0.553
340	B	1.492
341	B	1.317
342	B	1.173
343	B	0.84
344	B	0.679
345	B	0.679
346	B	0.934
347	B	0.662
348	B	0.74
349	B	0.655
350	B	0.453
351	B	0.576
352	B	0.503
353	B	0.269
354	B	0.622
355	B	0.348
356	B	0.36
357	B	0.656
358	B	0.844
359	B	2.132
360	B	2.476
361	B	1.385
362	B	0.859
363	B	1.028
364	B	0.98
365	B	0.814
366	B	0.692
367	B	0.628
368	B	0.581
369	B	1.17
370	B	1.106
371	B	0.849
372	B	0.884
373	B	0.771
374	B	0.779
375	B	0.785
376	B	0.616
377	B	0.515

378	B	0.48
379	B	1.091
380	B	0.688
381	B	0.755
382	B	0.757
383	B	0.812
384	B	0.62
385	B	1.204
386	B	1.656
387	B	1.119
388	B	1.695
389	B	1.084
390	B	0.767
391	B	0.767
392	B	0.747
393	B	0.945
394	B	0.98
395	B	0.533
396	B	0.606
397	B	0.526
398	B	0.693
399	B	0.48
400	B	0.428
401	B	0.352
402	B	0.467
403	B	0.449
404	B	0.359
405	B	0.503
406	B	0.389
407	B	0.711
408	B	0.722
409	B	1.014
410	B	1.635
411	B	1.664
412	B	2.943
413	B	2.127
414	B	2.211
415	B	2.985
416	B	3.205
417	B	3.024
418	B	2.268
419	B	1.627
420	B	2.134
421	B	1.238
422	B	0.88
423	B	0.833
424	B	0.512
425	B	0.398
426	B	0.597
427	B	0.565

428	B	0.526
429	B	0.441
430	B	0.425
431	B	0.691
432	B	0.843
433	B	0.866
434	B	0.781
435	B	1.06
436	B	1.264
437	B	1.191
438	B	0.859
439	B	0.775
440	B	0.818
441	B	0.614
442	B	0.421
443	B	0.476
444	B	0.439
445	B	0.518
446	B	0.709
447	B	0.584
448	B	1.056
449	B	0.936
450	B	0.903
451	B	0.635
452	B	0.712
453	B	0.88
454	B	0.696
455	B	0.596
456	B	0.625
457	B	0.54
458	B	0.778
459	B	1.063
460	B	1.916
461	B	2.668
462	B	2.794
463	B	2.87
464	B	3.046
465	B	2.714
466	B	0.969
467	B	0.673
468	B	0.567
469	B	0.932
470	B	0.913
471	B	1.013
472	B	1.117
473	B	0.865
474	B	1.758
475	B	2.128
476	B	1.442
477	B	1.93

478	B	1.64
